# Rollback, scissor-like closure of the Mongol-Okhotsk Ocean and formation of an orocline: magmatic migration based on a large archive of age data

**DOI:** 10.1093/nsr/nwab210

**Published:** 2021-11-29

**Authors:** Tao Wang, Ying Tong, Wenjiao Xiao, Lei Guo, Brian F Windley, Tatiana Donskaya, Shan Li, Narantsetseg Tserendash, Jianjun Zhang

**Affiliations:** Beijing SHRIMP Center, Institute of Geology, Chinese Academy of Geological Sciences, Beijing 100037, China; Key Laboratory of Earth Probe and Geodynamics, Chinese Academy of Geological Sciences, Beijing 100037, China; Beijing SHRIMP Center, Institute of Geology, Chinese Academy of Geological Sciences, Beijing 100037, China; Xinjiang Research Center for Mineral Resources, Xinjiang Institute of Ecology and Geography, Chinese Academy of Sciences, Urumqi 830011, China; Key Laboratory of Earth Probe and Geodynamics, Chinese Academy of Geological Sciences, Beijing 100037, China; School of Geography, Geology and the Environment, University of Leicester, Leicester LE1 7RH, UK; Institute of the Earth's Crust, Siberian Branch, Russian Academy of Sciences, Irkutsk 664033, Russia; Key Laboratory of Earth Probe and Geodynamics, Chinese Academy of Geological Sciences, Beijing 100037, China; Institute of Geology, Mongolian Academy of Sciences, Ulaanbaatar 15160, Mongolia; Key Laboratory of Earth Probe and Geodynamics, Chinese Academy of Geological Sciences, Beijing 100037, China

**Keywords:** magmatic migration, ocean closure, orocline, Mongol-Okhotsk Ocean

## Abstract

Tracing the closure of oceans with irregular margins and the formation of an orocline are crucial for understanding plate reconstruction and continental assembly. The eastern Central Asian Orogenic Belt, where the Mongol-Okhotsk orocline is situated, is one of the world's largest magmatic provinces. Using a large data set of U-Pb zircon ages, we updated the timing of many published igneous rocks, which allowed us to recognize tightly ‘folded’ linear Carboniferous-Jurassic magmatic belts that wrap around the Mongol-Okhotsk suture and their migrations both sutureward and suture-parallel. The new successive magmatic belts reveal a rollback, scissor-like (or zipper-like) closure of the Mongol-Okhotsk Ocean that was fundamentally controlled by coeval subduction rollback and rotation of the Siberian and Mongolian-Erguna blocks. This study also demonstrates the complex mechanisms and processes of the closure of an ocean with irregular margins and the formation of a consequent orocline.

## Teaser

A large data-set of ages of magmatic rocks reveals tightly 'curved' linear magmatic belts and their migrations towards the central suture, which indicates a scissor-like closure of the Mongol-Okhotsk Ocean. These well-documented relations help us better understand the closure process of an ocean with irregular margins and the formation of curved mountain belts.

## INTRODUCTION

There are many modern and ancient curved arc margins and/or oroclines in oceans and continents. Understanding how such curved, geological structures (mountain belts and orogens) form and evolve is a first-order problem in the Earth sciences [1], and consequently they have been well studied for their structure, composition and formation [2–10]. However, the style (and fate) of their development, the methods of final closure of the oceans and, particularly, the closure–(suture)–orocline relationships are still not well understood. Diagnosing an ancient suture zone that was derived from a well-defined paleo-ocean, is a key problem. In consequence, tracing and restoring the closure of paleo-oceans and the formation of ancient oroclines are useful constraints that can improve our understanding of plate reconstruction and continental assembly [11].

The Central Asian Orogenic Belt (CAOB; [12,13] including the Altaids [14]) is the world's largest Phanerozoic accretionary orogen that contains several suture zones and oroclines such as the Mongol-Okhotsk orocline in the eastern CAOB [14–21], thus the CAOB is a promising orogen for investigating ancient suture–orocline relationships. In spite of innumerable publications on most aspects of Earth sciences in the CAOB, there have been few studies on the relationships between the formation of sutures (closure of oceans) and the creation of oroclines. Nevertheless, the abundant, well-dated magmatic rocks in the eastern CAOB, particularly in eastern Mongolia, Trans-Baikalia in Russia and the Great Xing’an region in NE China, constitute one of the world's largest Phanerozoic felsic magmatic provinces (>5 500 000 km^2^ with >6000 igneous bodies, which occupy 60% of the outcrop area [12,22]). This magmatic province provides an invaluable databank for the study of magmatism–suture–orocline relationships.

Building on the geochemical data [23], we have produced a geochronological database of more than 2660 U-Pb zircon ages (446 are our data including 267 new unpublished ages), which we present in a series of digital maps of the magmatic rocks in the eastern CAOB. This enables us to define a series of tightly ‘folded’ magmatic belts that delineate the Mongol-Okhotsk orocline, to demonstrate the successive magmatic migrations, and to reconstruct the closure mechanism of the Mongol-Okhotsk Ocean as well as the formation of the orocline [15]. This paper presents a case study that demonstrates relationships between magmatism-sutures (closure of oceans) and oroclines.

## TECTONIC SETTING OF THE MONGOL-OKHOTSK SUTURE

**Figure 1.**
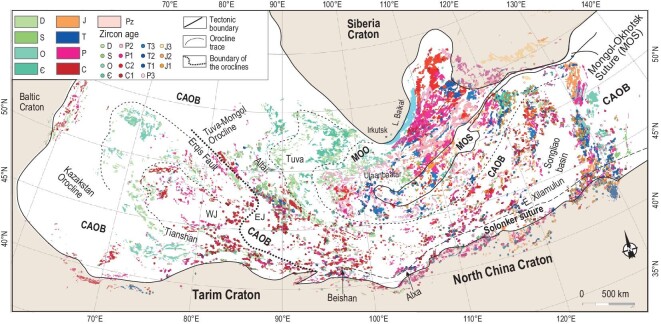
Tectono-magmatic map of the CAOB showing Cambrian-Jurassic granitic and related rocks. The major tectonic units are modified after Refs [14,18]. MOO = Mongol-Okhotsk orogen; MOS = Mongol-Okhotsk suture; Є = Cambrian; O = Ordovician; S = Silurian; D = Denonian; C = Carboniferous; P = Permian; T = Triassic; J = Jurassic; Pz = Paleozoic. The numbers 1, 2 and 3 that follow P, C, T, J represent early, middle and late phases, respectively.

The CAOB [13], which contains the younger and smaller Altaids [14], is a system of collages of many accretionary complexes, magmatic arcs, arc-related basins, ophiolites, seamounts and continental fragments. The CAOB is bounded by the Siberia Craton to the north, the Tarim-North China Craton to the south and the Baltica (East European) Craton to the northwest (Fig. 1) [14,18,24]. The CAOB is a typical accretionary orogen that records the long-lived accretion of the Paleo-Asian Ocean (PAO) and subsequent collisions of terranes and microcontinents during 1000–250 Ma [12,13,18]. The younger Altaids developed from ∼600 Ma to 250 Ma [14,15,18]. Two large oroclines are the Kazakhstan and Mongol-Okhotsk (or Mongolian); the Kazakhstan orocline is in the western CAOB and its formation was related to the Paleozoic closure of the Paleo-Asian Ocean. The Mongol-Okhotsk orocline is in the eastern CAOB (Fig. 1); formation of the western part of the orocline led to the Paleozoic closure of the Paleo-Asian Ocean, but the eastern part (core) was related to the Mesozoic closure of the Mongol-Okhotsk Ocean [18,20].

The Mongol-Okhotsk suture, located in the core of this eponymous orocline, extends for ∼3000 km from the Khangai Mountains in central Mongolia eastwards to Uda Bay in East Okhotsk [23,25,26]. To the north of the orocline is the Siberian Craton, to the south is the southern Mongolian Massif and to the east is the Pacific plate. The formation of the Mongol-Okhotsk suture played an important role in the final construction of the East Asian continent [16,18,27,28] from the late Paleozoic to late Mesozoic. The initiation of the active continental margin of the Mongol-Okhotsk Ocean may have been as early as Silurian-Devonian [29–31], but the mature, slightly curved, active margin began to form in the Carboniferous to early Permian [32–34]. The closure of the ocean started at the western end in the late Paleozoic, then closed progressively eastwards, terminating in the late Mesozoic in a scissor-like motion not far from the present-day Okhotsk Sea [25]*.* As a whole, the Mongol-Okhotsk Ocean closed by double-sided subduction [26,35]. The time of terminal closure is controversial: Early-Middle Jurassic [25,36], Late Jurassic (based on paleomagnetic data; [37]) or earliest or Middle-Late Cretaceous [28]. The progressive development of the Mongol-Okhotsk orocline with respect to the ocean closure is still not understood in detail. This study helps to resolve these issues by tracing the magmatic evolution of the orogen in relation to formation of the Mongol-Okhotsk orocline.

## MAGMATIC BELTS AROUND THE MONGOL-OKHOTSK SUTURE AND THEIR MIGRATION

**Figure 2.**
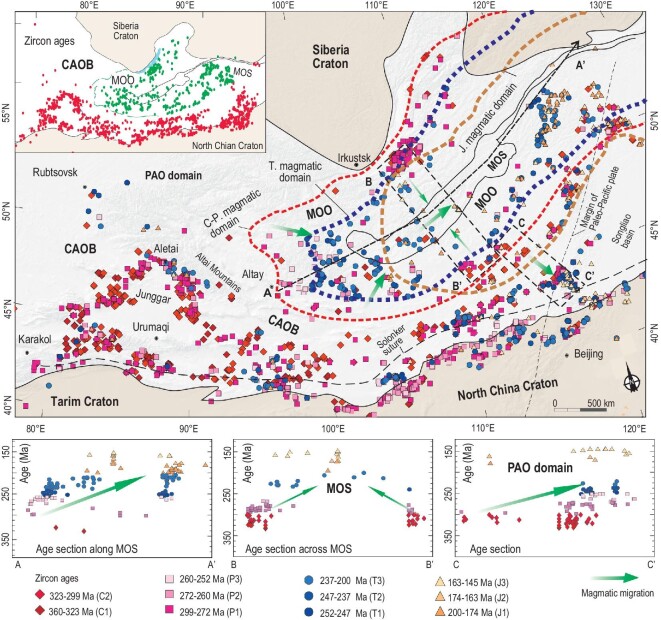
U-Pb zircon age distributions of Carboniferous-Jurassic granitic and related igneous rocks in the Mongol-Okhotsk orogen and adjacent areas. The red, blue and brown dotted lines mark the distribution limits of the Carboniferous-Permian, Triassic, and Jurassic magmatic rocks, respectively. The upper left inset diagram shows these U-Pb zircon age distributions within the Mongol-Okhotsk oceanic domain (green) and Paleo-Asian oceanic domain (red). The three inset diagrams at the bottom show the age variations along and around the sutures, which indicate magmatic migration. For abbreviations see Fig. 1. The ages, including our new data, are listed in Table S1.

**Figure 3.**
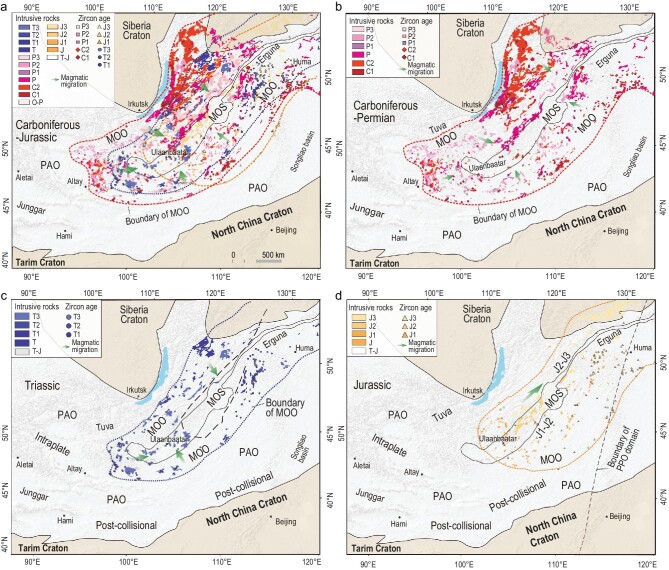
Maps of the (a) Carboniferous-Jurassic, (b) Carboniferous-Permian, (c) Triassic and (d) Jurassic granitic and related intrusive rocks in the Mongol-Okhotsk orogen showing their distribution limits (dotted lines) and migration directions.

Many different magmatic rocks occur in and around the Mongol-Okhotsk suture (Fig. 1). We have determined the zircon U-Pb ages of 350–145 Ma calc-alkaline granitic rocks mostly in the western and southern parts of the Mongol-Okhotsk orogen (Fig. 2). These newly dated rocks, combined with previously determined, coeval magmatic rocks in the northern, western and southern parts [26,29,33,34,38–47] enable us to follow the sequential and continuous development of three magmatic belts that wrap around the Mongol-Okhotsk suture (Figs 2 and 3).

### Carboniferous-Permian magmatic belt

A belt of Carboniferous-Permian magmatic rocks occurs in the northern (Trans-Baikalia), western and southeastern sides of the Mongol-Okhotsk orogen (Figs 2 and 3; [26,29,33,34,38–47]). The rocks of the belt in the northern (Trans-Baikalia) and western sides comprise granodiorites, biotite granites, leucogranites, monzonites, quartz syenites, syenites and mafic rocks [33,44]. Their rock associations and geochemical affinities are typical of an active continental margin. For instance, the mafic rocks are depleted in Nb, Ta and Ti and enriched in Sr, Ba and Pb [33]. Alkaline-peralkaline rocks (280–275 Ma) have A2-type granitic geochemical affinities and were probably formed in an (extensional) active continental margin [33,34].

The constituent rocks in the southeastern sides of the Mongol-Okhotsk orogen are mostly granodiorites, monzogranites and minor syenogranites [44,45]. They belong to the calc-alkaline, high-K calc-alkaline series and are mostly metaluminous to weakly peraluminous (A/CNK = 0.90–1.10) I-type granites, characterized by negative correlations between P_2_O_5_ and SiO_2_ [45]. Some monzogranite and syenogranite exhibit strong negative Nb-Ta Ti and Sr anomalies, and light rare Earth element (LREE)-enriched chondrite-normalized rare Earth element (REE) patterns with moderate Eu anomalies (Eu^*^ = 0.16–1.03) [44]. Locally, peraluminous (mostly A/CNK = 1.10–1.28) S-type granites in northeastern Mongolia are considered to have been emplaced in an active margin related to the subduction of the Mongol-Okhotsk oceanic plate [44]. Recently, Li et al. [48] reported 310–280 Ma igneous rocks in the Jiamusi Massif, northeastern China, and speculated that these rocks formed in a subduction setting of the Mongol-Okhotsk oceanic plate, rather than the Paleo-Pacific oceanic or the Paleo-Asian oceanic plates.

Furthermore, we provide two other lines of evidence that the Carboniferous-Permian magmatic belt formed by subduction of the Mongol-Okhotsk Ocean rather than the Paleo-Asian Ocean. First, the belt is curved and distinctly different from the linear EW-trending magmatic belts in the Paleo-Asian Ocean domain (Fig. S1). The curved belt wraps the Mongol-Okhotsk suture, whereas the magmatic belt in the southern CAOB of the Paleo-Asian Ocean domain extends in a straight line westward to the Altai-Junggar, thus constituting a >900-km-long, giant magmatic belt that contains abundant alkaline rocks and A-type granites (Fig. S1; [49]). Second, we can identify two belts with opposite magmatic migration directions though they are parallel and trend WSW–ENE in southeast Mongolia. The magmatic belt belonging to the Mongol-Okhotsk Ocean regime migrated northwards towards the Mongol-Okhotsk suture. In contrast, the magmatic belts belonging to the Paleo-Asian Ocean regime migrated southwards towards the Solonker-Xilamulun suture along the border between China and Mongolia (Fig. S1). This movement was controlled by southward accretion and final closure of the Paleo-Asian Ocean along the suture [20].

The above relations indicate that the Carboniferous-Permian magmatic belt around the Mongol-Okhotsk suture formed in a single active continental margin. This means that a fully fledged active margin of the Mongol-Okhotsk Ocean was probably initiated at least prior to ca. 350 Ma [33].

### Triassic magmatic belt

**Figure 4.**
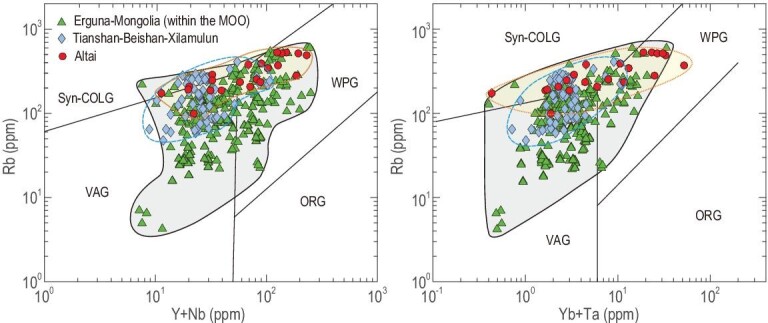
Comparison of the geochemical features and tectonic settings of Triassic granitoids from the Mongo-Okhotsk orogen (MOO) and other areas (Tianshan-Beishan-Xilamulun and Altai) of the CAOB: Erguna-Mongolian (MOO) continental arc belt, Tianshan-Beishan-Xilamulun post-collisional magmatic belt, and Altai intraplate magmatic belt. See the locations of these areas in Fig. S1. For data sources see Table S3.

The magmatic belt of Triassic rocks, surrounded by the peripheral Carboniferous-Permian magmatic belt, is located nearer to the Mongol-Okhotsk suture (Figs 2 and 3b). Our newly dated Triassic granitic rocks (250–200 Ma) occur on the southern side and especially at the western end of the suture (Fig. 2). Combined with previously determined Triassic granitic rocks, these rocks constitute a continuous magmatic belt that wraps around the whole Mongol-Okhotsk suture and were formed at an active margin (Fig. 3b). These rocks are largely granodiorites, monzogranites and syenogranites, most of which have metaluminous to weakly peraluminous I-type signatures, characterized by negative correlations between P_2_O_5_ and SiO_2_ [41]. Compared with other Triassic granitoids in the Paleo-Asian Ocean domain, these Triassic rocks around the Mongol-Okhotsk suture have more arc signatures [41,43,44]. In the Rb vs. Y+Nb diagram (Fig. 4), most of the Triassic granites with depletions of high field strength elements such as Nb and Ta fall in the volcanic arc granitic field. An arc setting is also supported by the presence of Late Triassic (230–200 Ma) to Early Jurassic (200–178 Ma) arc-type, porphyry copper-molybdenum deposits [38,50,51]. Some Triassic igneous rocks far away from the Mongol-Okhotsk suture formed in a back-arc environment by southward subduction of the Mongol-Okhotsk oceanic plate, such as the appinite-granites in Duobaoshan, south of the Erguna Massif [52].

These arc characteristics of Triassic magmatic rocks are different from those of coeval magmatic rocks that formed in a post-orogenic setting in Tianshan-Beishan-Xilamulun and in an intraplate setting in Altai within the Paleo-Asian Ocean domain (Fig. 4) [53,54]. Importantly, the Triassic magmatic belt shows sutureward younging from the Early to Late Triassic; this is obvious in the northern and western segments of the Mongol-Okhotsk suture (Figs 2 and 3c) [33], thus revealing important rollback subduction.

**Figure 5.**
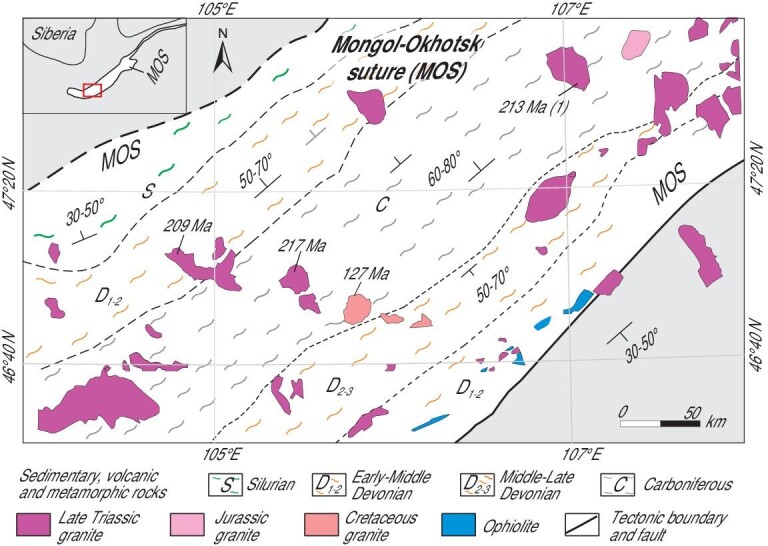
Local Triassic stitching plutons in the western segment of the Mongol-Okhotsk accretionary complexes (modified from a 1 : 1000 000-scale geological map of Mongolia based on our field work). These plutons (220–210 Ma) consist of slightly peraluminous, high-K calc-alkaline, biotite and hornblende-biotite granites of A2-type. They were intruded into accretionary complexes that were already folded and had a sub-vertical foliation, suggesting the suture had formed and the Mongol-Okhotsk Ocean had closed here at least by ca. 210 Ma. Zircon ages from Ref. [55] and our new data.

Many alkaline-peralkaline plutons (220–210 Ma) occur mainly in the hinge zone and adjacent areas. These plutons were probably emplaced in an active continental margin [33] and/or a post-accretionary extensional margin. Significantly, some plutons (220–210 Ma) were intruded into the western segment of ophiolite zone of the Mongol-Okhotsk accretionary complexes and can be considered as local stitching plutons (Fig. 5). This indicates that the Mongol-Okhotsk Ocean was mostly consumed here before emplacement of the 217 Ma plutons. However, at this time an ocean still existed farther east, and consequently the ocean closed from west to east in a scissor/zipper-like fashion.

### Jurassic magmatic belt

A belt of Jurassic magmatic rocks occurs mainly in the eastern Mongol-Okhotsk orogen where it extends along its whole length (Figs 2 and 3c). Early Jurassic rocks in the belt consist mainly of granodiorites-granites and calc-alkaline volcanic rocks (basalt-basaltic andesite-andesite), which exhibit continental arc signatures, as in the Erguna Massif and Xing’an region [45,56]. These rocks are also associated with coeval arc-type porphyry Cu-Mo ore deposits [38,50,51]. Some Middle Jurassic (170–160 Ma) granitoids in Erguna are peraluminous and have S-type and high Sr/Y ratios, probably suggesting an initial collisional setting related to the closure of the Mongol-Okhotsk Ocean [56]. A late collision may have given rise to a magmatic gap between 155 and 145 Ma [57], which would be in accord with the ambient regional compressional background of NE Asia, i.e. the Yanshan movement that generated giant folds and thrust belts in northern China and Mongolia [58]. Recent studies of detrital zircons from stratigraphic sections of the central East Basin of the Mongol-Okhotsk suture zone suggest the initiation of a Middle Jurassic collisional foreland basin, the development of which is assigned an age of ∼165–155 Ma [59], and some of the latest Jurassic alkaline-peralkaline rocks are associated with Early Cretaceous magmatic rocks (see below).

The Jurassic magmatic belt in the eastern Mongol-Okhotsk orogen is different from the Jurassic magmatic belt along the margin of the Paleo-Pacific Ocean. The former is concentrated only in the eastern Mongol-Okhotsk orogen and extends to the northeast, whereas the latter extends in a NE direction from the Erguna-Great Xing’an Range to the Taihang Mountains and to SE China [57].

Early Cretaceous (145–120 Ma) felsic-intermediate rocks and mafic lavas are widespread across the whole of the Mongol-Okhotsk orogen, as well as in much of NE Asia [22,33,56,60]. They have bimodal affinities and most of the felsic-intermediate rocks are associated with alkaline and/or A-type granites, suggesting an extensional setting [57]. All these Early Cretaceous magmatic rocks, together with associated coeval extensional structures (such as metamorphic core complexes [34,61] and extensional or rift basins [62]) indicate a large-scale post-collisional extension of the Mongol-Okhotsk orogen [61] followed by lithospheric thinning/delamination [35]. This is consistent with the terminal closure of the Mongol-Okhotsk Ocean at ca. 160–150 Ma.

**Figure 6.**
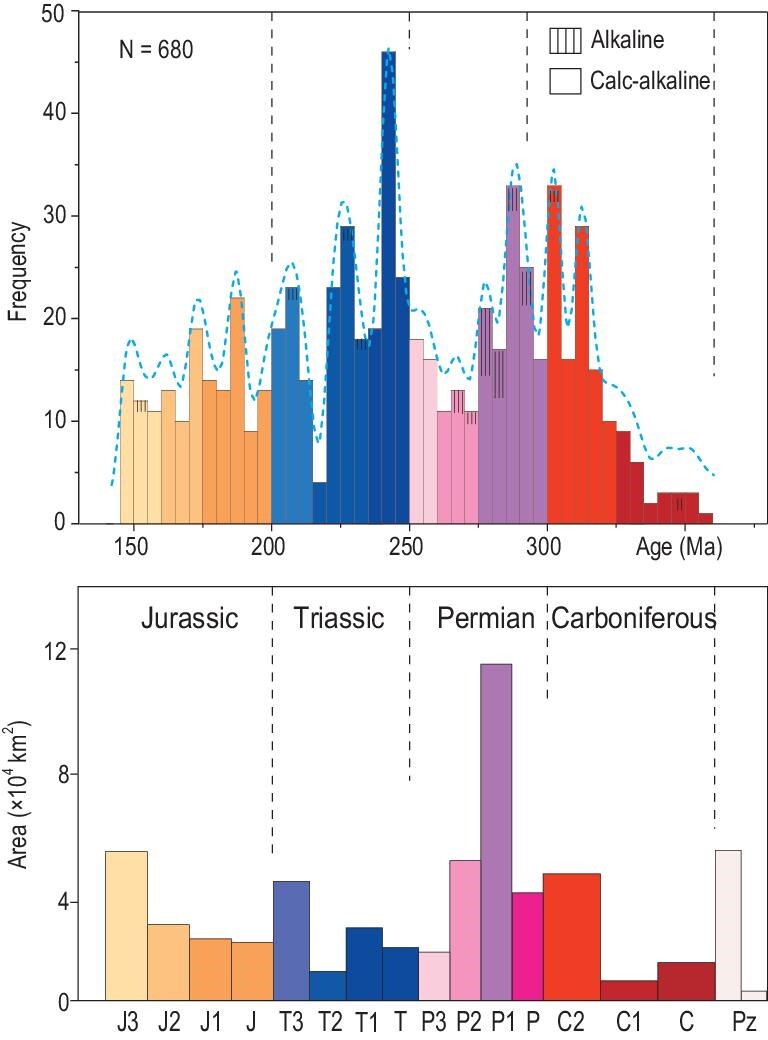
Age frequency and areal distributions of granitic and related igneous rocks in the Mongol-Okhotsk orogen. See explanations in the text and data in Table S1.

Figure 6 shows a summary of the zircon ages and areal distribution of these magmatic rocks. The zircon ages record semi-continuous magmatism from 350 to 150 Ma with only two major weak peaks at 310–290 Ma and 240–230 Ma, and with gaps at 270–260 Ma and 230 Ma. Alkaline magmatism mainly occurred near the end of each peak magmatism. These relations suggest semi-continuous phases of oceanic subduction. The areal distribution demonstrates that the Carboniferous-Permian igneous rocks are far more voluminous than the Triassic-Jurassic igneous rocks, particularly in the northern and western sides of the Mongol-Okhotsk suture. From this, we speculate that the Mongol-Okhotsk Ocean was large (wide) in the Carboniferous-Permian and small in the Triassic-Jurassic. This is consistent with recent paleomagnetic data [63–65].

## RESTORING CLOSURE OF THE MONGOL-OKHOTSK OCEAN: MAGMATIC PERSPECTIVE

The Mongol-Okhotsk orogen is a collage of different tectonic units (accretionary complexes, terrenes and massifs), and the margins of the ocean were variable in shape and composition. Consequently, the development of the magmatic rocks during the formation of the orogen was likely complex and irregular. Nevertheless, from a statistical viewpoint the Carboniferous-Jurassic magmatic belts have regular migration trends.

The Carboniferous-Triassic magmatic migration towards the Mongol-Okhotsk suture reflects oceanward migration. These relationships, combined with the alkaline magmatism, reveal retreating or rollback subduction or extensional accretion of the Mongol-Okhotsk oceanic plate. An oceanward migration of magmatism caused by successive rollbacks is a known tectonic process as in the formation of the Paleozoic Lachlan orogen in eastern Australia [66–68], and the Cretaceous magmatic belt along the western Pacific plate margin [69]. Moreover, several other oroclines have been recognized by alignment of their magmatic rocks, such as the early Permian granitoids in the New England orogen of eastern Australia [6]. Nevertheless, our study is the first not only to identify a tight orocline by two isoclinal, parallel-contact magmatic belts, but also to restore rollback subduction from oroclinal magmatic belts around a fossil suture zone.

**Figure 7.**
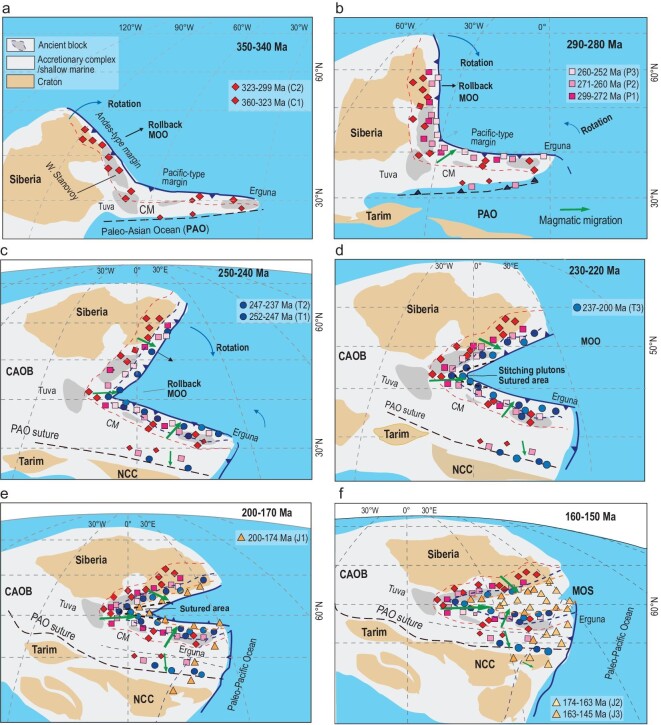
A schematic model showing rollback and scissor/zipper-like closure of the Mongol-Okhotsk Ocean. (a–c) Oceanward migration of Carboniferous-Triassic subduction-related magmatism and (d–f) consequent eastward Triassic-Jurassic migration along the suture. The black dashed lines show sutured or closure areas of the ocean. NCC = North China Craton. Paleogeographic reconstructions are modified from Ref. [25].

Many Late Triassic (ca. 220–200 Ma) plutons (some are alkaline) that were emplaced discordantly into the western segment of the Mongol-Okhotsk suture were post-accretionary stitching plutons (Fig. 3c). This indicates that the Mongol-Okhotsk Ocean closed and the western suture formed by Triassic (pre∼220 Ma) time. Furthermore, the eastward-younging Triassic-Jurassic magmatism along the suture zone (Fig. 2) indicates a continuous eastward suturing in a scissor- or zipper-like style. The magmatic evolution from Middle Jurassic calc-alkaline granitic rocks to Early Cretaceous alkaline rocks, which was associated with a tectonic transition from contraction to extension at ca. 150 Ma [61], strongly suggests that ca. 160–150 Ma was the most likely time for the terminal closure of the ocean in the Far East. This conclusion is in accordance with ophiolite isotopic ages [26], paleomagnetic data [37], seismic tomography and numerical modeling [70]. Accordingly, after integrating all the above information, we propose the following sequential relationships in a new tectonic model (Fig. 7).

**Figure 8.**
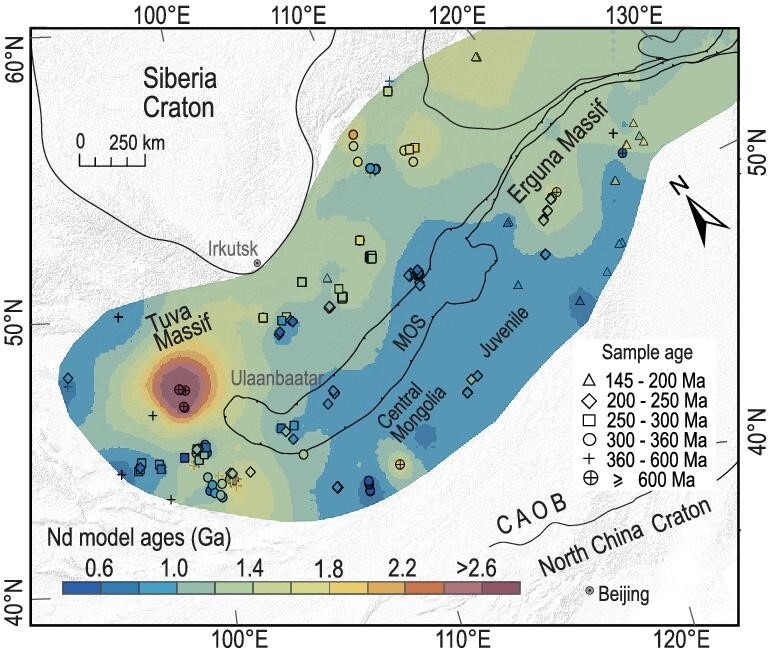
Two-stage distribution of Nd model ages of the Carboniferous-Jurassic granitic rocks in the Mongol-Okhotsk orogen. See explanations in the text and data in Table S2.

- A continuous linear or slightly curved, mature active margin of the Mongol-Okhotsk Ocean started at least by Carboniferous time. The northern segment was an Andean-type continental margin, but the southern segment was a juvenile, Japanese-type, accretionary complex that contained only a few ancient massifs such as the Erguna Massif. These relations are indicated by granitoid isotopic mapping (Fig. 8) that shows more positive ϵ_Nd_ (0 − +6) values with younger model ages (1.5–0.6 Ga), which are more widespread in the southern than in the northern margin. A comparable analogue is the modern plate regime in Alaska, which has a single arc with a continental signature on one side and a subduction/accretion belt with an oceanic signature on the other [71]. The Mongol-Okhotsk orocline contains all these relationships. Significantly, the orocline has preserved the juvenile crust well.
- The active margin began to be deformed and curved after at least the Permian. The ocean began its scissor/zipper-like closure by the Triassic (pre∼230 Ma), which was completed in the east by the Late Jurassic (ca. 160–150 Ma). The hinge of the orocline is at the junction between the northern and southern segments at the western end (present coordinates). The junction resembles the inflection point of the modern eastern Philippine plate subduction zone. Some global reconstruction models have suggested that the Mongol-Okhotsk Ocean was large and extended westward (present coordinates) to connect with the Paleo-Tethyan Ocean in Triassic time [70]. Our study, however, does not include such models.
- The curved margin of the Mongol-Okhotsk Ocean underwent rollback subduction. The subduction direction was toward the outer of the curves in a direction like that of other oroclines in Kazakhstan [15,17], the Mediterranean [7] and the Andes [1,9], but different from the curved subduction system in SE Asia, where the subduction direction is towards the interior [72]. Interestingly, the outer (western) part of the Mongol-Okhotsk orocline was formed by inner suture subduction of the Paleo-Asian Ocean (PAO) [18], and consequently the orocline was formed by two oceanic plate dynamics. Additionally, regarding the bending of the orocline, some extensional basins developed outside the hinge zone, accommodating the ‘folding’ [18,30].
- Oroclines with contorted/folded magmatic belts are known in other orogens [6,73]. However, in the Mongol-Okhotsk orogen we can identify not only the two tightly ‘folded’ parallel magmatic belts that make up the tight orocline, but also both sutureward (oceanward) and suture-parallel magmatic migrations.

There are two fundamentally different types of oroclines, progressive and secondary, that are generally interpreted as stress-perpendicular and stress-parallel [1]. Orogen-normal principal compression (including advanced subduction), asymmetric retreat (or rollback) on both subducting margins, orogen-parallel shortening and transpressional slip are the most likely principal mechanisms responsible for the formation of an orocline [1,4,5,74].

It has been speculated that the Mongol-Okhotsk orocline was formed by Carboniferous E–W shortening leading to N–S tectonics before the Late Permian to Late Triassic orthogonal N–S shortening [24,30]. However, we have found no evidence for such orogen-normal principal compression (advanced subduction), orogen-parallel shortening or transpressional slip, even when retreat or rollback subduction has clearly taken place. The rollback was apparently stronger in the northern limb and the hinge zone of the orocline, as demonstrated by (i) wider Carboniferous-Jurassic magmatic belts; (ii) much stronger sutureward migration of Carboniferous-Triassic magmatic belts in the northern and western segments of the Mongol-Okhotsk suture (Figs 2 and 3); and (iii) far more igneous rocks, particularly alkaline types, suggesting strong extensional accretion. This symmetric rollback, i.e. stronger in the northern and western segments of the suture, could have driven the initial curvature of the Mongol-Okhotsk oceanic margin. Significantly, clockwise rotation of the Siberian craton and anticlockwise rotation of the Mongolian-Erguna (Amuria) block took place at this time as revealed by integration of geological information [16,18], particularly paleomagnetic data [30,37]. This rollback could not have driven the rotation of the too large Arctic cratons that might have been conducive to the rotation, both of which promoted the closure of the Mongol-Okhotsk Ocean. This rotation was likely compressive and the bending of the orocline was not large. Thus, there was no large-scale extension capable of opening an ocean like the Asgard Sea that was driven by the late Mesoproterozoic clockwise rotation of Baltica with respect to Laurentia [10].

Accordingly, we propose that the two independent, but contemporaneous, rollback and rotation factors were the fundamental mechanisms responsible for the formation of the Mongol-Okhotsk orocline and the ocean closure. Our study sheds light on the vital role played by the rotation of convergent blocks and rollback subduction in the formation of a tight orocline.

As discussed above, the Mongol-Okhotsk orocline was formed by two oceanic plate dynamics: the eastern core (of the Mongol-Okhotsk orogen) caused by the Mongol-Okhotsk oceanic plate and the western outer part (the western CAOB) by the PAO plate. The giant curved arc systems of SE Asia could be a modern analogue [72]: to the east is the Pacific Ocean and to the west the Indian Ocean. Moreover, there is a larger Kazakhstan orocline in the western CAOB. Thus, the CAOB contains several oroclines and orogens that were derived from different plate dynamics. The fact that the eastern CAOB hosts one of the world's largest felsic igneous provinces with more than 6000 igneous bodies (ca. 5000 intrusions) allows us to speculate that these may represent the incipient stage in the formation of a Large Igneous Province (LIP), which is characterized by an abundance of igneous intrusions.

## CONCLUSIONS

Using a particularly large data set, we identified a series of tightly ‘folded’ linear (oroclinal) Carboniferous-Jurassic magmatic belts that wrap around the Mongol-Okhotsk suture, and we recognized both sutureward and suture-parallel magmatic migrations. These new findings reveal a rollback scissor/zipper-like ocean closure. Rollback of subducted plates and rotation of convergent blocks are the most likely fundamental mechanisms responsible for this kind of ocean closure and related orocline formation. This study demonstrates how coeval oceanward and suture-parallel magmatic migrations can reveal a rollback and scissor/zipper-like closure of an ocean, which make for formation and preservation of so many magmatic rocks. These processes help understand the complex closure of an ocean with curved and irregular margins and the formation of an orocline. Determining the magmatism of an ancient suture zone is a useful approach to unraveling the relationships of arc folding, oroclinal development and ocean closure/suture.

## Supplementary Material

nwab210_Supplemental_File
