# Supplementary material for: Rollback, scissor-like closure of the Mongol-Okhotsk Ocean and formation of an orocline: magmatic migration based on a large archive of age data
Source: Natl Sci Rev. 2021 Nov 29;9(5):nwab210. doi: 10.1093/nsr/nwab210 (PMC9084359; doi:10.1093/nsr/nwab210)
Supplement: nwab210_Supplemental_File [file nwab210_supplemental_file.pdf]

Supplementary information

Characteristics of granitic rocks of the Central Asian Orogenic Belt and comparisons with the Mongol-Okhotsk Orogen.

In order to understand better the background of the magmatic rocks within the Mongol-Okhotsk Orogen, we present the following figures. **Figures S1** shows the major tectonic units and all intrusive rocks in the CAOB, which compare well with the distribution of such magmatic rocks in the Mongol-Okhotsk Orogen and adjacent areas (the western and southern CAOB). See detailed explanations in the text.

Nb isotopic data of granitic rocks of the Mongol-Okhotsk Orogen

The Carboniferous-Jurassic granitic rocks in the Mongol-Okhotsk Orogen show marked variations in their Nd isotopes (Table S2). Granitic rocks to the northwest of the Mongol-Okhotsk suture have high negative εNd values (0- -17) and old model ages (2.8-1.2 Ga), whereas to the southeast of the suture there are mostly positive εNd values and young model ages (1.5 – 0.6 Ga). These relations suggest that the northern segment of the orogen is composed of ancient massifs (such as the Stannovoy-Tuva Massif) with Andean-type granitoid plutons and batholiths, whereas the southern segment comprises a juvenile accretionary complex with only a few ancient massifs such as the Erguna. For data see Table S2.

Supplementary figure captions

Figure S1. Maps of (a) Carboniferous-Jurassic, (b) Carboniferous-Permian, (c) Triassic, and (d) Jurassic granitic rocks in the main part of the CAOB. In Fig. S1-c, green, purple, and brown dotted lines represent the southern Mongol-Okhotsk arc granitic belt, Beishan-Xar Moron post-collisional granitic belt, and the Altai intraplate granitic belt, respectively (see [52]). Acronyms as in Fig. 2.

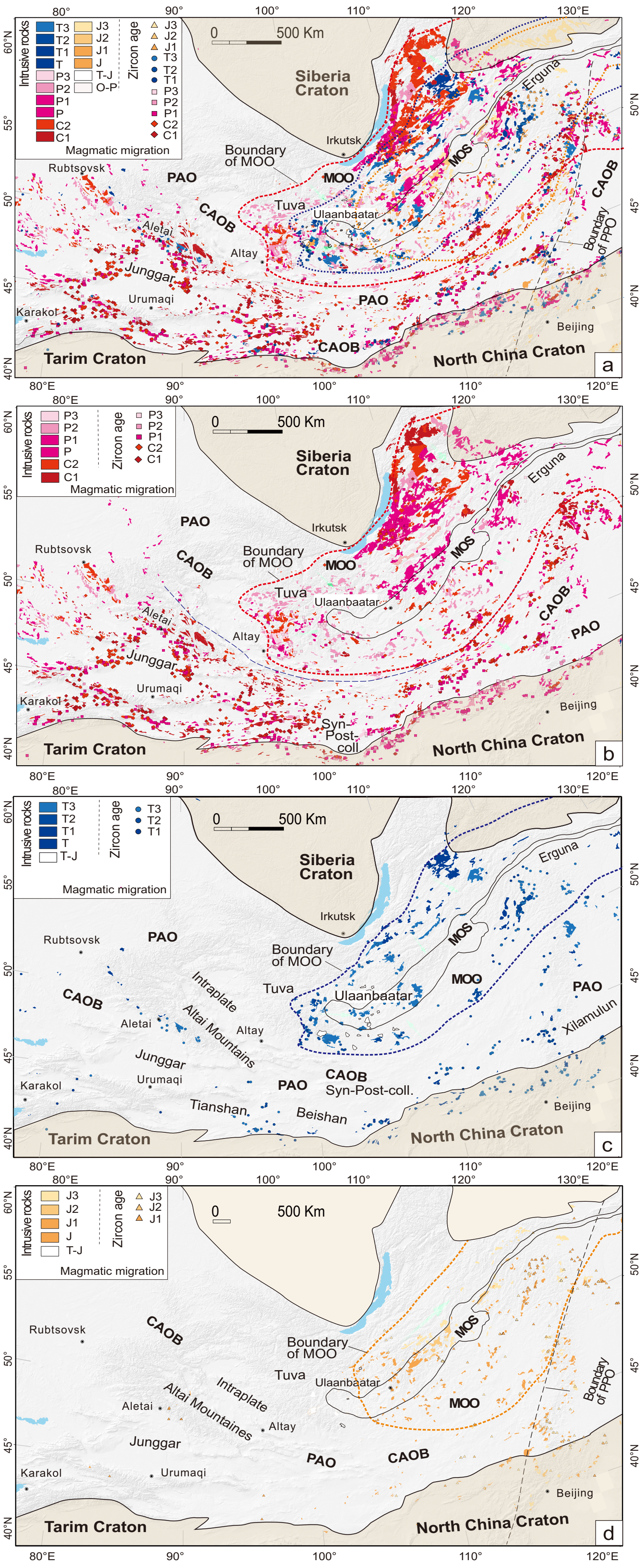

a
b
c
d
Intrusive rocks
Zircon age
Magmatic migration
500 Km
Siberia Craton
Tarim Craton
North China Craton
PAO
CAOB
MOO
MOS
Boundary of MOO
Boundary of PPO
Erguna
Irkutsk
Tuva
Ulaanbaatar
Rubtsovsk
Aletai
Altay
Junggar
Urumaqi
Karakol
Beijing
Syn-Post-coll
Syn-Post-coll.
Intraplate
Altai Mountains
Altai Mountaines
Xilamulun
Tianshan
Beishan

Figure S1

Supplementary table captions

Table S1 U-Pb zircon ages of Carboniferous-Jurassic magmatic (mostly granitic) rocks of the Mongol-Okhotsk Orogen and other regions of the Central Asian Orogenic Belt.

Table S1 U-Pb zircon ages of Carboniferous-Jurassic magmatic rocks of the Mongol-Okhotsk Orogen and other regions of the Central Asian Orogenic Belt

No.	Tectonic Group	Location	Sample	Pluton	Lithology	Longitude	Latitude	Age (Ma)	Error	Test_Method	Mineral	Reference	Year	Journal
1	Within the MOO	C./S. Mongolia	J1	Avdar	Rare-metal granite	105.45	47.62	224	10.0	SHRIMP,U-Pb	Zircon	Donskaya T.V. et al.	2008	Journal of the Geological Society
2	Within the MOO	C./S. Mongolia	WOL-12	Tukhum	Biotite granite	105.17	46.16	191	1.8	LA-ICP-MS,U-Pb	Zircon	Dostal J. et al.	2020	Frontiers in Earth Science
3	Within the MOO	C./S. Mongolia	WOL-15	Tukhum	Biotite granite	105.28	46.01	183	1.5	LA-ICP-MS,U-Pb	Zircon	Dostal J. et al.	2020	Frontiers in Earth Science
4	Within the MOO	C./S. Mongolia	WOL-14	Tukhum	Biotite granite	105.37	46.21	183	1.6	LA-ICP-MS,U-Pb	Zircon	Dostal J. et al.	2020	Frontiers in Earth Science
5	Within the MOO	C./S. Mongolia		Dashibalbar	A-type Granite	107.11	46.70	186	1.0	LA-ICP-MS,U-Pb	Zircon	Dostal Jarosalv et al.	2014	American Journal of Science
6	Within the MOO	C./S. Mongolia	T-17-2	Toumuertingaobao	Monzogranite	113.33	46.79	241	3.0	SHRIMP,U-Pb	Zircon	Jiang S H et al.	2010	Acta Geoscientica Sinica(ICWEA)
7	Within the MOO	C./S. Mongolia	N2028	Bogd Uul	Bi. leucogranite	107.05	47.86	208	9.0	LA-SF-ICP-MS,U-P	Zircon	Khishigsuren Sodnom	2003	Mongolia Geoscientist
8	Within the MOO	C./S. Mongolia	N2044	Bogd Uul	Granite-porphyry	107.11	47.69	224	30.0	LA-SF-ICP-MS,U-P	Zircon	Khishigsuren Sodnom	2009	Mongolia Geoscientist
9	Within the MOO	C./S. Mongolia	N49	Bogd Uul	Granite	106.98	47.77	191	5.0	LA-SF-ICP-MS,U-P	Zircon	Khishigsuren Sodnom	2009	Mongolia Geoscientist
10	Within the MOO	C./S. Mongolia	N2057	Bogd Uul	Porphyritic biotite granite	106.98	47.86	206	4.0	LA-SF-ICP-MS,U-P	Zircon	Khishigsuren Sodnom	2009	Mongolia Geoscientist
11	Within the MOO	C./S. Mongolia	NM60	NE of Uulan Bator	Rhyolite	106.49	47.95	334	1.0	Pb-Pb	Zircon	Kröner et al. unpublished		
12	Within the MOO	C./S. Mongolia	4001—1	Bayandulan	Monzogranite	111.08	45.97	277	0.7	LA-ICP-MS,U-Pb	Zircon	Li M et al.	2015	Geological Science and Technology Information(ICWEA)
13	Within the MOO	C./S. Mongolia	A-5	Aryn nuur	Biotite granite	113.97	47.22	229	2.2	SHRIMP,U-Pb	Zircon	Liu Yifei et al.	2010	Acta Geoscientica Sinica(ICWEA)
14	Within the MOO	C./S. Mongolia	56	Zhanchivlan massif	Leucogranite	106.50	47.73	227	8.0	SHRIMP,U-Pb	Zircon	Mazukabzov et al.	2006	Doklady Earth Sciences
15	Within the MOO	C./S. Mongolia	2011MO-74	Ereendavaa terrane	Mylonitic granite	111.76	48.79	172	2.0	SHRIMP,U-Pb	Zircon	Miao Laicheng et al.	2017	Journal of Asian Earth Sciences
16	Within the MOO	C./S. Mongolia	2011MO-75	Ereendavaa terrane	Granitic pegmatite	111.72	48.80	163	3.0	SHRIMP,U-Pb	Zircon	Miao Laicheng et al.	2017	Journal of Asian Earth Sciences
17	Within the MOO	C./S. Mongolia	2011MO-77	Ereendavaa terrane	Granite	111.64	48.84	174	3.0	SHRIMP,U-Pb	Zircon	Miao Laicheng et al.	2017	Journal of Asian Earth Sciences
18	Within the MOO	C./S. Mongolia	2011MO-83	Ereendavaa terrane	Mylonitic granite	112.89	49.37	185	4.0	SHRIMP,U-Pb	Zircon	Miao Laicheng et al.	2017	Journal of Asian Earth Sciences
19	Within the MOO	C./S. Mongolia	2011MO-93	Ereendavaa terrane	Mylonitic granite	112.87	49.43	296	5.0	SHRIMP,U-Pb	Zircon	Miao Laicheng et al.	2017	Journal of Asian Earth Sciences
20	Within the MOO	C./S. Mongolia	2011MO-98	Ereendavaa terrane	Potassic granite	112.87	49.43	289	2.0	SHRIMP,U-Pb	Zircon	Miao Laicheng et al.	2017	Journal of Asian Earth Sciences
21	Within the MOO	C./S. Mongolia	HTA	Gal-Shara	Alkaline granite	110.68	46.98	213	1.0	TIMS,U-Pb	Zircon	Neimark et al.	1993	Doklady Akademii
22	Within the MOO	C./S. Mongolia	71	Bayan-Ulan massif	Alkaline granite	107.94	47.26	221	5.0	LA-ICP-MS,U-Pb	Zircon	Reichow et al.	2010	Chemical Geology
23	Within the MOO	C./S. Mongolia	57	Dzarkhyngol massif	Bi. granite	108.19	48.71	211	1.5	LA-ICP-MS,U-Pb	Zircon	Reichow et al.	2010	Chemical Geology
24	Within the MOO	C./S. Mongolia	M14926-1.2		Rhyolite	105.48	45.17	279	2.0	LA-ICP-MS,U-Pb	Zircon	This study		
25	Within the MOO	C./S. Mongolia	M14925-31.1		K-spar granite	105.65	45.23	313	1.0	LA-ICP-MS,U-Pb	Zircon	This study		
26	Within the MOO	C./S. Mongolia	M13722-22.1		K-spar granite	111.18	45.34	309	3.0	LA-ICP-MS,U-Pb	Zircon	This study		
27	Within the MOO	C./S. Mongolia	M14922-4.1		Syenogranite	106.81	45.36	244	1.0	LA-ICP-MS,U-Pb	Zircon	This study		
28	Within the MOO	C./S. Mongolia	M14925-9.1		Syenite	105.23	45.43	256	1.0	LA-ICP-MS,U-Pb	Zircon	This study		
29	Within the MOO	C./S. Mongolia	M13722-1.1		Granodiorite	111.22	45.60	302	2.0	LA-ICP-MS,U-Pb	Zircon	This study		
30	Within the MOO	C./S. Mongolia	M13720-9.1		Bi. Granodiorite	112.17	45.61	310	2.0	LA-ICP-MS,U-Pb	Zircon	This study		
31	Within the MOO	C./S. Mongolia	M14926-4.1		Bi.granite	105.56	45.63	241	1.0	LA-ICP-MS,U-Pb	Zircon	This study		
32	Within the MOO	C./S. Mongolia	M14923-5.1		Rhyolite	106.95	45.65	235	2.0	LA-ICP-MS,U-Pb	Zircon	This study		
33	Within the MOO	C./S. Mongolia	M14926-7.1		Alkali-feldspar granite	105.42	45.70	240	1.0	LA-ICP-MS,U-Pb	Zircon	This study		
34	Within the MOO	C./S. Mongolia	M14924-1.3		K-spar granite	107.10	45.70	250	2.0	LA-ICP-MS,U-Pb	Zircon	This study		
35	Within the MOO	C./S. Mongolia	M13723-12.1		Gnessic granite	111.62	45.70	299	1.0	LA-ICP-MS,U-Pb	Zircon	This study		
36	Within the MOO	C./S. Mongolia	M14921-4.1		Hb. quartz syenite	107.24	45.73	243	1.0	LA-ICP-MS,U-Pb	Zircon	This study		
37	Within the MOO	C./S. Mongolia	M13723-35.1		Gnessic granodiorite	110.67	45.76	300	2.0	LA-ICP-MS,U-Pb	Zircon	This study		
38	Within the MOO	C./S. Mongolia	M13720-7.1		Muscovite granite	112.75	45.80	321	4.0	LA-ICP-MS,U-Pb	Zircon	This study		
39	Within the MOO	C./S. Mongolia	M13720-6.1		Bi. monzogranite	112.75	45.80	314	15.0	LA-ICP-MS,U-Pb	Zircon	This study		
40	Within the MOO	C./S. Mongolia	M13720-1.1		Bi.granite	112.91	45.82	315	13.0	LA-ICP-MS,U-Pb	Zircon	This study		
41	Within the MOO	C./S. Mongolia	M13722-9.1		K-spar granite	110.94	45.83	288	2.0	LA-ICP-MS,U-Pb	Zircon	This study		
42	Within the MOO	C./S. Mongolia	M14921-14.1		K-spar granite	107.43	45.85	244	1.0	LA-ICP-MS,U-Pb	Zircon	This study		
43	Within the MOO	C./S. Mongolia	M13723-5.1		Bi.Monzogranite	110.71	45.85	294	4.0	LA-ICP-MS,U-Pb	Zircon	This study		
44	Within the MOO	C./S. Mongolia	M13720-2.1		Granite	112.88	45.90	295	3.0	LA-ICP-MS,U-Pb	Zircon	This study		
45	Within the MOO	C./S. Mongolia	M13722-7.2		K-spar granite	111.50	45.93	290	2.0	LA-ICP-MS,U-Pb	Zircon	This study		
46	Within the MOO	C./S. Mongolia	M13720-4.6		Muscovite granite	112.97	45.96	324	3.0	LA-ICP-MS,U-Pb	Zircon	This study		
47	Within the MOO	C./S. Mongolia	M13720-4.1		Granite	112.97	45.96	315	5.0	LA-ICP-MS,U-Pb	Zircon	This study		
48	Within the MOO	C./S. Mongolia	M13720-4.8		Granite	112.97	45.96	289	2.0	LA-ICP-MS,U-Pb	Zircon	This study		
49	Within the MOO	C./S. Mongolia	M13722-6.1		K-spar granite	113.50	45.97	301	2.0	LA-ICP-MS,U-Pb	Zircon	This study		
50	Within the MOO	C./S. Mongolia	M14921-10.1		K-spar granite	107.50	46.05	255	1.0	LA-ICP-MS,U-Pb	Zircon	This study		
51	Within the MOO	C./S. Mongolia	M14927-9.2		Dacite	107.04	46.26	269	2.0	LA-ICP-MS,U-Pb	Zircon	This study		
52	Within the MOO	C./S. Mongolia	M13714-11.1		Hb. Bi. monzogranite	114.45	46.28	311	2.0	LA-ICP-MS,U-Pb	Zircon	This study		
53	Within the MOO	C./S. Mongolia	M13714-9.1		Bi. monzogranite	114.31	46.32	307	3.0	LA-ICP-MS,U-Pb	Zircon	This study		
54	Within the MOO	C./S. Mongolia	M13714-4.1		Granite	114.07	46.33	315	2.0	LA-ICP-MS,U-Pb	Zircon	This study		
55	Within the MOO	C./S. Mongolia	M13714-5.4		Granite	114.07	46.34	157	2.0	LA-ICP-MS,U-Pb	Zircon	This study		
56	Within the MOO	C./S. Mongolia	M14927-14.1		K-spar granite	107.07	46.37	282	1.0	LA-ICP-MS,U-Pb	Zircon	This study		
57	Within the MOO	C./S. Mongolia	M13714-2.1		Granitic mylonite	113.71	46.37	293	2.0	LA-ICP-MS,U-Pb	Zircon	This study		
58	Within the MOO	C./S. Mongolia	M13713-14.3		K-spar granite	113.31	46.71	272	12.0	LA-ICP-MS,U-Pb	Zircon	This study		
59	Within the MOO	C./S. Mongolia	M13713-13.1		K-spar granite	113.34	46.78	257	3.0	LA-ICP-MS,U-Pb	Zircon	This study		
60	Within the MOO	C./S. Mongolia	M13713-10.1		Granodiorite	113.69	47.13	243	2.0	LA-ICP-MS,U-Pb	Zircon	This study		
61	Within the MOO	C./S. Mongolia	M13713-9.1		Bi. monzogranite	114.03	47.18	238	2.0	LA-ICP-MS,U-Pb	Zircon	This study		
62	Within the MOO	C./S. Mongolia	M1373-4.1		Rhyolite	109.26	47.54	304	2.0	LA-ICP-MS,U-Pb	Zircon	This study		
63	Within the MOO	C./S. Mongolia	M1372-14.1		Bi. monzogranite	109.09	47.56	205	3.0	LA-ICP-MS,U-Pb	Zircon	This study		
64	Within the MOO	C./S. Mongolia	M1372-9.1		Hb. granite	108.47	47.70	300	2.0	LA-ICP-MS,U-Pb	Zircon	This study		
65	Within the MOO	C./S. Mongolia	M1372-5.3		Hb. granite	108.09	47.76	216	3.0	LA-ICP-MS,U-Pb	Zircon	This study		
66	Within the MOO	C./S. Mongolia	M1372-10.1		Bi. monzogranite	108.64	47.76	301	2.0	LA-ICP-MS,U-Pb	Zircon	This study		
67	Within the MOO	C./S. Mongolia	M1375-2.1		Monzogranite	111.13	47.77	200	2.0	LA-ICP-MS,U-Pb	Zircon	This study		
68	Within the MOO	C./S. Mongolia	M13712-6.1		Monzogranite	114.36	47.79	179	4.0	LA-ICP-MS,U-Pb	Zircon	This study		
69	Within the MOO	C./S. Mongolia	M13712-5.1		Andisite	114.40	47.86	237	2.0	LA-ICP-MS,U-Pb	Zircon	This study		
70	Within the MOO	C./S. Mongolia	M13712-3.1		Granodiorite	114.47	47.95	272	4.0	LA-ICP-MS,U-Pb	Zircon	This study		
71	Within the MOO	C./S. Mongolia	M13712-2.1		Bi. monzogranite	114.52	47.99	254	3.0	LA-ICP-MS,U-Pb	Zircon	This study		
72	Within the MOO	C./S. Mongolia	M13710-3.1		Rhyolite	115.29	48.14	283	2.0	LA-ICP-MS,U-Pb	Zircon	This study		
73	Within the MOO	C./S. Mongolia	M1379-4.1		Monzogranite	113.63	48.40	169	1.0	LA-ICP-MS,U-Pb	Zircon	This study		

74	Within the MOO	C./S. Mongolia	M1377-6.3		Tonalite	111.72	48.80	150	2.0	LA-ICP-MS,U-Pb	Zircon	This study		
75	Within the MOO	C./S. Mongolia	M1377-5.1		Bi.granite	111.69	48.82	148	5.0	LA-ICP-MS,U-Pb	Zircon	This study		
76	Within the MOO	C./S. Mongolia	M1376-3.1		Bi. monzogranite	111.47	49.04	172	1.0	LA-ICP-MS,U-Pb	Zircon	This study		
77	Within the MOO	C./S. Mongolia	M1376-4.1		Enclave	111.43	49.05	172	1.0	LA-ICP-MS,U-Pb	Zircon	This study		
78	Within the MOO	C./S. Mongolia	M1376-4.4		Gnessic Bi.granite	111.43	49.05	172	1.0	LA-ICP-MS,U-Pb	Zircon	This study		
79	Within the MOO	C./S. Mongolia	M13720-3.1		Granite	112.93	45.93	240	4.0	LA-ICP-MS,U-Pb	Zircon	This study		
80	Within the MOO	C./S. Mongolia	58	Yudygym massif	Granite	109.21	48.16	211	0.3	LA-ICP-MS,U-Pb	Zircon	Tsygankov A A et al.	2007	Russian Geology and Geophysics
81	Within the MOO	C./S. Mongolia	M639a	Onon Shear	Pegmatite	111.64	48.85	154	4.0	LA-ICP-MS,U-Pb	Zircon	Yannick Daoudene et	2009	International Journal of Earth Sciences
82	Within the MOO	C./S. Mongolia	M616	Ereendavaa	Pegmatite	111.67	48.81	149		LA-ICP-MS,U-Pb	Zircon	Yannick Daoudene et	2013	Journal of Asian Earth Sciences
83	Within the MOO	C./S. Mongolia	M615a	Ereendavaa	Pegmatite	111.67	48.82	149		LA-ICP-MS,U-Pb	Zircon	Yannick Daoudene et	2013	Journal of Asian Earth Sciences
84	Within the MOO	C./S. Mongolia	M603a	Ereendavaa	Pegmatite	111.63	48.83	156		LA-ICP-MS,U-Pb	Zircon	Yannick Daoudene et	2013	Journal of Asian Earth Sciences
85	Within the MOO	C./S. Mongolia	73	Dashibalar volcanoplutoniccon	Comendites	109.49	46.84	196	3.9	TIMS,U-Pb	Zircon	Yarmolyuk V V, et al	1997	Petrology
86	Within the MOO	C./S. Mongolia	A0344	Janchivlan	rare-metal granites	107.58	47.55	227	8.0	Rb–Sr	Whole-ro	Yarmolyuk V V, et al	2002	Geotectonics
87	Within the MOO	C./S. Mongolia		Zhanchivlan massif	Bi. granite	106.50	47.73	217	2.7	TIMS,U-Pb	Zircon	Yarmolyuk V V, et al	2002	Geotectonics
88	Within the MOO	C./S. Mongolia		Zhanchivlan massif	Plagiogranite	106.50	47.73	207	6.8	TIMS,U-Pb	Zircon	Yarmolyuk V V, et al	2002	Geotectonics
89	Within the MOO	Erguna and adjacent areas	Z11-69	Quansheng	Bi. monzogranite	120.55	47.75	301	3.0	LA-ICP-MS,U-Pb	Zircon	Cui F H., et al.	2013	Acta Geologica Sinica(ICWEA)
90	Within the MOO	Erguna and adjacent areas	04GW025	XAS	XAS pluton monzogranite	121.65	48.83	308	3.0	SIMS,U-Pb	Zircon	Dong Y et al.	2015	Journal of Asian Earth Sciences
91	Within the MOO	Erguna and adjacent areas	04GW021	ESH	pluton diorite	121.72	48.77	294	4.0	SIMS,U-Pb	Zircon	Dong Y et al.	2015	Journal of Asian Earth Sciences
92	Within the MOO	Erguna and adjacent areas	14GW179	XAS	XAS pluton monzogranite	121.65	48.83	309	2.0	LA-ICP-MS,U-Pb	Zircon	Dong Y., et al.	2015	Journal of Asian Earth Sciences
93	Within the MOO	Erguna and adjacent areas	GW04244	Taerqi	Granodiorite	121.19	47.98	320	1.0	LA-ICP-MS,U-Pb	Zircon	Du X L.	2012	Jilin University: Phd Dissertation
94	Within the MOO	Erguna and adjacent areas	11HSW7-2	Sunwu area	Monzogranite	127.33	49.99	168		LA-ICP-MS,U-Pb	Zircon	Li M et al.	2015	Acta Petrologica Sinica(ICWEA)
95	Within the MOO	Erguna and adjacent areas	TP2R5-1	Daniujun-Wuqihaxi	Bi. granite	120.19	48.11	303	8.0	LA-ICP-MS,U-Pb	Zircon	Liu Shaung.	2016	Jilin University: Master Dissertation
96	Within the MOO	Erguna and adjacent areas	TED-22	Taerqi	Syenogranite	121.08	48.00	315	1.0	LA-ICP-MS,U-Pb	Zircon	She Hongquan et al.	2012	Acta Petrologica Sinica(ICWEA)
97	Within the MOO	Erguna and adjacent areas	TED-19	Tarqixi	Porphyritic Syenogranite	121.12	48.02	314	1.0	LA-ICP-MS,U-Pb	Zircon	She Hongquan et al.	2012	Acta Petrologica Sinica(ICWEA)
98	Within the MOO	Erguna and adjacent areas	TED-17	Tarqixi	Granodiorite	121.15	48.01	313	1.0	LA-ICP-MS,U-Pb	Zircon	She Hongquan et al.	2012	Acta Petrologica Sinica(ICWEA)
99	Within the MOO	Erguna and adjacent areas	TED-06	Taerqi	Granite	121.23	47.97	310	2.0	LA-ICP-MS,U-Pb	Zircon	She Hongquan et al.	2012	Acta Petrologica Sinica(ICWEA)
100	Within the MOO	Erguna and adjacent areas	TED-10	Taerqi	Quartz diorite	121.27	47.98	309	2.0	LA-ICP-MS,U-Pb	Zircon	She Hongquan et al.	2012	Acta Petrologica Sinica(ICWEA)
101	Within the MOO	Erguna and adjacent areas	HHEJ-01	Honghuaerjierdaoqiao	Bi. granite	120.44	48.18	278	3.0	LA-ICP-MS,U-Pb	Zircon	She Hongquan et al.	2012	Acta Petrologica Sinica (ICWEA)
102	Within the MOO	Erguna and adjacent areas	YLKD-06	Xinhualinxi	Granodiorite	121.66	48.82	301	1.0	LA-ICP-MS,U-Pb	Zircon	She Hongquan et al.	2012	Acta Petrologica Sinica(ICWEA)
103	Within the MOO	Erguna and adjacent areas	YL2	Yili	granite	124.28	49.45	288	1.8	SHRIMP,U-Pb	Zircon	Wu C. et al.	2014	International Geology Review
104	Within the MOO	Erguna and adjacent areas	2.89E+06	Guguhe	Alkali-feldspar granite	125.41	49.98	254	4.0	TIMS,U-Pb	Zircon	Wu Fuyuan et al.	2002	Chemical Geology
105	Within the MOO	Erguna and adjacent areas	9805—2	Sizhanlinchang	Syenogranite	125.41	49.91	282	4.0	TIMS,U-Pb	Zircon	Wu Fuyuan et al.	2002	Chemical Geology
106	Within the MOO	Erguna and adjacent areas	0029-2	Taerqi	Syenogranite	121.19	47.98	335	5.0	LA-ICP-MS,U-Pb	Zircon	Zhang J et al.	2011	Global Geology(ICWEA)
107	Within the MOO	Erguna and adjacent areas	Z11-68	Quansheng	Bi. syenogranite	120.03	47.89	295	2.0	LA-ICP-MS,U-Pb	Zircon	Cui F H., et al.	2013	Acta Geologica Sinica(ICWEA)
108	Within the MOO	Erguna and adjacent areas	Z11-71	Quansheng	Syenogranite	120.61	47.90	306	2.0	LA-ICP-MS,U-Pb	Zircon	Cui F H., et al.	2013	Acta Geologica Sinica(ICWEA)
109	Within the MOO	Erguna and adjacent areas	Z11-81	Quansheng	Syenogranite	120.98	47.91	322	1.0	LA-ICP-MS,U-Pb	Zircon	Cui F H., et al.	2013	Acta Geologica Sinica(ICWEA)
110	Within the MOO	Erguna and adjacent areas	XLG-2	Xiaokele porphyry Cu–Mo dep	Granidiorite porphyry	124.55	51.67	153	1.7	LA-ICP-MS,U-Pb	Zircon	Deng Chang-Zhou et	2019	Ore Geology Reviews
111	Within the MOO	Erguna and adjacent areas	XLG-34	Xiaokele porphyry Cu–Mo dep	Granidiorite porphyry	124.55	51.67	150	1.6	LA-ICP-MS,U-Pb	Zircon	Deng Chang-Zhou et	2019	Ore Geology Reviews
112	Within the MOO	Erguna and adjacent areas	XLG-35	Xiaokele porphyry Cu–Mo dep	Diorite porphyry	124.55	51.67	148	1.3	LA-ICP-MS,U-Pb	Zircon	Deng Chang-Zhou et	2019	Ore Geology Reviews
113	Within the MOO	Erguna and adjacent areas	11-31-148	Yihewula	Granitoid	125.49	52.39	301	9.3	SHRIMP,U-Pb	Zircon	Ding X	2010	Jilin University: Master Dissertation
114	Within the MOO	Erguna and adjacent areas	04GW023	XNG	XNG pluton diorite	121.68	48.72	308	3.0	LA-ICP-MS,U-Pb	Zircon	Dong Y et al.	2015	Journal of Asian Earth Sciences
115	Within the MOO	Erguna and adjacent areas	04GW016	XSL	pluton gabbro diorite	121.78	48.70	358	2.0	SIMS,U-Pb	Zircon	Dong Y et al.	2015	Journal of Asian Earth Sciences
116	Within the MOO	Erguna and adjacent areas	14GW190	ESH	ESH pluton diorite	121.72	48.77	294	2.0	LA-ICP-MS,U-Pb	Zircon	Dong Y., et al.	2015	Journal of Asian Earth Sciences
117	Within the MOO	Erguna and adjacent areas	GW04249	Jianchazhan	Monzogranite	121.23	47.96	313	3.0	LA-ICP-MS,U-Pb	Zircon	Du X L.	2012	Jilin University: Phd Dissertation
118	Within the MOO	Erguna and adjacent areas	GW04025	Xing'an	Monzogranite	121.67	48.82	267	3.0	LA-ICP-MS,U-Pb	Zircon	Du X L.	2012	Jilin University: Phd Dissertation
119	Within the MOO	Erguna and adjacent areas	GW04465	Jiageda	Granodiorite	124.38	50.38	165	1.0	LA-ICP-MS,U-Pb	Zircon	Du X L.	2012	Jilin University: Phd Dissertation
120	Within the MOO	Erguna and adjacent areas	0071-3	Ganhe	Monzogranite	123.17	50.68	304		LA-ICP-MS,U-Pb	Zircon	Du X L.	2012	Jilin University: Phd Dissertation
121	Within the MOO	Erguna and adjacent areas	GW04126	Bajianfang	Monzogranite	120.37	51.32	196	3.0	LA-ICP-MS,U-Pb	Zircon	Du X L.	2012	Jilin University: Phd Dissertation
122	Within the MOO	Erguna and adjacent areas	GW04123	Kutiankan	Quartz diorite	120.58	51.33	244	4.0	LA-ICP-MS,U-Pb	Zircon	Du X L.	2012	Jilin University: Phd Dissertation
123	Within the MOO	Erguna and adjacent areas	GW04114	Moerdaoga	Monzogranite	120.67	51.33	198	2.0	LA-ICP-MS,U-Pb	Zircon	Du X L.	2012	Jilin University: Phd Dissertation
124	Within the MOO	Erguna and adjacent areas	GW04038	Jinhezhen	Syenogranite	121.50	51.34	203	4.0	LA-ICP-MS,U-Pb	Zircon	Du X L.	2012	Jilin University: Phd Dissertation
125	Within the MOO	Erguna and adjacent areas	GW04039	Jinhezhen	Syenogranite	121.51	51.36	197	4.0	LA-ICP-MS,U-Pb	Zircon	Du X L.	2012	Jilin University: Phd Dissertation
126	Within the MOO	Erguna and adjacent areas	GW03017	Tayuan	Monzogranite	124.27	51.39	220	3.0	LA-ICP-MS,U-Pb	Zircon	Du X L.	2012	Jilin University: Phd Dissertation
127	Within the MOO	Erguna and adjacent areas	GW03015		Gabbro	124.38	51.50	322		LA-ICP-MS,U-Pb	Zircon	Du X L.	2012	Jilin University: Phd Dissertation
128	Within the MOO	Erguna and adjacent areas	GW03008		Monzogranite	124.38	51.50	318		LA-ICP-MS,U-Pb	Zircon	Du X L.	2012	Jilin University: Phd Dissertation
129	Within the MOO	Erguna and adjacent areas	GW04054	Aluga	Monzogranite	121.58	51.63	206	2.0	LA-ICP-MS,U-Pb	Zircon	Du X L.	2012	Jilin University: Phd Dissertation
130	Within the MOO	Erguna and adjacent areas	GW04061	Awuni	Diorite	121.89	51.85	208	1.0	LA-ICP-MS,U-Pb	Zircon	Du X L.	2012	Jilin University: Phd Dissertation
131	Within the MOO	Erguna and adjacent areas	GW03290	Mangui	Monzogranite	121.89	52.09	187	3.0	LA-ICP-MS,U-Pb	Zircon	Du X L.	2012	Jilin University: Phd Dissertation
132	Within the MOO	Erguna and adjacent areas	GW03269	Mangui	Syenogranite	122.07	52.13	189	2.0	LA-ICP-MS,U-Pb	Zircon	Du X L.	2012	Jilin University: Phd Dissertation
133	Within the MOO	Erguna and adjacent areas	GW04069	Manguixi	Monzogranite	121.33	52.42	195	2.0	LA-ICP-MS,U-Pb	Zircon	Du X L.	2012	Jilin University: Phd Dissertation
134	Within the MOO	Erguna and adjacent areas	GW03193	Lvlin	Alkali-feldspar granite	123.08	52.47	187	2.0	LA-ICP-MS,U-Pb	Zircon	Du X L.	2012	Jilin University: Phd Dissertation
135	Within the MOO	Erguna and adjacent areas	GW03255	Fukeshan	Monzogranite	121.87	52.48	194	7.0	LA-ICP-MS,U-Pb	Zircon	Du X L.	2012	Jilin University: Phd Dissertation
136	Within the MOO	Erguna and adjacent areas	GW04077	Manguixi	Syenogranite	121.08	52.51	220	3.0	LA-ICP-MS,U-Pb	Zircon	Du X L.	2012	Jilin University: Phd Dissertation
137	Within the MOO	Erguna and adjacent areas	GW04098	Guanhuzhannan	Monzogranite	120.96	52.56	201	1.0	LA-ICP-MS,U-Pb	Zircon	Du X L.	2012	Jilin University: Phd Dissertation
138	Within the MOO	Erguna and adjacent areas	GW03251	Fukeshan	Monzogranite	121.89	52.64	189	2.0	LA-ICP-MS,U-Pb	Zircon	Du X L.	2012	Jilin University: Phd Dissertation
139	Within the MOO	Erguna and adjacent areas	GW03138	Pangu	Monzogranite	123.69	52.64	182	1.0	LA-ICP-MS,U-Pb	Zircon	Du X L.	2012	Jilin University: Phd Dissertation
140	Within the MOO	Erguna and adjacent areas	GW03177	Lvlin	Granitic gneiss	123.15	52.65	209	4.0	LA-ICP-MS,U-Pb	Zircon	Du X L.	2012	Jilin University: Phd Dissertation
141	Within the MOO	Erguna and adjacent areas	GW03181	Lvlin	Quartz diorite	123.15	52.65	192	3.0	LA-ICP-MS,U-Pb	Zircon	Du X L.	2012	Jilin University: Phd Dissertation
142	Within the MOO	Erguna and adjacent areas	GW03241	Fukeshan	Monzogranite	121.92	52.70	189	2.0	LA-ICP-MS,U-Pb	Zircon	Du X L.	2012	Jilin University: Phd Dissertation
143	Within the MOO	Erguna and adjacent areas	GW03207	Xilinji	Monzogranite	123.53	52.82	193	2.0	LA-ICP-MS,U-Pb	Zircon	Du X L.	2012	Jilin University: Phd Dissertation
144	Within the MOO	Erguna and adjacent areas	GW04088	Guanhuzhan	Felsic vein	120.72	52.89	249	4.0	LA-ICP-MS,U-Pb	Zircon	Du X L.	2012	Jilin University: Phd Dissertation
145	Within the MOO	Erguna and adjacent areas	GW04021	Zhufeng	Diorite	121.70	48.81	249	2.0	LA-ICP-MS,U-Pb	Zircon	Du X L.	2012	Jilin University: Phd Dissertation
146	Within the MOO	Erguna and adjacent areas	TY902-1			124.00	51.00	331	2.6	SIMS,U-Pb	Zircon	Feng Z Q., et al.	2014	Acta Petrologica Sinica(ICWEA)
147	Within the MOO	Erguna and adjacent areas	TY902-2			124.00	51.00	329	2.4	SIMS,U-Pb	Zircon	Feng Z Q., et al.	2014	Acta Petrologica Sinica(ICWEA)
148	Within the MOO	Erguna and adjacent areas	TY02		Metagabbros	124.28	51.50	315	2.5	SIMS,U-Pb	Zircon	Feng Z Q., et al.	2015	Journal of Asian Earth Sciences

149	Within the MOO	Erguna and adjacent areas	TY05		Metagabbros	124.38	51.50	312	2.8	SIMS,U-Pb	Zircon	Feng Z Q., et al.	2015	Journal of Asian Earth Sciences
150	Within the MOO	Erguna and adjacent areas	GW04512	Sankuanggou	Granodiorite	125.65	50.38	177	3.0	LA-ICP-MS,U-Pb	Zircon	Ge W C., et al.	2007	Chinese Science Bulletin
151	Within the MOO	Erguna and adjacent areas	GW04516	Huaduoshan	Granodiorite	125.73	50.38	176	3.0	LA-ICP-MS,U-Pb	Zircon	Ge W C., et al.	2007	Chinese Science Bulletin
152	Within the MOO	Erguna and adjacent areas	M030-1		Monzogranite	116.26	48.99	252		LA-ICP-MS,U-Pb	Zircon	Gou J et al.	2013	Journal of Jilin University (Earth Science Edition)(ICWEA)
153	Within the MOO	Erguna and adjacent areas	1076-1	Suwu-Jiayin	Granite	127.14	50.18	187	2.0	LA-ICP-MS,U-Pb	Zircon	Gou J et al.	2013	Journal of Jilin University (Earth Science Edition)(ICWEA)
154	Within the MOO	Erguna and adjacent areas	1055-1	Suwu-Jiayin	Granite	127.14	50.18	181	2.0	LA-ICP-MS,U-Pb	Zircon	Gou J et al.	2013	Journal of Jilin University (Earth Science Edition)(ICWEA)
155	Within the MOO	Erguna and adjacent areas	1055-1	Heihe	Granodiorite	127.14	50.18	181	2.0	LA-ICP-MS,U-Pb	Zircon	Gou J et al.	2013	Journal of Jilin University（Earth Science Edition）(ICWEA)
156	Within the MOO	Erguna and adjacent areas	M543-5	Manzhouli–Ergun	Granodiorite	116.14	48.64	251		LA-ICP-MS,U-Pb	Zircon	Gou Jun et al.	2013	Journal of Asian Earth Sciences
157	Within the MOO	Erguna and adjacent areas	M196-1	Manzhouli–Ergun	Syenogranite	119.94	49.78	256		LA-ICP-MS,U-Pb	Zircon	Gou Jun et al.	2013	Journal of Asian Earth Sciences
158	Within the MOO	Erguna and adjacent areas	SKG-2	Sankuanggou	Granodiorite	125.66	50.38	176	0.3	LA-ICP-MS,U-Pb	Zircon	Hao Y J et al.	2015	Journal of Asian Earth Sciences
159	Within the MOO	Erguna and adjacent areas	TS-6	Tongshan	Porphyritic granite	125.82	50.22	231	0.9	LA-ICP-MS,U-Pb	Zircon	Hao Y J., et al.	2015	Journal of Asian Earth Sciences
160	Within the MOO	Erguna and adjacent areas	DHS-0331	Daheishan	Porphyritic granite	124.47	50.52	150	4.2	LA-ICP-MS,U-Pb	Zircon	Hu X L., et al.	2014	Resource Geology
161	Within the MOO	Erguna and adjacent areas	DHS-0151	Daheishan	Ganodiorite	124.47	50.52	147	1.1	LA-ICP-MS,U-Pb	Zircon	Hu X L., et al.	2014	Resource Geology
162	Within the MOO	Erguna and adjacent areas	B1305		Granite	119.12	49.98	244		LA-ICP-MS,U-Pb	Zircon	Kang Y J et al.	2014	Geology in China(ICWEA)
163	Within the MOO	Erguna and adjacent areas	B1323	Badaguan ore district	Granitic porphyry	119.13	49.98	232		LA-ICP-MS,U-Pb	Zircon	Kang Y J et al.	2014	Geology in China(ICWEA)
164	Within the MOO	Erguna and adjacent areas	B1313		Granite-prophyry	119.15	49.98	237		LA-ICP-MS,U-Pb	Zircon	Kang Y J et al.	2014	Geology in China(ICWEA)
165	Within the MOO	Erguna and adjacent areas	B1314		Granite-prophyry	119.16	49.98	241		LA-ICP-MS,U-Pb	Zircon	Kang Y J et al.	2014	Geology in China(ICWEA)
166	Within the MOO	Erguna and adjacent areas	B1321	Badaguan ore district	Biotite granite	119.11	49.99	240		LA-ICP-MS,U-Pb	Zircon	Kang Y J et al.	2014	Geology in China(ICWEA)
167	Within the MOO	Erguna and adjacent areas	B1316		Syenogranite	119.13	49.99	241		LA-ICP-MS,U-Pb	Zircon	Kang Y J et al.	2014	Geology in China(ICWEA)
168	Within the MOO	Erguna and adjacent areas	A-23	Eastern part of Amazar–Gilyui	Quartz diorite	127.77	54.10	177	3.0	LA-ICP-MS,U-Pb	Zircon	Kotov A.V. et al.	2012	Doklady Earth Sciences
169	Within the MOO	Erguna and adjacent areas	LA-400	Central part of Amazar–Gilyui	Quartz diorite	123.60	54.30	173	1.0	LA-ICP-MS,U-Pb	Zircon	Kotov A.V. et al.	2012	Doklady Earth Sciences
170	Within the MOO	Erguna and adjacent areas	1530	Huolongmen	Monzonitic granite	126.04	50.17	352	3.5	SHRIMP,U-Pb	Zircon	Li C L et al.	2013	Geology in China(ICWEA)
171	Within the MOO	Erguna and adjacent areas	M8	Mohe	Monzogranite	122.68	52.80	187	5.0	SHRIMP,U-Pb	Zircon	Li Jinyi et al.	2004	Geological Bulletin of China
172	Within the MOO	Erguna and adjacent areas	MH-N2	Mohe	Monzogranite	122.11	53.10	212		LA-ICP-MS,U-Pb	Zircon	Li Liang et al.	2016	Resource Geology
173	Within the MOO	Erguna and adjacent areas	MH-N1	Mohe	Syenogranite	121.87	53.26	245		LA-ICP-MS,U-Pb	Zircon	Li Liang et al.	2016	Resource Geology
174	Within the MOO	Erguna and adjacent areas	15ER1-1	Halatusumu	Granodiorite	119.76	49.65	303	2.0	LA-ICP-MS,U-Pb	Zircon	Li Y., et al.	2017	Journal of Asian Earth Sciences
175	Within the MOO	Erguna and adjacent areas	15ER12	Qiyi pasture	Monzogranite	119.93	49.79	238	1.0	LA-ICP-MS,U-Pb	Zircon	Li Y., et al.	2017	Journal of Asian Earth Sciences
176	Within the MOO	Erguna and adjacent areas	15ER2	Erguna Massif	Quartz monzonite	120.05	51.01	240		LA-ICP-MS,U-Pb	Zircon	Li Y., et al.	2017	Journal of Asian Earth Sciences
177	Within the MOO	Erguna and adjacent areas	15ER4-1	Northern Moerdaoga	Monzogranite	120.75	51.55	239	3.0	LA-ICP-MS,U-Pb	Zircon	Li Y., et al.	2017	Journal of Asian Earth Sciences
178	Within the MOO	Erguna and adjacent areas	15ER4	Erguna Massif	Monzogranite	120.75	51.55	239		LA-ICP-MS,U-Pb	Zircon	Li Y., et al.	2017	Journal of Asian Earth Sciences
179	Within the MOO	Erguna and adjacent areas	15ER9	Erguna Massif	Monzogranite	120.92	51.88	246		LA-ICP-MS,U-Pb	Zircon	Li Y., et al.	2017	Journal of Asian Earth Sciences
180	Within the MOO	Erguna and adjacent areas	14ER9	Erguna Massif	Monzonite	123.61	52.40	246		LA-ICP-MS,U-Pb	Zircon	Li Y., et al.	2017	Journal of Asian Earth Sciences
181	Within the MOO	Erguna and adjacent areas	14ER16	Mohe–Luoguhe highway	Granodiorite	121.87	53.15	256	1.0	LA-ICP-MS,U-Pb	Zircon	Li Y., et al.	2017	Journal of Asian Earth Sciences
182	Within the MOO	Erguna and adjacent areas	HSW11		Basaltic andesite	127.08	49.94	242		LA-ICP-MS,U-Pb	Zircon	Li Y., et al.	2017	Gondwana Research
183	Within the MOO	Erguna and adjacent areas	13XA10	Xing'an Massif	Monzogranite	120.58	48.46	164		LA-ICP-MS,U-Pb	Zircon	Li Y., et al.	2018	Lithos
184	Within the MOO	Erguna and adjacent areas	13XA10-1	Xing'an Massif	Monzogranite	120.58	48.46	164		LA-ICP-MS,U-Pb	Zircon	Li Y., et al.	2018	Lithos
185	Within the MOO	Erguna and adjacent areas	16ER43-1	Xing'an Massif	Granodiorite	125.65	50.38	174		LA-ICP-MS,U-Pb	Zircon	Li Y., et al.	2018	Lithos
186	Within the MOO	Erguna and adjacent areas	13HH13	Xing'an Massif	Granodiorite	125.70	50.30	237		LA-ICP-MS,U-Pb	Zircon	Li Y., et al.	2018	Lithos
187	Within the MOO	Erguna and adjacent areas	13HH13-1	Xing'an Massif	Granodiorite	125.70	50.30	237		LA-ICP-MS,U-Pb	Zircon	Li Y., et al.	2018	Lithos
188	Within the MOO	Erguna and adjacent areas	13XA19	Xing'an Massif	Monzogranite	121.31	49.60	236		LA-ICP-MS,U-Pb	Zircon	Li Y., et al.	2018	Lithos
189	Within the MOO	Erguna and adjacent areas	13HH18-1	Xing'an Massif	Granodiorite	125.79	50.25	223		LA-ICP-MS,U-Pb	Zircon	Li Y., et al.	2018	Lithos
190	Within the MOO	Erguna and adjacent areas	13HH8	Xing'an Massif	Granodiorite	125.79	50.25	223		LA-ICP-MS,U-Pb	Zircon	Li Y., et al.	2018	Lithos
191	Within the MOO	Erguna and adjacent areas	CL-02	Chalukou	Monzogranite	123.88	51.17	172	2.0	LA-ICP-MS,U-Pb	Zircon	Li Z Z et al.	2014	Lithos
192	Within the MOO	Erguna and adjacent areas	ZK1503-1197	Chalukou	Monzogranite	123.88	51.17	163	1.0	LA-ICP-MS,U-Pb	Zircon	Li Zh Zh., et al.	2014	Lithos
193	Within the MOO	Erguna and adjacent areas	ZK808-1257	Chalukou	Aplite porphyry	123.88	51.17	148	1.0	LA-ICP-MS,U-Pb	Zircon	Li Zh Zh., et al.	2014	Lithos
194	Within the MOO	Erguna and adjacent areas	ZK1301-475	Chalukou	Granite porphyry	123.88	51.17	147	3.0	LA-ICP-MS,U-Pb	Zircon	Li Zh Zh., et al.	2014	Lithos
195	Within the MOO	Erguna and adjacent areas	ZK1702-289.5	Chalukou	Quartz porphyry	123.88	51.17	147	1.0	LA-ICP-MS,U-Pb	Zircon	Li Zh Zh., et al.	2014	Lithos
196	Within the MOO	Erguna and adjacent areas	17CG1-2	Erguna Massif	Granitic gneiss	118.34	49.46	245		LA-ICP-MS,U-Pb	Zircon	Liang C Y., et al.	2019	Geological Journal
197	Within the MOO	Erguna and adjacent areas	TW15		monzogranite	116.23	49.01	264		LA-ICP-MS,U-Pb	zircon	Liu H Ch., et al.	2018	Lithos
198	Within the MOO	Erguna and adjacent areas	TW6550	Delidenihaermode area	Granite	120.20	47.60	173		LA-ICP-MS,U-Pb	Zircon	Liu H Ch., et al.	2018	Lithos
199	Within the MOO	Erguna and adjacent areas	TW5892	Aobaowula volcanic vent	Granite	116.32	49.03	267		LA-ICP-MS,U-Pb	Zircon	Liu H Ch., et al.	2018	Lithos
200	Within the MOO	Erguna and adjacent areas	TW6137	Delidenihaermode area	Granite	120.22	47.40	241		LA-ICP-MS,U-Pb	Zircon	Liu H Ch., et al.	2018	Lithos
201	Within the MOO	Erguna and adjacent areas	TW6136	Delidenihaermode area	Granite	120.22	47.37	216		LA-ICP-MS,U-Pb	Zircon	Liu H Ch., et al.	2018	Lithos
202	Within the MOO	Erguna and adjacent areas	HD-243	Chalukou deposit in N Great X	Monzogranite	123.88	51.16	162	1.6	LA-ICP-MS,U-Pb	Zircon	Liu J et al.	2013	Acta Geologica Sinica(ICWEA)
203	Within the MOO	Erguna and adjacent areas	HD-273		Quartz porphyry	123.88	51.16	149		LA-ICP-MS,U-Pb	Zircon	Liu J et al.	2013	Acta Geologica Sinica(ICWEA)
204	Within the MOO	Erguna and adjacent areas	HD-47		Granite porphyry	123.88	51.16	148		LA-ICP-MS,U-Pb	Zircon	Liu J et al.	2013	Acta Geologica Sinica(ICWEA)
205	Within the MOO	Erguna and adjacent areas	HX-9		fine-grained granite	123.88	51.16	148		LA-ICP-MS,U-Pb	Zircon	Liu J et al.	2013	Acta Geologica Sinica(ICWEA)
206	Within the MOO	Erguna and adjacent areas	TP2R8-3		Ms. monzogranite	120.22	48.05	259	11.0	LA-ICP-MS,U-Pb	Zircon	Liu S.	2016	Master Thesis: Jilin University
207	Within the MOO	Erguna and adjacent areas	TP3R12		Porphyritic Monzogranite	120.24	48.03	239	8.0	LA-ICP-MS,U-Pb	Zircon	Liu S.	2016	Master Thesis: Jilin University
208	Within the MOO	Erguna and adjacent areas	P002LT1		Molynitic Monzogranite	126.70	50.96	293	2.1	TIMS,U-Pb	Zircon	Liu Y et al.	2011	Geology and Resources(ICWEA)
209	Within the MOO	Erguna and adjacent areas	P008TC12		Syenogranite	126.78	50.99	310	2.6	TIMS,U-Pb	Zircon	Liu Y et al.	2011	Geology and Resources(ICWEA)
210	Within the MOO	Erguna and adjacent areas	P11LT2		Granodiorite	126.41	51.10	306	2.0	TIMS,U-Pb	Zircon	Liu Y et al.	2011	Geology and Resources(ICWEA)
211	Within the MOO	Erguna and adjacent areas	12BDG-10		granodioritic porphyry	119.13	49.99	238		LA-ICP-MS,U-Pb	Zircon	Mi K F et al.	2017	Ore Geology Reviews
212	Within the MOO	Erguna and adjacent areas	14-WS-12	Badaguan	monzogranitic porphyry	117.28	49.42	192		LA-ICP-MS,U-Pb	Zircon	Mi K F. et al.	2017	Ore Geology Reviews
213	Within the MOO	Erguna and adjacent areas	14BDG-80	Badaguan	granitic porphyry	119.14	49.98	230		LA-ICP-MS,U-Pb	Zircon	Mi K F. et al.	2017	Ore Geology Reviews
214	Within the MOO	Erguna and adjacent areas	2002XKL-2	Lengchuan	Granodiorite	126.91	50.22	164	4.0	SHRIMP,U-Pb	Zircon	Miao L C et al.	2003	Chinese Science Bulletin (in Chinese)
215	Within the MOO	Erguna and adjacent areas	2002XKL-7	Mengluhedingzi	Bi. monzogranite	126.80	50.26	167	4.0	SHRIMP,U-Pb	Zircon	Miao L C et al.	2003	Chinese Science Bulletin (in Chinese)
216	Within the MOO	Erguna and adjacent areas	2002YW-26	Guyuan	Plagiogranite	124.31	50.91	226	2.0	SHRIMP,U-Pb	Zircon	Miao L C et al.	2003	Chinese Academy of Sciences
217	Within the MOO	Erguna and adjacent areas	2002XT-11	Xintian	Felsic dyke	124.20	50.15	208	2.0	SHRIMP,U-Pb	Zircon	Miao L C et al.	2003	Chinese Academy of Sciences
218	Within the MOO	Erguna and adjacent areas	0-567	Yikete	Syenogranite	125.82	49.96	309	3.0	SHRIMP,U-Pb	Zircon	Qu H et al.	2011	Geology in China(ICWEA)
219	Within the MOO	Erguna and adjacent areas	1411	Dalanggou	Alkali granite	125.98	49.99	299	3.0	SHRIMP,U-Pb	Zircon	Qu H et al.	2011	Geology in China(ICWEA)
220	Within the MOO	Erguna and adjacent areas	Mor-19	moerdaoga	Monzogranite	120.60	51.29	205	3.0	LA-ICP-MS,U-Pb	Zircon	She Hongquan et al.	2011	Journal of Jilin University (Science Edition)
221	Within the MOO	Erguna and adjacent areas	Mor-2	moerdaoga	Giant spot moyite	120.44	51.31	203	2.0	LA-ICP-MS,U-Pb	Zircon	She Hongquan et al.	2011	Journal of Jilin University (Science Edition)
222	Within the MOO	Erguna and adjacent areas	Mor-20	moerdaoga	Diorite	120.58	51.33	244	4.0	LA-ICP-MS,U-Pb	Zircon	She Hongquan et al.	2011	Journal of Jilin University (Science Edition)
223	Within the MOO	Erguna and adjacent areas	Mor-1	moerdaoga	Monzogranite	120.31	51.35	201	3.0	LA-ICP-MS,U-Pb	Zircon	She Hongquan et al.	2011	Journal of Jilin University (Science Edition)

224	Within the MOO	Erguna and adjacent areas	KDJ-1	Kadajiling	Granodiorite	121.82	51.41	201		LA-ICP-MS,U-Pb	Zircon	She Hongquan et al.	2011	Journal of Jilin University(Earth Science Edition)
225	Within the MOO	Erguna and adjacent areas	Mor-3	Fenshuishan	Biotite hornite plagioclase gneiss	120.89	51.47	200	2.0	LA-ICP-MS,U-Pb	Zircon	She Hongquan et al.	2011	Journal of Jilin University (Science Edition)
226	Within the MOO	Erguna and adjacent areas	Mor-4	Fenshuishan	Muscovite granite	120.89	51.47	200	5.0	LA-ICP-MS,U-Pb	Zircon	She Hongquan et al.	2011	Journal of Jilin University (Science Edition)
227	Within the MOO	Erguna and adjacent areas	Mor-17	moerdaoga	Monzogranite	120.83	51.48	244		LA-ICP-MS,U-Pb	Zircon	She Hongquan et al.	2011	Journal of Jilin University (Science Edition)
228	Within the MOO	Erguna and adjacent areas	Mor-18	moerdaoga	Monzogranite	120.81	51.49	203	3.0	LA-ICP-MS,U-Pb	Zircon	She Hongquan et al.	2011	Journal of Jilin University (Science Edition)
229	Within the MOO	Erguna and adjacent areas	TER-01	Tarqi	Granodiorite	120.80	48.01	308	1.0	LA-ICP-MS,U-Pb	Zircon	She Hongquan et al.	2012	Acta Petrologica Sinica(ICWEA)
230	Within the MOO	Erguna and adjacent areas	TED-16	Tarqixi	Syenogranite	121.15	48.01	313	2.0	LA-ICP-MS,U-Pb	Zircon	She Hongquan et al.	2012	Acta Petrologica Sinica(ICWEA)
231	Within the MOO	Erguna and adjacent areas	TED-26	Tarqi	Syenogranite	121.21	48.06	309	1.0	LA-ICP-MS,U-Pb	Zircon	She Hongquan et al.	2012	Acta Petrologica Sinica(ICWEA)
232	Within the MOO	Erguna and adjacent areas	TED-07	Taerqi	Syenogranite	121.23	47.97	314	2.0	LA-ICP-MS,U-Pb	Zircon	She Hongquan et al.	2012	Acta Petrologica Sinica(ICWEA)
233	Within the MOO	Erguna and adjacent areas	HHEJ-09	Honghuaerjierdaoqiao	Porphyritic Granite	120.41	48.12	289	2.0	LA-ICP-MS,U-Pb	Zircon	She Hongquan et al.	2012	Acta Petrologica Sinica (ICWEA)
234	Within the MOO	Erguna and adjacent areas	TRQ-01	Tarqi	Granodiorite	120.80	48.03	308	1.0	LA-ICP-MS,U-Pb	Zircon	She Hongquan et al.	2012	Acta Petrologica Sinica(ICWEA)
235	Within the MOO	Erguna and adjacent areas	TED-05	Tarqi	Syenogranite	121.28	47.83	164	1.0	LA-ICP-MS,U-Pb	Zircon	She Hongquan et al.	2012	Acta Petrologica Sinica(ICWEA)
236	Within the MOO	Erguna and adjacent areas	LZS-01	Lizishan	Granodiorite	120.83	48.36	161	3.0	LA-ICP-MS,U-Pb	Zircon	She Hongquan et al.	2012	Acta Petrologica Sinica
237	Within the MOO	Erguna and adjacent areas	BKT-02	Boketu-Zalantun	Granite	122.08	48.62	147	1.0	LA-ICP-MS,U-Pb	Zircon	She Hongquan et al.	2012	Journal of Jilin University (Earth Science Edition)(ICWEA)
238	Within the MOO	Erguna and adjacent areas	CGB-33	Chagan	Granite	116.34	48.75	260	2.0	LA-ICP-MS,U-Pb	Zircon	She Hongquan et al.	2012	Journal of Jilin University (Earth Science Edition)(ICWEA)
239	Within the MOO	Erguna and adjacent areas	W-16	Wunver	Granite	121.20	48.99	151	1.0	LA-ICP-MS,U-Pb	Zircon	She Hongquan et al.	2012	Acta Petrologica Sinica(ICWEA)
240	Within the MOO	Erguna and adjacent areas	WNE-19	Mianduhe	Granite	121.04	49.04	295	0.5	LA-ICP-MS,U-Pb	Zircon	She Hongquan et al.	2012	Acta Petrologica Sinica(ICWEA)
241	Within the MOO	Erguna and adjacent areas	WNE-18	Mianduhe	Bi. granite	121.05	49.05	300	1.0	LA-ICP-MS,U-Pb	Zircon	She Hongquan et al.	2012	Acta Petrologica Sinica(ICWEA)
242	Within the MOO	Erguna and adjacent areas	W-1		Biotite granite	120.03	49.38	204		LA-ICP-MS,U-Pb	Zircon	She Hongquan et al.	2012	Acta Petrologica Sinica(ICWEA)
243	Within the MOO	Erguna and adjacent areas	WLS-15	Wunvgetu	Bi. granite	117.32	49.41	198	3.0	LA-ICP-MS,U-Pb	Zircon	She Hongquan et al.	2012	Acta Petrologica Sinica(ICWEA)
244	Within the MOO	Erguna and adjacent areas	WLS-10	Wunvgetu	Bi. granite	117.29	49.43	203	3.0	LA-ICP-MS,U-Pb	Zircon	She Hongquan et al.	2012	Acta Petrologica Sinica(ICWEA)
245	Within the MOO	Erguna and adjacent areas	WLS-12	Wunvgetu	Porphyry	117.29	49.43	196	4.0	LA-ICP-MS,U-Pb	Zircon	She Hongquan et al.	2012	Acta Petrologica Sinica(ICWEA)
246	Within the MOO	Erguna and adjacent areas	WS-12	Wunvgetu	Granitic porphyry	117.29	49.43	182	3.0	SHRIMP,U-Pb	Zircon	She Hongquan et al.	2012	Acta Petrologica Sinica(ICWEA)
247	Within the MOO	Erguna and adjacent areas	WS-10	Wunvgetu	Granite	117.29	49.43	180	1.0	LA-ICP-MS,U-Pb	Zircon	She Hongquan et al.	2012	Acta Petrologica Sinica(ICWEA)
248	Within the MOO	Erguna and adjacent areas	WLS-07	Wunvgetu	Monzogranitic porphyry	117.30	49.43	204	2.0	LA-ICP-MS,U-Pb	Zircon	She Hongquan et al.	2012	Acta Petrologica Sinica(ICWEA)
249	Within the MOO	Erguna and adjacent areas	WLS-7	Wunvgetu	Monzogranitic porphyry	117.30	49.43	204	3.0	LA-ICP-MS,U-Pb	Zircon	She Hongquan et al.	2012	Acta Petrologica Sinica(ICWEA)
250	Within the MOO	Erguna and adjacent areas	YLB-11	Yuanlin	Bi. granite	121.14	49.83	314	5.0	LA-ICP-MS,U-Pb	Zircon	She Hongquan et al.	2012	Acta Petrologica Sinica(ICWEA)
251	Within the MOO	Erguna and adjacent areas	Y268	Yuanlin	Quartz porphyry vein	121.10	49.85	318	6.0	LA-ICP-MS,U-Pb	Zircon	She Hongquan et al.	2012	Acta Petrologica Sinica(ICWEA)
252	Within the MOO	Erguna and adjacent areas	TS-04	Tongshan	Granodiorite	125.79	50.25	167	1.0	LA-ICP-MS,U-Pb	Zircon	She Hongquan et al.	2012	Acta Petrologica Sinica(ICWEA)
253	Within the MOO	Erguna and adjacent areas	JGD-01	Wodouhe	Bi. granite	125.79	50.54	319	1.0	LA-ICP-MS,U-Pb	Zircon	She Hongquan et al.	2012	Acta Petrologica Sinica
254	Within the MOO	Erguna and adjacent areas	JGD-03	Wodouhe	Granite	125.79	50.54	171	1.0	LA-ICP-MS,U-Pb	Zircon	She Hongquan et al.	2012	Acta Petrologica Sinica
255	Within the MOO	Erguna and adjacent areas	GX-01	Gexian	Syenitic porphyry	123.55	50.63	266	1.0	LA-ICP-MS,U-Pb	Zircon	She Hongquan et al.	2012	Acta Petrologica Sinica (ICWEA)
256	Within the MOO	Erguna and adjacent areas	JGD-15	Xiaoyangqidong	Gnessic granite	124.70	50.89	223	1.0	LA-ICP-MS,U-Pb	Zircon	She Hongquan et al.	2012	Acta Petrologica Sinica
257	Within the MOO	Erguna and adjacent areas	JGD-23	Jinsongqiaodong	Granite	124.23	51.13	288	2.0	LA-ICP-MS,U-Pb	Zircon	She Hongquan et al.	2012	Acta Petrologica Sinica
258	Within the MOO	Erguna and adjacent areas	JGD-29	Jinsongqiaodong	Granite	124.39	51.20	209	1.0	LA-ICP-MS,U-Pb	Zircon	She Hongquan et al.	2012	Acta Petrologica Sinica
259	Within the MOO	Erguna and adjacent areas	JGD-30	Xintianzhengbei	Gnessic ranite	124.39	51.23	188	3.0	LA-ICP-MS,U-Pb	Zircon	She Hongquan et al.	2012	Acta Petrologica Sinica
260	Within the MOO	Erguna and adjacent areas	SKG-61	Sankuanggou	Bi. granite	125.72	51.36	178	0.4	LA-ICP-MS,U-Pb	Zircon	She Hongquan et al.	2012	Acta Petrologica Sinica(ICWEA)
261	Within the MOO	Erguna and adjacent areas	Mor14	moerdaoga	Monzogranite	120.92	51.88	259		LA-ICP-MS,U-Pb	Zircon	She Hongquan et al.	2012	Acta Petrologica Sinica
262	Within the MOO	Erguna and adjacent areas	BKT-17	Boketu-Zalantun	Monzogranite	122.74	48.70	230	1.0	LA-ICP-MS,U-Pb	Zircon	She Hongquan et al.	2012	Journal of Jilin University (Earth Science Edition)(ICWEA)
263	Within the MOO	Erguna and adjacent areas	Z11-82	Jinjianggou	Syenogranite	120.10	47.23	322	1.0	LA-ICP-MS,U-Pb	Zircon	Shi L., et al.	2014	Journal of Asian Earth Sciences
264	Within the MOO	Erguna and adjacent areas	Z11-64	Jinjianggou	Monzogranite	120.27	47.21	150	1.0	LA-ICP-MS,U-Pb	Zircon	Shi L., et al.	2014	Journal of Asian Earth Sciences
265	Within the MOO	Erguna and adjacent areas	Z11-72	Suhetun	Monzogranite	120.21	47.65	174	1.0	LA-ICP-MS,U-Pb	Zircon	Shi L., et al.	2014	Journal of Asian Earth Sciences
266	Within the MOO	Erguna and adjacent areas	Z11-67	Suhetun	Monzogranite	120.34	47.67	172	1.0	LA-ICP-MS,U-Pb	Zircon	Shi L., et al.	2014	Journal of Asian Earth Sciences
267	Within the MOO	Erguna and adjacent areas	Z11-80	Quansheng	Porphyritic quartz monzonite	120.90	47.88	179	1.0	LA-ICP-MS,U-Pb	Zircon	Shi L., et al.	2014	Journal of Asian Earth Sciences
268	Within the MOO	Erguna and adjacent areas	GW05157	Shierzhan	Monzogranite	125.68	51.20	300	2.0	LA-ICP-MS,U-Pb	Zircon	Sui Z M et al.	2009	Acta Petrologica Sinica(ICWEA)
269	Within the MOO	Erguna and adjacent areas	GW05157	Shierzhan	Syenogranite	125.68	51.20	298	2.0	LA-ICP-MS,U-Pb	Zircon	Sui Z M et al.	2009	Acta Petrologica Sinica(ICWEA)
270	Within the MOO	Erguna and adjacent areas	GW05129	Hehuashan	Granodiorite	127.08	50.89	171	2.0	LA-ICP-MS,U-Pb	Zircon	Sui Z M.	2007	Jilin University China: PHd Dissertation
271	Within the MOO	Erguna and adjacent areas	GW05113	Jiweidianzi	Granodioritic长-Monzogranite	126.27	51.82	176	1.0	LA-ICP-MS,U-Pb	Zircon	Sui Z M.	2007	Jilin University China: PHd Dissertation
272	Within the MOO	Erguna and adjacent areas	GW05120	Jiweidianzi	Monzogranite	126.27	51.82	176	2.0	LA-ICP-MS,U-Pb	Zircon	Sui Z M.	2007	Jilin University China: PHd Dissertation
273	Within the MOO	Erguna and adjacent areas	GW05112	Jiweidianzi	Monzogranite	125.79	51.88	190	2.0	LA-ICP-MS,U-Pb	Zircon	Sui Z M.	2007	Jilin University China: PHd Dissertation
274	Within the MOO	Erguna and adjacent areas	GW05085	Xinghua-Fanshengtun	Monzogranite	126.21	52.01	183	4.0	LA-ICP-MS,U-Pb	Zircon	Sui Z M.	2007	Jilin University China: PHd Dissertation
275	Within the MOO	Erguna and adjacent areas	GW05099	Hanjiayuanzi	Hb. quartz diorite	125.64	52.05	188	1.0	LA-ICP-MS,U-Pb	Zircon	Sui Z M.	2007	Jilin University: Phd Dissertation
276	Within the MOO	Erguna and adjacent areas	GW05099	Hanjiayuanzi	Diorite	125.64	52.05	188	2.0	LA-ICP-MS,U-Pb	Zircon	Sui Z M.	2007	Jilin University China: PHd Dissertation
277	Within the MOO	Erguna and adjacent areas	GW05073	Huairou	Monzogranite	126.33	52.27	236	1.0	LA-ICP-MS,U-Pb	Zircon	Sui Z M.	2007	Jilin University China: PHd Dissertation
278	Within the MOO	Erguna and adjacent areas	GW05067	Zhengqi	Granodiorite	126.14	52.53	190	1.0	LA-ICP-MS,U-Pb	Zircon	Sui Z M.	2007	Jilin University China: PHd Dissertation
279	Within the MOO	Erguna and adjacent areas	13ER14-1 (12ER32)	Mangui–Qiqian highway	Monzogranite	121.89	52.09	185	2.0	LA-ICP-MS,U-Pb	Zircon	Sun C Y., et l.	2017	Science China Earth Sciences
280	Within the MOO	Erguna and adjacent areas	12ER31-1	Northern Mangui	Syenogranite	122.54	52.46	173	2.0	LA-ICP-MS,U-Pb	Zircon	Sun C Y., et l.	2017	Science China Earth Sciences
281	Within the MOO	Erguna and adjacent areas	14ER14-1	Southern Amuer	Monzogranite	123.19	52.73	177	1.0	LA-ICP-MS,U-Pb	Zircon	Sun C Y., et l.	2017	Science China Earth Sciences
282	Within the MOO	Erguna and adjacent areas	18ER12-1	Southern Badaguan	Granitic gneiss	118.90	49.96	206	1.0	LA-ICP-MS,U-Pb	Zircon	Sun C Y., et l.	2019	Scientific Reports
283	Within the MOO	Erguna and adjacent areas	13TH1-1	Baiyinna	Monzogranite	125.84	52.47	196	1.0	LA-ICP-MS,U-Pb	Zircon	Sun C Y., et l.	2019	Scientific Reports
284	Within the MOO	Erguna and adjacent areas	14ER18-1	Mohe	Syenogranite	122.33	52.89	205	1.0	LA-ICP-MS,U-Pb	Zircon	Sun C Y., et l.	2019	Scientific Reports
285	Within the MOO	Erguna and adjacent areas	18ER19-1	Mohe	Syenogranite	122.11	53.10	211	1.0	LA-ICP-MS,U-Pb	Zircon	Sun C Y., et l.	2019	Scientific Reports
286	Within the MOO	Erguna and adjacent areas	1669-1	Qiqian	granodiorite	120.87	52.18	207	5.0	LA-ICP-MS,U-Pb	Zircon	Sun D Y., et al.	2013	International Geology Review
287	Within the MOO	Erguna and adjacent areas	1624-1	Manguixi	monzonite	121.50	52.23	156	2.0	LA-ICP-MS,U-Pb	Zircon	Sun D Y., et al.	2013	International Geology Review
288	Within the MOO	Erguna and adjacent areas	1666-1	Qiqian	granodiorite	120.99	52.31	210	2.0	LA-ICP-MS,U-Pb	Zircon	Sun D Y., et al.	2013	International Geology Review
289	Within the MOO	Erguna and adjacent areas	1608-1	Mangui	monzogranite	122.43	52.32	188	3.0	LA-ICP-MS,U-Pb	Zircon	Sun D Y., et al.	2013	International Geology Review
290	Within the MOO	Erguna and adjacent areas	1665-1	Qiqian	diorite	121.02	52.35	213	2.0	LA-ICP-MS,U-Pb	Zircon	Sun D Y., et al.	2013	International Geology Review
291	Within the MOO	Erguna and adjacent areas	1629-1	Manguixi	monzogranite	121.33	52.42	193	2.0	LA-ICP-MS,U-Pb	Zircon	Sun D Y., et al.	2013	International Geology Review
292	Within the MOO	Erguna and adjacent areas	1630-2	Manguixi	syenogranite	121.24	52.45	207	4.0	LA-ICP-MS,U-Pb	Zircon	Sun D Y., et al.	2013	International Geology Review
293	Within the MOO	Erguna and adjacent areas	1662-1	Qiqian	monzogranite	121.04	52.49	210	2.0	LA-ICP-MS,U-Pb	Zircon	Sun D Y., et al.	2013	International Geology Review
294	Within the MOO	Erguna and adjacent areas	1654-2	Yimuhe	monzogranite	120.29	52.75	250	3.0	LA-ICP-MS,U-Pb	Zircon	Sun D Y., et al.	2013	International Geology Review
295	Within the MOO	Erguna and adjacent areas	1588-1	Fukeshan	monzogranite	121.99	52.76	187	3.0	LA-ICP-MS,U-Pb	Zircon	Sun D Y., et al.	2013	International Geology Review
296	Within the MOO	Erguna and adjacent areas	1657-1	Yimu river	monzogranitic gneiss	120.08	52.77	253	3.0	LA-ICP-MS,U-Pb	Zircon	Sun D Y., et al.	2013	International Geology Review
297	Within the MOO	Erguna and adjacent areas	1605-1	Mohe	granodiorite	122.60	52.82	187	3.0	LA-ICP-MS,U-Pb	Zircon	Sun D Y., et al.	2013	International Geology Review
298	Within the MOO	Erguna and adjacent areas	1583-1	Mohe	monzogranite	122.33	52.89	199	2.0	LA-ICP-MS,U-Pb	Zircon	Sun D Y., et al.	2013	International Geology Review

299	Within the MOO	Erguna and adjacent areas	1637-1	Badaoka	monzogranite	121.11	52.91	248	4.0	LA-ICP-MS,U-Pb	Zircon	Sun D Y., et al.	2013	International Geology Review
300	Within the MOO	Erguna and adjacent areas	M548-4	Jiawula	diorite	116.24	48.69	189	3.0	LA-ICP-MS,U-Pb	Zircon	Sun Deyou et al.	2013	International Geology Review
301	Within the MOO	Erguna and adjacent areas	M232-1	Erguna	Rhyolite	121.00	52.00	214	2.0	LA-ICP-MS,U-Pb	Zircon	Sun Deyou et al.	2013	International Geology Review
302	Within the MOO	Erguna and adjacent areas	M261-1	Erguna	Rhyolite	121.00	52.00	179	2.0	LA-ICP-MS,U-Pb	Zircon	Sun Deyou et al.	2013	International Geology Review
303	Within the MOO	Erguna and adjacent areas	12ER11-1	Erguna Massif	Granite	120.17	51.25	247		LA-ICP-MS,U-Pb	Zircon	Tang J., et al.	2014	Lithos
304	Within the MOO	Erguna and adjacent areas	12ER11-2	Erguna Massif	Granite	120.17	51.25	247		LA-ICP-MS,U-Pb	Zircon	Tang J., et al.	2014	Lithos
305	Within the MOO	Erguna and adjacent areas	ER84003	Erentaolegai	Quartz Monzonite	116.55	48.41	294		LA-ICP-MS,U-Pb	Zircon	Tang J et al.	2014	Geology In China (ICWEA)
306	Within the MOO	Erguna and adjacent areas	ER84011	Erentaolegai	Bi. syenogranite	116.55	48.41	287		LA-ICP-MS,U-Pb	Zircon	Tang J et al.	2014	Geology In China (ICWEA)
307	Within the MOO	Erguna and adjacent areas	ER10401	Erentaolegai	Quartz porphyry	116.55	48.41	285		LA-ICP-MS,U-Pb	Zircon	Tang J et al.	2014	Geology In China (ICWEA)
308	Within the MOO	Erguna and adjacent areas	ER84007	Erentaolegai	Granite	116.55	48.41	244		LA-ICP-MS,U-Pb	Zircon	Tang J et al.	2014	Geology In China (ICWEA)
309	Within the MOO	Erguna and adjacent areas	11ER16-1	Erguna Massif	granodiorite	119.97	51.05	246	1.0	LA-ICP-MS,U-Pb	Zircon	Tang J et al.	2014	Lithos
310	Within the MOO	Erguna and adjacent areas	11ER17-1 (12ER10-1	Enhe–Shiwei highway	Syenogranite	120.04	51.05	242	3.0	LA-ICP-MS,U-Pb	Zircon	Tang J et al.	2014	Lithos
311	Within the MOO	Erguna and adjacent areas	11ER16-3		Granite	119.83	51.17	246		LA-ICP-MS,U-Pb	Zircon	Tang J et al.	2014	Lithos
312	Within the MOO	Erguna and adjacent areas	11ER18-1 (12ER11-1	Enhe–Shiwei highway	Monzogranite	120.03	51.19	247	2.0	LA-ICP-MS,U-Pb	Zircon	Tang J et al.	2014	Lithos
313	Within the MOO	Erguna and adjacent areas	11ER28-1	Erguna Massif	quartz–diorite	120.33	51.33	242	3.0	LA-ICP-MS,U-Pb	Zircon	Tang J et al.	2014	Lithos
314	Within the MOO	Erguna and adjacent areas	12ER18	Erguna Massif	diorite	120.45	51.37	244	3.0	LA-ICP-MS,U-Pb	Zircon	Tang J et al.	2014	Lithos
315	Within the MOO	Erguna and adjacent areas	12ER1-2	Badaguan	Syenogranite	118.83	49.94	155	3.0	LA-ICP-MS,U-Pb	Zircon	Tang J et al.	2015	Tectonophysics
316	Within the MOO	Erguna and adjacent areas	12ER1	Erguna Massif	Syenogranite	118.83	49.94	155		LA-ICP-MS,U-Pb	Zircon	Tang J et al.	2015	Tectonophysics
317	Within the MOO	Erguna and adjacent areas	12ER2-(1–4, 7–8)	Erguna Massif	Syenogranite	118.83	49.94	152		LA-ICP-MS,U-Pb	Zircon	Tang J et al.	2015	Tectonophysics
318	Within the MOO	Erguna and adjacent areas	12ER2-1	Southwestern Badaguan	Syenogranite	118.83	49.94	152	2.0	LA-ICP-MS,U-Pb	Zircon	Tang J et al.	2015	Tectonophysics
319	Within the MOO	Erguna and adjacent areas	13ER48-1	Badaguan	Syenogranite	118.84	49.94	150	2.0	LA-ICP-MS,U-Pb	Zircon	Tang J et al.	2015	Tectonophysics
320	Within the MOO	Erguna and adjacent areas	12ER12	Erguna Massif	Granodiorite	119.68	50.98	246		LA-ICP-MS,U-Pb	Zircon	Tang J et al.	2015	Tectonophysics
321	Within the MOO	Erguna and adjacent areas	11ER21	Weshi	Monzonite	119.82	51.22	155		LA-ICP-MS,U-Pb	Zircon	Tang J et al.	2015	Tectonophysics
322	Within the MOO	Erguna and adjacent areas	11ER22	Weshi	Monzonite	119.82	51.23	155		LA-ICP-MS,U-Pb	Zircon	Tang J et al.	2015	Tectonophysics
323	Within the MOO	Erguna and adjacent areas	13ER16	Erguna Massif	Quartz monzonite	121.50	52.16	155		LA-ICP-MS,U-Pb	Zircon	Tang J et al.	2015	Tectonophysics
324	Within the MOO	Erguna and adjacent areas	12ER35	Erguna Massif	Quartz monzonite	121.56	52.22	156		LA-ICP-MS,U-Pb	Zircon	Tang J et al.	2015	Tectonophysics
325	Within the MOO	Erguna and adjacent areas	13ER46-4	Badaguan Cu-Mo deposit	Granodiorite	119.14	49.98	228		LA-ICP-MS,U-Pb	Zircon	Tang J et al.	2016	Gondwana Research
326	Within the MOO	Erguna and adjacent areas	13ER46-1	Badaguan Cu-Mo deposit	Granodiorite	119.14	49.98	228	2.0	LA-ICP-MS,U-Pb	Zircon	Tang J et al.	2016	Gondwana Research
327	Within the MOO	Erguna and adjacent areas	11ER9-1 (13ER45-1)	Enhe-Manzhouli highway	Syenogranite	119.57	50.71	224	2.0	LA-ICP-MS,U-Pb	Zircon	Tang J et al.	2016	Gondwana Research
328	Within the MOO	Erguna and adjacent areas	11ER10-1	Qika	Monzogranite	119.53	50.74	235	5.0	LA-ICP-MS,U-Pb	Zircon	Tang J et al.	2016	Gondwana Research
329	Within the MOO	Erguna and adjacent areas	12ER9-1 (11ER10-1)	Qika	Granodiorite	119.53	50.74	202	2.0	LA-ICP-MS,U-Pb	Zircon	Tang J et al.	2016	Gondwana Research
330	Within the MOO	Erguna and adjacent areas	11ER13-1	Qika–Baka highway	Monzogranite	119.53	50.77	203	3.0	SIMS,U-Pb	Zircon	Tang J et al.	2016	Gondwana Research
331	Within the MOO	Erguna and adjacent areas	13ER21-1	Moerdaoga	Granodiorite	120.97	51.31	195	1.0	LA-ICP-MS,U-Pb	Zircon	Tang J et al.	2016	Gondwana Research
332	Within the MOO	Erguna and adjacent areas	12ER17-1	Moerdaoga	Monzogranite	120.43	51.31	205	2.0	LA-ICP-MS,U-Pb	Zircon	Tang J et al.	2016	Gondwana Research
333	Within the MOO	Erguna and adjacent areas	12ER16-3 (11ER27-1	Moerdaoga	Monzogranite	120.37	51.32	206	2.0	LA-ICP-MS,U-Pb	Zircon	Tang J et al.	2016	Gondwana Research
334	Within the MOO	Erguna and adjacent areas	13ER6-1 (12ER23-1)	Jinhe	Syenogranite	121.50	51.34	196	1.0	LA-ICP-MS,U-Pb	Zircon	Tang J et al.	2016	Gondwana Research
335	Within the MOO	Erguna and adjacent areas	11ER26-1 (12ER15-1	Moerdaoga	Monzogranite	120.31	51.35	206	2.0	LA-ICP-MS,U-Pb	Zircon	Tang J et al.	2016	Gondwana Research
336	Within the MOO	Erguna and adjacent areas	12ER19-1	Moerdaoga	Syenogranite	120.81	51.49	205	1.0	LA-ICP-MS,U-Pb	Zircon	Tang J et al.	2016	Gondwana Research
337	Within the MOO	Erguna and adjacent areas	12ER27-1	Genhe–Mangui highway	Granodiorite	121.76	51.58	197	2.0	LA-ICP-MS,U-Pb	Zircon	Tang J et al.	2016	Gondwana Research
338	Within the MOO	Erguna and adjacent areas	13ER8-1	Alongshan	Gabbro	121.54	51.67	204	1.0	LA-ICP-MS,U-Pb	Zircon	Tang J et al.	2016	Gondwana Research
339	Within the MOO	Erguna and adjacent areas	13ER8-6	Alongshan	Gabbro–diorite	121.54	51.67	204	2.0	LA-ICP-MS,U-Pb	Zircon	Tang J et al.	2016	Gondwana Research
340	Within the MOO	Erguna and adjacent areas	13ER31-6	Mangui	Granodiorite	120.93	51.86	227	6.0	SIMS,U-Pb	Zircon	Tang J et al.	2016	Gondwana Research
341	Within the MOO	Erguna and adjacent areas	13ER31-1	Xiniuerhe	Granodiorite	120.93	51.86	246	1.0	LA-ICP-MS,U-Pb	Zircon	Tang J et al.	2016	Gondwana Research
342	Within the MOO	Erguna and adjacent areas	13ER34-1	Qiqian	Gabbro	120.86	52.04	205	1.0	LA-ICP-MS,U-Pb	Zircon	Tang J et al.	2016	Gondwana Research
343	Within the MOO	Erguna and adjacent areas	13ER13-6	Erguna Massif	Gabbro–diorite	122.09	52.06	227		SIMS,U-Pb	Zircon	Tang J et al.	2016	Gondwana Research
344	Within the MOO	Erguna and adjacent areas	12ER34-1	Mangui	Syenogranite	121.65	52.21	206	1.0	LA-ICP-MS,U-Pb	Zircon	Tang J et al.	2016	Gondwana Research
345	Within the MOO	Erguna and adjacent areas	12ER30-5	Erguna Massif	Gabbro–diorite	122.57	52.42	200	1.0	LA-ICP-MS,U-Pb	Zircon	Tang J et al.	2016	Gondwana Research
346	Within the MOO	Erguna and adjacent areas	ER15-1 (11ER5-1)		Granodiorite	120.09	50.79	241	1.0	LA-ICP-MS,U-Pb	Zircon	Tang J et al.	2016	Gondwana Research
347	Within the MOO	Erguna and adjacent areas	M095-4	Arhashate Basin	Alkali-feldspar granite	116.03	48.55	208	2.0	LA-ICP-MS,U-Pb	Zircon	Wang Tianhao et al.	2014	Global Geology(ICWEA)
348	Within the MOO	Erguna and adjacent areas	M141-1	Dashimo Basin	Monzogranite	117.06	49.44	179	1.0	LA-ICP-MS,U-Pb	Zircon	Wang Tianhao et al.	2014	Global Geology(ICWEA)
349	Within the MOO	Erguna and adjacent areas	M114-1	Wunugetu	Granite	11.30	49.42	180	1.0	LA-ICP-MS,U-Pb	Zircon	Wang Tianhao et al.	2014	Global Geology(ICWEA)
350	Within the MOO	Erguna and adjacent areas	M114-2	Wunugetu	Granite	11.30	49.42	179	1.0	LA-ICP-MS,U-Pb	Zircon	Wang Tianhao et al.	2014	Global Geology(ICWEA)
351	Within the MOO	Erguna and adjacent areas	11ER1-1 (ER6-1)	Xiahulin	Alkali feldspar granite	120.16	50.64	186	3.0	LA-ICP-MS,U-Pb	Zircon	Wang W. et al.	2012	Geological Journal of China Universities(ICWEA)
352	Within the MOO	Erguna and adjacent areas	ER12-1 (ER22-1)		Syenogranite	120.03	50.86	229	4.0	LA-ICP-MS,U-Pb	Zircon	Wang W. et al.	2012	Geological Journal of China Universities(ICWEA)
353	Within the MOO	Erguna and adjacent areas	DB-03	Baogedewula	Granite porphyry	116.75	48.12	153	1.0	LA-ICP-MS,U-Pb	Zircon	Wang W. et al.	2012	Geological Journal of China Universities(ICWEA)
354	Within the MOO	Erguna and adjacent areas	MZ18-2 (12MZ2)	Wunugetu mountain	Monzogranite	117.32	49.41	180	2.0	LA-ICP-MS,U-Pb	Zircon	Wang W. et al.	2012	Geological Journal of China Universities(ICWEA)
355	Within the MOO	Erguna and adjacent areas	MZ17-1	Lingquan Basin	Mineralized quartz porphyry	117.30	49.42	183	2.0	LA-ICP-MS,U-Pb	Zircon	Wang W. et al.	2012	Geological Journal of China Universities(ICWEA)
356	Within the MOO	Erguna and adjacent areas	MZ23-1	Chalataolegai mountain	Monzogranite	117.01	49.47	171	2.0	LA-ICP-MS,U-Pb	Zircon	Wang W. et al.	2012	Geological Journal of China Universities(ICWEA)
357	Within the MOO	Erguna and adjacent areas	ER6-1	Shanghulin	Syenogranite	120.16	50.64	186	3.0	LA-ICP-MS,U-Pb	Zircon	Wang W. et al.	2012	Geological Journal Of China Universities (ICWEA)
358	Within the MOO	Erguna and adjacent areas	R6-1	Shanghulin	Syenogranite	120.16	50.64	186	3.0	LA-ICP-MS,U-Pb	Zircon	Wang W. et al.	2012	Geological Journal of China Universities(in Chinese)
359	Within the MOO	Erguna and adjacent areas	ER15-1	Shanghulin	Syenogranite	120.09	50.79	241	1.0	LA-ICP-MS,U-Pb	Zircon	Wang W. et al.	2012	Geological Journal Of China Universities (ICWEA)
360	Within the MOO	Erguna and adjacent areas	ZKS1-1	Enhe	Monzogranite	120.08	50.81	185	1.0	LA-ICP-MS,U-Pb	Zircon	Wang W. et al.	2012	Geological Journal of China Universities(ICWEA)
361	Within the MOO	Erguna and adjacent areas	ER12-1	Shanghulin	Syenogranite	120.03	50.86	229	4.0	LA-ICP-MS,U-Pb	Zircon	Wang W. et al.	2012	Geological Journal Of China Universities (ICWEA)
362	Within the MOO	Erguna and adjacent areas	W-9		Monzogranite porphyry	120.02	49.38	180		LA-ICP-MS,U-Pb	Zircon	Wang Y., et al.	2015	Gondwana Research
363	Within the MOO	Erguna and adjacent areas	YL4	Yili	monzogranite	124.28	49.46	350	2.3	SHRIMP,U-Pb	Zircon	Wu C. et al.	2014	International Geology Review
364	Within the MOO	Erguna and adjacent areas	9806—3	Guguhe	Syenogranite	125.41	49.98	264	5.0	TIMS,U-Pb	Zircon	Wu Fuyuan et al.	2002	Chemical Geology
365	Within the MOO	Erguna and adjacent areas	9843—1	Daheishan	Syenogranite	126.47	50.23	292	4.0	TIMS,U-Pb	Zircon	Wu Fuyuan et al.	2002	Chemical Geology
366	Within the MOO	Erguna and adjacent areas	2.88834e+006	Huaduoshan	Granodiorite	125.73	50.38	170		LA-ICP-MS,U-Pb	Zircon	Wu Fuyuan et al.	2003	Lithos
367	Within the MOO	Erguna and adjacent areas	PM15TC92		Monzogranite	126.44	50.21	176	2.0	LA-ICP-MS,U-Pb	Zircon	Wu J W et al.	2012	Master Thesis: China University of Geosciences, Beijing
368	Within the MOO	Erguna and adjacent areas	PM8LT5	Abaote-Chaganchulute	Granodiorite	124.85	50.38	159	0.9	LA-ICP-MS,U-Pb	Zircon	Wu J W et al.	2012	Master Thesis: China University of Geosciences, Beijing
369	Within the MOO	Erguna and adjacent areas	R003	Wuliquanbei	Monzogranite	119.89	47.24	240	4.0	LA-ICP-MS,U-Pb	Zircon	Xie H et al.	2011	Journal of Jilin University（Earth Science Edition）(ICWEA)
370	Within the MOO	Erguna and adjacent areas	1312	Huolongmeng	Monzogranite	125.86	49.79	175	2.0	SHRIMP,U-Pb	Zircon	Xu W X et al.	2018	Geology and Resources(ICWEA)
371	Within the MOO	Erguna and adjacent areas	13GW348	Xing'an Block	Syenogranite	120.82	48.07	244		LA-ICP-MS,U-Pb	Zircon	Yang H., et al.	2016	Journal of Asian Earth Sciences
372	Within the MOO	Erguna and adjacent areas	PM001-6-1	Taerqi	Monzogranite	121.26	48.00	167	1.0	LA-ICP-MS,U-Pb	Zircon	Yuan X Y	2015	Master Thesis: Jilin University
373	Within the MOO	Erguna and adjacent areas	PM001-10-1	Taerqi	Syenogranite	121.27	48.01	169	2.0	LA-ICP-MS,U-Pb	Zircon	Yuan X Y	2015	Master Thesis: Jilin University
374	Within the MOO	Erguna and adjacent areas	PM001-27-1	Taerqi	Bi. Monzogranite	121.34	48.03	166	1.0	LA-ICP-MS,U-Pb	Zircon	Yuan X Y	2015	Master Thesis: Jilin University

375	Within the MOO	Erguna and adjacent areas	XK10-1	Datushan	Hb.Bi, Quartz diorite	127.12	50.18	187	1.0	LA-ICP-MS,U-Pb	Zircon	Zeng T., et al.	2011	Journal of Jilin University（Earth Science Edition）(ICWEA)
376	Within the MOO	Erguna and adjacent areas	xk10-11	Dapingshan	Twomica Monzogranite	126.75	50.28	171	1.0	LA-ICP-MS,U-Pb	Zircon	Zeng T., et al.	2011	Journal of Jilin University（Earth Science Edition）(ICWEA)
377	Within the MOO	Erguna and adjacent areas	W-8		Granite porphyry	117.25	49.41	201		SHRIMP,U-Pb	Zircon	Zhang F F., et al.	2016	Journal of Asian Earth Sciences
378	Within the MOO	Erguna and adjacent areas	1210	Duobaoshan	Monzodiorite	125.80	50.23	292	7.0	SHRIMP,U-Pb	Zircon	Zhang L D et al.	2011	Acta Mineralogica Sinica(ICWEA)
379	Within the MOO	Erguna and adjacent areas	D490	Boluonuo	Hb. Gabbro	117.34	50.07	297	1.0	LA-ICP-MS,U-Pb	Zircon	Zhang S H et al.	2009	International Journal of Earth Sciences
380	Within the MOO	Erguna and adjacent areas	Lzk1-285	Luohe	Tonalite	125.87	50.25	244		LA-ICP-MS,U-Pb	Zircon	Zhao Chao et al.	2019	Lithos
381	Within the MOO	Erguna and adjacent areas	YJD03	Yuejin	Granodiorite	124.69	50.27	242	1.8	LA-ICP-MS,U-Pb	Zircon	Zhao Chao et al.	2019	Lithos
382	Within the MOO	Erguna and adjacent areas	MT3-1	Mantoushan	Granodiorite	125.85	50.27	240		LA-ICP-MS,U-Pb	Zircon	Zhao Chao et al.	2019	Lithos
383	Within the MOO	Erguna and adjacent areas	Lzk501-290.5	Luohe	Granodiorite	125.89	50.27	225	2.0	LA-ICP-MS,U-Pb	Zircon	Zhao Chao et al.	2019	Lithos
384	Within the MOO	Erguna and adjacent areas	14DB18-1	Duobaoshan	Tonalite	124.77	50.24	223	2.6	LA-ICP-MS,U-Pb	Zircon	Zhao Chao et al.	2019	Lithos
385	Within the MOO	Erguna and adjacent areas	TC53	Xinkailin	Granodiorite	126.79	50.27	171	21.0	LA-ICP-MS,U-Pb	Zircon	Zhao H B et al.	2007	Geological Science(ICWEA)
386	Within the MOO	Erguna and adjacent areas	TC78	Xinkailin	Monzogranite	126.79	50.27	147	2.0	LA-ICP-MS,U-Pb	Zircon	Zhao H B et al.	2007	Geological Science(ICWEA)
387	Within the MOO	Erguna and adjacent areas	PM014LT1		Alkali-feldspar granite	125.29	49.61	299	1.1	LA-ICP-MS,U-Pb	Zircon	Zhao H L et al.	2011	Global Geology(ICWEA)
388	Within the MOO	Erguna and adjacent areas	D1285	Hantaqi-Xinkailing	Monzogranitic mylonite	126.85	50.02	345	2.0	TIMS,U-Pb	Zircon	Zhao H L et al.	2011	Global Geology(ICWEA)
389	Within the MOO	Erguna and adjacent areas	D0994	Hantaqi-Xinkailing	Alkali-feldspar granite	125.56	50.03	304	2.0	SHRIMP,U-Pb	Zircon	Zhao H L et al.	2011	Global Geology(ICWEA)
390	Within the MOO	Erguna and adjacent areas	PM010B43	Hantaqi-Xinkailing	Monzogranitic mylonite	126.39	50.04	310	3.0	SHRIMP,U-Pb	Zircon	Zhao H L et al.	2011	Global Geology(ICWEA)
391	Within the MOO	Erguna and adjacent areas	D0991	Hantaqi-Xinkailing	Alkali-feldspar granite	125.43	50.07	300	3.0	SHRIMP,U-Pb	Zircon	Zhao H L et al.	2011	Global Geology(ICWEA)
392	Within the MOO	Erguna and adjacent areas	D2528d	Hantaqi-Xinkailing	Syenite	126.21	50.30	310	3.0	TIMS,U-Pb	Zircon	Zhao H L et al.	2011	Global Geology(ICWEA)
393	Within the MOO	Erguna and adjacent areas	D2528		Granodiorite	126.73	50.30	345	1.7	TIMS,U-Pb	Zircon	Zhao H L et al.	2011	Global Geology(ICWEA)
394	Within the MOO	SE. Sino-Mongol border	SZK301-TW1	Suonaga	Bi. Granite	117.89	46.28	173	1.4	LA-ICP-MS,U-Pb	Zircon	Liang Y W et al.	2013	Geology in China(ICWEA)
395	Within the MOO	SE. Sino-Mongol border	SZK301-TW2	Suonaga	Porphyritic granite	117.89	46.28	166	1.8	LA-ICP-MS,U-Pb	Zircon	Liang Y W et al.	2013	Geology in China(ICWEA)
396	Within the MOO	SE. Sino-Mongol border	S-11	Bayanshuhetu	Monzogranite	115.00	45.50	296	3.5	LA-ICP-MS,U-Pb	Zircon	Shen Xiaoli et al.	2013	Journal of Jilin University（Earth Science Edition）(ICWEA)
397	Within the MOO	SE. Sino-Mongol border		Wulandele	Granodiorite	112.74	44.80	299	2.4	SHRIMP,U-Pb	Zircon	Tao J X et al.	2009	Rock and Mineral Analysis(ICWEA)
398	Within the MOO	SE. Sino-Mongol border	N16802-2-9		Monzogranite	112.66	44.81	166	2.0	LA-ICP-MS,U-Pb	Zircon	This study		
399	Within the MOO	SE. Sino-Mongol border	M13718-3.1		Twomica monzogranite	115.08	45.80	180	2.0	LA-ICP-MS,U-Pb	Zircon	This study		
400	Within the MOO	SE. Sino-Mongol border	N17426-2-1		Monzogranite	116.83	45.82	163	1.0	LA-ICP-MS,U-Pb	Zircon	This study		
401	Within the MOO	SE. Sino-Mongol border	N17422-4-1		Monzogranite	119.20	46.56	229	2.0	LA-ICP-MS,U-Pb	Zircon	This study		
402	Within the MOO	SE. Sino-Mongol border	N17422-3-1		Granite	119.13	46.56	233	1.0	LA-ICP-MS,U-Pb	Zircon	This study		
403	Within the MOO	SE. Sino-Mongol border	N17423-2-1		Monzogranite	117.84	46.11	304	1.0	LA-ICP-MS,U-Pb	Zircon	This study		
404	Within the MOO	SE. Sino-Mongol border	M13717-5.1		K-spar granite	115.67	45.82	301	1.0	LA-ICP-MS,U-Pb	Zircon	This study		
405	Within the MOO	SE. Sino-Mongol border	M13717-9.1		K-spar granite	115.84	45.76	306	1.0	LA-ICP-MS,U-Pb	Zircon	This study		
406	Within the MOO	SE. Sino-Mongol border	M13717-11.1		K-spar granite	115.61	45.67	308	2.0	LA-ICP-MS,U-Pb	Zircon	This study		
407	Within the MOO	SE. Sino-Mongol border	M13717-14.1		Hb. granodiorite	115.54	45.66	318	3.0	LA-ICP-MS,U-Pb	Zircon	This study		
408	Within the MOO	SE. Sino-Mongol border	M16603-6.1		Monzogranite	113.77	45.16	332	4.0	LA-ICP-MS,U-Pb	Zircon	This study		
409	Within the MOO	SE. Sino-Mongol border	M16603-4		Monzogranite	113.80	45.15	300	4.0	LA-ICP-MS,U-Pb	Zircon	This study		
410	Within the MOO	SE. Sino-Mongol border	M16607-23		Granodiorite	112.70	44.99	319	4.0	LA-ICP-MS,U-Pb	Zircon	This study		
411	Within the MOO	SE. Sino-Mongol border	M16607-5.3		Granodiorite	112.70	44.90	350	5.0	LA-ICP-MS,U-Pb	Zircon	This study		
412	Within the MOO	SE. Sino-Mongol border	N16730-1-15		Monzogranite	112.70	44.77	304	3.0	LA-ICP-MS,U-Pb	Zircon	This study		
413	Within the MOO	SE. Sino-Mongol border	N15323-6.1		Granodiorite	112.74	44.76	162	2.0	LA-ICP-MS,U-Pb	Zircon	This study		
414	Within the MOO	SE. Sino-Mongol border	H11-05			112.56	44.75	161	1.0	LA-ICP-MS,U-Pb	Zircon	This study		
415	Within the MOO	SE. Sino-Mongol border	N16730-2-5		Monzogranite	112.66	44.75	300	3.0	LA-ICP-MS,U-Pb	Zircon	This study		
416	Within the MOO	SE. Sino-Mongol border	AHGY-40	Aqinchulu	Monzogranite	117.85	46.11	296	3.8	SHRIMP,U-Pb	Zircon	Wang Z H et al.	2014	Journal of Jilin University (Earth Science Edition)(ICWEA)
417	Within the MOO	SE. Sino-Mongol border	D4506-TW2	Abaote-Chaganchulute	Syenogranite	118.20	46.65	321	2.0	LA-ICP-MS,U-Pb	Zircon	Wu J W et al.	2012	China University of Geosciences，Beijing:Master Dissertation
418	Within the MOO	SE. Sino-Mongol border	D4506-TW1	Abaote-Chaganchulute	Syenogranite	118.20	46.65	325	1.2	LA-ICP-MS,U-Pb	Zircon	Wu J W et al.	2012	China University of Geosciences，Beijing:Master Dissertation
419	Within the MOO	SE. Sino-Mongol border	D4612-TW1	Abaote-Chaganchulute	Syenogranite	118.16	46.63	327	1.3	LA-ICP-MS,U-Pb	Zircon	Wu J W et al.	2012	China University of Geosciences，Beijing:Master Dissertation
420	Within the MOO	SE. Sino-Mongol border	PM201-9TW1	Ashigen-Baolageshan	Syenogranite	118.75	46.36	312	1.8	LA-ICP-MS,U-Pb	Zircon	Wu J W et al.	2012	Master Thesis: China University of Geosciences, Beijing
421	Within the MOO	SE. Sino-Mongol border	D7202-TW1	Ashigen-Baolageshan	Monzogranite	118.32	46.34	289	14.0	LA-ICP-MS,U-Pb	Zircon	Wu J W et al.	2012	China University of Geosciences，Beijing:Master Dissertation
422	Within the MOO	SE. Sino-Mongol border	PM107-6TW1	Ashigen-Baolageshan	Syenogranite	118.26	46.34	312	1.1	LA-ICP-MS,U-Pb	Zircon	Wu J W et al.	2012	Master Thesis: China University of Geosciences, Beijing
423	Within the MOO	SE. Sino-Mongol border	D7201-TW2	Ashigen-Baolageshan	Monzogranite	118.32	46.33	293	8.1	LA-ICP-MS,U-Pb	Zircon	Wu J W et al.	2012	China University of Geosciences，Beijing:Master Dissertation
424	Within the MOO	SE. Sino-Mongol border	D7201-TW1	Ashigen-Baolageshan	Monzogranite	118.32	46.33	306	2.2	LA-ICP-MS,U-Pb	Zircon	Wu J W et al.	2012	China University of Geosciences，Beijing:Master Dissertation
425	Within the MOO	SE. Sino-Mongol border	GS3374	Arenshaobu	Bi. Monzogranite	111.63	44.65	308	2.0	LA-ICP-MS,U-Pb	Zircon	Xu L Q., et al.	2012	Geological Bulletin Of China (ICWEA)
426	Within the MOO	SE. Sino-Mongol border	GS2397	Arenshaobu	Bi. granite	111.59	44.62	317	2.0	LA-ICP-MS,U-Pb	Zircon	Xu L Q., et al.	2012	Geological Bulletin Of China (ICWEA)
427	Within the MOO	SE. Sino-Mongol border	3634—1	Binbalechagan	Syenogranite	119.11	46.51	220	2.5	SHRIMP,U-Pb	Zircon	Yang J Q et al.	2012	Geological Survey and Research(ICWEA)
428	Within the MOO	SE. Sino-Mongol border	E10925-7	Diyajingaoshu	Syenogranite	112.05	44.82	274	2.0	LA-ICP-MS,U-Pb	Zircon	Yang Qidi et al.	2014	Chinese Aacademy of Geological Sciences: PHd Dissertation
429	Within the MOO	SE. Sino-Mongol border	YUM-16/2	Mandakh massif	Alkali granosyenite	108.56	44.50	292	1.0	TIMS,U-Pb	Zircon	Yarmolyuk V V, et al	2008	
430	Within the MOO	SE. Sino-Mongol border	BYTG03	Moruogeqing	Monzodiorite	114.73	45.22	313	4.1	SHRIMP,U-Pb	Zircon	Yun F et al.	2011	Mineral Deposits(ICWEA)
431	Within the MOO	SE. Sino-Mongol border	09NM38	Wulandele	Bi. Monzogranite	112.66	44.75	161	0.7	LA-ICP-MS,U-Pb	Zircon	Zhang K et al.	2013	Global Geology(ICWEA)
432	Within the MOO	SE. Sino-Mongol border	11		Monzogranite	117.83	46.11	299		LA-ICP-MS,U-Pb	Zircon	Zhang W Y	2008	Chinese Academy of Geological Sciences: Phd Dissertation
433	Within the MOO	Trans-Baikal region	Zim 583	Zimovechensky	Alkali nepheline-bearing syenite	111.47	53.68	289	3.60	SHRIMP,U-Pb	Zircon	Izbrodin Ivan et al.	2020	Geoscience Frontiers
434	Within the MOO	Trans-Baikal region	K22A	Koma	Alkali nepheline-bearing syenite	107.49	52.11	314	5.60	SHRIMP,U-Pb	Zircon	Izbrodin Ivan et al.	2020	Geoscience Frontiers
435	Within the MOO	Trans-Baikal region	T-573	Tuchinsky	Alkali nepheline-bearing syenite	111.51	53.82	280	2.60	SHRIMP,U-Pb	Zircon	Izbrodin Ivan et al.	2020	Geoscience Frontiers
436	Within the MOO	Trans-Baikal region		Burpala	syenite	108.69	51.93	283	8.00	TIMS,U-Pb	Zircon	Budnikov et al.	1995	Doklady Akademii Nauk
437	Within the MOO	Trans-Baikal region	3	Burpala	Foliated porphy granodioritesric	108.32	52.81	290	3.00	TIMS,U-Pb	Zircon	Budnikov et al.	1995	Doklady Akademii Nauk - Rossijskaya Akademiya Nauk
438	Within the MOO	Trans-Baikal region	7		Granite	113.38	56.23	278		TIMS,U-Pb	Zircon	Bukharov et al.	1992	Geologiya i Geofizika;(Russian Federation)
439	Within the MOO	Trans-Baikal region	16		Biotite granite	114.11	56.57	285		TIMS,U-Pb	Zircon	Bukharov et al.	1992	Geologiya i Geofizika;(Russian Federation)
440	Within the MOO	Trans-Baikal region	15		Coarse-grained biotite granite	114.14	56.71	313		TIMS,U-Pb	Zircon	Bukharov et al.	1992	Geologiya i Geofizika;(Russian Federation)
441	Within the MOO	Trans-Baikal region	13		Biotite granite	112.66	56.90	301		TIMS,U-Pb	Zircon	Bukharov et al.	1992	Geologiya i Geofizika;(Russian Federation)
442	Within the MOO	Trans-Baikal region	14		Granosyenites	112.92	56.99	325		TIMS,U-Pb	Zircon	Bukharov et al.	1992	Geologiya i Geofizika;(Russian Federation)
443	Within the MOO	Trans-Baikal region	12		Granite	116.39	57.48	272		TIMS,U-Pb	Zircon	Bukharov et al.	1992	Geologiya i Geofizika;(Russian Federation)
444	Within the MOO	Trans-Baikal region	M02/109	Buteel	Foliated granite	105.12	50.13	229	1.2	SHRIMP,U-Pb	Zircon	Donskaya T.V. et al.	2008	Journal of the Geological Society
445	Within the MOO	Trans-Baikal region	NM 1	Buteel	Gneissic granite	105.12	50.13	211	1.2	SHRIMP,U-Pb	Zircon	Donskaya T.V. et al.	2008	Journal of the Geological Society
446	Within the MOO	Trans-Baikal region	M02/107	Buteel	Granodiorite	105.86	50.30	240	2.6	SHRIMP,U-Pb	Zircon	Donskaya T.V. et al.	2008	Journal of the Geological Society
447	Within the MOO	Trans-Baikal region	66	Kataev complex	Rhyolite	106.05	50.32	226	3.0	TIMS,U-Pb	Zircon	Donskaya T.V. et al.	2008	Journal of the Geological Society
448	Within the MOO	Trans-Baikal region	M02/111	Buteel	Felsic volcanic	105.14	50.66	265	1.2	SHRIMP,U-Pb	Zircon	Donskaya T.V. et al.	2008	Journal of the Geological Society
449	Within the MOO	Trans-Baikal region	5153	Khambin	Volcanics	106.31	51.49	226	3.0	SHRIMP,U-Pb	Zircon	Donskaya T.V. et al.	2012	Russian Geology and Geophysics
450	Within the MOO	Trans-Baikal region	6525	Buteel	Granodioritic gneiss	105.12	50.13	223	5.0	SHRIMP,U-Pb	Zircon	Donskaya T.V. et al.	2012	Russian Geology and Geophysics

451	Within the MOO	Trans-Baikal region		Zaza complex	Granite gneiss	113.77	54.10	323	24.0	SHRIMP,U-Pb	Zircon	Doroshkevich A.G. et	2018	Russian Geology and Geophysics
452	Within the MOO	Trans-Baikal region	V-U-435	Verkhnii Uligli	Albitized nepheline syenite	113.75	55.03	249	3.9	LA-ICP-MS,U-Pb	Zircon	Doroshkevich A.G. et	2018	Russian Geology and Geophysics
453	Within the MOO	Trans-Baikal region	V-U-436	Verkhnii Uligli	Nepheline syenite	113.75	55.03	244	4.9	SHRIMP,U-Pb	Zircon	Doroshkevich A.G. et	2018	Russian Geology and Geophysics
454	Within the MOO	Trans-Baikal region	395-1	Tsipa	Nepheline syenite	113.75	55.03	241	2.9	SHRIMP,U-Pb	Zircon	Doroshkevich A.G. et	2018	Russian Geology and Geophysics
455	Within the MOO	Trans-Baikal region	PU-38	Pravyi Uligli	Nepheline syenite	113.58	55.13	249	1.4	LA-ICP-MS,U-Pb	Zircon	Doroshkevich A.G. et	2018	Russian Geology and Geophysics
456	Within the MOO	Trans-Baikal region	Sy-103	Synnyr	Nepheline syenite	113.45	54.78	290	3.5	LA–ICP-MS	Zircon	Izbrodin	2017	Russian Geology and Geophysics
457	Within the MOO	Trans-Baikal region	Chi-504/14		alkali syenites	113.43	54.73	311	1.1	LA–ICP-MS	Zircon	Izbrodin.	2017	Russian Geology and Geophysics
458	Within the MOO	Trans-Baikal region	63	Ermakov deposit	Syenites	110.35	51.42	227	1.9	LA-ICP-MS,U-Pb	Zircon	Izbrodin.	2020	Geoscience Frontiers
459	Within the MOO	Trans-Baikal region	K-22A	Koma	syenite	107.46	52.34	314	6.6	LA-ICP-MS,U-Pb	Zircon	Izbrodin.	2020	Geoscience Frontiers
460	Within the MOO	Trans-Baikal region	T-573	Tuchinsky	syenite	111.64	53.46	280	2.6	LA-ICP-MS,U-Pb	Zircon	Izbrodin.	2020	Geoscience Frontiers
461	Within the MOO	Trans-Baikal region	NM 1	Buteel Range	Granite gneiss	105.36	50.07	211	1.0	Pb-Pb	Zircon	Kelty et al.	2008	Tectonophysics(ICWEA)
462	Within the MOO	Trans-Baikal region	023a-04	Unegetei	leucogranite	108.42	52.08	290	2.8	LA-SF-ICP-MS,U-P	Zircon	Khubanov V.B. et al.	2016	Russian Geology and Geophysics
463	Within the MOO	Trans-Baikal region	Te-01-06	Temen	Porphyritic biotite granite	108.10	52.24	317	2.3	LA-SF-ICP-MS,U-P	Zircon	Khubanov V.B. et al.	2016	Russian Geology and Geophysics
464	Within the MOO	Trans-Baikal region	Khs-55a	Khasurta	syenite	108.37	52.25	280	1.9	LA-SF-ICP-MS,U-P	Zircon	Khubanov V.B. et al.	2016	Russian Geology and Geophysics
465	Within the MOO	Trans-Baikal region	Khs-59a	Khasurta	Amphibole-biotite monzonite	108.34	52.26	283	2.5	LA-SF-ICP-MS,U-P	Zircon	Khubanov V.B. et al.	2016	Russian Geology and Geophysics
466	Within the MOO	Trans-Baikal region	Bu-113-04	Burgasy	Quartz syenite	107.86	52.27	290	3.8	LA-SF-ICP-MS,U-P	Zircon	Khubanov V.B. et al.	2016	Russian Geology and Geophysics
467	Within the MOO	Trans-Baikal region	Gl-10-03	Goltsovyi	Weakly gneissoid biotite granite	107.65	52.29	312	2.2	LA-SF-ICP-MS,U-P	Zircon	Khubanov V.B. et al.	2016	Russian Geology and Geophysics
468	Within the MOO	Trans-Baikal region	PR-103a-04	Burgasy	Quartz monzonite	107.87	52.29	289	2.6	LA-SF-ICP-MS,U-P	Zircon	Khubanov V.B. et al.	2016	Russian Geology and Geophysics
469	Within the MOO	Trans-Baikal region	Be-2-02	Angyr River	granite	107.63	52.35	302	2.2	LA-SF-ICP-MS,U-P	Zircon	Khubanov V.B. et al.	2016	Russian Geology and Geophysics
470	Within the MOO	Trans-Baikal region	Zg-65/1	Zelenaya Griva	Biotite gneiss-granite	107.64	52.40	324	2.1	LA-ICP-MS,U-Pb	Zircon	Khubanov V.B. et al.	2016	Russian Geology and Geophysics
471	Within the MOO	Trans-Baikal region	Khn-62n	Khangintui	Quartz monzonite	108.10	52.43	300	2.1	LA-SF-ICP-MS,U-P	Zircon	Khubanov V.B. et al.	2016	Russian Geology and Geophysics
472	Within the MOO	Trans-Baikal region	Br-03-11	Ulyun River	granite	110.80	54.58	293	1.3	LA-SF-ICP-MS,U-P	Zircon	Khubanov V.B. et al.	2016	Russian Geology and Geophysics
473	Within the MOO	Trans-Baikal region	Zim-583	Zimovechinsky		108.44	51.72	289	3.6	LA-ICP-MS,U-Pb	Zircon	Kotov A.V. et al.	2013	Geology
474	Within the MOO	Trans-Baikal region	25	Zaza complex	Gabbro	108.70	52.40	303	7.3	LA-ICP-MS,U-Pb	Zircon	Kotov A.V. et al.	2013	Geology
475	Within the MOO	Trans-Baikal region	NM 2	Buteel Range	Granodioritic gneiss	105.12	50.23	231	1.0	Pb-Pb	Zircon	Kozakov et al.	2007	Petrology(ICWEA)
476	Within the MOO	Trans-Baikal region	9	Barguzin complex	Granodiorite	109.70	52.87	289	1.0	LA-ICP-MS,U-Pb	Zircon	Kozubova et al	1980	Doklady Akademii Nauk SSSR
477	Within the MOO	Trans-Baikal region	Metaluminous suite	Bryansky volcanoplutonic comp	Metaluminous syeniteand granitetra	108.03	51.64	280	14.0	LA-ICP-MS,U-Pb	Zircon	Litvinovsky et al.	2002	Chemical Geology
478	Within the MOO	Trans-Baikal region		Bryansky volcanoplutonic comp	Alkali-feldspar syeniteMetaluminou	108.03	51.64	279	1.3	LA-ICP-MS,U-Pb	Zircon	Litvinovsky et al.	2002	Chemical Geology
479	Within the MOO	Trans-Baikal region	38	Uda dyke swarm	Trachydacite, trachytes	109.58	52.39	301	4.7	SHRIMP,U-Pb	Zircon	Lykhin et al.	2001	Geology of Ore Deposits
480	Within the MOO	Trans-Baikal region		Ermakov deposit	Leucogranite	110.35	51.42	226	1.0	SHRIMP,U-Pb	Zircon	Lykhin et al.	2010	Geology of Ore Deposits
481	Within the MOO	Trans-Baikal region		Ermakov deposit	Granite	110.35	51.42	224	5.0	SHRIMP,U-Pb	Zircon	Lykhin et al.	2010	Geology of Ore Deposits
482	Within the MOO	Trans-Baikal region		Ermakov deposit	granite	109.70	51.72	332	1.0	SHRIMP,U-Pb	Zircon	Lykhin et al.	2010	Geology of Ore Deposits
483	Within the MOO	Trans-Baikal region		Ermakov deposit	granite	109.70	51.72	325	3.0	SHRIMP,U-Pb	Zircon	Lykhin et al.	2010	Geology of Ore Deposits
484	Within the MOO	Trans-Baikal region		Ermakov deposit	Gabbro	109.70	51.72	316	1.0	SHRIMP,U-Pb	Zircon	Lykhin et al.	2010	Geology of Ore Deposits
485	Within the MOO	Trans-Baikal region		Ermakov deposit	Granite	109.70	51.72	316	8.3	SHRIMP,U-Pb	Zircon	Lykhin et al.	2010	Geology of Ore Deposits
486	Within the MOO	Trans-Baikal region	1		Fine-grained leucogranite	111.66	54.94	272	4.0	TIMS,U-Pb	Zircon	Neimark et al.	1993	Doklady Akademii
487	Within the MOO	Trans-Baikal region	2	Barguzin complex	Pegmatite	111.92	55.06	326	27.0	TIMS,U-Pb	Zircon	Neimark et al.	1993	Doklady Akademii
488	Within the MOO	Trans-Baikal region	21		Diorites	110.53	55.51	301	2.0	TIMS,U-Pb	Zircon	Neimark et al.	1993	Doklady Akademii
489	Within the MOO	Trans-Baikal region	Gl-10-03	Goltsov	Bi. granite	107.28	51.99	313	3.0	LA-ICP-MS,U-Pb	Zircon	Ochir G. et al.	2014	ACTA GEOLOGICA SINICA (English Edition)
490	Within the MOO	Trans-Baikal region	B611	Mangirtui	PA gneissic granite	107.45	50.03	153	0.7	LA-ICP-MS,U-Pb	Zircon	Reichow et al.	2010	Chemical Geology
491	Within the MOO	Trans-Baikal region	A92	Kharitonovo complex	PA syenite	107.49	50.47	229	0.6	LA-ICP-MS,U-Pb	Zircon	Reichow et al.	2010	Chemical Geology
492	Within the MOO	Trans-Baikal region	82	Mangirtui massif	Peralkaline granite	107.52	50.74	152	0.7	LA-ICP-MS,U-Pb	Zircon	Reichow et al.	2010	Chemical Geology
493	Within the MOO	Trans-Baikal region		Kharitonovo volcanoplutonic co	Peralkaline syenite	107.09	51.25	229	0.6	LA-ICP-MS,U-Pb	Zircon	Reichow et al.	2010	Chemical Geology
494	Within the MOO	Trans-Baikal region		Kharitonovo volcanoplutonic co	Syenites, alkaline granite	107.09	51.25	221	1.0	LA-ICP-MS,U-Pb	Zircon	Reichow et al.	2010	Chemical Geology
495	Within the MOO	Trans-Baikal region	B361-20	Ust-Khilok pluton	Syenite	107.34	51.62	279	3.0	LA-ICP-MS,U-Pb	Zircon	Reichow et al.	2010	Chemical Geology
496	Within the MOO	Trans-Baikal region		Bryansky volcanoplutonic comp	Peralkaline syenitenordmarkite	108.03	51.64	277	1.1	LA-ICP-MS,U-Pb	Zircon	Reichow et al.	2010	Chemical Geology
497	Within the MOO	Trans-Baikal region		Bryansky volcanoplutonic comp	Comendite,trachyandesite dykes	108.03	51.64	268	1.0	LA-ICP-MS,U-Pb	Zircon	Reichow et al.	2010	Chemical Geology
498	Within the MOO	Trans-Baikal region	36	Nadein pluton	Syenites, granite	107.47	51.80	267	8.0	LA-ICP-MS,U-Pb	Zircon	Reichow et al.	2010	Chemical Geology
499	Within the MOO	Trans-Baikal region	29	Zaza complex	Syenites, granite	108.61	51.92	289	3.7	LA-ICP-MS,U-Pb	Zircon	Reichow et al.	2010	Chemical Geology
500	Within the MOO	Trans-Baikal region	M501	Khorinsk complex	Syenite	109.87	52.33	281	0.8	LA-ICP-MS,U-Pb	Zircon	Reichow et al.	2010	Chemical Geology
501	Within the MOO	Trans-Baikal region	M350	Khorinsk complex	PA granite	109.87	52.44	274	0.9	LA-ICP-MS,U-Pb	Zircon	Reichow et al.	2010	Chemical Geology
502	Within the MOO	Trans-Baikal region	34	Khorinsk volcanoplutonic comp	Trachyte dykes	109.93	52.47	288	4.0	LA-ICP-MS,U-Pb	Zircon	Reichow et al.	2010	Chemical Geology
503	Within the MOO	Trans-Baikal region		Khorinsk volcanoplutonic comp	Trachyandesite dykes	109.93	52.47	282	5.0	LA-ICP-MS,U-Pb	Zircon	Reichow et al.	2010	Chemical Geology
504	Within the MOO	Trans-Baikal region		Khorinsk volcanoplutonic comp	Peralkaline granite	109.93	52.47	279	0.8	LA-ICP-MS,U-Pb	Zircon	Reichow et al.	2010	Chemical Geology
505	Within the MOO	Trans-Baikal region		Khorinsk volcanoplutonic comp	Alkali-feldspar syenite	109.93	52.47	278	1.2	LA-ICP-MS,U-Pb	Zircon	Reichow et al.	2010	Chemical Geology
506	Within the MOO	Trans-Baikal region		Khorinsk volcanoplutonic comp	Peralkaline granite	109.93	52.47	274	0.9	LA-ICP-MS,U-Pb	Zircon	Reichow et al.	2010	Chemical Geology
507	Within the MOO	Trans-Baikal region		Khorinsk volcanoplutonic comp	Alkaline syenites and granite	109.93	52.47	270	15.0	LA-ICP-MS,U-Pb	Zircon	Reichow et al.	2010	Chemical Geology
508	Within the MOO	Trans-Baikal region	16	Chivirkuy complex	Qtz monzonite	109.69	53.71	298	8.0	LA-ICP-MS,U-Pb	Zircon	Reichow et al.	2010	Chemical Geology
509	Within the MOO	Trans-Baikal region	A516		PA granite pegmatite	108.15	50.41	219		SHRIMP,U-Pb	Zircon	Reichow M K et al.	2010	Chemical Geology
510	Within the MOO	Trans-Baikal region	A92-a		PA syenite	107.02	51.19	229		SHRIMP,U-Pb	Zircon	Reichow M K et al.	2010	Chemical Geology
511	Within the MOO	Trans-Baikal region	871		AFS syenite	106.98	51.21	230		SHRIMP,U-Pb	Zircon	Reichow M K et al.	2010	Chemical Geology
512	Within the MOO	Trans-Baikal region	B343	Ust-Khilok	Syenite	107.29	51.51	279		SHRIMP,U-Pb	Zircon	Reichow M K et al.	2010	Chemical Geology
513	Within the MOO	Trans-Baikal region	B627	Bryansky complex	PA syenite	108.11	51.65	277		SHRIMP,U-Pb	Zircon	Reichow M K et al.	2010	Chemical Geology
514	Within the MOO	Trans-Baikal region		Bryansky complex	AFS syenite	108.14	51.67	279		SHRIMP,U-Pb	Zircon	Reichow M K et al.	2010	Chemical Geology
515	Within the MOO	Trans-Baikal region	M499	Khorinsk	Quartz monzonite (country rock)	109.95	52.36	286		SHRIMP,U-Pb	Zircon	Reichow M K et al.	2010	Chemical Geology
516	Within the MOO	Trans-Baikal region	M347	Khorinsk	AFS syenite	109.70	52.37	278		SHRIMP,U-Pb	Zircon	Reichow M K et al.	2010	Chemical Geology
517	Within the MOO	Trans-Baikal region	Gl-10-03	Goltsovyi	Weakly gneissoid biotite granite	107.65	52.29	313	3.0	SIMS,U-Pb	Zircon	Ripp et al.	2009	Petrology
518	Within the MOO	Trans-Baikal region	8	Barguzin complex	Gneissic Bi. granite	113.39	54.13	290	8.0	TIMS,U-Pb	Zircon	Ruzhentsev et al.	2007	Geology
519	Within the MOO	Trans-Baikal region	BD 2417	Bitu-Dzhida	Rare-metal granite	102.18	51.05	311	10.0	LA-ICP-MS,U-Pb	Zircon	Sal'nikova.	2014	Petrology
520	Within the MOO	Trans-Baikal region	M15817-2.1		Dacite porphyry	104.36	49.36	224	5.0	LA-ICP-MS,U-Pb	Zircon	This study		
521	Within the MOO	Trans-Baikal region	NM 4	Buteel Range	Syenite, unfoliated	105.29	50.10	266	1.0	TIMS,U-Pb	Zircon	Tomurtogoo O et al.	2005	Journal of the Geological Society
522	Within the MOO	Trans-Baikal region	62	Orot deposit	Leucogranite	110.54	51.78	232	1.7	TIMS,U-Pb	Zircon	Tomurtogoo O et al.	2005	Journal of the Geological Society
523	Within the MOO	Trans-Baikal region	30	Bryansky complex	Syenites	108.05	51.56	287		SHRIMP,U-Pb	Zircon	Tsygankov A A et al.	2007	Russian Geology and Geophysics
524	Within the MOO	Trans-Baikal region	5	Barguzin complex	Bi. granite	107.91	52.20	313	3.0	LA-ICP-MS,U-Pb	Zircon	Tsygankov A A et al.	2007	Russian Geology and Geophysics
525	Within the MOO	Trans-Baikal region	Khs-59a	Khasurta	Amphibole-biotite monzonite	108.34	52.26	284	5.7	LA-ICP-MS,U-Pb	Zircon	Tsygankov A A et al.	2007	Russian Geology and Geophysics
526	Within the MOO	Trans-Baikal region	8		Amphibole-biotite monzonites	109.02	52.29	284	5.0	SHRIMP,U-Pb	Zircon	Tsygankov A A et al.	2007	Russian Geology and Geophysics
527	Within the MOO	Trans-Baikal region	10		granite	107.94	52.31	325	3.0	SHRIMP,U-Pb	Zircon	Tsygankov A A et al.	2007	Russian Geology and Geophysics

528	Within the MOO	Trans-Baikal region	9		Porphyric quartz syenites	108.79	52.41	302	4.0	SHRIMP,U-Pb	Zircon	Tsygankov A A et al.	2007	Russian Geology and Geophysics
529	Within the MOO	Trans-Baikal region	Khn-62n	Khangintui	Quartz monzonite	108.10	52.43	302	3.7	LA-ICP-MS,U-Pb	Zircon	Tsygankov A A et al.	2007	Russian Geology and Geophysics
530	Within the MOO	Trans-Baikal region	28	Zaza complex	granite	109.12	52.57	294	1.0	LA-ICP-MS,U-Pb	Zircon	Tsygankov A A et al.	2007	Russian Geology and Geophysics
531	Within the MOO	Trans-Baikal region	13	Vitimkan complex	Qtz monzonite	109.43	52.94	292	1.0	LA-ICP-MS,U-Pb	Zircon	Tsygankov A A et al.	2007	Russian Geology and Geophysics
532	Within the MOO	Trans-Baikal region	11		Quartz monzonites	112.65	53.14	279	2.0	SHRIMP,U-Pb	Zircon	Tsygankov A A et al.	2007	Russian Geology and Geophysics
533	Within the MOO	Trans-Baikal region	14	Vitimkan complex	Granite	113.36	54.67	288	2.0	LA-ICP-MS,U-Pb	Zircon	Tsygankov A A et al.	2007	Russian Geology and Geophysics
534	Within the MOO	Trans-Baikal region	59	Menza massif	Granite	108.02	49.35	206	4.4	SHRIMP,U-Pb	Zircon	Tsygankov A A et al.	2010	Russian Geology and Geophysics
535	Within the MOO	Trans-Baikal region	B626-1	Bryansky complex	AFS syenite	108.06	50.59	279	1.3	SHRIMP,U-Pb	Zircon	Tsygankov A A et al.	2010	Russian Geology and Geophysics
536	Within the MOO	Trans-Baikal region	64	Malo-Kunaley massif	Alkaline syenites	107.94	50.61	233	5.0	SHRIMP,U-Pb	Zircon	Tsygankov A A et al.	2010	Russian Geology and Geophysics
537	Within the MOO	Trans-Baikal region	83	Pokrovka massif	Bt-Amph granite Granosyenite	108.01	50.82	161		SHRIMP,U-Pb	Zircon	Tsygankov A A et al.	2010	Russian Geology and Geophysics
538	Within the MOO	Trans-Baikal region	023a-04	Unegetei	Leucogranite	108.70	51.13	289	3.7	SHRIMP,U-Pb	Zircon	Tsygankov A A et al.	2010	Russian Geology and Geophysics
539	Within the MOO	Trans-Baikal region	78	Tugnuy graben, Ichetuy suite	Trachybasalts, trachydacites, trach	107.58	51.32	158	8.0	SHRIMP,U-Pb	Zircon	Tsygankov A A et al.	2010	Russian Geology and Geophysics
540	Within the MOO	Trans-Baikal region	35	Ust-Khilok pluton	Monzonites, syenites	107.34	51.62	280	18.0	SHRIMP,U-Pb	Zircon	Tsygankov A A et al.	2010	Russian Geology and Geophysics
541	Within the MOO	Trans-Baikal region	PR-113-04		Qtz syenite	107.98	51.97	287		SHRIMP,U-Pb	Zircon	Tsygankov A A et al.	2010	Russian Geology and Geophysics
542	Within the MOO	Trans-Baikal region	BKL-431		Leucogranite	108.53	51.98	303		SHRIMP,U-Pb	Zircon	Tsygankov A A et al.	2010	Russian Geology and Geophysics
543	Within the MOO	Trans-Baikal region	Xc59a-02	Khasurta massif	Monzonite	108.83	52.02	284	5.3	SHRIMP,U-Pb	Zircon	Tsygankov A A et al.	2010	Russian Geology and Geophysics
544	Within the MOO	Trans-Baikal region	023a-04	Unegetei	leucogranite	108.42	52.08	289	2.3	SIMS,U-Pb	Zircon	Tsygankov A A et al.	2010	Russian Geology and Geophysics
545	Within the MOO	Trans-Baikal region	Gl10-03	Barguzin complex	Granite (Bt)	107.45	52.17	313		SHRIMP,U-Pb	Zircon	Tsygankov A A et al.	2010	Russian Geology and Geophysics
546	Within the MOO	Trans-Baikal region	31	Zaza complex	Granite	107.59	52.20	286	1.0	LA-ICP-MS,U-Pb	Zircon	Tsygankov A A et al.	2010	Russian Geology and Geophysics
547	Within the MOO	Trans-Baikal region	M340	Khorinsk complex	PA granite	109.68	52.20	279	0.8	SHRIMP,U-Pb	Zircon	Tsygankov A A et al.	2010	Russian Geology and Geophysics
548	Within the MOO	Trans-Baikal region	050-04	Romanovka massif	Monzonite	112.67	52.20	279	2.4	SHRIMP,U-Pb	Zircon	Tsygankov A A et al.	2010	Russian Geology and Geophysics
549	Within the MOO	Trans-Baikal region	Te-01-06	Temen	Porphyritic biotite granite	108.10	52.24	318	4.0	SIMS,U-Pb	Zircon	Tsygankov A A et al.	2010	Russian Geology and Geophysics
550	Within the MOO	Trans-Baikal region	Bu-113-04	Burgasy	Quartz syenite	107.86	52.27	287	4.1	SIMS,U-Pb	Zircon	Tsygankov A A et al.	2010	Russian Geology and Geophysics
551	Within the MOO	Trans-Baikal region	Zg51/2	Zelenaya Griva massif	calc-alkaline granite	107.48	52.27	325	2.8	SHRIMP,U-Pb	Zircon	Tsygankov A A et al.	2010	Russian Geology and Geophysics
552	Within the MOO	Trans-Baikal region	Te01-06		Porphyritic granite	107.53	52.30	318		SHRIMP,U-Pb	Zircon	Tsygankov A A et al.	2010	Russian Geology and Geophysics
553	Within the MOO	Trans-Baikal region	Bur 305 10	pulaskite	Alkaline granite	108.35	52.32	294	1.0	SIMS,U-Pb	Zircon	Tsygankov A A et al.	2010	Russian Geology and Geophysics
554	Within the MOO	Trans-Baikal region	Or28-02		gabbronorite	107.83	52.40	290		SIMS,U-Pb	Zircon	Tsygankov A A et al.	2010	Russian Geology and Geophysics
555	Within the MOO	Trans-Baikal region	166-05	Transbaikalia	Granite	108.02	52.40	286		SHRIMP,U-Pb	Zircon	Tsygankov A A et al.	2010	Russian Geology and Geophysics
556	Within the MOO	Trans-Baikal region	543/4-04		Leucogranite	108.33	52.40	294		SHRIMP,U-Pb	Zircon	Tsygankov A A et al.	2010	Russian Geology and Geophysics
557	Within the MOO	Trans-Baikal region	Xn62-02	Khangintui	Qtz syenite	108.53	52.40	302	3.7	SHRIMP,U-Pb	Zircon	Tsygankov A A et al.	2010	Russian Geology and Geophysics
558	Within the MOO	Trans-Baikal region	M516-1	Transbaikalia	Syenite porphyry	110.02	52.50	280		SHRIMP,U-Pb	Zircon	Tsygankov A A et al.	2010	Russian Geology and Geophysics
559	Within the MOO	Trans-Baikal region	023a-04	Unegetei	leucogranite	109.65	52.86	289	3.7	SIMS,U-Pb	Zircon	Tsygankov A A et al.	2010	Russian Geology and Geophysics
560	Within the MOO	Trans-Baikal region	7	Barguzin complex	Granodiorite	108.67	52.96	290	3.0	SIMS,U-Pb	Zircon	Tsygankov A A et al.	2010	Russian Geology and Geophysics
561	Within the MOO	Trans-Baikal region	BKL-267		Granodiorite	109.50	53.92	289		SIMS,U-Pb	Zircon	Tsygankov A A et al.	2010	Russian Geology and Geophysics
562	Within the MOO	Trans-Baikal region	74	Zhanchivlan massif	Li-F granite	108.89	49.21	195	0.6	TIMS,U-Pb	Zircon	Yarmolyuk V V, et al	1997	Petrology
563	Within the MOO	Trans-Baikal region	39	Zhirim dyke swarm	Trachydacites	107.62	51.49	303	3.0	TIMS,U-Pb	Zircon	Yarmolyuk V V, et al	1997	Petrology
564	Within the MOO	Trans-Baikal region	23	Ermakov deposit	Leucogranite	109.70	51.72	333	10.0	TIMS,U-Pb	Zircon	Yarmolyuk V V, et al	1997	Petrology
565	Within the MOO	Trans-Baikal region	26		Foliated subalkaline granite	107.57	52.08	286	1.0	TIMS,U-Pb	Zircon	Yarmolyuk V V, et al	1997	Petrology
566	Within the MOO	Trans-Baikal region	4		porphyric granodiorites	109.20	52.73	289	1.0	TIMS,U-Pb	Zircon	Yarmolyuk V V, et al	1997	Petrology
567	Within the MOO	Trans-Baikal region	18		monzonite	109.09	52.89	292	1.0	TIMS,U-Pb	Zircon	Yarmolyuk V V, et al	1997	Petrology
568	Within the MOO	Trans-Baikal region	37	Ganzurin and Armak plutons	Syenites and leucogranite	106.93	51.69	275	4.0	TIMS,U-Pb	Zircon	Yarmolyuk V V, et al	2002	Geotectonics
569	Within the MOO	Trans-Baikal region	32	Zaza complex	Syenites, granite	108.32	52.24	286	1.0	TIMS,U-Pb	Zircon	Yarmolyuk V V, et al	2002	Geotectonics
570	Within the MOO	Trans-Baikal region	51	Selenga complex	Diorites, granodiorites,granite	103.97	49.19	280	14.0	TIMS,U-Pb	Zircon	Yarmolyuk V V, et al	2013	Geology
571	Within the MOO	Trans-Baikal region	68	Erdenetiyn deposit	Porphyries	102.92	49.23	221	14.0	TIMS,U-Pb	Zircon	Yarmolyuk V V, et al	2013	Geology
572	Within the MOO	Trans-Baikal region	VG-7/61	Gal-Shara massif	Alkaline granite	111.65	53.40	213	1.0	ID TIMS,U-Pb	Zircon	Yarmolyuk V V, et al	2019	Doklady Earth Sciences
573	Within the MOO	W./C. Mongolia	DZB-1/1	Zuun-Bogd massif	Alkaline granite	101.80	44.77	279	1.0	ID-TIMS,U-Pb	Zircon	Alexander M.et al.	2015	Journal of Asian Earth Sciences
574	Within the MOO	W./C. Mongolia	BaTs-3/2	Khar-Uzuur massif	Alkaline granite	99.46	45.21	284	1.0	ID-TIMS,U-Pb	Zircon	Alexander M.et al.	2015	Journal of Asian Earth Sciences
575	Within the MOO	W./C. Mongolia		Daltyn-Am stock	Granite	100.04	46.53	253	2.0	TIMS,U-Pb	Zircon	Budnikov et al.	1999	Mongolian Geoscientist
576	Within the MOO	W./C. Mongolia	P15	Khangai Highland	Granitoid	100.04	46.79	247		TIMS,U-Pb	Zircon	Budnikov et al.	1999	Mongolian Geoscientist
577	Within the MOO	W./C. Mongolia	DH-12	Hangayn area (central Mongoli	Granite	102.47	45.45	241	1.5	SHRIMP,U-Pb	Zircon	D. Orolmaa et al.	2008	Russian Geology and Geophysics
578	Within the MOO	W./C. Mongolia	M411	Tuya Valley	Felsic metavolcanic	102.54	45.75	344	3.0	SHRIMP,U-Pb	Zircon	Donskaya T.V. et al.	2008	Journal of the Geological Society
579	Within the MOO	W./C. Mongolia	36864	Guchni Us granite	Granite	102.72	45.42	241	2.0	SHRIMP,U-Pb	Zircon	Hanzl & Aichler	2007	Final report of the International Development Co-operation project of the Czech Republic Czech Geologi-cal Survey, Brno & MPRAM,
580	Within the MOO	W./C. Mongolia	M407	Guchni Us granite	Granite	102.61	45.40	244	3.0	SHRIMP,U-Pb	Zircon	Hrdlickova K et al.	2008	Journal of Geosciences
581	Within the MOO	W./C. Mongolia	H0341	Chandman Khayrkhan	Lucogranites	99.31	45.10	344	2.0	LA_ICP_MS	Zircon	Hrdlič ková et al.	2008	Journal of Geosciences
582	Within the MOO	W./C. Mongolia	A65-07.	Dzaraa Uula	Monzogabbro	96.98	46.58	269	4.1	SHRIMP,U-Pb	Zircon	Izbrodin	2011	Russian Geology and Geophysics
583	Within the MOO	W./C. Mongolia	M99-17		Granodiorite	102.38	45.91	230		LA-ICP-MS,U-Pb	Zircon	Jahn et al.	2004	Journal of Asian Earth Sciences
584	Within the MOO	W./C. Mongolia	M99-16	Nariyn Teel pluton	Granite	101.53	46.08	229	5.6	LA-ICP-MS,U-Pb	Zircon	Jahn et al.	2004	Journal of Asian Earth Sciences
585	Within the MOO	W./C. Mongolia	M99-05		Granite	100.02	46.48	250		LA-ICP-MS,U-Pb	Zircon	Jahn et al.	2004	Journal of Asian Earth Sciences
586	Within the MOO	W./C. Mongolia	M99-13		Microgranite	100.29	46.64	250		LA-ICP-MS,U-Pb	Zircon	Jahn et al.	2004	Journal of Asian Earth Sciences
587	Within the MOO	W./C. Mongolia	M99-09		Granite	99.97	46.82	250		LA-ICP-MS,U-Pb	Zircon	Jahn et al.	2004	Journal of Asian Earth Sciences
588	Within the MOO	W./C. Mongolia	M99-07		Granite	100.05	46.82	250		LA-ICP-MS,U-Pb	Zircon	Jahn et al.	2004	Journal of Asian Earth Sciences
589	Within the MOO	W./C. Mongolia	M99-08		Granite	100.05	46.82	237		LA-ICP-MS,U-Pb	Zircon	Jahn et al.	2004	Journal of Asian Earth Sciences
590	Within the MOO	W./C. Mongolia	KHAN 06/1	Erdene Tsogt	Granitoid	99.97	47.02	246	10.0	TIMS,U-Pb	Zircon	Jahn et al.	2004	Journal of Asian Earth Sciences
591	Within the MOO	W./C. Mongolia	M186	Baga Bogd W	Granite dyke	101.03	44.76	338	3.0	SHRIMP,U-Pb	Zircon	Kelty et al.	2008	Tectonophysics
592	Within the MOO	W./C. Mongolia	5914	Trans-Altai Gobi	Granitoid	99.27	45.09	330	4.0	TIMS,U-Pb	Zircon	Kozakov et al.	2007	Petrology
593	Within the MOO	W./C. Mongolia	5907	Trans-Altai Gobi	Granitoid	99.27	45.09	274	4.0	TIMS,U-Pb	Zircon	Kozakov et al.	2007	Petrology
594	Within the MOO	W./C. Mongolia	SKH-3	Trans-Altai Gobi	Granitoid	99.27	45.09	274	5.0	TIMS,U-Pb	Zircon	Kozakov et al.	2007	Petrology
595	Within the MOO	W./C. Mongolia	M175	Sharlin	Greywacke	103.52	46.86	354	3.0	SHRIMP,U-Pb	Zircon	Kozakov et al.	2009	Stratigraphy and Geological Correlation
596	Within the MOO	W./C. Mongolia	KhAN-4476	Khoit-Tamir Massif	Granodiorite	100.95	47.49	255	1.0	TIMS,U-Pb	Zircon	Kozakov et al.	2002	Contributions to Mineralogy and Petrology
597	Within the MOO	W./C. Mongolia	TsTs-4455	Tsetserleg Massif	Granite	101.32	47.39	255	1.0	TIMS,U-Pb	Zircon	Kozakov I K., et al.	2008	Doklady Earth Sciences
598	Within the MOO	W./C. Mongolia	KhT-2/20	Khantaishir graben	Alkaline granite	97.43	46.00	294	4.0	LA-ICP-MS,U-Pb	Zircon	Kozlovsky et al. Journ	2015	Doklady Earth Sciences
599	Within the MOO	W./C. Mongolia	BKh-13/6	Usgekhiin Massif	Quartz-diorite	101.76	45.33	261	2.0	TIMS,U-Pb	Zircon	Kröner et al. unpublished		
600	Within the MOO	W./C. Mongolia	KhAn-25/2	Bu-Tsagan Massif	Monzodiorite	98.64	45.96	253	2.0	TIMS,U-Pb	Zircon	Kröner et al. unpublished		
601	Within the MOO	W./C. Mongolia	6041	Lower Baidarik Massif	Quartz monzonite	99.29	46.05	242	2.0	TIMS,U-Pb	Zircon	Kröner et al. unpublished		
602	Within the MOO	W./C. Mongolia	O-12 /04 (DH12)		Granite	102.17	45.33	241		LA-ICP-MS,U-Pb	Zircon	Orolmaa et al.	2008	Russian Geology and Geophysics
603	Within the MOO	W./C. Mongolia	P22	Khangai Highland	Granitoid	102.62	46.60	288		LA-ICP-MS,U-Pb	Zircon	Orolmaa et al.	2008	Russian Geology and Geophysics
604	Within the MOO	W./C. Mongolia	44	Khangay complex	Qtz monzonite	99.38	46.14	242	2.0	LA-ICP-MS,U-Pb	Zircon	Sal’nikova.	2014	Petrology

605	Within the MOO	W./C. Mongolia	48	Sharausgol complex	Monzodiorite	98.28	46.20	253	2.0	LA-ICP-MS,U-Pb	Zircon	Sal'nikova.	2014	Petrology
606	Within the MOO	W./C. Mongolia	P4	Khangai Highland	Granitoid	96.84	48.00	288		LA-ICP-MS,U-Pb	Zircon	Sal'nikova.	2014	Petrology
607	Within the MOO	W./C. Mongolia	M14925-6.1		K-spar granite	105.07	45.33	271	1.0	LA-ICP-MS,U-Pb	Zircon	This study		
608	Within the MOO	W./C. Mongolia	M1595-36.1		Alkali-feldspar granite	103.71	46.30	310	1.0	LA-ICP-MS,U-Pb	Zircon	This study		
609	Within the MOO	W./C. Mongolia	M1591-3.4		Mylonitic rhyolite	101.23	46.34	281	2.0	LA-ICP-MS,U-Pb	Zircon	This study		
610	Within the MOO	W./C. Mongolia	M1595-32.2		Monzonite	103.58	46.40	321	1.0	LA-ICP-MS,U-Pb	Zircon	This study		
611	Within the MOO	W./C. Mongolia	M1595-5.1		K-spar granite	104.17	46.43	207	2.0	LA-ICP-MS,U-Pb	Zircon	This study		
612	Within the MOO	W./C. Mongolia	M1591-11.1		Gnessis	101.57	46.45	325	2.0	LA-ICP-MS,U-Pb	Zircon	This study		
613	Within the MOO	W./C. Mongolia	M1596-2.1		Alkali-feldspar granite	104.19	46.52	231	1.0	LA-ICP-MS,U-Pb	Zircon	This study		
614	Within the MOO	W./C. Mongolia	M1596-5.1		Monzogranite	104.23	46.53	231	1.0	LA-ICP-MS,U-Pb	Zircon	This study		
615	Within the MOO	W./C. Mongolia	M1597-2.1		Granite	104.41	46.58	210	1.0	LA-ICP-MS,U-Pb	Zircon	This study		
616	Within the MOO	W./C. Mongolia	M17826-1.1		Granodiorite	104.56	47.23	209	3.0	LA-ICP-MS,U-Pb	Zircon	This study		
617	Within the MOO	W./C. Mongolia	M17824-10.4		Monzogranite	100.79	47.38	227	3.0	LA-ICP-MS,U-Pb	Zircon	This study		
618	Within the MOO	W./C. Mongolia	M15828-35.1		Gnessis	100.37	47.39	234	1.0	LA-ICP-MS,U-Pb	Zircon	This study		
619	Within the MOO	W./C. Mongolia	M15828-34.1		Granodiorite	100.37	47.40	240	2.0	LA-ICP-MS,U-Pb	Zircon	This study		
620	Within the MOO	W./C. Mongolia	M15828-33.1		Gnessis	100.37	47.40	230	1.0	LA-ICP-MS,U-Pb	Zircon	This study		
621	Within the MOO	W./C. Mongolia	M15828-32.1		Granitic mylonite	100.37	47.40	211	1.0	LA-ICP-MS,U-Pb	Zircon	This study		
622	Within the MOO	W./C. Mongolia	M17824-1.1		Monzogranite	100.85	47.40	232	5.0	LA-ICP-MS,U-Pb	Zircon	This study		
623	Within the MOO	W./C. Mongolia	M15828-30.3		Granitic mylonite	100.37	47.41	221	1.0	LA-ICP-MS,U-Pb	Zircon	This study		
624	Within the MOO	W./C. Mongolia	M15828-31.1		Granitic mylonite	100.37	47.41	220	1.0	LA-ICP-MS,U-Pb	Zircon	This study		
625	Within the MOO	W./C. Mongolia	M17824-9.1		Granodiorite	100.83	47.42	243	1.0	LA-ICP-MS,U-Pb	Zircon	This study		
626	Within the MOO	W./C. Mongolia	M17824-8.1		Monzogranite	100.83	47.43	235	2.0	LA-ICP-MS,U-Pb	Zircon	This study		
627	Within the MOO	W./C. Mongolia	M17824-8.6		Monzogranite	100.83	47.43	220	3.0	LA-ICP-MS,U-Pb	Zircon	This study		
628	Within the MOO	W./C. Mongolia	M17824-2.1		Granite	100.87	47.45	225	2.0	LA-ICP-MS,U-Pb	Zircon	This study		
629	Within the MOO	W./C. Mongolia	M15820-8.3		Alkali-feldspar granite	102.70	48.13	261	1.0	LA-ICP-MS,U-Pb	Zircon	This study		
630	Within the MOO	W./C. Mongolia	M15822-38.1		Andesite	101.38	48.33	241	1.0	LA-ICP-MS,U-Pb	Zircon	This study		
631	Within the MOO	W./C. Mongolia	M15822-34.2		Granite	101.40	48.41	230	2.0	LA-ICP-MS,U-Pb	Zircon	This study		
632	Within the MOO	W./C. Mongolia	M15823-41.2		Granitic mylonite	101.21	48.47	240	1.0	LA-ICP-MS,U-Pb	Zircon	This study		
633	Within the MOO	W./C. Mongolia	M15823-10.7		Granitic mylonite	100.96	48.51	248	1.0	LA-ICP-MS,U-Pb	Zircon	This study		
634	Within the MOO	W./C. Mongolia	M15821-7.1		Granitic mylonite	101.80	48.54	230	1.0	LA-ICP-MS,U-Pb	Zircon	This study		
635	Within the MOO	W./C. Mongolia	M15821-5.1		Alkali-feldspar granite	101.82	48.57	223	1.0	LA-ICP-MS,U-Pb	Zircon	This study		
636	Within the MOO	W./C. Mongolia	M15822-4.4		Rhyolite	101.29	48.82	238	2.0	LA-ICP-MS,U-Pb	Zircon	This study		
637	Within the MOO	W./C. Mongolia	M15822-7.1		Granite	101.25	48.91	225	1.0	LA-ICP-MS,U-Pb	Zircon	This study		
638	Within the MOO	W./C. Mongolia	M299	Baga Bogd W	Syenite	101.03	44.76	349	6.0	SHRIMP,U-Pb	Zircon	Tomurtogoo O et al.	2005	Journal of the Geological Society
639	Within the MOO	W./C. Mongolia	KHAN 09/46	Tatsin Gol	Granitoid	101.71	46.36	253	5.0	TIMS,U-Pb	Zircon	Yarmolyuk V V, et al	2002	Geotectonics
640	Within the MOO	W./C. Mongolia	P21	Khangai Highland	Granitoid	101.40	45.99	229		TIMS,U-Pb	Zircon	Yarmolyuk V V, et al	2008	Doklady Earth Sciences
641	Within the MOO	W./C. Mongolia	P8	Khangai Highland	Granitoid	98.44	46.59	260		TIMS,U-Pb	Zircon	Yarmolyuk V V, et al	2008	Doklady Earth Sciences
642	Within the MOO	W./C. Mongolia	P28	Khangai Highland	Granitoid	101.17	47.60	255		TIMS,U-Pb	Zircon	Yarmolyuk V V, et al	2008	Doklady Earth Sciences
643	Within the MOO	W./C. Mongolia	P30	Khangai Highland	Granitoid	99.54	47.91	247		TIMS,U-Pb	Zircon	Yarmolyuk V V, et al	2008	Doklady Earth Sciences
644	Within the MOO	W./C. Mongolia	KHT 4/11	Ulan Ula Massif	Granitoid	96.66	45.90	284	1.0	TIMS,U-Pb	Zircon	Yarmolyuk V V, et al	2013	Geology
645	Within the MOO	W./C. Mongolia	KHAN 06/23	Dzhargalant Massif	Granitoid	99.35	48.73	246	1.0	TIMS,U-Pb	Zircon	Yarmolyuk V V, et al	2013	Geology
646	Within the MOO	W./C. Mongolia	P18	Khangai Highland	Granitoid	101.56	45.62	261		TIMS,U-Pb	Zircon	Yarmolyuk V V, et al	2013	Geology
647	Within the MOO	W./C. Mongolia	42	Khangay complex	Qtz diorite	101.92	45.65	261	2.0	TIMS,U-Pb	Zircon	Yarmolyuk V V, et al	2013	Geology
648	Within the MOO	W./C. Mongolia	45	Khangay complex	Granite	102.43	45.74	241	1.5	TIMS,U-Pb	Zircon	Yarmolyuk V V, et al	2013	Geology
649	Within the MOO	W./C. Mongolia	P20	Khangai Highland	Granitoid	101.59	45.93	230		TIMS,U-Pb	Zircon	Yarmolyuk V V, et al	2013	Geology
650	Within the MOO	W./C. Mongolia	P9	Khangai Highland	Granitoid	98.78	46.11	253		TIMS,U-Pb	Zircon	Yarmolyuk V V, et al	2013	Geology
651	Within the MOO	W./C. Mongolia	P10	Khangai Highland	Granitoid	99.25	46.14	260		TIMS,U-Pb	Zircon	Yarmolyuk V V, et al	2013	Geology
652	Within the MOO	W./C. Mongolia	P17	Khangai Highland	Granitoid	100.84	46.22	247		TIMS,U-Pb	Zircon	Yarmolyuk V V, et al	2013	Geology
653	Within the MOO	W./C. Mongolia	69	Nariyn Teel massif	Granite	99.90	46.28	229	6.0	TIMS,U-Pb	Zircon	Yarmolyuk V V, et al	2013	Geology
654	Within the MOO	W./C. Mongolia	BKH 6/5	Erdene Tsogt	Granitoid	100.79	46.29	240	1.0	TIMS,U-Pb	Zircon	Yarmolyuk V V, et al	2013	Geology
655	Within the MOO	W./C. Mongolia	P13	Khangai Highland	Granitoid	99.79	46.30	250		TIMS,U-Pb	Zircon	Yarmolyuk V V, et al	2013	Geology
656	Within the MOO	W./C. Mongolia	P12	Khangai Highland	Granitoid	99.33	46.30	242		TIMS,U-Pb	Zircon	Yarmolyuk V V, et al	2013	Geology
657	Within the MOO	W./C. Mongolia	P7	Khangai Highland	Granitoid	97.67	46.44	293		TIMS,U-Pb	Zircon	Yarmolyuk V V, et al	2013	Geology
658	Within the MOO	W./C. Mongolia	P6	Khangai Highland	Granitoid	97.26	46.48	262		TIMS,U-Pb	Zircon	Yarmolyuk V V, et al	2013	Geology
659	Within the MOO	W./C. Mongolia	P5	Khangai Highland	Granitoid	97.19	46.49	269		TIMS,U-Pb	Zircon	Yarmolyuk V V, et al	2013	Geology
660	Within the MOO	W./C. Mongolia	50	Daltyn-Am stock	Monzogranite	100.04	46.53	241	4.0	TIMS,U-Pb	Zircon	Yarmolyuk V V, et al	2013	Geology
661	Within the MOO	W./C. Mongolia	P14	Khangai Highland	Granitoid	100.16	46.64	250		TIMS,U-Pb	Zircon	Yarmolyuk V V, et al	2013	Geology
662	Within the MOO	W./C. Mongolia	46	Khangay complex	Monzogranite	100.17	46.80	234	5.0	TIMS,U-Pb	Zircon	Yarmolyuk V V, et al	2013	Geology
663	Within the MOO	W./C. Mongolia	P23	Khangai Highland	Granitoid	102.81	46.99	228		TIMS,U-Pb	Zircon	Yarmolyuk V V, et al	2013	Geology
664	Within the MOO	W./C. Mongolia	P16	Khangai Highland	Granitoid	99.76	47.14	249		TIMS,U-Pb	Zircon	Yarmolyuk V V, et al	2013	Geology
665	Within the MOO	W./C. Mongolia	70	Kharkhor massif	Bi. granite	101.66	47.21	228	2.7	TIMS,U-Pb	Zircon	Yarmolyuk V V, et al	2013	Geology
666	Within the MOO	W./C. Mongolia	KHAN 09/41	Buyant Gol	Granitoid	97.93	47.23	258	2.0	TIMS,U-Pb	Zircon	Yarmolyuk V V, et al	2013	Geology
667	Within the MOO	W./C. Mongolia	49	Sharausgol complex	Qtz monzodiorite	100.34	47.41	238	2.5	TIMS,U-Pb	Zircon	Yarmolyuk V V, et al	2013	Geology
668	Within the MOO	W./C. Mongolia	P27	Khangai Highland	Granitoid	101.27	47.49	255		TIMS,U-Pb	Zircon	Yarmolyuk V V, et al	2013	Geology
669	Within the MOO	W./C. Mongolia	47	Sharausgol complex	Granite	101.40	47.60	255	1.0	TIMS,U-Pb	Zircon	Yarmolyuk V V, et al	2013	Geology
670	Within the MOO	W./C. Mongolia	43	Khangay complex	Granodiorite	100.91	47.64	255	1.0	TIMS,U-Pb	Zircon	Yarmolyuk V V, et al	2013	Geology
671	Within the MOO	W./C. Mongolia	P25	Khangai Highland	Granitoid	103.62	47.64	247		TIMS,U-Pb	Zircon	Yarmolyuk V V, et al	2013	Geology
672	Within the MOO	W./C. Mongolia	KHAN 08/34	Yarugin Gol	Granitoid	97.17	47.73	302	8.0	TIMS,U-Pb	Zircon	Yarmolyuk V V, et al	2013	Geology
673	Within the MOO	W./C. Mongolia	P29	Khangai Highland	Granitoid	101.77	47.86	220		TIMS,U-Pb	Zircon	Yarmolyuk V V, et al	2013	Geology
674	Within the MOO	W./C. Mongolia	P31	Khangai Highland	Granitoid	99.84	47.91	267		TIMS,U-Pb	Zircon	Yarmolyuk V V, et al	2013	Geology
675	Within the MOO	W./C. Mongolia	P32	Khangai Highland	Granitoid	99.75	48.12	257		TIMS,U-Pb	Zircon	Yarmolyuk V V, et al	2013	Geology
676	Within the MOO	W./C. Mongolia	P33	Khangai Highland	Granitoid	99.82	48.28	246		TIMS,U-Pb	Zircon	Yarmolyuk V V, et al	2013	Geology
677	Within the MOO	W./C. Mongolia	P11	Khangai Highland	Granitoid	98.82	48.52	268		TIMS,U-Pb	Zircon	Yarmolyuk V V, et al	2013	Geology
678	Within the MOO	W./C. Mongolia	P2	Khangai Highland	Granitoid	95.92	48.52	299		TIMS,U-Pb	Zircon	Yarmolyuk V V, et al	2013	Geology
679	Within the MOO	W./C. Mongolia	P34	Khangai Highland	Granitoid	100.13	48.69	256		TIMS,U-Pb	Zircon	Yarmolyuk V V, et al	2013	Geology
680	Within the MOO	W./C. Mongolia	P1	Khangai Highland	Granitoid	96.40	49.07	264		TIMS,U-Pb	Zircon	Yarmolyuk V V, et al	2013	Geology
681	Within the MOO	W./C. Mongolia	KHAN 07/41	Tosontsengel Massif	Granitoid	98.06	49.14	268	1.0	TIMS,U-Pb	Zircon	Yarmolyuk V V, et al	2013	Geology
682	Within the MOO	Central Mongolia	MO-136	Olzit area	rhyolite	106.31	44.96	207	2.0	SHRIMP,U-Pb	Zircon	Zhu Mingshuai et al.	2016	Journal of Asian Earth Sciences

683	Within the MOO	Central Mongolia	MO-143	Olzit area	miarolitic peralkaline granite	106.28	44.97	209	2.0	SHRIMP,U-Pb	Zircon	Zhu Mingshuai et al.	2016	Journal of Asian Earth Sciences
684	Within the MOO	Central Mongolia	MO-151	Olzit area	biotite-bearing granite	106.29	44.97	213	3.0	SHRIMP,U-Pb	Zircon	Zhu Mingshuai et al.	2016	Journal of Asian Earth Sciences
685	Within the MOO	W./C. Mongolia	15082302A	Khangai Highland	Bt–Hbl granodiorite	100.00	46.81	262	1.8	LA-ICP-MS,U-Pb	Zircon	Dolzodmaa et al.	2020	Mongolian Geoscientist
686	Within the MOO	W./C. Mongolia	11083003A	Khangai Highland	Bt–Hbl granodiorite	100.00	46.81	254	3.8	LA-ICP-MS,U-Pb	Zircon	Dolzodmaa et al.	2020	Mongolian Geoscientist
687	Within the MOO	W./C. Mongolia	11083001A9	Khangai Highland	Bt granite	99.88	46.81	263	5.7	LA-ICP-MS,U-Pb	Zircon	Dolzodmaa et al.	2020	Mongolian Geoscientist
688	Within the MOO	W./C. Mongolia	110829T01F1	Khangai Highland	Granite	99.87	46.80	266	3.0	LA-ICP-MS,U-Pb	Zircon	Dolzodmaa et al.	2020	Mongolian Geoscientist
689	Within the MOO	W./C. Mongolia	15082401A	Khangai Highland	Bt–Hbl granodiorite	99.66	47.24	260	2.0	LA-ICP-MS,U-Pb	Zircon	Dolzodmaa et al.	2020	Mongolian Geoscientist
690	Within the MOO	W./C. Mongolia	15082401C	Khangai Highland	MME in Bt–Hbl granodiorite	99.66	47.24	258	2.7	LA-ICP-MS,U-Pb	Zircon	Dolzodmaa et al.	2020	Mongolian Geoscientist
691	Within the MOO	W./C. Mongolia	11082803A	Khangai Highland	Bt–Hbl granodiorite	100.21	47.72	262	2.0	LA-ICP-MS,U-Pb	Zircon	Dolzodmaa et al.	2020	Mongolian Geoscientist
692	Within the MOO	W./C. Mongolia	15082305D2	Khangai Highland	MME in Bt–Hbl granodiorite	99.87	46.94	262	3.7	LA-ICP-MS,U-Pb	Zircon	Dolzodmaa et al.	2020	Mongolian Geoscientist
693	Within the MOO	W./C. Mongolia	15082801A	Khangai Highland	Bt–Hbl granodiorite	101.97	47.42	249	2.4	LA-ICP-MS,U-Pb	Zircon	Dolzodmaa et al.	2020	Mongolian Geoscientist
694	Within the MOO	W./C. Mongolia	15082002A	Khangai Highland	Bt granite	101.75	45.83	249	3.1	LA-ICP-MS,U-Pb	Zircon	Dolzodmaa et al.	2020	Mongolian Geoscientist
695	Within the MOO	W./C. Mongolia	15082710	Khangai Highland	Pegmatitic granite	101.42	47.50	238	2.2	LA-ICP-MS,U-Pb	Zircon	Dolzodmaa et al.	2020	Mongolian Geoscientist
696	Within the MOO	W./C. Mongolia	15082302D	Khangai Highland	Granitic dike	100.00	46.81	239	2.6	LA-ICP-MS,U-Pb	Zircon	Dolzodmaa et al.	2020	Mongolian Geoscientist
697	Outside of the MOO	Altai	10	Ertix hanging-wall	Granitic gneiss	89.87	47.25	278	7.0	LA-ICP-MS,U-Pb	Zircon	Briggs et al.	2007	Geologcal Society of America Bulletin
698	Outside of the MOO	Altai	HB28	Tuokesalei	Mafic microgranular enclave	86.07	48.32	313	13.0	LA-ICP-MS,U-Pb	Zircon	Cai Keda et al.	2012	Gondwana Research
699	Outside of the MOO	Altai	HB29	Tuokesalei	Granodiorite	86.07	48.32	313	5.0	LA-ICP-MS,U-Pb	Zircon	Cai Keda et al.	2012	Gondwana Research
700	Outside of the MOO	Altai	Mk54	Bodonchin river	Granodiorite	93.17	45.89	354	4.0	LA-ICP-MS,U-Pb	Zircon	Cai Keda et al.	2015	Lithos
701	Outside of the MOO	Altai	Mk57	Bodonchin river	Granodiorite	93.19	45.89	360	5.6	LA-ICP-MS,U-Pb	Zircon	Cai Keda et al.	2015	Lithos
702	Outside of the MOO	Altai	Mk58	Bodonchin	Rapakivi granite	93.19	46.03	317	3.0	LA-ICP-MS,U-Pb	Zircon	Cai Keda et al.	2015	Lithos
703	Outside of the MOO	Altai	Mk33	South Bulgan	Alkali feldspar granite	91.38	46.60	227	3.0	LA-ICP-MS,U-Pb	Zircon	Cai Keda et al.	2015	Lithos
704	Outside of the MOO	Altai	Mk41	North Bulgan	Diorite	92.28	46.63	350	3.0	LA-ICP-MS,U-Pb	Zircon	Cai Keda et al.	2015	Lithos
705	Outside of the MOO	Altai	AT-1	Jianjunshan	Granite	88.81	47.20	150	3.0	SHRIMP,U-Pb	Zircon	Chen Bin et al.	2002	Geol.
706	Outside of the MOO	Altai	22890	Bayan	Biotite–muscovite granite	91.33	46.78	215	3.0	SHRIMP,U-Pb	Zircon	Dash et al.	2016	Journal of Asian Earth Sciences
707	Outside of the MOO	Altai	08AL18	Kalatongke	Ddiorite	89.43	46.75	308	7.0	LA-ICP-MS,U-Pb	Zircon	Gao Jianfeng et al.	2013	Lithos
708	Outside of the MOO	Altai	Be-01	Belokuriha massif (Chernovaya	Granite	84.76	51.94	250	6.0	LA-ICP-MS,U-Pb	Zircon	Glorie et al.	2011	Gondwana Research
709	Outside of the MOO	Altai	11ALT-17	Areletuobie	Granite	90.24	46.53	294	4.6	LA-ICP-MS,U-Pb	Zircon	He Dengfa et al.	2018	Geoscience Frontiers
710	Outside of the MOO	Altai	TS89	Tseel	Metagranodiorite	97.16	45.04	289	2.0	SHRIMP,U-Pb	Zircon	Helo et al.	2006	Chemical Geology
711	Outside of the MOO	Altai	Ga-1	Trans-Altai Gobi	Granitoid	99.03	44.53	271	7.0	TIMS,U-Pb	Zircon	Kozakov et al.	2007	Petrology
712	Outside of the MOO	Altai	8-625	Mesoabyssal	Plagiogranite	81.88	51.13	319	7.0	SHRIMP,U-Pb	Zircon	Kuibida et al.	2013	Russian Geology and Geophysics
713	Outside of the MOO	Altai	Ch-07-13	Baturinka	Tonalites	81.75	49.52	323		LA-ICP-MS,U-Pb	Zircon	Kuibida et al.	2016	Geoscience Frontiers
714	Outside of the MOO	Altai	KYBLK	Keyinbulake	Granite	87.31	47.98	279	3.5	LA-ICP-MS,U-Pb	Zircon	Li Xiangren et al.	2012	Xinjiang Geology(ICWEA)
715	Outside of the MOO	Altai	FY-0	SW-Fuyun	Granitoid	89.49	46.97	291	5.5	LA-ICP-MS,U-Pb	Zircon	Lin Zhengfan et al.	2019	Journal of Asian Earth Sciences
716	Outside of the MOO	Altai	ALR-0	Alaer	Biotite granite	89.71	47.32	216	3.1	LA-ICP-MS,U-Pb	Zircon	Lin Zhengfan et al.	2019	Journal of Asian Earth Sciences
717	Outside of the MOO	Altai	XBD-0	Xibodu	Biotite granite	88.42	47.36	213	4.2	LA-ICP-MS,U-Pb	Zircon	Lin Zhengfan et al.	2019	Journal of Asian Earth Sciences
718	Outside of the MOO	Altai	HLS-0	Halasu	Granite	88.47	47.65	286	11.0	LA-ICP-MS,U-Pb	Zircon	Lin Zhengfan et al.	2019	Journal of Asian Earth Sciences
719	Outside of the MOO	Altai	SKL-0	Shangkelan	Biotite granite	88.23	47.78	209	4.1	LA-ICP-MS,U-Pb	Zircon	Lin Zhengfan et al.	2019	Journal of Asian Earth Sciences
720	Outside of the MOO	Altai	KLS-0	Kalasu	Granite	88.05	47.89	287	4.0	LA-ICP-MS,U-Pb	Zircon	Lin Zhengfan et al.	2019	Journal of Asian Earth Sciences
721	Outside of the MOO	Altai	R	Keketuoha	Granite	89.73	47.33	211	0.8	LA-ICP-MS,U-Pb	Zircon	Liu Feng et al.	2014	Ore Geology Reviews
722	Outside of the MOO	Altai	KP1-08-3	Keketuohai	Pegmatite	89.82	47.19	208	0.8	LA-ICP-MS,U-Pb	Zircon	Ren Baoqin et al.	2011	Acta Mineralogica Sinica(ICWEA)
723	Outside of the MOO	Altai	JMK10-A	Qunkudong	Pegmatite	88.79	47.80	196	1.0	LA-ICP-MS,U-Pb	Zircon	Ren Baoqin et al.	2011	Acta Mineralogica Sinica(ICWEA)
724	Outside of the MOO	Altai	08QH-25	Saertielieke	Alkaline granite	86.46	47.74	308	6.0	LA-ICP-MS,U-Pb	Zircon	Shen Xiaoming et al.	2011	Journal of Asian Earth Sciences
725	Outside of the MOO	Altai	08QH-27	Jierdekala	Alkaline granite	86.47	47.75	291	7.0	LA-ICP-MS,U-Pb	Zircon	Shen Xiaoming et al.	2011	Journal of Asian Earth Sciences
726	Outside of the MOO	Altai	08FY-16	Jiedongkudongke	Rhyolite porphyry	89.62	49.51	279	3.0	LA-ICP-MS,U-Pb	Zircon	Shen Xiaoming et al.	2013	Xinjiang Geology(ICWEA)
727	Outside of the MOO	Altai	T15709-2	Wuzunbulake	Porphyritic granodiorite	94.05	43.52	301	3.0	LA-ICP-MS,U-Pb	Zircon	Song Peng et al.	2019	Lithos
728	Outside of the MOO	Altai	A15703-1	Guersi	Diorite	90.02	46.02	303	3.0	LA-ICP-MS,U-Pb	Zircon	Song Peng et al.	2019	Lithos
729	Outside of the MOO	Altai	A15703-2	Hadanxun	Granite	90.02	46.39	290	3.0	LA-ICP-MS,U-Pb	Zircon	Song Peng et al.	2019	Lithos
730	Outside of the MOO	Altai	A15703-4	Areletuobie	Monzonite	90.31	46.46	285	9.0	LA-ICP-MS,U-Pb	Zircon	Song Peng et al.	2019	Lithos
731	Outside of the MOO	Altai	A15704-4	Chagan	Granodiorite	88.36	46.52	275	2.0	LA-ICP-MS,U-Pb	Zircon	Song Peng et al.	2019	Lithos
732	Outside of the MOO	Altai	A14830-2	Songkeke	Tonalite	90.67	46.81	285	9.0	LA-ICP-MS,U-Pb	Zircon	Song Peng et al.	2019	Lithos
733	Outside of the MOO	Altai	A14829-1	Kungeyite	Granodiorite	90.27	46.95	260	12.0	LA-ICP-MS,U-Pb	Zircon	Song Peng et al.	2019	Lithos
734	Outside of the MOO	Altai	D05126-1	Aletainan	two-mica granite	88.09	47.81	275	1.7	SHRIMP,U-Pb	Zircon	Sun Guihua et al.	2009	Geology in China(ICWEA)
735	Outside of the MOO	Altai	CH15-7	Tadonglang	Granite	87.65	47.91	355	5.0	LA-ICP-MS,U-Pb	Zircon	Sun Min et al.	2009	Sci China Sri D-Earth Sci
736	Outside of the MOO	Altai	Bu9-1	Chonghudongnan	Chonghur south	87.00	48.06	281	10.0	LA-ICP-MS,U-Pb	Zircon	Sun Min et al.	2009	Sci China Sri D-Earth Sci
737	Outside of the MOO	Altai	380105	Takeshiken	Syenogranite	90.91	46.08	286	1.0	TIMS,U-Pb	Zircon	Tong Ying et al.	2006	Acta Petrologica Sinica(ICWEA)
738	Outside of the MOO	Altai	4001	Bulgen	Alkaline granite	90.81	46.19	358	4.0	SHRIMP,U-Pb	Zircon	Tong Ying et al.	2012	Academy of Management Journal
739	Outside of the MOO	Altai	A4	Adenbluk	Monzogranite	88.93	47.05	271	5.0	TIMS,U-Pb	Zircon	Tong Ying et al.	2014	Academy of Management Journal
740	Outside of the MOO	Altai	3055	Xibodu	Monzogranite	88.42	47.36	267	6.0	LA-ICP-MS,U-Pb	Zircon	Tong Ying et al.	2014	Academy of Management Journal
741	Outside of the MOO	Altai	152	Daqiaonan	Bi-granite	88.20	47.52	267	5.0	SHRIMP,U-Pb	Zircon	Tong Ying et al.	2014	Academy of Management Journal
742	Outside of the MOO	Altai	3198	Burjin	Monzogranite	86.81	47.69	268	5.0	LA-ICP-MS,U-Pb	Zircon	Tong Ying et al.	2014	Academy of Management Journal
743	Outside of the MOO	Altai	A3	Aweitan	Granite	88.07	47.80	271	2.0	TIMS,U-Pb	Zircon	Tong Ying et al.	2014	Academy of Management Journal
744	Outside of the MOO	Altai	372			80.25	40.95	225	4.0	TIMS,U-Pb	Zircon	Vladimirov et al.	2001	Geologiyai Geofizika
745	Outside of the MOO	Altai				79.16	41.95	333	3.0	TIMS,U-Pb	Zircon	Vladimirov et al.	2001	Geologiyai Geofizika
746	Outside of the MOO	Altai				77.04	44.72	301	7.0	TIMS,U-Pb	Zircon	Vladimirov et al.	2001	Geologiyai Geofizika
747	Outside of the MOO	Altai	QMQK	Qiemuerqieke	gabbro	87.88	47.78	276	2.1	SHRIMP,U-Pb	Zircon	Wan Bo et al.	2013	Lithosphere
748	Outside of the MOO	Altai	ASK-6	Asikadongte	Muscovite sodium feldspar granite	90.30	47.29	219	2.9	LA-ICP-MS,U-Pb	Zircon	Wang Chunlong et al.	2015	Acta Petrologica Sinica(ICWEA)
749	Outside of the MOO	Altai	ASK-1	Asikadongte	Muscovite sodium feldspar granite	90.30	47.29	223	4.6	LA-ICP-MS,U-Pb	Zircon	Wang Chunlong et al.	2015	Acta Petrologica Sinica(ICWEA)
750	Outside of the MOO	Altai	258	Lamazhao	granite	88.42	47.75	276	9.0	SHRIMP,U-Pb	Zircon	Wang Tao et al.	2005	Acta Petrologica Sinica(ICWEA)
751	Outside of the MOO	Altai	T5	Keketuohai	granite	88.81	47.20	198	7.0	SHRIMP,U-Pb	Zircon	Wang Tao et al.	2007	Ore Geology Reviews
752	Outside of the MOO	Altai	122/1	Keketuohai	granite	88.81	47.20	212	6.0	SHRIMP,U-Pb	Zircon	Wang Tao et al.	2007	Ore Geology Reviews
753	Outside of the MOO	Altai	T1	Keketuohai	granite	88.81	47.20	220	9.0	SHRIMP,U-Pb	Zircon	Wang Tao et al.	2007	Ore Geology Reviews
754	Outside of the MOO	Altai	3037	Aler	Monzogranite	88.83	47.50	210	3.0	LA-ICP-MS,U-Pb	Zircon	Wang Tao et al.	2014	American Journal of Sociology
755	Outside of the MOO	Altai	3038	Aler	Monzogranite	88.83	47.50	213	1.0	LA-ICP-MS,U-Pb	Zircon	Wang Tao et al.	2014	American Journal of Sociology
756	Outside of the MOO	Altai	3033	Aler	Monzogranite	88.83	47.50	216	1.0	LA-ICP-MS,U-Pb	Zircon	Wang Tao et al.	2014	American Journal of Sociology
757	Outside of the MOO	Altai	3214	Shangkelan	Granite	88.23	47.78	220	1.0	SHRIMP,U-Pb	Zircon	Wang Tao et al.	2014	American Journal of Sociology
758	Outside of the MOO	Altai	3013	Ala'er	Monzogranite	86.70	48.16	212	2.0	TIMS,U-Pb	Zircon	Wang Tao et al.	2014	American Journal of Sociology
759	Outside of the MOO	Altai	YLKZK1204-9	wall rock of the Aketas gold de		88.75	47.75	309	4.7	LA-ICP-MS,U-Pb	Zircon	Wei Xiaofeng et al.	2015	Chinese Journal of Geochemistry
760	Outside of the MOO	Altai	HL-017	Jiaerbasidao	granite	90.06	46.58	287	2.6	SHRIMP,U-Pb	Zircon	Yang Fuquan et al.	2013	Ore Geology Reviews
761	Outside of the MOO	Altai	HL-028	Kuerqis Fe deposit	granite	90.03	46.60	274	0.5	LA-ICP-MS,U-Pb	Zircon	Yang Fuquan et al.	2013	Ore Geology Reviews

762	Outside of the MOO	Altai	AG-01	Kuerqis Fe deposit	granite	90.03	46.60	279	0.9	LA-ICP-MS,U-Pb	Zircon	Yang Fuquan et al.	2013	Ore Geology Reviews
763	Outside of the MOO	Altai	JEBSD	Jiaerbasidao	Granodiorite	88.57	47.57	287	2.6	SHRIMP,U-Pb	Zircon	Yang Fuquan et al.	2013	Ore Geology Reviews
764	Outside of the MOO	Altai	ALT14-53	Habahe	Biotite granite	89.49	47.12	359	4.0	LA-ICP-MS,U-Pb	Zircon	Yu Yang et al.	2019	Journal of Asian Earth Sciences
765	Outside of the MOO	Altai	FY-77	Keketuohai	biotite granodiorite	89.82	47.05	359	5.0	LA-ICP-MS,U-Pb	Zircon	Yuan Chao et al.	2007	Chemical Geology
766	Outside of the MOO	Altai	Alt-13	Ashile	granodiorite	86.83	48.33	318	6.0	LA-ICP-MS,U-Pb	Zircon	Yuan Chao et al.	2007	Chemical Geology
767	Outside of the MOO	Altai	Daqiao-East-01	Daqio East	Granite	89.68	47.38	340	3.0	LA-ICP-MS,U-Pb	Zircon	Zhang Chen et al.	2017	Gondwana Research
768	Outside of the MOO	Altai	Tawati	Tawati	Granite	90.49	46.84	334	4.9	LA-ICP-MS,U-Pb	Zircon	Zhang Chen et al.	2019	Lithos
769	Outside of the MOO	Altai	Qinghe-North	Qinghe-North	Granite	90.25	46.86	345	4.9	LA-ICP-MS,U-Pb	Zircon	Zhang Chen et al.	2019	Lithos
770	Outside of the MOO	Altai	Kumaier	Kumaier	Granite	90.01	46.92	331	4.5	LA-ICP-MS,U-Pb	Zircon	Zhang Chen et al.	2019	Lithos
771	Outside of the MOO	Altai			Granite	82.07	51.33	251	5.0	TIMS,U-Pb	Zircon	Vladimirov A.G et al.	1997	Geologiyai Geofizika
772	Outside of the MOO	Altai	3820		Granite	82.48	51.16	244	2.0	TIMS,U-Pb	Zircon	Vladimirov A.G et al.	2001	Geologiyai Geofizika
773	Outside of the MOO	Altai			Granite	87.51	49.65	182	19.0	TIMS,U-Pb	Zircon	Vladimirov A.G et al.	2001	Geologiyai Geofizika
774	Outside of the MOO	Altai	353		Granite	81.77	50.28	245	7.0	TIMS,U-Pb	Zircon	Vladimirov A.G et al.	2005	Geologiyai Geofizika
775	Outside of the MOO	Altai	Pa-23		Granite	86.33	49.53	196	4.0	TIMS,U-Pb	Zircon	Vladimirov A.G et al.	2005	Geologiyai Geofizika
776	Outside of the MOO	Altai			Granite	82.88	49.55	231	11.0	TIMS,U-Pb	Zircon	Vladimirov A.G et al.	2005	Geologiyai Geofizika
777	Outside of the MOO	Altai	354		Granite	86.62	49.68	245	2.0	TIMS,U-Pb	Zircon	Vladimirov A.G et al.	2005	Geologiyai Geofizika
778	Outside of the MOO	Altai	08TK16	Irtish belt	Diorite	82.71	50.02	252	2.6	LA-ICP-MS,U-Pb	Zircon	Zhang Chuanlin et al.	2012	Journal of Asian Earth Sciences
779	Outside of the MOO	Altai	08TK15	Irtish belt	Gneiss granite	82.71	50.02	281	1.0	LA-ICP-MS,U-Pb	Zircon	Zhang Chuanlin et al.	2012	Journal of Asian Earth Sciences
780	Outside of the MOO	Altai	JMK-09	Qiongkuer Village	Pegmatite	88.81	47.83	192	2.3	LA-ICP-MS,U-Pb	Zircon	Zhang Xin et al.	2016	Journal of Asian Earth Sciences
781	Outside of the MOO	Altai	AZB-01	Kaluan–Azubai–Qiongkuer	Pegmatite	88.83	47.87	192	2.0	LA-ICP-MS,U-Pb	Zircon	Zhang Xin et al.	2016	Journal of Asian Earth Sciences
782	Outside of the MOO	Altai	KLA803	Kaluan–Azubai–Qiongkuer	Granite	88.83	47.91	225	2.3	LA-ICP-MS,U-Pb	Zircon	Zhang Xin et al.	2016	Journal of Asian Earth Sciences
783	Outside of the MOO	Altai	456920	Aladong	Biotite monzonitic granite	89.73	47.33	219	3.0	LA-ICP-MS,U-Pb	Zircon	Zhang Yafeng et al.	2015	Acta Geologica Sinica(ICWEA)
784	Outside of the MOO	Altai	1111-1	Aladong	Biotite feldspar granite	89.62	47.46	210	5.0	LA-ICP-MS,U-Pb	Zircon	Zhang Yafeng et al.	2015	Acta Geologica Sinica(ICWEA)
785	Outside of the MOO	Altai	o9-2	Wuqiagou	Granite	89.75	47.00	294	6.0	LA-ICP-MS,U-Pb	Zircon	Zhang Yue et al.	2015	Northwestern Geology(ICWEA)
786	Outside of the MOO	Altai	KZ	Kezile	Granite	88.40	47.35	290	4.3	LA-ICP-MS,U-Pb	Zircon	Zhao Yumei et al.	2016	Geology and Prospecting(ICWEA)
787	Outside of the MOO	Altai	H01	Wodongke	Granite	90.06	46.58	299	4.1	SHRIMP,U-Pb	Zircon	Zhou Gang et al.	2015	Geology in China(ICWEA)
788	Outside of the MOO	Altai	ZR01	Sarewuzeng	Granite	88.50	47.50	318	1.5	SHRIMP,U-Pb	Zircon	Zhou Gang et al.	2015	Geology in China(ICWEA)
789	Outside of the MOO	Altai	09HTW1	Wodongke	Granite	85.98	48.37	299	4.1	SHRIMP,U-Pb	Zircon	Zhou Gang et al.	2015	Geology in China(ICWEA)
790	Outside of the MOO	Altai	09HTW2	Sarewuzeng	Granite	85.99	48.36	318	1.5	SHRIMP,U-Pb	Zircon	Zhou Gang et al.	2015	Geology in China(ICWEA)
791	Outside of the MOO	Altai	D-1	No. 3 pegmatite dike	Pegmatite	89.81	47.20	187	2.0	LA-ICP-MS,U-Pb	Zircon	Zhou Qifeng et al.	2015	Resource Geology
792	Outside of the MOO	Altai	D-3	No. 3 pegmatite dike	Pegmatite	89.81	47.20	187	2.7	LA-ICP-MS,U-Pb	Zircon	Zhou Qifeng et al.	2015	Resource Geology
793	Outside of the MOO	Altai	D-7	No. 3 pegmatite dike	Pegmatite	89.81	47.20	193	6.4	LA-ICP-MS,U-Pb	Zircon	Zhou Qifeng et al.	2015	Resource Geology
794	Outside of the MOO	Altai	D-8	No. 3 pegmatite dike	Pegmatite	89.81	47.20	199	4.2	LA-ICP-MS,U-Pb	Zircon	Zhou Qifeng et al.	2015	Resource Geology
795	Outside of the MOO	Altai	D-4	No. 3 pegmatite dike	Pegmatite	89.81	47.20	211	6.3	LA-ICP-MS,U-Pb	Zircon	Zhou Qifeng et al.	2015	Resource Geology
796	Outside of the MOO	Alxa	D019	Alxa youqi Jinchangshan	Granodiorite	100.93	39.68	294		LA-ICP-MS,U-Pb	Zircon	Chen Wei et al.	2013	Mineralogy and Petrology(ICWEA)
797	Outside of the MOO	Alxa	09AL22-1	nuoergong	Monzogranite	104.83	39.89	278	2.0	SIMS,U-Pb	Zircon	Dan Wei et al.	2014	Lithos
798	Outside of the MOO	Alxa	09AL22-2	nuoergong	Monzogranite	104.83	39.89	278		LA-ICP-MS,U-Pb	Zircon	Dan Wei et al.	2014	Lithos
799	Outside of the MOO	Alxa	09AL12-1	Nuoergong	A-type granite	104.97	39.93	279	2.0	LA-ICP-MS,U-Pb	Zircon	Dan Wei et al.	2014	Lithos
800	Outside of the MOO	Alxa	09AL25-1	Bayannuoergong batholith	Monzogranite	104.90	39.98	278		SIMS,U-Pb	Zircon	Dan Wei et al.	2014	Lithos
801	Outside of the MOO	Alxa	09AL27	nuoergong	Monzogranite	104.89	40.10	284	3.0	SIMS,U-Pb	Zircon	Dan Wei et al.	2014	Lithos
802	Outside of the MOO	Alxa	09AL29	nuoergong	Monzogranite	104.89	40.10	284		SIMS,U-Pb	Zircon	Dan Wei et al.	2014	Lithos
803	Outside of the MOO	Alxa	09AL35	Bayannuoergong batholith	Granodiorite	104.77	40.16	281	2.0	SIMS,U-Pb	Zircon	Dan Wei et al.	2014	Lithos
804	Outside of the MOO	Alxa	09AL37	Bayannuoergong batholith	Granodiorite	104.71	40.17	283	2.0	LA-ICP-MS,U-Pb	Zircon	Dan Wei et al.	2014	Lithos
805	Outside of the MOO	Alxa	09AL38	Bayannuoergong batholith	Granodiorite	104.71	40.17	283		SIMS,U-Pb	Zircon	Dan Wei et al.	2014	Lithos
806	Outside of the MOO	Alxa	09AL39	Bayannuoergong batholith	Granodiorite	104.71	40.17	283		SIMS,U-Pb	Zircon	Dan Wei et al.	2014	Lithos
807	Outside of the MOO	Alxa	09AL70	Bijierai	Diorite	104.38	40.39	280	2.0	SIMS,U-Pb	Zircon	Dan Wei et al.	2014	Lithos
808	Outside of the MOO	Alxa	09AL73	Bayannuoergong batholith	Quartz diorite	104.38	40.40	282	2.0	LA-ICP-MS,U-Pb	Zircon	Dan Wei et al.	2014	Lithos
809	Outside of the MOO	Alxa	09AL28-1	Bayannuoergong batholith	Bayannuoergong mafic enclave	104.89	40.10	278		SIMS,U-Pb	Zircon	Dan Wei et al.	2015	Lithos
810	Outside of the MOO	Alxa	09AL152	Bayannuoergong batholith	Yamaitu granodiorite	105.49	40.24	270		SIMS,U-Pb	Zircon	Dan Wei et al.	2015	Lithos
811	Outside of the MOO	Alxa	09AL157	Bayannuoergong batholith	Yamaitu mafic enclave	105.49	40.24	272		SIMS,U-Pb	Zircon	Dan Wei et al.	2015	Lithos
812	Outside of the MOO	Alxa	09AL127	Hetun	Monzogranite	104.97	39.42	345	4.0	SIMS,U-Pb	Zircon	Dan Wei et al.	2016	Gondwana Research
813	Outside of the MOO	Alxa	09AL200	South Diebusige	Monzogranite	106.15	40.47	337	5.0	SIMS,U-Pb	Zircon	Dan Wei et al.	2016	Gondwana Research
814	Outside of the MOO	Alxa	09AL198	Diebusige	Monzogranite	106.15	40.47	344	5.0	SIMS,U-Pb	Zircon	Dan Wei et al.	2016	Gondwana Research
815	Outside of the MOO	Alxa	09AL191	Diebusige	Monzogranite	106.15	40.47	346	4.0	SIMS,U-Pb	Zircon	Dan Wei et al.	2016	Gondwana Research
816	Outside of the MOO	Alxa	11BJ01E	Bijiertai	Gabbro	104.55	40.40	274	3.0	LA-ICP-MS,U-Pb	Zircon	Feng JY et al.	2013	Journal of Asian Earth Sciences
817	Outside of the MOO	Alxa	11S03-5	Qinggele	Gabbro	105.38	40.48	306	3.0	LA-ICP-MS,U-Pb	Zircon	Feng JY et al.	2013	Journal of Asian Earth Sciences
818	Outside of the MOO	Alxa	AL0710-1	Bayanhonggerisumu	Biotite plagioclase gneiss	105.72	40.47	286	2.1	Zr LA-ICP-MS,U-P	Zircon	Geng Yuanshen et al.	2012	ACTA PETROLOGICA SINICA(ICWEA)
819	Outside of the MOO	Alxa	AL0822-1	Deerhetongdaoban	Biotite plagioclase gneiss	105.51	40.11	276	2.0	LA-ICP-MS,U-Pb	Zircon	Geng Yuansheng et a	2012	Acta Petrologica Sinica(ICWEA)
820	Outside of the MOO	Alxa	AL0822-3	Deerhetongdaoban	Gneissic granite	105.51	40.11	287	2.0	LA-ICP-MS,U-Pb	Zircon	Geng Yuansheng et a	2012	Acta Petrologica Sinica(ICWEA)
821	Outside of the MOO	Alxa	AL0718-1	Honggeeryulin	Tonalite	105.50	40.23	269	2.0	LA-ICP-MS,U-Pb	Zircon	Geng Yuansheng et a	2012	Acta Petrologica Sinica(ICWEA)
822	Outside of the MOO	Alxa	AL0805-1	Bijiertaishan	Granodioritic gneiss	104.46	40.39	284	3.0	LA-ICP-MS,U-Pb	Zircon	Geng Yuansheng et a	2012	Acta Petrologica Sinica(ICWEA)
823	Outside of the MOO	Alxa	AL0805-4	Bijiertaishan	Granodioritic gneiss	104.46	40.39	289	3.0	LA-ICP-MS,U-Pb	Zircon	Geng Yuansheng et a	2012	Acta Petrologica Sinica(ICWEA)
824	Outside of the MOO	Alxa	AL0810-1	Gandegannan	Dioritic gneiss	104.37	40.42	276	2.0	LA-ICP-MS,U-Pb	Zircon	Geng Yuansheng et a	2012	Acta Petrologica Sinica(ICWEA)
825	Outside of the MOO	Alxa	AL0810-2	Gandegannan	Dioritic gneiss	104.37	40.42	279	2.0	LA-ICP-MS,U-Pb	Zircon	Geng Yuansheng et a	2012	Acta Petrologica Sinica(ICWEA)
826	Outside of the MOO	Alxa	AL0709-1	Bayanhonggerisumu	Dioritic gneiss	105.86	40.44	276	2.0	LA-ICP-MS,U-Pb	Zircon	Geng Yuansheng et a	2012	Acta Petrologica Sinica(ICWEA)
827	Outside of the MOO	Alxa	AL0705-1	Bayanhonggerisumu	Dioritic gneiss	105.92	40.49	270	2.0	LA-ICP-MS,U-Pb	Zircon	Geng Yuansheng et a	2012	Acta Petrologica Sinica(ICWEA)
828	Outside of the MOO	Alxa	LS11-3-3.1	Nianpangou	Weakly deformed granite	102.24	39.10	324	2.6	LA-MC-ICP-MS	Zircon	Gong Jianghua et al.	2018	Acta Petrologica et Mineralogica(ICWEA)
829	Outside of the MOO	Alxa	LS11-3-2.1	Nianpangou	Weakly deformed granite	102.24	39.10	327	1.2	LA-MC-ICP-MS	Zircon	Gong Jianghua et al.	2018	Acta Petrologica et Mineralogica(ICWEA)
830	Outside of the MOO	Alxa	LS10-5-1.1	Yejili	hornblende gabbro	102.56	39.14	267	1.8	LA-ICP-MS,U-Pb	Zircon	Gong Jianghua et al.	2018	Acta Geologica Sinica(ICWEA)
831	Outside of the MOO	Alxa	LS10-5-4.1	Yejili	Gabbro	102.49	39.15	293	2.2	LA-ICP-MS,U-Pb	Zircon	Gong Jianghua et al.	2018	Acta Geologica Sinica(ICWEA)
832	Outside of the MOO	Alxa	T2-019	Eastgovi Province	Granite cataclastic	106.61	42.66	314	5.0	SHRIMP,U-Pb	Zircon	Guy Alexandra et al.	2014	Gondwana Research
833	Outside of the MOO	Alxa	T2-015	Eastgovi Province	Granodiorite pegmatoid	106.87	42.79	288	8.0	SHRIMP,U-Pb	Zircon	Guy Alexandra et al.	2014	Gondwana Research
834	Outside of the MOO	Alxa	T2-025	Eastgovi Province	Granite-porphyry	106.52	42.94	329	6.0	SHRIMP,U-Pb	Zircon	Guy Alexandra et al.	2014	Gondwana Research
835	Outside of the MOO	Alxa	T2-029	Eastgovi Province	Granite	106.21	43.01	319	5.0	SHRIMP,U-Pb	Zircon	Guy Alexandra et al.	2014	Gondwana Research
836	Outside of the MOO	Alxa	T1-302	Eastgovi Province	Granite porphyry	102.88	43.14	319	6.0	SHRIMP,U-Pb	Zircon	Guy Alexandra et al.	2014	Gondwana Research
837	Outside of the MOO	Alxa	T2-042	Eastgovi Province	Granodiorite	106.72	43.23	318	8.0	SHRIMP,U-Pb	Zircon	Guy Alexandra et al.	2014	Gondwana Research
838	Outside of the MOO	Alxa	T2-046	Eastgovi Province	Granite	106.83	43.40	318	9.0	SHRIMP,U-Pb	Zircon	Guy Alexandra et al.	2014	Gondwana Research
839	Outside of the MOO	Alxa	HQ0781	Yinggete-Bagemaode	Granite	105.74	41.54	313	4.5	SHRIMP,U-Pb	Zircon	Han Baofu et al.	2010	Acta Petrologica Et Mineralogica(ICWEA)

840	Outside of the MOO	Alxa	HQ0794	Kuchuwula	Granite	105.25	41.65	277	2.0	SHRIMP,U-Pb	Zircon	Han Baofu et al.	2010	Acta Petrologica Et Mineralogica(ICWEA)
841	Outside of the MOO	Alxa	H.Q0794	Kuchuwula	Granite	105.25	41.65	277	2.1	SHRIMP,U-Pb	Zircon	Han Baofu et al.	2010	Acta Petrologica Et Mineralogica(ICWEA)
842	Outside of the MOO	Alxa	HQ0779	Yilian	Granite	105.43	41.68	278	4.4	SHRIMP,U-Pb	Zircon	Han Baofu et al.	2010	Acta Petrologica Et Mineralogica(ICWEA)
843	Outside of the MOO	Alxa	HQ0799	Yilian	Granite	105.43	41.68	278	4.0	SHRIMP,U-Pb	Zircon	Han Baofu et al.	2010	Acta Petrologica Et Mineralogica(ICWEA)
844	Outside of the MOO	Alxa	DSM-6	Dongshengmiao pluton	Quartz diorite	107.05	41.10	275	1.0	LA-ICP-MS,U-Pb	Zircon	Hu Chuansheng et al.	2015	International Geology Review
845	Outside of the MOO	Alxa	DSM-2	Dongshengmiao pluton	Porphyritic granite	107.06	41.11	284	2.0	LA-ICP-MS,U-Pb	Zircon	Hu Chuansheng et al.	2015	International Geology Review
846	Outside of the MOO	Alxa	DSM-1	Dongshengmiao pluton	Porphyritic granite	107.06	41.12	287	1.0	LA-ICP-MS,U-Pb	Zircon	Hu Chuansheng et al.	2015	International Geology Review
847	Outside of the MOO	Alxa	11S04-7	Qinggele	Gabbro	105.67	40.33	262		LA-ICP-MS,U-Pb	Zircon	Jianyun Feng et al.	2013	Journal of Asian Earth Sciences
848	Outside of the MOO	Alxa	11BJ02F	Bijiertai	Peridotite	104.55	40.40	274	3.0	LA-ICP-MS,U-Pb	Zircon	Jianyun Feng et al.	2013	Journal of Asian Earth Sciences
849	Outside of the MOO	Alxa	KHB-1807	Khan-Bogd massif	Peralkaline granite	107.62	42.98	290	1.0	TIMS,U-Pb	Zircon	Kröner et al. unpublished		
850	Outside of the MOO	Alxa	Not-given	Noen and Tost Ranges	Peralk. granite dyke	100.87	43.00	318	1.0	TIMS,U-Pb	Zircon	Kröner et al. unpublished		
851	Outside of the MOO	Alxa	PM38-11-3	Bayinnuoergong	Enclave (monzodiorite)	104.82	40.02	277		LA-ICP-MS,U-Pb	Zircon	Li Jie	2012	China University of Geosciences, Beijing:Master Dissertation
852	Outside of the MOO	Alxa	PM39-15-1	Bayinnuoergong	Monzongranite	104.82	40.02	277		LA-ICP-MS,U-Pb	Zircon	Li Jie	2012	China University of Geosciences, Beijing:Master Dissertation
853	Outside of the MOO	Alxa	PM133-3-1	bayanwulashan	granidiorite	104.40	40.08	265		LA-ICP-MS,U-Pb	Zircon	Li Jie	2012	China University of Geosciences, Beijing:Master Dissertation
854	Outside of the MOO	Alxa	PM133-6-2	bayanwulashan	Enclave (monzodiorite)	104.40	40.08	265		LA-ICP-MS,U-Pb	Zircon	Li Jie	2012	China University of Geosciences, Beijing:Master Dissertation
855	Outside of the MOO	Alxa	PM133-7-2	bayanwulashan	Enclave (monzodiorite)	104.40	40.08	265		LA-ICP-MS,U-Pb	Zircon	Li Jie	2012	China University of Geosciences, Beijing:Master Dissertation
856	Outside of the MOO	Alxa	PM38-15-1	Bayinnuoergong	Enclave (monzodiorite)	104.40	40.08	265		LA-ICP-MS,U-Pb	Zircon	Li Jie	2012	China University of Geosciences, Beijing:Master Dissertation
857	Outside of the MOO	Alxa	P133	bayanwulashan	Granodiorite	104.34	40.65	265	1.3	LA-ICP-MS,U-Pb	Zircon	Li Jie	2012	China University of Geosciences, Beijing:Master Dissertation
858	Outside of the MOO	Alxa	WD9016	Bayinnuoer	Monzogranite	104.86	40.68	278	1.0	LA-ICP-MS,U-Pb	Zircon	Li Jie	2012	China University of Geosciences, Beijing:Master Dissertation
859	Outside of the MOO	Alxa	AHD5TW1	Honggueryulin	Granite	105.52	40.15	273	1.0	TIMS,U-Pb	Zircon	Li Junjian et al.	2006	China University of Geosciences, Beijing:Phd Dissertation
860	Outside of the MOO	Alxa	AD58TW3	Habudahalashan	Quartz porphyry	104.28	40.30	299	5.6	TIMS,U-Pb	Zircon	Li Junjian et al.	2006	China University of Geosciences, Beijing:Phd Dissertation
861	Outside of the MOO	Alxa	AD81TW1	Honggueryulin	Granite	105.40	40.47	295	1.7	TIMS,U-Pb	Zircon	Li Junjian et al.	2006	China University of Geosciences, Beijing:Phd Dissertation
862	Outside of the MOO	Alxa	AD110TW1	Wulijibei	Granodiorite	104.47	40.83	254	3.3	SHRIMP,U-Pb	Zircon	Li Junjian et al.	2006	China University of Geosciences, Beijing:Phd Dissertation
863	Outside of the MOO	Alxa	AD111TW1	Baogeqi	Gabbro	104.25	40.94	266	4.5	SHRIMP,U-Pb	Zircon	Li Junjian et al.	2006	China University of Geosciences, Beijing:Phd Dissertation
864	Outside of the MOO	Alxa	AD66-4	Zhulazhaga	Granite porphyry	105.17	39.61	280	6.0	SHRIMP,U-Pb	Zircon	Li Junjian et al.	2010	Earth Science Frontiers(ICWEA)
865	Outside of the MOO	Alxa	AD113	Zhulazhaga	Granite porphyry	104.84	40.18	280	6.0	SHRIMP,U-Pb	Zircon	Li Junjian et al.	2010	Earth Science Frontiers(ICWEA)
866	Outside of the MOO	Alxa	AD67-2TW1	Zhulazhaga	Dioritic porphyrite	104.84	40.18	280	5.0	SHRIMP,U-Pb	Zircon	Li Junjian et al.	2010	Earth Science Frontiers(ICWEA)
867	Outside of the MOO	Alxa	DSM04A	Dongshenmiao area	Granitic porphyries	107.05	41.10	285	2.1	LA-ICP-MS,U-Pb	Zircon	Lin LiNa, Xiao Wenji	2014	American Journal of Science
868	Outside of the MOO	Alxa	HG05B	Wenguanshan	Granitic porphyries	107.48	41.50	292	2.1	LA-ICP-MS,U-Pb	Zircon	Lin LiNa, Xiao Wenji	2014	American Journal of Science
869	Outside of the MOO	Alxa	HG11	Wenguanshan area	Dolerite dyke	107.76	41.51	263	1.7	LA-ICP-MS,U-Pb	Zircon	Lin LiNa, Xiao Wenji	2014	American Journal of Science
870	Outside of the MOO	Alxa	HG22A	Wujiahe	Granitic porphyries	108.05	41.76	292	2.1	LA-ICP-MS,U-Pb	Zircon	Lin LiNa, Xiao Wenji	2014	American Journal of Science
871	Outside of the MOO	Alxa	TW3598-2	Hanggale	Enclave	103.18	40.73	258	3.2	LA-ICP-MS,U-Pb	Zircon	Liu zhibo et al.	2014	Geological Review(ICWEA)
872	Outside of the MOO	Alxa	P13TW	Hanggale	Granodiorite	103.18	40.73	258	3.1	LA-ICP-MS,U-Pb	Zircon	Liu zhibo et al.	2014	Geological Review(ICWEA)
873	Outside of the MOO	Alxa	HX1619-2	Hanggale	Diorite xenoliths	103.18	40.73	258	3.2	LA-ICP-MS,U-Pb	Zircon	Liu zhibo et al.	2014	Geological Review(ICWEA)
874	Outside of the MOO	Alxa	HX1619-3	Hanggale	Granodiorite	103.18	40.73	258	3.2	LA-ICP-MS,U-Pb	Zircon	Liu zhibo et al.	2014	Geological Review(ICWEA)
875	Outside of the MOO	Alxa	WZ11	Kebu massif	Diorite	106.17	40.61	291	4.0	SHRIMP,U-Pb	Zircon	Luo hongling et al.	2007	Acta Petrologica Et Mineralogica(ICWEA)
876	Outside of the MOO	Alxa	ZQ05-06	Wuliangsitai pluton from Urad	A-type granite	108.19	41.34	277	3.0	SHRIMP,U-Pb	Zircon	Luo hongling et al.	2009	Acta Petrologica Et Mineralogica(ICWEA)
877	Outside of the MOO	Alxa	PM029-11-YQ1	Wuliji-Tamusu	Granidiorite	104.58	40.84	266	3.7	LA-ICP-MS,U-Pb	Zircon	Lv Bingting	2013	China University of Geosciences, Beijing:Master Dissertation
878	Outside of the MOO	Alxa	PM131-13-YQ1	Wuliji-Tamusu	Biotite monzogranite	104.54	40.91	250	1.7	LA-ICP-MS,U-Pb	Zircon	Lv Bingting	2013	China University of Geosciences, Beijing:Master Dissertation
879	Outside of the MOO	Alxa	PM041-3-YQ1	Wuliji-Tamusu	K-feldspar granite	104.76	40.91	250	1.6	LA-ICP-MS,U-Pb	Zircon	Lv Bingting	2013	China University of Geosciences, Beijing:Master Dissertation
880	Outside of the MOO	Alxa	HZ822-13	Huogeqi, Langshan	Diorite	106.68	40.28	274	1.0	LA-MC-ICP-MS	Zircon	Pi Qiaohui et al.	2010	Mineral Deposits(ICWEA)
881	Outside of the MOO	Alxa	14LQ26A	Bayan Nuru area	Quartz diorite	101.07	39.47	269	1.0	LA-ICP-MS,U-Pb	Zircon	Qian Liu et al.	2017	Litos
882	Outside of the MOO	Alxa	14LQ30A	Bayan Nuru area	Syenogranite	100.81	39.58	268	1.0	LA-ICP-MS,U-Pb	Zircon	Qian Liu et al.	2017	Litos
883	Outside of the MOO	Alxa	14LQ31A	Bayan Nuru area	Syenogranite	100.67	39.61	269	1.0	LA-ICP-MS,U-Pb	Zircon	Qian Liu et al.	2017	Litos
884	Outside of the MOO	Alxa	14LQ33A	Bayan Nuru area	Granodiorite	100.59	39.65	281	2.0	LA-ICP-MS,U-Pb	Zircon	Qian Liu et al.	2017	Litos
885	Outside of the MOO	Alxa	14LQ34A	Bayan Nuru area	Granodiorite	103.13	39.72	277	1.0	LA-ICP-MS,U-Pb	Zircon	Qian Liu et al.	2017	Litos
886	Outside of the MOO	Alxa	14LQ35A	Bayan Nuru area	Granodiorite	103.07	39.89	270	1.0	LA-ICP-MS,U-Pb	Zircon	Qian Liu et al.	2017	Litos
887	Outside of the MOO	Alxa	14LQ01A	Bayan Nuru area	Monzogranite	105.01	39.90	268	1.0	LA-ICP-MS,U-Pb	Zircon	Qian Liu et al.	2017	Litos
888	Outside of the MOO	Alxa	14LQ07A	Bayan Nuru area	Hornblende gabbro	104.92	39.93	300	1.0	LA-ICP-MS,U-Pb	Zircon	Qian Liu et al.	2017	Litos
889	Outside of the MOO	Alxa	14LQ10B	Bayan Nuru area	Hornblende gabbro	104.85	39.96	279	1.0	LA-ICP-MS,U-Pb	Zircon	Qian Liu et al.	2017	Litos
890	Outside of the MOO	Alxa	14LQ11D	Bayan Nuru area	Quartz diorite	104.91	39.99	268	1.0	LA-ICP-MS,U-Pb	Zircon	Qian Liu et al.	2017	Litos
891	Outside of the MOO	Alxa	14LQ14A	Bayan Nuru area	Syenogranite	104.81	40.19	281	1.0	LA-ICP-MS,U-Pb	Zircon	Qian Liu et al.	2017	Litos
892	Outside of the MOO	Alxa	14LQ38A	Bayan Nuru area	Granodiorite	104.47	40.83	241	1.0	LA-ICP-MS,U-Pb	Zircon	Qian Liu et al.	2017	Litos
893	Outside of the MOO	Alxa	14LQ44A	Bayan Nuru area	Monzogranite	104.35	40.92	242	2.0	LA-ICP-MS,U-Pb	Zircon	Qian Liu et al.	2017	Litos
894	Outside of the MOO	Alxa	P4-16HX1	Alxa youqi Hanggale	Monzogranite	103.18	40.84	253		LA-ICP-MS,U-Pb	Zircon	Ran Gao et al.	2012	Geological Bulletin of China(ICWEA)
895	Outside of the MOO	Alxa	P4-16HX3	Alxa youqi Hanggale	Monzogranite	103.18	40.84	254	3.3	LA-ICP-MS,U-Pb	Zircon	Ran Gao et al.	2012	Geological Bulletin of China(ICWEA)
896	Outside of the MOO	Alxa	11LS175	Wuliqidong	Monzogranite	104.87	40.99	252	2.0	LA-ICP-MS,U-Pb	Zircon	Shi Xingjun	2015	China University of Geosciences, Beijing:Disseration Dissertation
897	Outside of the MOO	Alxa	LS035	Halinudeng	Granite	105.75	40.40	284	1.8	LA-ICP-MS,U-Pb	Zircon	Shi Xingjun et al.	2012	Geological Bulletin of China(ICWEA)
898	Outside of the MOO	Alxa	12LS165	Tamusu	K-feldspar granite	103.52	40.76	272	0.9	LA-ICP-MS,U-Pb	Zircon	Shi Xingjun et al.	2014	Acta Petrologica et Mineralogica(ICWEA)
899	Outside of the MOO	Alxa	12LS152	Tamusu	Granodiorite	103.49	40.78	247	1.1	LA-ICP-MS,U-Pb	Zircon	Shi Xingjun et al.	2014	Acta Petrologica et Mineralogica(ICWEA)
900	Outside of the MOO	Alxa	12LS160	Tamusu	Diorite	103.49	40.78	249	1.1	LA-ICP-MS,U-Pb	Zircon	Shi Xingjun et al.	2014	Acta Petrologica et Mineralogica(ICWEA)
901	Outside of the MOO	Alxa	11LS170	Wuliqi	Monzogranite	104.48	40.81	254	2.0	LA-ICP-MS,U-Pb	Zircon	Shi Xingjun et al.	2014	Lithos
902	Outside of the MOO	Alxa	11LS96	Hurentaolegai	Granite	104.37	40.93	266	2.0	LA-ICP-MS,U-Pb	Zircon	Shi Xingjun et al.	2014	Lithos
903	Outside of the MOO	Alxa	11LS106	Baogeqi	Gabbro	104.23	40.93	264	3.0	LA-ICP-MS,U-Pb	Zircon	Shi Xingjun et al.	2014	Lithos
904	Outside of the MOO	Alxa	11LS122	Sharijimiao	Tonalite	104.46	40.96	252	2.0	LA-ICP-MS,U-Pb	Zircon	Shi Xingjun et al.	2014	Lithos
905	Outside of the MOO	Alxa	11LS91	Wenduermaodao	Granodiorite	104.40	40.98	254	2.0	LA-ICP-MS,U-Pb	Zircon	Shi Xingjun et al.	2014	Lithos
906	Outside of the MOO	Alxa	15ALS43		Diorite	101.42	39.60	287	2.0	LA-ICP-MS,U-Pb	Zircon	Song et al.	2018	Lithos
907	Outside of the MOO	Alxa	15ALS35		Dacite porphyry	101.09	39.64	295	2.0	LA-ICP-MS,U-Pb	Zircon	Song et al.	2018	Lithos
908	Outside of the MOO	Alxa	15ALS16		Granite	101.10	39.65	315	6.0	LA-ICP-MS,U-Pb	Zircon	Song et al.	2018	Lithos
909	Outside of the MOO	Alxa	15ALS04		Granite	100.08	39.67	296	3.0	LA-ICP-MS,U-Pb	Zircon	Song et al.	2018	Lithos
910	Outside of the MOO	Alxa	15ALS03		Granodiorite enclave	100.08	39.67	302	3.0	LA-ICP-MS,U-Pb	Zircon	Song et al.	2018	Lithos
911	Outside of the MOO	Alxa	15ALS05		Granodiorite	100.14	39.69	290	3.0	LA-ICP-MS,U-Pb	Zircon	Song et al.	2018	Lithos
912	Outside of the MOO	Alxa	15ALS06		Granite	100.14	39.69	291	3.0	LA-ICP-MS,U-Pb	Zircon	Song et al.	2018	Lithos
913	Outside of the MOO	Alxa	15ALS10		Gabbroic diorite enclave	100.14	39.69	293	4.0	LA-ICP-MS,U-Pb	Zircon	Song et al.	2018	Lithos
914	Outside of the MOO	Alxa	15ALS39		Diorite	100.84	39.69	295	2.0	LA-ICP-MS,U-Pb	Zircon	Song et al.	2018	Lithos
915	Outside of the MOO	Alxa	15ALS37		Granite	100.85	39.71	317	2.0	LA-ICP-MS,U-Pb	Zircon	Song et al.	2018	Lithos
916	Outside of the MOO	Alxa	KHB-1802	Khan-Bogd massif	Trachyrhyolite	107.79	43.13	331	1.0	TIMS,U-Pb	Zircon	Wang Wanqiong et al	2008	Stratigraphy&Geological Correlation
917	Outside of the MOO	Alxa	T5	De'rsidi from Urad zhongqi	Monzogranite	108.97	41.93	266	3.0	SHRIMP,U-Pb	Zircon	Wang Wanqiong et al	2012	Journal of Jilin University(Earth Science Edition)(ICWEA)

918	Outside of the MOO	Alxa	PM34-9-YQ1	Yabulaishan	Gabbro diorite	103.77	39.93	278	1.0	LA-ICP-MS,U-Pb	Zircon	Wang Xingjun	2012	China University of Geosciences, Beijing:Phd Dissertation
919	Outside of the MOO	Alxa	PM34-ZK-02-YQ1	Yabulaishan	Hornblende gabbro	103.77	39.93	278	1.0	LA-ICP-MS,U-Pb	Zircon	Wang Xingjun	2012	China University of Geosciences, Beijing:Phd Dissertation
920	Outside of the MOO	Alxa	PM35-9-YQ1	Yabulaishan	Hornblendite	103.77	39.93	278	1.0	LA-ICP-MS,U-Pb	Zircon	Wang Xingjun	2012	China University of Geosciences, Beijing:Phd Dissertation
921	Outside of the MOO	Alxa	DD2011-TW1	Yabulaishan	Gabbro	103.69	40.00	278	1.0	LA-ICP-MS,U-Pb	Zircon	Wang Xingjun	2012	China University of Geosciences, Beijing:Phd Dissertation
922	Outside of the MOO	Alxa	WD5012-TW1	Bijiertaiaobao	Gabbro	104.40	40.39	267	1.0	LA-ICP-MS,U-Pb	Zircon	Wang Xingjun	2012	China University of Geosciences, Beijing:Phd Dissertation
923	Outside of the MOO	Alxa	PM32-13-YQ1	bijiertai	Hornblende gabbro	104.53	40.43	267	1.0	LA-ICP-MS,U-Pb	Zircon	Wang Xingjun	2012	China University of Geosciences, Beijing:Phd Dissertation
924	Outside of the MOO	Alxa	PM32-14-YQ1	bijiertai	Gabbro	104.53	40.43	267	1.0	LA-ICP-MS,U-Pb	Zircon	Wang Xingjun	2012	China University of Geosciences, Beijing:Phd Dissertation
925	Outside of the MOO	Alxa	PM33-7-YQ1	bijiertai	Hornblendite	104.53	40.43	267	1.0	LA-ICP-MS,U-Pb	Zircon	Wang Xingjun	2012	China University of Geosciences, Beijing:Phd Dissertation
926	Outside of the MOO	Alxa	WD2011-TW1	Yabulaishan	Gabbro	103.96	40.98	248	0.8	LA-ICP-MS,U-Pb	Zircon	Wang Xingjun	2012	China University of Geosciences, Beijing:Phd Dissertation
927	Outside of the MOO	Alxa	T1	De'rsidi from Urad zhongqi	Monzogranite	108.97	41.93	279	3.0	SHRIMP,U-Pb	Zircon	Wang Xingjun	2012	Journal of Jilin University(Earth Science Edition)(ICWEA)
928	Outside of the MOO	Alxa	WD6011-TW1	Bijiertaiaobao	Amphibolite	104.54	40.37	286	1.7	LA-ICP-MS,U-Pb	Zircon	Wang Zengzhen et al.	2012	China University of Geosciences, Beijing:Phd Dissertation
929	Outside of the MOO	Alxa	12WLT-13-4	Langshan Shaerchulu	Granodiorite	106.86	41.25	273	1.6	LA-ICP-MS,U-Pb	Zircon	Wang Zengzhen et al.	2015	Journal of Asian Earth Sciences
930	Outside of the MOO	Alxa	12WLT-17-5	Langshan	Leuconorite	106.93	41.48	255	1.5	LA-ICP-MS,U-Pb	Zircon	Wang Zengzhen et al.	2015	Journal of Asian Earth Sciences
931	Outside of the MOO	Alxa	12WLT-18-1	Langshan Zhunshabaertai	Gabbro	106.98	41.63	320	1.9	LA-ICP-MS,U-Pb	Zircon	Wang Zengzhen et al.	2015	Journal of Asian Earth Sciences
932	Outside of the MOO	Alxa	12WLT-19-3	Langshan	Gabbro	107.01	41.63	328	2.1	LA-ICP-MS,U-Pb	Zircon	Wang Zengzhen et al.	2015	Journal of Asian Earth Sciences
933	Outside of the MOO	Alxa	NQ-1	Niuquanshan-Bayannuoergong	Granite	103.44	39.93	273	7.4	LA-ICP-MS,U-Pb	Zircon	Wu Kanglin	2011	Chang'an University:Master Dissertation
934	Outside of the MOO	Alxa	Z1	Zongnaishan	Tonalite	103.36	40.80	273	3.9	LA-ICP-MS,U-Pb	Zircon	Wu Kanglin	2011	Chang'an University:Master Dissertation
935	Outside of the MOO	Alxa	Z2	Zongnaishan	Tonalite	103.60	40.85	251		LA-ICP-MS,U-Pb	Zircon	Wu Kanglin	2011	Chang'an University:Master Dissertation
936	Outside of the MOO	Alxa	Z4	Zongnaishan	Tonalite	104.38	40.89	274	4.2	LA-ICP-MS,U-Pb	Zircon	Wu Kanglin	2011	Chang'an University:Master Dissertation
937	Outside of the MOO	Alxa	Z3	Zongnaishan	Tonalite	103.77	40.93	274	4.2	LA-ICP-MS,U-Pb	Zircon	Wu Kanglin	2011	Chang'an University:Master Dissertation
938	Outside of the MOO	Alxa	12WLT-08-1	Langshan	Gabbro–diorite	107.48	41.31	273	2.3	LA-ICP-MS,U-Pb	Zircon	Wu Kanglin	2015	Journal of Asian Earth Sciences
939	Outside of the MOO	Alxa	BY-01	Niuquanshan-Bayannuoergong	Granite	104.87	40.10	272	9.0	LA-ICP-MS,U-Pb	Zircon	Wu Yafei et al.	2011	Chang'an University:Master Dissertation
940	Outside of the MOO	Alxa	12ND-8	Dongshenmiao area	Monzogranite	107.05	41.13	259		LA-MC-ICP-MS	Zircon	Wu Yafei et al.	2014	Bulletin of Mineralogy,Petrology and Geochemistry(ICWEA)
941	Outside of the MOO	Alxa	Sh-05	Zongnaishan	Dioritie	103.52	40.76	268	1.4	LA-ICP-MS,U-Pb	Zircon	Xie Fenquan	2014	China University of Geosciences, Beijing:Master Dissertation
942	Outside of the MOO	Alxa	Sh-01	Zongnaishan	Granite	103.69	40.83	237	1.9	LA-ICP-MS,U-Pb	Zircon	Xie Fenquan	2014	China University of Geosciences, Beijing:Master Dissertation
943	Outside of the MOO	Alxa	Wh-12	Shalazhashan	Granodiorite	104.45	40.85	236	1.1	LA-ICP-MS,U-Pb	Zircon	Xie Fenquan	2014	China University of Geosciences, Beijing:Master Dissertation
944	Outside of the MOO	Alxa	Wh-09	Shalazhashan	Granite	104.35	40.92	249	0.8	LA-ICP-MS,U-Pb	Zircon	Xie Fenquan	2014	China University of Geosciences, Beijing:Master Dissertation
945	Outside of the MOO	Alxa	12ND-9	Dongshenmiao area	Monzogranite	107.06	41.13	259		LA-MC-ICP-MS	Zircon	Xie Fenquan	2014	Bulletin of Mineralogy,Petrology and Geochemistry(ICWEA)
946	Outside of the MOO	Alxa	Bh-01	Bayin Nur gong	Granite	104.91	40.01	252	1.0	LA-ICP-MS,U-Pb	Zircon	Xu Dongzhuo et al.	2014	China University of Geosciences, Beijing:Master Dissertation
947	Outside of the MOO	Alxa	WD2011	Wuliji	Gabbro	103.98	41.08	248	0.9	LA-ICP-MS,U-Pb	Zircon	Yang Qidi et al.	2014	Geological Bulletin of China(ICWEA)
948	Outside of the MOO	Alxa	11LS88	Shalazhashan	Biotite granodiorite	104.36	40.98	301	2.0	LA-ICP-MS,U-Pb	Zircon	Yarmolyuk V. V. et a	2014	Geological Bulletin of China(ICWEA)
949	Outside of the MOO	Alxa	12LS119	Yabulai Mountain	Syenogranite	103.07	39.64	285	1.2	LA-ICP-MS,U-Pb	Zircon	Ye Ke et al.	2016	Acta Petrologica et Mineralogica(ICWEA)
950	Outside of the MOO	Alxa	12LS103	Yabulai Mountain	Tonalite	103.69	39.95	272	1.0	LA-ICP-MS,U-Pb	Zircon	Ye Ke et al.	2016	Acta Petrologica et Mineralogica(ICWEA)
951	Outside of the MOO	Alxa	13AX39	Yabulai Mountain	Granodiorite	103.64	39.97	280	0.6	LA-ICP-MS,U-Pb	Zircon	Ye Ke et al.	2016	Acta Petrologica et Mineralogica(ICWEA)
952	Outside of the MOO	Alxa	PM044-11-YQ1	Hariaoribuge	Granodiorite	103.77	41.75	272	1.0	LA-ICP-MS,U-Pb	Zircon	Zeng Yong	2014	China University of Geosciences, Beijing:Master Dissertation
953	Outside of the MOO	Alxa	PH046-1-YQ1	Hariaoribuge	Monzodiorite	103.67	41.77	276	1.1	LA-ICP-MS,U-Pb	Zircon	Zeng Yong	2014	China University of Geosciences, Beijing:Master Dissertation
954	Outside of the MOO	Alxa	12LS146		Basic enclave	103.58	39.74	269	3.6	LA-ICP-MS,U-Pb	Zircon	Zhang Lei unpublished		
955	Outside of the MOO	Alxa	12LS138		Monzogranite	103.58	39.74	274	1.2	LA-ICP-MS,U-Pb	Zircon	Zhang Lei unpublished		
956	Outside of the MOO	Alxa	13AX84		Dacite	103.66	39.88	260	1.0	LA-ICP-MS,U-Pb	Zircon	Zhang Lei unpublished		
957	Outside of the MOO	Alxa	12LS80		Plagioclase granite	103.77	39.88	271	3.4	LA-ICP-MS,U-Pb	Zircon	Zhang Lei unpublished		
958	Outside of the MOO	Alxa	12LS87		Dioritic enclave	103.77	39.88	271	1.5	LA-ICP-MS,U-Pb	Zircon	Zhang Lei unpublished		
959	Outside of the MOO	Alxa	12LS104		Basic rock	103.67	39.92	274	1.4	LA-ICP-MS,U-Pb	Zircon	Zhang Lei unpublished		
960	Outside of the MOO	Alxa	LS095		Diabase	104.92	39.95	287		LA-ICP-MS,U-Pb	Zircon	Zhang Lei unpublished		
961	Outside of the MOO	Alxa	12LS31		Granodiorite	104.90	39.96	268	1.6	LA-ICP-MS,U-Pb	Zircon	Zhang Lei unpublished		
962	Outside of the MOO	Alxa	LS030		Migmatite	105.71	40.12	273	1.0	LA-ICP-MS,U-Pb	Zircon	Zhang Lei unpublished		
963	Outside of the MOO	Alxa	LS026		Granite porphyry	105.33	40.14	275	1.3	LA-ICP-MS,U-Pb	Zircon	Zhang Lei unpublished		
964	Outside of the MOO	Alxa	13AX21		Medium grained granite	104.17	40.16	269	1.0	LA-ICP-MS,U-Pb	Zircon	Zhang Lei unpublished		
965	Outside of the MOO	Alxa	12LS49		Granite	104.71	40.17	279	2.0	LA-ICP-MS,U-Pb	Zircon	Zhang Lei unpublished		
966	Outside of the MOO	Alxa	LS092		Granite	104.92	40.17	279	1.6	LA-ICP-MS,U-Pb	Zircon	Zhang Lei unpublished		
967	Outside of the MOO	Alxa	LS091		Gabbro	105.37	40.17	271	5.4	LA-ICP-MS,U-Pb	Zircon	Zhang Lei unpublished		
968	Outside of the MOO	Alxa	13AX01		K-feldspar granite	105.39	40.18	270	1.2	LA-ICP-MS,U-Pb	Zircon	Zhang Lei unpublished		
969	Outside of the MOO	Alxa	13AX15		Granite	105.39	40.19	270	4.5	LA-ICP-MS,U-Pb	Zircon	Zhang Lei unpublished		
970	Outside of the MOO	Alxa	13AX16		Granite	105.39	40.19	270	4.5	LA-ICP-MS,U-Pb	Zircon	Zhang Lei unpublished		
971	Outside of the MOO	Alxa	13AX17		Granite	105.39	40.19	270	4.5	LA-ICP-MS,U-Pb	Zircon	Zhang Lei unpublished		
972	Outside of the MOO	Alxa	13AX04		Gabbro	105.39	40.18	272	1.7	LA-ICP-MS,U-Pb	Zircon	Zhang Lei unpublished		
973	Outside of the MOO	Alxa	13AX07		Gabbro	105.39	40.18	272	1.7	LA-ICP-MS,U-Pb	Zircon	Zhang Lei unpublished		
974	Outside of the MOO	Alxa	13AX08		Gabbro	105.39	40.19	272	1.7	LA-ICP-MS,U-Pb	Zircon	Zhang Lei unpublished		
975	Outside of the MOO	Alxa	11LS41		Medium grained granite	105.51	40.20	271	1.0	LA-ICP-MS,U-Pb	Zircon	Zhang Lei unpublished		
976	Outside of the MOO	Alxa	11LS40		Coarse grained granite	105.52	40.20	271	0.5	LA-ICP-MS,U-Pb	Zircon	Zhang Lei unpublished		
977	Outside of the MOO	Alxa	11LS31		Monzogranite	105.25	40.21	273	2.0	LA-ICP-MS,U-Pb	Zircon	Zhang Lei unpublished		
978	Outside of the MOO	Alxa	LS054		Enclave	105.51	40.23	269	2.0	LA-ICP-MS,U-Pb	Zircon	Zhang Lei unpublished		
979	Outside of the MOO	Alxa	LS055		Enclave	105.51	40.23	270	0.8	LA-ICP-MS,U-Pb	Zircon	Zhang Lei unpublished		
980	Outside of the MOO	Alxa	LS104		Granite	104.77	40.24	267	2.2	LA-ICP-MS,U-Pb	Zircon	Zhang Lei unpublished		
981	Outside of the MOO	Alxa	12LS02		Fine-grained granite	105.46	40.38	276		LA-ICP-MS,U-Pb	Zircon	Zhang Lei unpublished		
982	Outside of the MOO	Alxa	12LS17		Monzogranite	105.44	40.42	287		LA-ICP-MS,U-Pb	Zircon	Zhang Lei unpublished		
983	Outside of the MOO	Alxa	LS087		Gabbro	105.37	40.44	275	1.4	LA-ICP-MS,U-Pb	Zircon	Zhang Lei unpublished		
984	Outside of the MOO	Alxa	LS004		K-feldspar granite	108.24	41.15	254		LA-ICP-MS,U-Pb	Zircon	Zhang Lei unpublished		
985	Outside of the MOO	Alxa	LS005		K-feldspar granite	108.24	41.15	254		LA-ICP-MS,U-Pb	Zircon	Zhang Lei unpublished		
986	Outside of the MOO	Alxa	LS012		Mixed granite	105.58	41.28	321	1.5	LA-ICP-MS,U-Pb	Zircon	Zhang Lei unpublished		
987	Outside of the MOO	Alxa	LS013		Mixed granite	105.58	41.28	321	1.5	LA-ICP-MS,U-Pb	Zircon	Zhang Lei unpublished		
988	Outside of the MOO	Alxa	WZ08	Wulatezhongqi	Granodiorite	108.84	41.68	291	4.0	SHRIMP,U-Pb	Zircon	Zhang Shuanhong et a	2009	International Journal of Earth Sciences
989	Outside of the MOO	Alxa	07223-1	Dahongshan South	Diorite	108.60	41.31	282	3.0	LA-ICP-MS,U-Pb	Zircon	Zhang Shuanhong et a	2010	Acta Petrologica et Mineralogica(ICWEA)
990	Outside of the MOO	Alxa	07215-1	Saiwusu South	Monzogranite	107.09	41.35	239	1.0	LA-ICP-MS,U-Pb	Zircon	Zhang Shuanhong et a	2010	Acta Petrologica et Mineralogica(ICWEA)
991	Outside of the MOO	Alxa	07189-1	Wulate Middle Banner	Quartz diorite	108.46	41.64	276	4.0	LA-ICP-MS,U-Pb	Zircon	Zhang Shuanhong et a	2010	Acta Petrologica et Mineralogica(ICWEA)
992	Outside of the MOO	Alxa	AB10-48	Huhetaoergai	Biotite granodiorite	101.66	42.02	356	3.0	SIMS,U-Pb	Zircon	Zhang W et al.	2017	Journal of Asian Earth Sciences
993	Outside of the MOO	Alxa	W08-170	Zhuxiaobuguhe	Granodiorite	101.58	42.02	286	2.0	SIMS,U-Pb	Zircon	Zhang W et al.	2017	Journal of Asian Earth Sciences
994	Outside of the MOO	Alxa	AYQ-25	Shazaoquan	Quartz diorite	100.50	40.02	302	9.2	LA-ICP-MS,U-Pb	Zircon	Zhang Wei et al.	2014	Geological Journal of China Universities(ICWEA)
995	Outside of the MOO	Alxa	W08-30	Wuliji-Chaganchulu	Granite	104.95	40.77	251	2.0	SIMS,U-Pb	Zircon	Zhang Wen et al.	2013	Scientia Sinica(Terrae)(ICWEA)

996	Outside of the MOO	Alxa	LS-8		Granite	106.32	40.62	329	1.5	LA-ICP-MS,U-Pb	Zircon	Zhang Wen et al.	2013	Scientia Sinica(Terrae)(ICWEA)
997	Outside of the MOO	Alxa	LS-11-32		Granite	105.03	39.42	359	4.1	LA-ICP-MS,U-Pb	Zircon	Zhang, et al.	2013	Scientia Sinica(Terrae)(ICWEA)
998	Outside of the MOO	Alxa	SL04-28	Beiqigetao pluton	Gabbro	108.09	41.53	269	8.0	SHRIMP,U-Pb	Zircon	Zhao Lei et al.	2011	Acta Petrologica Sinica(ICWEA)
999	Outside of the MOO	Alxa	AD51TW1	Salatoerhan	Granodiorite	104.43	40.83	233	5.8	TIMS,U-Pb	Zircon	Zhao Zelin et al.	2016	Geological Bulletin of China(ICWEA)
1000	Outside of the MOO	Alxa	AWD4TW1	Wuliji	Granite	104.44	40.87	228	0.4	TIMS,U-Pb	Zircon	Zhao Zelin et al.	2016	Geological Bulletin of China(ICWEA)
1001	Outside of the MOO	Alxa	AWD3TW1	Wuliji	Granodiorite	104.39	40.91	208	0.8	TIMS,U-Pb	Zircon	Zhao Zelin et al.	2016	Geological Bulletin of China(ICWEA)
1002	Outside of the MOO	Alxa	W08-276	Yagan	Granite	102.51	42.09	283	2.2	SIMS,U-Pb	Zircon	Zheng Rongguo et al.	2013	Acta Petrologica Sinica(ICWEA)
1003	Outside of the MOO	Alxa	AB10-27	Quagan Qulu ophiolite	Gabbro	104.88	40.73	275	3.0	SHRIMP,U-Pb	Zircon	Zheng Rongguo et al.	2014	Gondwana Research
1004	Outside of the MOO	Alxa	TL-26	Yingba Area	Pegmatite vein	105.60	41.63	146	1.6	LA-ICP-MS,U-Pb	Zircon	Zhou Yinzhang et al.	2012	Tectonophysics
1005	Outside of the MOO	Alxa	10L-100	Yingba Area	Gneissic granodiorite	105.60	41.64	325	1.6	LA-ICP-MS,U-Pb	Zircon	Zhou Yinzhang et al.	2014	Acta Geologica Sinica(ICWEA)
1006	Outside of the MOO	Beishan	PSS01	Pobei	Olivine gabbro	91.52	40.52	274	4.0	LA-ICP-MS,U-Pb	Zircon	Ao S J, et al.	2010	Gondwana Research.
1007	Outside of the MOO	Beishan	HSS08	Hongshishan	Olivine gabbro	92.03	40.92	282	2.6	LA-ICP-MS,U-Pb	Zircon	Ao S J, et al.	2010	Gondwana Research.
1008	Outside of the MOO	Beishan	D4314	Dahuoluonan	Granodiorite	96.71	41.46	260	1.5	LA-ICP-MS,U-Pb	Zircon	Bu T, et al.	2019	Geological bulletin of China (ICWEA).
1009	Outside of the MOO	Beishan	JDTW2	SuanJingZiYiNa（HongLiuYu	spilite	97.76	41.38	360	1.4	LA-ICP-MS,U-Pb	Zircon	Chen C, et al.	2017	Acta Sedimentologica Sinica (ICWEA).
1010	Outside of the MOO	Beishan	JZ2-2	Jinwozi	Granodiorite	95.76	41.25	359		TIMS,U-Pb	Zircon	Chen F W, et al.	1999	Geological Review (ICWEA).
1011	Outside of the MOO	Beishan	TW1024-1	Baiheshan	Quartzdiorite	98.15	42.23	351	1.5	LA-ICP-MS,U-Pb	Zircon	Duan L F, et al.	2020	Geological bulletin of China (ICWEA).
1012	Outside of the MOO	Beishan	BS41	Jiujing	Monzogranite	97.58	41.11	270		LA-ICP-MS,U-Pb	Zircon	Fan H.H. et al.	2006	Uranium Geology (ICWEA).
1013	Outside of the MOO	Beishan		Shibanjing	Plagiogranite	98.37	41.74	338	1.1	LA-ICP-MS,U-Pb	Zircon	Fu Y G, et al.	2020	Acta Geologica Sinica (English Edition).
1014	Outside of the MOO	Beishan		shibanjing	plagiogranite	98.37	41.74	338	1.1	LA-ICP-MS,U-Pb	Zircon	Fu Y G, et al.	2020	Acta Geologica Sinica (English Edition).
1015	Outside of the MOO	Beishan	B-27/1149	Zamyn Belgekh Pluton	leucogranite	98.36	43.08	299	8.0	LA-ICP-MS,U-Pb	Zircon	Hanzl P, et al.	2008	Journal of Geosciences.
1016	Outside of the MOO	Beishan	A-26/1018	Zamyn Belgekh Pluton	biotite-hornblende granodiorite	96.56	43.14	288	15.0	LA-ICP-MS,U-Pb	Zircon	Hanzl P, et al.	2008	Journal of Geosciences.
1017	Outside of the MOO	Beishan	E-27/1138	Zamyn Belgekh Pluton	Monzonite	97.15	43.28	326	33.0	LA-ICP-MS,U-Pb	Zircon	Hanzl P, et al.	2008	Journal of Geosciences.
1018	Outside of the MOO	Beishan	C-28/1028	The Trans-Altay Intrusive Com	Porphyritic granite	97.36	43.28	333	18.0	LA-ICP-MS,U-Pb	Zircon	Hanzl P, et al.	2008	Journal of Geosciences.
1019	Outside of the MOO	Beishan	TW6104	Fengleishan	Monzogranite	99.09	41.94	320	1.0	LA-ICP-MS,U-Pb	Zircon	Hao Z Y, et al.	2019	Geology in China (ICWEA).
1020	Outside of the MOO	Beishan	501/1tw	Hongliuhe	Granodiorite	94.73	41.53	257		TIMS,U-Pb	Zircon	Hu A Q, et al.	1997	Geological evolution and metallogenic law in Northern Xinjiang.
1021	Outside of the MOO	Beishan	H1	Hongliuhedaqiaonan	Diabase	94.67	41.56	222		TIMS,U-Pb	Zircon	Hu A Q, et al.	1997	Geological evolution and metallogenic law in Northern Xinjiang.
1022	Outside of the MOO	Beishan	1830-3	Hexidongdanyuan	Monzogranite	94.61	41.59	257		TIMS,U-Pb	Zircon	Hu A Q, et al.	1997	Geological evolution and metallogenic law in Northern Xinjiang.
1023	Outside of the MOO	Beishan	1831-2	Hexizhan	Granite	94.61	41.59	257		TIMS,U-Pb	Zircon	Hu A Q, et al.	1997	Geological evolution and metallogenic law in Northern Xinjiang.
1024	Outside of the MOO	Beishan	1503/1-2(?)	Tianhuyidanyuan	Monzogranite	94.49	41.63	222		TIMS,U-Pb	Zircon	Hu A Q, et al.	1997	Geological evolution and metallogenic law in Northern Xinjiang.
1025	Outside of the MOO	Beishan	GPG-9	Gongpoquan	Granodiorite	97.30	41.48	340		SHRIMP,U-Pb	Zircon	Jiang S H, et al.	2002	Earth and Environment (ICWEA).
1026	Outside of the MOO	Beishan		HongLiuDaQuan	monzogranite	98.62	40.51	267	3.7	LA-ICP-MS,U-Pb	Zircon	Jin Y H, et al.	2020	Mineral exploration (ICWEA).
1027	Outside of the MOO	Beishan		HongLiuDaQuan	diorite	98.62	40.51	282	3.7	LA-ICP-MS,U-Pb	Zircon	Jin Y H, et al.	2020	Mineral exploration (ICWEA).
1028	Outside of the MOO	Beishan		Hongliudaquan	Monzogranite	98.62	40.51	267	3.7	LA-ICP-MS,U-Pb	Zircon	Jin Y H, et al.	2020	Mineral exploration (ICWEA).
1029	Outside of the MOO	Beishan		Hongliudaquan	Diorite	98.62	40.51	282	3.7	LA-ICP-MS,U-Pb	Zircon	Jin Y H, et al.	2020	Mineral exploration (ICWEA).
1030	Outside of the MOO	Beishan	YUM-10/1	Tavan-Takhil-Ula Massif	Granodiorite	97.33	43.15	302	3.0	TIMS,U-Pb	Zircon	Kozakov I K, et al.	2004	Geodynamic Evolution of the Central Asian Foldbelt
1031	Outside of the MOO	Beishan	28-5325	Gobi-Tienshan pluton	Granodiorite	97.27	43.15	301	1.0	Pb-Pb	Zircon	Kozakov I K, et al.	2005	Geodynamic Evolution of the Central Asian Foldbelt
1032	Outside of the MOO	Beishan	M33/06	Gobi-Tienshan pluton	Granodiorite	96.85	43.20	300	2.0	SHRIMP,U-Pb	Zircon	Kozakov I K, et al.	2005	Geodynamic Evolution of the Central Asian Foldbelt
1033	Outside of the MOO	Beishan	MS3- 1	Mazhuangshan	Quartz porphyry	95.88	41.32	309		TIMS,U-Pb	Zircon	Li H Q, et al.	1999	Chinese Journal of Geology (ICWEA).
1034	Outside of the MOO	Beishan	MS1- 5	Mazhuangshan	Rhyolitic porphyry	95.88	41.35	301		TIMS,U-Pb	Zircon	Li H Q, et al.	1999	Chinese Journal of Geology (ICWEA).
1035	Outside of the MOO	Beishan		Baishan	Bi-plagiogranite	95.92	42.52	181	3.0	TIMS,U-Pb	Zircon	Li H Q, et al.	2005	Acta Geologica Sinica (ICWEA).
1036	Outside of the MOO	Beishan	D2141-1	Baishan	Bi-plagiogranite	95.98	42.51	239	8.0	SHRIMP,U-Pb	Zircon	Li H Q, et al.	2006	Geological Bulletin of China (ICWEA).
1037	Outside of the MOO	Beishan		Baishan	plagiogranite	95.93	42.52	236	6.0	TIMS,U-Pb	Zircon	Li H Q, et al.	2006	Geological Bulletin of China (ICWEA).
1038	Outside of the MOO	Beishan	TW2	Yuanbaoshanfushiyanji	Monzogranite	99.63	42.38	315	2.7	LA-ICP-MS,U-Pb	Zircon	Li J, et al.	2019	Acta Petrologica et Mineralogica(ICWEA).
1039	Outside of the MOO	Beishan	P2RZ1	Yuanbaoshanfushiyanji	Granodiorite	99.52	42.44	317	3.1	LA-ICP-MS,U-Pb	Zircon	Li J, et al.	2019	Acta Petrologica et Mineralogica(ICWEA).
1040	Outside of the MOO	Beishan	P2RZ2	Yuanbaoshanfushiyanji	Trondhjemite	99.58	42.50	314	2.3	LA-ICP-MS,U-Pb	Zircon	Li J, et al.	2019	Acta Petrologica et Mineralogica(ICWEA).
1041	Outside of the MOO	Beishan	14TW20	Lishitandiqu	Monzogranite	98.97	42.05	309	1.4	LA-ICP-MS,U-Pb	Zircon	Li M, et al.	2018	Earth science (ICWEA).
1042	Outside of the MOO	Beishan	06TW27	Lishitandiqu	Granodiorite	98.51	42.08	306	1.2	LA-ICP-MS,U-Pb	Zircon	Li M, et al.	2018	Earth science (ICWEA).
1043	Outside of the MOO	Beishan	02TW1	Lishitandiqu	Tonalite	98.96	42.09	311	1.4	LA-ICP-MS,U-Pb	Zircon	Li M, et al.	2018	Earth science (ICWEA).
1044	Outside of the MOO	Beishan	TW2005	Neimengguhazhu	Granodiorite	98.96	42.15	299	1.7	LA-ICP-MS,U-Pb	Zircon	Li M, et al.	2019	Earth science (ICWEA).
1045	Outside of the MOO	Beishan	01TW32	Neimengguhazhu	Alkali feldspar granite	99.96	42.15	289	1.3	LA-ICP-MS,U-Pb	Zircon	Li M, et al.	2019	Earth science (ICWEA).
1046	Outside of the MOO	Beishan	23TW15	Neimengguhazhu	Monzogranite	99.97	42.23	306	1.3	LA-ICP-MS,U-Pb	Zircon	Li M, et al.	2019	Earth science (ICWEA).
1047	Outside of the MOO	Beishan	16TW10	Lishitandiqu	Granodiorite	98.97	42.09	310	1.4	LA-ICP-MS,U-Pb	Zircon	Li M, et al.	2020	Earth science (ICWEA).
1048	Outside of the MOO	Beishan	02TW32	Hazhu	Granodiorite	98.93	42.13	262	1.1	LA-ICP-MS,U-Pb	Zircon	Li M, et al.	2020	Canadian Journal of Earth Sciences.
1049	Outside of the MOO	Beishan	02TW31	Hazhu	Granodiorite	98.93	42.13	264	1.2	LA-ICP-MS,U-Pb	Zircon	Li M, et al.	2020	Canadian Journal of Earth Sciences.
1050	Outside of the MOO	Beishan	02TW19	Hazhu	Monzogranite	98.93	42.13	270	1.1	LA-ICP-MS,U-Pb	Zircon	Li M, et al.	2020	Canadian Journal of Earth Sciences.
1051	Outside of the MOO	Beishan	02TW24	Hazhu	Monzogranite	98.93	42.13	277	1.2	LA-ICP-MS,U-Pb	Zircon	Li M, et al.	2020	Canadian Journal of Earth Sciences.
1052	Outside of the MOO	Beishan	MSD09821-6.1		Granite	94.87	41.13	224	1.0	LA-ICP-MS,U-Pb	Zircon	Li Shan et al.	2012	Lithos.
1053	Outside of the MOO	Beishan	HNS09823-2.2		Granite	95.50	41.20	217	2.0	LA-ICP-MS,U-Pb	Zircon	Li Shan et al.	2012	Lithos.
1054	Outside of the MOO	Beishan	B70822-4		Granite	95.52	41.18	221	3.0	LA-ICP-MS,U-Pb	Zircon	Li Shan et al.	2012	Lithos.
1055	Outside of the MOO	Beishan	B80625-6		Quartz-syenite	95.37	41.23	221	2.0	LA-ICP-MS,U-Pb	Zircon	Li Shan et al.	2012	Lithos.
1056	Outside of the MOO	Beishan	B80628-3		Granite	95.77	41.23	222	2.0	LA-ICP-MS,U-Pb	Zircon	Li Shan et al.	2012	Lithos.
1057	Outside of the MOO	Beishan	DQ09819-8		Quartz-syenite	95.35	41.27	225	1.0	LA-ICP-MS,U-Pb	Zircon	Li Shan et al.	2012	Lithos.
1058	Outside of the MOO	Beishan	B70817-2.1		Granite	96.72	41.45	238	1.0	LA-ICP-MS,U-Pb	Zircon	Li Shan et al.	2012	Lithos.
1059	Outside of the MOO	Beishan	B70817-2.3		Granite	96.72	41.45	240	2.0	LA-ICP-MS,U-Pb	Zircon	Li Shan et al.	2012	Lithos.
1060	Outside of the MOO	Beishan	DDQ09824-1.1	Dongdaquan	Syenogranie	96.09	41.20	279	1.1	LA-ICP-MS,U-Pb	Zircon	Li Shan et al.	2013	Journal of Asian Earth Sciences.
1061	Outside of the MOO	Beishan	DDQ09824-2.1	Dongdaquan	Mylonitic granodiorite	96.16	41.21	279	1.0	LA-ICP-MS,U-Pb	Zircon	Li Shan et al.	2013	Journal of Asian Earth Sciences.
1062	Outside of the MOO	Beishan	DDQ09824-3	Dongdaquan	Gneissic monzogranite	96.18	41.20	277	2.0	LA-ICP-MS,U-Pb	Zircon	Li Shan et al.	2013	Journal of Asian Earth Sciences.
1063	Outside of the MOO	Beishan	B80628-8.2	Wufengshan	Syenogranie	95.90	41.25	275	3.0	LA-ICP-MS,U-Pb	Zircon	Li Shan et al.	2013	Journal of Asian Earth Sciences.
1064	Outside of the MOO	Beishan	LY-9-18-1	LiuYuanXi	lamprophyre	95.42	41.13	230		LA-ICP-MS,U-Pb	Zircon	Liu C, et al.	2006	Acta Petrologica Sinica (ICWEA).
1065	Outside of the MOO	Beishan	LY-9-19-1	LiuYuanXi	lamprophyre	95.42	41.13	230		LA-ICP-MS,U-Pb	Zircon	Liu C, et al.	2006	Acta Petrologica Sinica (ICWEA).
1066	Outside of the MOO	Beishan	LY-9-19-2	Liuyuanxi	Lamprophyre	95.42	41.13	230		TIMS,U-Pb	Zircon	Liu C, et al.	2006	Acta Petrologica Sinica (ICWEA).
1067	Outside of the MOO	Beishan		Maanshanbei	Granite	96.21	41.65	238	4.3	U-Pb	Zircon	Liu M Q, et al.	2006	Acta Petrologica et Mineralogica (ICWEA).
1068	Outside of the MOO	Beishan	Rh(01)Nd3	Quedongshan	Monzogranite	96.73	42.45	286		LA-ICP-MS,U-Pb	Zircon	Liu M Q, et al.	2018	Geological Bulletin of China (ICWEA).
1069	Outside of the MOO	Beishan	Rh(01)Nd1	Maanshanbei	Hb-Bi-Monzogranite	96.28	42.70	238		LA-ICP-MS,U-Pb	Zircon	Liu M Q, et al.	2018	Geological Bulletin of China (ICWEA).
1070	Outside of the MOO	Beishan	M-5	DaShanTouNaYanTi	diorite	95.48	41.32	357	0.9	LA-ICP-MS,U-Pb	Zircon	Ma L.2017	2017	Lanzhou University, Lanzhou.
1071	Outside of the MOO	Beishan		Huolezhadegaihuanzhuangyant	Granite	96.25	42.58	237	2.0	LA-ICP-MS,U-Pb	Zircon	Mu K.	2019	Xian:Chang`an University.
1072	Outside of the MOO	Beishan		HuoLeZhaDeGaiHuanZhuangY	Porphyritic granite	96.25	42.58	237	2.0	LA-ICP-MS,U-Pb	Zircon	Mu K.	2019	Xian:Chang`an University.
1073	Outside of the MOO	Beishan		Huolezhadegaihuanzhuangyant	Granite	96.25	42.58	237	2.0	LA-ICP-MS,U-Pb	Zircon	Mu K.	2019	Xian:Chang`an University.

1074	Outside of the MOO	Beishan	HNS99-5	Huaniushan	K-spar granite	95.67	41.13	194		40Ar/43Ar	Biotite	Nie F J, et al.	2002	Chinese Journal of Geology (ICWEA).
1075	Outside of the MOO	Beishan	NJS01-B1	Mingshui	Monzogranite	97.13	42.16	218		40Ar/42Ar	Biotite	Nie F J, et al.	2002	Earth and Environment (ICWEA).
1076	Outside of the MOO	Beishan	LSS8-3	Liushashan	Granodiorite	98.41	42.28	260		40Ar/39Ar	Biotite	Nie F J, et al.	2002	Earth and Environment (ICWEA).
1077	Outside of the MOO	Beishan	3PgTW-2	Dashishan	Hb-Granite	95.67	42.30	273		LA-ICP-MS,U-Pb	Zircon	Qi R R, et al.	2006	Acta Petrologica et Mineralogica (ICWEA).
1078	Outside of the MOO	Beishan	MEC2-1	Juyanhai	Bi- syenogranite	100.06	41.44	312	1.0	LA-ICP-MS,U-Pb	Zircon	Song B, et al.	2020	Acta Geoscientica Sinica (ICWEA).
1079	Outside of the MOO	Beishan	MEC2-2	Juyanhai	Bi-monzogranite	100.06	41.44	315	2.0	LA-ICP-MS,U-Pb	Zircon	Song B, et al.	2020	Acta Geoscientica Sinica (ICWEA).
1080	Outside of the MOO	Beishan	BJS-14		Diabase	92.05	40.91	280	4.8	LA-ICP-MS,U-Pb	Zircon	Su B X, et al.	2011	Journal of Asian Earth Sciences.
1081	Outside of the MOO	Beishan	LD-δ		Diorite	92.05	40.91	280	4.8	LA-ICP-MS,U-Pb	Zircon	Su B X, et al.	2011	Journal of Asian Earth Sciences.
1082	Outside of the MOO	Beishan	09HSS3-4		Dacite	92.02	40.92	265		LA-ICP-MS,U-Pb	Zircon	Su B X, et al.	2011	Journal of Asian Earth Sciences.
1083	Outside of the MOO	Beishan	HSS12	Hongshishan	Silicified diorite	92.02	40.91	280	4.8	LA-ICP-MS,U-Pb	Zircon	Su B X, et al.	2011	Journal of Asian Earth Sciences.
1084	Outside of the MOO	Beishan	HSS6-1	Hongshishan	Rhyolite	92.02	40.95	279	2.9	LA-ICP-MS,U-Pb	Zircon	Su B X, et al.	2011	Journal of Asian Earth Sciences.
1085	Outside of the MOO	Beishan	TW6241-1	Gansuliuyuan	syenogranite	95.82	41.21	289	1.4	LA-ICP-MS,U-Pb	Zircon	Sun H R, et al.	2020	Journal of JiLin University (Earth Science Edition)(ICWEA).
1086	Outside of the MOO	Beishan	X-466	XianShuiQuan	Gneissic granite	95.92	42.20	254		TIMS,U-Pb	Zircon	Tang J H, et al.	2007	Acta Petrologica Sinica (ICWEA).
1087	Outside of the MOO	Beishan	X-468	XianShuiQuan	Gneissic granite	95.92	42.20	254		TIMS,U-Pb	Zircon	Tang J H, et al.	2007	Acta Petrologica Sinica (ICWEA).
1088	Outside of the MOO	Beishan	X-469	XianShuiQuan	Gneissic granite	95.92	42.20	254		TIMS,U-Pb	Zircon	Tang J H, et al.	2007	Acta Petrologica Sinica (ICWEA).
1089	Outside of the MOO	Beishan	X-471	XianShuiQuan	Gneissic granite	95.92	42.20	254		TIMS,U-Pb	Zircon	Tang J H, et al.	2007	Acta Petrologica Sinica (ICWEA).
1090	Outside of the MOO	Beishan	X-472	XianShuiQuan	Gneissic granite	95.92	42.20	254		TIMS,U-Pb	Zircon	Tang J H, et al.	2007	Acta Petrologica Sinica (ICWEA).
1091	Outside of the MOO	Beishan	X-473	XianShuiQuan	Gneissic granite	95.92	42.20	254		TIMS,U-Pb	Zircon	Tang J H, et al.	2007	Acta Petrologica Sinica (ICWEA).
1092	Outside of the MOO	Beishan	X643	Xianshuiquan	Gneissic granite	95.87	42.60	254	10.0	TIMS,U-Pb	Zircon	Tang J H, et al.	2007	Acta Petrologica Sinica (ICWEA).
1093	Outside of the MOO	Beishan	X-415	Huangshannan	Mus.-granite	95.89	42.57	260	1.4	LA-ICP-MS,U-Pb	Zircon	Tang J H, et al.	2008	Acta Petrologica Sinica (ICWEA).
1094	Outside of the MOO	Beishan	X-528	Tuladonggengou	Two-mica granite	95.89	42.57	275	8.3	LA-ICP-MS,U-Pb	Zircon	Tang J H, et al.	2008	Acta Petrologica Sinica (ICWEA).
1095	Outside of the MOO	Beishan	B70820-2.1	Jinmiaogoujinkuangnan	Gneissic granite	97.32	40.53	270		LA-ICP-MS,U-Pb	Zircon	This study		
1096	Outside of the MOO	Beishan	B70818-9.1	Yinaoxia	K-spar granite	96.53	41.08	280		LA-ICP-MS,U-Pb	Zircon	This study		
1097	Outside of the MOO	Beishan	B70818-5.2	Tongchangkouyinan	K-spar granite	96.56	41.16	280		LA-ICP-MS,U-Pb	Zircon	This study		
1098	Outside of the MOO	Beishan	B70822-8	Huaniushanbei	Granodiorite	95.33	41.18	224		LA-ICP-MS,U-Pb	Zircon	This study		
1099	Outside of the MOO	Beishan	B70817-8.2	Tongchangkoubei	Granodiorite	96.59	41.32	280		LA-ICP-MS,U-Pb	Zircon	This study		
1100	Outside of the MOO	Beishan	B70815-11.1	Tiaohunan	Plagiogranite	96.46	41.52	280		LA-ICP-MS,U-Pb	Zircon	This study		
1101	Outside of the MOO	Beishan	B70815-4.8	Choushuijingxi	Pyroxenite	96.21	42.01	300		LA-ICP-MS,U-Pb	Zircon	This study		
1102	Outside of the MOO	Beishan	B70815-9.2	Chaganchunzijingbeixi	Monzogranite	96.35	42.05	280		LA-ICP-MS,U-Pb	Zircon	This study		
1103	Outside of the MOO	Beishan	B70814-4.2	Gongpoquanyihaokuangquxice	Diorite	97.26	42.10	270		LA-ICP-MS,U-Pb	Zircon	This study		
1104	Outside of the MOO	Beishan	B70814-1	Shuangjingzixi	Granodiorite	97.70	42.62	270		LA-ICP-MS,U-Pb	Zircon	This study		
1105	Outside of the MOO	Beishan	YF15-2	Gansumazhuangshan	K-spar granite	95.88	41.32	318	1.0	LA-ICP-MS,U-Pb	Zircon	Wang Q S, et al.	2020	Acta Petrologica Sinica (ICWEA).
1106	Outside of the MOO	Beishan	MZS7-6	Gansumazhuangshan	Granodiorite	95.88	41.35	320	0.8	LA-ICP-MS,U-Pb	Zircon	Wang Q S, et al.	2020	Acta Petrologica Sinica (ICWEA).
1107	Outside of the MOO	Beishan	NJ-26A	NiuQuanZiSheLuHunZaYan	gabbro	96.75	41.60	354	3.3	LA-ICP-MS,U-Pb	Zircon	Wang S D.	2017	Wuhan: China University of Geosciences, Wuhan.
1108	Outside of the MOO	Beishan	H2-2	Mazongshanyemajie	Plagiogranite	97.04	41.51	311		TIMS,U-Pb	Zircon	Wang Y B.	1994	Geological Bulletin of China (ICWEA).
1109	Outside of the MOO	Beishan	642-1	LangWaShan	Granodiorite	97.08	42.17	278		LA-ICP-MS,U-Pb	Zircon	Wang Y B.	1994	Geological Bulletin of China (ICWEA).
1110	Outside of the MOO	Beishan	BSQ-1	Baishiquan	Gabbro	95.00	41.95	284	8.0	TIMS,U-Pb	Zircon	Wu H, et al.	2005	Acta Geologica Sinica (ICWEA).
1111	Outside of the MOO	Beishan	BSQ-3	Baishiquan	Quartz diorite	95.00	41.95	285	10.0	TIMS,U-Pb	Zircon	Wu H, et al.	2005	Acta Geologica Sinica (ICWEA).
1112	Outside of the MOO	Beishan	GHSN-01	HeiShanTongNieKuangChuang	gabbro	95.99	41.39	356	0.6	ID-TIMS,U-Pb	Zircon	Xie W, et al.	2012	Lithos.
1113	Outside of the MOO	Beishan	GHCN-01	HeiShanTongNieKuangChuang	gabbro	95.99	41.39	358	5.0	SHRIMP,U-Pb	Zircon	Xie W, et al.	2012	Lithos.
1114	Outside of the MOO	Beishan	GHCN-02	HeiShanTongNieKuangChuang	gabbro	95.99	41.39	357	4.0	SHRIMP,U-Pb	Zircon	Xie W, et al.	2012	Lithos.
1115	Outside of the MOO	Beishan		HongLiuHe	Miscellaneous rock mass	95.50	41.35	359	5.7	SHRIMP,U-Pb	Zircon	Yan H Q, et al.	2012	Northwestern Geology (ICWEA).
1116	Outside of the MOO	Beishan	GS	GuaiShiShanChaoJiXingZaYan	gabbro	96.23	41.50	359	3.9	SHRIMP,U-Pb	Zircon	Yang J G, et al.	2016	Geotectonica et Metallogenia (ICWEA).
1117	Outside of the MOO	Beishan	PM501Rz-6	Xiaohongshannan	Granodiorite	97.24	42.41	305	1.9	LA-ICP-MS,U-Pb	Zircon	Yang W B, et al.	2020	Geological bulletin of China (ICWEA).
1118	Outside of the MOO	Beishan	D5300Rz-1	Xiaohongshannan	Granodiorite	97.25	42.41	316	1.6	LA-ICP-MS,U-Pb	Zircon	Yang W B, et al.	2020	Geological bulletin of China (ICWEA).
1119	Outside of the MOO	Beishan	DQ-4	DongQiYiShanHuaGangYanTi	Biotite monzogranite	99.65	41.42	355	5.0	SHRIMP,U-Pb	Zircon	Yang Y Q, et al.	2013	Acta Geoscientica Sinica (ICWEA).
1120	Outside of the MOO	Beishan	DQ-2	DongQiYiShanHuaGangYanTi	Medium-grained gneiss biotite mon	99.73	41.45	359	4.0	SHRIMP,U-Pb	Zircon	Yang Y Q, et al.	2013	Acta Geoscientica Sinica (ICWEA).
1121	Outside of the MOO	Beishan	DQ-1	DongQiYiShanHuaGangYanTi	Medium grained two-mica granite	99.75	41.47	355	4.0	SHRIMP,U-Pb	Zircon	Yang Y Q, et al.	2013	Acta Geoscientica Sinica (ICWEA).
1122	Outside of the MOO	Beishan	141068	Lebaquan-niujuanzi	English cloud diorite	97.02	41.55	360	3.0	LA-ICP-MS,U-Pb	Zircon	Yu J Y, et al.	2016	Lithos.
1123	Outside of the MOO	Beishan	132053	Lebaquan-niujuanzi	Pale granite	96.77	41.65	359	5.0	LA-ICP-MS,U-Pb	Zircon	Yu J Y, et al.	2016	Lithos.
1124	Outside of the MOO	Beishan	16BS047	Chijinxia	Quartz monzonite	97.41	40.15	273	2.0	LA-ICP-MS,U-Pb	Zircon	Yuan Y.	2019	Wuhan: China University of Geosciences, Wuhan.
1125	Outside of the MOO	Beishan	16BS052	Chijinxia	Mafic enclave	97.41	40.15	279	1.0	LA-ICP-MS,U-Pb	Zircon	Yuan Y.	2019	Wuhan: China University of Geosciences, Wuhan.
1126	Outside of the MOO	Beishan	16BS055	Chijinxia	Granodiorite	97.41	40.15	296	3.0	LA-ICP-MS,U-Pb	Zircon	Yuan Y.	2019	Wuhan: China University of Geosciences, Wuhan.
1127	Outside of the MOO	Beishan	16BS056	Qingshannongchang	Diorite	97.36	40.44	274	2.0	LA-ICP-MS,U-Pb	Zircon	Yuan Y.	2019	Wuhan: China University of Geosciences, Wuhan.
1128	Outside of the MOO	Beishan	16BS058-2	Qingshannongchang	Mafic enclave	97.36	40.44	274	2.0	LA-ICP-MS,U-Pb	Zircon	Yuan Y.	2019	Wuhan: China University of Geosciences, Wuhan.
1129	Outside of the MOO	Beishan	16BS098	Qiaowanfushiyanti	Diorite	96.74	40.57	288	2.0	LA-ICP-MS,U-Pb	Zircon	Yuan Y.	2019	Wuhan: China University of Geosciences, Wuhan.
1130	Outside of the MOO	Beishan	X10-46-1	Shuangjingzi	Quartzdiorite	97.12	42.11	281	1.0	LA-ICP-MS,U-Pb	Zircon	Yuan Y.	2019	Wuhan: China University of Geosciences, Wuhan.
1131	Outside of the MOO	Beishan	X10-47-1	Shuangjingzi	Quartzdiorite	97.12	42.11	287	1.0	LA-ICP-MS,U-Pb	Zircon	Yuan Y.	2019	Wuhan: China University of Geosciences, Wuhan.
1132	Outside of the MOO	Beishan	X10-45-1	Shuangjingzi	Dacite	97.12	42.11	325	1.0	LA-ICP-MS,U-Pb	Zircon	Yuan Y.	2019	Wuhan: China University of Geosciences, Wuhan.
1133	Outside of the MOO	Beishan	X10-44-1	Shuangjingzi	Granodiorite	97.12	42.11	330	2.0	LA-ICP-MS,U-Pb	Zircon	Yuan Y.	2019	Wuhan: China University of Geosciences, Wuhan.
1134	Outside of the MOO	Beishan	AB09-103	Qiaowan Pluton	Monzogranite	96.69	40.70	285	3.7	LA-ICP-MS,U-Pb	Zircon	Zhang W, et al.	2012	Journal of Asian Earth Sciences.
1135	Outside of the MOO	Beishan	AB09-101	Bandaoshan Pluton	monzogranite	99.46	41.03	285	3.7	LA-ICP-MS,U-Pb	Zircon	Zhang W, et al.	2012	Journal of Asian Earth Sciences.
1136	Outside of the MOO	Beishan	AB09-110	In'Aoxia Pluton	Granodiorite	96.89	41.05	285	3.7	LA-ICP-MS,U-Pb	Zircon	Zhang W, et al.	2012	Journal of Asian Earth Sciences.
1137	Outside of the MOO	Beishan	W08-295	DongQiYiShanHuaGangYanTi	granite	99.73	41.47	356	2.0	SIMS,U-Pb	Zircon	Zhang W, et al.	2012	Lithos.
1138	Outside of the MOO	Beishan	040k13-807	Kumutageshalongdong	Monzogranite	93.25	41.47	293	6.0	SHRIMP,U-Pb	Zircon	Zhang X M, et al.	2006	Geological Bulletin of China (ICWEA).
1139	Outside of the MOO	Beishan	LY2-27	LiuYuan	Gabbro	95.25	41.01	272	2.4	SHRIMP,U-Pb	Zircon	Zhang Y Y, et al.	2011	Gondwana Research.
1140	Outside of the MOO	Beishan	LY2-30	LiuYuan	Gabbro	95.25	41.01	272	2.4	SHRIMP,U-Pb	Zircon	Zhang Y Y, et al.	2011	Gondwana Research.
1141	Outside of the MOO	Beishan	LY15-7-3	LiuYuan	Diorite	95.25	41.01	273	4.4	SHRIMP,U-Pb	Zircon	Zhang Y Y, et al.	2011	Gondwana Research.
1142	Outside of the MOO	Beishan	05LY18-25		Basalt	95.25	41.01	280		SHRIMP,U-Pb	Zircon	Zhang Y Y, et al.	2011	Gondwana Research.
1143	Outside of the MOO	Beishan	LY15-7	LiuYuan	Diorite	95.25	41.01	291	4.9	SHRIMP,U-Pb	Zircon	Zhang Y Y, et al.	2011	Gondwana Research.
1144	Outside of the MOO	Beishan	LY9-18-1	LiuYuan	mafic/ultramafic rock	96.33	41.05	230		SHRIMP,U-Pb	Zircon	Zhang Y Y, et al.	2011	Gondwana Research.
1145	Outside of the MOO	Beishan	LY9-19-1		mafic/ultramafic rock	96.33	41.05	230		SHRIMP,U-Pb	Zircon	Zhang Y Y, et al.	2011	Gondwana Research.
1146	Outside of the MOO	Beishan	LY9-19-2		mafic/ultramafic rock	96.33	41.05	230		SHRIMP,U-Pb	Zircon	Zhang Y Y, et al.	2011	Gondwana Research.
1147	Outside of the MOO	Beishan	LY-11-7	LiuYuan	mafic/ultramafic rock	96.33	41.05	250		SHRIMP,U-Pb	Zircon	Zhang Y Y, et al.	2011	Gondwana Research.
1148	Outside of the MOO	Beishan	LY-11-3	LiuYuan	mafic/ultramafic rock	96.33	41.05	250	9.0	SHRIMP,U-Pb	Zircon	Zhang Y Y, et al.	2011	Gondwana Research.
1149	Outside of the MOO	Beishan	LY-1-33	LiuYuan	mafic/ultramafic rock	96.33	41.05	280		SHRIMP,U-Pb	Zircon	Zhang Y Y, et al.	2011	Gondwana Research.
1150	Outside of the MOO	Beishan	LY-5-11	LiuYuan	mafic/ultramafic rock	96.33	41.05	280		SHRIMP,U-Pb	Zircon	Zhang Y Y, et al.	2011	Gondwana Research.
1151	Outside of the MOO	Beishan	LY15-3	LiuYuan	mafic/ultramafic rock	96.33	41.05	290		SHRIMP,U-Pb	Zircon	Zhang Y Y, et al.	2011	Gondwana Research.

1152	Outside of the MOO	Beishan	LY15-4	LiuYuan	mafic/ultramafic rock	96.33	41.05	290		SHRIMP,U-Pb	Zircon	Zhang Y Y, et al.	2011	Gondwana Research.
1153	Outside of the MOO	Beishan	LY15-6	LiuYuan	mafic/ultramafic rock	96.33	41.05	290		SHRIMP,U-Pb	Zircon	Zhang Y Y, et al.	2011	Gondwana Research.
1154	Outside of the MOO	Beishan	LY-2-6	LiuYuan	mafic/ultramafic rock	96.33	41.05	290		SHRIMP,U-Pb	Zircon	Zhang Y Y, et al.	2011	Gondwana Research.
1155	Outside of the MOO	Beishan	LY-8-11	LiuYuan	mafic/ultramafic rock	96.33	41.05	290		SHRIMP,U-Pb	Zircon	Zhang Y Y, et al.	2011	Gondwana Research.
1156	Outside of the MOO	Beishan	LY-8-13	LiuYuan	mafic/ultramafic rock	96.33	41.05	290		SHRIMP,U-Pb	Zircon	Zhang Y Y, et al.	2011	Gondwana Research.
1157	Outside of the MOO	Beishan	LY-8-19	LiuYuan	mafic/ultramafic rock	96.33	41.05	290		SHRIMP,U-Pb	Zircon	Zhang Y Y, et al.	2011	Gondwana Research.
1158	Outside of the MOO	Beishan	TW48	Heihongshan	Plagiogranite	99.12	42.36	342	4.7	LA-ICP-MS,U-Pb	Zircon	Zhang Z P, et al.	2020	Geological bulletin of China (ICWEA).
1159	Outside of the MOO	Beishan	SZYH4-3	Shazaoyuanfushiyanti	Bi-syenogranite	98.12	40.43	246	2.0	LA-ICP-MS,U-Pb	Zircon	Zhao H G, et al.	2020	Acta Geologica Sinica(ICWEA).
1160	Outside of the MOO	Beishan	SZYH3-1	Shazaoyuanfushiyanti	Bi-Monzogranite	98.13	40.42	249	3.5	LA-ICP-MS,U-Pb	Zircon	Zhao H G, et al.	2020	Acta Geologica Sinica(ICWEA).
1161	Outside of the MOO	Beishan	SZYH1-1	Shazaoyuanfushiyanti	Bi-Granodiorite	98.13	40.43	252	1.9	LA-ICP-MS,U-Pb	Zircon	Zhao H G, et al.	2020	Acta Geologica Sinica(ICWEA).
1162	Outside of the MOO	Beishan	SZYH2-2	Shazaoyuanfushiyanti	Bi-Quartzdiorite	98.13	40.42	252	2.1	LA-ICP-MS,U-Pb	Zircon	Zhao H G, et al.	2020	Acta Geologica Sinica(ICWEA).
1163	Outside of the MOO	Beishan	SJZH2-2	Suanjingziaidakezhigraniteti	Bi-Granodiorite	97.63	41.49	351	5.3	LA-ICP-MS,U-Pb	Zircon	Zhao H G, et al.	2020	Acta Geologica Sinica(ICWEA).
1164	Outside of the MOO	Beishan	SJZH1-1	Suanjingziaidakezhigraniteti	Gneissic Bi-Granodiorite	97.63	41.51	352	5.2	LA-ICP-MS,U-Pb	Zircon	Zhao H G, et al.	2020	Acta Geologica Sinica(ICWEA).
1165	Outside of the MOO	Beishan	SJZH3-1	Suanjingziaidakezhigraniteti	Bi-Monzogranite	97.69	41.51	350	4.5	LA-ICP-MS,U-Pb	Zircon	Zhao H G, et al.	2020	Acta Geologica Sinica(ICWEA).
1166	Outside of the MOO	Beishan	P16TW3	Baiyunshanshelvyandaibeice	Granodiorite	99.98	40.08	349	1.0	LA-ICP-MS,U-Pb	Zircon	Zhao Z N, et al.	2020	Mineralogy and petrology(ICWEA).
1167	Outside of the MOO	Beishan	P16YQ6	BaiYunShanDiQu	Intermediate granodiorite	98.42	41.61	349	2.0	LA-ICP-MS,U-Pb	Zircon	Zhao Z N, et al.	2020	Mineralogy and petrology(ICWEA).
1168	Outside of the MOO	Beishan	BS47-6	Xiaoxigong	Granite	96.83	40.80	269	2.3	LA-ICP-MS,U-Pb	Zircon	Zheng Rongguo et al.	2020	Lithos.
1169	Outside of the MOO	Beishan	BS47–2	Xiaoxigong	Granite	96.85	40.81	268	2.6	LA-ICP-MS,U-Pb	Zircon	Zheng Rongguo et al.	2020	Lithos.
1170	Outside of the MOO	Beishan	DJQ11-9	Xiaoxigong	Granite	96.82	40.82	267	1.3	LA-ICP-MS,U-Pb	Zircon	Zheng Rongguo et al.	2020	Lithos.
1171	Outside of the MOO	Beishan	BS03-4	Yinwaxia	gabbros	96.83	41.03	267	3.3	LA-ICP-MS,U-Pb	Zircon	Zheng Rongguo et al.	2020	Lithos.
1172	Outside of the MOO	Beishan	BS03-5	Yinwaxia	Granite	96.83	41.03	280	2.7	LA-ICP-MS,U-Pb	Zircon	Zheng Rongguo et al.	2020	Lithos.
1173	Outside of the MOO	Beishan	BS03-10	Yinwaxia	Granite	96.84	41.02	280	2.5	LA-ICP-MS,U-Pb	Zircon	Zheng Rongguo et al.	2020	Lithos.
1174	Outside of the MOO	Beishan	BS03-6	Yinwaxia	diorites	96.82	41.05	270	1.9	LA-ICP-MS,U-Pb	Zircon	Zheng Rongguo et al.	2020	Lithos.
1175	Outside of the MOO	Beishan		Baishiquan	K-spar granite	94.95	41.97	303	18.0	LA-ICP-MS,U-Pb	Zircon	Zhou Taofa et al.	2010	Acta Petrologica Sinica (ICWEA).
1176	Outside of the MOO	Beishan		BaiShiQuan	Potassium feldspar granite	94.95	41.97	303	18.0	LA-ICP-MS,U-Pb	Zircon	Zhou Taofa et al.	2010	Acta Petrologica Sinica (ICWEA).
1177	Outside of the MOO	Beishan		Baishandong	Granite	95.94	42.50	285	4.5	LA-ICP-MS,U-Pb	Zircon	Zhou Taofa et al.	2010	Acta Petrologica Sinica (ICWEA).
1178	Outside of the MOO	Beishan		BaiShanDong	granite	95.94	42.50	285	4.5	LA-ICP-MS,U-Pb	Zircon	Zhou Taofa et al.	2010	Acta Petrologica Sinica (ICWEA).
1179	Outside of the MOO	Beishan	PM03TW1	Heihongshan	Monzogranite	98.93	42.63	315	2.5	LA-ICP-MS,U-Pb	Zircon	Zhu W, et al.	2019	Geological bulletin of China (ICWEA).
1180	Outside of the MOO	E. Junggar	X-623	Yiwu	Monzogranite	94.82	43.30	285	1.4	LA-ICP-MS,U-Pb	Zircon	Briggs et al.	2007	Geological Society of America Bulletin.
1181	Outside of the MOO	E. Junggar	S166-1	Batamayineishan	Dacite	89.46	46.41	273	5.0	LA-ICP-MS,U-Pb	Zircon	Briggs et al.	2007	Geological Society of America Bulletin.
1182	Outside of the MOO	E. Junggar	X08-09-01	Yemaquan	Monzogranite	90.39	45.32	300	2.0	LA-ICP-MS,U-Pb	Zircon	Gan lin et al.	2010	Acta Petrologica Sinica(ICWEA).
1183	Outside of the MOO	E. Junggar	XH08-11	Yemaquanyanti	Granodiorite	90.47	45.32	297	6.0	LA-ICP-MS,U-Pb	Zircon	Gan lin et al.	2010	Acta Petrologica Sinica(ICWEA).
1184	Outside of the MOO	E. Junggar	XH08-09-1	Yemaquanyanti	Monzogranite	90.04	45.33	300	2.0	LA-ICP-MS,U-Pb	Zircon	Gan lin et al.	2010	Acta Petrologica Sinica(ICWEA).
1185	Outside of the MOO	E. Junggar	X08-11	Yemaquan	Granodiorite	90.40	45.35	297	6.0	LA-ICP-MS,U-Pb	Zircon	Gan lin et al.	2010	Acta Petrologica Sinica(ICWEA).
1186	Outside of the MOO	E. Junggar	XH08-01	Yemaquanyanti	Alkali-feldspar granite	90.29	45.38	304	3.0	LA-ICP-MS,U-Pb	Zircon	Gan lin et al.	2010	Acta Petrologica Sinica(ICWEA).
1187	Outside of the MOO	E. Junggar	X08-01	Yemaquan	Alkali-feldspar granite	90.30	45.43	304	3.0	LA-ICP-MS,U-Pb	Zircon	Gan lin et al.	2010	Acta Petrologica Sinica(ICWEA).
1188	Outside of the MOO	E. Junggar	XH08-03	Yemaquanyanti	Monzogranite	90.38	45.43	298	2.0	LA-ICP-MS,U-Pb	Zircon	Gan lin et al.	2010	Acta Petrologica Sinica(ICWEA).
1189	Outside of the MOO	E. Junggar	X08-03	Yemaquan	Monzogranite	90.26	45.47	298	2.0	LA-ICP-MS,U-Pb	Zircon	Gan lin et al.	2010	Acta Petrologica Sinica(ICWEA).
1190	Outside of the MOO	E. Junggar	KTG39	Kalatongke	Diorite	89.68	46.76	308	7.0	LA-ICP-MS,U-Pb	Zircon	Gao Jianfeng et al.	2013	Lithos
1191	Outside of the MOO	E. Junggar	KTG07	Kalatongke	Ferrodiorite	89.68	46.76	308	3.6	LA-ICP-MS,U-Pb	Zircon	Gao Jianfeng et al.	2013	Lithos
1192	Outside of the MOO	E. Junggar		Aotawukedongxi	Granodiorite	90.05	44.99	306	1.5	LA-ICP-MS,U-Pb	Zircon	Gao Shanlin et al.	2013	Xinjiang Geology(ICWEA).
1193	Outside of the MOO	E. Junggar	H7-6-26-3.1	lvshigou	Diabase porphyrite	95.04	44.05	233	5.0	SHRIMP,U-Pb	Zircon	Gao Wenjuan et al.	2015	Xinjiang Geology(ICWEA).
1194	Outside of the MOO	E. Junggar	44252	Kubusunanyanti	Alkaline granite	90.22	45.10	286	3.0	LA-ICP-MS,U-Pb	Zircon	Han baofu et al.	2004	Chinese Science Bulletin(ICWEA).
1195	Outside of the MOO	E. Junggar	H13701-9.1	lvshigou	Gabbro-diorite	95.22	44.04	228		SHRIMP,U-Pb	Zircon	Han Baofu et al.	2006	Acta Petrologica Sinica(ICWEA).
1196	Outside of the MOO	E. Junggar	ZR01	Halasutongkuangqu	Quartz-monzonite	90.17	46.47	266	3.7	SHRIMP,U-Pb	Zircon	Han Baofu et al.	2006	Acta Petrologica Sinica(ICWEA).
1197	Outside of the MOO	E. Junggar	K19	Dajiashan	Alkali-feldspar granite	92.63	43.84	287	2.0	SHRIMP,U-Pb	Zircon	Han Baofu et al.	2007	Chinese Science Bulletin(ICWEA).
1198	Outside of the MOO	E. Junggar	LY8	Laoyaquanyanti	Alkali-feldspar granite	89.77	45.33	300	5.0	LA-ICP-MS,U-Pb	Zircon	Han Yujie et al.	2012	Acta Mineralogica Sinica(ICWEA).
1199	Outside of the MOO	E. Junggar	LY6	Laoyaquanyanti	Alkali-feldspar granite	89.75	45.33	301	2.0	LA-ICP-MS,U-Pb	Zircon	Han Yujie et al.	2012	Acta Mineralogica Sinica(ICWEA).
1200	Outside of the MOO	E. Junggar	12LSK-10	Laoshankoubanyantongkuangq	Granodioritic mylonite	90.08	46.52	360	1.0	LA-ICP-MS,U-Pb	Zircon	Hong Tao et al.	2015	Acta Petrologica Sinica(ICWEA).
1201	Outside of the MOO	E. Junggar	H13630-4.1	Baoxi	Monzogranite	95.00	43.91	340	0.8	LA-ICP-MS,U-Pb	Zircon	Hou Jiyao .	2015	Master's thesis(ICWEA)（zhongguodizhidaxue).
1202	Outside of the MOO	E. Junggar	H13630-7.1	Huixigouyantizhongbu	Granite	95.08	44.15	337	2.3	LA-ICP-MS,U-Pb	Zircon	Hou Jiyao .	2015	Master's thesis(ICWEA)（zhongguodizhidaxue).
1203	Outside of the MOO	E. Junggar	H13630-9.1	Luotuoshitougou	Monzogranite	95.04	44.19	337	2.0	LA-ICP-MS,U-Pb	Zircon	Hou Jiyao .	2015	Master's thesis(ICWEA)（zhongguodizhidaxue).
1204	Outside of the MOO	E. Junggar	D6083	Kalamailishelvyan	Tuff	89.94	45.07	343	5.0	LA-ICP-MS,U-Pb	Zircon	Huang Gang et al.	2012	Geological Bulletin of China(ICWEA).
1205	Outside of the MOO	E. Junggar	DH103	Santanghudiquyi	Andesite	94.24	43.66	329	1.9	LA-ICP-MS,U-Pb	Zircon	Huang Wei	2012	Earth Science(ICWEA).
1206	Outside of the MOO	E. Junggar	ML05	Santanghudiquyi	Dacite	94.26	43.74	331	2.3	LA-ICP-MS,U-Pb	Zircon	Huang Wei	2012	Earth Science(ICWEA).
1207	Outside of the MOO	E. Junggar	H13916-1.1	Yiwuxiamaya	Alkaline granite	95.33	43.02	312	1.0	LA-ICP-MS,U-Pb	Zircon	Huang Wei	2014	Master's thesis(ICWEA)(zhongguodizhidaxue).
1208	Outside of the MOO	E. Junggar	H13911-7.1	Hamishichengzi	Alkaline granite	93.72	43.04	310	1.0	LA-ICP-MS,U-Pb	Zircon	Huang Wei	2014	Master's thesis(ICWEA)(zhongguodizhidaxue).
1209	Outside of the MOO	E. Junggar	T90912-2.2	Balikun	Alkaline granite	93.15	43.91	289	1.0	LA-ICP-MS,U-Pb	Zircon	Huang Wei	2014	Master's thesis(ICWEA)(zhongguodizhidaxue).
1210	Outside of the MOO	E. Junggar	ZHB-13-2	Zhaheba	Rhyolite	89.21	46.50	276	2.8	SHRIMP,U-Pb	Zircon	Li Di et al.	2014	Lithos
1211	Outside of the MOO	E. Junggar	K17-34	Batamayineishan Formation	Andesite at a depth of 219 m	89.13	46.53	315	1.6	LA-ICP-MS,U-Pb	Zircon	Li Di et al.	2014	Lithos
1212	Outside of the MOO	E. Junggar	K17-19	Batamayineishan Formation	Andesite at a depth of 103 m	89.13	46.53	317	2.1	LA-ICP-MS,U-Pb	Zircon	Li Di et al.	2014	Lithos
1213	Outside of the MOO	E. Junggar	07AL03-11	Qinghe	Gabbro	89.92	46.73	273	2.4	SHRIMP,U-Pb	Zircon	Li Di et al.	2014	Lithos
1214	Outside of the MOO	E. Junggar	02FY05	Bierangkudongke	Granite	88.92	47.03	273	6.0	SHRIMP,U-Pb	Zircon	Li Di et al.	2014	Lithos
1215	Outside of the MOO	E. Junggar		Xiaopu	Bi-granite	94.18	43.50	311	9.0	SHRIMP,U-Pb	Zircon	Li wuping et al.	2001	Acta Geoscientica Sinica(ICWEA).
1216	Outside of the MOO	E. Junggar	B01	Qitaixianbeilekudongkeyanti	Syenogranite	90.13	45.14	306	5.0	SHRIMP,U-Pb	Zircon	Li Yuechen et al.	2007	Acta Petrologica Sinica(ICWEA).
1217	Outside of the MOO	E. Junggar		Beilekudongke	Syenogranite	90.13	45.15	306	5.0	SHRIMP,U-Pb	Zircon	Li Yuechen et al.	2007	Acta Petrologica Sinica(ICWEA).
1218	Outside of the MOO	E. Junggar	H7-6-26-2.2	lvshigou	Diabase porphyrite	95.04	44.05	242		LA-ICP-MS,U-Pb	Zircon	Li Yuechen et al.	2010	Journal of Xinjiang University(ICWEA).
1219	Outside of the MOO	E. Junggar	S215-1	Yemaquan	Dacite	90.19	45.37	339	3.0	LA-ICP-MS,U-Pb	Zircon	Li Zhensheng et al.	2016	Acta Geologica Sinica(ICWEA).
1220	Outside of the MOO	E. Junggar	S213-1	Yemaquan	Dacite	90.21	45.56	316	5.0	LA-ICP-MS,U-Pb	Zircon	Li Zhensheng et al.	2016	Acta Geologica Sinica(ICWEA).
1221	Outside of the MOO	E. Junggar	YBS02	Yebushan	Granite	90.42	45.62	268	4.0	SHRIMP,U-Pb	Zircon	Li Zhensheng et al.	2016	Acta Geologica Sinica(ICWEA).
1222	Outside of the MOO	E. Junggar	S244-1	Yemaquan	Dacite	89.49	45.99	319	3.0	LA-ICP-MS,U-Pb	Zircon	Li Zhensheng et al.	2016	Acta Geologica Sinica(ICWEA).
1223	Outside of the MOO	E. Junggar	02ET09	Huangyangshan	Enclaves	90.19	46.14	302	6.6	SHRIMP,U-Pb	Zircon	Li Zonghuai et al.	2004	Acta Geologica Sinica(ICWEA).
1224	Outside of the MOO	E. Junggar	02ET04	Huagangyan	Granodiorite	90.16	46.18	299	8.7	SHRIMP,U-Pb	Zircon	Li Zonghuai et al.	2004	Acta Geologica Sinica(ICWEA).
1225	Outside of the MOO	E. Junggar	S2 UM-041124	Sabei	Arfvedsonite granite	90.34	45.19	310	7.0	SHRIMP,U-Pb	Zircon	Lin Jinfu et al.	2007	Acta Petrologica Sinica(ICWEA).
1226	Outside of the MOO	E. Junggar		Sabei	Ferrite granite	90.30	45.20	310	7.0	SHRIMP,U-Pb	Zircon	Lin jinfu et al.	2007	Acta Petrologica Sinica(ICWEA).
1227	Outside of the MOO	E. Junggar		Sabei	Ferrite granite	90.30	45.20	314	10.0	SHRIMP,U-Pb	Zircon	Lin jinfu et al.	2007	Acta Petrologica Sinica(ICWEA).
1228	Outside of the MOO	E. Junggar	S2	Sabeixikuangfukuangdefujian	Arfvedsonite granite	90.35	45.26	313	2.0	SHRIMP,U-Pb	Zircon	Lin Jinfu et al.	2007	Acta Petrologica Sinica(ICWEA).
1229	Outside of the MOO	E. Junggar	S4	Sabeixikuangfukuangdefujian	Arfvedsonite granite	90.37	45.26	314	5.0	SHRIMP,U-Pb	Zircon	Lin Jinfu et al.	2007	Acta Petrologica Sinica(ICWEA).

1230	Outside of the MOO	E. Junggar	S1	Haoxikuangmaine Ceheiyunmu	Tin quartz vein	90.36	45.26	324	3.4	SHRIMP,U-Pb	Zircon	Lin Jinfu et al.	2008	Geology in China(ICWEA).
1231	Outside of the MOO	E. Junggar	SJ02	Sujiquan	Alkali feldspar granite	90.33	45.03	304	1.9	LA-ICP-MS,U-Pb	Zircon	Liu Wei et al.	2013	Lithos
1232	Outside of the MOO	E. Junggar	SR01	Sareshenke	Peralkaline granite	90.31	45.14	301	2.4	LA-ICP-MS,U-Pb	Zircon	Liu Wei et al.	2013	Lithos
1233	Outside of the MOO	E. Junggar	G167	Balebagayi	Alkali feldspar granite	90.29	45.16	311	2.1	LA-ICP-MS,U-Pb	Zircon	Liu Wei et al.	2013	Lithos
1234	Outside of the MOO	E. Junggar	BL02	Beilekudouke	Syenogranite	90.12	45.19	302	2.0	LA-ICP-MS,U-Pb	Zircon	Liu Wei et al.	2013	Lithos
1235	Outside of the MOO	E. Junggar	OT03	Balebagayi	Granodiorite	90.31	45.20	310	2.0	LA-ICP-MS,U-Pb	Zircon	Liu Wei et al.	2013	Lithos
1236	Outside of the MOO	E. Junggar	KLP03	SW. portion of Kurankazigan	Granodiorite	90.32	45.31	315	2.0	LA-ICP-MS,U-Pb	Zircon	Liu Wei et al.	2013	Lithos
1237	Outside of the MOO	E. Junggar	G133	N. Kurankazigan	Syenogranite	90.35	45.42	309	2.1	LA-ICP-MS,U-Pb	Zircon	Liu Wei et al.	2013	Lithos
1238	Outside of the MOO	E. Junggar	G44		Peralkaline granite	89.45	46.14	325	2.0	LA-ICP-MS,U-Pb	Zircon	Liu Wei et al.	2013	Lithos
1239	Outside of the MOO	E. Junggar	G23	Tasigake monzogranite	Monzogranite	89.55	46.15	322	2.0	LA-ICP-MS,U-Pb	Zircon	Liu Wei et al.	2013	Lithos
1240	Outside of the MOO	E. Junggar	G71	Saertielieke	Peralkaline granite	89.21	46.30	322	2.2	LA-ICP-MS,U-Pb	Zircon	Liu Wei et al.	2013	Lithos
1241	Outside of the MOO	E. Junggar	C01-55	Songkaersu Formation	Basaltic andesites	90.08	45.22	338	5.0	LA-ICP-MS,U-Pb	Zircon	Luo Ting et al.	2016	Gondwana Research
1242	Outside of the MOO	E. Junggar	e066-17	Songkaersu Formation	Rhyolitic tuff	90.07	45.20	346	3.0	LA-ICP-MS,U-Pb	Zircon	Luo Ting et al.	2016	Gondwana Research
1243	Outside of the MOO	E. Junggar	X-627	Yiwu	Alkali-feldspar granite	94.79	43.25	284	1.1	LA-ICP-MS,U-Pb	Zircon	Mao Qigui et al.	2008	Acta Petrologica Sinica(ICWEA).
1244	Outside of the MOO	E. Junggar	K28-5-1	Batamayineishan	Rhyolite	89.35	46.38	280	2.5	SHRIMP,U-Pb	Zircon	Mao Qigui et al.	2008	Acta Petrologica Sinica(ICWEA).
1245	Outside of the MOO	E. Junggar	0410TW2	Mayindongbo	Gnessic B-granite	90.13	46.61	283	4.0	SHRIMP,U-Pb	Zircon	Mao Qigui et al.	2008	Acta Petrologica Sinica(ICWEA).
1246	Outside of the MOO	E. Junggar	H7-6-26-2.1	lvshigou	Diabase porphyrite	95.04	44.05	278		SHRIMP,U-Pb	Zircon	Mao Xiang et al.	2014	Journal of Asian Earth Sciences
1247	Outside of the MOO	E. Junggar		Aletunkunduo	Plagiogranite of ophiolite suite in A	94.93	43.77	351	6.0	SIMS,U-Pb	Zircon	Qin Biao et al.	2012	Xinjiang Geology(ICWEA).
1248	Outside of the MOO	E. Junggar	08WLG-16	Saertielieke	Alkaline granite	89.30	46.15	308	6.0	LA-ICP-MS,U-Pb	Zircon	Shen Xiaoming et al.	2011	Journal of Asian Earth Sciences
1249	Outside of the MOO	E. Junggar	08WLG-07	Jierdekala	Alkaline granite	89.38	46.23	291	7.0	LA-ICP-MS,U-Pb	Zircon	Shen Xiaoming et al.	2011	Journal of Asian Earth Sciences
1250	Outside of the MOO	E. Junggar	08WLG1-6	Saertielieke	Alkaline granite	89.16	46.31	308	6.0	LA-ICP-MS,U-Pb	Zircon	Shen Xiaoming et al.	2011	Journal of Asian Earth Sciences
1251	Outside of the MOO	E. Junggar	SJ39	Sujiquan	Bi-granite	90.20	45.03	304	2.0	LA-ICP-MS,U-Pb	Zircon	Su Yuping et al.	2006	Acta Petrologica Et Mineralogica(ICWEA).
1252	Outside of the MOO	E. Junggar		Sujiquan	Bi-granite	90.42	45.50	304	2.0	SHRIMP,U-Pb	Zircon	Su Yuping et al.	2006	Acta Petrologica Et Mineralogica(ICWEA).
1253	Outside of the MOO	E. Junggar	09BJE-79	Baijiandong section	Rhyolite	89.28	44.97	307	1.5	LA-ICP-MS,U-Pb	Zircon	Su Yuping et al.	2012	Gondwana Research
1254	Outside of the MOO	E. Junggar	D75-1	Hadonglikeshan	Diorite	93.84	43.24	316	3.0	SHRIMP,U-Pb	Zircon	Sun Guihua et al.	2005	Geological Review(ICWEA).
1255	Outside of the MOO	E. Junggar		Haerlike	Diorite	93.82	43.23	316	3.0	SHRIMP,U-Pb	Zircon	Sun guihua et al.	2005	Geological Review(ICWEA).
1256	Outside of the MOO	E. Junggar	SHJ-8-4	Batamayineishan Formation	Volcanic rocks	90.45	44.87	350	6.3	SHRIMP,U-Pb	Zircon	Tan Jiayi et al.	2009	Acta Petrologica Sinica(ICWEA).
1257	Outside of the MOO	E. Junggar	DST-1-5	Batamayineishan Formation	Andesite	91.05	43.78	346	7.1	SHRIMP,U-Pb	Zircon	Tan Jiayi et al.	2010	Acta Petrologica Sinica(ICWEA).
1258	Outside of the MOO	E. Junggar	HY19	Sabei	Riebeckite granite	90.24	45.14	306	3.0	LA-ICP-MS,U-Pb	Zircon	Tang Hongfeng et al.	2007	Acta Petrologica Sinica(ICWEA).
1259	Outside of the MOO	E. Junggar	021-17-1	Xumubanhebeice	Hb-gabrro	89.07	45.15	319	3.0	LA-ICP-MS,U-Pb	Zircon	Tian Jian .	2014	Doctoral thesis(ICWEA).
1260	Outside of the MOO	E. Junggar	021-2-1	Dishuiquan	K-spar granite	89.07	45.15	321	2.0	LA-ICP-MS,U-Pb	Zircon	Tian Jian .	2014	Doctoral thesis(ICWEA).
1261	Outside of the MOO	E. Junggar	025-5-1	Wucaicheng	K-spar granite	89.08	45.17	341	4.0	LA-ICP-MS,U-Pb	Zircon	Tian Jian .	2014	Doctoral thesis(ICWEA).
1262	Outside of the MOO	E. Junggar	022-7-1	Wucaicheng	K-spar granite	89.10	45.17	341	5.0	LA-ICP-MS,U-Pb	Zircon	Tian Jian .	2014	Doctoral thesis(ICWEA).
1263	Outside of the MOO	E. Junggar	45778	Wucaicheng Pluton	K-spar granite	89.07	45.17	341	5.1	LA-ICP-MS,U-Pb	Zircon	Tian Jian et al.	2015	Acta Petrologica Sinica(ICWEA).
1264	Outside of the MOO	E. Junggar	40042	bulgen	Alkaline granite	90.81	46.19	353	3.0	LA-ICP-MS,U-Pb	Zircon	Tong Ying et al.	2012	Doctoral thesis(ICWEA).
1265	Outside of the MOO	E. Junggar	sample 4	Fuyun	Unfoliated granitic dike	89.30	47.00	286	12.0	CAMECA,U-Pb	Zircon	Tong Ying et al.	2006	Acta Petrologica Sinica(ICWEA).
1266	Outside of the MOO	E. Junggar	X-379	Badashi	Monzogranite	94.31	42.99	298	2.0	LA-ICP-MS,U-Pb	Zircon	Wang Chuansheng et	2009	Acta Petrologica Sinica(ICWEA).
1267	Outside of the MOO	E. Junggar	X-537	Yiwu	Monzogranite	94.83	43.30	285	1.4	LA-ICP-MS,U-Pb	Zircon	Wang Chuansheng et	2010	Acta Petrologica Sinica(ICWEA).
1268	Outside of the MOO	E. Junggar	BLK2	Dakouzishan	Ferrite granite	92.30	43.77	284	0.7	LA-ICP-MS,U-Pb	Zircon	Wang Chuansheng et	2010	Acta Petrologica Sinica(ICWEA).
1269	Outside of the MOO	E. Junggar	BLK-2	Dajiashan	Alkali granite	92.58	43.87	284	1.0	LA-ICP-MS,U-Pb	Zircon	Wang Chuansheng et	2010	Acta Petrologica Sinica(ICWEA).
1270	Outside of the MOO	E. Junggar	AG-01	wall rock of the Aketas gold de	Granite	89.80	46.73	309	4.7	LA-ICP-MS,U-Pb	Zircon	Wei Xiaofeng et al.	2015	Chinese Journal of Geochemistry(ICWEA).
1271	Outside of the MOO	E. Junggar	HL-011	Halasu III ore district	Alkali granite porphyry	90.03	46.60	327	2.1	LA-ICP-MS,U-Pb	Zircon	Wu Chao et al.	2015	Ore Geology Reviews
1272	Outside of the MOO	E. Junggar	H14825-1.1	Xianhongshankou	K-spar granite	90.65	43.59	295	3.6	LA-ICP-MS,U-Pb	Zircon	Wu Gongcheng et al.	2015	Xinjiang Geology(ICWEA).
1273	Outside of the MOO	E. Junggar	H14825-1.7	Xianhongshankou	Bi-granodiorite	90.65	43.59	297	3.4	LA-ICP-MS,U-Pb	Zircon	Wu Gongcheng et al.	2015	Xinjiang Geology(ICWEA).
1274	Outside of the MOO	E. Junggar	QJ-3-3	Hongliuxiadiqubodongqiangji	Mylonitic granite	91.36	44.07	348	2.8	SIMS,U-Pb	Zircon	Wu Qi et al.	2012	Acta Petrologica Sinica(ICWEA).
1275	Outside of the MOO	E. Junggar	023-3-1	Kalasayi	K-spar granite	89.43	45.35	308	3.2	LA-ICP-MS,U-Pb	Zircon	Wu Qi et al.	2015	Geological Bulletin of China(ICWEA).
1276	Outside of the MOO	E. Junggar	052-7-1	Kalasayi	K-spar granite	89.43	45.35	310	2.0	LA-ICP-MS,U-Pb	Zircon	Wu Qi et al.	2015	Geological Bulletin of China(ICWEA).
1277	Outside of the MOO	E. Junggar	ZF3	West Hill ophiolitic mélange	Andesitic tuff	91.73	44.39	342	1.2	SIMS,U-Pb	Zircon	Xu Xingwang et al.	2015	Journal of Asian Earth Sciences
1278	Outside of the MOO	E. Junggar	ZF2	West Hill ophiolitic mélange	Auartz diorite	91.74	44.39	348	4.3	SIMS,U-Pb	Zircon	Xu Xingwang et al.	2015	Journal of Asian Earth Sciences
1279	Outside of the MOO	E. Junggar	ZF20	West Hill ophiolitic mélange	Granitic porphyry	91.25	44.54	324	4.2	SIMS,U-Pb	Zircon	Xu Xingwang et al.	2015	Journal of Asian Earth Sciences
1280	Outside of the MOO	E. Junggar	ZF16	West Hill ophiolitic mélange	Granodiorite	91.26	44.53	332	1.4	SIMS,U-Pb	Zircon	Xu Xingwang et al.	2015	Journal of Asian Earth Sciences
1281	Outside of the MOO	E. Junggar	H13702-3.1	lvshigou	Gabbro dyke	95.22	44.04	219	4.0	SHRIMP,U-Pb	Zircon	Xue Chunji et al.	2010	Earth Science Frontiers(ICWEA).
1282	Outside of the MOO	E. Junggar	H02	Halasutongkuangqu	Quartz-diorite	90.17	46.47	216	4.6	SHRIMP,U-Pb	Zircon	Xue Chunji et al.	2010	Earth Science Frontiers(ICWEA).
1283	Outside of the MOO	E. Junggar	YKH07	Yulekenhalasubanyantongkuan	Bi quartz porphyry dyke	90.04	46.60	347	1.8	LA-ICP-MS,U-Pb	Zircon	Yang Fuquan et al.	2012	Acta Petrologica Sinica(ICWEA).
1284	Outside of the MOO	E. Junggar	44221	Kubusunanyanti	Granodiorite	90.22	45.10	287	2.0	LA-ICP-MS,U-Pb	Zircon	Yang Gaoxue et al.	2008	Geology in China(ICWEA).
1285	Outside of the MOO	E. Junggar	IV25-1	Kubusunan	Granodiorite	90.19	45.26	287	2.0	LA-ICP-MS,U-Pb	Zircon	Yang Gaoxue et al.	2008	Geology in China(ICWEA).
1286	Outside of the MOO	E. Junggar	06TW-D1191	Huangyangshan	Enclaves of quartz monzonite	90.42	45.05	300	6.0	LA-ICP-MS,U-Pb	Zircon	Yang Gaoxue et al.	2009	Acta Petrologica Sinica(ICWEA).
1287	Outside of the MOO	E. Junggar	06TW-X-29-1	Huangyangshan pluton	Alkaline granite	90.32	45.07	311	12.0	LA-ICP-MS,U-Pb	Zircon	Yang Gaoxue et al.	2009	Acta Petrologica Sinica(ICWEA).
1288	Outside of the MOO	E. Junggar	K28-16-1	Batamayineishan	Rhyolite	89.40	46.38	276	3.0	SHRIMP,U-Pb	Zircon	Yang Gaoxue et al.	2010	Geotectonica et Metallogenia(ICWEA).
1289	Outside of the MOO	E. Junggar	D1191	Huagangyan	Enclaves	90.42	45.05	300	6.0	LA-ICP-MS,U-Pb	Zircon	Yang Gaoxue et al.	2011	Journal of Asian Earth Sciences
1290	Outside of the MOO	E. Junggar	X-29-1	Huangyangshan pluton o	A-type granites	90.32	45.07	311	5.0	LA-ICP-MS,U-Pb	Zircon	Yang Gaoxue et al.	2011	Journal of Asian Earth Sciences
1291	Outside of the MOO	E. Junggar	12XL03	Fuyunxianxilekudongketongkua	Granite porphyry	90.63	45.12	315	2.4	LA-ICP-MS,U-Pb	Zircon	You Jun et al.	2016	Acta Petrologica Sinica(ICWEA).
1292	Outside of the MOO	E. Junggar	11XL05	Fuyunxianxilekudongketongkua	Granite porphyry	90.63	45.12	325	2.2	LA-ICP-MS,U-Pb	Zircon	You Jun et al.	2016	Acta Petrologica Sinica(ICWEA).
1293	Outside of the MOO	E. Junggar	11XL07	Fuyunxianxilekudongketongkua	Monzogranite	90.63	45.12	328	1.6	LA-ICP-MS,U-Pb	Zircon	You Jun et al.	2016	Acta Petrologica Sinica(ICWEA).
1294	Outside of the MOO	E. Junggar	12XL01	Fuyunxianxilekudongketongkua	Granodiorite	90.63	45.12	340	2.8	CAMECA,U-Pb	Zircon	You Jun et al.	2016	Acta Petrologica Sinica(ICWEA).
1295	Outside of the MOO	E. Junggar	K09	Daliugou	Bi-monzogranite	93.71	43.58	288	3.0	SHRIMP,U-Pb	Zircon	Yuan Chao et al	2010	Lithos
1296	Outside of the MOO	E. Junggar	K18	Shiquanzi	Gabbro	92.10	43.79	301	6.0	SHRIMP,U-Pb	Zircon	Yuan Chao et al	2010	Lithos
1297	Outside of the MOO	E. Junggar		Diqubeilekudongke	Bi-syenogranite	90.05	45.18	283	2.0	LA-ICP-MS,U-Pb	Zircon	Yuan Chao et al	2010	Lithos
1298	Outside of the MOO	E. Junggar		Kalatongke	Biotite syenite	89.68	46.77	287	5.0	SHRIMP,U-Pb	Zircon	Yuan Chao et al	2010	Lithos
1299	Outside of the MOO	E. Junggar	SB1	Kalamailigouzaodaidongnanbu	Granodiorite porphyry	90.20	44.84	306	9.3	SHRIMP,U-Pb	Zircon	Zhang Feng et al.	2014	Xinjiang Geology(ICWEA).
1300	Outside of the MOO	E. Junggar	SB17	Kalamailigouzaodaidongnanbu	Granodiorite porphyry	90.20	44.84	316	7.8	SHRIMP,U-Pb	Zircon	Zhang Feng et al.	2014	Xinjiang Geology(ICWEA).
1301	Outside of the MOO	E. Junggar	H13702-1.1	lvshigou	Gabbro	95.23	44.04	218	4.8	SHRIMP,U-Pb	Zircon	Zhang Jianjun et al.	2019	Acta Petrologica Et Mineralogica(ICWEA).
1302	Outside of the MOO	E. Junggar	H7-6-26-1.1	lvshigou	Diabase porphyrite	95.04	44.06	346	0.9	LA-ICP-MS,U-Pb	Zircon	Zhang Jianjun et al.	2019	Acta Petrologica Et Mineralogica(ICWEA).
1303	Outside of the MOO	E. Junggar	H7-6-26-1.2	lvshigou	Diabase porphyrite	95.04	44.06	355	4.7	SHRIMP,U-Pb	Zircon	Zhang Jianjun et al.	2019	Acta Petrologica Et Mineralogica(ICWEA).
1304	Outside of the MOO	E. Junggar	STH2	Santanghupendimalangaoxianz	Basalt	94.40	43.93	307	5.3	LA-ICP-MS,U-Pb	Zircon	Zhang Yong et al.	2010	Acta Petrologica Sinica(ICWEA).
1305	Outside of the MOO	E. Junggar	STH3	Santanghupendimalangaoxianz	Basalt	94.40	43.93	325	3.0	LA-ICP-MS,U-Pb	Zircon	Zhang Yong et al.	2010	Acta Petrologica Sinica(ICWEA).
1306	Outside of the MOO	E. Junggar	STH1	Santanghupendimalangaoxianz	Basalt	94.40	43.93	344	2.4	LA-ICP-MS,U-Pb	Zircon	Zhang Yong et al.	2010	Acta Petrologica Sinica(ICWEA).
1307	Outside of the MOO	E. Junggar	SETH12-20	Sadongtuohaitiekuangqu	Diorite	90.32	45.96	269	0.7	LA-ICP-MS,U-Pb	Zircon	Zhang Yuanyuan et al	2009	Acta Petrologica Sinica(ICWEA).

1308	Outside of the MOO	E. Junggar	SHJ-8–4	Batamayineishan Formation	Rachyandesite	90.35	44.75	350	6.0	SHRIMP,U-Pb	Zircon	Zhang Yuanyuan et al	2013	GSA Bulletin.
1309	Outside of the MOO	E. Junggar		Batamayineishan Formation	Volcanic rocks	90.47	44.83	350	6.0	LA-ICP-MS,U-Pb	Zircon	Zhang Yuanyuan et al	2015	Gondwana Research
1310	Outside of the MOO	E. Junggar	H710-4.1	Balegantinghadongshanyanti	K-spar granite	95.02	44.09	309	1.3	LA-ICP-MS,U-Pb	Zircon	Zhao Jianxin	2014	Master's thesis(ICWEA).
1311	Outside of the MOO	E. Junggar	H703-06-8.2	Qionghebayanti	Bi-quartz monzonite	95.04	44.08	354	1.0	LA-ICP-MS,U-Pb	Zircon	Zhao Jianxin	2014	Master's thesis(ICWEA).
1312	Outside of the MOO	E. Junggar	H13630-5.1	Qionghebayanti	Bi-monzonite	95.09	44.12	346	1.5	LA-ICP-MS,U-Pb	Zircon	Zhao Jianxin	2014	Master's thesis(ICWEA).
1313	Outside of the MOO	E. Junggar	K28-18-1	Batamayineishan	Rhyolite	89.45	46.35	276	3.1	SHRIMP,U-Pb	Zircon	Zhou Gang et al.	2007	Acta Petrologica Sinica(ICWEA).
1314	Outside of the MOO	E. Junggar	sample 7	S. Ertix	Unfoliated granite	89.31	47.00	278	7.0	CAMECA,U-Pb	Zircon	Zhou Gang et al.	2007	Acta Petrologica Sinica(ICWEA).
1315	Outside of the MOO	E. Junggar	06Q3	Hadaxun	Monzonite	90.17	46.39	290	3.6	SHRIMP,U-Pb	Zircon	Zhou Gang et al.	2009	Acta Petrologica Sinica(ICWEA).
1316	Outside of the MOO	Xilamulun	PM210-12-1	Linxi-Chifeng	Medium fine-grained Monzogranite	119.69	42.00	270	1.6	LA-ICP-MS,U-Pb	Zircon			1:50 000 regional surveys of Yuan Baoshan coal mine in Inner Mongolia.
1317	Outside of the MOO	Xilamulun	PM210-6-1	Linxi-Chifeng	Medium coarse Biotite Granite	119.67	42.02	248	1.2	LA-ICP-MS,U-Pb	Zircon			1:50 000 regional surveys of Yuan Baoshan coal mine in Inner Mongolia.
1318	Outside of the MOO	Xilamulun	PM201-18-1	Linxi-Chifeng	Medium fine-grained Monzogranite	119.46	42.20	159	1.6	LA-ICP-MS,U-Pb	Zircon			1:50 000 regional surveys of Yuan Baoshan coal mine in Inner Mongolia.
1319	Outside of the MOO	Xilamulun	PM201-24-2	Linxi-Chifeng	Medium coarse Porphyritic biotite r	119.43	42.21	160	1.1	LA-ICP-MS,U-Pb	Zircon			1:50 000 regional surveys of Yuan Baoshan coal mine in Inner Mongolia.
1320	Outside of the MOO	Xilamulun	PM201-23-2	Linxi-Chifeng	Fine grained Monzogranite	119.44	42.21	161	3.9	LA-ICP-MS,U-Pb	Zircon			1:50 000 regional surveys of Yuan Baoshan coal mine in Inner Mongolia.
1321	Outside of the MOO	Xilamulun	D2711-1	Linxi-Chifeng	Medium coarse Muscovite monzoni	119.47	42.30	251	2.3	LA-ICP-MS,U-Pb	Zircon			1:50 000 regional surveys of Yuan Baoshan coal mine in Inner Mongolia.
1322	Outside of the MOO	Xilamulun	PM205-9-2	Linxi-Chifeng	Medium coarseGranidiorite	119.47	42.29	354	1.1	LA-ICP-MS,U-Pb	Zircon			1:50 000 regional surveys of Yuan Baoshan coal mine in Inner Mongolia.
1323	Outside of the MOO	Xilamulun	D2710-1	Linxi-Chifeng	Medium coarse Bi-syenite	119.54	42.30	349	1.7	LA-ICP-MS,U-Pb	Zircon			1:50 000 regional surveys of Yuan Baoshan coal mine in Inner Mongolia.
1324	Outside of the MOO	Xilamulun	PM037-TW9-4	Linxi-Chifeng	Bi-Monzogranite	118.49	43.02	155	0.8	LA-ICP-MS,U-Pb	Zircon			1:50 000 regional surveys of Yuan Baoshan coal mine in Inner Mongolia.
1325	Outside of the MOO	Xilamulun	PM037-TW12-2	Linxi-Chifeng	Fine grained Monzogranite	118.47	43.04	276	1.4	LA-ICP-MS,U-Pb	Zircon			1:50 000 regional surveys of Yuan Baoshan coal mine in Inner Mongolia.
1326	Outside of the MOO	Xilamulun	PM036-5TW5-1	Linxi-Chifeng	Granite	118.44	43.05	252	2.0	LA-ICP-MS,U-Pb	Zircon			1:50 000 regional surveys of Yuan Baoshan coal mine in Inner Mongolia.
1327	Outside of the MOO	Xilamulun	PM034-48TW1	Linxi-Chifeng	Quartz-diorite	118.28	43.07	158	1.7	LA-ICP-MS,U-Pb	Zircon			1:50 000 regional surveys of Yuan Baoshan coal mine in Inner Mongolia.
1328	Outside of the MOO	Xilamulun	PM034-7TW1	Linxi-Chifeng	Porphyritic monzogranite	118.21	43.11	149	2.3	LA-ICP-MS,U-Pb	Zircon			1:50 000 regional surveys of Yuan Baoshan coal mine in Inner Mongolia.
1329	Outside of the MOO	Xilamulun	0CY016-3	Proterozoic rock mass	Hornblende Gabbro	120.36	41.83	269	2.0	LA-ICP-MS,U-Pb	Zircon	Bai X et al.	2013	International Journal of Earth Sciences
1330	Outside of the MOO	Xilamulun	CY033-3	Archaean Xiaotaizigou Formati	Hornblende Gabbro	120.41	41.84	295	3.0	LA-ICP-MS,U-Pb	Zircon	Bai X et al.	2013	International Journal of Earth Sciences
1331	Outside of the MOO	Xilamulun	FX002-1	Archaean Xiaotaizigou Formati	Biotite-diorite	121.61	42.16	267	1.0	LA-ICP-MS,U-Pb	Zircon	Bai X et al.	2013	International Journal of Earth Sciences
1332	Outside of the MOO	Xilamulun	CY021-1	Archaean Xiaotaizigou Formati	Hornblende Gabbro	120.50	42.17	276	2.0	LA-ICP-MS,U-Pb	Zircon	Bai X et al.	2013	International Journal of Earth Sciences
1333	Outside of the MOO	Xilamulun	CY005-2	Archaean Xiaotaizigou Formati	Hornblende Gabbro	120.83	42.20	259	2.0	LA-ICP-MS,U-Pb	Zircon	Bai X et al.	2013	International Journal of Earth Sciences
1334	Outside of the MOO	Xilamulun	FX007-1	Archaean Xiaotaizigou Formati	Monzogranite	121.88	42.44	251	2.0	LA-ICP-MS,U-Pb	Zircon	Bai X et al.	2013	International Journal of Earth Sciences
1335	Outside of the MOO	Xilamulun	6		Diorite	113.80	43.31	309		TIMS,U-Pb	Zircon	Chen Bin et al.	2000	Tectonophysics
1336	Outside of the MOO	Xilamulun	93SS-2	Baiyin Baoli Road Rock Mass	Quartz-diorite	113.57	43.61	309	8.0	SHRIMP,U-Pb	Zircon	Chen Bin et al.	2000	Tectonophysics
1337	Outside of the MOO	Xilamulun	BP24-5	Baerhan Lama Temple Rock su	Monzogranite	113.80	43.47	254	4.0	SHRIMP,U-Pb	Zircon	Chen Bin et al.	2001	Geological Review (ICWEA)
1338	Outside of the MOO	Xilamulun	93SS-1/2	Baiyin Baoli rock mass	Quartz-diorite	113.60	43.57	309	8.0	SHRIMP,U-Pb	Zircon	Chen Bin et al.	2001	Geological Review (ICWEA)
1339	Outside of the MOO	Xilamulun	HLT-2	Harato Rock	Granite	113.55	43.52	234	5.0	SHRIMP,U-Pb	Zircon	Chen Bin et al.	2009	Journal of Asian Earth Sciences
1340	Outside of the MOO	Xilamulun	BLD-1	Baolidao rock mass	Diorite	113.57	43.61	310	5.0	SHRIMP,U-Pb	Zircon	Chen Bin et al.	2009	Journal of Asian Earth Sciences
1341	Outside of the MOO	Xilamulun	XH-2	Baolidao arc rocks	quartz diorite(Quartz-diorite)	116.08	43.82	310	5.0	SHRIMP,U-Pb	Zircon	Chen Bin et al.	2009	Journal of Asian Earth Sciences
1342	Outside of the MOO	Xilamulun		Dayingzi	Syenogranite	118.63	42.42	268	2.8	LA-ICP-MS,U-Pb	Zircon	Chi Aihua et al.	2015	Journal of Jilin University (Earth Science Edition) (ICWEA).
1343	Outside of the MOO	Xilamulun	PRC-22	Chifeng Louzi Dian	Monzogranite	119.08	42.00	253	3.0	TIMS,U-Pb	Zircon	Davis et al.	2001	
1344	Outside of the MOO	Xilamulun		Guodi Mountain	Granite	119.08	42.00	253	3.0	U-Pb	Zircon	Davis G A et al.	1998	Department of Geology , Peking
1345	Outside of the MOO	Xilamulun	JB6024	Fengshan	Diorite	117.12	41.23	315	2.8	LA-ICP-MS,U-Pb	Zircon	Feng Yonggang et al.	2009	Acta Scientiarum Naturalium Universitatis Pekinensis (ICWEA).
1346	Outside of the MOO	Xilamulun	JB6037-1	Fengshan	Monzogranite	117.41	41.23	306	6.0	LA-ICP-MS,U-Pb	Zircon	Feng Yonggang et al.	2009	Acta Scientiarum Naturalium Universitatis Pekinensis (ICWEA).
1347	Outside of the MOO	Xilamulun	DQ08-110	Shenshuiling, Hohhot	Granidiorite	112.02	41.06	148	1.0	LA-ICP-MS,U-Pb(Z	Zircon	Guo Lei et al.	2012	International Geology Review
1348	Outside of the MOO	Xilamulun	AEG-05	Aoergai copper mine	Granidiorite	118.84	43.85	245	1.8	SHRIMP,U-Pb	Zircon	Guo Zhijun et al.	2012	Geology in China (ICWEA).
1349	Outside of the MOO	Xilamulun	WBW09-16-4	Amuwu rock mass	Monzogranite	114.06	43.95	235	3.2	LA-ICP-MS,U-Pb	Zircon	Guo Zhitao et al.	2014	Global Geology (ICWEA).
1350	Outside of the MOO	Xilamulun	H20	Hadamiao gold complex	Quartz-diorite	113.62	42.31	273	2.4	LA-ICP-MS,U-Pb	Zircon	Hao Baiwu et al.	2010	Acta Petrologica et Mineralogica (ICWEA)
1351	Outside of the MOO	Xilamulun	XN09一203	Xianghuangqi	Bi-moyite	113.87	42.12	263	2.1	LA-ICP-MS,U-Pb	Zircon	Hao Baiwu et al.	2011	Kunming: Kunming University of Science and Technology.(ICWEA).
1352	Outside of the MOO	Xilamulun	XB09-001	Xianghuang Banner North	Monzogranite	113.85	42.30	266	2.0	LA-ICP-MS,U-Pb	Zircon	Hao Baiwu et al.	2011	Kunming: Kunming University of Science and Technology.(ICWEA).
1353	Outside of the MOO	Xilamulun	B09-033	Hadamiao gold deposit comple	Granite porphyry	113.63	42.31	270	1.6	LA-ICP-MS,U-Pb	Zircon	Hao Baiwu et al.	2011	Kunming: Kunming University of Science and Technology.(ICWEA).
1354	Outside of the MOO	Xilamulun	B09-020	Hada Temple quartz diorite roc	Quartz-diorite	113.63	42.31	273	2.4	LA-ICP-MS,U-Pb	Zircon	Hao Baiwu et al.	2011	Kunming: Kunming University of Science and Technology.(ICWEA).
1355	Outside of the MOO	Xilamulun	BW09-501	Hadamiao area	Granidiorite	113.56	42.33	267	1.3	LA-ICP-MS,U-Pb	Zircon	Hao Baiwu et al.	2011	Kunming: Kunming University of Science and Technology.(ICWEA).
1356	Outside of the MOO	Xilamulun	B09-018	Hada Temple	Granite porphyry	113.52	42.34	258	2.7	LA-ICP-MS,U-Pb	Zircon	Hao Baiwu et al.	2011	Kunming: Kunming University of Science and Technology.(ICWEA).
1357	Outside of the MOO	Xilamulun	B000-209	Au - bearing diorite porphyry i	Diorite porphyry	113.55	42.39	269	2.5	LA-ICP-MS,U-Pb	Zircon	Hao Baiwu et al.	2011	Kunming: Kunming University of Science and Technology.(ICWEA).
1358	Outside of the MOO	Xilamulun	XN09-203	Xianghuangqi	Bi-moyite	113.87	42.12	263	2.0	LA-ICP-MS,U-Pb	Zircon	Hao Baiwu et al.	2012	Journal of Jilin University(Earth Science Edition) (ICWEA).
1359	Outside of the MOO	Xilamulun	HY09-005	Narenwula biotite granite body	Biotite granite	114.23	42.24	267	1.4	LA-ICP-MS,U-Pb	Zircon	Hao Baiwu et al.	2012	Mineralogy and Petrology (ICWEA).
1360	Outside of the MOO	Xilamulun	SDG1	Shadgay	Monzogranite	109.61	40.80	222	2.0	SHRIMP,U-Pb	Zircon	Hou Wanrong et al.	2011	Journal of Jilin University(Earth Science Edition) (ICWEA).
1361	Outside of the MOO	Xilamulun	HLT-1	Harato Rock	Syenogranite	113.72	43.56	233	2.0	LA-ICP-MS,U-Pb	Zircon	Hu Chuansheng et al.	2015	Journal of Asian Earth Sciences
1362	Outside of the MOO	Xilamulun	HLT-2	Harato Rock	Syenogranite	113.72	43.56	344	2.0	LA-ICP-MS,U-Pb	Zircon	Hu Chuansheng et al.	2015	Journal of Asian Earth Sciences
1363	Outside of the MOO	Xilamulun	BLD-1	aiyin Baoli Road Rock Mass	Granidiorite	113.63	43.65	316	1.0	LA-ICP-MS,U-Pb	Zircon	Hu Chuansheng et al.	2015	Journal of Asian Earth Sciences
1364	Outside of the MOO	Xilamulun	BLD-3	Baiyin Baoli Road Rock Mass	Monzogranite	113.63	43.65	322	1.0	LA-ICP-MS,U-Pb	Zircon	Hu Chuansheng et al.	2015	Journal of Asian Earth Sciences
1365	Outside of the MOO	Xilamulun	NMD25	Benbatu rock mass	Hornblende biotite granodiorite	112.61	43.73	337	2.3	LA-ICP-MS,U-Pb	Zircon	Huang Dingling et al.	2014	Beijing: China University of Geosciences, Beijing.(ICWEA).
1366	Outside of the MOO	Xilamulun	NMD17	Sayinwusu Pluton	Granite	112.33	43.67	274	2.3	LA-ICP-MS,U-Pb	Zircon	Huang Dingling et al.	2014	Beijing: China University of Geosciences, Beijing.(ICWEA).
1367	Outside of the MOO	Xilamulun	NM78	Donggou Nanshan Rock	Monzogranite	112.69	41.33	312	4.0	SHRIMP,U-Pb	Zircon	Huang Dingling et al.	2014	Beijing: China University of Geosciences, Beijing.(ICWEA).
1368	Outside of the MOO	Xilamulun	NM73-1	Donggou Nanshan Rock	Monzogranite	112.69	41.33	325	3.6	SHRIMP,U-Pb	Zircon	Huang Dingling et al.	2014	Beijing: China University of Geosciences, Beijing.(ICWEA).
1369	Outside of the MOO	Xilamulun	NM145	Bayinaobao Granite Pluton	Granite	113.30	42.22	347	9.2	SHRIMP,U-Pb	Zircon	Huang Dingling et al.	2014	Beijing: China University of Geosciences, Beijing.(ICWEA).
1370	Outside of the MOO	Xilamulun	NMD09	Amenwusu Pluton	Granite	112.22	42.58	256	1.4	LA-ICP-MS,U-Pb	Zircon	Huang Dingling et al.	2014	Beijing: China University of Geosciences, Beijing.(ICWEA).
1371	Outside of the MOO	Xilamulun	NMD11	Hugil Rock	Granite	112.74	43.17	291	1.7	LA-ICP-MS,U-Pb	Zircon	Huang Dingling et al.	2014	Beijing: China University of Geosciences, Beijing.(ICWEA).
1372	Outside of the MOO	Xilamulun	DYCWL-3	chuanwulu	biotite monzonite	76.80	40.75	286	2.5	LA-ICP-MS,U-Pb	Zircon	Huang He et al.	2008	Acta Geologica Sinica(ICWEA)
1373	Outside of the MOO	Xilamulun	DYCWL-4	chuanwulu	biotite diorite	76.82	40.79	291	5.9	LA-ICP-MS,U-Pb	Zircon	Huang He et al.	2008	Acta Geologica Sinica(ICWEA)
1374	Outside of the MOO	Xilamulun	q-02	yingmailai	granite	80.79	42.00	291	2.6	LA-ICP-MS,U-Pb	Zircon	Huang He et al.	2008	Acta Geologica Sinica(ICWEA)
1375	Outside of the MOO	Xilamulun	P1W68	Horn hole	Migmatitic granite	119.21	41.93	327	0.7	TIMS,U-Pb	Zircon	Jia Wen et al.	1999	Geology of Inner Mongolia (ICWEA).
1376	Outside of the MOO	Xilamulun	CGHDM01	Hugiert–Changanhadamiao	diabase	110.47	42.42	284	4.0	SHRIMP,U-Pb	Zircon	Jian Ping et al.	2010	Lithos
1377	Outside of the MOO	Xilamulun	CGHDM01-2	Hugiert–Changanhadamiao	diabase	110.47	42.42	288	6.0	SHRIMP,U-Pb	Zircon	Jian Ping et al.	2010	Lithos
1378	Outside of the MOO	Xilamulun	SLS04-3	Mandula	Diabase	109.15	42.53	250	2.4	SHRIMP,U-Pb	Zircon	Jian Ping et al.	2010	Lithos
1379	Outside of the MOO	Xilamulun	MSL13-2	Mongolian Solonker	Diorite	109.15	42.53	252	1.1	SHRIMP,U-Pb	Zircon	Jian Ping et al.	2010	Lithos
1380	Outside of the MOO	Xilamulun	SLS04-4	Mandula	Diabase	109.15	42.53	253	2.3	SHRIMP,U-Pb	Zircon	Jian Ping et al.	2010	Lithos
1381	Outside of the MOO	Xilamulun	SLS06	Mandula	Diabase	109.15	42.53	274	2.5	SHRIMP,U-Pb	Zircon	Jian Ping et al.	2010	Lithos
1382	Outside of the MOO	Xilamulun	SLS01	Mandula	Gabbro-diorite	109.15	42.53	292	2.3	SHRIMP,U-Pb	Zircon	Jian Ping et al.	2010	Lithos
1383	Outside of the MOO	Xilamulun	MSL02	Mongolian Solonker	Tonalite	109.15	42.53	295	2.4	SHRIMP,U-Pb	Zircon	Jian Ping et al.	2010	Lithos
1384	Outside of the MOO	Xilamulun	MSL01	Mongolian Solonker	Gabbro	109.15	42.53	297	1.7	SHRIMP,U-Pb	Zircon	Jian Ping et al.	2010	Lithos
1385	Outside of the MOO	Xilamulun	SLS02	Mandula	Granite	109.15	42.53	313	1.8	SHRIMP,U-Pb	Zircon	Jian Ping et al.	2010	Lithos

1386	Outside of the MOO	Xilamulun	SLS05	Mandula	Granite	109.15	42.53	314	3.1	SHRIMP,U-Pb	Zircon	Jian Ping et al.	2010	Lithos
1387	Outside of the MOO	Xilamulun	MSL03	Mongolian Solonker	Trondhjemite	109.15	42.53	324	2.7	SHRIMP,U-Pb	Zircon	Jian Ping et al.	2010	Lithos
1388	Outside of the MOO	Xilamulun	N301	Honghualiang granite	Granite	115.38	40.92	235	2.0	SHRIMP,U-Pb	Zircon	Jiang Neng et al.	2007	Geochimica et Cosmochimica Acta
1389	Outside of the MOO	Xilamulun	ME10-6	Menn Tolgoi	muscovite plagiogranite	121.37	45.21	234	3.0	LA-ICP-MS,U-Pb	Zircon	Jiang Sihong et al.	2011	Journal of Jilin University(Earth Science Edition) (ICWEA).
1390	Outside of the MOO	Xilamulun	ME10-9	Menn Tolgoi	Bi-plagiogranite	121.37	45.21	241	1.0	LA-ICP-MS,U-Pb	Zircon	Jiang Sihong et al.	2011	Journal of Jilin University(Earth Science Edition) (ICWEA).
1391	Outside of the MOO	Xilamulun	ME10-21	Menn Tolgoi	Bi-syenite	121.15	45.22	155	1.0	LA-ICP-MS,U-Pb	Zircon	Jiang Sihong et al.	2011	Journal of Jilin University(Earth Science Edition) (ICWEA).
1392	Outside of the MOO	Xilamulun	M10-21	Menn Tolgoi	Bi-syenite	121.15	45.22	155	1.0	LA-ICP-MS,U-Pb	Zircon	Jiang Sihong et al.	2011	Journal of Jilin University(Earth Science Edition) (ICWEA).
1393	Outside of the MOO	Xilamulun	DJ10-15	Rock vein	Quartz porphyry	118.18	43.36	146	0.9	LA-MC-ICP-MS	Zircon	Jiang Sihong et al.	2012	Acta Petrologica Sinica (ICWEA).
1394	Outside of the MOO	Xilamulun	DJ10-8	Tangjiayingzi dyke	Anshan porphyrite	118.26	43.59	252	1.8	LA-MC-ICP-MS	Zircon	Jiang Sihong et al.	2012	Acta Petrologica Sinica (ICWEA).
1395	Outside of the MOO	Xilamulun	DJ10-1	The southern end of Daxinganl	Felsite	118.26	43.69	171	1.4	LA-MC-ICP-MS	Zircon	Jiang Sihong et al.	2012	Acta Petrologica Sinica (ICWEA)
1396	Outside of the MOO	Xilamulun	DJ10-3	Rock vein	靡细岩	118.26	43.69	171	1.1	LA-MC-ICP-MS	Zircon	Jiang Sihong et al.	2012	Acta Petrologica Sinica (ICWEA).
1397	Outside of the MOO	Xilamulun	DJ10-4	Maanshan rock mass	Bi-Monzogranite	118.00	43.78	280	1.3	LA-MC-ICP-MS	Zircon	Jiang Sihong et al.	2012	Acta Petrologica Sinica (ICWEA).
1398	Outside of the MOO	Xilamulun	DJ10-6	Dasi Duan Village Quarry	Bi-Monzogranite	118.18	43.81	243	2.0	LA-ICP-MS,U-Pb	Zircon	Jiang Sihong et al.	2012	Acta Petrologica Sinica (ICWEA).
1399	Outside of the MOO	Xilamulun	DJ10-18	Shuangjingzi Complex	Gneissic Bi-granite	118.29	43.30	278	1.4	LA-MC-ICP-MS	Zircon	Jiang Sihong et al.	2014	Geology in China (ICWEA).
1400	Outside of the MOO	Xilamulun	PM33TW1	Guangxingyuan retested rock m	Granidiorite	117.62	42.91	263	2.5	SHRIMP,U-Pb	Zircon	Jiang Xiaojun et al.	2011	Acta Geologica Sinica (ICWEA).
1401	Outside of the MOO	Xilamulun	TW5532-1	Guangxingyuan retested rock m	Quartz-Monzonite	117.73	42.92	264	2.1	SHRIMP,U-Pb	Zircon	Jiang Xiaojun et al.	2011	Acta Geologica Sinica (ICWEA).
1402	Outside of the MOO	Xilamulun	JN-51	Ulan Hada Granite Body	Medium coarse Alkali-feldspar gran	113.50	42.33	265	1.5	LA-ICP-MS,U-Pb	Zircon	Jiang Xiaojun et al.	2013	Geological Bulletin of China(ICWEA)
1403	Outside of the MOO	Xilamulun	P27b6-1	Honggeltu Granite	Flesh red Medium coarse Granite	113.28	41.69	269	1.6	LA-ICP-MS,U-Pb	Zircon	Jiang Xiaojun et al.	2014	Chang chun: Jilin University.(ICWEA).
1404	Outside of the MOO	Xilamulun	JN-57	Honggeltu Granite	Flesh red Medium coarse Granite	113.52	42.27	272	1.2	LA-ICP-MS,U-Pb	Zircon	Jiang Xiaojun et al.	2014	Chang chun: Jilin University.(ICWEA).
1405	Outside of the MOO	Xilamulun	D42-1	Shuangjingzi	Granitic gneiss	118.33	43.25	238	2.7	SHRIMP,U-Pb	Zircon	Li Jinyi et al.	2007	Acta Petrologica Sinica (ICWEA).
1406	Outside of the MOO	Xilamulun	D98-1	Shuangjingzi	Dimica granite	118.07	43.29	229	4.1	SHRIMP,U-Pb	Zircon	Li Jinyi et al.	2007	Acta Petrologica Sinica (ICWEA)
1407	Outside of the MOO	Xilamulun	D105-3	Shuangjingzi	Dimica granite	118.11	43.33	237	2.7	SHRIMP,U-Pb	Zircon	Li Jinyi et al.	2007	Acta Petrologica Sinica (ICWEA).
1408	Outside of the MOO	Xilamulun	11SH-3	Shahutong	Granidiorite	117.32	43.79	275	3.0	SHRIMP,U-Pb	Zircon	Li Shan et al.	2014	Gondwana Research.
1409	Outside of the MOO	Xilamulun	11XL-5.2	Beidashan	Granidiorite	117.60	43.95	277	3.0	SHRIMP,U-Pb	Zircon	Li Shan et al.	2014	Gondwana Research.
1410	Outside of the MOO	Xilamulun	XL920-8	Sumutai	Granodorite	116.90	43.54	252	2.0	SHRIMP,U-Pb	Zircon	Li Shan et al.	2016	Scientific Reports
1411	Outside of the MOO	Xilamulun	11SH-5	Salihada	Granodorite	117.57	43.67	253	4.0	SHRIMP,U-Pb	Zircon	Li Shan et al.	2016	Scientific Reports
1412	Outside of the MOO	Xilamulun	XL922-2	Beikeli	Granodorite	116.02	43.69	255	2.0	SHRIMP,U-Pb	Zircon	Li Shan et al.	2016	Scientific Reports
1413	Outside of the MOO	Xilamulun	XL921-14	Baiyinwendu	Granodorite	116.65	43.77	251	2.0	SHRIMP,U-Pb	Zircon	Li Shan et al.	2016	Scientific Reports
1414	Outside of the MOO	Xilamulun	11SH-3	Shahutong	Granidiorite	117.32	43.79	275	3.0	SHRIMP,U-Pb	Zircon	Li Shan et al.	2016	Gondwana Research.
1415	Outside of the MOO	Xilamulun	11XL-5.2	Beidashan	Granidiorite	117.60	43.95	277	3.0	SHRIMP,U-Pb	Zircon	Li Shan et al.	2016	Gondwana Research.
1416	Outside of the MOO	Xilamulun	11SJ-03	Shuangjingzi	Two-mica granite	118.15	43.26	248	1.0	LA-ICP-MS,U-Pb	Zircon	Li Shan et al.	2017	Journal of Geophysical Research: Solid Earth
1417	Outside of the MOO	Xilamulun	LX913-10	Chuangjiayingzi	Monzogranite	118.18	43.81	245	3.0	LA-ICP-MS,U-Pb	Zircon	Li Shan et al.	2017	Journal of Geophysical Research: Solid Earth
1418	Outside of the MOO	Xilamulun	XF915-4.1	Jianshexi	Tonalite	118.75	43.86	245	4.0	SHRIMP,U-Pb	Zircon	Li Shan et al.	2017	Journal of Geophysical Research: Solid Earth
1419	Outside of the MOO	Xilamulun	11XF-6	Jianshedong	Granodorite	118.85	43.88	250	2.0	LA-ICP-MS,U-Pb	Zircon	Li Shan et al.	2017	Journal of Geophysical Research: Solid Earth
1420	Outside of the MOO	Xilamulun	11XL-4.1	Xinlinzhen	Granodorite	117.82	43.97	251	3.0	SHRIMP,U-Pb	Zircon	Li Shan et al.	2017	Journal of Geophysical Research: Solid Earth
1421	Outside of the MOO	Xilamulun	8875	Fangkuangzi	granitic gneiss	118.24	43.35	272	2.0	LA-ICP-MS,U-Pb	Zircon	Li Y.L et al.	2014	Lithos.
1422	Outside of the MOO	Xilamulun	8877	Fangkuangzi	granitic gneiss	118.25	43.33	265	2.0	LA-ICP-MS,U-Pb	Zircon	Li Y.L et al.	2014	Lithos.
1423	Outside of the MOO	Xilamulun	2228802	Daijiawopu	granitine	118.27	43.38	270	1.0	LA-ICP-MS,U-Pb	Zircon	Li Y.L et al.	2014	Lithos.
1424	Outside of the MOO	Xilamulun	010-7	Yuejin	granodiorite vein	115.96	43.67	302	2.0	LA-ICP-MS,U-Pb	Zircon	Li Y.L et al.	2014	Lithos.
1425	Outside of the MOO	Xilamulun	801	Buliemiao	Mylonitic granodiorite	116.72	43.78	263	3.0	LA-ICP-MS,U-Pb	Zircon	Li Y.L et al.	2014	Lithos.
1426	Outside of the MOO	Xilamulun	8875	Room frame ditch	Granitic gneiss	118.32	43.32	272	1.6	LA-ICP-MS,U-Pb	Zircon	Li Yilong et al.	2009	Earth Science (ICWEA).
1427	Outside of the MOO	Xilamulun	8877	Room frame ditch	Granitic gneiss	118.33	43.31	265	1.8	LA-ICP-MS,U-Pb	Zircon	Li Yilong et al.	2009	Earth Science (ICWEA).
1428	Outside of the MOO	Xilamulun	10-110-8	Bayan Obo	Granite	110.08	41.77	262	5.8	LA-ICP-MS,U-Pb	Zircon	Ling Mingxing et al.	2014	Lithos.
1429	Outside of the MOO	Xilamulun	10-110-6	Bayan Obo	Quartz monzonite	110.08	41.77	263	4.3	LA-ICP-MS,U-Pb	Zircon	Ling Mingxing et al.	2014	Lithos.
1430	Outside of the MOO	Xilamulun	10-110-1	Bayan Obo	Quartz monzonite	110.08	41.77	265	3.4	LA-ICP-MS,U-Pb	Zircon	Ling Mingxing et al.	2014	Lithos.
1431	Outside of the MOO	Xilamulun	10-110-7	Bayan Obo	Quartz monzonite	110.08	41.77	267	3.9	LA-ICP-MS,U-Pb	Zircon	Ling Mingxing et al.	2014	Lithos.
1432	Outside of the MOO	Xilamulun	07.110-3.1	Bayan Obo	Granite	110.08	41.77	267	2.8	LA-ICP-MS,U-Pb	Zircon	Ling Mingxing et al.	2014	Lithos.
1433	Outside of the MOO	Xilamulun	10-110-3	Bayan Obo	Granite	110.08	41.77	267	2.8	LA-ICP-MS,U-Pb	Zircon	Ling Mingxing et al.	2014	Lithos.
1434	Outside of the MOO	Xilamulun	10-110-4	Bayan Obo	Granite	110.08	41.77	268	10.0	LA-ICP-MS,U-Pb	Zircon	Ling Mingxing et al.	2014	Lithos.
1435	Outside of the MOO	Xilamulun	10-110-5	Bayan Obo	Quartz monzonite	110.08	41.77	272	5.3	LA-ICP-MS,U-Pb	Zircon	Ling Mingxing et al.	2014	Lithos.
1436	Outside of the MOO	Xilamulun	10-110-2	Bayan Obo	Granite	110.08	41.77	273	8.1	LA-ICP-MS,U-Pb	Zircon	Ling Mingxing et al.	2014	Lithos.
1437	Outside of the MOO	Xilamulun	BJG1	Arctic rock masses	Granite	111.33	41.63	264	3.4	LA-ICP-MS,U-Pb	Zircon	Liu Changfeng et al.	2010	Geoscience (ICWEA).
1438	Outside of the MOO	Xilamulun	GS1	Amawusu Granodiorite	Granidiorite	112.35	41.97	267	9.0	LA-ICP-MS,U-Pb	Zircon	Liu Changfeng et al.	2010	Acta Geologica Sinica (ICWEA).
1439	Outside of the MOO	Xilamulun	GS2	Broong monzonitic granite	Monzogranite	112.38	42.00	239	4.0	LA-ICP-MS,U-Pb	Zircon	Liu Changfeng et al.	2010	Acta Geologica Sinica (ICWEA).
1440	Outside of the MOO	Xilamulun	WET14	Wuerta Gaole Temple Rock Ma	Monzogranite	112.15	42.44	256	3.4	LA-ICP-MS,U-Pb	Zircon	Liu Changfeng et al.	2010	Mineralogy and Petrology (ICWEA).
1441	Outside of the MOO	Xilamulun	WET19	Uerta Gaole Temple Rock Mas	Syenogranite	112.15	42.53	261	2.0	LA-ICP-MS,U-Pb	Zircon	Liu Changfeng et al.	2010	Mineralogy and Petrology (ICWEA).
1442	Outside of the MOO	Xilamulun	GS3	Geertu muscovite syenite granit	Syenogranite	112.30	42.89	238	6.0	LA-ICP-MS,U-Pb	Zircon	Liu Changfeng et al.	2010	Acta Geologica Sinica (ICWEA).
1443	Outside of the MOO	Xilamulun	GS4	Geertu muscovite syenite granit	Monzogranite dike	112.38	42.93	224	8.0	LA-ICP-MS,U-Pb	Zircon	Liu Changfeng et al.	2010	Acta Geologica Sinica (ICWEA).
1444	Outside of the MOO	Xilamulun	DM-1	Damiao pluton	Granodiorite	111.51	41.04	261	4.0	LA-ICP-MS,U-Pb	Zircon	Liu Changfeng et al.	2015	Lithos.
1445	Outside of the MOO	Xilamulun	FLS-1	Fulengshan pluton	K-feldspar granite	111.95	41.34	235	2.0	LA-ICP-MS,U-Pb	Zircon	Liu Changfeng et al.	2015	Lithos.
1446	Outside of the MOO	Xilamulun	XLCB-1	Xielichabu Pluton	Granodiorite	111.90	41.38	261	5.0	LA-ICP-MS,U-Pb	Zircon	Liu Changfeng et al.	2015	Lithos.
1447	Outside of the MOO	Xilamulun	DJP-1	Dajingpo Pluton	Monzonitic granite	111.33	41.78	271	2.0	LA-ICP-MS,U-Pb	Zircon	Liu Changfeng et al.	2015	Lithos.
1448	Outside of the MOO	Xilamulun	SQ-1	Saqi pluton	Syenogranite	111.84	42.07	254	5.0	LA-ICP-MS,U-Pb	Zircon	Liu Changfeng et al.	2015	Lithos.
1449	Outside of the MOO	Xilamulun	Zk2805 125m	Bilihe gold deposit	Dacite porphyry	113.54	42.39	259	3.0	LA-ICP-MS,U-Pb	Zircon	Liu Changfeng et al.	2015	Lithos.
1450	Outside of the MOO	Xilamulun	14L3-5	Bilihe gold deposit	Dacite porphyry	113.54	42.39	261	2.0	LA-ICP-MS,U-Pb	Zircon	Liu Changfeng et al.	2015	Lithos.
1451	Outside of the MOO	Xilamulun	Zk2805 103m	Bilihe gold deposit	Dacite porphyry	113.54	42.39	266	2.0	LA-ICP-MS,U-Pb	Zircon	Liu Changfeng et al.	2015	Lithos.
1452	Outside of the MOO	Xilamulun	Zk2805 76m	Bilihe gold deposit	Dacite porphyry	113.54	42.39	267	3.0	LA-ICP-MS,U-Pb	Zircon	Liu Changfeng et al.	2015	Lithos.
1453	Outside of the MOO	Xilamulun	Zk2805 55m	Bilihe gold deposit	Granodiorite	113.54	42.39	268	2.0	LA-ICP-MS,U-Pb	Zircon	Liu Changfeng et al.	2015	Lithos.
1454	Outside of the MOO	Xilamulun	Zk2805 94m	Bilihe gold deposit	Dacite porphyry	113.54	42.39	269	3.0	LA-ICP-MS,U-Pb	Zircon	Liu Changfeng et al.	2015	Lithos.
1455	Outside of the MOO	Xilamulun	HB145-2	Wanshigou Rock Mass	Syenogranite	114.15	41.11	239	4.4	LA-ICP-MS,U-Pb	Zircon	Liu Changyou et al.	2014	Beijing: China University of Geosciences, Beijing.
1456	Outside of the MOO	Xilamulun	NM05	Shanda Rock	Syenogranite	110.20	41.42	241	2.9	SHRIMP,U-Pb	Zircon	Liu Changyou et al.	2014	Beijing: China University of Geosciences, Beijing.
1457	Outside of the MOO	Xilamulun	NM104	Sangu Xicun Rock Mass	Alkali-feldspar granite	113.15	41.42	222	2.6	SHRIMP,U-Pb	Zircon	Liu Changyou et al.	2014	Beijing: China University of Geosciences, Beijing.
1458	Outside of the MOO	Xilamulun	NM110	Sangu Xicun Rock Mass	Alkali-feldspar granite	113.15	41.42	232	2.5	SHRIMP,U-Pb	Zircon	Liu Changyou et al.	2014	Beijing: China University of Geosciences, Beijing.
1459	Outside of the MOO	Xilamulun	HB08	New house rock mass	Alkali-feldspar granite	113.25	41.44	250	16.0	LA-ICP-MS,U-Pb	Zircon	Liu Changyou et al.	2014	Beijing: China University of Geosciences, Beijing.
1460	Outside of the MOO	Xilamulun	HB127-2	Jiangjialiang Pluton	Alkali-feldspar granite	114.51	41.46	246	4.4	LA-ICP-MS,U-Pb	Zircon	Liu Changyou et al.	2014	Beijing: China University of Geosciences, Beijing.
1461	Outside of the MOO	Xilamulun	HB06	Zhong Aliwusu Pluton	Quartz-syenite	113.14	41.60	251	6.2	LA-ICP-MS,U-Pb	Zircon	Liu Changyou et al.	2014	Beijing: China University of Geosciences, Beijing.
1462	Outside of the MOO	Xilamulun	NMH13-1	Zhangerlinzi Granite	Alkali-feldspar granite	113.78	41.77	238	3.0	LA-ICP-MS,U-Pb	Zircon	Liu Changyou et al.	2014	Beijing: China University of Geosciences, Beijing.
1463	Outside of the MOO	Xilamulun	NM82	Wenjiacun Rock Mass	Alkali-feldspar granite	113.63	41.82	228	2.9	SHRIMP,U-Pb	Zircon	Liu Changyou et al.	2014	Beijing: China University of Geosciences, Beijing.

1464	Outside of the MOO	Xilamulun	HB89-2	Daolahudong Village Rock Ma	Alkali-feldspar granite	113.95	41.91	224	17.0	LA-ICP-MS,U-Pb	Zircon	Liu Changyou et al.	2014	Beijing: China University of Geosciences, Beijing.
1465	Outside of the MOO	Xilamulun	WL60420	Longtou Mountain1	Monzogranite	118.14	43.94	226	4.0	TIMS,U-Pb	Zircon	Liu et al.	2005	Chemical Geology.
1466	Outside of the MOO	Xilamulun	06Y37	Yuan Baoshan	Quartz-Monzonite	118.76	42.46	269	3.0	LA-ICP-MS,U-Pb	Zircon	Liu J et al.	2010	Gondwana Research
1467	Outside of the MOO	Xilamulun	JG21-38-72	Daqihundi Pluton	Quartz-diorite	117.55	44.13	325	3.0	LA-ICP-MS,U-Pb	Zircon	Liu Jianfeng et al.	2009	Chang chun: Jilin University.
1468	Outside of the MOO	Xilamulun	XW21-30	Baiyin Tolgoi Pluton	Dimica granite	117.45	44.13	317	2.1	LA-ICP-MS,U-Pb	Zircon	Liu Jianfeng et al.	2009	Chang chun: Jilin University.
1469	Outside of the MOO	Xilamulun	XW4-19	Benbatu Group 1	Foliated basalt	117.71	44.16	318	3.4	LA-ICP-MS,U-Pb	Zircon	Liu Jianfeng et al.	2009	Chang chun: Jilin University.
1470	Outside of the MOO	Xilamulun	JG21-42-79	Venus Pluton	Quartz-diorite	117.83	44.27	322	3.0	LA-ICP-MS,U-Pb	Zircon	Liu Jianfeng et al.	2009	Acta Geologica Sinica (ICWEA).
1471	Outside of the MOO	Xilamulun	JG21-43-18	Dashizhai Formation	Rhyolite	117.71	44.39	274	2.8	LA—ICPMS	Zircon	Liu Jianfeng et al.	2009	Chang chun: Jilin University.
1472	Outside of the MOO	Xilamulun	ZL45-2	Bayar Tuhusuo	Gabbro	120.59	45.03	274	1.5	LA-ICP-MS,U-Pb	Zircon	Liu Jianfeng et al.	2009	Chang chun: Jilin University.
1473	Outside of the MOO	Xilamulun	ZL45-1	Bayar Tuhusuo	Gabbro	120.59	45.03	275	1.7	LA-ICP-MS,U-Pb	Zircon	Liu Jianfeng et al.	2009	Chang chun: Jilin University.
1474	Outside of the MOO	Xilamulun	ZL33-01	Benbatu Formation	Volcanic tuff	120.39	45.13	324	3.5	LA-ICP-MS,U-Pb	Zircon	Liu Jianfeng et al.	2009	Chang chun: Jilin University.
1475	Outside of the MOO	Xilamulun	JG21-29-58	Jiantun rock mass	Granidiorite	118.81	43.89	249	2.3	LA-ICP-MS,U-Pb	Zircon	Liu Jianfeng et al.	2013	Acta Petrologica Sinica (ICWEA).
1476	Outside of the MOO	Xilamulun	AST01-1	Zhuanshanzi	Granidiorite	117.48	43.84	246	0.9	LA-ICP-MS,U-Pb	Zircon	Liu Jianfeng et al.	2014	Acta Geologica Sinica (ICWEA).
1477	Outside of the MOO	Xilamulun	PM003-1	Mongolia Yingzi	Granidiorite	119.92	42.21	253	2.4	LA-ICP-MS,U-Pb	Zircon	Liu Jue et al.	2015	Geological Bulletin of China (ICWEA).
1478	Outside of the MOO	Xilamulun	H30-19	Hadamiao area	Diorite	113.63	42.31	267	3.0	SHRIMP,U-Pb	Zircon	Liu Jun et al.	2014	Acta Petrologica Sinica (ICWEA).
1479	Outside of the MOO	Xilamulun	HK3	Hadamiao area	Granite porphyry	113.63	42.31	271	3.0	SHRIMP,U-Pb	Zircon	Liu Jun et al.	2014	Acta Petrologica Sinica (ICWEA).
1480	Outside of the MOO	Xilamulun	WL53063	Xiaochengzi	Biotite granodiorite and Monzogran	117.92	43.48	146	4.8	SHRIMP,U-Pb	Zircon	Liu W et al.	2009	International Journal of Earth Sciences.
1481	Outside of the MOO	Xilamulun		Ma'anzi	Biotite syenogranite and Monzogran	118.07	43.80	146	3.7	SHRIMP,U-Pb	Zircon	Liu W et al.	2009	International Journal of Earth Sciences.
1482	Outside of the MOO	Xilamulun	LW97066	Ma'anzi	Biotite syenogranite and Monzogran	118.07	43.80	166	2.4	SHRIMP,U-Pb	Zircon	Liu W et al.	2009	International Journal of Earth Sciences.
1483	Outside of the MOO	Xilamulun		Longtou Mountain2	Hornblastic granite diorite and Mon	118.24	43.83	241	3.2	SHRIMP,U-Pb	Zircon	Liu W et al.	2009	International Journal of Earth Sciences.
1484	Outside of the MOO	Xilamulun	LW97066	Maanshan	Granite	118.07	43.80	146	3.7	SHRIMP,U-Pb	Zircon	Liu Wei et al.	2007	Acta Petrologica Sinica (ICWEA).
1485	Outside of the MOO	Xilamulun	LW97077	Yongdusan 1	Granite	118.19	43.81	241	3.2	SHRIMP,U-Pb	Zircon	Liu Wei et al.	2007	Acta Petrologica Sinica (ICWEA).
1486	Outside of the MOO	Xilamulun	HG-05	Huangtuliang Gold Mine	Monzogranite	115.66	40.95	245	2.0	LA-ICP-MS,U-Pb	Zircon	Liu Xiaoyu et al.	2014	Shijiazhuang: Hebei GEO University
1487	Outside of the MOO	Xilamulun	NL07-15	muhanbasitao	Hornblende gabbro	82.46	43.61	317	2.2	LA-ICP-MS,U-Pb	Zircon	Liu Xin et al.	2011	Acta Petrologica Sinica (ICWEA)
1488	Outside of the MOO	Xilamulun	CHS-21	Haoyaoerhudong	amphibole olivine gabbro	109.28	41.68	182	1.0	LA-ICP-MS,U-Pb	Zircon	Liu Y F et al.	2014	Ore Geology Reviews
1489	Outside of the MOO	Xilamulun	CSH-9	Haoyaoerhudong	amphibole olivine gabbro	109.27	41.72	282	1.0	LA-ICP-MS,U-Pb	Zircon	Liu Y F et al.	2014	Ore Geology Reviews
1490	Outside of the MOO	Xilamulun	CSH-3	Haoyaoerhudong	porphyritic diorite	109.27	41.72	288	3.0	LA-ICP-MS,U-Pb	Zircon	Liu Y F et al.	2014	Ore Geology Reviews
1491	Outside of the MOO	Xilamulun	BS-13	Bayern Daba	Quartz-diorite	117.55	44.11	327	1.6	SHRIMP,U-Pb	Zircon	Liu Yifei et al.	2010	Geological Bulletin of China (ICWEA).
1492	Outside of the MOO	Xilamulun	72601	Sheshen cliff rock mass	Granite	115.76	41.07	228	3.1	SHRIMP,U-Pb	Zircon	Liu Yin et al.	2010	Chengdu: Chengdu University of Technology
1493	Outside of the MOO	Xilamulun	BL-4	Bogdawula mining area	Granite（containing mine）	114.48	44.08	241	2.5	SHRIMP,U-Pb	Zircon	Liu Yong et al.	2012	Acta Petrologica Sinica (ICWEA).
1494	Outside of the MOO	Xilamulun	09WJ-GTHF-110	Bilich Gold Mine	Potash granite porphyry	113.56	42.40	264		LA-ICP-MS,U-Pb	Zircon	Lu Yanming et al.	2012	Acta Petrologica Sinica (ICWEA).
1495	Outside of the MOO	Xilamulun	09Y008-1	Bilich Gold Mine	Monzonitic granite porphyry	113.56	42.40	280	6.8	LA-ICP-MS,U-Pb	Zircon	Lu Yanming et al.	2012	Acta Petrologica Sinica (ICWEA).
1496	Outside of the MOO	Xilamulun	09B-1	Bilich Gold Mine	Granodiorite	113.56	42.40	284	4.2	LA-ICP-MS,U-Pb	Zircon	Lu Yanming et al.	2012	Acta Petrologica Sinica (ICWEA).
1497	Outside of the MOO	Xilamulun		Hadamiao Gold Mine	Granite porphyry	113.62	42.31	272	3.3	LA-ICP-MS,U-Pb	Zircon	Lu Yinghuai et al.	2009	Acta Petrologica Sinica (ICWEA).
1498	Outside of the MOO	Xilamulun	BY06-30	Bayan Obo Bayinzhurihe	quartz diorite	109.55	41.80	265	2.0	LA-ICP-MS,U-Pb	Zircon	Luo Hongling et al.	2013	Geological journal of China Universities
1499	Outside of the MOO	Xilamulun	BP-16	Luanping Rock Mass	Globular diorite	117.20	41.01	284	8.0	LA-ICP-MS,U-Pb	Zircon	Ma Fang et al.	2004	Geological Review (ICWEA).
1500	Outside of the MOO	Xilamulun	D3014-TW1	Dora Gato	Quartz-diorite	117.60	44.18	305	0.8	LA-ICP-MS,U-Pb	Zircon	Ma Shiwei et al.	2013	Beijing: China University of Geosciences, Beijing.
1501	Outside of the MOO	Xilamulun	PM105-4TW1	Dora Gato	Quartz-diorite	117.69	44.25	312	1.9	LA-ICP-MS,U-Pb	Zircon	Ma Shiwei et al.	2013	Beijing: China University of Geosciences, Beijing.
1502	Outside of the MOO	Xilamulun	D3021-TW1	Dora Gato	Quartz-diorite	117.70	44.23	310	0.9	LA-ICP-MS,U-Pb	Zircon	Ma Shiwei et al.	2013	Beijing: China University of Geosciences, Beijing.
1503	Outside of the MOO	Xilamulun	PM105-17TW2	Dora Gato	Diorite	117.71	44.25	319	1.5	LA-ICP-MS,U-Pb	Zircon	Ma Shiwei et al.	2013	Beijing: China University of Geosciences, Beijing.
1504	Outside of the MOO	Xilamulun	D3020-TW1	Dora Gato	Diorite	117.68	44.25	329	1.1	LA-ICP-MS,U-Pb	Zircon	Ma Shiwei et al.	2013	Beijing: China University of Geosciences, Beijing.
1505	Outside of the MOO	Xilamulun	D4008-TW1	Venus Pluton	Quartz-diorite	117.84	44.25	313	1.2	LA-ICP-MS,U-Pb	Zircon	Ma Shiwei et al.	2013	Beijing: China University of Geosciences, Beijing.
1506	Outside of the MOO	Xilamulun	PM103-3TW1	Golden River Farm	Gabbro	117.92	44.27	321	2.0	LA-ICP-MS,U-Pb	Zircon	Ma Shiwei et al.	2013	Beijing: China University of Geosciences, Beijing.
1507	Outside of the MOO	Xilamulun	BLN-5-2	Porono Rock	Quartz-diorite	117.42	41.08	296	4.0	SHRIMP,U-Pb	Zircon	Ma Xu et al.	2012	Scientia Sinica(Terrae) (ICWEA).
1508	Outside of the MOO	Xilamulun	D1087	Panshan	Quartz-Monzonite dike	117.19	41.09	206	2.0	SHRIMP,U-Pb	Zircon	Ma Yinsheng et al.	2007	Acta Petrologica Sinica (ICWEA).
1509	Outside of the MOO	Xilamulun	By98228	Hanjiadian	Quartz monzonite	117.17	41.63	250	4.0	TIMS,U-Pb	Zircon	Mao Debao et al.	2003	Acta Petrologica Sinica (ICWEA).
1510	Outside of the MOO	Xilamulun	By99087	Hanjiadian	Syenogranite	117.10	41.67	247	3.0	TIMS,U-Pb	Zircon	Mao Debao et al.	2003	Acta Petrologica Sinica (ICWEA).
1511	Outside of the MOO	Xilamulun	By98046	Hanjiadian	Monzogranite	117.13	41.68	247	2.0	TIMS,U-Pb	Zircon	Mao Debao et al.	2003	Acta Petrologica Sinica (ICWEA).
1512	Outside of the MOO	Xilamulun	HG-68-1	Huanggang	K-feldspar granite	117.48	43.64	145	1.6	LA-ICP-MS,U-Pb	Zircon	Mei W et al.	2014	Geosciences Journa
1513	Outside of the MOO	Xilamulun	SZW07-15	Xihouhaozi Pluton	Granite	111.72	41.80	266	3.4	SIMS,U-Pb	Zircon	Meng Qingpeng et al.	2013	Geological Bulletin of China (ICWEA).
1514	Outside of the MOO	Xilamulun	GZZ-1	Guzuizi	Monzogranite	115.21	40.83	236	2.0	SHRIMP,U-Pb	Zircon	Miao et al.	2002	Ore Geology Reviews
1515	Outside of the MOO	Xilamulun	21NMG-105	Baolige	Monzogranite	117.46	42.18	296	7.0	SHRIMP,U-Pb	Zircon	Miao et al.	2003	Chinese Science Bulletin
1516	Outside of the MOO	Xilamulun	Hu09-20	Mount Daqing	biotite granite	111.84	41.06	275	1.0	LA-ICP-MS,U-Pb	Zircon	Mo Nan et al.	2014	Acta Scientiarum Naturalium Universitatis Pekinensis(ICWEA)
1517	Outside of the MOO	Xilamulun	D13LXXL02	Linxi County, Xinlin Town	Granidiorite	118.03	43.94	255	3.0	LA-ICP-MS,U-Pb	Zircon	Northeast group project self-te		
1518	Outside of the MOO	Xilamulun	D13LXXL01	Linxi County, Xinlin Town	Granidiorite	118.03	43.93	259	3.3	LA-ICP-MS,U-Pb	Zircon	Northeast group project self-te		
1519	Outside of the MOO	Xilamulun	D4013TW1	Qianjin field	Fine grained Bi-granidiorite	117.92	44.20	283		LA-ICP-MS,U-Pb	Zircon	Pei Weixun et al.	2014	Beijing: China University of Geosciences, Beijing.
1520	Outside of the MOO	Xilamulun	PM102-41TW1	Qianjin field	Medium fine-grained Diorite	117.98	44.20	313	1.7	LA-ICP-MS,U-Pb	Zircon	Pei Weixun et al.	2014	Beijing: China University of Geosciences, Beijing.
1521	Outside of the MOO	Xilamulun	PM102-30TW1	Qianjin field	Diorite porphyrite	117.99	44.21	280	2.0	LA-ICP-MS,U-Pb	Zircon	Pei Weixun et al.	2014	Beijing: China University of Geosciences, Beijing.
1522	Outside of the MOO	Xilamulun	PM102-25TW1	Qianjin field	Hybrid Fine grained Bi-granidiorite	117.99	44.21	301	0.9	LA-ICP-MS,U-Pb	Zircon	Pei Weixun et al.	2014	Beijing: China University of Geosciences, Beijing.
1523	Outside of the MOO	Xilamulun	PM102-15TW1	Qianjin field	Medium biotite potassium feldspar	117.99	44.23	280	1.0	LA-ICP-MS,U-Pb	Zircon	Pei Weixun et al.	2014	Beijing: China University of Geosciences, Beijing.
1524	Outside of the MOO	Xilamulun	WB08-N3	Narenwula Rock	Alkali-feldspar granite	114.51	42.42	145	1.3	LA-ICP-MS,U-Pb	Zircon	Qin Ya et al.	2012	Journal of Jilin University(Earth Science Edition) (ICWEA).
1525	Outside of the MOO	Xilamulun	MX16	Xilinhot A-type granite body	Miarolitic Granite	116.19	43.87	276	2.0	SHRIMP,U-Pb	Zircon	Shi Guanghai et al.	2004	Chinese Science Bulletin (ICWEA).
1526	Outside of the MOO	Xilamulun	MS3-2	Suzuoqi area	Granite	113.56	43.55	204	12.0	SHRIMP,U-Pb	Zircon	Shi Yuruo et al.	2004	Acta Geologica Sinica (ICWEA).
1527	Outside of the MOO	Xilamulun	MS4-1	Suzuoqi area	Monzogranite	113.87	43.84	222	4.0	SHRIMP,U-Pb	Zircon	Shi Yuruo et al.	2004	Acta Geologica Sinica (ICWEA).
1528	Outside of the MOO	Xilamulun	MS3-1	Baoerhan Lama Temple	High potassium peraluminous grani	113.66	43.57	222	6.0	SHRIMP,U-Pb	Zircon	Shi Yuruo et al.	2005	Beijing: Chinese Academy of Geological Sciences.
1529	Outside of the MOO	Xilamulun	MB1-2	Sonidzuoqi area	Granite	114.89	43.73	297	2.0	SHRIMP,U-Pb	Zircon	Shi Yuruo et al.	2016	Journal of Asian Earth Sciences
1530	Outside of the MOO	Xilamulun	MB1-1	Sonidzuoqi area	Granodiorite	114.89	43.73	320	8.0	SHRIMP,U-Pb	Zircon	Shi Yuruo et al.	2016	Journal of Asian Earth Sciences
1531	Outside of the MOO	Xilamulun	MB1-5	Sonidzuoqi area	Quartz-diorite	114.89	43.73	320	3.0	SHRIMP,U-Pb	Zircon	Shi Yuruo et al.	2016	Journal of Asian Earth Sciences
1532	Outside of the MOO	Xilamulun	MB1-6	Sonidzuoqi area	Tonalite	114.89	43.73	329	3.0	SHRIMP,U-Pb	Zircon	Shi Yuruo et al.	2016	Journal of Asian Earth Sciences
1533	Outside of the MOO	Xilamulun	BYN31	Hercynian intrusions	granodiorite	118.88	44.45	273	4.4	LA-ICP-MS,U-Pb	Zircon	Shu Q H et al.	2013	Economic Geology
1534	Outside of the MOO	Xilamulun	BYN37	Hercynian intrusions	granite	118.88	44.45	275	2.4	LA-ICP-MS,U-Pb	Zircon	Shu Q H et al.	2013	Economic Geology
1535	Outside of the MOO	Xilamulun	3P4TW14-2	Mandula	Gabbro	110.37	42.37	280	1.0	TIMS,U-Pb	Zircon	Tao Jixiong et al.	2003	Geological Survey and Research (ICWEA).
1536	Outside of the MOO	Xilamulun	M1288-2.1			111.40	43.73	304	3.0	LA-ICP-MS,U-Pb	Zircon	This study		
1537	Outside of the MOO	Xilamulun	M1497-12.2			110.11	43.72	307	3.0	LA-ICP-MS,U-Pb	Zircon	This study		
1538	Outside of the MOO	Xilamulun	M1288-1.1			111.37	43.70	275	1.0	LA-ICP-MS,U-Pb	Zircon	This study		
1539	Outside of the MOO	Xilamulun	M1288-1.7		Coarse Monzogranite	111.37	43.70	320	1.0	LA-ICP-MS,U-Pb	Zircon	This study		
1540	Outside of the MOO	Xilamulun	N14624-2.1			111.48	43.37	276	2.0	LA-ICP-MS,U-Pb	Zircon	This study		
1541	Outside of the MOO	Xilamulun	N14625-10.1			111.16	43.24	279	1.0	LA-ICP-MS,U-Pb	Zircon	This study		

1542	Outside of the MOO	Xilamulun	N14616-3.1			110.20	41.69	160	5.0	LA-ICP-MS,U-Pb	Zircon	This study		
1543	Outside of the MOO	Xilamulun	N14617-2.1			110.44	42.38	314	2.0	LA-ICP-MS,U-Pb	Zircon	This study		
1544	Outside of the MOO	Xilamulun	N14617-1.1			110.23	42.58	285	3.0	LA-ICP-MS,U-Pb	Zircon	This study		
1545	Outside of the MOO	Xilamulun	M12731-15.1			109.11	42.62	279	1.0	LA-ICP-MS,U-Pb	Zircon	This study		
1546	Outside of the MOO	Xilamulun	M12731-17.1			109.13	42.64	256	1.0	LA-ICP-MS,U-Pb	Zircon	This study		
1547	Outside of the MOO	Xilamulun	M12731-6.1			109.32	42.70	288	2.0	LA-ICP-MS,U-Pb	Zircon	This study		
1548	Outside of the MOO	Xilamulun	M12731-7.1			109.33	42.70	286	3.0	LA-ICP-MS,U-Pb	Zircon	This study		
1549	Outside of the MOO	Xilamulun	N14628-2.1			110.61	42.85	282	2.0	LA-ICP-MS,U-Pb	Zircon	This study		
1550	Outside of the MOO	Xilamulun	M12728-5.4			109.36	42.96	280	4.0	LA-ICP-MS,U-Pb	Zircon	This study		
1551	Outside of the MOO	Xilamulun	M1285-7			109.83	42.97	284	3.0	LA-ICP-MS,U-Pb	Zircon	This study		
1552	Outside of the MOO	Xilamulun	M1285-6.1			109.85	42.96	238	1.0	LA-ICP-MS,U-Pb	Zircon	This study		
1553	Outside of the MOO	Xilamulun	M1285-1.1			110.01	42.98	244	1.0	LA-ICP-MS,U-Pb	Zircon	This study		
1554	Outside of the MOO	Xilamulun	M14910-2.1			110.20	42.99	283	2.0	LA-ICP-MS,U-Pb	Zircon	This study		
1555	Outside of the MOO	Xilamulun	M12728-2.1			109.34	43.00	278	4.0	LA-ICP-MS,U-Pb	Zircon	This study		
1556	Outside of the MOO	Xilamulun	M12730-10.1			109.52	43.00	285	1.0	LA-ICP-MS,U-Pb	Zircon	This study		
1557	Outside of the MOO	Xilamulun	M12726-7.3			109.27	43.00	289	1.0	LA-ICP-MS,U-Pb	Zircon	This study		
1558	Outside of the MOO	Xilamulun	M12726-2			109.27	43.01	290	1.0	LA-ICP-MS,U-Pb	Zircon	This study		
1559	Outside of the MOO	Xilamulun	M12730-11.2			109.47	43.01	283	1.0	LA-ICP-MS,U-Pb	Zircon	This study		
1560	Outside of the MOO	Xilamulun	M14917-1.3			109.33	43.02	274	3.0	LA-ICP-MS,U-Pb	Zircon	This study		
1561	Outside of the MOO	Xilamulun	M12729-1.1			109.36	43.02	285	1.0	LA-ICP-MS,U-Pb	Zircon	This study		
1562	Outside of the MOO	Xilamulun	M12729-5.1			109.38	43.02	285	1.0	LA-ICP-MS,U-Pb	Zircon	This study		
1563	Outside of the MOO	Xilamulun	N14630-3.1			110.92	43.02	285	2.0	LA-ICP-MS,U-Pb	Zircon	This study		
1564	Outside of the MOO	Xilamulun	N14630-3.5			110.92	43.02	285	2.0	LA-ICP-MS,U-Pb	Zircon	This study		
1565	Outside of the MOO	Xilamulun	M14913-37.2			109.49	43.04	308	3.0	LA-ICP-MS,U-Pb	Zircon	This study		
1566	Outside of the MOO	Xilamulun	M12729-13.1			109.58	43.06	281	1.0	LA-ICP-MS,U-Pb	Zircon	This study		
1567	Outside of the MOO	Xilamulun	M12727-19.1			109.31	43.09	311	2.0	LA-ICP-MS,U-Pb	Zircon	This study		
1568	Outside of the MOO	Xilamulun	M14910-30.2			110.71	43.09	282	3.0	LA-ICP-MS,U-Pb	Zircon	This study		
1569	Outside of the MOO	Xilamulun	M14910-30.7			110.71	43.09	287	2.0	LA-ICP-MS,U-Pb	Zircon	This study		
1570	Outside of the MOO	Xilamulun	M12727-17.1			109.34	43.10	315	2.0	LA-ICP-MS,U-Pb	Zircon	This study		
1571	Outside of the MOO	Xilamulun	N14629-12.1			110.93	43.10	149	2.0	LA-ICP-MS,U-Pb	Zircon	This study		
1572	Outside of the MOO	Xilamulun	N14629-12.3			110.93	43.10	277	2.0	LA-ICP-MS,U-Pb	Zircon	This study		
1573	Outside of the MOO	Xilamulun	N14629-10.1		Biotite granite	110.96	43.10	154	6.0	LA-ICP-MS,U-Pb	Zircon	This study		
1574	Outside of the MOO	Xilamulun	N1471-4.1			111.23	43.11	145	1.0	LA-ICP-MS,U-Pb	Zircon	This study		
1575	Outside of the MOO	Xilamulun	N1471-3.1			111.23	43.11	146	1.0	LA-ICP-MS,U-Pb	Zircon	This study		
1576	Outside of the MOO	Xilamulun	N1471-42-4.3		Granite dike	111.23	43.11	238	2.0	LA-ICP-MS,U-Pb	Zircon	This study		
1577	Outside of the MOO	Xilamulun	N1471-4.2			111.23	43.11	238	2.0	LA-ICP-MS,U-Pb	Zircon	This study		
1578	Outside of the MOO	Xilamulun	N1471-3.4			111.23	43.11	291	3.0	LA-ICP-MS,U-Pb	Zircon	This study		
1579	Outside of the MOO	Xilamulun	M12727-15			109.33	43.13	315	2.0	LA-ICP-MS,U-Pb	Zircon	This study		
1580	Outside of the MOO	Xilamulun	M1286-5.1			110.08	43.13	289	3.0	LA-ICP-MS,U-Pb	Zircon	This study		
1581	Outside of the MOO	Xilamulun	M12727-14.5			109.29	43.14	316	3.0	LA-ICP-MS,U-Pb	Zircon	This study		
1582	Outside of the MOO	Xilamulun	M12727-5			109.17	43.16	250	1.0	LA-ICP-MS,U-Pb	Zircon	This study		
1583	Outside of the MOO	Xilamulun	M12727-8.1			109.21	43.16	251	2.0	LA-ICP-MS,U-Pb	Zircon	This study		
1584	Outside of the MOO	Xilamulun	M1284-17.1			110.02	43.20	234	1.0	LA-ICP-MS,U-Pb	Zircon	This study		
1585	Outside of the MOO	Xilamulun	M1284-10.1			110.08	43.23	220	1.0	LA-ICP-MS,U-Pb	Zircon	This study		
1586	Outside of the MOO	Xilamulun	M14911-6.1			110.37	43.24	237	3.0	LA-ICP-MS,U-Pb	Zircon	This study		
1587	Outside of the MOO	Xilamulun	M1284-9.1			110.06	43.26	220	1.0	LA-ICP-MS,U-Pb	Zircon	This study		
1588	Outside of the MOO	Xilamulun	M1499-18.1			109.82	43.27	302	6.0	LA-ICP-MS,U-Pb	Zircon	This study		
1589	Outside of the MOO	Xilamulun	M1284-8.1			110.04	43.28	222	1.0	LA-ICP-MS,U-Pb	Zircon	This study		
1590	Outside of the MOO	Xilamulun	M1284-7.1			110.04	43.28	326	1.0	LA-ICP-MS,U-Pb	Zircon	This study		
1591	Outside of the MOO	Xilamulun	M1499-40.1			110.30	43.28	238	3.0	LA-ICP-MS,U-Pb	Zircon	This study		
1592	Outside of the MOO	Xilamulun	M1284-5.1			110.05	43.31	226	2.0	LA-ICP-MS,U-Pb	Zircon	This study		
1593	Outside of the MOO	Xilamulun	M14911-8.1			110.43	43.32	302	3.0	LA-ICP-MS,U-Pb	Zircon	This study		
1594	Outside of the MOO	Xilamulun	M1499-13.1			109.59	43.31	358	3.0	LA-ICP-MS,U-Pb	Zircon	This study		
1595	Outside of the MOO	Xilamulun	M1499-11.1			109.60	43.33	294	3.0	LA-ICP-MS,U-Pb	Zircon	This study		
1596	Outside of the MOO	Xilamulun	M1499-35.1			110.24	43.33	260	2.0	LA-ICP-MS,U-Pb	Zircon	This study		
1597	Outside of the MOO	Xilamulun	M1499-10.6			109.63	43.34	214	2.0	LA-ICP-MS,U-Pb	Zircon	This study		
1598	Outside of the MOO	Xilamulun	M1498-4.1			109.64	43.61	223	3.0	LA-ICP-MS,U-Pb	Zircon	This study		
1599	Outside of the MOO	Xilamulun	WL60422	Yelaigai	Monzogranite	118.14	43.94	148	1.0	TIMS,U-Pb	Zircon	This study		
1600	Outside of the MOO	Xilamulun	05FW180	Telegut	Diorite porphyrite	117.12	43.75	287	3.0	LA-ICP-MS,U-Pb	Zircon	Tian Dexin et al.	2015	Chang chun: Jilin University.
1601	Outside of the MOO	Xilamulun	MXA12	Xilinhot	Garnet Granite	116.43	43.85	316	3.0	SHRIMP,U-Pb	Zircon	Tian Dexin et al.	2015	Chang chun: Jilin University.
1602	Outside of the MOO	Xilamulun	05FW174	Melindaba	Quartz-diorite	117.11	43.98	301	1.0	LA-ICP-MS,U-Pb	Zircon	Tian Dexin et al.	2015	Chang chun: Jilin University.
1603	Outside of the MOO	Xilamulun	XW15-24-2	Misheng Temple	Quartz-diorite	117.53	44.11	319	2.3	LA-ICP-MS,U-Pb	Zircon	Tian Dexin et al.	2015	Chang chun: Jilin University.
1604	Outside of the MOO	Xilamulun	XZJ	Xiaozhangjiakou basic-ultrabas	Olivine pyroxenite	115.70	40.88	220	5.0	SHRIMP,U-Pb	Zircon	Tian Wei et al.	2007	Acta Petrologica Sinica (ICWEA).
1605	Outside of the MOO	Xilamulun	99-1	Bayinchahan	Hornblastic granite diorite	113.54	42.06	262	6.1	LA-ICP-MS,U-Pb	Zircon	Tong Ying et al.	2010	Acta Geoscientica Sinica (ICWEA).
1606	Outside of the MOO	Xilamulun	99-6	Hada Temple	Biotite Quartz-diorite	113.28	42.15	277	8.5	LA-ICP-MS,U-Pb	Zircon	Tong Ying et al.	2010	Acta Geoscientica Sinica (ICWEA).
1607	Outside of the MOO	Xilamulun	99-20	Eastern Soviet Union	Monzogranite	113.87	43.83	217	5.4	LA-ICP-MS,U-Pb	Zircon	Tong Ying et al.	2010	Acta Geoscientica Sinica (ICWEA).
1608	Outside of the MOO	Xilamulun	E101-1.1	Baolag	Alkali granite	112.97	43.73	285	0.9	LA-ICP-MS,U-Pb	Zircon	Tong Ying et al.	2015	Journal of Asian Earth Sciences
1609	Outside of the MOO	Xilamulun	E930-1	Saiyinwusu	Alkali granite	112.35	43.68	279	0.9	LA-ICP-MS,U-Pb	Zircon	Tong Ying et al.	2015	Journal of Asian Earth Sciences
1610	Outside of the MOO	Xilamulun	Hq06	Baihuagou Rock Mass	Gneous Quartz-diorite	115.20	41.07	253	3.0	LA-ICP-MS,U-Pb	Zircon	Wang Fang et al.	2009	Acta Petrologica Sinica (ICWEA).
1611	Outside of the MOO	Xilamulun	Hq01	Hailiutu rock mass	Monzogranite	115.25	41.16	254	11.0	LA-ICP-MS,U-Pb	Zircon	Wang Fang et al.	2009	Acta Petrologica Sinica (ICWEA).
1612	Outside of the MOO	Xilamulun	ZL21C	Zhenningbao Rock Mass	Gneous Granite	115.25	41.16	287	1.0	LA-ICP-MS,U-Pb	Zircon	Wang Fang et al.	2009	Acta Petrologica Sinica (ICWEA).
1613	Outside of the MOO	Xilamulun	Hq33	Akagi Current Map	Monzogranite	115.09	41.17	299	3.0	LA-ICP-MS,U-Pb	Zircon	Wang Fang et al.	2009	Acta Petrologica Sinica (ICWEA).
1614	Outside of the MOO	Xilamulun	05A06	Labagoumen, Huairou County	Diorite	116.62	40.89	288	4.8	SHRIMP,U-Pb	Zircon	Wang Huichu et al.	2007	Acta Petrologica Sinica (ICWEA).
1615	Outside of the MOO	Xilamulun	05J05	Tianqiao Town, Fengning Cour	Biotite granite	116.98	40.99	280	5.6	SHRIMP,U-Pb	Zircon	Wang Huichu et al.	2007	Acta Petrologica Sinica (ICWEA).
1616	Outside of the MOO	Xilamulun	JN-35-1	Wenduer Temple South	Biotite hornblende quartz diorite	113.25	42.08	272	2.4	LA-ICP-MS,U-Pb	Zircon	Wang WanQiong et a	2013	Acta Petrologica Sinica (ICWEA)
1617	Outside of the MOO	Xilamulun	JN-56	Wenduer Temple South	Granidiorite	113.57	42.40	269	1.3	LA-ICP-MS,U-Pb	Zircon	Wang WanQiong et a	2013	Acta Petrologica Sinica (ICWEA)
1618	Outside of the MOO	Xilamulun	P37b8-2	Laoquangou	Hornblende Gabbro	112.54	41.21	297	1.7	LA-ICP-MS,U-Pb	Zircon	Wang Wanqiong et al	2014	Chang chun: Jilin University.
1619	Outside of the MOO	Xilamulun	JN71	Hadatu	Bi-Monzogranite	113.27	41.81	273	1.3	LA-ICP-MS,U-Pb	Zircon	Wang Wanqiong et al	2014	Chang chun: Jilin University.

1620	Outside of the MOO	Xilamulun	P02B16-2	Temple of windur	Medium coarseGranite	113.48	41.81	267	1.4	LA-ICP-MS,U-Pb	Zircon	Wang Wanqiong et al	2014	Chang chun: Jilin University.
1621	Outside of the MOO	Xilamulun	P38b2-1	Temple of windur	Medium fine-grained hornblastic gr	112.60	42.00	271	1.3	LA-ICP-MS,U-Pb	Zircon	Wang Wanqiong et al	2014	Chang chun: Jilin University.
1622	Outside of the MOO	Xilamulun	JN35	Bainai Temple	Quartz-diorite	112.96	42.13	272	2.4	LA-ICP-MS,U-Pb	Zircon	Wang Wanqiong et al	2014	Chang chun: Jilin University.
1623	Outside of the MOO	Xilamulun	WL119	Villasto	Granidiorite	117.46	44.07	298	2.5	SHRIMP,U-Pb	Zircon	Wang Xinyu et al.	2013	Geoscience (ICWEA).
1624	Outside of the MOO	Xilamulun	WL89	Villasto	Quartz-diorite	117.46	44.07	308	4.2	SHRIMP,U-Pb	Zircon	Wang Xinyu et al.	2013	Geoscience (ICWEA).
1625	Outside of the MOO	Xilamulun	WL212	Villasto	Bi-Monzogranite	117.46	44.07	314	3.4	SHRIMP,U-Pb	Zircon	Wang Xinyu et al.	2013	Geoscience (ICWEA).
1626	Outside of the MOO	Xilamulun	WL95	Villasto	Biotite granite	117.46	44.07	321	4.1	SHRIMP,U-Pb	Zircon	Wang Xinyu et al.	2013	Geoscience (ICWEA).
1627	Outside of the MOO	Xilamulun	CF06-042	Chaoyanggou	Gneissic Granite	119.05	41.94	150	1.0	SHRIMP,U-Pb	Zircon	Wang Yanbin et al.	2010	Acta Petrologica et Mineralogica (ICWEA).
1628	Outside of the MOO	Xilamulun	CF06-065-2	Zhalanyingzi	Gneissic Granite	119.06	41.97	254	1.2	SHRIMP,U-Pb	Zircon	Wang Yanbin et al.	2010	Acta Petrologica et Mineralogica (ICWEA).
1629	Outside of the MOO	Xilamulun	ZK-015	Sadaigoumen porphyry molybd	Potassic monzogranite	116.60	41.27	248	1.2	LA-ICP-MS,U-Pb	Zircon	Wei Ran et al.	2013	Geology in China (ICWEA).
1630	Outside of the MOO	Xilamulun	SD-006	Sadaigoumen porphyry molybd	Monzogranite	116.60	41.27	257	0.9	LA-ICP-MS,U-Pb	Zircon	Wei Ran et al.	2013	Geology in China (ICWEA).
1631	Outside of the MOO	Xilamulun	SD-008	Sadaigoumen porphyry molybd	Potassic monzogranite	116.60	41.27	257	0.9	LA-ICP-MS,U-Pb	Zircon	Wei Ran et al.	2013	Geology in China (ICWEA).
1632	Outside of the MOO	Xilamulun	FW02-170	Yiwu Jianshan	Granodiorite enclave	121.70	41.58	153		LA-ICP-MS,U-Pb	Zircon	Wu Fuyuan et al.	2006	Acta Petrologica Sinica (ICWEA)
1633	Outside of the MOO	Xilamulun	FW02-168	Yiwu Jianshan	Two-mica granite	121.70	41.58	163		LA-ICP-MS,U-Pb	Zircon	Wu Fuyuan et al.	2006	Acta Petrologica Sinica (ICWEA)
1634	Outside of the MOO	Xilamulun	FW02-156	Jianlazi	Two-mica granite	121.47	41.59	169		LA-ICP-MS,U-Pb	Zircon	Wu Fuyuan et al.	2006	Acta Petrologica Sinica (ICWEA)
1635	Outside of the MOO	Xilamulun	FW02-158	Yangjiazhangzi pluton	Granite dyke	121.47	41.59	182		LA-ICP-MS,U-Pb	Zircon	Wu Fuyuan et al.	2006	Acta Petrologica Sinica (ICWEA)
1636	Outside of the MOO	Xilamulun	FW02-151	Jianlazi	Two-mica granite	121.44	41.63	154		LA-ICP-MS,U-Pb	Zircon	Wu Fuyuan et al.	2006	Acta Petrologica Sinica (ICWEA)
1637	Outside of the MOO	Xilamulun	03JH047	Haitang Mountain	Granite	121.81	41.98	176	1.0	LA-ICP-MS,U-Pb	Zircon	Wu Fuyuan et al.	2006	Acta Petrologica Sinica (ICWEA).
1638	Outside of the MOO	Xilamulun	03JH059	Haitang Mountain	Granite	121.79	42.01	152	1.0	LA-ICP-MS,U-Pb	Zircon	Wu Fuyuan et al.	2006	Acta Petrologica Sinica (ICWEA).
1639	Outside of the MOO	Xilamulun	03JH054	Haitang Mountain	Granite	121.79	42.01	163	1.0	LA-ICP-MS,U-Pb	Zircon	Wu Fuyuan et al.	2006	Acta Petrologica Sinica (ICWEA).
1640	Outside of the MOO	Xilamulun	05FW095	Wanbao	Monzogranite	118.15	43.26	222	3.0	LA-ICP-MS,U-Pb	Zircon	Wu Fuyuan et al.	2011	Journal of Asian Earth Sciences
1641	Outside of the MOO	Xilamulun	05FW092	Wanbao	Mylonitize Granite	118.15	43.26	237	3.0	LA-ICP-MS,U-Pb	Zircon	Wu Fuyuan et al.	2011	Journal of Asian Earth Sciences
1642	Outside of the MOO	Xilamulun	05FW110	Shuangjing	Diorite	118.42	43.30	246	2.0	LA-ICP-MS,U-Pb	Zircon	Wu Fuyuan et al.	2011	Journal of Asian Earth Sciences
1643	Outside of the MOO	Xilamulun	05FW099	Yuanbaoshan	Monzogranite	118.46	43.34	273	3.0	LA-ICP-MS,U-Pb	Zircon	Wu Fuyuan et al.	2011	Journal of Asian Earth Sciences
1644	Outside of the MOO	Xilamulun	05FW121	Huanggangliang	Monzogranite	117.65	43.50	146	2.0	LA-ICP-MS,U-Pb	Zircon	Wu Fuyuan et al.	2011	Journal of Asian Earth Sciences
1645	Outside of the MOO	Xilamulun	05FW178	Telegute	Monzogranite	117.12	43.75	280	3.0	LA-ICP-MS,U-Pb	Zircon	Wu Fuyuan et al.	2011	Journal of Asian Earth Sciences
1646	Outside of the MOO	Xilamulun	05FW176	Dusheyetu	Monzogranite	117.15	43.75	321	1.0	LA-ICP-MS,U-Pb	Zircon	Wu Fuyuan et al.	2011	Journal of Asian Earth Sciences
1647	Outside of the MOO	Xilamulun	05FW133	Donghuang	Gabbro	117.91	43.76	252	5.0	LA-ICP-MS,U-Pb	Zircon	Wu Fuyuan et al.	2011	Journal of Asian Earth Sciences
1648	Outside of the MOO	Xilamulun	05FW137	Daqing Ranch	Syenogranite	117.84	43.77	283	2.0	LA-ICP-MS,U-Pb	Zircon	Wu Fuyuan et al.	2011	Journal of Asian Earth Sciences
1649	Outside of the MOO	Xilamulun	05FW167	Chaoyanggou	Monzogranite	117.57	43.87	246	2.0	LA-ICP-MS,U-Pb	Zircon	Wu Fuyuan et al.	2011	Journal of Asian Earth Sciences
1650	Outside of the MOO	Xilamulun	05FW140	Beidashan	Granodiorite	118.02	43.93	241	2.0	LA-ICP-MS,U-Pb	Zircon	Wu Fuyuan et al.	2011	Journal of Asian Earth Sciences
1651	Outside of the MOO	Xilamulun	05FW148	Chaoyanggou	Diorite enclave	118.17	44.13	150	4.0	LA-ICP-MS,U-Pb	Zircon	Wu Fuyuan et al.	2011	Journal of Asian Earth Sciences
1652	Outside of the MOO	Xilamulun	05FW161	Qianjinchang	Granidiorite	117.92	44.14	275	2.0	LA-ICP-MS,U-Pb	Zircon	Wu Fuyuan et al.	2011	Journal of Asian Earth Sciences
1653	Outside of the MOO	Xilamulun	05FW155	Qianjinchang	Granidiorite	117.86	44.16	274	1.0	LA-ICP-MS,U-Pb	Zircon	Wu Fuyuan et al.	2011	Journal of Asian Earth Sciences
1654	Outside of the MOO	Xilamulun	05FW162	Daqing Ranch	Monzogranite	117.67	44.19	274	4.0	LA-ICP-MS,U-Pb	Zircon	Wu Fuyuan et al.	2011	Journal of Asian Earth Sciences
1655	Outside of the MOO	Xilamulun	J3	Jiguanshan	granite porphyry(Granite porphyry）	119.12	42.41	156	1.3	SIMS,U-Pb	Zircon	Wu Huaying et al.	2014	Ore Geology Reviews
1656	Outside of the MOO	Xilamulun	0911J4	Jiguanshan	granite porphyry(Granite porphyry）	119.12	42.41	168	1.7	SIMS,U-Pb	Zircon	Wu Huaying et al.	2014	Ore Geology Reviews
1657	Outside of the MOO	Xilamulun	P4-GS24	Ulanba Mountain Rock Mass	Granite	112.75	41.58	225	1.6	U-Pb	Zircon	Wu Jiafu et al.	2012	Geological Survey and Research (ICWEA).
1658	Outside of the MOO	Xilamulun	CSHG-7	Changshanhao	Biotite granite	109.27	41.69	267	1.0	LA-ICP-MS,U-Pb	Zircon	Xiao Wei et al.	2012	Acta Petrologica Sinica (ICWEA).
1659	Outside of the MOO	Xilamulun	CSHG-4	Changshanhao	Biotite granite	109.27	41.69	274	2.0	LA-ICP-MS,U-Pb	Zircon	Xiao Wei et al.	2012	Acta Petrologica Sinica (ICWEA).
1660	Outside of the MOO	Xilamulun	wb08-n2	Daolang Huduge Rock	Hornblende biotite granodiorite	114.78	42.35	265	2.0	LA-ICP-MS,U-Pb	Zircon	Xing Jilin et al.	2010	Chang chun: Jilin University.
1661	Outside of the MOO	Xilamulun	wb08-n1	Daolang Huduge Rock	Hornblende biotite granodiorite	114.78	42.35	270	2.2	LA-ICP-MS,U-Pb	Zircon	Xing Jilin et al.	2010	Chang chun: Jilin University.
1662	Outside of the MOO	Xilamulun	801-4	Forward field	Porphyritic biotite granite	117.98	44.15	273	7.7	LA-ICP-MS,U-Pb	Zircon	Xu Jiajia et al.	2012	Acta Scientiarum Naturalium Universitatis Pekinensis (ICWEA).
1663	Outside of the MOO	Xilamulun	731-2	Forward field	Biotite granite	117.96	44.20	278	22.0	LA-ICP-MS,U-Pb	Zircon	Xu Jiajia et al.	2012	Acta Scientiarum Naturalium Universitatis Pekinensis (ICWEA).
1664	Outside of the MOO	Xilamulun	830-21	Forward field	Biotite granite	117.96	44.20	278	4.3	LA-ICP-MS,U-Pb	Zircon	Xu Jiajia et al.	2012	Acta Scientiarum Naturalium Universitatis Pekinensis (ICWEA).
1665	Outside of the MOO	Xilamulun	ZK0707-2	Villasto	Quartz-diorite	117.45	44.08	311	2.0	SHRIMP,U-Pb	Zircon	Xue Huaimin et al.	2010	Acta Petrologica et Mineralogica (ICWEA).
1666	Outside of the MOO	Xilamulun	ZK0302-1	Villasto	Diorite	117.47	44.06	310	2.0	SHRIMP,U-Pb	Zircon	Xue Huaimin et al.	2010	Acta Petrologica et Mineralogica (ICWEA).
1667	Outside of the MOO	Xilamulun	1.89162e+006	Bayern Daba silver-lead-zinc n	Granidiorite	117.55	44.20	319	3.0	SHRIMP,U-Pb	Zircon	Xue Huaimin et al.	2010	Acta Petrologica et Mineralogica (ICWEA).
1668	Outside of the MOO	Xilamulun	DB-4	Daolundaba mining area	Dactylic clastic tuff	117.96	44.21	300	5.0	SHRIMP,U-Pb	Zircon	Xue Huaimin et al.	2010	Acta Petrologica et Mineralogica (ICWEA).
1669	Outside of the MOO	Xilamulun	HB25	Dananfangzi Pluton	Coarse-grained granite	113.49	41.63	277	6.2	LA-ICP-MS,U-Pb	Zircon	Yan Huiying et al.	2014	Beijing: China University of Geosciences, Beijing.
1670	Outside of the MOO	Xilamulun	HB75	Erdaowa rock mass	Medium fine-grained granite	113.40	41.91	280	5.1	LA-ICP-MS,U-Pb	Zircon	Yan Huiying et al.	2014	Beijing: China University of Geosciences, Beijing.
1671	Outside of the MOO	Xilamulun	06JH126	Jianfang plutons	syenite	118.00	41.20	254	2.0	LA-ICP-MS,U-Pb	Zircon	Yang Jin Hui et al.	2012	2013 annual meeting of Institute of Geology and Geophysics, CAS(ICWEA)
1672	Outside of the MOO	Xilamulun	06JH62	Liangjia	quartz syenites(Quartz-syenite）	117.11	41.65	246	2.0	SIMS,U-Pb	Zircon	Yang Jin Hui et al.	2012	2012 annual meeting of Institute of Geology and Geophysics, CAS(ICWEA)
1673	Outside of the MOO	Xilamulun	E10101-1.1	Rock mass near Ulan Chongerg	Alkali granite	113.00	43.71	284	1.0	LA-ICP-MS,U-Pb	Zircon	Yang Qidi et al.	2014	Beijing: Chinese Academy of Geological Sciences.
1674	Outside of the MOO	Xilamulun	E10930-1	Rock mass near Saiyinwusu	Porphyritic syenogranite	112.32	43.65	279	1.0	LA-ICP-MS,U-Pb	Zircon	Yang Qidi et al.	2014	Beijing: Chinese Academy of Geological Sciences.
1675	Outside of the MOO	Xilamulun	GZ10-65	Tu Mu Fu Zhou rock mass	Bi-Syenogranite	119.30	44.31	154	1.0	LA-ICP-MS,U-Pb	Zircon	Yang Qidi et al.	2014	Acta Petrologica Sinica (ICWEA).
1676	Outside of the MOO	Xilamulun	GZ10-16	GanZhuer Temple	Bi-Monzogranite	119.66	44.84	228		LA-ICP-MS,U-Pb	Zircon	Yang Qidi et al.	2014	Beijing: Chinese Academy of Geological Sciences.
1677	Outside of the MOO	Xilamulun	007-163	Bilihe	quartz diorit porphyry	113.54	42.38	261	2.0	LA-ICP-MS,U-Pb	Zircon	Yang Zhiming et al.	2015	Gondwana Research
1678	Outside of the MOO	Xilamulun	B09-16	Bilihe	Andesitic tuff	113.54	42.38	270	4.0	LA-ICP-MS,U-Pb	Zircon	Yang Zhiming et al.	2015	Gondwana Research
1679	Outside of the MOO	Xilamulun	000-119.5	Bilihe	Rhyolite	113.54	42.38	274	3.0	LA-ICP-MS,U-Pb	Zircon	Yang Zhiming et al.	2015	Gondwana Research
1680	Outside of the MOO	Xilamulun	B1-2	Bilihe	quartz diorit porphyry	113.55	42.39	259	3.0	LA-ICP-MS,U-Pb	Zircon	Yang Zhiming et al.	2015	Gondwana Research
1681	Outside of the MOO	Xilamulun	BLH08-4	Bilihe	Syenogranite porphyry	113.55	42.41	253	3.0	LA-ICP-MS,U-Pb	Zircon	Yang Zhiming et al.	2015	Gondwana Research
1682	Outside of the MOO	Xilamulun	Cd-5	Linxi	Peraluminous granite	118.13	43.27	240		LA-ICP_MS	Zircon	Ye Xusong et al.	2011	Geological Science and Technology Information (ICWEA).
1683	Outside of the MOO	Xilamulun	2192—1	Xilinhot	Peraluminous granite	116.59	43.75	248	7.1	LA-ICP_MS	Zircon	Ye Xusong et al.	2011	Geological Science and Technology Information (ICWEA).
1684	Outside of the MOO	Xilamulun	0250—12	Xilinhot	Peraluminous granite	116.18	43.78	240		LA-ICP_MS	Zircon	Ye Xusong et al.	2011	Geological Science and Technology Information (ICWEA).
1685	Outside of the MOO	Xilamulun	2322—1	Linxi	Peraluminous granite	118.17	43.79	238	7.4	LA-ICP_MS	Zircon	Ye Xusong et al.	2011	Geological Science and Technology Information (ICWEA).
1686	Outside of the MOO	Xilamulun	G1-16	Baiyinnuoer	Diorite porphyrite	118.86	44.43	242	3.6	LA-ICP-MS,U-Pb	Zircon	Yi Jian et al.	2012	Geological Science and Technology Information (ICWEA).
1687	Outside of the MOO	Xilamulun	SK850-04	Baiyinnuoer	Quartz-Monzonite	118.86	44.43	243	1.4	LA-ICP-MS,U-Pb	Zircon	Yi Jian et al.	2012	Geological Science and Technology Information (ICWEA).
1688	Outside of the MOO	Xilamulun	1.21009e+007	Kangbaolan Chengzi granite bo	Medium grain porphyritic monzogra	114.83	41.60	150	2.2	SHRIMP,U-Pb	Zircon	Zeng Anbin et al.	2014	Chengdu: Chengdu University of Technology(ICWEA)
1689	Outside of the MOO	Xilamulun	C270	Chehugou	Syenogranite porphyry	118.50	42.42	266	4.0	SHRIMP,U-Pb	Zircon	Zeng Q D et al.	2012	Geological Magazine
1690	Outside of the MOO	Xilamulun	C134	Chehugou	Granite porphyry	118.51	42.44	246	3.0	SHRIMP,U-Pb	Zircon	Zeng Q D et al.	2012	Geological Magazine
1691	Outside of the MOO	Xilamulun	SB-D-1D	Dashigou biotite potassium feld	Bi-moyite	113.33	41.50	344	5.3	SHRIMP,U-Pb	Zircon	Zhang Chen et al.	2007	Acta Petrologica Sinica (ICWEA).
1692	Outside of the MOO	Xilamulun	FP2	Husihai	granodiorite	116.98	40.99	310	5.0	SHRIMP,U-Pb	Zircon	zhang et al.	2007	Journal of the Geological Society
1693	Outside of the MOO	Xilamulun	D018-1	Daguangding	quartz diorite	117.64	41.27	324	6.0	SHRIMP,U-Pb	Zircon	zhang et al.	2007	Journal of the Geological Society
1694	Outside of the MOO	Xilamulun	HFG-2	Guanglingshan	Monzogranite	117.27	41.17	254	4.0	SHRIMP,U-Pb	Zircon	Zhang et al.	2009	Earth and Planetary Science Letters
1695	Outside of the MOO	Xilamulun	D485	Hong Shi Li	Pyroxenite	117.27	41.17	349	4.0	LA-ICP-MS,U-Pb	Zircon	Zhang et al.	2009	Earth and Planetary Science Letters
1696	Outside of the MOO	Xilamulun	D169-2	Daguangding	Quartz diorite	117.45	41.24	314	6.0	SHRIMP,U-Pb	Zircon	Zhang et al.	2009	Earth and Planetary Science Letters
1697	Outside of the MOO	Xilamulun	D315-3	Jianping	Monzogranite	119.63	41.88	237	1.0	LA-ICP-MS,U-Pb	Zircon	Zhang et al.	2009	Earth and Planetary Science Letters

1698	Outside of the MOO	Xilamulun	D315-1	Jianping	Syenogranite dike	119.63	41.88	241	2.0	LA-ICP-MS,U-Pb	Zircon	Zhang et al.	2009	Earth and Planetary Science Letters
1699	Outside of the MOO	Xilamulun	D315	Jianping	Granodiorite	119.63	41.88	304	2.0	LA-ICP-MS,U-Pb	Zircon	Zhang et al.	2009	Earth and Planetary Science Letters
1700	Outside of the MOO	Xilamulun	FX09-2	Sijiazi	granodiorite	121.85	42.19	218	2.0	CASIMS,U-Pb	Zircon	Zhang et al.	2012	Lithos
1701	Outside of the MOO	Xilamulun	FX09-1	Sijiazi	monzodiorite	121.85	42.19	222	2.0	CASIMS,U-Pb	Zircon	Zhang et al.	2012	Lithos
1702	Outside of the MOO	Xilamulun	FX09-11	Sijiazi	granite	121.95	42.19	221	3.0	CASIMS,U-Pb	Zircon	Zhang et al.	2012	Lithos
1703	Outside of the MOO	Xilamulun	DSL09-4	Dashaoleng	granite	121.89	42.39	220	2.0	CASIMS,U-Pb	Zircon	Zhang et al.	2012	Lithos
1704	Outside of the MOO	Xilamulun	PA09-3	Ping'anzi	granite	121.88	42.51	238	2.0	CASIMS,U-Pb	Zircon	Zhang et al.	2012	Lithos
1705	Outside of the MOO	Xilamulun	07233-1	Shadegai, Baotou	Alkaline granite	109.62	40.81	235	3.0	LA-ICP-MS,U-Pb	Zircon	Zhang et al.	2014	Earth-Science Reviews
1706	Outside of the MOO	Xilamulun	D078-2	Lingying, Fengning	Dacite	117.08	41.03	156	4.0	LA-ICP-MS,U-Pb	Zircon	Zhang et al.	2014	Earth-Science Reviews
1707	Outside of the MOO	Xilamulun	09061-1	Chengjiawopu, Beipiao North Y	Quartz-Monzonite	120.48	42.02	164	1.0	LA-ICP-MS,U-Pb	Zircon	Zhang et al.	2014	Earth-Science Reviews
1708	Outside of the MOO	Xilamulun	07D006-1	Zhoujiawopu, SE Chifeng	Quartz trachyandesite	119.06	42.09	169	1.0	LA-ICP-MS,U-Pb	Zircon	Zhang et al.	2014	Earth-Science Reviews
1709	Outside of the MOO	Xilamulun	07D010-1	Zhoujiawopu, southeast of Chif	Quartz monzobiorite	119.13	42.10	159	2.0	LA-ICP-MS,U-Pb	Zircon	Zhang et al.	2014	Earth-Science Reviews
1710	Outside of the MOO	Xilamulun	08170-2	Peijiadian, East Chifeng	Rhyolitic tuff	120.27	42.30	162	2.0	LA-ICP-MS,U-Pb	Zircon	Zhang et al.	2014	Earth-Science Reviews
1711	Outside of the MOO	Xilamulun	09064-1	Nantaxiang, East Chifeng	Rhyolite	120.28	42.30	164	3.0	LA-ICP-MS,U-Pb	Zircon	Zhang et al.	2014	Earth-Science Reviews
1712	Outside of the MOO	Xilamulun	07D046-1	Jianchang, northwest of Chifen	Diorite porphyrite	118.79	42.50	171	3.0	LA-ICP-MS,U-Pb	Zircon	Zhang et al.	2014	Earth-Science Reviews
1713	Outside of the MOO	Xilamulun	09075-1	East Laohahe, East Chifeng	Rhyolitic tuff	119.65	42.56	163	2.0	LA-ICP-MS,U-Pb	Zircon	Zhang et al.	2014	Earth-Science Reviews
1714	Outside of the MOO	Xilamulun	HFH-1	Hushiha rock mass	Granite dike	117.00	40.98	301	8.0	SHRIMP,U-Pb	Zircon	Zhang Shuanhong et a	2004	Beijing: Chinese Academy of Geological Sciences.
1715	Outside of the MOO	Xilamulun	FP2030304	Hushiha rock mass	Porphyritic granite	116.98	40.98	304	3.0	SHRIMP,U-Pb	Zircon	Zhang Shuanhong et a	2004	Beijing: Chinese Academy of Geological Sciences.
1716	Outside of the MOO	Xilamulun	SD020-3	Longhua pluton	Genissic quartz-diorite	117.80	41.33	311	2.0	SHRIMP,U-Pb	Zircon	Zhang Shuanhong et a	2007	Journal of the Geological Society
1717	Outside of the MOO	Xilamulun	99YG25	Yangkeleng	Volcanic ash （volcanic ash）	110.37	40.63	290	6.0	SHRIMP,U-Pb	Zircon	Zhang Shuanhong et a	2009	International Journal of Earth sciences
1718	Outside of the MOO	Xilamulun	D464	Xianghuangqi–Wudaoyingzi pl	Diorite	116.75	40.96	283	2.0	LA-ICP-MS,U-Pb	Zircon	Zhang Shuanhong et a	2009	International Journal of Earth sciences
1719	Outside of the MOO	Xilamulun	Ch9808146	Zhengningbu	Deformed granite （Metamorphic g	115.64	40.98	273	1.0	Pb-Pb Evaporation	Zircon	Zhang Shuanhong et a	2009	International Journal of Earth sciences
1720	Outside of the MOO	Xilamulun	D215-1	Zhoutaizi pluton	Quartz-diorite	117.56	40.98	291	3.0	LA-ICP-MS,U-Pb	Zircon	Zhang Shuanhong et a	2009	International Journal of Earth sciences
1721	Outside of the MOO	Xilamulun	D077-1	Tianqiao pluton	Quartz-diorite	116.97	40.99	288	2.0	LA-ICP-MS,U-Pb	Zircon	Zhang Shuanhong et a	2009	International Journal of Earth sciences
1722	Outside of the MOO	Xilamulun	RP-16	Luanping	Orbicular diorite （Spherical diorit	117.27	41.00	284	8.0	SHRIMP,U-Pb	Zircon	Zhang Shuanhong et a	2009	International Journal of Earth sciences
1723	Outside of the MOO	Xilamulun	Ch9808160	Xianghuangqi–Wudaoyingzi pl	Diorite	116.97	41.03	274	6.0	SHRIMP,U-Pb	Zircon	Zhang Shuanhong et a	2009	International Journal of Earth sciences
1724	Outside of the MOO	Xilamulun	D079-1	Lingying pluton	Granidiorite	117.10	41.03	288	4.0	LA-ICP-MS,U-Pb	Zircon	Zhang Shuanhong et a	2009	International Journal of Earth sciences
1725	Outside of the MOO	Xilamulun	D252	Xianghuangqi–Wudaoyingzi pl	Evjite	116.95	41.05	276	2.0	LA-ICP-MS,U-Pb	Zircon	Zhang Shuanhong et a	2009	International Journal of Earth sciences
1726	Outside of the MOO	Xilamulun	X3-3	Yuanhengyong	Quartz diorite （Quartz-diorite）	110.63	41.08	282	5.0	TIMS,U-Pb	Zircon	Zhang Shuanhong et a	2009	International Journal of Earth sciences
1727	Outside of the MOO	Xilamulun		Qinghechang	Quartz diorite （Quartz-diorite）	110.80	41.18	299	4.0	TIMS,U-Pb	Zircon	Zhang Shuanhong et a	2009	International Journal of Earth sciences
1728	Outside of the MOO	Xilamulun	D404-1	Ningbao East, Chicheng Town	Gneissose granite	115.67	40.98	292	3.0	LA-ICP-MS,U-Pb	Zircon	Zhang Shuanhong et a	2010	Acta Petrologica et Mineralogica (ICWEA).
1729	Outside of the MOO	Xilamulun	07251-1	Alkali room	Monzogranite	117.20	41.63	253	1.0	LA-ICP-MS,U-Pb	Zircon	Zhang Shuanhong et a	2010	Acta Petrologica et Mineralogica (ICWEA).
1730	Outside of the MOO	Xilamulun	07027-2	Grand Culence	Bi-granodiorite	113.44	41.89	268	2.0	LA-ICP-MS,U-Pb	Zircon	Zhang Shuanhong et a	2010	Acta Petrologica et Mineralogica (ICWEA).
1731	Outside of the MOO	Xilamulun	NM010-1	Bupleurum	Gabbro diorite	118.49	42.33	271	2.0	LA-ICP-MS,U-Pb	Zircon	Zhang Shuanhong et a	2010	Acta Petrologica et Mineralogica (ICWEA).
1732	Outside of the MOO	Xilamulun	07236-1	Yongfucun	syenite	110.22	40.57	224	2.0	LA-ICP-MS,U-Pb	Zircon	Zhang Shuanhong et a	2012	lithos
1733	Outside of the MOO	Xilamulun	10097-1	Yunwushan	Monzogranite	116.62	40.95	244	2.0	LA-ICP-MS,U-Pb	Zircon	Zhang Shuanhong et a	2014	Earth-Science Reviews
1734	Outside of the MOO	Xilamulun	10095-1	Yunwushan	Monzogranite	116.63	41.04	247	2.0	LA-ICP-MS,U-Pb	Zircon	Zhang Shuanhong et a	2014	Earth-Science Reviews
1735	Outside of the MOO	Xilamulun	D478	Jizhazi	Monzogranite	116.77	41.07	229	5.0	LA-ICP-MS,U-Pb	Zircon	Zhang Shuanhong et a	2014	Earth-Science Reviews
1736	Outside of the MOO	Xilamulun	CGDLH04	Damaoqi Chegendalai	enclave（Diorite）	110.83	41.89	239	2.0	SHRIMP,U-Pb	Zircon	Zhang wei et al.	2010	Geological Bulletin of China(ICWEA)
1737	Outside of the MOO	Xilamulun	CGDLH05	Damaoqi Chegendalai	Granidiorite	110.83	41.89	245	1.0	SHRIMP,U-Pb	Zircon	Zhang wei et al.	2010	Geological Bulletin of China(ICWEA)
1738	Outside of the MOO	Xilamulun	GY15	Guyang area	Quartz-diorite	110.36	41.01	272	7.0	SHRIMP,U-Pb	Zircon	Zhang Wei et al.	2012	Geology in China (ICWEA).
1739	Outside of the MOO	Xilamulun	GY-41	Gu Yang	Diorite	110.36	41.01	266	4.0	SHRIMP,U-Pb	Zircon	Zhang wei et al.	2012	Geology in China (ICWEA)
1740	Outside of the MOO	Xilamulun	GY16	Guyang area	Diorite	110.39	41.11	293	4.0	SHRIMP,U-Pb	Zircon	Zhang Wei et al.	2012	Geology in China (ICWEA).
1741	Outside of the MOO	Xilamulun	GY16-3	Gu Yang	Diorite	110.39	41.11	296	4.0	SHRIMP,U-Pb	Zircon	Zhang wei et al.	2012	Geology in China (ICWEA)
1742	Outside of the MOO	Xilamulun	GY36	Guyang area	Tonalite	110.05	41.17	277	3.0	SHRIMP,U-Pb	Zircon	Zhang Wei et al.	2012	Geology in China (ICWEA).
1743	Outside of the MOO	Xilamulun	GY41	Guyang area	Diorite	110.45	41.68	266	4.0	SHRIMP,U-Pb	Zircon	Zhang Wei et al.	2012	Geology in China (ICWEA).
1744	Outside of the MOO	Xilamulun	HP9	Hengshan	gabbo	111.95	42.97	167	6.0	LA-ICP-MS,U-Pb	Zircon	Zhang X H et al.	2010	Geological Magazine
1745	Outside of the MOO	Xilamulun	NM07-138	Guyang batholith	dyke	110.54	41.07	276	2.1	LA-ICP-MS,U-Pb	Zircon	Zhang Xiaohui et al.	2011	Lithos
1746	Outside of the MOO	Xilamulun	NM07-106	Guyang batholith	diorite	110.47	41.08	279	1.4	LA-ICP-MS,U-Pb	Zircon	Zhang Xiaohui et al.	2011	Lithos
1747	Outside of the MOO	Xilamulun	NM07-108	Guyang batholith	granodiorite	110.47	41.08	280	1.9	LA-ICP-MS,U-Pb	Zircon	Zhang Xiaohui et al.	2011	Lithos
1748	Outside of the MOO	Xilamulun	NM07-73	Guyang batholith	gabbro	110.62	41.09	280	1.9	LA-ICP-MS,U-Pb	Zircon	Zhang Xiaohui et al.	2011	Lithos
1749	Outside of the MOO	Xilamulun	NM07-77	Guyang batholith	gabbro	110.62	41.08	280	1.9	LA-ICP-MS,U-Pb	Zircon	Zhang Xiaohui et al.	2011	Lithos
1750	Outside of the MOO	Xilamulun	NM07-76	Guyang batholith	gabbroic diorite	110.62	41.09	280	1.9	LA-ICP-MS,U-Pb	Zircon	Zhang Xiaohui et al.	2011	Lithos
1751	Outside of the MOO	Xilamulun	NM07-56	Guyang batholith	gabbroic diorite	110.62	41.07	286	2.8	LA-ICP-MS,U-Pb	Zircon	Zhang Xiaohui et al.	2011	Lithos
1752	Outside of the MOO	Xilamulun	NM07-94	Guyang batholith	dyke	110.33	41.12	276	2.1	LA-ICP-MS,U-Pb	Zircon	Zhang Xiaohui et al.	2011	Lithos
1753	Outside of the MOO	Xilamulun	NM07-97	Guyang batholith	diorite	110.34	41.12	279	1.4	LA-ICP-MS,U-Pb	Zircon	Zhang Xiaohui et al.	2011	Lithos
1754	Outside of the MOO	Xilamulun	NM07-156	Guyang batholith	granite	110.88	41.13	282	2.1	LA-ICP-MS,U-Pb	Zircon	Zhang Xiaohui et al.	2011	Lithos
1755	Outside of the MOO	Xilamulun	NM07-144	Guyang batholith	granodiorite	110.38	41.17	280	1.9	LA-ICP-MS,U-Pb	Zircon	Zhang Xiaohui et al.	2011	Lithos
1756	Outside of the MOO	Xilamulun	NM07-145/1	Guyang batholith	MME	110.40	41.17	281	1.6	LA-ICP-MS,U-Pb	Zircon	Zhang Xiaohui et al.	2011	Lithos
1757	Outside of the MOO	Xilamulun	NM07-181	Guyang batholith	diorite	110.83	41.18	279	1.4	LA-ICP-MS,U-Pb	Zircon	Zhang Xiaohui et al.	2011	Lithos
1758	Outside of the MOO	Xilamulun	BLS-4	Xinmin Township, Arhorqin ba	Rhyolite porphyry	120.11	44.06	160	2.0	LA-ICP-MS,U-Pb	Zircon	Zhang Xiaojing et al.	2010	Acta Petrologica Sinica (ICWEA).
1759	Outside of the MOO	Xilamulun		Damiao Rock	Granidiorite	111.58	41.92	265	7.0	LA-ICP-MS,U-Pb	Zircon	Zhang Xuebin et al.	2009	Acta Petrologica Sinica (ICWEA).
1760	Outside of the MOO	Xilamulun	21-46	Tongdingshan Pluton	Monzogranite	113.52	42.04	237	4.2	TIMS,U-Pb	Zircon	Zhang Xuebin et al.	2011	Bulletin of Geological Science and Technology (ICWEA).
1761	Outside of the MOO	Xilamulun	21-22	Hexiu Village-Minzhu Village	Syenogranite	113.68	42.08	232	1.1	TIMS,U-Pb	Zircon	Zhang Xuebin et al.	2011	Bulletin of Geological Science and Technology (ICWEA).
1762	Outside of the MOO	Xilamulun		Nianzigou Molybdenum Mine	Medium coarse Bi-Monzogranite	118.63	42.42	152	1.6	SHRIMP,U-Pb	Zircon	Zhang Xuebin et al.	2011	Mineral Deposits (ICWEA).
1763	Outside of the MOO	Xilamulun		Erlianhot	Granite	112.08	43.51	277	5.1	LA-ICP-MS,U-Pb	Zircon	Zhang Xuebin et al.	2014	China Earth Science Joint Academic Conference.
1764	Outside of the MOO	Xilamulun	NM111	Hemu Rock	Moyite	113.62	42.08	229	3.4	LA-ICP-MS,U-Pb	Zircon	Zhang Xuebin et al.	2014	Beijing: China University of Geosciences, Beijing.
1765	Outside of the MOO	Xilamulun	DS-147-N	Mandulatu Granite Porphyry	Granite porphyry	113.65	43.87	151	5.0	LA-ICP-MS,U-Pb	Zircon	Zhang Xuebin et al.	2014	Beijing: China University of Geosciences, Beijing.
1766	Outside of the MOO	Xilamulun	P01-b6	Daqing Ranch	Quartz-diorite	117.55	44.13	330	2.0	LA-ICP-MS,U-Pb	Zircon	Zhang Xuebin et al.	2014	Beijing: China University of Geosciences, Beijing.
1767	Outside of the MOO	Xilamulun	XW09-b6	Forward field	Biotite granite	117.89	44.13	271	1.3	LA-ICP-MS,U-Pb	Zircon	Zhang Xuebin et al.	2014	Beijing: China University of Geosciences, Beijing.
1768	Outside of the MOO	Xilamulun	XW01-b7	Forward field	Granidiorite	117.90	44.22	274	1.1	LA-ICP-MS,U-Pb	Zircon	Zhang Xuebin et al.	2014	Beijing: China University of Geosciences, Beijing.
1769	Outside of the MOO	Xilamulun	DL-01	Dalunda dam	Medium coarse Biotite granite	117.96	44.26	292	0.8	LA-ICP-MS,U-Pb	Zircon	Zhang Xuebin et al.	2014	Acta Petrologica Sinica (ICWEA).
1770	Outside of the MOO	Xilamulun	DL-14	Dalunda dam	Medium coarse Biotite granite	117.96	44.26	293	0.9	LA-ICP-MS,U-Pb	Zircon	Zhang Xuebin et al.	2014	Acta Petrologica Sinica (ICWEA).
1771	Outside of the MOO	Xilamulun	XW03-b7	Forward field	Biotite granite	117.97	44.27	282	1.2	LA-ICP-MS,U-Pb	Zircon	Zhang Xuebin et al.	2014	Beijing: China University of Geosciences, Beijing.
1772	Outside of the MOO	Xilamulun	ZJEH-28-U.Pb01	Ula-Usu Tuhundi	Monzogranite	116.55	44.42	302	3.2	LA-ICP-MS,U-Pb	Zircon	Zhang Xuebin et al.	2014	Beijing: China University of Geosciences, Beijing.
1773	Outside of the MOO	Xilamulun	U-P-16	Ula	Medium coarse Gabbro-diorite	116.55	44.42	310	1.0	LA-ICP-MS,U-Pb	Zircon	Zhang Xuebin et al.	2014	Beijing: China University of Geosciences, Beijing.
1774	Outside of the MOO	Xilamulun	U-P-22	Uheer Chulu	Medium fine-grained Gabbro	116.55	44.42	317	1.2	LA-ICP-MS,U-Pb	Zircon	Zhang Xuebin et al.	2014	Beijing: China University of Geosciences, Beijing.
1775	Outside of the MOO	Xilamulun	U-P-19	Uher Chulu	Monzogranite	116.55	44.42	326	1.4	LA-ICP-MS,U-Pb	Zircon	Zhang Xuebin et al.	2014	Beijing: China University of Geosciences, Beijing.

1776	Outside of the MOO	Xilamulun	WHHS-46-U.Pb02	Uheer Chulu	Granidiorite	116.55	44.42	331	1.8	LA-ICP-MS,U-Pb	Zircon	Zhang Xuebin et al.	2014	Beijing: China University of Geosciences, Beijing.
1777	Outside of the MOO	Xilamulun	WHHS-48-U.P01	Uher Chulu	Granidiorite	116.55	44.42	332	2.6	LA-ICP-MS,U-Pb	Zircon	Zhang Xuebin et al.	2014	Beijing: China University of Geosciences, Beijing.
1778	Outside of the MOO	Xilamulun	DY09-1	Ningcheng	gabbro	118.20	41.33	219	1.0	LA-ICP-MS,U-Pb	Zircon	Zhang Z et al.	2014	Journal of Asian Earth Sciences
1779	Outside of the MOO	Xilamulun	DY09-8，DY09-10	Ningcheng	pyroxenite	118.20	41.38	225	2.0	LA-ICP-MS,U-Pb	Zircon	Zhang Z et al.	2014	Journal of Asian Earth Sciences
1780	Outside of the MOO	Xilamulun	DY09-14	Ningcheng	pyroxenite	118.23	41.40	226	2.0	LA-ICP-MS,U-Pb	Zircon	Zhang Z et al.	2014	Journal of Asian Earth Sciences
1781	Outside of the MOO	Xilamulun	DY09-12	Jiwangyingzi	Jiwangyingzi Formation diorite	118.50	41.42	253	4.0	LA-ICP-MS,U-Pb	Zircon	Zhang Z et al.	2014	Journal of Asian Earth Sciences
1782	Outside of the MOO	Xilamulun	DY09-11	Jiwangyingzi	Jiwangyingzi Formation granitic gn	118.50	41.42	298	4.0	LA-ICP-MS,U-Pb	Zircon	Zhang Z et al.	2014	Journal of Asian Earth Sciences
1783	Outside of the MOO	Xilamulun	DY09-3	Ningcheng	gabbro diorite	118.45	41.45	220	7.0	LA-ICP-MS,U-Pb	Zircon	Zhang Z et al.	2014	Journal of Asian Earth Sciences
1784	Outside of the MOO	Xilamulun	DY09-5	Ningcheng	gabbro diorite	118.46	41.43	228	2.0	LA-ICP-MS,U-Pb	Zircon	Zhang Z et al.	2014	Journal of Asian Earth Sciences
1785	Outside of the MOO	Xilamulun	DY09-9，DY09-10-2	Ningcheng	quartz diorite	118.47	41.47	226	2.0	LA-ICP-MS,U-Pb	Zircon	Zhang Z et al.	2014	Journal of Asian Earth Sciences
1786	Outside of the MOO	Xilamulun	NM08-113	ophiolite complex	Diorite porphyry	112.63	43.83	314	2.9	SHRIMP,U-Pb	Zircon	Zhang Zhicheng et al.	2015	Journal of Asian Earth Sciences
1787	Outside of the MOO	Xilamulun	NM10-72	ophiolite complex	Plagioclase granite	112.73	43.83	345	5.5	SHRIMP,U-Pb	Zircon	Zhang Zhicheng et al.	2015	Journal of Asian Earth Sciences
1788	Outside of the MOO	Xilamulun	NM08-137	ophiolite complex	Gabbro	112.73	43.83	353	2.7	SHRIMP,U-Pb	Zircon	Zhang Zhicheng et al.	2015	Journal of Asian Earth Sciences
1789	Outside of the MOO	Xilamulun	NM10-73	ophiolite complex	Gabbro	112.73	43.83	353	3.7	SHRIMP,U-Pb	Zircon	Zhang Zhicheng et al.	2015	Journal of Asian Earth Sciences
1790	Outside of the MOO	Xilamulun	U0013-102	Tongxunlian	granodiorite	116.23	43.85	318	1.0	LA-ICP-MS,U-Pb	Zircon	Zhou W X et al.	2014	Geological Journal
1791	Outside of the MOO	Xilamulun	U35-48-1	Tongxunlian	gabbro	116.23	43.86	319	1.4	LA-ICP-MS,U-Pb	Zircon	Zhou W X et al.	2014	Geological Journal
1792	Outside of the MOO	E. Xilamulun		Jianpingzhen	Monzogranite	124.42	42.82	249	1.0	LA-ICP-MS,U-Pb	Zircon	Cao H.H, et al.	2013	Lithos
1793	Outside of the MOO	E. Xilamulun		Yishan	Monzogranite	124.40	43.05	247	1.0	LA-ICP-MS,U-Pb	Zircon	Cao H.H, et al.	2013	Lithos
1794	Outside of the MOO	E. Xilamulun	YJG8		Biotite granodiorite	130.90	43.12	256		LA-ICP-MS,U-Pb	Zircon	Chen Cong .	2017	Doctoral thesis: Jilin University(ICWEA)
1795	Outside of the MOO	E. Xilamulun	SDG3		Biotite tonalite	130.92	43.00	265		LA-ICP-MS,U-Pb	Zircon	Chen Cong, et al.	2017	Journal of Asian Earth Sciences
1796	Outside of the MOO	E. Xilamulun	13GW010		Quartz diorite	131.24	44.99	204		LA-ICP-MS,U-Pb	Zircon	Chen Yanhong et al.	2015	Acta Geologica Sinica(ICWEA)
1797	Outside of the MOO	E. Xilamulun	13GW012		Syenogranite	131.11	45.01	210		LA-ICP-MS,U-Pb	Zircon	Chen Yanhong et al.	2015	Acta Geologica Sinica(ICWEA)
1798	Outside of the MOO	E. Xilamulun	12GW015		Diorite	131.53	45.08	208		LA-ICP-MS,U-Pb	Zircon	Chen Yanhong et al.	2015	Acta Geologica Sinica(ICWEA)
1799	Outside of the MOO	E. Xilamulun	12GW016		Syenogranite	131.53	45.08	209		LA-ICP-MS,U-Pb	Zircon	Chen Yanhong et al.	2015	Acta Geologica Sinica(ICWEA)
1800	Outside of the MOO	E. Xilamulun	13GW017		Monzogranite	131.53	45.08	211		LA-ICP-MS,U-Pb	Zircon	Chen Yanhong et al.	2015	Acta Geologica Sinica(ICWEA)
1801	Outside of the MOO	E. Xilamulun	13GW018		Quartz diorite	131.53	45.08	211		LA-ICP-MS,U-Pb	Zircon	Chen Yanhong et al.	2015	Acta Geologica Sinica(ICWEA)
1802	Outside of the MOO	E. Xilamulun	12GW019		Gabbroic diorite	131.52	45.14	213		LA-ICP-MS,U-Pb	Zircon	Chen Yanhong et al.	2015	Acta Geologica Sinica(ICWEA)
1803	Outside of the MOO	E. Xilamulun	BJZ-3	Bajiaziyanti	birkremite	127.52	42.42	218	6.0	SHRIMP,U-Pb	Zircon	Cheng Yuruo, et al.	2005	Acta Petrologica Sinica(ICWEA)
1804	Outside of the MOO	E. Xilamulun	H H-1		Monzogranite	127.51	42.98	166		SHRIMP,U-Pb	Zircon	Cheng Yuruo, et al.	2005	Acta Petrologica Sinica(ICWEA)
1805	Outside of the MOO	E. Xilamulun		Mayihe	Monzogranite	126.97	41.82	155		TIMS,U-Pb	Zircon	Fang Wenchang.	1993	Changchun: Jilin Science and Technology Press
1806	Outside of the MOO	E. Xilamulun		Xiaolihe	granite	126.00	42.38	338		TIMS,U-Pb	Zircon	Fang Wenchang.	1993	Changchun: Jilin Science and Technology Press
1807	Outside of the MOO	E. Xilamulun		Dongqing	granite	128.08	42.83	208		TIMS,U-Pb	Zircon	Fang Wenchang.	1993	Changchun: Jilin Science and Technology Press
1808	Outside of the MOO	E. Xilamulun		Wolong	quartz diorite	124.37	42.93	193		TIMS,U-Pb	Zircon	Fang Wenchang.	1993	Changchun: Jilin Science and Technology Press
1809	Outside of the MOO	E. Xilamulun		Shimen	Granodiorite	128.75	42.97	245		TIMS,U-Pb	Zircon	Fang Wenchang.	1993	Changchun: Jilin Science and Technology Press
1810	Outside of the MOO	E. Xilamulun		Yangmulin	Granodiorite	126.18	43.67	231		TIMS,U-Pb	Zircon	Fang Wenchang.	1993	Changchun: Jilin Science and Technology Press
1811	Outside of the MOO	E. Xilamulun		Dongtazi	plagiogranite	126.92	43.77	181		TIMS,U-Pb	Zircon	Fang Wenchang.	1993	Changchun: Jilin Science and Technology Press
1812	Outside of the MOO	E. Xilamulun		Laoyaogou	Granodiorite	126.50	43.95	173		TIMS,U-Pb	Zircon	Fang Wenchang.	1993	Changchun: Jilin Science and Technology Press
1813	Outside of the MOO	E. Xilamulun		Shuangyazi	Monzogranite	126.78	43.95	148		TIMS,U-Pb	Zircon	Fang Wenchang.	1993	Changchun: Jilin Science and Technology Press
1814	Outside of the MOO	E. Xilamulun		Zhujia	plagiogranite	126.90	44.17	185		TIMS,U-Pb	Zircon	Fang Wenchang.	1993	Changchun: Jilin Science and Technology Press
1815	Outside of the MOO	E. Xilamulun		Qijia	Granodiorite	126.87	44.38	177		TIMS,U-Pb	Zircon	Fang Wenchang.	1993	Changchun: Jilin Science and Technology Press
1816	Outside of the MOO	E. Xilamulun	1709A	Dongqing	Gt Monzogranite	128.23	42.81	159	5.0	TIMS,U-Pb	Zircon	Fu Changliang .	2009	Master thesis: Jilin University(ICWEA)
1817	Outside of the MOO	E. Xilamulun	D0849-1	Xiaoxinancha	High-Mg diorite	130.89	43.21	240	1.0	LA-ICP-MS,U-Pb	Zircon	Fu Changliang, et al.	2009	Master thesis: Jilin University(ICWEA)
1818	Outside of the MOO	E. Xilamulun	D0832-6	Taipingling	High-Mg diorite	131.00	43.26	241	1.0	LA-ICP-MS,U-Pb	Zircon	Fu Changliang, et al.	2009	Master thesis: Jilin University(ICWEA)
1819	Outside of the MOO	E. Xilamulun		Xiaoxinancha	High-Mg diorite	130.89	43.21	240	1.0	LA-ICP-MS,U-Pb	Zircon	Fu Changliang, et al.	2010	Acta Geologica Sinica(ICWEA)
1820	Outside of the MOO	E. Xilamulun	409D0849-1	Xiaoxinancha	High-Mg diorite	130.89	43.21	240	1.0	LA-ICP-MS,U-Pb	Zircon	Fu Changliang, et al.	2010	Acta Petrologica et Mineralogica(ICWEA)
1821	Outside of the MOO	E. Xilamulun		Jilinhuichuntaipinggoucunyanti		131.00	43.26	241	1.0	LA-ICP-MS,U-Pb	Zircon	Fu changliang, et al.	2010	Acta Petrologica Sinica(ICWEA)
1822	Outside of the MOO	E. Xilamulun	H-56		Granodiorite	129.77	42.10	246	1.0	LA-ICP-MS,U-Pb	Zircon	Fu-Yuan Wu.	2007	Chemical Geology
1823	Outside of the MOO	E. Xilamulun	H-31		Granodiorite	129.89	42.39	182	2.0	LA-ICP-MS,U-Pb	Zircon	Fu-Yuan Wu.	2007	Chemical Geology
1824	Outside of the MOO	E. Xilamulun	H-5		Granodiorite	130.46	42.43	193	1.0	LA-ICP-MS,U-Pb	Zircon	Fu-Yuan Wu.	2007	Chemical Geology
1825	Outside of the MOO	E. Xilamulun	MZ4	Baoshan	alkali-feldspar granite	128.86	43.09	253	3.0	LA-ICP-MS,U-Pb	Zircon	Gao Yang, et al.	2009	Acta Petrologica et Mineralogica(ICWEA)
1826	Outside of the MOO	E. Xilamulun	YC1-1	Drill hole	diorite	126.38	45.07	319	1.0	LA-ICP-MS,U-Pb	Zircon	Gao Zhenquan, et al.	2006	Acta Geologica Sinica(ICWEA)
1827	Outside of the MOO	E. Xilamulun	H15-72-1	Liangziling	Monzogranite	128.69	44.86	192	1.0	LA-ICP-MS,U-Pb	Zircon	Ge Maohui, et al.	2020	Acta Petrologica et Mineralogica(ICWEA)
1828	Outside of the MOO	E. Xilamulun	H15-70-1	Qingyunshan	Syenogranite	128.42	45.13	200	1.0	LA-ICP-MS,U-Pb	Zircon	Ge Maohui, et al.	2020	Acta Petrologica et Mineralogica(ICWEA)
1829	Outside of the MOO	E. Xilamulun	D13NA02	Ninganhongchengcun	granite	129.42	44.35	261	2.0	LA-ICP-MS,U-Pb	Zircon	Ge Wenchun , et al.	2005	Acta Petrologica Sinica(ICWEA)
1830	Outside of the MOO	E. Xilamulun	D13CSh02	Linkouchushandong	Granodiorite	130.00	45.12	261	2.0	LA-ICP-MS,U-Pb	Zircon	Ge Wenchun , et al.	2005	Acta Petrologica Sinica(ICWEA)
1831	Outside of the MOO	E. Xilamulun	D13DM02	Linkoudamadanggou	混合Granodiorite	130.08	45.41	258	2.0	LA-ICP-MS,U-Pb	Zircon	Ge Wenchun , et al.	2005	Acta Petrologica Sinica(ICWEA)
1832	Outside of the MOO	E. Xilamulun	GW04530	Daheishan	Granodiorite	126.33	43.49	170	3.0	LA-ICP-MS,U-Pb	Zircon	GE WenChun, et al.	2007	Chinese Science Bulletin(ICWEA)
1833	Outside of the MOO	E. Xilamulun		Daheishan	Monzogranite	126.31	43.50	178	3.0	LA-ICP-MS,U-Pb	Zircon	GE WenChun, et al.	2007	Chinese Science Bulletin(ICWEA)
1834	Outside of the MOO	E. Xilamulun	GW04542	Daheishan	Monzogranite	126.31	43.50	178	3.0	LA-ICP-MS,U-Pb	Zircon	GE WenChun, et al.	2007	Chinese Science Bulletin(ICWEA)
1835	Outside of the MOO	E. Xilamulun		Yongxin	Monzogranite	129.33	42.62	243	1.0	LA-ICP-MS,U-Pb	Zircon	Guan Qingbin, et al.	2016	Acta Petrologica Sinica(ICWEA)
1836	Outside of the MOO	E. Xilamulun	2.88505e+006	Chaihe	Monzogranite	129.69	44.69	256	5.0	SHRIMP,U-Pb	Zircon	Guan Qingbin, et al.	2016	Acta Petrologica Sinica(ICWEA)
1837	Outside of the MOO	E. Xilamulun		Nantian	Granodiorite	129.22	42.34	235	1.0	LA-ICP-MS,U-Pb	Zircon	Guan Qingbin, et al.	2020	GR(ICWEA).
1838	Outside of the MOO	E. Xilamulun		Dalingou	Granodiorite	129.45	42.55	176	1.0	LA-ICP-MS,U-Pb	Zircon	Guan Qingbin, et al.	2020	GR(ICWEA).
1839	Outside of the MOO	E. Xilamulun		Fudongzhen	Biotite granite	129.17	42.59	249	1.0	LA-ICP-MS,U-Pb	Zircon	Guan Qingbin, et al.	2020	GR(ICWEA).
1840	Outside of the MOO	E. Xilamulun		Daxin	Monzogranite	129.39	42.62	196	1.0	LA-ICP-MS,U-Pb	Zircon	Guan Qingbin, et al.	2020	GR(ICWEA).
1841	Outside of the MOO	E. Xilamulun	2.92529e+006	Xiangshuiyuanzi	moyite	125.36	42.95	184	3.0	TIMS,U-Pb	Zircon	Li Jingyan, et al.	2014	Acta Petrologica Sinica(ICWEA)
1842	Outside of the MOO	E. Xilamulun	SCS024	Shancheshan	quartz diorite	126.14	42.99	164		LA-ICP-MS,U-Pb	Zircon	Li Jingyan, et al.	2014	Acta Petrologica Sinica(ICWEA)
1843	Outside of the MOO	E. Xilamulun	2.85039e+006	Jiangmifeng	diorite	126.73	43.97	173	4.0	SHRIMP,U-Pb	Zircon	Li Jingyan, et al.	2014	Acta Petrologica Sinica(ICWEA)
1844	Outside of the MOO	E. Xilamulun	DY1044	Shulan	Granodiorite	126.89	44.34	190	2.0	LA-ICP-MS,U-Pb	Zircon	Li Jingyan, et al.	2014	Acta Petrologica Sinica(ICWEA)
1845	Outside of the MOO	E. Xilamulun		Daganhe	Monzogranite	124.81	42.34	242	1.0	LA-ICP-MS,U-Pb	Zircon	Liu Jin, et al.	2016	Acta Petrologica Sinica(ICWEA)
1846	Outside of the MOO	E. Xilamulun		Jianshanzi	Monzogranite	124.68	42.42	251	2.0	LA-ICP-MS,U-Pb	Zircon	Liu Jin, et al.	2016	Acta Petrologica Sinica(ICWEA)
1847	Outside of the MOO	E. Xilamulun		Baoxing	Granodiorite	124.81	42.42	235	1.0	LA-ICP-MS,U-Pb	Zircon	Liu Jin, et al.	2016	Acta Petrologica Sinica(ICWEA)
1848	Outside of the MOO	E. Xilamulun		Shudetun	Diorite	124.86	42.43	224	2.0	LA-ICP-MS,U-Pb	Zircon	Liu Jin.	2017	Doctoral thesis: Jilin University(ICWEA)
1849	Outside of the MOO	E. Xilamulun		Shuangling	Monzogranite	124.99	42.54	176	2.0	LA-ICP-MS,U-Pb	Zircon	Liu Jin.	2017	Doctoral thesis: Jilin University(ICWEA)
1850	Outside of the MOO	E. Xilamulun		Zhenxing	Biotite granite	124.96	42.62	180	1.0	LA-ICP-MS,U-Pb	Zircon	Liu Jin.	2017	Doctoral thesis: Jilin University(ICWEA)
1851	Outside of the MOO	E. Xilamulun	SMZM-01	Liukesongyanti	gabbro	127.88	43.09	262	0.7	LA-ICP-MS,U-Pb	Zircon	Liu Xianfan, et al.	2010	Earth Science Frontiers(ICWEA)
1852	Outside of the MOO	E. Xilamulun	YJ2-100		Monzogranite	129.01	42.10	246		LA-ICP-MS,U-Pb	Zircon	Ma XingHua, et al.	2015	Journal of Asian Earth Sciences
1853	Outside of the MOO	E. Xilamulun	YJ-30		Granodiorite	129.20	42.32	201		LA-ICP-MS,U-Pb	Zircon	Ma XingHua, et al.	2015	Journal of Asian Earth Sciences

1854	Outside of the MOO	E. Xilamulun	YJ2-73		Monzogranite	128.95	42.33	243		LA-ICP-MS,U-Pb	Zircon	Ma XingHua, et al.	2015	Journal of Asian Earth Sciences
1855	Outside of the MOO	E. Xilamulun	YJ-36		Monzogranite	129.02	42.37	251		LA-ICP-MS,U-Pb	Zircon	Ma XingHua, et al.	2015	Journal of Asian Earth Sciences
1856	Outside of the MOO	E. Xilamulun	YJ2-32		Diorite	129.21	42.46	172		LA-ICP-MS,U-Pb	Zircon	Ma XingHua, et al.	2015	Journal of Asian Earth Sciences
1857	Outside of the MOO	E. Xilamulun	YJ-8		Granodiorite	129.21	42.46	172		LA-ICP-MS,U-Pb	Zircon	Ma XingHua, et al.	2015	Journal of Asian Earth Sciences
1858	Outside of the MOO	E. Xilamulun	YJ2-7		Quartz monzonite	129.21	42.64	175		LA-ICP-MS,U-Pb	Zircon	Ma XingHua, et al.	2015	Journal of Asian Earth Sciences
1859	Outside of the MOO	E. Xilamulun	YJ2-6		Granodiorite	129.21	42.64	177		LA-ICP-MS,U-Pb	Zircon	Ma XingHua, et al.	2015	Journal of Asian Earth Sciences
1860	Outside of the MOO	E. Xilamulun	YJ-1		Granodiorite	129.21	42.64	201		LA-ICP-MS,U-Pb	Zircon	Ma XingHua, et al.	2015	Journal of Asian Earth Sciences
1861	Outside of the MOO	E. Xilamulun	YJ2-5		Granodiorite	129.22	42.64	190		LA-ICP-MS,U-Pb	Zircon	Ma XingHua, et al.	2015	Journal of Asian Earth Sciences
1862	Outside of the MOO	E. Xilamulun	YJ2-3		Monzogranite	129.22	42.64	197		LA-ICP-MS,U-Pb	Zircon	Ma XingHua, et al.	2015	Journal of Asian Earth Sciences
1863	Outside of the MOO	E. Xilamulun	WQ-6		Monzogranite	130.61	43.56	221		LA-ICP-MS,U-Pb	Zircon	Ma XingHua, et al.	2015	Journal of Asian Earth Sciences
1864	Outside of the MOO	E. Xilamulun		Bajiazi	Quartz diorite	127.52	42.42	218	6.0	SHRIMP,U-Pb	Zircon	Miao, et al.	2005	Doctoral thesis: Jilin University(ICWEA)
1865	Outside of the MOO	E. Xilamulun		Erdaogou	Granodiorite	127.51	42.45	223	2.0	SHRIMP,U-Pb	Zircon	Miao, et al.	2005	Doctoral thesis: Jilin University(ICWEA)
1866	Outside of the MOO	E. Xilamulun	JEDG-4	Erdaogou	Granodiorite	127.51	42.45	223	2.0	SHRIMP,U-Pb	Zircon	Miao, et al.	2015	
1867	Outside of the MOO	E. Xilamulun	HNH-1	Huangnihe	Monzogranite	127.51	42.98	166	2.0	SHRIMP,U-Pb	Zircon	Miao, et al.	2015	
1868	Outside of the MOO	E. Xilamulun		Jilinnanbumayiheyantiheiyunm		127.14	41.85	236	6.0	LA-ICP-MS,U-Pb	Zircon	Pei Fuping .	2008	Doctoral thesis: Jilin University(ICWEA)
1869	Outside of the MOO	E. Xilamulun		Huichangshanchangyan		127.14	41.85	226	3.3	LA-ICP-MS,U-Pb	Zircon	Pei xianzhi, et al.	2007	Journal of Earth Sciences and Environment(ICWEA)
1870	Outside of the MOO	E. Xilamulun		Heilongjiangshengjin jinkuangl		130.77	44.23	220	2.8	LA-ICP-MS,U-Pb	Zircon	Qian ye, et al.	2012	Journal of Earth Sciences and Environment(ICWEA)
1871	Outside of the MOO	E. Xilamulun	STH-2	Shihecuixi	Granodiorite	128.71	44.86	198	3.0	LA-ICP-MS,U-Pb	Zircon	Qin Jihua, et al.	2016	Mineral Deposits(ICWEA)
1872	Outside of the MOO	E. Xilamulun	SMZG-1	Liukesong	Granodiorite	127.88	43.09	263	1.0	LA-ICP-MS,U-Pb	Zircon	S. Liu, et al.	2010	
1873	Outside of the MOO	E. Xilamulun	Bai-1	Baishishan	Granodiorite	127.57	43.58	196		TIMS,U-Pb	Zircon	Sun DeYou, et al.	2001	Acta Petrologica Sinica(ICWEA)
1874	Outside of the MOO	E. Xilamulun		Dayushan	Granodiorite	126.47	43.13	248	4.0	TIMS,U-Pb	Zircon	Sun Deyou, et al.	2004	Acta Petrologica Sinica(ICWEA)
1875	Outside of the MOO	E. Xilamulun	DY0322-1	Caoshanyanti	biotite adamellite	126.76	41.83	177	3.0	LA-ICP-MS,U-Pb	Zircon	Sun Deyou, et al.	2005	Geochemistry(ICWEA)
1876	Outside of the MOO	E. Xilamulun	2.6765e+006	Sandaohe	syenogranite	127.77	43.84	216	3.0	LA-ICP-MS,U-Pb	Zircon	Sun Deyou, et al.	2005	Geochemistry(ICWEA)
1877	Outside of the MOO	E. Xilamulun	MG-7	Tianqiaogang	alkali-feldspar granite	126.98	43.85	182	3.0	SHRIMP,U-Pb	Zircon	Sun Deyou, et al.	2005	Earth Science Frontiers(ICWEA)
1878	Outside of the MOO	E. Xilamulun	2.85547e+006	Tianqiaogang	alkali-feldspar granite	126.98	43.85	190	2.0	LA-ICP-MS,U-Pb	Zircon	Sun Deyou, et al.	2005	Earth Science Frontiers(ICWEA)
1879	Outside of the MOO	E. Xilamulun		Tianqiaogang	Alkali feldspar granite	126.98	43.85	182	3.0	SHRIMP,U-Pb	Zircon	Sun Deyou, et al.	2005	Earth Science Frontiers(ICWEA)
1880	Outside of the MOO	E. Xilamulun	2.85912e+006	Sandaohe	syenogranite	127.77	43.89	216	3.0	LA-ICP-MS,U-Pb	Zircon	Sun Deyou, et al.	2005	Earth Science Frontiers(ICWEA)
1881	Outside of the MOO	E. Xilamulun		Sandaohe	Syenogranite	127.77	43.89	216	3.0	LA-ICP-MS,U-Pb	Zircon	Sun Deyou, et al.	2005	Earth Science Frontiers(ICWEA)
1882	Outside of the MOO	E. Xilamulun		Qingshan	Syenogranite	128.83	42.46	194	3.0	LA-ICP-MS,U-Pb	Zircon	Tang Hongfeng, et al.	2007	Geotectonica et Metallogenia(ICWEA)
1883	Outside of the MOO	E. Xilamulun		Yongxin	Monzogranite	129.32	42.55	236	1.0	LA-ICP-MS,U-Pb	Zircon	Tang Hongfeng, et al.	2007	Geotectonica et Metallogenia(ICWEA)
1884	Outside of the MOO	E. Xilamulun		Yongxin	Monzogranite	129.44	42.59	237	2.0	LA-ICP-MS,U-Pb	Zircon	Tang Hongfeng, et al.	2007	Geotectonica et Metallogenia(ICWEA)
1885	Outside of the MOO	E. Xilamulun	2.77621e+006	Shimen	Granodiorite	128.99	43.04	182		LA-ICP-MS,U-Pb	Zircon	Tang Jie, et al.	2016	Bulletin of Mineralogy, Petrology and Geochemistry(ICWEA)
1886	Outside of the MOO	E. Xilamulun	41667	Sandaohe	syenogranite	127.77	43.89	216	3.0	LA-ICP-MS,U-Pb	Zircon	Tang Jie, et al.	2016	Bulletin of Mineralogy, Petrology and Geochemistry(ICWEA)
1887	Outside of the MOO	E. Xilamulun	SDG8		quartz-diorite	130.91	42.99	258		LA-ICP-MS,U-Pb	Zircon	Wang Wanqiong .	2014	Doctoral thesis: Jilin University(ICWEA)
1888	Outside of the MOO	E. Xilamulun		Liushugou	Monzogranite	128.96	42.77	245	1.0	LA-ICP-MS,U-Pb	Zircon	Wang Zhiwei .	2017	Doctoral thesis: Jilin University(ICWEA)
1889	Outside of the MOO	E. Xilamulun		Mengshan	Quartz monzonite	128.87	42.77	249	1.0	LA-ICP-MS,U-Pb	Zircon	Wang Zhiwei .	2017	Doctoral thesis: Jilin University(ICWEA)
1890	Outside of the MOO	E. Xilamulun		Huangnilingzi	Monzogranite	127.69	42.98	252	1.0	LA-ICP-MS,U-Pb	Zircon	Wang Zhiwei .	2017	Doctoral thesis: Jilin University(ICWEA)
1891	Outside of the MOO	E. Xilamulun		Yitong	Monzogranite	125.29	43.29	244	2.0	LA-ICP-MS,U-Pb	Zircon	Wang Zhiwei .	2017	Doctoral thesis: Jilin University(ICWEA)
1892	Outside of the MOO	E. Xilamulun		Shanhe	Quartz monzonite	126.00	43.45	252	1.0	LA-ICP-MS,U-Pb	Zircon	Wang Zhiwei .	2017	Doctoral thesis: Jilin University(ICWEA)
1893	Outside of the MOO	E. Xilamulun		Xingmengzaoshandainanyuan		128.20	42.93	246	1.0	LA-ICP-MS,U-Pb	Zircon	Wang Zijin, et al.	2013	Geological Bulletin of China(ICWEA)
1894	Outside of the MOO	E. Xilamulun		Xingmengzaoshandainanyuan		127.36	44.00	275	3.0	LA-ICP-MS,U-Pb	Zircon	Wang Zijin, et al.	2013	Geological Bulletin of China(ICWEA)
1895	Outside of the MOO	E. Xilamulun	13JH28-1		Biotite monzogranite	128.96	42.77	245		LA-ICP-MS,U-Pb	Zircon	Wang Zijin, et al.	2016	Doctoral thesis: Jilin University(ICWEA)
1896	Outside of the MOO	E. Xilamulun	13YB3-2		Quartz monzonite	128.87	42.77	249		LA-ICP-MS,U-Pb	Zircon	Wang Zijin, et al.	2016	Doctoral thesis: Jilin University(ICWEA)
1897	Outside of the MOO	E. Xilamulun	13HJD-1		Syenogranitic porphyry	131.40	45.13	203		LA-ICP-MS,U-Pb	Zircon	Wang Zijin, et al.	2016	Doctoral thesis: Jilin University(ICWEA)
1898	Outside of the MOO	E. Xilamulun	M9A		Granodiorite	130.69	45.16	258		SHRIMP,U-Pb	Zircon	WU Cailai, et al.	2000	Acta Geologica Sinica(ICWEA)
1899	Outside of the MOO	E. Xilamulun	2.88472e+006	Chushan	Granodiorite	130.04	45.12	254	4.0	SHRIMP,U-Pb	Zircon	Wu Fuyuan, et al.	2001	Acta Petrologica Sinica(ICWEA)
1900	Outside of the MOO	E. Xilamulun	2.86606e+006	Wujimi	Monzogranite	127.80	45.20	180	3.0	SHRIMP,U-Pb	Zircon	Wu Fuyuan, et al.	2001	Acta Petrologica Sinica(ICWEA)
1901	Outside of the MOO	E. Xilamulun	99SW109	Hongqiling	淡色gabbro	126.42	42.90	216	5.0	SHRIMP,U-Pb	Zircon	Wu Fuyuan, et al.	2004	Earthquake Research in China(ICWEA)
1902	Outside of the MOO	E. Xilamulun	P4-5	Pianhechuan	plagiogranite	127.38	43.26	217	3.0	SHRIMP,U-Pb	Zircon	Wu Fuyuan, et al.	2004	Earthquake Research in China(ICWEA)
1903	Outside of the MOO	E. Xilamulun	MG-143	Fangniugou	Monzogranite	124.78	42.27	261	20.0	SHRIMP,U-Pb	Zircon	Wu Fuyuan, et al.	2010	Journal of Asian Earth Sciences
1904	Outside of the MOO	E. Xilamulun	MG-119	Jianshanzi	Monzogranite	124.76	42.30	254	8.0	SHRIMP,U-Pb	Zircon	Wu Fuyuan, et al.	2010	Journal of Asian Earth Sciences
1905	Outside of the MOO	E. Xilamulun	MG-139	Tukouzi	Granodiorite	124.91	42.33	163	4.0	SHRIMP,U-Pb	Zircon	Wu Fuyuan, et al.	2010	Journal of Asian Earth Sciences
1906	Outside of the MOO	E. Xilamulun	MG-36	Tukouzi	Granodiorite	125.08	42.35	163	7.0	SHRIMP,U-Pb	Zircon	Wu Fuyuan, et al.	2010	Journal of Asian Earth Sciences
1907	Outside of the MOO	E. Xilamulun	MG-28	Songshuzui	Granodiorite	124.93	42.37	243	5.0	SHRIMP,U-Pb	Zircon	Wu Fuyuan, et al.	2010	Journal of Asian Earth Sciences
1908	Outside of the MOO	E. Xilamulun	MG-108	Jianshanzi	Granodiorite	124.66	42.40	163	2.0	SHRIMP,U-Pb	Zircon	Wu Fuyuan, et al.	2010	Journal of Asian Earth Sciences
1909	Outside of the MOO	E. Xilamulun	MG-21	Dasanjiazi	diorite	124.47	42.41	174	3.0	SHRIMP,U-Pb	Zircon	Wu Fuyuan, et al.	2010	Journal of Asian Earth Sciences
1910	Outside of the MOO	E. Xilamulun	DY036-6	Dasanjianzi	diorite	124.47	42.41	178	3.0	LA-ICP-MS,U-Pb	Zircon	Wu Fuyuan, et al.	2010	Journal of Asian Earth Sciences
1911	Outside of the MOO	E. Xilamulun	MG-103	Jianshanzi	Granodiorite	124.68	42.42	243	5.0	SHRIMP,U-Pb	Zircon	Wu Fuyuan, et al.	2010	Journal of Asian Earth Sciences
1912	Outside of the MOO	E. Xilamulun	MG-182	Erlingba	quartz diorite	124.54	42.42	169	3.0	SHRIMP,U-Pb	Zircon	Wu Fuyuan, et al.	2010	Journal of Asian Earth Sciences
1913	Outside of the MOO	E. Xilamulun	MG-40	Heishantou	Monzogranite	125.55	42.43	163	4.0	SHRIMP,U-Pb	Zircon	Wu Fuyuan, et al.	2010	Journal of Asian Earth Sciences
1914	Outside of the MOO	E. Xilamulun	DY053-2	Hudingzi	Granodiorite	125.49	42.46	167	3.0	LA-ICP-MS,U-Pb	Zircon	Wu Fuyuan, et al.	2010	Journal of Asian Earth Sciences
1915	Outside of the MOO	E. Xilamulun	YZ02-52	Dadong	Granodiorite	129.62	42.46	190	3.0	LA-ICP-MS,U-Pb	Zircon	Wu Fuyuan, et al.	2010	Journal of Asian Earth Sciences
1916	Outside of the MOO	E. Xilamulun	DY051-1	Tuanshanzi	Granodiorite	125.43	42.47	175	2.0	LA-ICP-MS,U-Pb	Zircon	Wu Fuyuan, et al.	2010	Journal of Asian Earth Sciences
1917	Outside of the MOO	E. Xilamulun	MG-38	Hudingzi	Monzogranite	125.52	42.49	165	9.0	SHRIMP,U-Pb	Zircon	Wu Fuyuan, et al.	2010	Journal of Asian Earth Sciences
1918	Outside of the MOO	E. Xilamulun	MG-32	Helong	Monzogranite	124.80	42.51	163	4.0	SHRIMP,U-Pb	Zircon	Wu Fuyuan, et al.	2010	Journal of Asian Earth Sciences
1919	Outside of the MOO	E. Xilamulun	MG-64	Xiaosiping	Monzogranite	125.19	42.55	242	7.0	SHRIMP,U-Pb	Zircon	Wu Fuyuan, et al.	2010	Journal of Asian Earth Sciences
1920	Outside of the MOO	E. Xilamulun	MG-15	Fangmu	quartz diorite	124.62	42.60	175	6.0	SHRIMP,U-Pb	Zircon	Wu Fuyuan, et al.	2010	Journal of Asian Earth Sciences
1921	Outside of the MOO	E. Xilamulun	MG-16	Liushugou	Granodiorite	124.56	42.61	158	4.0	SHRIMP,U-Pb	Zircon	Wu Fuyuan, et al.	2010	Journal of Asian Earth Sciences
1922	Outside of the MOO	E. Xilamulun	DY0504-2	Zhiancun	syenogranite	126.46	42.76	182	3.0	LA-ICP-MS,U-Pb	Zircon	Wu Fuyuan, et al.	2010	Journal of Asian Earth Sciences
1923	Outside of the MOO	E. Xilamulun	DY0506-1	Xingnongcun	syenogranite	126.53	42.79	185	2.0	LA-ICP-MS,U-Pb	Zircon	Wu Fuyuan, et al.	2010	Journal of Asian Earth Sciences
1924	Outside of the MOO	E. Xilamulun	DY126-2	Qingyang	syenogranite	125.84	42.91	259	3.0	LA-ICP-MS,U-Pb	Zircon	Wu Fuyuan, et al.	2010	Journal of Asian Earth Sciences
1925	Outside of the MOO	E. Xilamulun	MG-12	Renao	Monzogranite	125.20	42.93	171	6.0	SHRIMP,U-Pb	Zircon	Wu Fuyuan, et al.	2010	Journal of Asian Earth Sciences
1926	Outside of the MOO	E. Xilamulun	2.92532e+006	Xiangshuiyuanzi	Monzogranite	125.36	42.95	184	3.0	TIMS,U-Pb	Zircon	Wu Fuyuan, et al.	2010	Journal of Asian Earth Sciences
1927	Outside of the MOO	E. Xilamulun	FW00-122	Dakangshan	birkremite	125.86	42.97	264	5.0	SHRIMP,U-Pb	Zircon	Wu Fuyuan, et al.	2010	Journal of Asian Earth Sciences
1928	Outside of the MOO	E. Xilamulun	2.92377e+006	Tiande	Monzogranite	124.79	42.99	162	3.0	LA-ICP-MS,U-Pb	Zircon	Wu Fuyuan, et al.	2010	Journal of Asian Earth Sciences
1929	Outside of the MOO	E. Xilamulun	MG-13	Tiande	Monzogranite	124.80	42.99	158	3.0	SHRIMP,U-Pb	Zircon	Wu Fuyuan, et al.	2010	Journal of Asian Earth Sciences
1930	Outside of the MOO	E. Xilamulun	SCS01-1	Shancheshan	Granodiorite	126.14	42.99	175	3.0	LA-ICP-MS,U-Pb	Zircon	Wu Fuyuan, et al.	2010	Journal of Asian Earth Sciences
1931	Outside of the MOO	E. Xilamulun	DY0509-5	Liushuhe	diorite	126.59	42.99	182	1.0	LA-ICP-MS,U-Pb	Zircon	Wu Fuyuan, et al.	2010	Journal of Asian Earth Sciences

1932	Outside of the MOO	E. Xilamulun	FW02-197	Shimenyanti	Granodiorite	128.83	43.02	183	4.0	LA-ICP-MS,U-Pb	Zircon	Wu Fuyuan, et al.	2010	Journal of Asian Earth Sciences
1933	Outside of the MOO	E. Xilamulun	DY143-2	Anyi	Monzogranite	125.11	43.04	252	2.0	LA-ICP-MS,U-Pb	Zircon	Wu Fuyuan, et al.	2010	Journal of Asian Earth Sciences
1934	Outside of the MOO	E. Xilamulun	07YB36		Syenite	128.49	43.07	225		LA-ICP-MS,U-Pb	Zircon	Wu Fuyuan, et al.	2010	Journal of Asian Earth Sciences
1935	Outside of the MOO	E. Xilamulun	DY118-1	Baishishan	Granodiorite	126.75	43.09	182	2.0	LA-ICP-MS,U-Pb	Zircon	Wu Fuyuan, et al.	2010	Journal of Asian Earth Sciences
1936	Outside of the MOO	E. Xilamulun	DY124-2	Dahongshilazi	alkali-feldspar granite	125.85	43.10	260	3.0	LA-ICP-MS,U-Pb	Zircon	Wu Fuyuan, et al.	2010	Journal of Asian Earth Sciences
1937	Outside of the MOO	E. Xilamulun	97103-1	Yima	Granodiorite	126.33	43.10	170	1.0	LA-ICP-MS,U-Pb	Zircon	Wu Fuyuan, et al.	2010	Journal of Asian Earth Sciences
1938	Outside of the MOO	E. Xilamulun	DY123-4	Dahongshilazi	alkali-feldspar granite	125.96	43.11	251	2.0	LA-ICP-MS,U-Pb	Zircon	Wu Fuyuan, et al.	2010	Journal of Asian Earth Sciences
1939	Outside of the MOO	E. Xilamulun	FW02-191	Erhedian	alkali-feldspar granite	128.01	43.11	253	2.0	LA-ICP-MS,U-Pb	Zircon	Wu Fuyuan, et al.	2010	Journal of Asian Earth Sciences
1940	Outside of the MOO	E. Xilamulun	2.93034e+006	Dayushu	Granodiorite	126.47	43.13	248	4.0	TIMS,U-Pb	Zircon	Wu Fuyuan, et al.	2010	Journal of Asian Earth Sciences
1941	Outside of the MOO	E. Xilamulun	DY141-1	Zumin	Monzogranite	125.13	43.17	163	1.0	LA-ICP-MS,U-Pb	Zircon	Wu Fuyuan, et al.	2010	Journal of Asian Earth Sciences
1942	Outside of the MOO	E. Xilamulun	DY023-2	Qingniushan	syenogranite	125.28	43.25	178	2.0	LA-ICP-MS,U-Pb	Zircon	Wu Fuyuan, et al.	2010	Journal of Asian Earth Sciences
1943	Outside of the MOO	E. Xilamulun	DY020-1	Quanyangou	Monzogranite	125.29	43.29	178	4.0	LA-ICP-MS,U-Pb	Zircon	Wu Fuyuan, et al.	2010	Journal of Asian Earth Sciences
1944	Outside of the MOO	E. Xilamulun	2.92304e+006	Shichangtun	Granodiorite	124.78	43.33	184	2.0	TIMS,U-Pb	Zircon	Wu Fuyuan, et al.	2010	Journal of Asian Earth Sciences
1945	Outside of the MOO	E. Xilamulun	DY081-1	Shichangtun	quartz diorite	124.76	43.36	184	2.0	TIMS,U-Pb	Zircon	Wu Fuyuan, et al.	2010	Journal of Asian Earth Sciences
1946	Outside of the MOO	E. Xilamulun	2.9227e+006	Daduanshugou	Monzogranite	124.81	43.37	176	3.0	LA-ICP-MS,U-Pb	Zircon	Wu Fuyuan, et al.	2010	Journal of Asian Earth Sciences
1947	Outside of the MOO	E. Xilamulun	DY018-1	Quchaihe	Monzogranite	126.18	43.39	177	2.0	LA-ICP-MS,U-Pb	Zircon	Wu Fuyuan, et al.	2010	Journal of Asian Earth Sciences
1948	Outside of the MOO	E. Xilamulun	FW02-194	Liushuheyanti	quartz diorite	128.45	43.45	253	2.0	LA-ICP-MS,U-Pb	Zircon	Wu Fuyuan, et al.	2010	Journal of Asian Earth Sciences
1949	Outside of the MOO	E. Xilamulun	98SW122	Baishishan	Granodiorite	127.57	43.58	190	2.0	SHRIMP,U-Pb	Zircon	Wu Fuyuan, et al.	2010	Journal of Asian Earth Sciences
1950	Outside of the MOO	E. Xilamulun	FW02-184	Baishishan	Granodiorite	127.58	43.57	181	2.0	LA-ICP-MS,U-Pb	Zircon	Wu Fuyuan, et al.	2010	Journal of Asian Earth Sciences
1951	Outside of the MOO	E. Xilamulun	FW02-188	Baishishan	Granodiorite	127.75	43.65	187	4.0	LA-ICP-MS,U-Pb	Zircon	Wu Fuyuan, et al.	2010	Journal of Asian Earth Sciences
1952	Outside of the MOO	E. Xilamulun	YB03-176	Laosongling	Monzogranite	129.37	43.83	197	2.0	LA-ICP-MS,U-Pb	Zircon	Wu Fuyuan, et al.	2010	Journal of Asian Earth Sciences
1953	Outside of the MOO	E. Xilamulun	DY144-1	Beishan	Syenogranite	126.53	43.85	166	2.0	LA-ICP-MS,U-Pb	Zircon	Wu Fuyuan, et al.	2010	Journal of Asian Earth Sciences
1954	Outside of the MOO	E. Xilamulun	98SW125	Tiangang	Monzogranite	126.92	43.90	175	3.0	SHRIMP,U-Pb	Zircon	Wu Fuyuan, et al.	2010	Journal of Asian Earth Sciences
1955	Outside of the MOO	E. Xilamulun	98SW126	Tiangang	Dioritic enclave	126.92	43.90	175	4.0	SHRIMP,U-Pb	Zircon	Wu Fuyuan, et al.	2010	Journal of Asian Earth Sciences
1956	Outside of the MOO	E. Xilamulun	98SW124	Jiangmifeng	Granodiorite	126.73	43.97	173	4.0	SHRIMP,U-Pb	Zircon	Wu Fuyuan, et al.	2010	Journal of Asian Earth Sciences
1957	Outside of the MOO	E. Xilamulun	DY0519-1	Weicaohe	Monzogranite	128.01	44.04	266	1.0	LA-ICP-MS,U-Pb	Zircon	Wu Fuyuan, et al.	2010	Journal of Asian Earth Sciences
1958	Outside of the MOO	E. Xilamulun	YB03-94	Xidahe	Granodiorite	130.57	44.07	195	2.0	LA-ICP-MS,U-Pb	Zircon	Wu Fuyuan, et al.	2010	Journal of Asian Earth Sciences
1959	Outside of the MOO	E. Xilamulun	YB03-77	Shahezi	Syenogranite	130.75	44.15	216	4.0	LA-ICP-MS,U-Pb	Zircon	Wu Fuyuan, et al.	2010	Journal of Asian Earth Sciences
1960	Outside of the MOO	E. Xilamulun	YB03-81	Shahezi	Granodiorite	130.74	44.18	197	4.0	LA-ICP-MS,U-Pb	Zircon	Wu Fuyuan, et al.	2010	Journal of Asian Earth Sciences
1961	Outside of the MOO	E. Xilamulun	YB03-168	Taipinglingyanti	Granodiorite	130.34	44.20	204	2.0	LA-ICP-MS,U-Pb	Zircon	Wu Fuyuan, et al.	2010	Journal of Asian Earth Sciences
1962	Outside of the MOO	E. Xilamulun	DY105-2	Xiangshui	Monzogranite	126.91	44.24	172	3.0	LA-ICP-MS,U-Pb	Zircon	Wu Fuyuan, et al.	2010	Journal of Asian Earth Sciences
1963	Outside of the MOO	E. Xilamulun	YB03-108	Ziyang	Monzogranite	130.83	44.29	209	3.0	LA-ICP-MS,U-Pb	Zircon	Wu Fuyuan, et al.	2010	Journal of Asian Earth Sciences
1964	Outside of the MOO	E. Xilamulun	YB03-64	Badaoqiaoyanti	Granodiorite	131.01	44.30	193	3.0	LA-ICP-MS,U-Pb	Zircon	Wu Fuyuan, et al.	2010	Journal of Asian Earth Sciences
1965	Outside of the MOO	E. Xilamulun	DY104-2	Shulan	Granodiorite	126.89	44.34	190	2.0	LA-ICP-MS,U-Pb	Zircon	Wu Fuyuan, et al.	2010	Journal of Asian Earth Sciences
1966	Outside of the MOO	E. Xilamulun	SLS01-1	Silengshan	alkali-feldspar granite	126.28	44.39	314	5.0	TIMS,U-Pb	Zircon	Wu Fuyuan, et al.	2010	Journal of Asian Earth Sciences
1967	Outside of the MOO	E. Xilamulun	DY0545-1	Jiujiecun	syenogranite	129.06	44.39	185	2.0	LA-ICP-MS,U-Pb	Zircon	Wu Fuyuan, et al.	2010	Journal of Asian Earth Sciences
1968	Outside of the MOO	E. Xilamulun	YB03-127	Jiguanlazi	Monzogranite	130.52	44.39	227	4.0	LA-ICP-MS,U-Pb	Zircon	Wu Fuyuan, et al.	2010	Journal of Asian Earth Sciences
1969	Outside of the MOO	E. Xilamulun	YB03-122	Taipingling	Tonalite	130.68	44.39	205	2.0	LA-ICP-MS,U-Pb	Zircon	Wu Fuyuan, et al.	2010	Journal of Asian Earth Sciences
1970	Outside of the MOO	E. Xilamulun	DY103-2	Liangjiashan	alkali-feldspar granite	126.78	44.43	191	2.0	LA-ICP-MS,U-Pb	Zircon	Wu Fuyuan, et al.	2010	Journal of Asian Earth Sciences
1971	Outside of the MOO	E. Xilamulun	YB03-113	Taipingling	Monzogranite	130.76	44.43	205	5.0	LA-ICP-MS,U-Pb	Zircon	Wu Fuyuan, et al.	2010	Journal of Asian Earth Sciences
1972	Outside of the MOO	E. Xilamulun	DY0535-1	Shihecun	syenogranite	129.26	44.50	192	1.0	LA-ICP-MS,U-Pb	Zircon	Wu Fuyuan, et al.	2010	Journal of Asian Earth Sciences
1973	Outside of the MOO	E. Xilamulun	DY0540-1	Fahecun	syenogranite	128.81	44.53	196	1.0	LA-ICP-MS,U-Pb	Zircon	Wu Fuyuan, et al.	2010	Journal of Asian Earth Sciences
1974	Outside of the MOO	E. Xilamulun	97SW008	Yimianpo	Felsic dyke	128.91	44.93	147	10.0	SHRIMP,U-Pb	Zircon	Wu Fuyuan, et al.	2010	Journal of Asian Earth Sciences
1975	Outside of the MOO	E. Xilamulun	97SW009	Hufeng	Monzogranite	128.91	44.93	179	7.0	SHRIMP,U-Pb	Zircon	Wu Fuyuan, et al.	2010	Journal of Asian Earth Sciences
1976	Outside of the MOO	E. Xilamulun	97SW005	Yimianpo	Granodiorite	128.13	45.08	183	4.0	SHRIMP,U-Pb	Zircon	Wu Fuyuan, et al.	2010	Journal of Asian Earth Sciences
1977	Outside of the MOO	E. Xilamulun	DY0559-1	Xincuntun	Monzogranite	128.28	45.18	332	1.0	LA-ICP-MS,U-Pb	Zircon	Wu Fuyuan, et al.	2010	Journal of Asian Earth Sciences
1978	Outside of the MOO	E. Xilamulun	97SW001	Wujimi	Syenogranite	127.80	45.20	180	3.0	SHRIMP,U-Pb	Zircon	Wu Fuyuan, et al.	2010	Journal of Asian Earth Sciences
1979	Outside of the MOO	E. Xilamulun	YZ02-27-2	Bailiping	Monzogranite	128.83	42.05	248	2.0	LA-ICP-MS,U-Pb	Zircon	Wu Fuyuan, et al.	2011	Journal of Asian Earth Sciences
1980	Outside of the MOO	E. Xilamulun	YZ02-28	Bailiping	diorite	128.90	42.07	178	2.0	LA-ICP-MS,U-Pb	Zircon	Wu Fuyuan, et al.	2011	Journal of Asian Earth Sciences
1981	Outside of the MOO	E. Xilamulun	MG-142	Hongshilazi	Monzogranite	124.87	42.17	172	3.0	SHRIMP,U-Pb	Zircon	Wu Fuyuan, et al.	2011	Journal of Asian Earth Sciences
1982	Outside of the MOO	E. Xilamulun	YZ02-40	Liudong	Granodiorite	129.16	42.18	246	3.0	LA-ICP-MS,U-Pb	Zircon	Wu Fuyuan, et al.	2011	Journal of Asian Earth Sciences
1983	Outside of the MOO	E. Xilamulun	YZ02-25-2	Bailiping	Monzogranite	128.75	42.18	245	3.0	LA-ICP-MS,U-Pb	Zircon	Wu Fuyuan, et al.	2011	Journal of Asian Earth Sciences
1984	Outside of the MOO	E. Xilamulun	YZ02-22-2	Bailiping	Monzogranite	128.82	42.20	245	6.0	LA-ICP-MS,U-Pb	Zircon	Wu Fuyuan, et al.	2011	Journal of Asian Earth Sciences
1985	Outside of the MOO	E. Xilamulun	YZ02-43	Liudong	quartz diorite	129.18	42.23	220	2.0	LA-ICP-MS,U-Pb	Zircon	Wu Fuyuan, et al.	2011	Journal of Asian Earth Sciences
1986	Outside of the MOO	E. Xilamulun	YZ02-16-1	Bailiping	syenogranite	128.66	42.37	187	3.0	LA-ICP-MS,U-Pb	Zircon	Wu Fuyuan, et al.	2011	Journal of Asian Earth Sciences
1987	Outside of the MOO	E. Xilamulun	YZ02-33	Gaoling	Granodiorite	129.26	42.46	170	3.0	LA-ICP-MS,U-Pb	Zircon	Wu Fuyuan, et al.	2011	Journal of Asian Earth Sciences
1988	Outside of the MOO	E. Xilamulun	YZ02-12-3	Bailiping	tonalite	128.94	42.48	285	9.0	LA-ICP-MS,U-Pb	Zircon	Wu Fuyuan, et al.	2011	Journal of Asian Earth Sciences
1989	Outside of the MOO	E. Xilamulun	YZ02-50	Tianfozhishan	Monzogranite	129.62	42.58	196	7.0	LA-ICP-MS,U-Pb	Zircon	Wu Fuyuan, et al.	2011	Journal of Asian Earth Sciences
1990	Outside of the MOO	E. Xilamulun	YZ02-45	Gaoling	Monzogranite	129.20	42.62	192	2.0	LA-ICP-MS,U-Pb	Zircon	Wu Fuyuan, et al.	2011	Journal of Asian Earth Sciences
1991	Outside of the MOO	E. Xilamulun	YZ02-49	Longyandong	quartz diorite	129.50	42.66	251	2.0	LA-ICP-MS,U-Pb	Zircon	Wu Fuyuan, et al.	2011	Journal of Asian Earth Sciences
1992	Outside of the MOO	E. Xilamulun	FW00-43	Huangniling	Monzogranite	127.88	42.80	158	3.0	TIMS,U-Pb	Zircon	Wu Fuyuan, et al.	2011	Journal of Asian Earth Sciences
1993	Outside of the MOO	E. Xilamulun	YZ02-10	Mengshan	Granodiorite	128.89	42.83	184	2.0	LA-ICP-MS,U-Pb	Zircon	Wu Fuyuan, et al.	2011	Journal of Asian Earth Sciences
1994	Outside of the MOO	E. Xilamulun	FW00-37	Huangniling	Monzogranite	128.04	42.87	168	3.0	TIMS,U-Pb	Zircon	Wu Fuyuan, et al.	2011	Journal of Asian Earth Sciences
1995	Outside of the MOO	E. Xilamulun	YZ02-2	Dakai	Monzogranite	128.51	42.87	249	4.0	LA-ICP-MS,U-Pb	Zircon	Wu Fuyuan, et al.	2011	Journal of Asian Earth Sciences
1996	Outside of the MOO	E. Xilamulun	FW00-45	Xiaobutun	quartz diorite	128.39	42.91	260	2.0	LA-ICP-MS,U-Pb	Zircon	Wu Fuyuan, et al.	2011	Journal of Asian Earth Sciences
1997	Outside of the MOO	E. Xilamulun	YZ02-5	Mengshan	Monzogranite	128.72	42.91	174	3.0	LA-ICP-MS,U-Pb	Zircon	Wu Fuyuan, et al.	2011	Journal of Asian Earth Sciences
1998	Outside of the MOO	E. Xilamulun	YZ02-7	Mengshan	Granodiorite	128.71	42.93	181	2.0	LA-ICP-MS,U-Pb	Zircon	Wu Fuyuan, et al.	2011	Journal of Asian Earth Sciences
1999	Outside of the MOO	E. Xilamulun	YZ02-1	Liangbing	alkali-feldspar granite	128.42	42.96	190	2.0	LA-ICP-MS,U-Pb	Zircon	Wu Fuyuan, et al.	2011	Journal of Asian Earth Sciences
2000	Outside of the MOO	E. Xilamulun	YZ02-4	Xinhe	Gt Monzogranite	128.53	42.96	179	2.0	LA-ICP-MS,U-Pb	Zircon	Wu Fuyuan, et al.	2011	Journal of Asian Earth Sciences
2001	Outside of the MOO	E. Xilamulun	FW00-56	Yushuchuan	Monzogranite	129.11	42.98	186	1.0	LA-ICP-MS,U-Pb	Zircon	Wu Fuyuan, et al.	2011	Journal of Asian Earth Sciences
2002	Outside of the MOO	E. Xilamulun	FW00-116	Dahuanggou	diorite	130.63	42.98	201	10.0	TIMS,U-Pb	Zircon	Wu Fuyuan, et al.	2011	Journal of Asian Earth Sciences
2003	Outside of the MOO	E. Xilamulun	FW00-197	Shimen	Granodiorite	128.83	43.02	183	4.0	LA-ICP-MS,U-Pb	Zircon	Wu Fuyuan, et al.	2011	Journal of Asian Earth Sciences
2004	Outside of the MOO	E. Xilamulun	FW00-104	Shiren	alkali-feldspar granite	129.47	43.03	253	2.0	LA-ICP-MS,U-Pb	Zircon	Wu Fuyuan, et al.	2011	Journal of Asian Earth Sciences
2005	Outside of the MOO	E. Xilamulun	FW00-58	Miantian	Granodiorite	129.66	43.03	189	1.0	LA-ICP-MS,U-Pb	Zircon	Wu Fuyuan, et al.	2011	Journal of Asian Earth Sciences
2006	Outside of the MOO	E. Xilamulun	FW00-54	Shimen	Granodiorite	128.99	43.04	182	2.0	LA-ICP-MS,U-Pb	Zircon	Wu Fuyuan, et al.	2011	Journal of Asian Earth Sciences
2007	Outside of the MOO	E. Xilamulun	FW00-120	Dahuanggou	Granodiorite	130.65	43.05	203	2.0	TIMS,U-Pb	Zircon	Wu Fuyuan, et al.	2011	Journal of Asian Earth Sciences
2008	Outside of the MOO	E. Xilamulun	FW00-50	Qinglinzi	syenite	128.55	43.07	223	1.0	LA-ICP-MS,U-Pb	Zircon	Wu Fuyuan, et al.	2011	Journal of Asian Earth Sciences
2009	Outside of the MOO	E. Xilamulun	07YB23	Qinglinzi	gabbro	128.55	43.08	229	2.0	LA-ICP-MS,U-Pb	Zircon	Wu Fuyuan, et al.	2011	Journal of Asian Earth Sciences

2010	Outside of the MOO	E. Xilamulun	FW00-66	Sandaogou	Monzogranite	130.73	43.08	205	1.0	LA-ICP-MS,U-Pb	Zircon	Wu Fuyuan, et al.	2011	Journal of Asian Earth Sciences
2011	Outside of the MOO	E. Xilamulun	FW00-191	Erhedian	alkali-feldspar granite	128.01	43.11	253	2.0	LA-ICP-MS,U-Pb	Zircon	Wu Fuyuan, et al.	2011	Journal of Asian Earth Sciences
2012	Outside of the MOO	E. Xilamulun	FW00-110	Liangbing	Monzogranite	128.85	43.13	247	1.0	TIMS,U-Pb	Zircon	Wu Fuyuan, et al.	2011	Journal of Asian Earth Sciences
2013	Outside of the MOO	E. Xilamulun	FW00-34	Liukesong	diorite	127.90	43.15	247	1.0	SHRIMP,U-Pb	Zircon	Wu Fuyuan, et al.	2011	Journal of Asian Earth Sciences
2014	Outside of the MOO	E. Xilamulun	07YB04	Jianchanggou	syenite	128.76	43.27	228	2.0	LA-ICP-MS,U-Pb	Zircon	Wu Fuyuan, et al.	2011	Journal of Asian Earth Sciences
2015	Outside of the MOO	E. Xilamulun	FW00-193	Changlecun	Monzogranite	128.41	43.41	179	1.0	LA-ICP-MS,U-Pb	Zircon	Wu Fuyuan, et al.	2011	Journal of Asian Earth Sciences
2016	Outside of the MOO	E. Xilamulun	FW00-91	Daxingou	Granodiorite	129.65	43.43	187	1.0	LA-ICP-MS,U-Pb	Zircon	Wu Fuyuan, et al.	2011	Journal of Asian Earth Sciences
2017	Outside of the MOO	E. Xilamulun	YB03-188	Hongshitun	Monzogranite	129.41	43.51	266	3.0	LA-ICP-MS,U-Pb	Zircon	Wu Fuyuan, et al.	2011	Journal of Asian Earth Sciences
2018	Outside of the MOO	E. Xilamulun	YB03-253	Dadingzi	tonalite	129.90	43.58	277	3.0	LA-ICP-MS,U-Pb	Zircon	Wu Fuyuan, et al.	2011	Journal of Asian Earth Sciences
2019	Outside of the MOO	E. Xilamulun	YB03-250	Dadingzi	Granodiorite	129.94	43.60	189	3.0	LA-ICP-MS,U-Pb	Zircon	Wu Fuyuan, et al.	2011	Journal of Asian Earth Sciences
2020	Outside of the MOO	E. Xilamulun	YB03-245	Dadingzi	syenogranite	130.02	43.60	200	3.0	LA-ICP-MS,U-Pb	Zircon	Wu Fuyuan, et al.	2011	Journal of Asian Earth Sciences
2021	Outside of the MOO	E. Xilamulun	FW00-78	Qishiergedingzi	Monzogranite	130.63	43.62	203	2.0	LA-ICP-MS,U-Pb	Zircon	Wu Fuyuan, et al.	2011	Journal of Asian Earth Sciences
2022	Outside of the MOO	E. Xilamulun	FW00-88	Luotuoshan	Monzogranite	129.53	43.64	190	2.0	LA-ICP-MS,U-Pb	Zircon	Wu Fuyuan, et al.	2011	Journal of Asian Earth Sciences
2023	Outside of the MOO	E. Xilamulun	FW00-81	Qishiergedingzi	quartz diorite	130.50	43.65	199	8.0	LA-ICP-MS,U-Pb	Zircon	Wu Fuyuan, et al.	2011	Journal of Asian Earth Sciences
2024	Outside of the MOO	E. Xilamulun	YB03-238	Xidahe	diorite	130.15	43.66	257	5.0	LA-ICP-MS,U-Pb	Zircon	Wu Fuyuan, et al.	2011	Journal of Asian Earth Sciences
2025	Outside of the MOO	E. Xilamulun	YB03-185	Nangoutun	plagiogranite	129.28	43.67	203	3.0	LA-ICP-MS,U-Pb	Zircon	Wu Fuyuan, et al.	2011	Journal of Asian Earth Sciences
2026	Outside of the MOO	E. Xilamulun	YB03-184	Chunyang	syenogranite	129.37	43.70	203	2.0	LA-ICP-MS,U-Pb	Zircon	Wu Fuyuan, et al.	2011	Journal of Asian Earth Sciences
2027	Outside of the MOO	E. Xilamulun	FW00-86	Chunyang	Monzogranite	129.43	43.70	215	5.0	LA-ICP-MS,U-Pb	Zircon	Wu Fuyuan, et al.	2011	Journal of Asian Earth Sciences
2028	Outside of the MOO	E. Xilamulun	YB03-201	Erchazigou	syenogranite	129.74	43.72	218	2.0	LA-ICP-MS,U-Pb	Zircon	Wu Fuyuan, et al.	2011	Journal of Asian Earth Sciences
2029	Outside of the MOO	E. Xilamulun	YB03-214	Xiangshuihezi	Granodiorite	129.85	43.83	187	2.0	LA-ICP-MS,U-Pb	Zircon	Wu Fuyuan, et al.	2011	Journal of Asian Earth Sciences
2030	Outside of the MOO	E. Xilamulun	FW00-73	Naozhi	Monzogranite	130.97	43.89	196	3.0	LA-ICP-MS,U-Pb	Zircon	Wu Fuyuan, et al.	2011	Journal of Asian Earth Sciences
2031	Outside of the MOO	E. Xilamulun	YB03-213	Xiangshuihezi	Granodiorite	129.88	43.94	180	3.0	LA-ICP-MS,U-Pb	Zircon	Wu Fuyuan, et al.	2011	Journal of Asian Earth Sciences
2032	Outside of the MOO	E. Xilamulun	YB03-70	Daxushan	rhyolite	130.76	44.11	213	3.0	LA-ICP-MS,U-Pb	Zircon	Wu Fuyuan, et al.	2011	Journal of Asian Earth Sciences
2033	Outside of the MOO	E. Xilamulun	YB03-66	Badaoqiao	Monzogranite	131.06	44.28	205	3.0	LA-ICP-MS,U-Pb	Zircon	Wu Fuyuan, et al.	2011	Journal of Asian Earth Sciences
2034	Outside of the MOO	E. Xilamulun	YB03-40	Taipingling	tonalite	130.83	44.46	203	3.0	LA-ICP-MS,U-Pb	Zircon	Wu Fuyuan, et al.	2011	Journal of Asian Earth Sciences
2035	Outside of the MOO	E. Xilamulun	YB03-35	Wuhuashan	Granodiorite	131.12	44.54	203	3.0	LA-ICP-MS,U-Pb	Zircon	Wu Fuyuan, et al.	2011	Journal of Asian Earth Sciences
2036	Outside of the MOO	E. Xilamulun	YB03-58	Taipingling	Granodiorite	130.56	44.56	202	2.0	LA-ICP-MS,U-Pb	Zircon	Wu Fuyuan, et al.	2011	Journal of Asian Earth Sciences
2037	Outside of the MOO	E. Xilamulun	YB03-22	Wuhuashan	Granodiorite	131.05	44.58	207	2.0	LA-ICP-MS,U-Pb	Zircon	Wu Fuyuan, et al.	2011	Journal of Asian Earth Sciences
2038	Outside of the MOO	E. Xilamulun	YB03-54	Hongfangzi	Monzogranite	130.61	44.59	206	3.0	LA-ICP-MS,U-Pb	Zircon	Wu Fuyuan, et al.	2011	Journal of Asian Earth Sciences
2039	Outside of the MOO	E. Xilamulun	YB03-50	Xidagangzi	rhyolite	130.82	44.60	203	2.0	LA-ICP-MS,U-Pb	Zircon	Wu Fuyuan, et al.	2011	Journal of Asian Earth Sciences
2040	Outside of the MOO	E. Xilamulun	FW00-121	Dakangshan	birkremite	125.86	42.97	267	1.0	TIMS,U-Pb	Zircon	Wu Guang, et al.	2009	Acta Petrologica Sinica(ICWEA)
2041	Outside of the MOO	E. Xilamulun	DY0540-2		Syenogranite	128.81	44.53	196		LA-ICP-MS,U-Pb	Zircon	Wu Guang, et al.	2009	Acta Petrologica Sinica(ICWEA)
2042	Outside of the MOO	E. Xilamulun		Yanbianhelongdiqushan		129.10	42.53	175	2.0	LA-ICP-MS,U-Pb	Zircon	Wu Pengfei, et al.	2013	Geological Journal of China Universities
2043	Outside of the MOO	E. Xilamulun	97SAW027	Chaihe	Monzogranite	129.69	44.69	254	4.0	SHRIMP,U-Pb	Zircon	WU Zhiping, et al.	2004	Geotectonica et Metallogenia(ICWEA)
2044	Outside of the MOO	E. Xilamulun	97SAW028	Chushan	Granodiorite	130.04	45.12	256	5.0	SHRIMP,U-Pb	Zircon	WU Zhiping, et al.	2004	Geotectonica et Metallogenia(ICWEA)
2045	Outside of the MOO	E. Xilamulun	98SW119	Qingshan	Granodiorite	130.57	45.48	270	4.0	SHRIMP,U-Pb	Zircon	WU Zhiping, et al.	2004	Geotectonica et Metallogenia(ICWEA)
2046	Outside of the MOO	E. Xilamulun	X13	Wuxing	Syenogranite	125.60	43.60	159	3.0	LA-ICP-MS,U-Pb	Zircon	Xu Bowen, et al.	2015	Acta Geologica Sinica(ICWEA)
2047	Outside of the MOO	E. Xilamulun		Xiaoxinganlingzhongbu		126.45	43.19	263	1.0	LA-ICP-MS,U-Pb	Zircon	Xu Meijun, et al.	2013	Acta Petrologica Sinica(ICWEA)
2048	Outside of the MOO	E. Xilamulun		Tianqiaoling	Dacite	129.38	43.34	175	3.0	LA-ICP-MS,U-Pb	Zircon	Xu Wenliang, et al.	2013	Journal of Jilin University（Earth Science Edition）(ICWEA).
2049	Outside of the MOO	E. Xilamulun		Suiyangzhen	Dacite	130.92	44.42	184	2.0	LA-ICP-MS,U-Pb	Zircon	Xu Wenliang, et al.	2013	Journal of Jilin University（Earth Science Edition）(ICWEA).
2050	Outside of the MOO	E. Xilamulun	14GW366	Daerlong	Granodiorite	130.61	45.51	274	2.0	LA-ICP-MS,U-Pb	Zircon	Yang Linchun, et al.	2016	Xinjiang Geology(ICWEA)
2051	Outside of the MOO	E. Xilamulun	14GW364	Daerlong	Granodiorite	130.61	45.51	275	2.0	LA-ICP-MS,U-Pb	Zircon	Yang Linchun, et al.	2016	Xinjiang Geology(ICWEA)
2052	Outside of the MOO	E. Xilamulun		Hunchunnan	Syenogranite	130.53	42.57	199	4.0	LA-ICP-MS,U-Pb	Zircon	Yang Meng, et al.	2015	Acta Petrologica Sinica(ICWEA)
2053	Outside of the MOO	E. Xilamulun		Hunchunnan	Diorite	130.46	42.58	210	3.0	LA-ICP-MS,U-Pb	Zircon	Yang Meng, et al.	2015	Acta Petrologica Sinica(ICWEA)
2054	Outside of the MOO	E. Xilamulun		Hunchunnan	Granodiorite	130.37	42.64	237	5.0	LA-ICP-MS,U-Pb	Zircon	Yang Meng, et al.	2015	Acta Petrologica Sinica(ICWEA)
2055	Outside of the MOO	E. Xilamulun		Hunchunnan	Diorite	130.68	42.66	206	3.0	LA-ICP-MS,U-Pb	Zircon	Yang Meng, et al.	2015	Acta Petrologica Sinica(ICWEA)
2056	Outside of the MOO	E. Xilamulun		Hunchunnan	Diorite	130.41	42.66	245	3.0	LA-ICP-MS,U-Pb	Zircon	Yang Meng, et al.	2015	Acta Petrologica Sinica(ICWEA)
2057	Outside of the MOO	E. Xilamulun		Hunchunnan	Monzogranite	130.46	42.68	212	4.0	LA-ICP-MS,U-Pb	Zircon	Yang Meng, et al.	2015	Acta Petrologica Sinica(ICWEA)
2058	Outside of the MOO	E. Xilamulun		Hunchunnan	Quartz diorite	130.29	42.70	245	4.0	LA-ICP-MS,U-Pb	Zircon	Yang Meng, et al.	2015	Acta Petrologica Sinica(ICWEA)
2059	Outside of the MOO	E. Xilamulun		Hunchunnan	Monzogranite	130.25	42.72	248	5.0	LA-ICP-MS,U-Pb	Zircon	Yang Meng, et al.	2015	Acta Petrologica Sinica(ICWEA)
2060	Outside of the MOO	E. Xilamulun	11GW019		Monzogranite	131.87	45.45	223		LA-ICP-MS,U-Pb	Zircon	Yang Mingchun, et al	2015	Acta Petrologica Sinica(ICWEA)
2061	Outside of the MOO	E. Xilamulun	12GW036		Monzogranite	131.88	45.45	218		LA-ICP-MS,U-Pb	Zircon	Yang Mingchun, et al	2015	Acta Petrologica Sinica(ICWEA)
2062	Outside of the MOO	E. Xilamulun	11HNA11		Granodiorite	128.55	43.83	252		LA-ICP-MS,U-Pb	Zircon	Yu Jiejiang, et al.	2013	Acta Petrologica Sinica(ICWEA)
2063	Outside of the MOO	E. Xilamulun	11HNA4		Monzogranite	128.72	44.10	255		LA-ICP-MS,U-Pb	Zircon	Yu Jiejiang, et al.	2013	Acta Petrologica Sinica(ICWEA)
2064	Outside of the MOO	E. Xilamulun	VV12	Vladivostok Area	Granite	131.75	42.98	250	3.5	LA-ICP-MS,U-Pb	Zircon	Yukiyasu TSUTSUM	2014	Journal of Mineralogical and Petrological Sciencs
2065	Outside of the MOO	E. Xilamulun	VV11	Vladivostok Area	Granite	131.85	42.98	302	2.4	LA-ICP-MS,U-Pb	Zircon	Yukiyasu TSUTSUM	2014	Journal of Mineralogical and Petrological Sciencs
2066	Outside of the MOO	E. Xilamulun	09361-1		Granodiorite	124.29	42.73	173		LA-ICP-MS,U-Pb	Zircon	Zhang Chao, et al.	2014	Acta Petrologica Sinica(ICWEA)
2067	Outside of the MOO	E. Xilamulun	09354-1		Granodiorite	124.72	43.09	164		LA-ICP-MS,U-Pb	Zircon	Zhang Chao, et al.	2014	Acta Petrologica Sinica(ICWEA)
2068	Outside of the MOO	E. Xilamulun		Gaoling	Granodiorite	129.21	42.46	180	1.0	LA-ICP-MS,U-Pb	Zircon	Zhang Chao.	2014	Doctoral thesis: Jilin University(ICWEA)
2069	Outside of the MOO	E. Xilamulun		Yueshandong	Diorite	129.12	42.55	178	2.0	LA-ICP-MS,U-Pb	Zircon	Zhang Chao.	2014	Doctoral thesis: Jilin University(ICWEA)
2070	Outside of the MOO	E. Xilamulun		Shiguoshuiku	Granodiorite	129.21	42.64	193	1.0	LA-ICP-MS,U-Pb	Zircon	Zhang Chao.	2014	Doctoral thesis: Jilin University(ICWEA)
2071	Outside of the MOO	E. Xilamulun	LK14		Granodiorite	124.80	42.33	169		LA-ICP-MS,U-Pb	Zircon	Zhang HaiHong, et al	2017	Doctoral thesis: Jilin University(ICWEA)
2072	Outside of the MOO	E. Xilamulun	LK11		Gabbro	124.34	42.34	163		LA-ICP-MS,U-Pb	Zircon	Zhang HaiHong, et al	2017	Doctoral thesis: Jilin University(ICWEA)
2073	Outside of the MOO	E. Xilamulun	LK07-1		Gabbro	124.17	42.64	175		LA-ICP-MS,U-Pb	Zircon	Zhang HaiHong, et al	2017	Doctoral thesis: Jilin University(ICWEA)
2074	Outside of the MOO	E. Xilamulun	LK05-1		Granodiorite	124.32	42.71	163		LA-ICP-MS,U-Pb	Zircon	Zhang HaiHong, et al	2017	Doctoral thesis: Jilin University(ICWEA)
2075	Outside of the MOO	E. Xilamulun	LK03-1		granodiorite	124.44	42.88	165		LA-ICP-MS,U-Pb	Zircon	Zhang HaiHong, et al	2017	Doctoral thesis: Jilin University(ICWEA)
2076	Outside of the MOO	E. Xilamulun	LK20-2		monzogranite	125.44	43.38	175		LA-ICP-MS,U-Pb	Zircon	Zhang HaiHong, et al	2017	Doctoral thesis: Jilin University(ICWEA)
2077	Outside of the MOO	E. Xilamulun	11LK31-3		Granodiorite	125.02	43.39	170		LA-ICP-MS,U-Pb	Zircon	Zhang HaiHong, et al	2017	Doctoral thesis: Jilin University(ICWEA)
2078	Outside of the MOO	E. Xilamulun	FK53	Faku	Granodiorite	123.26	42.50	265	4.0	SHRIMP,U-Pb	Zircon	Zhang Xiaohui, et al.	2005	Acta Petrologica Sinica(ICWEA)
2079	Outside of the MOO	E. Xilamulun	FK51	Fakuyanti	Granodiorite	123.43	42.50	284	3.0	SHRIMP,U-Pb	Zircon	Zhang Xiaohui, et al.	2005	Acta Petrologica Sinica(ICWEA)
2080	Outside of the MOO	E. Xilamulun	FK04-5	Faku	gabbro	123.49	42.50	241	6.0	SHRIMP,U-Pb	Zircon	Zhang Xiaohui, et al.	2005	Acta Petrologica Sinica(ICWEA)
2081	Outside of the MOO	E. Xilamulun		Bailiping	Monzogranite	128.83	42.05	248	2.0	LA-ICP-MS,U-Pb	Zircon	Zhang Yanbinet al.	2004	Science in China(Series D:Earth Sciences)(ICWEA)
2082	Outside of the MOO	E. Xilamulun		Bailiping	Diorite	128.94	42.07	178	2.0	LA-ICP-MS,U-Pb	Zircon	Zhang Yanbinet al.	2004	Science in China(Series D:Earth Sciences)(ICWEA)
2083	Outside of the MOO	E. Xilamulun		Bailiping	Monzogranite	128.75	42.18	245	3.0	LA-ICP-MS,U-Pb	Zircon	Zhang Yanbinet al.	2004	Science in China(Series D:Earth Sciences)(ICWEA)
2084	Outside of the MOO	E. Xilamulun		Bailiping	Monzogranite	128.82	42.20	245	6.0	LA-ICP-MS,U-Pb	Zircon	Zhang Yanbinet al.	2004	Science in China(Series D:Earth Sciences)(ICWEA)
2085	Outside of the MOO	E. Xilamulun		Bailiping	Syenogranite	128.66	42.37	187	3.0	LA-ICP-MS,U-Pb	Zircon	Zhang Yanbinet al.	2004	Science in China(Series D:Earth Sciences)(ICWEA)
2086	Outside of the MOO	E. Xilamulun		Mengshan	Granodiorite	128.89	42.83	184	2.0	LA-ICP-MS,U-Pb	Zircon	Zhang Yanbinet al.	2004	Science in China(Series D:Earth Sciences)(ICWEA)
2087	Outside of the MOO	E. Xilamulun		Dakai	Monzogranite	128.51	42.87	249	4.0	LA-ICP-MS,U-Pb	Zircon	Zhang Yanbinet al.	2004	Science in China(Series D:Earth Sciences)(ICWEA)

2088	Outside of the MOO	E. Xilamulun		Mengshan	Granodiorite	128.71	42.93	181	2.0	LA-ICP-MS,U-Pb	Zircon	Zhang Yanbinet al.	2004	Science in China(Series D:Earth Sciences)(ICWEA)
2089	Outside of the MOO	E. Xilamulun	FW00-40	Huangniling	Granodiorite	127.64	42.87	171	5.0	SHRIMP,U-Pb	Zircon	Zhang, et al.	2004	Science in China(Series D:Earth Sciences)(ICWEA)
2090	Outside of the MOO	E. Xilamulun	basalt1-6	baishansongjiangzhen	Basalt	128.06	42.05	245	13.0	LA-ICP-MS,U-Pb	Zircon	Zhao Yuandong, et al	2009	Journal of Jilin University（Earth Science Edition）(ICWEA)
2091	Outside of the MOO	E. Xilamulun		Yanbianhelongdiqushan	Granite	129.11	42.14	173	2.0	LA-ICP-MS,U-Pb	Zircon	Zhao Yuandong, et al	2009	Journal of Jilin University（Earth Science Edition）(ICWEA)
2092	Outside of the MOO	E. Xilamulun	D13BSLZ02	Yongjixianshuanghezhenbaishi	Monzogranite	126.13	43.45	174	2.0	LA-ICP-MS,U-Pb	Zircon	Zhao Yuandong, et al	2009	Journal of Jilin University（Earth Science Edition）(ICWEA)
2093	Outside of the MOO	E. Xilamulun	D1018-1	Yongjixianshuanghezhenbaishi	alkali-feldspar granite	127.92	43.55	214	3.0	LA-ICP-MS,U-Pb	Zircon	Zhao Yuandong, et al	2009	Journal of Jilin University（Earth Science Edition）(ICWEA)
2094	Outside of the MOO	E. Xilamulun	D4006-1	Yongjixianshuanghezhenbaishi	Monzogranite	127.85	43.57	203	3.0	LA-ICP-MS,U-Pb	Zircon	Zhao Yuandong, et al	2009	Journal of Jilin University（Earth Science Edition）(ICWEA)
2095	Outside of the MOO	E. Xilamulun	D1052-1	Yongjixianshuanghezhenbaishi	Granodiorite	127.83	43.63	191	2.0	LA-ICP-MS,U-Pb	Zircon	Zhao Yuandong, et al	2009	Journal of Jilin University（Earth Science Edition）(ICWEA)
2096	Outside of the MOO	E. Xilamulun	D1086-1	Yongjixianshuanghezhenbaishi	Granodiorite	127.94	43.64	199	3.0	LA-ICP-MS,U-Pb	Zircon	Zhao Yuandong, et al	2009	Journal of Jilin University（Earth Science Edition）(ICWEA)
2097	Outside of the MOO	E. Xilamulun	D7057-1	Yongjixianshuanghezhenbaishi	Granodiorite	127.95	43.64	198	1.8	LA-ICP-MS,U-Pb	Zircon	Zhao Yuandong, et al	2009	Journal of Jilin University（Earth Science Edition）(ICWEA)
2098	Outside of the MOO	E. Xilamulun	D13NA01	Ninganhongchengcun	granite	129.42	44.35	260	2.5	LA-ICP-MS,U-Pb	Zircon	Zhao Yuandong, et al	2009	Journal of Jilin University（Earth Science Edition）(ICWEA)
2099	Outside of the MOO	E. Xilamulun	Dn015	Taipingling	Granodiorite	130.49	44.39	201	1.0	LA-ICP-MS,U-Pb	Zircon	Zhao Yuandong, et al	2009	Journal of Jilin University（Earth Science Edition）(ICWEA)
2100	Outside of the MOO	E. Xilamulun	D13CSh01	Linkouchushandong	Granodiorite	130.03	45.12	252	2.4	LA-ICP-MS,U-Pb	Zircon	Zhao Yuandong, et al	2009	Journal of Jilin University（Earth Science Edition）(ICWEA)
2101	Outside of the MOO	E. Xilamulun	D13DM01	Linkoudamadanggou	Granite	130.08	45.41	264	3.0	LA-ICP-MS,U-Pb	Zircon	Zhao Yuandong, et al	2009	Journal of Jilin University（Earth Science Edition）(ICWEA)
2102	Outside of the MOO	Jiamusi Block	HLJ-04	Baoqing area	andesite	132.07	46.14	229	4.0	LA-ICP-MS,U-Pb	zircon	Li GY et al.	2020	Geological Journal
2103	Outside of the MOO	Jiamusi Block	HLJ-05	Baoqing area	andesite	132.05	46.20	238	1.0	LA-ICP-MS,U-Pb	zircon	Li GY et al.	2020	Geological Journal
2104	Outside of the MOO	Jiamusi Block	HLJ-30	Baoqing area	andesite	132.05	46.20	239	3.0	LA-ICP-MS,U-Pb	zircon	Li GY et al.	2020	Geological Journal
2105	Outside of the MOO	Jiamusi Block	HLJ-31	Baoqing area	andesite	132.05	46.20	241	1.0	LA-ICP-MS,U-Pb	zircon	Li GY et al.	2020	Geological Journal
2106	Outside of the MOO	NE China	13GW104	Liudao	Alkali feldspar granite	132.10	46.05	272	3.0	LA-ICP-MS,U-Pb	Zircon	Bi J H et al.	2016	Lithos
2107	Outside of the MOO	NE China	13GW090	Fangshan	Monzogranite	132.60	46.05	281	2.0	LA-ICP-MS,U-Pb	Zircon	Bi J H et al.	2016	Lithos
2108	Outside of the MOO	NE China	13GW086	Jianchazhan	Granodiorite	132.62	46.05	302	4.0	LA-ICP-MS,U-Pb	Zircon	Bi J H et al.	2016	Lithos
2109	Outside of the MOO	NE China	13GW124	Liudao	Graphic granite	131.84	46.11	270	2.0	LA-ICP-MS,U-Pb	Zircon	Bi J H et al.	2016	Lithos
2110	Outside of the MOO	NE China	13GW094	Tuanshan	Granodiorite	132.67	46.11	287	3.0	LA-ICP-MS,U-Pb	Zircon	Bi J H et al.	2016	Lithos
2111	Outside of the MOO	NE China	13GW096	Suolun	Monzogranite	132.56	46.11	282	2.0	LA-ICP-MS,U-Pb	Zircon	Bi J H et al.	2016	Lithos
2112	Outside of the MOO	NE China	13GW560	Sifenchang	Monzogranite	132.63	46.17	302	3.0	LA-ICP-MS,U-Pb	Zircon	Bi J H et al.	2016	Lithos
2113	Outside of the MOO	NE China	13GW564	Hongqi	Monzogranite	132.43	46.26	267	2.0	LA-ICP-MS,U-Pb	Zircon	Bi J H et al.	2016	Lithos
2114	Outside of the MOO	NE China	13GW554	Yifenchang	Monzogranite	132.68	46.31	301	2.0	LA-ICP-MS,U-Pb	Zircon	Bi J H et al.	2016	Lithos
2115	Outside of the MOO	NE China	13GW550	Jijiandui	Syenogranite	132.67	46.40	294	2.0	LA-ICP-MS,U-Pb	Zircon	Bi J H et al.	2018	Earth Science Frontiers(ICWEA)
2116	Outside of the MOO	NE China	13GW545	Qifenchang	Monzogranite	132.53	46.41	305	3.0	LA-ICP-MS,U-Pb	Zircon	Bi J H et al.	2018	Earth Science Frontiers(ICWEA)
2117	Outside of the MOO	NE China	10GW255	Shenglicun	Granitic porphyry	131.97	46.45	300	4.0	LA-ICP-MS,U-Pb	Zircon	Bi J H et al.	2018	Earth Science Frontiers(ICWEA)
2118	Outside of the MOO	NE China	13GW157	Jubaoshan	Monzogranite	131.93	46.53	290	2.0	LA-ICP-MS,U-Pb	Zircon	Bi J H et al.	2018	Earth Science Frontiers(ICWEA)
2119	Outside of the MOO	NE China	15GW235	Tuoyaozi	Alkali feldspar granite	130.77	46.24	276	3.0	LA-ICP-MS,U-Pb	Zircon	Dong Y et al.	2017	Gondwana Research
2120	Outside of the MOO	NE China	15GW075	Mengjiagang	Monzogranite	130.65	46.40	272	2.0	LA-ICP-MS,U-Pb	Zircon	Dong Y et al.	2017	Gondwana Research
2121	Outside of the MOO	NE China	15GW073	Mingyi	Granodiorite	130.51	46.44	278	2.0	LA-ICP-MS,U-Pb	Zircon	Dong Y et al.	2017	Gondwana Research
2122	Outside of the MOO	NE China	15GW265	Qingbei	Monzogranite	130.64	46.51	266	2.0	LA-ICP-MS,U-Pb	Zircon	Dong Y et al.	2017	Gondwana Research
2123	Outside of the MOO	NE China	15GW261	Qingbei	Monzogranite	130.65	46.53	263	3.0	LA-ICP-MS,U-Pb	Zircon	Dong Y et al.	2017	Gondwana Research
2124	Outside of the MOO	NE China	15GW248	Hengtoushan	Monzogranite	130.63	46.63	267	3.0	LA-ICP-MS,U-Pb	Zircon	Dong Y et al.	2017	Gondwana Research
2125	Outside of the MOO	NE China	SN122	Zuankong	Monzogranite	124.62	43.81	181	3.0	LA-ICP-MS,U-Pb	Zircon	Gao Yanguang .	2007	Doctoral thesis: China University of Geosciences, Beijing(ICWEA)
2126	Outside of the MOO	NE China		Yanbianhelongdiqu	Diorite	129.11	42.14	173	2.0	LA-ICP-MS,U-Pb	Zircon	Gao Zhenquan et al.	2006	Acta Geologica Sinica(ICWEA)
2127	Outside of the MOO	NE China	Q2-1	Drill Hole	granite	124.58	43.78	161	5.0	LA-ICP-MS,U-Pb	Zircon	Gao Zhenquan et al.	2006	Acta Geologica Sinica(ICWEA)
2128	Outside of the MOO	NE China	SN121	Drill Hole	Monzogranite	124.60	43.79	165	2.0	LA-ICP-MS,U-Pb	Zircon	Gao Zhenquan et al.	2006	Acta Geologica Sinica(ICWEA)
2129	Outside of the MOO	NE China	SN72	Drill Hole	syenogranite	124.62	43.81	161	4.0	LA-ICP-MS,U-Pb	Zircon	Gao Zhenquan et al.	2006	Acta Geologica Sinica(ICWEA)
2130	Outside of the MOO	NE China	H15-69-1	Qingyunshan	Syenogranite	128.42	45.13	189	1.0	LA-ICP-MS,U-Pb	Zircon	Ge M H et al.	2017	Tectonophysics
2131	Outside of the MOO	NE China	H15-63-4	Maoershan	Monzogranite	127.50	45.28	181	1.0	LA-ICP-MS,U-Pb	Zircon	Ge M H et al.	2017	Tectonophysics
2132	Outside of the MOO	NE China	H15-68-1	Yimancun	Syenogranite	127.84	45.32	174	1.0	LA-ICP-MS,U-Pb	Zircon	Ge M H et al.	2017	Tectonophysics
2133	Outside of the MOO	NE China	H15-66-1	Jidiantun	Monzogranite	127.69	45.39	180	1.0	LA-ICP-MS,U-Pb	Zircon	Ge M H et al.	2017	Tectonophysics
2134	Outside of the MOO	NE China	MZ06	Meizuo	Granodiorite	130.67	46.08	259	4.0	LA-ICP-MS,U-Pb	Zircon	Huang Y C et al.	2008	Journal of Jilin University（Earth Science Edition）(ICWEA)
2135	Outside of the MOO	NE China	15XH21–1	Erzhan	Quartz monzodiorite	128.63	44.01	267	2.0	LA-ICP-MS,U-Pb	Zircon	Long X Y et al.	2019	Geological Journal
2136	Outside of the MOO	NE China	15XH22–1	Erzhan	Quartz monzonite	128.64	43.99	267	2.0	LA-ICP-MS,U-Pb	Zircon	Long X Y et al.	2019	Geological Journal
2137	Outside of the MOO	NE China	15XH24–1	Erzhan	Monzogranite	128.54	44.03	271	2.0	LA-ICP-MS,U-Pb	Zircon	Long X Y et al.	2019	Geological Journal
2138	Outside of the MOO	NE China	15XH23–1	Erzhan	Monzogranite	128.54	44.03	262	2.0	LA-ICP-MS,U-Pb	Zircon	Long X Y et al.	2019	Geological Journal
2139	Outside of the MOO	NE China	15XH25–1	Erzhan	Monzogranite	128.51	44.07	263	4.0	LA-ICP-MS,U-Pb	Zircon	Long X Y et al.	2019	Geological Journal
2140	Outside of the MOO	NE China	15XH27–1	Erzhan	Alkali feldspar granite	128.42	44.09	263	2.0	LA-ICP-MS,U-Pb	Zircon	Long X Y et al.	2019	Geological Journal
2141	Outside of the MOO	NE China	15XH26–1	Erzhan	Quartz monzonite	128.49	44.10	262	3.0	LA-ICP-MS,U-Pb	Zircon	Long X Y et al.	2019	Geological Journal
2142	Outside of the MOO	NE China	15XH20–1	Hengdaohezi	Syenogranite	129.34	44.89	260	3.0	LA-ICP-MS,U-Pb	Zircon	Long X Y et al.	2019	Geological Journal
2143	Outside of the MOO	NE China	16XH8–1	Luobei	Granodiorite	130.42	45.40	272	2.0	LA-ICP-MS,U-Pb	Zircon	Long X Y et al.	2019	Geological Journal
2144	Outside of the MOO	NE China	16XH9–1	Boli	Granodiorite	130.26	45.42	258	1.0	LA-ICP-MS,U-Pb	Zircon	Long X Y et al.	2019	Geological Journal
2145	Outside of the MOO	NE China	16XH13–1	Luobei	Monzogranite	130.96	45.45	272	2.0	LA-ICP-MS,U-Pb	Zircon	Long X Y et al.	2019	Geological Journal
2146	Outside of the MOO	NE China	16XH10–1	Boli	Granodiorite	130.10	45.67	256	1.0	LA-ICP-MS,U-Pb	Zircon	Long X Y et al.	2019	Geological Journal
2147	Outside of the MOO	NE China	16XH10–4	Boli	Granite aplite	130.10	45.67	257	3.0	LA-ICP-MS,U-Pb	Zircon	Long X Y et al.	2019	Geological Journal
2148	Outside of the MOO	NE China	15XH16–1	Luobei	Quartz monzonite	129.91	45.69	267	4.0	LA-ICP-MS,U-Pb	Zircon	Long X Y et al.	2019	Geological Journal
2149	Outside of the MOO	NE China	16XH12–1	Boli	Granodiorite	130.14	45.84	261	1.0	LA-ICP-MS,U-Pb	Zircon	Long X Y et al.	2019	Geological Journal
2150	Outside of the MOO	NE China	X-4	Xinhuatun	Granodiorite	120.00	40.00	184	4.0	SHRIMP,U-Pb	Zircon	Wu Fuyuan et al.	2000	Tectonophysics
2151	Outside of the MOO	NE China	97-SAW-005	Dawangzhezi	Granodiorite	128.13	45.08	183	4.0	SHRIMP,U-Pb	Zircon	Wu Fuyuan et al.	2000	Tectonophysics
2152	Outside of the MOO	NE China	M9B	Shichang	Granodiorite	130.69	45.16	267	2.0	SHRIMP,U-Pb	Zircon	Wu Fuyuan et al.	2001	Acta Petrologica Sinica(ICWEA)
2153	Outside of the MOO	NE China	98SAW119	Qingshan	Granodiorite	130.57	45.48	270	4.0	SHRIMP,U-Pb	Zircon	Wu Fuyuan et al.	2001	Acta Petrologica Sinica(ICWEA)
2154	Outside of the MOO	NE China	9718-1	Tianqiaogang	Alkali-feldspar granite	126.98	43.85	190	2.0	TIMS,U-Pb	Zircon	Wu Fuyuan et al.	2002	Chemical Geology
2155	Outside of the MOO	NE China	DY0556-1	Jijiadian	Syenogranite	127.70	45.38	190	1.0	LA-ICP-MS,U-Pb	Zircon	Wu Fuyuan et al.	2011	Journal of Asian Earth Sciences
2156	Outside of the MOO	NE China	97SW101	Yanshouxian	Syenogranite	128.50	45.79	199	5.0	LA-ICP-MS,U-Pb	Zircon	Wu Fuyuan et al.	2011	Journal of Asian Earth Sciences
2157	Outside of the MOO	NE China	98SW104	Yanshouxian	Felsic dyke	128.51	45.80	147	6.0	LA-ICP-MS,U-Pb	Zircon	Wu Fuyuan et al.	2011	Journal of Asian Earth Sciences
2158	Outside of the MOO	NE China	98SW103	Yanshouxian	Granodiorite	128.51	45.80	191	4.0	LA-ICP-MS,U-Pb	Zircon	Wu Fuyuan et al.	2011	Journal of Asian Earth Sciences
2159	Outside of the MOO	E.Songliao basin	10GW251	Jinshan	Granodiorite	131.72	47.03	260	8.0	LA-ICP-MS,U-Pb	Zircon	Bi J H et al.	2014	Journal of Earth Sciences and Environment(ICWEA)
2160	Outside of the MOO	E.Songliao basin	13GW197	Jinshan	Monzogranite	131.71	47.08	278	3.0	LA-ICP-MS,U-Pb	Zircon	Bi J H et al.	2014	Journal of Earth Sciences and Environment(ICWEA)
2161	Outside of the MOO	E.Songliao basin	11GW041	Jinshan	Monzogranite	131.71	47.08	261	3.0	LA-ICP-MS,U-Pb	Zircon	Bi J H et al.	2014	Journal of Earth Sciences and Environment(ICWEA)
2162	Outside of the MOO	E.Songliao basin	13GW578	Zelin	Syenogranite	132.37	47.23	259	3.0	LA-ICP-MS,U-Pb	Zircon	Bi J H et al.	2018	Earth Science Frontiers(ICWEA)
2163	Outside of the MOO	E.Songliao basin	LM1	Luming	Monzogranite	128.68	47.33	183	2.0	LA-ICP-MS,U-Pb	Zircon	Cheng et al.	2015	Acta Petrologica Sinica(ICWEA)
2164	Outside of the MOO	E.Songliao basin	LM2	Luming	Monzogranite	128.68	47.33	187	2.0	LA-ICP-MS,U-Pb	Zircon	Cheng et al.	2015	Acta Petrologica Sinica(ICWEA)
2165	Outside of the MOO	E.Songliao basin	H15-61-5	Yuquan	Monzodiorite	127.23	45.39	179	3.0	LA-ICP-MS,U-Pb	Zircon	Ge M H et al.	2017	Tectonophysics

2166	Outside of the MOO	E.Songliao basin	H15-08-1	Taoshanzhen	Diorite	128.19	46.92	181	1.0	LA-ICP-MS,U-Pb	Zircon	Ge M H et al.	2018	Lithos
2167	Outside of the MOO	E.Songliao basin	H15-11-1	Shenshuzhen	Granodiorite	128.35	46.92	178	2.0	LA-ICP-MS,U-Pb	Zircon	Ge M H et al.	2018	Lithos
2168	Outside of the MOO	E.Songliao basin	H15-14-1	Shenshuzhen	Monzogranite	128.70	46.96	191	1.0	LA-ICP-MS,U-Pb	Zircon	Ge M H et al.	2018	Lithos
2169	Outside of the MOO	E.Songliao basin	H15-16-1	Liangxiangzhen	Monzogranite	129.11	47.05	195	2.0	LA-ICP-MS,U-Pb	Zircon	Ge M H et al.	2018	Lithos
2170	Outside of the MOO	E.Songliao basin	H15-40-1	Dalazi	Granite	129.98	47.38	241	1.0	LA-ICP-MS,U-Pb	Zircon	Ge M H et al.	2018	Lithos
2171	Outside of the MOO	E.Songliao basin	H15-39-1	Dalazi	Diorite	129.98	47.38	246	1.0	LA-ICP-MS,U-Pb	Zircon	Ge M H et al.	2018	Lithos
2172	Outside of the MOO	E.Songliao basin	H15-34-1	Tiexijie	Syenogranite	129.49	47.39	210	1.0	LA-ICP-MS,U-Pb	Zircon	Ge M H et al.	2018	Lithos
2173	Outside of the MOO	E.Songliao basin	H15-35-1	Tiexijie	Monzogranite	129.63	47.39	220	1.0	LA-ICP-MS,U-Pb	Zircon	Ge M H et al.	2018	Lithos
2174	Outside of the MOO	E.Songliao basin	H15-38-1	Dalazi	Syenogranite	129.91	47.40	242	2.0	LA-ICP-MS,U-Pb	Zircon	Ge M H et al.	2018	Lithos
2175	Outside of the MOO	E.Songliao basin	H15-37-1	Wujiazi	Granodiorite	129.79	47.41	251	1.0	LA-ICP-MS,U-Pb	Zircon	Ge M H et al.	2018	Lithos
2176	Outside of the MOO	E.Songliao basin	H15-55-01	Sanzhancun	Syenogranite	128.65	46.20	196	1.0	LA-ICP-MS,U-Pb	Zircon	Ge M H et al.	2019	Lithosphere
2177	Outside of the MOO	E.Songliao basin	H15-56-01	Sanzhancun	Syenogranite	128.54	46.27	196	1.0	LA-ICP-MS,U-Pb	Zircon	Ge M H et al.	2019	Lithosphere
2178	Outside of the MOO	E.Songliao basin	H15-57-01	Sanzhancun	Tonalite	128.53	46.42	186	1.0	LA-ICP-MS,U-Pb	Zircon	Ge M H et al.	2019	Lithosphere
2179	Outside of the MOO	E.Songliao basin	H15-58-01	Sanzhancun	Granodiorite	128.54	46.44	181	1.0	LA-ICP-MS,U-Pb	Zircon	Ge M H et al.	2019	Lithosphere
2180	Outside of the MOO	E.Songliao basin	H15-59-01	Sanzhancun	Monzogranite	128.68	46.56	182	1.0	LA-ICP-MS,U-Pb	Zircon	Ge M H et al.	2019	Lithosphere
2181	Outside of the MOO	E.Songliao basin	H15-10-01	Tieli	Syenogranite	128.19	46.92	190	2.0	LA-ICP-MS,U-Pb	Zircon	Ge M H et al.	2019	Lithosphere
2182	Outside of the MOO	E.Songliao basin	1078-1	Sunhaoxian	Alkali feldspar granite	127.30	48.72	184	2.0	LA-ICP-MS,U-Pb	Zircon	Gou J et al.	2013	Journal of Jilin University（Earth Science Edition）(ICWEA)
2183	Outside of the MOO	E.Songliao basin	1076-1	Sunhaoxian	Monzogranite	127.23	48.73	187	2.0	LA-ICP-MS,U-Pb	Zircon	Gou J et al.	2013	Journal of Jilin University（Earth Science Edition）(ICWEA)
2184	Outside of the MOO	E.Songliao basin	1045-3	Jiayinxian	Alkali feldspar granite	129.86	49.07	210	2.0	LA-ICP-MS,U-Pb	Zircon	Gou J et al.	2013	Journal of Jilin University（Earth Science Edition）(ICWEA)
2185	Outside of the MOO	E.Songliao basin	13HYL3-1	Dadingzi	Syenogranite	129.78	46.05	204	2.0	LA-ICP-MS,U-Pb	Zircon	Guo P et al.	2016	International Geology Review
2186	Outside of the MOO	E.Songliao basin	13HYL9-1	Dadingzi	Syenogranite	129.60	46.93	204	2.0	LA-ICP-MS,U-Pb	Zircon	Guo P et al.	2016	International Geology Review
2187	Outside of the MOO	E.Songliao basin	13HYL8-1	Dadingzi	Syenogranite	129.60	46.93	205	2.0	LA-ICP-MS,U-Pb	Zircon	Guo P et al.	2016	International Geology Review
2188	Outside of the MOO	E.Songliao basin	XLJG-061	Xulaojiugou	Monzogranite	128.47	47.29	180	1.0	LA-ICP-MS,U-Pb	Zircon	Hu et al.	2014	Journal of Asian Earth Sciences
2189	Outside of the MOO	E.Songliao basin	XLJG-011	Xulaojiugou	Monzogranite	128.47	47.29	181	1.0	LA-ICP-MS,U-Pb	Zircon	Hu et al.	2014	Journal of Asian Earth Sciences
2190	Outside of the MOO	E.Songliao basin	LM-0101	Luming	Monzogranite	128.53	47.37	181	2.0	LA-ICP-MS,U-Pb	Zircon	Hu et al.	2014	Journal of Asian Earth Sciences
2191	Outside of the MOO	E.Songliao basin	13HYC35–1	Luobei	Monzogranite	130.70	47.58	262	1.0	LA-ICP-MS,U-Pb	Zircon	Long X Y et al.	2019	Geological Journal
2192	Outside of the MOO	E.Songliao basin	14HT16–1	Yichun	Syenogranite	129.44	48.13	257	1.0	LA-ICP-MS,U-Pb	Zircon	Long X Y et al.	2019	Geological Journal
2193	Outside of the MOO	E.Songliao basin	14HT17–1	Yichun	Monzonite	129.74	48.14	259	1.0	LA-ICP-MS,U-Pb	Zircon	Long X Y et al.	2019	Geological Journal
2194	Outside of the MOO	E.Songliao basin	HTW9–1	Yichun	Alkali feldspar granite	129.68	48.23	271	1.0	LA-ICP-MS,U-Pb	Zircon	Long X Y et al.	2019	Geological Journal
2195	Outside of the MOO	E.Songliao basin	14HT20–1	Yichun	Quartz monzonite	129.99	48.40	258	2.0	LA-ICP-MS,U-Pb	Zircon	Long X Y et al.	2019	Geological Journal
2196	Outside of the MOO	E.Songliao basin	STH-1	Shihecuixi	Monzogranite	128.67	44.87	201	2.0	LA-ICP-MS,U-Pb	Zircon	Qin J F et al.	2016	Lithosphere
2197	Outside of the MOO	E.Songliao basin	9780-2	Qingshuicun	Alkali-feldspar granite	129.78	48.27	222	5.0	LA-ICP-MS,U-Pb	Zircon	Sun D Y et al.	2004	Journal of Jilin University（Earth Science Edition）(ICWEA)
2198	Outside of the MOO	E.Songliao basin	ST1	Shilin	Syenogranite	120.00	40.00	207	3.0	LA-ICP-MS,U-Pb	Zircon	Sun L X et al.	2012	Earth Science Frontiers(ICWEA)
2199	Outside of the MOO	E.Songliao basin	P31-4	Fengmao	Monzogranite	129.61	47.35	211	1.0	LA-ICP-MS,U-Pb	Zircon	Wei H et al.	2012	Earth Science
2200	Outside of the MOO	E.Songliao basin	1002-1	Helinlinchang	Quartz diorite	130.06	47.38	260	1.0	LA-ICP-MS,U-Pb	Zircon	Wei H et al.	2012	Earth Science
2201	Outside of the MOO	E.Songliao basin	P6-3	Sihaolinchang	Monzogranite	130.07	47.37	261	1.0	LA-ICP-MS,U-Pb	Zircon	Wei H et al.	2012	Earth Science
2202	Outside of the MOO	E.Songliao basin	P10-1	Sihaolinchang	Granodiorite	129.99	47.38	234	2.0	LA-ICP-MS,U-Pb	Zircon	Wei H et al.	2012	Earth Science
2203	Outside of the MOO	E.Songliao basin	P10-2	Sihaolinchang	Granodiorite	129.99	47.38	244	2.0	LA-ICP-MS,U-Pb	Zircon	Wei H et al.	2012	Earth Science
2204	Outside of the MOO	E.Songliao basin	1006-1	Dalazi	Monzogranite	129.91	47.40	262	2.0	LA-ICP-MS,U-Pb	Zircon	Wei H et al.	2012	Earth Science
2205	Outside of the MOO	E.Songliao basin	P18-9	Sandaolinchang	Monzogranite	129.82	47.41	260	1.0	LA-ICP-MS,U-Pb	Zircon	Wei H et al.	2012	Earth Science
2206	Outside of the MOO	E.Songliao basin	P24-4	Fengmaolinchang	Granite porphyry	129.68	47.41	210	2.0	LA-ICP-MS,U-Pb	Zircon	Wei H et al.	2012	Earth Science
2207	Outside of the MOO	E.Songliao basin	1007-1	Fenglinlinchang	Monzogranite	129.72	47.42	264	1.0	LA-ICP-MS,U-Pb	Zircon	Wei H et al.	2012	Earth Science
2208	Outside of the MOO	E.Songliao basin	1015-1	Xiaoxilin	Monzogranite	129.30	47.49	212	2.0	LA-ICP-MS,U-Pb	Zircon	Wei H et al.	2012	Earth Science
2209	Outside of the MOO	E.Songliao basin	1009-1	Baiyunlinchang	Monzogranite	129.79	47.58	261	1.0	LA-ICP-MS,U-Pb	Zircon	Wei H et al.	2012	Earth Science
2210	Outside of the MOO	E.Songliao basin	9757-4	Majiatun	Alkali-feldspar granite	129.00	46.06	212	2.0	TIMS,U-Pb	Zircon	Wu Fuyuan et al.	2002	Chemical Geology
2211	Outside of the MOO	E.Songliao basin	9767-2	Milin	Syenogranite	128.83	46.52	197	2.0	TIMS,U-Pb	Zircon	Wu Fuyuan et al.	2002	Chemical Geology
2212	Outside of the MOO	E.Songliao basin	9773-1	Tuanjie	Syenogranite	128.79	46.72	201	3.0	LA-ICP-MS,U-Pb	Zircon	Wu Fuyuan et al.	2011	Journal of Asian Earth Sciences
2213	Outside of the MOO	E.Songliao basin	9766-1	Langxiang	Granodiorite	128.89	46.93	200	2.0	LA-ICP-MS,U-Pb	Zircon	Wu Fuyuan et al.	2011	Journal of Asian Earth Sciences
2214	Outside of the MOO	E.Songliao basin	9777-1	Shichang	Granodiorite	128.30	46.94	175	2.0	LA-ICP-MS,U-Pb	Zircon	Wu Fuyuan et al.	2011	Journal of Asian Earth Sciences
2215	Outside of the MOO	E.Songliao basin	00SW231	Dafeng	Monzogranite	129.41	47.40	201	4.0	LA-ICP-MS,U-Pb	Zircon	Wu Fuyuan et al.	2011	Journal of Asian Earth Sciences
2216	Outside of the MOO	E.Songliao basin	DY0380-1	Xiaoxilin	Granodiorite	129.24	47.46	200	3.0	LA-ICP-MS,U-Pb	Zircon	Wu Fuyuan et al.	2011	Journal of Asian Earth Sciences
2217	Outside of the MOO	E.Songliao basin	P40-1	Taiqing	Monzonite	129.24	47.59	210	2.0	LA-ICP-MS,U-Pb	Zircon	Wu Fuyuan et al.	2011	Journal of Asian Earth Sciences
2218	Outside of the MOO	E.Songliao basin	DY0385-1	Chaoxiantun	Alkali-feldspar granite	129.05	47.65	176	2.0	LA-ICP-MS,U-Pb	Zircon	Wu Fuyuan et al.	2011	Journal of Asian Earth Sciences
2219	Outside of the MOO	E.Songliao basin	00SW225	Hongqi	Granodiorite	128.97	47.70	198	4.0	LA-ICP-MS,U-Pb	Zircon	Wu Fuyuan et al.	2011	Journal of Asian Earth Sciences
2220	Outside of the MOO	E.Songliao basin	HSW6-4	Xingfulinchang	Syenogranite	127.42	48.70	183	2.0	LA-ICP-MS,U-Pb	Zircon	Xu W L et al.	2013	Journal of Asian Earth Sciences
2221	Outside of the MOO	E.Songliao basin	HSW6-12	Xingfulinchang	Syenogranite	127.42	48.70	185	2.0	LA-ICP-MS,U-Pb	Zircon	Xu W L et al.	2013	Journal of Asian Earth Sciences
2222	Outside of the MOO	E.Songliao basin	HSW2-6	Xingfulinchang	Monzogranite	127.26	48.71	176	1.0	LA-ICP-MS,U-Pb	Zircon	Xu W L et al.	2013	Journal of Asian Earth Sciences
2223	Outside of the MOO	E.Songliao basin	HSW2-3	Fengshuigou	Monzogranite	127.34	49.72	175	1.0	LA-ICP-MS,U-Pb	Zircon	Xu W L et al.	2013	Journal of Asian Earth Sciences
2224	Outside of the MOO	E.Songliao basin	10GW240	Liulian	Granodiorite	133.07	47.92	284	2.0	LA-ICP-MS,U-Pb	Zircon	Yu J J et al.	2013	Gondwana Research
2225	Outside of the MOO	E.Songliao basin	11GW240	Liulian	Granodiorite	133.07	47.92	284	2.0	LA-ICP-MS,U-Pb	Zircon	Yu J J et al.	2013	Gondwana Research
2226	Outside of the MOO	E.Songliao basin	GW07005	Zhengdashan	Granodiorite	126.90	49.05	319	3.0	LA-ICP-MS,U-Pb	Zircon	Zhang Y L et al.	2010	Acta Petrologica Sinica(ICWEA)
2227	Outside of the MOO	E.Songliao basin	GW07007	Zhengdashan	Monzogranite	126.90	49.05	316	4.0	LA-ICP-MS,U-Pb	Zircon	Zhang Y L et al.	2010	Acta Petrologica Sinica(ICWEA)
2228	Outside of the MOO	E.Songliao basin	GW07009	Zhengdashan	Monzogranite	126.91	49.10	315	4.0	LA-ICP-MS,U-Pb	Zircon	Zhang Y L et al.	2010	Acta Petrologica Sinica(ICWEA)
2229	Outside of the MOO	E.Songliao basin	15YL12-4	Zhangguangcai Range	Granite	127.35	45.65	184	1.0	LA-ICP-MS,U-Pb	Zircon	Zhu C Y et al.	2017	Lithos
2230	Outside of the MOO	E.Songliao basin	15YL10-1	Zhangguangcai Range	Granodiorite	127.48	45.68	186	1.0	LA-ICP-MS,U-Pb	Zircon	Zhu C Y et al.	2017	Lithos
2231	Outside of the MOO	E.Songliao basin	15YL18-4	Zhangguangcai Range	Granodiorite	128.18	46.92	183	1.0	LA-ICP-MS,U-Pb	Zircon	Zhu C Y et al.	2017	Lithos
2232	Outside of the MOO	E.Songliao basin	15YL28-1	Zhangguangcai Range	Granite	129.53	47.35	186	1.0	LA-ICP-MS,U-Pb	Zircon	Zhu C Y et al.	2017	Lithos
2233	Outside of the MOO	E.Songliao basin	15YL14-1	Zhangguangcai Range	Granodiorite	128.50	47.60	183	1.0	LA-ICP-MS,U-Pb	Zircon	Zhu C Y et al.	2017	Lithos
2234	Outside of the MOO	NE China	12GW029	Tiexi	Diorite	131.46	45.43	296	2.0	LA-ICP-MS,U-Pb	Zircon	Yang H et al.	2015	Gondwana Research
2235	Outside of the MOO	NE China	12GW032	Huafengshan	Monzogranite	131.81	45.58	295	3.0	LA-ICP-MS,U-Pb	Zircon	Yang H et al.	2015	Gondwana Research
2236	Outside of the MOO	NE China	14GW107	Damadang	Granodiorite	130.12	45.42	258	2.0	LA-ICP-MS,U-Pb	Zircon	Yang H et al.	2016	Journal of Asian Earth Sciences
2237	Outside of the MOO	NE China	14GW088	Tuchengzi	Quartz diorite	130.02	45.69	250	2.0	LA-ICP-MS,U-Pb	Zircon	Yang H et al.	2016	Journal of Asian Earth Sciences
2238	Outside of the MOO	NE China	14GW079	Hexing	Syenogranite	130.00	45.72	246	2.0	LA-ICP-MS,U-Pb	Zircon	Yang H et al.	2016	Journal of Asian Earth Sciences
2239	Outside of the MOO	NE China	14GW083	Dongfanghong	Granodiorite	130.00	45.70	253	3.0	LA-ICP-MS,U-Pb	Zircon	Yang H et al.	2016	Journal of Asian Earth Sciences
2240	Outside of the MOO	NE China	14GW074	Shengchan II	Monzogranite	130.13	45.86	258	2.0	LA-ICP-MS,U-Pb	Zircon	Yang H et al.	2016	Journal of Asian Earth Sciences
2241	Outside of the MOO	NE China	14GW068	Jinfeng	Syenogranite	130.26	45.86	254	2.0	LA-ICP-MS,U-Pb	Zircon	Yang H et al.	2016	Journal of Asian Earth Sciences
2242	Outside of the MOO	NE China	14GW073	Shengchan I	Granodiorite	130.15	45.87	249	4.0	LA-ICP-MS,U-Pb	Zircon	Yang H et al.	2016	Journal of Asian Earth Sciences
2243	Outside of the MOO	NE China	14GW067	Fuxing	Granodiorite	130.29	45.87	256	2.0	LA-ICP-MS,U-Pb	Zircon	Yang H et al.	2016	Journal of Asian Earth Sciences

2244	Outside of the MOO	NE China	LMN	Luming	Monzogranite	120.00	40.00	176	2.0	LA-ICP-MS,U-Pb	Zircon	Yang J H et al.	2012	Chemical Geology
2245	Outside of the MOO	NE China	CLN	Cuiling	Quartz diorite	120.00	40.00	178	1.0	LA-ICP-MS,U-Pb	Zircon	Yang J H et al.	2012	Chemical Geology
2246	Outside of the MOO	NE China	HJHN1	Huojihe	Monzogranite	120.00	40.00	186	2.0	LA-ICP-MS,U-Pb	Zircon	Yang J H et al.	2012	Chemical Geology
2247	Outside of the MOO	NE China	11HNA4-1	Xiaobeihu	Monzogranite	128.72	44.10	255	2.0	LA-ICP-MS,U-Pb	Zircon	Yu J J et al.	2013	Gondwana Research
2248	Outside of the MOO	NE China	11HNA11-1	Weicaohe	Granodiorite	129.44	44.38	252	2.0	LA-ICP-MS,U-Pb	Zircon	Yu J J et al.	2013	Gondwana Research
2249	Outside of the MOO	NE China	11HNA1-1	Huangqigou	Quartz diorite	129.44	44.38	256	1.0	LA-ICP-MS,U-Pb	Zircon	Yu J J et al.	2013	Gondwana Research
2250	Outside of the MOO	NE China	12YQ01	Yongqing	Granodiorite	131.07	46.32	265	1.0	LA-ICP-MS,U-Pb	Zircon	Yu J J et al.	2013	Gondwana Research
2251	Outside of the MOO	NE China	16GW126	Mudanjiang	Granodiorite	129.38	44.64	219	1.0	LA-ICP-MS,U-Pb	Zircon	Zhao D et al.	2018	Lithos
2252	Outside of the MOO	NE China	16GW124	Mudanjiang	Diorite	129.38	44.64	221	1.0	LA-ICP-MS,U-Pb	Zircon	Zhao D et al.	2018	Lithos
2253	Outside of the MOO	NE China	16GW121	Mudanjiang	Monzogranite	129.38	44.66	217	1.0	LA-ICP-MS,U-Pb	Zircon	Zhao D et al.	2018	Lithos
2254	Outside of the MOO	NE China	16GW114	Mudanjiang	Granodiorite	129.57	44.67	215	1.0	LA-ICP-MS,U-Pb	Zircon	Zhao D et al.	2018	Lithos
2255	Outside of the MOO	NE China	16GW110	Mudanjiang	Diorite	129.57	44.68	221	1.0	LA-ICP-MS,U-Pb	Zircon	Zhao D et al.	2018	Lithos
2256	Outside of the MOO	NE China	15YL40-1	Zhangguangcai Range	Granite	126.72	43.82	191	1.0	LA-ICP-MS,U-Pb	Zircon	Zhu C Y et al.	2017	Lithos
2257	Outside of the MOO	NE China	15YL36-5	Zhangguangcai Range	Monzogranite	126.92	43.88	191	1.0	LA-ICP-MS,U-Pb	Zircon	Zhu C Y et al.	2017	Lithos
2258	Outside of the MOO	NE China	15YL38-1	Zhangguangcai Range	Granodiorite	127.02	44.35	163	1.0	LA-ICP-MS,U-Pb	Zircon	Zhu C Y et al.	2017	Lithos
2259	Outside of the MOO	S.Mongolia	AJW-03-116	Oyu Tolgoi porphyry Cu-Au di	320 intrusions	106.86	43.06	320		SHRIMP,U-Pb	Zircon	Alan J. Wainwright et	2011	Geological Society of America Bulletin
2260	Outside of the MOO	S.Mongolia	AJW-03-055	Oyu Tolgoi porphyry Cu-Au di	LVS andesite	106.86	43.06	339	2.0	SHRIMP,U-Pb	Zircon	Alan J. Wainwright et	2011	Geological Society of America Bulletin
2261	Outside of the MOO	S.Mongolia	AJW-03-062	Oyu Tolgoi porphyry Cu-Au di	Rhyolite	106.86	43.06	340	3.0	SHRIMP,U-Pb	Zircon	Alan J. Wainwright et	2011	Geological Society of America Bulletin
2262	Outside of the MOO	S.Mongolia	AJW-03-082	Oyu Tolgoi porphyry Cu-Au di	HbBt Andesite	106.86	43.06	345	3.0	SHRIMP,U-Pb	Zircon	Alan J. Wainwright et	2011	Geological Society of America Bulletin
2263	Outside of the MOO	S.Mongolia	AJW-06-485	Oyu Tolgoi porphyry Cu-Au di	OTHS	106.86	43.06	346		SHRIMP,U-Pb	Zircon	Alan J. Wainwright et	2011	Geological Society of America Bulletin
2264	Outside of the MOO	S.Mongolia	AJW-03-205	Oyu Tolgoi porphyry Cu-Au di	LVS dacite	106.86	43.06	347	2.0	SHRIMP,U-Pb	Zircon	Alan J. Wainwright et	2011	Geological Society of America Bulletin
2265	Outside of the MOO	S.Mongolia	AJW-04-371	Oyu Tolgoi porphyry Cu-Au di	UVS	106.86	43.06	347	2.0	SHRIMP,U-Pb	Zircon	Alan J. Wainwright et	2011	Geological Society of America Bulletin
2266	Outside of the MOO	S.Mongolia	AJW-03-074	Oyu Tolgoi porphyry Cu-Au di	350 intrusions	106.86	43.06	350	4.0	SHRIMP,U-Pb	Zircon	Alan J. Wainwright et	2011	Geological Society of America Bulletin
2267	Outside of the MOO	S.Mongolia	M127	Nemegt Uul	Dacite dyke	101.53	43.46	319	3.0	SHRIMP,U-Pb	Zircon	Demoux A. et al.	2009	
2268	Outside of the MOO	S.Mongolia	EGD001(75)	Oyu Tologoi	Granodiorite	106.84	43.05	348	4.0	SHRIMP,U-Pb	Zircon	Dolgopolova A. et al.	2013	Lithos
2269	Outside of the MOO	S.Mongolia	M14913-5.1			108.58	42.96	254	2.0	LA-ICP-MS,U-Pb	Zircon	This study		
2270	Outside of the MOO	S.Mongolia	M14913-2.1			108.59	42.97	249	2.0	LA-ICP-MS,U-Pb	Zircon	This study		
2271	Outside of the MOO	S.Mongolia	M14913-4.1			108.59	42.97	258	3.0	LA-ICP-MS,U-Pb	Zircon	This study		
2272	Outside of the MOO	S.Mongolia	M14913-1.1			108.60	42.97	258	1.0	LA-ICP-MS,U-Pb	Zircon	This study		
2273	Outside of the MOO	S.Mongolia	M14914-6.1			108.40	43.14	301	3.0	LA-ICP-MS,U-Pb	Zircon	This study		
2274	Outside of the MOO	S.Mongolia	M14914-11.1			107.20	43.18	307	2.0	LA-ICP-MS,U-Pb	Zircon	This study		
2275	Outside of the MOO	S.Mongolia	M14915-2.1			107.14	43.19	292	3.0	LA-ICP-MS,U-Pb	Zircon	This study		
2276	Outside of the MOO	S.Mongolia	M14915-1.1			107.14	43.19	300	4.0	LA-ICP-MS,U-Pb	Zircon	This study		
2277	Outside of the MOO	Selemdzha Terrane	V-78-3	Zlatoustovsk Complex	Granite	133.51	52.74	241	11.0	LA-ICP-MS,U-Pb	Zircon	Sorokin, Andrey A. et	2021	Lihthos
2278	Outside of the MOO	Selemdzha Terrane	V-93	Zlatoustovsk Complex	Granodiorite	132.63	52.89	253	2.0	LA-ICP-MS,U-Pb	Zircon	Sorokin, Andrey A. et	2021	Lihthos
2279	Outside of the MOO	Selemdzha Terrane	C-821	Zlatoustovsk Complex	Granodiorite	132.17	53.03	251	2.0	LA-ICP-MS,U-Pb	Zircon	Sorokin, Andrey A. et	2021	Lihthos
2280	Outside of the MOO	SW Mongolia	DZB 1/1	Zuun Bogd Massif.	Granitoid	101.98	43.17	279	1.0	TIMS,U-Pb	Zircon	Yarmolyuk V V, et al	2008	Doklady Earth Sciences
2281	Outside of the MOO	Tianshan	AX12	Tulasu And Its Surrounding Ar	Andesite	81.61	44.24	356	2.0	SHRIMP,U-Pb	Zircon	An Fang et al.	2016	Gondwana Research
2282	Outside of the MOO	Tianshan	15T56A		Rhyolite	81.91	43.44	316	3.0	LA-ICP-MS,U-Pb	Zircon	Cao YC et al.	2017	Lithos
2283	Outside of the MOO	Tianshan	15T94		Rhyolite	81.12	43.50	336	3.0	LA-ICP-MS,U-Pb	Zircon	Cao YC et al.	2017	Lithos
2284	Outside of the MOO	Tianshan	1642		Biotite monzonitic granite	81.33	45.19	299	6.0	SHRIMP,U-Pb	Zircon	Chen BH et al.	2008	Acta Petrologica Sinica (ICWEA)
2285	Outside of the MOO	Tianshan	1624-2	Yukeke	rhyolite	81.08	45.17	300	5.0	SHRIMP,U-Pb	Zircon	Chen bihe et al.	2007	Acta Petrologica Sinica(ICWEA)
2286	Outside of the MOO	Tianshan	1702	Wulasitai	Biotite granodiorite	81.13	45.22	294	4.0	SHRIMP,U-Pb	Zircon	Chen bihe et al.	2007	Acta Petrologica Sinica(ICWEA)
2287	Outside of the MOO	Tianshan	1642-1	Xiadongaola	biotiteMonzonite granite	81.20	45.22	299	6.0	SHRIMP,U-Pb	Zircon	Chen bihe et al.	2007	Acta Petrologica Sinica(ICWEA)
2288	Outside of the MOO	Tianshan	SSY	Sangshuyuanziyanti	Monzonite granite	88.86	42.17	296	4.0	LA-ICP-MS,U-Pb	Zircon	Chen Chao et al.	2013	Journal of Earth Science (ICWEA)
2289	Outside of the MOO	Tianshan	A1	zhongbao	granodiorite	88.71	42.19	293	3.0	LA-ICP-MS,U-Pb	Zircon	Chen Chao et al.	2013	Journal of Earth Science (ICWEA)
2290	Outside of the MOO	Tianshan	YD01	yandong	Monzonite granite	92.82	42.11	333	4.0	SHRIMP,U-Pb	Zircon	Chen Fuwen et al.	2005	Acta Geologica Sinica (ICWEA)
2291	Outside of the MOO	Tianshan	TC48	Tuwu	granite porphyry	92.69	42.15	334	3.0	SHRIMP,U-Pb	Zircon	Chen Fuwen et al.	2005	Acta Geologica Sinica (ICWEA)
2292	Outside of the MOO	Tianshan	TGLS1		Granite dyke	80.78	42.40	285	2.0	CAMECA,U-Pb	Zircon	Gao J et al.	2011	Tectonphysics
2293	Outside of the MOO	Tianshan	KKS5	Kekesuhe	Monzonite granite	81.91	42.76	281	8.7	LA-ICP-MS,U-Pb	Zircon	Gao Jun et al.	2009	Earth Science(ICWEA)
2294	Outside of the MOO	Tianshan	KKS12	Kekesuhe	granite	81.90	42.82	352	5.0	LA-ICP-MS,U-Pb	Zircon	Gao Jun et al.	2009	Earth Science(ICWEA)
2295	Outside of the MOO	Tianshan	KKS1	Kekesuhe	syenite	81.90	42.87	349	6.0	LA-ICP-MS,U-Pb	Zircon	Gao Jun et al.	2009	Earth Science(ICWEA)
2296	Outside of the MOO	Tianshan	W8037	Changawuzi	Gneissic porphyritic tonalite	80.84	42.47	326	3.0	SHRIMP,U-Pb	Zircon	Gou longlong et al.	2012	Lithos
2297	Outside of the MOO	Tianshan	W8026	Alasan pluton	P-quartz monzonite	80.84	42.46	333	3.0	LA-ICP-MS,U-Pb	Zircon	Gou longlong et al.	2012	Lithos
2298	Outside of the MOO	Tianshan	W8028	Changawuzi	Gneissic granodiorite	80.78	42.52	293	3.0	SHRIMP,U-Pb	Zircon	Gou longlong et al.	2012	Lithos
2299	Outside of the MOO	Tianshan	W8027	Alasan pluton	Granodiorite	80.77	42.52	294	2.2	ICP-MS,U-Pb	Zircon	Gou longlong et al.	2012	Lithos
2300	Outside of the MOO	Tianshan	无	central tianshan	leucogranite	90.28	41.82	354	16.0	SHRIMP,U-Pb	Zircon	Guo Zhaojie et al.	2007	Acta Petrologica Sinica (ICWEA)
2301	Outside of the MOO	Tianshan	W8026	Yamansubei	Quartz monzonite	84.39	44.10	319	2.9	SHRIMP,U-Pb	Zircon	Han B et al.	2010	Xinjiang Geology (ICWEA)
2302	Outside of the MOO	Tianshan	DK-6		Granodiorite	84.39	44.10	316	3.0	SHRIMP,U-Pb	Zircon	Han BF et al.	2010	Geological Society of America Bulletin
2303	Outside of the MOO	Tianshan	02KK101	Huangshandong	norite	89.68	46.76	287	5.0	SHRIMP,U-Pb	Zircon	Hanbaofu et al.	2003	Chinese Science Bulletin (ICWEA)
2304	Outside of the MOO	Tianshan	02HSD-02	Huangshandong	biotitehypersthenfels	94.73	42.27	274	3.0	SHRIMP,U-Pb	Zircon	Hanbaofu et al.	2004	Chinese Science Bulletin (ICWEA)
2305	Outside of the MOO	Tianshan	06XJ-74	（Middle Part）Yangchang	Monzonite granite	82.62	43.61	317	3.0	LA-ICP-MS,U-Pb	Zircon	Hu Jun et al	2016	Acta Petrologica Sinica (ICWEA)
2306	Outside of the MOO	Tianshan	020XW-11a		granite	92.52	41.74	301	4.6	SHRIMP,U-Pb	Zircon	Hu Yuanqing et al.	2009	Geological Science and Technology Information(ICWEA)
2307	Outside of the MOO	Tianshan	Q02		Porphyritic two-mica granite	80.90	41.91	291	3.0	LA-ICP-MS,U-Pb	Zircon	Huang He et al.	2011	Acta Geologica Sinica(ICWEA)
2308	Outside of the MOO	Tianshan	Qab-03	yingmailai	dark inclusion	80.81	41.98	285	3.7	LA-ICP-MS,U-Pb	Zircon	Huang He et al.	2011	Acta Geologica Sinica(ICWEA)
2309	Outside of the MOO	Tianshan	DYCML3-01		Monzonite-Bt monzonite-Bt adame	76.87	40.92	286	3.0	LA-ICP-MS,U-Pb	Zircon	Huang He et al.	2012	Lithos
2310	Outside of the MOO	Tianshan	BLG13-01		Bt monzonite	77.24	40.87	291	3.0	LA-ICP-MS,U-Pb	Zircon	Huang He et al.	2015	Acta Petrologica Et Mineralogica(ICWEA).
2311	Outside of the MOO	Tianshan	TLK-01		Altered felsic lava flow	81.78	42.08	285	2.0	LA-ICP-MS,U-Pb	Zircon	Huang He et al.	2015	Lithos
2312	Outside of the MOO	Tianshan	WLSTII-3		Granite	86.56	42.90	359	4.0	LA-ICP-MS,U-Pb	Zircon	Huang He et al.	2015	Acta Geologica Sinica(ICWEA)
2313	Outside of the MOO	Tianshan	WLSTIII-6		K-feldspar granite	86.64	42.90	312	4.0	LA-ICP-MS,U-Pb	Zircon	Huang He et al.	2015	Acta Geologica Sinica(ICWEA)
2314	Outside of the MOO	Tianshan	BLTIV-9		Granodiorite	86.12	42.91	306	5.0	LA-ICP-MS,U-Pb	Zircon	Huang He et al.	2015	Acta Geologica Sinica(ICWEA)
2315	Outside of the MOO	Tianshan	LHT14-2	Laohutai	volcanic breccia	83.31	43.63	295	3.2	LA-ICP-MS,U-Pb	Zircon	Huang He et al.	2015	Lithos
2316	Outside of the MOO	Tianshan	TKSB14-1	Tekesinan	biotitegranite	81.90	42.87	353	2.0	LA-ICP-MS,U-Pb	Zircon	Huang He et al.	2020	Earth-Science Reviews
2317	Outside of the MOO	Tianshan	TKSC14-8	Tekesi（Southern Part ）	monzonitic granite	81.90	42.87	353	2.0	LA-ICP-MS,U-Pb	Zircon	Huang He et al.	2020	Earth-Science Reviews
2318	Outside of the MOO	Tianshan	TKSD14-1	Tekesi（Southern Part ）	granite	81.90	42.90	341	2.0	LA-ICP-MS,U-Pb	Zircon	Huang He et al.	2020	Earth-Science Reviews
2319	Outside of the MOO	Tianshan	TKSE14-14	Tekesidong	granite	81.99	43.25	304	5.6	LA-ICP-MS,U-Pb	Zircon	Huang He et al.	2020	Earth-Science Reviews
2320	Outside of the MOO	Tianshan	XYD14-6	Xinyuanbei	granodiorite	83.25	43.35	323	2.9	LA-ICP-MS,U-Pb	Zircon	Huang He et al.	2020	Earth-Science Reviews
2321	Outside of the MOO	Tianshan	TKSF14-10	Tekesibei	granite	81.87	43.39	251	6.3	LA-ICP-MS,U-Pb	Zircon	Huang He et al.	2020	Earth-Science Reviews

2322	Outside of the MOO	Tianshan	TKSG14-6	Tekesi（North）	granite	81.87	43.39	251	6.3	LA-ICP-MS,U-Pb	Zircon	Huang He et al.	2020	Earth-Science Reviews
2323	Outside of the MOO	Tianshan	TKSE14-15	Tekesidong	gabbro	81.69	43.43	334	3.5	LA-ICP-MS,U-Pb	Zircon	Huang He et al.	2020	Earth-Science Reviews
2324	Outside of the MOO	Tianshan	XFG14-6	Xinyuan（North）	diorite	83.48	43.53	317	2.9	LA-ICP-MS,U-Pb	Zircon	Huang He et al.	2020	Earth-Science Reviews
2325	Outside of the MOO	Tianshan	XFG14-7	Xinyuanbeipiandong	diorite	83.48	43.53	308	2.7	LA-ICP-MS,U-Pb	Zircon	Huang He et al.	2020	Earth-Science Reviews
2326	Outside of the MOO	Tianshan	XYE14-8	Xinyuannan	diorite	83.48	43.53	317	2.9	LA-ICP-MS,U-Pb	Zircon	Huang He et al.	2020	Earth-Science Reviews
2327	Outside of the MOO	Tianshan	XYC14-7	Xinyuanbei	granite	83.33	43.57	315	3.0	LA-ICP-MS,U-Pb	Zircon	Huang He et al.	2020	Earth-Science Reviews
2328	Outside of the MOO	Tianshan	XYB14-4	Xinyuanbei	granodiorite	83.31	43.62	297	5.5	LA-ICP-MS,U-Pb	Zircon	Huang He et al.	2020	Earth-Science Reviews
2329	Outside of the MOO	Tianshan	XYA14-7	Xinyuanbei	granite porphyry	83.31	43.62	314	1.9	LA-ICP-MS,U-Pb	Zircon	Huang He et al.	2020	Earth-Science Reviews
2330	Outside of the MOO	Tianshan	XYB14-7	Xinyuanbei	diorite	83.32	43.61	312	3.5	LA-ICP-MS,U-Pb	Zircon	Huang He et al.	2020	Earth-Science Reviews
2331	Outside of the MOO	Tianshan	NLKB14-10	Nileke（Southern Part ）	granite	82.48	43.89	296	3.4	LA-ICP-MS,U-Pb	Zircon	Huang He et al.	2020	Earth-Science Reviews
2332	Outside of the MOO	Tianshan	YNB14-5	Yining（The Eastern Part ）（	monzonitic granite	82.07	44.07	250	3.6	LA-ICP-MS,U-Pb	Zircon	Huang He et al.	2020	Earth-Science Reviews
2333	Outside of the MOO	Tianshan	YNA14-5	Yining（The Eastern Part ）（	granite	82.10	44.09	305	3.1	LA-ICP-MS,U-Pb	Zircon	Huang He et al.	2020	Earth-Science Reviews
2334	Outside of the MOO	Tianshan	R12T16		Granitoids	77.21	40.36	229	2.5	LA-ICP-MS,U-Pb	Zircon	Huang He.	2013	Doctoral thesis: China University of Geosciences
2335	Outside of the MOO	Tianshan	CWL-03	Chuanwulu	granite	92.68	42.96	353	3.2	SHRIMP,U-Pb	Zircon	Izokh et al.	2011	Russian Geology and Geophysics
2336	Outside of the MOO	Tianshan	280001		Coarse-grained granite	76.33	40.79	281	2.0	CAMECA,U-Pb	Zircon	Konopelko et al.	2007	Lithos
2337	Outside of the MOO	Tianshan	280701		Ovoidal granite	76.42	40.80	279	8.0	CAMECA,U-Pb	Zircon	Konopelko et al.	2007	Lithos
2338	Outside of the MOO	Tianshan	209202		Rapakivi granite	79.08	41.66	297	4.0	CAMECA,U-Pb	Zircon	Konopelko et al.	2007	Lithos
2339	Outside of the MOO	Tianshan	206801		Coarse-grained granite	79.08	41.82	279	8.0	CAMECA,U-Pb	Zircon	Konopelko et al.	2007	Lithos
2340	Outside of the MOO	Tianshan	206801		Quartz porphyry	79.08	41.82	279	8.0	CAMECA,U-Pb	Zircon	Konopelko et al.	2007	Lithos
2341	Outside of the MOO	Tianshan	215701		Rapakivi granite	79.08	41.82	292	3.0	SHRIMP,U-Pb	Zircon	Konopelko et al.	2007	Lithos
2342	Outside of the MOO	Tianshan	416506		Granite	79.12	42.00	299	4.0	SHRIMP,U-Pb	Zircon	Konopelko et al.	2009	Ore Geology Reviews
2343	Outside of the MOO	Tianshan	416801		Leucogranite	79.13	42.03	295	4.0	SHRIMP,U-Pb	Zircon	Konopelko et al.	2009	Ore Geology Reviews
2344	Outside of the MOO	Tianshan	416000		Granite	79.09	42.06	291	5.0	SHRIMP,U-Pb	Zircon	Konopelko et al.	2009	Ore Geology Reviews
2345	Outside of the MOO	Tianshan	416803		Leucogranite	79.28	42.10	289	6.0	SHRIMP,U-Pb	Zircon	Konopelko et al.	2009	Ore Geology Reviews
2346	Outside of the MOO	Tianshan	LG2-1	Lailisigaodongyanti	granodiorite-porphyry	93.88	42.03	228	0.5	LA-ICP-MS,U-Pb	Zircon	Lei Ruxiong et al.	2013	Acta Petrologica Sinica (ICWEA)
2347	Outside of the MOO	Tianshan	X-640	Yamansubei	moyite	84.70	44.05	343	2.7	SIMS,U-Pb	Zircon	Li Chao et al.	2013	Chinese Journal of Geology (ICWEA)
2348	Outside of the MOO	Tianshan	SK05	Kuitunheshelvyan	plagiogranite	94.69	42.35	278	4.0	SHRIMP,U-Pb	Zircon	Li Huaqin et al.	2004	Acta Geoscientica Sinica (ICWEA)
2349	Outside of the MOO	Tianshan	AKTL2	Sanchakoutongkuangqu	granite porphyry	81.31	43.02	340	2.3	LA-ICP-MS,U-Pb	Zircon	Li Jilei et al.	2010	Acta Petrologica Sinica (ICWEA)
2350	Outside of the MOO	Tianshan	AKTL9	Adengtaodiqu	granite porphyry	81.32	43.03	354	2.3	LA-ICP-MS,U-Pb	Zircon	Li Jilei et al.	2010	Acta Petrologica Sinica (ICWEA)
2351	Outside of the MOO	Tianshan	YTS7-01		Tonalite porphyry	82.48	43.73	269	3.0	LA-ICP-MS,U-Pb	Zircon	Li NB et al.	2013	Acta Petrologica Sinica(ICWEA)
2352	Outside of the MOO	Tianshan	10QKS01	Qiongkushitai	granodiorite	81.91	42.79	355	2.0	LA-ICP-MS,U-Pb	Zircon	Li Ping et al.	2011	Xi'an: Chang'an University
2353	Outside of the MOO	Tianshan	10CK25	Baluntainan	diorite	82.24	42.87	337	1.1	LA-ICP-MS,U-Pb	Zircon	Li Ping et al.	2011	Xi'an: Chang'an University
2354	Outside of the MOO	Tianshan	10NLT02	Nalatishandongdongkong	diorite	86.58	42.87	353	2.4	LA-ICP-MS,U-Pb	Zircon	Li Ping et al.	2011	Xi'an: Chang'an University
2355	Outside of the MOO	Tianshan	08XYO2	Qiabuhebei	diorite	82.22	42.89	314	2.5	LA-ICP-MS,U-Pb	Zircon	Li Ping et al.	2011	Xi'an: Chang'an University
2356	Outside of the MOO	Tianshan	08KKS3	Kekesuhe	diorite	83.48	43.13	319	2.6	LA-ICP-MS,U-Pb	Zircon	Li Ping et al.	2011	Xi'an: Chang'an University
2357	Outside of the MOO	Tianshan	07-1a	Wenquan	diorite	84.98	43.17	340	1.1	LA-ICP-MS,U-Pb	Zircon	Li Ping et al.	2011	Xi'an: Chang'an University
2358	Outside of the MOO	Tianshan	10QKS02	Qiongkushitai	granodiorite	91.55	42.12	272	1.6	SHRIMP,U-Pb	Zircon	Li Shaozhen et al.	2006	Geological Bulletin of China(ICWEA)
2359	Outside of the MOO	Tianshan	A307	Kezidongtage	granodiorite	92.82	42.16	301	13.0	SHRIMP,U-Pb	Zircon	Li Wenming et al.	2002	Northwestern Geology (ICWEA)
2360	Outside of the MOO	Tianshan	BS-09	Tuwu	granite porphyry	93.11	42.47	313	8.0	SHRIMP,U-Pb	Zircon	Li Wenming et al.	2002	Northwestern Geology (ICWEA)
2361	Outside of the MOO	Tianshan	G425	Dananhu	Monzonite granite	91.76	42.17	316	4.0	SHRIMP,U-Pb	Zircon	Li Wenqian et al.	2006	Acta Geologica Sinica (ICWEA)
2362	Outside of the MOO	Tianshan	11CG02	Nuodongxibeihuagangyan	moyite	82.27	43.68	292	3.7	LA-ICP-MS,U-Pb	Zircon	Li Xiaoying et al.	2012	Xi'an: Chang'an University
2363	Outside of the MOO	Tianshan	C425	Cai（Middle Part）	biotiteMonzonite granite	84.88	43.31	343	3.7	LA-ICP-MS,U-Pb	Zircon	Li Xiaoying et al.	2013	Xi'an: Chang'an University
2364	Outside of the MOO	Tianshan	11CG01	Chagangnuodongyanti	granodiorite	83.33	43.57	324	3.3	LA-ICP-MS,U-Pb	Zircon	Li Xiaoying et al.	2013	Xi'an: Chang'an University
2365	Outside of the MOO	Tianshan	11ZK01	Zeketaiyanti	Monzonite granite	82.66	43.67	304	4.0	LA-ICP-MS,U-Pb	Zircon	Li Xiaoying et al.	2013	Xi'an: Chang'an University
2366	Outside of the MOO	Tianshan	11WL01-b	Wulangdabanyanti	granite porphyry	84.92	43.92	304	5.6	LA-ICP-MS,U-Pb	Zircon	Li Xiaoying et al.	2013	Xi'an: Chang'an University
2367	Outside of the MOO	Tianshan		Saikendonglu	diorite	84.04	43.52	281	9.0	LA-ICP-MS,U-Pb	Zircon	Li Yongjun et al.	2007	Acta Petrologica Sinica (ICWEA)
2368	Outside of the MOO	Tianshan	11KL01-a	Yilanbasitaoyanti	granodiorite-porphyry	83.99	43.56	331	6.0	LA-ICP-MS,U-Pb	Zircon	Li Yongjun et al.	2007	Acta Petrologica Sinica (ICWEA)
2369	Outside of the MOO	Tianshan	NL07-16	muhanbasitao	granite	82.44	43.53	319	2.4	LA-ICP-MS,U-Pb	Zircon	Liu Xin et al.	2012	Acta Petrologica Sinica (ICWEA)
2370	Outside of the MOO	Tianshan	KWSY16	alataoshan	rhyolite	82.38	44.30	271	6.5	LA-ICP-MS,U-Pb	Zircon	Liu zhiqiang et al.	2005	Acta Petrologica Sinica(ICWEA)
2371	Outside of the MOO	Tianshan	KWSY26	alataoshan	granite	82.42	44.95	292	4.9	LA-ICP-MS,U-Pb	Zircon	Liu Zhiqiang et al.	2005	Acta Petrologica Sinica (ICWEA)
2372	Outside of the MOO	Tianshan	KWSY7	alataoshan	granite	82.40	45.10	298	5.7	LA-ICP-MS,U-Pb	Zircon	Liu Zhiqiang et al.	2005	Acta Petrologica Sinica (ICWEA)
2373	Outside of the MOO	Tianshan	XJ050	Wenquan	dacite	82.45	42.36	285	4.0	SHRIMP,U-Pb	Zircon	Long LL et al	2011	Lithos
2374	Outside of the MOO	Tianshan	DK13	Dongkugonglu	biotitegranite	80.80	42.52	247	0.9	LA-ICP-MS,U-Pb	Zircon	Long LL et al	2011	Lithos
2375	Outside of the MOO	Tianshan	DK14	Dongkugonglu	biotitegranodiorite	81.30	42.60	297	0.8	LA-ICP-MS,U-Pb	Zircon	Long LL et al	2011	Lithos
2376	Outside of the MOO	Tianshan	WQ2	Wenquan	biotiteMonzonite granite	82.21	42.88	322	5.0	LA-ICP-MS,U-Pb	Zircon	Long LL et al	2011	Lithos
2377	Outside of the MOO	Tianshan	QK5	Qiongkushitaihe	biotitegranite	82.21	42.88	325	5.0	LA-ICP-MS,U-Pb	Zircon	Long LL et al	2011	Lithos
2378	Outside of the MOO	Tianshan	DK28	Du–Ku High Road	Granitic gneiss	84.33	43.17	321	1.1	LA-ICP-MS,U-Pb	Zircon	Long LL et al	2011	Lithos
2379	Outside of the MOO	Tianshan	KKS10		Biotite granite	81.88	42.83	352	5.0	SHRIMP,U-Pb	Zircon	Long LL et al.	2011	Lithos
2380	Outside of the MOO	Tianshan	DK24		Granitic mylonite	84.25	43.16	342	1.0	LA-ICP-MS,U-Pb	Zircon	Long LL et al.	2011	Lithos
2381	Outside of the MOO	Tianshan	BK13	Bikaiyanti	granite	79.69	41.70	289	5.5	LA-ICP-MS,U-Pb	Zircon	Luo Jinhai et al.	2008	Acta Petrologica Sinica (ICWEA)
2382	Outside of the MOO	Tianshan	20070K16-62	Hamibaiganhu	alkali-feldspar granite	80.86	41.85	258	3.0	LA-ICP-MS,U-Pb	Zircon	Ma LeTian et al.	2010	Earth Science (ICWEA)
2383	Outside of the MOO	Tianshan	762	Baluntai	moyite	87.89	42.23	272	2.2	LA-ICP-MS,U-Pb	Zircon	Ma X X et al.	2014	Gondwana Research
2384	Outside of the MOO	Tianshan	776	Baluntai	moyite	87.87	42.23	275	2.2	LA-ICP-MS,U-Pb	Zircon	Ma X X et al.	2014	Gondwana Research
2385	Outside of the MOO	Tianshan	766	Baluntai	alkali-feldspar granite	88.66	42.45	352	2.9	LA-ICP-MS,U-Pb	Zircon	Ma X X et al.	2014	Gondwana Research
2386	Outside of the MOO	Tianshan	777	Baluntai	biotitemoyite	86.22	42.45	292	2.3	LA-ICP-MS,U-Pb	Zircon	Ma X X et al.	2014	Gondwana Research
2387	Outside of the MOO	Tianshan	787	Baluntai	biotitemoyite	86.27	42.68	300	3.1	LA-ICP-MS,U-Pb	Zircon	Ma X X et al.	2014	Gondwana Research
2388	Outside of the MOO	Tianshan	797-1	Baluntai	granite	87.20	42.82	352	2.8	LA-ICP-MS,U-Pb	Zircon	Ma X X et al.	2014	Gondwana Research
2389	Outside of the MOO	Tianshan	794	Baluntai	biotitemoyite	87.10	42.82	346	2.7	LA-ICP-MS,U-Pb	Zircon	Ma X X et al.	2014	Gondwana Research
2390	Outside of the MOO	Tianshan	638	Baluntai	granodiorite	87.65	42.84	267	3.6	LA-ICP-MS,U-Pb	Zircon	Ma X X et al.	2014	Gondwana Research
2391	Outside of the MOO	Tianshan	792	Baluntai	moyite	87.46	42.85	337	2.9	LA-ICP-MS,U-Pb	Zircon	Ma X X et al.	2014	Gondwana Research
2392	Outside of the MOO	Tianshan	788	Baluntai	biotitemoyite	86.56	42.88	263	2.0	LA-ICP-MS,U-Pb	Zircon	Ma X X et al.	2014	Gondwana Research
2393	Outside of the MOO	Tianshan	791	Baluntai	moyite	86.90	42.92	339	3.4	LA-ICP-MS,U-Pb	Zircon	Ma X X et al.	2014	Gondwana Research
2394	Outside of the MOO	Tianshan	08KC11	ahongkailide	Monzonite granite	86.48	41.72	341	5.7	LA-ICP-MS,U-Pb	Zircon	Ma Xuxuan et al.	2013	Geological Bulletin of China (ICWEA)
2395	Outside of the MOO	Tianshan	798		Granodiorite	87.18	42.82	352	3.0	LA-ICP-MS,U-Pb	Zircon	Ma Xuxuan et al.	2014	Gondwana Research.
2396	Outside of the MOO	Tianshan	763		K-feldspar granite	87.46	42.85	337	3.0	LA-ICP-MS,U-Pb	Zircon	Ma Xuxuan et al.	2014	Gondwana Research.
2397	Outside of the MOO	Tianshan	KECII-11		Biotite granite	85.57	41.96	311	4.0	LA-ICP-MS,U-Pb	Zircon	Ma Xuxuan et al.	2017	Phd dissertation
2398	Outside of the MOO	Tianshan	QGX-8		Biotite monzogranite	86.21	42.02	302	3.0	LA-ICP-MS,U-Pb	Zircon	Ma Xuxuan et al.	2017	Phd dissertation
2399	Outside of the MOO	Tianshan	D201-1	Dananhu	Monzonite granite	91.63	42.31	357	6.2	SHRIMP,U-Pb	Zircon	Ma XX et al.	2002	Xinjiang Geology(ICWEA)

2400	Outside of the MOO	Tianshan	T2	kailidenanhuagangyan	Monzonite granite	91.90	42.13	268	1.6	SHRIMP,U-Pb	Zircon	Ma XX et al.	2006	Geological Bulletin of China(ICWEA)
2401	Outside of the MOO	Tianshan	C13TKS18		Red rhyolite	81.93	43.43	325	8.0	LA-ICP-MS,U-Pb	Zircon	Ni Jiati·Abudongxun	2018	Lithos
2402	Outside of the MOO	Tianshan	C15ZS07		Rhyolite	81.30	43.02	355	3.0	LA-ICP-MS,U-Pb	Zircon	Qin Q et al.	2018	Lithos
2403	Outside of the MOO	Tianshan	C13TKS02		Gray rhyolite	81.88	43.36	351	5.0	LA-ICP-MS,U-Pb	Zircon	Qin Q et al.	2018	Lithos
2404	Outside of the MOO	Tianshan	D09025	Aqikekudongkenan	granodiorite	91.75	41.69	318	5.0	SHRIMP,U-Pb	Zircon	Ren Yan et al.	2006	Geological Bulletin of China(ICWEA)
2405	Outside of the MOO	Tianshan	D215-3	Kezidongkalasayi	granodiorite	91.86	41.67	350	7.0	SHRIMP,U-Pb	Zircon	Song Biao et al.	2006	Geological Bulletin of China(ICWEA)
2406	Outside of the MOO	Tianshan	NO.569		granite	84.36	43.33	312	2.5	SHRIMP,U-Pb	Zircon	Su WB et al.	2008	Xinjiang Geology (ICWEA)
2407	Outside of the MOO	Tianshan	04XJ-263	Southern Awulale Mountain	syenite	81.43	44.74	316	4.0	LA-ICP-MS,U-Pb	Zircon	Su WB et al.	2011	Lithos
2408	Outside of the MOO	Tianshan	06XJ-34		granite	82.58	43.62	317	3.0	LA-ICP-MS,U-Pb	Zircon	Sun Guihua et al.	2014	Lithos
2409	Outside of the MOO	Tianshan	06XJ017	Lamasu	granodiorite-porphyry	81.63	44.22	349	2.0	LA-ICP-MS,U-Pb	Zircon	Sun Guihua et al.	2014	Journal of Asian Earth Sciences
2410	Outside of the MOO	Tianshan	06XJ013		Granite porphyry	81.43	44.75	289	3.0	LA-ICP-MS,U-Pb	Zircon	Sun LH et al.	2010	Lithos
2411	Outside of the MOO	Tianshan	06XJ-04		Dacite	81.42	44.74	316	4.0	LA-ICP-MS,U-Pb	Zircon	Sun LS et al.	2010	Lithos
2412	Outside of the MOO	Tianshan	06XJ34		Granitic porphyry	81.60	44.21	349	2.0	LA-ICP-MS,U-Pb	Zircon	Tang et al.	2013	Journal of Asian Earth Sciences.
2413	Outside of the MOO	Tianshan		Huangshannan	granite	94.67	42.20	260	1.0	LA-ICP-MS,U-Pb	Zircon	Tang GJ et al	2008	Acta Petrologica Sinica (ICWEA)
2414	Outside of the MOO	Tianshan		Dabate	granite porphyry	81.52	44.75	289	2.0	LA-ICP-MS,U-Pb	Zircon	Tang GJ et al	2008	Acta Petrologica Sinica (ICWEA)
2415	Outside of the MOO	Tianshan	BLG13-1		Monzonite granite	77.24	40.88	283	2.8	LA-ICP-MS,U-Pb	Zircon	Tang GJ et al.	2008	Acta Petrologica Sinica (ICWEA)
2416	Outside of the MOO	Tianshan	KZI-9		granite	85.59	41.97	283	5.1	LA-ICP-MS,U-Pb	Zircon	Tang GJ et al.	2008	Acta Petrologica Sinica (ICWEA)
2417	Outside of the MOO	Tianshan	QEM14-1	Qiao（The Eastern Part ）Ma	Biotite granite	84.42	43.69	184	2.9	LA-ICP-MS,U-Pb	Zircon	Tang GJ et al.	2008	Acta Petrologica Sinica (ICWEA)
2418	Outside of the MOO	Tianshan	06XJ71	（Middle Part）Yangchang	Monzonite granite	88.45	42.35	252	4.0	LA-ICP-MS,U-Pb	Zircon	Tang GJ et al.		Acta Petrologica Sinica (ICWEA)
2419	Outside of the MOO	Tianshan	DB-1-9		Monzonite granite	76.54	40.11	285	2.7	LA-ICP-MS,U-Pb	Zircon	Tanggongjian et al.	2008	Acta Petrologica Sinica (ICWEA)
2420	Outside of the MOO	Tianshan	ST15-1-6	Tianshan（Southern Part ）	granite	87.73	41.89	285	3.0	LA-ICP-MS,U-Pb	Zircon	Tangjunhua et al.	2008	Acta Petrologica Sinica (ICWEA)
2421	Outside of the MOO	Tianshan	HDHLS-7		syenogranite	76.54	40.11	291	3.0	LA-ICP-MS,U-Pb	Zircon	This study		
2422	Outside of the MOO	Tianshan	DB-2-2		Qz syenite	76.54	40.11	285	3.0	LA-ICP-MS,U-Pb	Zircon	This study		
2423	Outside of the MOO	Tianshan	BYBLK-1①		alkali-feldspar granite	77.24	40.88	291	3.4	LA-ICP-MS,U-Pb	Zircon	This study		
2424	Outside of the MOO	Tianshan	ST15-1-4	Tianshan（Southern Part ）	granodiorite	87.72	41.86	288	3.0	LA-ICP-MS,U-Pb	Zircon	This study		
2425	Outside of the MOO	Tianshan	ST15-2-3	Tianshan（Southern Part ）	granodiorite	87.75	41.96	294	3.0	LA-ICP-MS,U-Pb	Zircon	This study		
2426	Outside of the MOO	Tianshan	ST15-3-5	Tianshan（Southern Part ）	granodiorite	87.76	41.97	300	3.0	LA-ICP-MS,U-Pb	Zircon	This study		
2427	Outside of the MOO	Tianshan	KEC-11		Monzonite granite	85.57	41.96	311	4.0	LA-ICP-MS,U-Pb	Zircon	This study		
2428	Outside of the MOO	Tianshan	KEC I-11		Garnet-bearing granite	85.59	41.97	282	4.0	LA-ICP-MS,U-Pb	Zircon	This study		
2429	Outside of the MOO	Tianshan	QGX-7		alkali-feldspar granite	86.21	42.02	302	3.3	LA-ICP-MS,U-Pb	Zircon	This study		
2430	Outside of the MOO	Tianshan	HLSI-11①②		syenogranite	85.98	42.05	292	8.1	LA-ICP-MS,U-Pb	Zircon	This study		
2431	Outside of the MOO	Tianshan	HLSI-12		Monzonite granite	85.97	42.10	301	4.8	LA-ICP-MS,U-Pb	Zircon	This study		
2432	Outside of the MOO	Tianshan	KMS15-1-3	Kumishi	quartz diorite	88.03	42.16	293	3.0	LA-ICP-MS,U-Pb	Zircon	This study		
2433	Outside of the MOO	Tianshan	KMS15-2-4	Kumishi	granite	88.03	42.15	301	3.0	LA-ICP-MS,U-Pb	Zircon	This study		
2434	Outside of the MOO	Tianshan	ST15-4-6	Tianshan（Southern Part ）	gabbro	88.40	42.28	304	3.0	LA-ICP-MS,U-Pb	Zircon	This study		
2435	Outside of the MOO	Tianshan	ST15-5-4	Tianshan（Southern Part ）	granite	88.47	42.29	292	3.0	LA-ICP-MS,U-Pb	Zircon	This study		
2436	Outside of the MOO	Tianshan	T15-5-3	Tianshan（Middle Part）	granite	87.33	42.35	304	3.0	LA-ICP-MS,U-Pb	Zircon	This study		
2437	Outside of the MOO	Tianshan	T15-7-4	Tianshan（Middle Part）	granodiorite	88.66	42.45	341	3.0	LA-ICP-MS,U-Pb	Zircon	This study		
2438	Outside of the MOO	Tianshan	798-2		diorite	86.23	42.45	302	4.4	LA-ICP-MS,U-Pb	Zircon	This study		
2439	Outside of the MOO	Tianshan	CWH-09		Granodiorite	86.23	42.45	302	4.0	LA-ICP-MS,U-Pb	Zircon	This study		
2440	Outside of the MOO	Tianshan	T15-9-1	Tianshan（Middle Part）	granodiorite	88.53	42.44	319	3.0	LA-ICP-MS,U-Pb	Zircon	This study		
2441	Outside of the MOO	Tianshan	T15-4-6	Tianshan（Middle Part）	granite	87.33	42.46	301	3.0	LA-ICP-MS,U-Pb	Zircon	This study		
2442	Outside of the MOO	Tianshan	ST15-6-9	（Southern Part ）Tianshan	granite	86.90	42.48	302	3.0	LA-ICP-MS,U-Pb	Zircon	This study		
2443	Outside of the MOO	Tianshan	T15-4-3	Tianshan（Middle Part）	granite	87.31	42.49	300	3.0	LA-ICP-MS,U-Pb	Zircon	This study		
2444	Outside of the MOO	Tianshan	T15-3-3	Tianshan（Middle Part）	granite	87.30	42.51	289	4.0	LA-ICP-MS,U-Pb	Zircon	This study		
2445	Outside of the MOO	Tianshan	T15-1-4	Tianshan（Middle Part）	granite	86.90	42.61	354	4.0	LA-ICP-MS,U-Pb	Zircon	This study		
2446	Outside of the MOO	Tianshan	WLST-6		alkali-feldspar granite	86.12	42.85	346	2.0	LA-ICP-MS,U-Pb	Zircon	This study		
2447	Outside of the MOO	Tianshan	WLST-3		biotiteMonzonite granite	86.64	42.90	313	4.4	LA-ICP-MS,U-Pb	Zircon	This study		
2448	Outside of the MOO	Tianshan	BLT-10		diorite	86.64	42.90	359	3.6	LA-ICP-MS,U-Pb	Zircon	This study		
2449	Outside of the MOO	Tianshan	MTSL-1		biotitediorite	86.12	42.91	311	6.3	LA-ICP-MS,U-Pb	Zircon	This study		
2450	Outside of the MOO	Tianshan	CT15-2-6	Tianshan（Middle Part）	granite	86.35	42.97	286	3.0	LA-ICP-MS,U-Pb	Zircon	This study		
2451	Outside of the MOO	Tianshan	CT15-1-6	Tianshan（Middle Part）	quartz diorite	86.79	43.06	323	3.0	LA-ICP-MS,U-Pb	Zircon	This study		
2452	Outside of the MOO	Tianshan	CT15-1-5	Tianshan（Middle Part）	Biotite granite	86.79	43.06	332	4.0	LA-ICP-MS,U-Pb	Zircon	This study		
2453	Outside of the MOO	Tianshan	LLSGL17-3-1	Lailisigaole	granodiorite	82.91	44.11	299	2.0	LA-ICP-MS,U-Pb	Zircon	This study		
2454	Outside of the MOO	Tianshan	XJ695	Boluokenuyanti	moyite	81.93	42.74	338	8.0	LA-ICP-MS,U-Pb	Zircon	Wang B et al.	2007	Acta Petrologica Sinica (ICWEA)
2455	Outside of the MOO	Tianshan	XJ626-1	Boluohuonuyanti	diorite	84.41	43.69	294	7.0	LA-ICP-MS,U-Pb	Zircon	Wang B et al.	2007	Acta Petrologica Sinica (ICWEA)
2456	Outside of the MOO	Tianshan	XJ702		Biotite K-granite	84.44	43.77	280	5.0	LA-ICP-MS,U-Pb	Zircon	Wang B et al.	2007	Acta Petrologica Sinica(ICWEA)
2457	Outside of the MOO	Tianshan	D05072-1	Hadonglikeshan	biotitegranite	84.45	43.77	301	7.0	LA-ICP-MS,U-Pb	Zircon	Wang B et al.	2007	Acta Petrologica Sinica (ICWEA)
2458	Outside of the MOO	Tianshan	XJ701	Boluohuonuyanti	biotitegranite	83.53	43.84	272	6.0	LA-ICP-MS,U-Pb	Zircon	Wang B et al.	2007	Acta Petrologica Sinica (ICWEA)
2459	Outside of the MOO	Tianshan	XJ694	Boluokenuyanti	biotitegranite	83.56	43.84	266	6.0	LA-ICP-MS,U-Pb	Zircon	Wang B et al.	2007	Acta Petrologica Sinica (ICWEA)
2460	Outside of the MOO	Tianshan	B101		Granodiorite-K-granite	84.43	43.71	294	7.0	LA-ICP-MS,U-Pb	Zircon	Wang B et al.	2009	International Journal of Earth Sciences
2461	Outside of the MOO	Tianshan	KMX13	Gangou	granite	84.44	43.77	280	5.0	LA-ICP-MS,U-Pb	Zircon	Wang B et al.	2009	Acta Petrologica Sinica (ICWEA)
2462	Outside of the MOO	Tianshan	B94		Granodiorite-K-granite	83.52	43.90	285	7.0	LA-ICP-MS,U-Pb	Zircon	Wang B et al.	2009	International Journal of Earth Sciences
2463	Outside of the MOO	Tianshan	05KC151		Monzonite granite	92.87	41.46	323	5.9	SHRIMP,U-Pb	Zircon	Wang Bo et al.	2006	Geological Bulletin of China(ICWEA)
2464	Outside of the MOO	Tianshan	XJ604	Kekesuyanti	moyite	77.24	40.88	273	2.0	LA-ICP-MS,U-Pb	Zircon	Wang Bo et al.	2007	Acta Petrologica Sinica (ICWEA)
2465	Outside of the MOO	Tianshan	XJ676	Kekesuyanti	moyite	81.90	42.82	341	6.0	LA-ICP-MS,U-Pb	Zircon	Wang Bo et al.	2007	Acta Petrologica Sinica (ICWEA)
2466	Outside of the MOO	Tianshan	P5TW1	Baodongtuyanti	diorite	81.97	44.51	348	2.2	SHRIMP,U-Pb	Zircon	Wang Bo et al.	2014	Master thesis: China University of Geosciences
2467	Outside of the MOO	Tianshan	KXX14	Kexiaxiyanzhu	diorite	81.97	44.51	351	3.8	SHRIMP,U-Pb	Zircon	Wang Bo et al.	2014	Master thesis: China University of Geosciences
2468	Outside of the MOO	Tianshan	KXX23	Kexiaxiyanzhu	diorite	81.97	44.51	356	2.2	SHRIMP,U-Pb	Zircon	Wang Bo et al.	2014	Master thesis: China University of Geosciences
2469	Outside of the MOO	Tianshan	KXX33	Kexiaxiyanzhu	granodiorite	81.97	44.51	357	3.0	SHRIMP,U-Pb	Zircon	Wang Chao et al.	2014	Master thesis: China University of Geosciences
2470	Outside of the MOO	Tianshan	Ta-4		K-feldspar granite	86.75	43.13	270	1.0	LA-ICP-MS,U-Pb	Zircon	Wang Degui et al.	2009	Acta Petrologica Sinica(ICWEA)
2471	Outside of the MOO	Tianshan	KXX11	Kexiaxiyanzhu	diorite	86.66	43.10	270	0.7	LA-ICP-MS,U-Pb	Zircon	Wang Honggang et al	2009	Acta Petrologica Sinica (ICWEA)
2472	Outside of the MOO	Tianshan		Tiangedongyanti	alkali-feldspar granite	86.63	43.17	270	0.7	LA-ICP-MS,U-Pb	Zircon	Wang Honggang et al	2009	Acta Petrologica Sinica (ICWEA)
2473	Outside of the MOO	Tianshan	TS11-27		biotitegranodiorite	88.54	42.46	320	2.0	LA-ICP-MS,U-Pb	Zircon	Wang Honggang et al	2014	Acta Petrologica Sinica (ICWEA)
2474	Outside of the MOO	Tianshan	B102	Borohoro Mountain	granite	88.52	42.51	330	2.3	LA-ICP-MS,U-Pb	Zircon	Wang Honggang et al	2014	Acta Petrologica Sinica (ICWEA)
2475	Outside of the MOO	Tianshan	DK12-46-1		Quartz diorite	84.43	43.73	300	2.0	LA-ICP-MS,U-Pb	Zircon	Wang JL et al.	2018	Journal of Asian Earth Sciences.
2476	Outside of the MOO	Tianshan	DK12-47-1		Granodiorite	84.43	43.70	305	1.0	LA-ICP-MS,U-Pb	Zircon	Wang Juli et al.	2018	Journal of Asian Earth Sciences.
2477	Outside of the MOO	Tianshan	DK12-45-1		Granodiorite	84.41	43.76	304	2.0	LA-ICP-MS,U-Pb	Zircon	Wang Juli et al.	2018	Journal of Asian Earth Sciences.

2478	Outside of the MOO	Tianshan	BL2	Baluntaiyibeidiqu	moyite	81.43	44.74	317	8.0	SHRIMP,U-Pb	Zircon	Wang M et al.	2006	Acta Geologica Sinica (ICWEA)
2479	Outside of the MOO	Tianshan	10CG12		Granite	84.89	43.21	328	0.9	LA-ICP-MS,U-Pb	Zircon	Wang M et al.	2018	Ore Geology Reviews
2480	Outside of the MOO	Tianshan	10CG26		Granodiorite	84.87	43.31	332	0.9	LA-ICP-MS,U-Pb	Zircon	Wang M et al.	2018	Ore Geology Reviews
2481	Outside of the MOO	Tianshan	DK12-43-1		Granodiorite	84.44	43.78	332	3.0	LA-ICP-MS,U-Pb	Zircon	Wang M et al.	2018	Journal of Asian Earth Sciences.
2482	Outside of the MOO	Tianshan	C14BL21		Ignimbrite	81.85	45.17	300	3.0	CAMECA,U-Pb	Zircon	Wang M et al.	2018	Journal of Asian Earth Sciences.
2483	Outside of the MOO	Tianshan	C14BL27		Rhyolite	81.55	45.18	303	1.0	CAMECA,U-Pb	Zircon	Wang M et al.	2018	Journal of Asian Earth Sciences.
2484	Outside of the MOO	Tianshan		Bailingshan	granite	91.25	41.83	297	3.0	SHRIMP,U-Pb	Zircon	Wang XS et al.	2005	Mineral Deposits(ICWEA)
2485	Outside of the MOO	Tianshan		Dabateyanti	granite porphyry	90.89	41.78	329	9.3	LA-ICP-MS,U-Pb	Zircon	Wang XS et al.	2006	Acta Petrologica Sinica (ICWEA)
2486	Outside of the MOO	Tianshan	Xhz-9		Fayalite syenite / Hb syenite	78.83	39.75	280	2.0	CAMECA,U-Pb	Zircon	Wang XS et al.	2011	Acta Petrologica Sinica(ICWEA)
2487	Outside of the MOO	Tianshan	n.g.		Rhyolite	81.22	42.94	340	4.0	LA-ICP-MS,U-Pb	Zircon	Wang XS et al.	2018	Geological Journal
2488	Outside of the MOO	Tianshan	X356-7	Hongyuntan	granodiorite	93.03	42.17	322	10.0	SHRIMP,U-Pb	Zircon	Wang Zhiliang et al.	2006	Geological Bulletin of China(ICWEA)
2489	Outside of the MOO	Tianshan	CHF1	Chihubanyantongkuangqu	granite porphyry	94.89	42.08	240	5.6	SHRIMP,U-Pb	Zircon	Wang ZP et al.	2015	Xinjiang Geology (ICWEA)
2490	Outside of the MOO	Tianshan		Bayingou	plagiogranite	84.83	44.02	325	7.1	SHRIMP,U-Pb	Zircon	Wanglongsheng et al.	2005	Geological Review(ICWEA)
2491	Outside of the MOO	Tianshan	JHB14-11	Jinghenan	biotitegranodiorite	82.87	44.15	196	3.9	SHRIMP,U-Pb	Zircon	Wei X and Xu YG	2008	Master's Thesis: Xinjiang University(ICWEA)
2492	Outside of the MOO	Tianshan	JHC14-11	Jinghenan	moyite	83.09	44.34	201	2.9	SHRIMP,U-Pb	Zircon	Wu Changzhi et al.	2008	Master's Thesis: Xinjiang University(ICWEA)
2493	Outside of the MOO	Tianshan	DMZ14-10	Dongmazha	moyite	82.10	44.09	305	3.1	SHRIMP,U-Pb	Zircon	Wu Hua et al.	2008	Master's Thesis: Xinjiang University(ICWEA)
2494	Outside of the MOO	Tianshan	10NLT01-1	Nalatiyanti	alkali-feldspar granite	94.89	42.24	276	1.1	SIMS,U-Pb	Zircon	Wu Jingang et al.	2015	Gondwana Research
2495	Outside of the MOO	Tianshan	08XY1-1	Qiakebuhe	granite	83.94	43.18	297	2.4	LA-ICP-MS,U-Pb	Zircon	Xiao Wenjiao et al.	2010	Acta Petrologica et Mineralogica(ICWEA)
2496	Outside of the MOO	Tianshan	03T- 96	Huochengxianguozigou	hornblende granite	81.11	43.24	348	0.8	TIMS,U-Pb	Zircon	Xu ShiQi .	2006	Northwestern Geology (ICWEA)
2497	Outside of the MOO	Tianshan	03T- 58	Zhaosumeikuangnan	granodiorite	84.46	43.71	287	0.8	TIMS,U-Pb	Zircon	Xu ShiQi .	2006	Northwestern Geology (ICWEA)
2498	Outside of the MOO	Tianshan	08TL1-4	Talmjirgal	biotitegranite	82.52	43.01	345	3.0	LA-ICP-MS,U-Pb	Zircon	Xu ShiQi .	2010	Acta Petrologica et Mineralogica(ICWEA)
2499	Outside of the MOO	Tianshan	03T- 127	Tuokexun314Guodao	alkali-feldspar granite	88.53	42.52	350	0.3	TIMS,U-Pb	Zircon	Xu Xingwang et al.	2006	Northwestern Geology (ICWEA)
2500	Outside of the MOO	Tianshan	03T-106	Huochengxianguozigou	hornblende granite	81.04	44.42	352	1.6	TIMS,U-Pb	Zircon	Xu XueYi et al.	2006	Northwestern Geology (ICWEA)
2501	Outside of the MOO	Tianshan	KK4-1		Monzonitic granite	82.04	42.87	352	9.0	LA-ICP-MS,U-Pb	Zircon	Xu Xueyi et al.	2010	Acta Petrologica Et Mineralogica(ICWEA).
2502	Outside of the MOO	Tianshan	TL2-7	Nalatiqiabuhebei	granodiorite	81.99	42.91	352	9.0	LA-ICP-MS,U-Pb	Zircon	Xu Xueyi et al.	2010	Northwestern Geology (ICWEA)
2503	Outside of the MOO	Tianshan	TL1-1	Nalati	Monzonite granite	83.86	43.46	349	7.0	LA-ICP-MS,U-Pb	Zircon	Xu Xueyi et al.	2010	Northwestern Geology (ICWEA)
2504	Outside of the MOO	Tianshan	XY2_1		Monzonitic granite	84.53	43.68	316		LA-ICP-MS,U-Pb	Zircon	Xu Xueyi et al.	2010	Acta Petrologica Et Mineralogica(ICWEA).
2505	Outside of the MOO	Tianshan	KK3-1	Kekesuhedongan	biotitegranite	83.56	44.27	321	7.0	LA-ICP-MS,U-Pb	Zircon	Xu XueYi et al.	2010	Northwestern Geology (ICWEA)
2506	Outside of the MOO	Tianshan	10QKS2-1		Granodiorite	82.25	42.87	314	3.0	LA-ICP-MS,U-Pb	Zircon	Xu Xueyi et al.	2013	Journal of Asian Earth Sciences.
2507	Outside of the MOO	Tianshan	10QKS1-3		Biotite monzonitic granite	82.25	42.86	337	1.0	LA-ICP-MS,U-Pb	Zircon	Xu Xueyi et al.	2013	Journal of Asian Earth Sciences.
2508	Outside of the MOO	Tianshan	08TL2-1		Quartz diorite	82.51	43.02	345	3.0	LA-ICP-MS,U-Pb	Zircon	Xu Xueyi et al.	2013	Journal of Asian Earth Sciences.
2509	Outside of the MOO	Tianshan	XXY2-1	Nalatiqiabuhebei	Monzonite granite	82.95	44.08	354	0.7	ICP-MS,U-Pb	Zircon	Xu XY et al.	2011	Earth Science Frontiers(ICWEA)
2510	Outside of the MOO	Tianshan	D7		Granodiorite porphyry	82.96	44.07	346	1.0	LA-ICP-MS,U-Pb	Zircon	Xu XY et al.	2011	Earth Science Frontiers(ICWEA)
2511	Outside of the MOO	Tianshan	10QLT2-1		Muscovite granite	83.45	43.12	357	8.0	LA-ICP-MS,U-Pb	Zircon	Xu XY et al.	2013	Journal of Asian Earth Sciences.
2512	Outside of the MOO	Tianshan	SCG2	Shuangchagou Area	granite	87.89	42.23	277	2.7	LA-ICP-MS,U-Pb	Zircon	Xu XY et al.	2015	Gondwana Research
2513	Outside of the MOO	Tianshan	776-1	Kumux	Syenite	87.87	42.23	277	2.2	LA-ICP-MS,U-Pb	Zircon	Xu XY et al.	2015	Gondwana Research
2514	Outside of the MOO	Tianshan	773	Kumux	Biotitegranite	88.53	42.40	283	2.3	LA-ICP-MS,U-Pb	Zircon	Xu XY et al.	2015	Gondwana Research
2515	Outside of the MOO	Tianshan	777-2	Kumux	K-feldspargranite	88.39	42.28	293	3.2	LA-ICP-MS,U-Pb	Zircon	Xue Chunji et al.	2015	Gondwana Research
2516	Outside of the MOO	Tianshan	769	Kumux	Diorite	88.32	42.27	295	4.2	LA-ICP-MS,U-Pb	Zircon	Xue CJ et al.	2015	Gondwana Research
2517	Outside of the MOO	Tianshan	D14		granodiorite-porphyry	82.45	43.68	302	4.0	LA-ICP-MS,U-Pb	Zircon	Yan Yonghong et al.	2013	Earth Science Frontiers(ICWEA)
2518	Outside of the MOO	Tianshan	QJSY	Qunjisayiyanti	granite porphyry	77.38	40.48	262	2.7	LA-ICP-MS,U-Pb	Zircon	Yang Fuquan et al.	2001	Earth Science Frontiers(ICWEA)
2519	Outside of the MOO	Tianshan	07Y-1011	tonghuashan	granodiorite	88.13	42.15	295	1.2	SHRIMP,U-Pb	Zircon	Yang Jingsui et al.	2011	Acta Petrologica Sinica (ICWEA)
2520	Outside of the MOO	Tianshan	07Y-1040		Quartz syenite porphyry	88.02	42.00	295	1.0	LA-ICP-MS,U-Pb	Zircon	Yang JS et al.	2011	Acta Petrologica Sinica(ICWEA)
2521	Outside of the MOO	Tianshan	YX8-1	Eastern End Of The Awulale M	diorite	84.22	43.41	284	3.6	LA-ICP-MS,U-Pb	Zircon	Yang W B et al.	2011	Gondwana Research
2522	Outside of the MOO	Tianshan	755	Eastern End Of The Awulale M	K-feldspargranite	84.22	43.41	311	2.1	LA-ICP-MS,U-Pb	Zircon	Yang W B et al.	2011	Gondwana Research
2523	Outside of the MOO	Tianshan	ALT-1101		Biotite monzonitic granite	82.37	45.11	297	2.0	LA-ICP-MS,U-Pb	Zircon	Yin JY et al.	2016	Gondwana Research.
2524	Outside of the MOO	Tianshan	TS1344		Granodiorite	86.91	42.87	354	2.0	LA-ICP-MS,U-Pb	Zircon	Yin JY et al.	2017	Gondwana Research.
2525	Outside of the MOO	Tianshan	TS1343		Granodiorite	86.90	42.91	354	2.0	LA-ICP-MS,U-Pb	Zircon	Yin JY et al.	2017	Gondwana Research.
2526	Outside of the MOO	Tianshan	MQS17-2-1	Mangqisu	two-mica granite	85.71	42.50	292	2.0	LA-ICP-MS,U-Pb	Zircon	Yu et al.	2020	Acta Geologica Sinica(ICWEA)
2527	Outside of the MOO	Tianshan	ST15-7-5	Mangqisu	tonalite	85.71	42.48	294	3.0	LA-ICP-MS,U-Pb	Zircon	Yu et al.	2020	Acta Geologica Sinica(ICWEA)
2528	Outside of the MOO	Tianshan	NLKC14-10	Nilekedongbei	biotitemoyite	83.70	43.84	300	2.8	LA-ICP-MS,U-Pb	Zircon	Yu Xue（middle part	2016	Doctoral thesis: China University of Geosciences
2529	Outside of the MOO	Tianshan	TC1	Hamidiqukumutage kuangchua	gabbro	93.18	41.68	306	3.4	SHRIMP,U-Pb	Zircon	Zhang Changqing et a	2010	Geological Bulletin of China(ICWEA)
2530	Outside of the MOO	Tianshan	TC2	Hamidiqukumutage kuangchua	gabbro	93.18	41.68	307	3.3	SHRIMP,U-Pb	Zircon	Zhang Changqing et a	2010	Geological Bulletin of China(ICWEA)
2531	Outside of the MOO	Tianshan	10Hl-9-1	Halegati	Monzonite granite	88.94	37.96	358	1.3	LA-ICP-MS,U-Pb	Zircon	Jiang Hanbing et al.	2014	Xinjiang Geology (ICWEA)
2532	Outside of the MOO	Tianshan	04XJ-73	Mazhashan	Syenite	78.83	39.74	286	2.9	SHRIMP,U-Pb	Zircon	Su Linhua et al.	2008	Journal of Jilin University (Earth Science Edition) (ICWEA)
2533	Outside of the MOO	Tokur Terrane	C-760	Ingaglinsk Complex	Granodiorite	132.27	53.06	254	6.0	LA-ICP-MS,U-Pb	Zircon	Sorokin, Andrey A. et	2021	Lihthos
2534	Outside of the MOO	Tokur Terrane	C-742	Ingaglinsk Complex	Granodiorite	133.13	53.12	253	4.0	LA-ICP-MS,U-Pb	Zircon	Sorokin, Andrey A. et	2021	Lihthos
2535	Outside of the MOO	W Mongolia	1-65/01	Hyargas Nuur Lake	Picritoid	93.18	49.10	335	5.4	SHRIMP,U-Pb	Zircon	Doroshkevich A. G. e	2013	Geology
2536	Outside of the MOO	W. Junggar	1702	Urastai	Granidiorite	81.63	45.20	294	4.0	SHRIMP,U-Pb	Zircon	Chen Bihe et al.	2007	Acta Petrologica Sinica(ICWEA).
2537	Outside of the MOO	W. Junggar	KLMS	Kurumsu	Moyite	84.12	46.30	302	2.0	SHRIMP,U-Pb	Zircon	Chen Jiafu et al.	2010	Lithos.
2538	Outside of the MOO	W. Junggar	SLK	Seric	Moyite	84.58	46.38	304	2.0	SHRIMP,U-Pb	Zircon	Chen Jiafu et al.	2010	Lithos.
2539	Outside of the MOO	W. Junggar	No.1 KLMS	Kurumsu	Moyite	84.02	46.39	302	2.0	SHRIMP,U-Pb	Zircon	Chen Jiafu et al.	2010	Lithos.
2540	Outside of the MOO	W. Junggar	NO.2 SLK	Sailike	Moyite	84.54	46.43	304	2.0	SHRIMP,U-Pb	Zircon	Chen Jiafu et al.	2010	Lithos.
2541	Outside of the MOO	W. Junggar	TLG	Toroghe	Granite	85.17	46.45	281	4.0	SHRIMP,U-Pb	Zircon	Chen Jiafu et al.	2010	Lithos.
2542	Outside of the MOO	W. Junggar	NO.17 TLG	Toroghe	Granidiorite	85.15	46.78	281	3.0	SHRIMP,U-Pb	Zircon	Chen Jiafu et al.	2010	Lithos.
2543	Outside of the MOO	W. Junggar	NO.15 DYS	Dainsu	Pyroxene diorite	84.34	46.90	345	3.0	SHRIMP,U-Pb	Zircon	Chen Jiafu et al.	2010	Lithos.
2544	Outside of the MOO	W. Junggar	DYS	Dainsu	Pyroxene diorite	84.35	46.90	346	3.0	SHRIMP,U-Pb	Zircon	Chen Jiafu et al.	2010	Lithos.
2545	Outside of the MOO	W. Junggar	ABDL	Abdullah	Monzogranite	83.28	46.99	332	3.0	SHRIMP,U-Pb	Zircon	Chen Jiafu et al.	2010	Lithos.
2546	Outside of the MOO	W. Junggar	No.14 ABDL	Abdullah	Monzogranite	83.30	46.97	332	3.0	SHRIMP,U-Pb	Zircon	Chen Jiafu et al.	2010	Lithos.
2547	Outside of the MOO	W. Junggar	BEG	Burgan	Granidiorite	83.08	47.07	332	3.0	SHRIMP,U-Pb	Zircon	Chen Jiafu et al.	2010	Lithos.
2548	Outside of the MOO	W. Junggar	No.13 BEG	Buergan	Granidiorite	83.15	47.09	332	3.0	SHRIMP,U-Pb	Zircon	Chen Jiafu et al.	2010	Lithos.
2549	Outside of the MOO	W. Junggar	ZQS	Zhu Qingshan	Granidiorite	83.01	47.10	325	3.0	SHRIMP,U-Pb	Zircon	Chen Jiafu et al.	2010	Lithos.
2550	Outside of the MOO	W. Junggar	NO.12 ZQS	Zhuqingshan rock mass	Granidiorite	83.03	47.10	325	3.0	SHRIMP,U-Pb	Zircon	Chen Jiafu et al.	2010	Lithos.
2551	Outside of the MOO	W. Junggar	NO.16 LST	Last	Moyite	85.69	47.17	321	5.0	LA-ICP-MS,U-Pb	Zircon	Chen Jiafu et al.	2010	Lithos.
2552	Outside of the MOO	W. Junggar	LST	Rust	Moyite	85.57	47.19	321	5.0	LA-ICP-MS,U-Pb	Zircon	Chen Jiafu et al.	2010	Lithos.
2553	Outside of the MOO	W. Junggar	XZ-17	Yegzkala	Bi-Monzogranite	84.81	46.01	308	3.0	SHRIMP,U-Pb	Zircon	Chen Shi et al.	2010	Acta Petrologica Sinica(ICWEA).
2554	Outside of the MOO	W. Junggar	TW5-TW13-7	Zaskar Kayi	Quartz-diorite	83.20	45.91	315	5.0	SHRIMP,U-Pb	Zircon	Chen Y.,et al.	2006	Geological Bulletin of China(ICWEA).
2555	Outside of the MOO	W. Junggar	TW5-T13-7	Barruk	Quartz-diorite	83.20	45.91	315	5.0	SHRIMP,U-Pb	Zircon	Chen Y.,et al.	2006	Geological Bulletin of China(ICWEA).

2556	Outside of the MOO	W. Junggar	HT	Hatu	Monzogranite	84.14	46.04	320	3.2	LA-ICP-MS,U-Pb	Zircon	Di Pengfei et al.	2010	Master's thesis.Lanzhou University.(ICWEA).
2557	Outside of the MOO	W. Junggar	SED	Seldon	Monzogranite	84.13	46.19	309	2.5	LA-ICP-MS,U-Pb	Zircon	Di Pengfei et al.	2010	Master's thesis.Lanzhou University.(ICWEA).
2558	Outside of the MOO	W. Junggar	TST	Tast	Monzogranite	86.12	47.13	337	4.0	LA-ICP-MS,U-Pb	Zircon	Fan Yu et al.	2007	Acta Petrologica Sinica(ICWEA).
2559	Outside of the MOO	W. Junggar	627KY5	Karamay	Granite porphyry	84.85	45.65	315	1.0	LA-ICP-MS,U-Pb	Zircon	Feng Qianwen et al.	2012	Acta Petrologica Sinica(ICWEA).
2560	Outside of the MOO	W. Junggar	627KY1	Karamay	Diorite porphyrite	84.83	45.68	303	1.2	LA-ICP-MS,U-Pb	Zircon	Feng Qianwen et al.	2012	Acta Petrologica Sinica(ICWEA).
2561	Outside of the MOO	W. Junggar	627KY2	Karamay	Bi-Monzogranite	84.83	45.68	319	1.0	LA-ICP-MS,U-Pb	Zircon	Feng Qianwen et al.	2012	Acta Petrologica Sinica(ICWEA).
2562	Outside of the MOO	W. Junggar	929-3	Red Mountain	Diorite porphyrite	85.16	45.97	302	1.0	LA-ICP-MS,U-Pb	Zircon	Feng Qianwen et al.	2012	Acta Petrologica Sinica(ICWEA).
2563	Outside of the MOO	W. Junggar	929-4a	Red Mountain	Diorite	85.16	45.97	302	1.0	LA-ICP-MS,U-Pb	Zircon	Feng Qianwen et al.	2012	Acta Petrologica Sinica(ICWEA).
2564	Outside of the MOO	W. Junggar	930-8	Red Mountain	Diorite	85.07	45.97	303	1.0	LA-ICP-MS,U-Pb	Zircon	Feng Qianwen et al.	2012	Acta Petrologica Sinica(ICWEA).
2565	Outside of the MOO	W. Junggar	930-9	Red Mountain	Bi-Monzogranite	85.07	45.97	304	1.0	LA-ICP-MS,U-Pb	Zircon	Feng Qianwen et al.	2012	Acta Petrologica Sinica(ICWEA).
2566	Outside of the MOO	W. Junggar	930-1	Red Mountain	Diorite porphyrite	85.16	46.00	302	1.0	LA-ICP-MS,U-Pb	Zircon	Feng Qianwen et al.	2012	Acta Petrologica Sinica(ICWEA).
2567	Outside of the MOO	W. Junggar	930-2	Red Mountain	Diorite	85.16	46.00	304	1.0	LA-ICP-MS,U-Pb	Zircon	Feng Qianwen et al.	2012	Acta Petrologica Sinica(ICWEA).
2568	Outside of the MOO	W. Junggar	MEG	Miaoergou	Granite	83.83	45.50	306	8.8	LA-ICP-MS,U-Pb	Zircon	Gao Shanlin et al.	2006	Xinjiang Geology(ICWEA).
2569	Outside of the MOO	W. Junggar	KLMY	Karamay	Granite	84.77	45.70	317	3.6	LA-ICP-MS,U-Pb	Zircon	Gao Shanlin et al.	2006	Xinjiang Geology(ICWEA).
2570	Outside of the MOO	W. Junggar	AKBS	Akbastau	Granite	84.22	45.72	318	2.9	LA-ICP-MS,U-Pb	Zircon	Gao Shanlin et al.	2006	Xinjiang Geology(ICWEA).
2571	Outside of the MOO	W. Junggar	AK154-2	Akebasitao	Alkali-feldspar granite	84.26	45.67	305	4.0	LA-ICP-MS,U-Pb	Zircon	Geng Yuansheng et a	2009	Chemical Geology.
2572	Outside of the MOO	W. Junggar	AK6	Akbastau	Diorite enclave	84.36	45.76	304	2.0	LA-ICP-MS,U-Pb	Zircon	Geng Yuansheng et a	2009	Chemical Geology.
2573	Outside of the MOO	W. Junggar	KM9802-11	Karamay	Diorite enclave	84.82	45.70	306	5.0	LA-ICP-MS,U-Pb	Zircon	Geng Yuansheng et a	2010	Chemical Geology.
2574	Outside of the MOO	W. Junggar	KM9918-3	Karamay	Alkali-feldspar granite	83.75	45.65	296	7.0	LA-ICP-MS,U-Pb	Zircon	Geng Yuansheng et a	2011	Chemical Geology.
2575	Outside of the MOO	W. Junggar	MG123-2	Miaoergou	Charnockite	83.87	45.52	305	3.0	SHRIMP,U-Pb	Zircon	Geng Yuansheng et a	2012	Chemical Geology.
2576	Outside of the MOO	W. Junggar	MG164-2	Miaoergou	Charnockite	83.95	45.52	305	3.0	LA-ICP-MS,U-Pb	Zircon	Geng Yuansheng et a	2013	Chemical Geology.
2577	Outside of the MOO	W. Junggar	MG9811-1	Miaoergou	Charnockite	84.03	45.55	296	3.0	LA-ICP-MS,U-Pb	Zircon	Geng Yuansheng et a	2014	Chemical Geology.
2578	Outside of the MOO	W. Junggar	MG9951-1	Miaoergou	Alkali-feldspar granite	83.88	45.53	298	4.0	LA-ICP-MS,U-Pb	Zircon	Geng Yuansheng et a	2015	Chemical Geology.
2579	Outside of the MOO	W. Junggar	KLMY3	Karamay (North)	Granite	84.81	45.68	318	5.0	LA-ICP-MS,U-Pb	Zircon	Han Baofu et al.	2006	Acta Petrologica Sinica(ICWEA).
2580	Outside of the MOO	W. Junggar	TLG1	Targen	Moyite	83.57	45.69	287	6.0	SHRIMP,U-Pb	Zircon	Han Baofu et al.	2006	Acta Petrologica Sinica(ICWEA).
2581	Outside of the MOO	W. Junggar	KLMY21	Karamay North	Moyite	84.86	45.75	315	6.0	SHRIMP,U-Pb	Zircon	Han Baofu et al.	2006	Acta Petrologica Sinica(ICWEA).
2582	Outside of the MOO	W. Junggar	KLMY16	Akbastau	Moyite	84.36	45.79	276	5.0	SHRIMP,U-Pb	Zircon	Han Baofu et al.	2006	Acta Petrologica Sinica(ICWEA).
2583	Outside of the MOO	W. Junggar	Gn-32	Hatu (Ushete)	Moyite	84.08	46.02	302	4.0	LA-ICP-MS,U-Pb	Zircon	Han Baofu et al.	2006	Acta Petrologica Sinica(ICWEA).
2584	Outside of the MOO	W. Junggar	Gn-9	Tiechanggou	Moyite	84.34	46.15	308	4.0	LA-ICP-MS,U-Pb	Zircon	Han Baofu et al.	2006	Acta Petrologica Sinica(ICWEA).
2585	Outside of the MOO	W. Junggar	Gn-29	Doranale	Diorite	82.77	46.39	300	4.0	SHRIMP,U-Pb	Zircon	Han Baofu et al.	2006	Acta Petrologica Sinica(ICWEA).
2586	Outside of the MOO	W. Junggar	Gn29	Doranale	Diorite	82.76	46.42	300	4.0	LA-ICP-MS,U-Pb	Zircon	Han Baofu et al.	2006	Acta Petrologica Sinica(ICWEA).
2587	Outside of the MOO	W. Junggar	Gn-40	Kuztau	Granidiorite	84.58	46.90	325	4.0	LA-ICP-MS,U-Pb	Zircon	Han Baofu et al.	2006	Acta Petrologica Sinica(ICWEA).
2588	Outside of the MOO	W. Junggar	Gn-71	South Slope of Sawuer Mounta	Bi-moyite	85.71	46.99	338	4.0	SHRIMP,U-Pb	Zircon	Han Baofu et al.	2006	Acta Petrologica Sinica(ICWEA).
2589	Outside of the MOO	W. Junggar	Gn-14	Tacheng Basin North	Bi-moyite	83.42	47.11	303	4.0	LA-ICP-MS,U-Pb	Zircon	Han Baofu et al.	2006	Acta Petrologica Sinica(ICWEA).
2590	Outside of the MOO	W. Junggar	Gn-76	SaWuEr mountain	Granite	86.07	47.26	299	4.0	SHRIMP,U-Pb	Zircon	Han Baofu et al.	2006	Acta Petrologica Sinica(ICWEA).
2591	Outside of the MOO	W. Junggar	KL-19	Karamay	Diorite	84.78	45.71	316	2.8	SHRIMP,U-Pb	Zircon	He Jingbo et al.	2011	Earth Science Frontiers(ICWEA).
2592	Outside of the MOO	W. Junggar	70-RGC-85	Karamay East	Apatite in alkaline granite	84.87	45.67	321	6.7	TIMS,U-Pb	Zircon	Kwon(1989)	1989	
2593	Outside of the MOO	W. Junggar	13JC1-4	Well JC1 in the Jimunai Basin	Basalt	85.67	47.33	313	2.1	CAMECA,U-Pb	Zircon	Li Di et al.	2015	Journal of Asian Earth ences.
2594	Outside of the MOO	W. Junggar	07HT112	V rock mass	Granodiorite	84.55	45.47	311	3.3	SHRIMP,U-Pb	Zircon	Liu Y.l.,et al.	2009	Geology and Exploration(ICWEA).
2595	Outside of the MOO	W. Junggar	KWSY26	Kasamblac	Granite	82.47	44.96	292	4.9	LA-ICP-MS,U-Pb	Zircon	Liu Zhiqiang et al.	2005	Acta Petrologica Sinica(ICWEA).
2596	Outside of the MOO	W. Junggar	KWSY7	Kong Wu Sai		82.37	45.28	298	6.0	LA-ICP-MS,U-Pb	Zircon	Liu Zhiqiang et al.	2005	Acta Petrologica Sinica(ICWEA).
2597	Outside of the MOO	W. Junggar	KWSY16	Bole	Rhyolite	82.40	45.32	271	7.0	LA-ICP-MS,U-Pb	Zircon	Liu Zhiqiang et al.	2005	Acta Petrologica Sinica(ICWEA).
2598	Outside of the MOO	W. Junggar	08TW01	Coxon Ophiolite	Gabbro	86.88	47.50	337	1.3	LA-ICP-MS,U-Pb	Zircon	Ni Kang.,et al.	2013	Northwestern Geology(ICWEA).
2599	Outside of the MOO	W. Junggar	09AL25	Coxon Ophiolite	Gabbro	86.72	47.57	332	1.5	LA-ICP-MS,U-Pb	Zircon	Ni Kang.,et al.	2013	Northwestern Geology(ICWEA).
2600	Outside of the MOO	W. Junggar	AKBST	Akbastau	Monzogranite	84.36	45.78	296		LA-ICP-MS,U-Pb	Zircon	Pang Zhenjia et al.	2007	Master's thesis.Chang`an University.(ICWEA).
2601	Outside of the MOO	W. Junggar	H T	Hatu	Monzogranite	84.13	46.03	320	3.2	LA-ICP-MS,U-Pb	Zircon	Shang Zhaocong et al	2012	Master's thesis.Lanzhou University.(ICWEA).
2602	Outside of the MOO	W. Junggar	SLD	Seldon	Monzogranite	84.14	46.19	309	2.5	LA-ICP-MS,U-Pb	Zircon	Shang Zhaocong et al	2012	Master's thesis.Lanzhou University.(ICWEA).
2603	Outside of the MOO	W. Junggar	D07057	Targen	Monzogranite	83.54	45.77	299	2.5	SHRIMP,U-Pb	Zircon	Song Biao et al.	2011	Geological Bulletin of China(ICWEA).
2604	Outside of the MOO	W. Junggar	M1	Miaoergou	Alkali-feldspar granite	83.85	45.53	305	2.0	LA-ICP-MS,U-Pb	Zircon	Su Yuping et al.	2006	Geochemistry(ICWEA).
2605	Outside of the MOO	W. Junggar	K9	Karamay	Alkali-feldspar granite	84.75	45.71	296	4.0	LA-ICP-MS,U-Pb	Zircon	Su Yuping et al.	2006	Geochemistry(ICWEA).
2606	Outside of the MOO	W. Junggar	AS3	Akbastau	Alkali-feldspar granite	84.28	45.76	303	3.0	LA-ICP-MS,U-Pb	Zircon	Su Yuping et al.	2006	Geochemistry(ICWEA).
2607	Outside of the MOO	W. Junggar	H7	Red Mountain	Alkali-feldspar granite	85.19	45.97	301	4.0	LA-ICP-MS,U-Pb	Zircon	Su Yuping et al.	2006	Geochemistry(ICWEA).
2608	Outside of the MOO	W. Junggar	TAST	Tast	Monzogranite	86.13	47.15	337	4.0	LA-ICP-MS,U-Pb	Zircon	Sun Wanlong.,et al.	2018	Geological Bulletin of China(ICWEA).
2609	Outside of the MOO	W. Junggar	QQH-14	Xalxikay	Alkali-feldspar granite	86.25	47.75	291	9.3	LA-ICP-MS,U-Pb	Zircon	Tan Lugui.,et al.	2008	Journal of Jilin University（Earth Science Edition）(ICWEA).
2610	Outside of the MOO	W. Junggar	06XJ04	Dabate	Dacite	81.43	44.74	316	4.0	LA-ICP-MS,U-Pb	Zircon	Tang G.J. et al.	2010	Lithos.
2611	Outside of the MOO	W. Junggar	06XJ-153	Baugutu	Diorite porphyrite	84.44	45.40	314	4.0	LA-ICP-MS,U-Pb	Zircon	Tang G.J. et al.	2010	Chemical Geology
2612	Outside of the MOO	W. Junggar	06XJ-147	Baugutu	Quartz diorite porphyry	84.54	45.47	311	4.0	LA-ICP-MS,U-Pb	Zircon	Tang G.J. et al.	2010	Chemical Geology
2613	Outside of the MOO	W. Junggar	06XJ134	Karamay	Diorite dike	85.76	45.68	309	3.0	LA-ICP-MS,U-Pb	Zircon	Tang G.J. et al.	2012	Lithos.
2614	Outside of the MOO	W. Junggar	06XJ136	Karamay	Granite	84.74	45.69	314	3.0	LA-ICP-MS,U-Pb	Zircon	Tang G.J. et al.	2012	Lithos.
2615	Outside of the MOO	W. Junggar	06XJ182	Karamay	Granite	84.74	45.69	316	3.0	LA-ICP-MS,U-Pb	Zircon	Tang G.J. et al.	2012	Lithos.
2616	Outside of the MOO	W. Junggar	06XJ131	Karamay	Biotite granite	85.57	45.71	312	3.0	LA-ICP-MS,U-Pb	Zircon	Tang G.J. et al.	2012	Lithos.
2617	Outside of the MOO	W. Junggar	06XJ123	Karamay	Granite porphyry	85.87	45.70	304	3.0	SHRIMP,U-Pb	Zircon	Tang G.J. et al.	2012	Lithos.
2618	Outside of the MOO	W. Junggar	06XJ121-2	Karamay	Monzonite enclave	85.89	45.72	309	3.0	LA-ICP-MS,U-Pb	Zircon	Tang G.J. et al.	2012	Lithos.
2619	Outside of the MOO	W. Junggar	06XJ120	Karamay	Granite	84.87	45.76	304	3.0	LA-ICP-MS,U-Pb	Zircon	Tang G.J. et al.	2012	Lithos.
2620	Outside of the MOO	W. Junggar	DB	Dabat	Granite porphyry	81.43	44.75	317	8.0	SHRIMP,U-Pb	Zircon	Tang G.J., et al.	2008	Acta Petrologica Sinica(ICWEA).
2621	Outside of the MOO	W. Junggar	P20TW2	Laba	Granidiorite	83.18	45.36	295	2.3	LA-ICP-MS,U-Pb	Zircon	Wei Rongzhu et al.	2010	Acta Petrologica et Mineralogica(ICWEA).
2622	Outside of the MOO	W. Junggar	P20TW1	Laba	Granidiorite	83.17	45.40	287	5.0	LA-ICP-MS,U-Pb	Zircon	Wei Rongzhu et al.	2010	Acta Petrologica et Mineralogica(ICWEA).
2623	Outside of the MOO	W. Junggar		Akebasitao	Alkali-feldspar granite	84.42	45.75	303	3.0	SHRIMP,U-Pb	Zircon	Xu G.Y.	2018	Master's thesis.China University Of Geosciences.(ICWEA).
2624	Outside of the MOO	W. Junggar	RM	Red Mountain	Alkali-feldspar granite	85.15	45.97	305	4.0	SHRIMP,U-Pb	Zircon	Xu Xin et al.	2006	Geology in China(ICWEA).
2625	Outside of the MOO	W. Junggar	KLMY	Karamay	Moyite	84.76	45.71	308	7.0	SHRIMP,U-Pb	Zircon	Xu Xin et al.	2010	Acta Petrologica Sinica(ICWEA).
2626	Outside of the MOO	W. Junggar	AKBS T	Akbastau	Moyite	84.35	45.79	290	8.0	SHRIMP,U-Pb	Zircon	Xu Xin et al.	2010	Acta Petrologica Sinica(ICWEA).
2627	Outside of the MOO	W. Junggar	H T	Hatu	Granite	84.14	46.04	302	4.0	SHRIMP,U-Pb	Zircon	Xu Xin et al.	2010	Acta Petrologica Sinica(ICWEA).
2628	Outside of the MOO	W. Junggar	TCG	Tiechanggou	Moyite	84.51	46.15	308	4.0	SHRIMP,U-Pb	Zircon	Xu Xin et al.	2010	Acta Petrologica Sinica(ICWEA).
2629	Outside of the MOO	W. Junggar	09MYL-37	Aketasi	Granidiorite	83.28	45.61	307	2.0	LA-ICP-MS,U-Pb	Zircon	Xu Z., et al.	2012	Lithos.
2630	Outside of the MOO	W. Junggar	09JBL-4	Zhanbeileta	Granidiorite	83.50	45.65	311	1.0	LA-ICP-MS,U-Pb	Zircon	Xu Z., et al.	2012	Lithos.
2631	Outside of the MOO	W. Junggar	09MYL-56	Shangdebulake	Diorite dike	83.26	45.67	318	4.0	SIMS,U-Pb	Zircon	Xu Z., et al.	2012	Lithos.
2632	Outside of the MOO	W. Junggar	09JBL-3	Jiangbule	Alkali-feldspar granite	83.46	45.68	309	2.0	LA-ICP-MS,U-Pb	Zircon	Xu Z., et al.	2012	Lithos.
2633	Outside of the MOO	W. Junggar	08MYL-53	Aketasi	Granidiorite	83.32	45.68	308	2.0	LA-ICP-MS,U-Pb	Zircon	Xu Z., et al.	2012	Lithos.

2634	Outside of the MOO	W. Junggar	08MYL-54	Aketasi	Granidiorite	83.34	45.70	313	3.0	SHRIMP,U-Pb	Zircon	Xu Z., et al.	2012	Lithos.
2635	Outside of the MOO	W. Junggar	08TLG-1	Taergen	Alkali-feldspar granite	83.52	45.75	309	4.0	LA-ICP-MS,U-Pb	Zircon	Xu Z., et al.	2012	Lithos.
2636	Outside of the MOO	W. Junggar	OK-16	Wakensala	Monzogranite	86.30	47.02	324	6.0	SHRIMP,U-Pb	Zircon	Yuan Feng et al.	2006	Acta Geologica Sinica(ICWEA).
2637	Outside of the MOO	W. Junggar	OK-17	Wakensala	Monzogranite	86.29	47.01	324	6.2	SHRIMP,U-Pb	Zircon	Yuan Feng et al.	2006	Acta Petrologica Sinica(ICWEA).
2638	Outside of the MOO	W. Junggar	STS-1	Centas	Monzogranite	86.03	47.07	328	6.0	SHRIMP,U-Pb	Zircon	Yuan Feng et al.	2006	Acta Geologica Sinica(ICWEA).
2639	Outside of the MOO	W. Junggar	TST-10	Tast	Monzogranite	86.06	47.21	314	3.2	SHRIMP,U-Pb	Zircon	Yuan Feng et al.	2006	Acta Petrologica Sinica(ICWEA).
2640	Outside of the MOO	W. Junggar	TST-15	Tast	Monzogranite	86.06	47.21	314	3.2	SHRIMP,U-Pb	Zircon	Yuan Feng et al.	2006	Acta Petrologica Sinica(ICWEA).
2641	Outside of the MOO	W. Junggar	KY-5	Koitas	Alkali-feldspar granite	86.18	47.22	298	4.6	SHRIMP,U-Pb	Zircon	Yuan Feng et al.	2006	Acta Petrologica Sinica(ICWEA).
2642	Outside of the MOO	W. Junggar	KY-5	Koytas	Alkali-feldspar granite	85.23	47.42	298	4.6	SHRIMP,U-Pb	Zircon	Yuan Feng et al.	2006	Acta Geologica Sinica(ICWEA).
2643	Outside of the MOO	W. Junggar	QQH-14	Chaqihai	Alkali-feldspar granite	86.34	47.44	291	9.3	SHRIMP,U-Pb	Zircon	Yuan Feng et al.	2006	Acta Petrologica Sinica(ICWEA).
2644	Outside of the MOO	W. Junggar	13XTS-139	Qiyi (Utubulak) Rock Mass	Granite porphyry	82.04	44.79	332	4.2	LA-ICP-MS,U-Pb	Zircon	Zhang Chengli et al.		Unpublished.
2645	Outside of the MOO	W. Junggar	13XTS-219	Santai Rock	Medium coarse grained porphyritic	82.06	44.86	325	4.7	LA-ICP-MS,U-Pb	Zircon	Zhang Chengli et al.		Unpublished.
2646	Outside of the MOO	W. Junggar	13XTS-136	Tussay Pluton	Granite porphyry	82.27	45.01	312	11.0	LA-ICP-MS,U-Pb	Zircon	Zhang Chengli et al.		Unpublished.
2647	Outside of the MOO	W. Junggar	13XTS-132	Tussay Pluton	Granite porphyry	82.28	45.02	325	4.1	LA-ICP-MS,U-Pb	Zircon	Zhang Chengli et al.		Unpublished.
2648	Outside of the MOO	W. Junggar	13XTS-179	Chahawusu Pluton	Gray-white medium coarse-grained	80.54	45.05	299	7.2	LA-ICP-MS,U-Pb	Zircon	Zhang Chengli et al.		Unpublished.
2649	Outside of the MOO	W. Junggar	13XTS-121	Tussay Rock	Granite porphyry dike	82.38	45.07	308	3.4	LA-ICP-MS,U-Pb	Zircon	Zhang Chengli et al.		Unpublished.
2650	Outside of the MOO	W. Junggar	13XTS-120	Tussay Pluton	Cyclic porphyry granite	82.38	45.07	310	2.3	LA-ICP-MS,U-Pb	Zircon	Zhang Chengli et al.		Unpublished.
2651	Outside of the MOO	W. Junggar	13XTS-144	Aksuman Rock	Coarse gray-white porphyritic biotit	81.12	45.09	306	5.6	LA-ICP-MS,U-Pb	Zircon	Zhang Chengli et al.		Unpublished.
2652	Outside of the MOO	W. Junggar	08TW02	Kekesentao	granodiorite	86.74	47.54	286	1.6	LA-ICP-MS,U-Pb	Zircon	Zhang Chuanlin et al.	2012	Journal of Asian Earth ences.
2653	Outside of the MOO	W. Junggar	TW02	Kekesentao area	granodiorite	86.74	47.54	316	2.1	LA-ICP-MS,U-Pb	Zircon	Zhang Chuanlin et al.	2012	Journal of Asian Earth ences.
2654	Outside of the MOO	W. Junggar	08TW03	Kekesentao area	potassic granite	86.71	47.58	300	1.6	LA-ICP-MS,U-Pb	Zircon	Zhang Chuanlin et al.	2012	Journal of Asian Earth ences.
2655	Outside of the MOO	W. Junggar		Karjiao	ivernite	86.28	47.18	303	8.0	SHRIMP,U-Pb	Zircon	Zhou Taofa et al.	2007	Acta Geologica Sinica.
2656	Outside of the MOO	W. Junggar	OK-4	Wakensala	Monzogranite	86.30	47.02	324	6.0	SHRIMP,U-Pb	Zircon	Zhou Taofa et al.	2008	Acta Petrologica Sinica(ICWEA).
2657	Outside of the MOO	W. Junggar	STS-1	Centas	Monzogranite	86.03	47.07	328	6.0	SHRIMP,U-Pb	Zircon	Zhou Taofa et al.	2008	Acta Petrologica Sinica(ICWEA).
2658	Outside of the MOO	W. Junggar	KYTS	Koytas	Moyite	86.18	47.19	298	5.0	SHRIMP,U-Pb	Zircon	Zhou Taofa et al.	2008	Acta Petrologica Sinica(ICWEA).
2659	Outside of the MOO	W. Junggar	KEJ	Karan	Granite porphyry	86.36	47.19	302	7.0	SHRIMP,U-Pb	Zircon	Zhou Taofa et al.	2008	Acta Petrologica Sinica(ICWEA).
2660	Outside of the MOO	W. Junggar	QQH	Xalxikay	Moyite	86.32	47.43	291	9.0	SHRIMP,U-Pb	Zircon	Zhou Taofa et al.	2008	Acta Petrologica Sinica(ICWEA).
2661	Within the MOO		D0925	Undifferentiated	Granodiorite	105.00	46.15	296	3.0	LA-ICP-MS,U-Pb	Zircon	Ariuntsetseg Ganbat e	2021	Lithos
2662	Within the MOO		D1710	Batkhaan	Syenogranite	104.13	47.07	282	3.0	LA-ICP-MS,U-Pb	Zircon	Ariuntsetseg Ganbat e	2021	Lithos
2663	Within the MOO		D1709	Batkhaan	Rhyolite	104.77	47.08	274	2.0	LA-ICP-MS,U-Pb	Zircon	Ariuntsetseg Ganbat e	2021	Lithos
2664	Within the MOO		D1742	Delgerkhaan	Monzogranite	104.62	46.67	240	3.0	LA-ICP-MS,U-Pb	Zircon	Ariuntsetseg Ganbat e	2021	Lithos
2665	Within the MOO		D0815	Delgerkhaan	Granodiorite	104.51	46.94	238	2.5	LA-ICP-MS,U-Pb	Zircon	Ariuntsetseg Ganbat e	2021	Lithos
2666	Within the MOO		D0817	Delgerkhaan	Granodiorite	104.46	46.97	236	2.0	LA-ICP-MS,U-Pb	Zircon	Ariuntsetseg Ganbat e	2021	Lithos
2667	Within the MOO		D1718	Delgerkhaan	Monzogranite	104.04	47.14	230	1.6	LA-ICP-MS,U-Pb	Zircon	Ariuntsetseg Ganbat e	2021	Lithos
2668	Within the MOO		D1726	Zambalkhudag	Granodiorite	104.34	46.94	220	2.0	LA-ICP-MS,U-Pb	Zircon	Ariuntsetseg Ganbat e	2021	Lithos

Note: MOO = Mongol-Okhotsk Orogen; E. = Eastern; S. = Southern; N. = Northern; W. = Western; C. = Central; SE = Southeastern; NE= Northeastern; SW = Southwestern; Bi = bitiote; Hb = hornblende; Mus = muscovite; ICWEA = In Chinese with English abstract

Unpublished data analytical methods:

Sample name starting with M17

1) In-situ zircon U–Pb isotopic analysis was performed at the National Research Center for Geoanalysis, Chinese Academy of Geological Sciences, using a Thermo Elmetll ICPMS attached to a New Wave 193 nm laser ablation system with an in-house sample cell. Standard zircon GEMOC GJ-1 ($^{207}Pb/^{206}Pb$ age of 601.7±1.3 Ma) was used as external standards, while Plesovice (intercept age of 337.1±0.4 Ma) was used to control the accuracy. Samples were analyzed in runs of ca. 15 analyses including 5 zircon standards and 10 sample points. Most analyses were carried out using a beam with a 35μm diameter and a repetition rate of 10 Hz. U-Pb ages were calculated from the raw signal data using the on-line software package GLITTER (ver. 4.4) (http://www.mq.edu.au/GEMOC). The results are plotted using Isoplot3.0 (Ludwig, 2003).

Sample name starting with M13, M14, N17, N14 and etc

2) U–Pb Zircon geochronological analysis was completed in the Continental Tectonics and Dynamics Laboratory of Institute of Geology, Chinese Academy of Geological Sciences. U-Pb abundance data were measured by the latest Neptune Plus multiple collector ICP-MS (MC-ICP-MS) of Thermo Fisher Co. Ltd. The laser-ablation system used in the measurement is developed by the GeoLasPro 193nm invented by the U.S. Coherent Co. Ltd. Helium was used as a carrier gas to enhance transport efficiency of ablated material. The spot size of laser ablation beam is 24-44μm., whose size is 32μm in normal conditions, adopted more in experiment. Laser energy adopted in the measurements was 10J/cm^2 in energy density and 8 Hz in frequency. U and Pb in zircon were ionized in plasma with hyperthermia up to 8000°C. Using a dynamic zoom and extended dispersion, the instrument is able to collect U-Pb isotopes' mass numbers of great differences. In-situ measurement of U-Pb isotope can be done within the micro area of zircon. During the measurement, the instrument spent 4 seconds measuring the background and 23 seconds measuring every spot. The MC-ICP-MS operating conditions were optimized with the measurements of reference zircon 91500, to provide the maximum sensitivity and maintain minimum oxide production rate of ThO+Th<2% as well as lowest background. The accuracy of the data is verified by using GJ-1 as auxiliary standard. The MC-ICP-MS measurement was carried out using time resolved analysis operated in fast peak-hopping and DUAL detector mode using a short integration time. The Harvard standard zircon 91500 and one GJ-1 were measured for every 5-10 sample spots. The data was calculated by the ICPMSDataCal program in ref. Liu et al.,2009 and the Isoplot program in ref. Ludwig, 2003.

Sample name starting with M15, M16

3) LA-ICP-MS U–Pb dating was performed on an excimer (193 nm wavelength) LA-ICP-MS at the State Key Laboratory of Continental Dynamics at Northwest University in Xi'an following the methods described by Yuan et al. (2003). An Agilent 7500a (Dynamic Reaction Cell) inductively coupled plasma-mass spectrometer (ICP-MS) from Perkin Elmer/SCIEX was used. The GeoLas 200 M laser-ablation system was used for the laser ablation experiments. Sites for dating were selected based on the CL and photomicrograph images. Spots with diameters of 30 - 40 μm were used. Common Pb corrections were made using the methods described by Andersen (2002). In addition, the U, Th and Pb concentrations were calibrated by using 29Si as an internal standard and NIST SRM 610 as a reference standard. The isotopic ratios were calculated using GLITTER 4.0 (Macquarie University) and were corrected for both instrumental mass bias and depth-dependent elemental and isotopic fractionation by using Harvard zircon 91500 as an external standard. Concordia diagrams and weighted mean ages were produced using the ISOPLOT/Ex 3.23 program (Ludwig, 2003).

Sample name starting with M12 and B70

4) Zircon U-Pb isotopic analysis was completed at the Isotope Laboratory of Tianjin Center, China Geological Survey. Zircon dating was conducted on Agilent 7500a inductively coupled plasma mass spectrometer (ICP-MS) equipped with the New Wave 193 nmFX laser ablation system. The laser system delivered a beam ofUV light (193 nm) from a frequency-quintupled Neptune instrument(Thermo Fisher Company). Analyses were carried out with abeam diameter of 35 μm, repetition rate of 8–10 Hz and energyof 10–11 J/cm2. Data acquisition for each analysis was 20 s forthe background and 40 s for the signal. Mass discrimination withthe MS and residual elemental fractionation were corrected bycalibration against a homogeneous zircon standard (TEMORA/GJ-1). Typical operating conditions of the ICP-MS in the experimentand detailed analytical procedures from Liu et al. (2008) werestrictly followed. Off-line selection, background integration, analysissignals and time-drift correction and quantitative calibration wereconducted with the in-house software (ICPMSDataCal) (Liu etal., 2008). Common Pb corrections were processed with nonradiogenic 204Pb, following the method of Andersen (2002), andused an average present-day crustal composition (Stacey andKramers, 1975) as the common Pb which was assumed to beintroduced mainly from surface contamination or the gold coatingduring sample preparation. Concordia diagrams and age calculationswere made using Isoplot (v 3.0) (Ludwig, 2003).

Supplementary table captions

Table S2 Nd isotopic data of granitic rocks of the Mongol-Okhotsk Orogen.

Table S2 Nd isotopic data of the granitic rocks of the Mongol-Okhotsk Orogen

No.	Sample	Tectonic unit	Location and unit	Longitude	Latitude	Lithology	Pluton	Age	Sm	Nd	$^{147}Sm/^{144}Nd$	$^{143}Nd/^{144}Nd$	2σ	εNd(0)	$f_{Sm/Nd}$	εNd(t)	T_{DM2} (Ga)	References	
1	NM 1	N. of MOS	Transbaikalia-Mongolia	105.54	50.26	Orthogneiss	Suhbaatar	211	16.57	2.86	0.1042	0.512626	12	-0.23	-0.47	2.3	0.81	Donskaya et al., 2008	Journal of the Geological Society, London
2	NM 2	N. of MOS	Transbaikalia-Mongolia	105.64	50.2025	Granodioritic gneiss	Suhbaatar	230	12.04	1.79	0.0901	0.512614	12	-0.47	-0.54	2.7	0.79	Donskaya et al., 2008	Journal of the Geological Society, London
3	NM 4	N. of MOS	Transbaikalia-Mongolia	105.35	50.38389	Syenite	Suhbaatar	266	86.62	14.38	0.1004	0.512305	9	-6.5	-0.49	3.2	1.30	Donskaya et al., 2008	Journal of the Geological Society, London
4	M02/107	N. of MOS	Transbaikalia-Mongolia	104.82	49.90083	Granodiorite	Suhbaatar	240	20.05	3.4	0.1026	0.512685	13	0.917	-0.48	3.9	0.70	Donskaya et al., 2008	Journal of the Geological Society, London
5	M02/109	N. of MOS	Transbaikalia-Mongolia	104.89	49.96972	Granite	Suhbaatar	229	38.87	6.07	0.0944	0.51266	10	0.429	-0.52	3.4	0.73	Donskaya et al., 2008	Journal of the Geological Society, London
6	M02/111	N. of MOS	Transbaikalia-Mongolia	105.10	50.30306	Felsic metavolcanicrock	Suhbaatar	265	20.53	3.13	0.0921	0.5125	12	-2.69	-0.53	0.7	0.96	Donskaya et al., 2008	Journal of the Geological Society, London
7	178B	N. of MOS	Transbaikalia-Mongolia	111.50	54.08	Carbonate, Nepheline syenite	Snezhny	480	25.80	150	0.0998	0.512237		-7.8	-0.49	-1.9	1.36	Doroshkevich et al., 2012	Lithos
8	8099	N. of MOS	Transbaikalia-Mongolia	111.50	54.08	Apatite, Nepheline syenite	Snezhny	480	357	2202	0.0981	0.51226		-7.4	-0.50	-1.3	1.32	Doroshkevich et al., 2012	Lithos
9	178	N. of MOS	Transbaikalia-Mongolia	111.50	54.08	Nepheline syenite	Snezhny	480	7.80	45.90	0.1027	0.512244		-7.7	-0.48	-1.9	1.37	Doroshkevich et al., 2012	Lithos
10	44-9a	N. of MOS	Transbaikalia-Mongolia	111.87	54.13	Carbonate, urtite	Mukhalski	300	4.75	20.50	0.1403	0.512545		-1.8	-0.29	0.3	1.03	Doroshkevich et al., 2012	Lithos
11	22-MK	N. of MOS	Transbaikalia-Mongolia	111.87	54.13	Apatite, urtite	Mukhalski	300	103	585	0.1058	0.512496		-2.8	-0.46	0.7	1.00	Doroshkevich et al., 2012	Lithos
12	59-7	N. of MOS	Transbaikalia-Mongolia	111.87	54.13	Nepheline syenite	Mukhalski	300	0.79	4.47	0.1073	0.512491		-2.9	-0.45	0.6	1.02	Doroshkevich et al., 2012	Lithos
13	126-24	N. of MOS	Transbaikalia-Mongolia	111.87	54.13	Ijolite	Mukhalski	300	2.19	9.36	0.1415	0.512642		0.1	-0.28	2.2	0.88	Doroshkevich et al., 2012	Lithos
14	725/5	N. of MOS	Transbaikalia-Mongolia	111.83	54.13	Carbonate, Nepheline syenite	Verkhne-Burulzayski	300	4.31	25.60	0.1017	0.512531		-2.1	-0.48	1.5	0.94	Doroshkevich et al., 2012	Lithos
15	723/4	N. of MOS	Transbaikalia-Mongolia	111.83	54.13	Nepheline syenite	Verkhne-Burulzayski	300	6.13	29.70	0.1247	0.512585		-1.0	-0.37	1.7	0.92	Doroshkevich et al., 2012	Lithos
16	367	N. of MOS	Transbaikalia-Mongolia	111.80	54.08	Pyroxenite	Nizhne-Burulzayski	500	3.34	13.60	0.1488	0.512642		0.1	-0.24	3.1	0.97	Doroshkevich et al., 2012	Lithos
17	369	N. of MOS	Transbaikalia-Mongolia	111.80	54.08	Ijolite	Nizhne-Burulzayski	500	3.13	12.00	0.1577	0.512658		0.4	-0.20	2.9	0.99	Doroshkevich et al., 2012	Lithos
18	7922	N. of MOS	Transbaikalia-Mongolia	111.85	54.12	Pyroxenite	Sayzhenski	500	8.54	37.50	0.1375	0.512643		0.1	-0.30	3.9	0.91	Doroshkevich et al., 2012	Lithos
19	7931	N. of MOS	Transbaikalia-Mongolia	111.85	54.12	Nepheline syenite	Sayzhenski	500	2.27	14.70	0.0935	0.512495		-2.8	-0.52	3.8	0.92	Doroshkevich et al., 2012	Lithos
20	7892	N. of MOS	Transbaikalia-Mongolia	111.85	54.12	Ijolite	Sayzhenski	500	4.77	23.30	0.1237	0.512647		0.2	-0.37	4.8	0.83	Doroshkevich et al., 2012	Lithos
21	175	N. of MOS	Transbaikalia-Mongolia	111.85	54.12	Ijolite	Sayzhenski	500	2.37	13.80	0.1040	0.512343		-5.8	-0.47	0.2	1.21	Doroshkevich et al., 2012	Lithos
22	7871	N. of MOS	Transbaikalia-Mongolia	111.85	54.12	Pyroxenite	Sayzhenski	500	8.77	41.00	0.1293	0.512461		-3.5	-0.34	0.8	1.16	Doroshkevich et al., 2012	Lithos
23	506	N. of MOS	Transbaikalia-Mongolia	111.58	54.20	Alkaline syenite	Amalat	300	2.66	14.70	0.1095	0.512573		-1.3	-0.44	2.1	0.89	Doroshkevich et al., 2012	Lithos
24	A-509	N. of MOS	Transbaikalia-Mongolia	111.58	54.20	Carbonate, Nepheline syenite	Amalat	300	4.49	24.60	0.1105	0.512557		-1.6	-0.44	1.7	0.92	Doroshkevich et al., 2012	Lithos
25	509	N. of MOS	Transbaikalia-Mongolia	111.58	54.20	Ijolite	Amalat	300	7.32	42.80	0.1035	0.512561		-1.5	-0.47	2.1	0.89	Doroshkevich et al., 2012	Lithos
26	B627	N. of MOS	Transbaikalia-Mongolia	108.40	50.64	PA syenite	Bryansky	280	18.74	113.5	0.0999	0.512317		-6.3	-0.49	-2.8	1.27	Litvinovsky B.A. et al., 2002	Chemical Geology
27	B388	N. of MOS	Transbaikalia-Mongolia	108.42	50.59	PA granite	Bryansky	280	10.92	56.44	0.1170	0.512364		-5.3	-0.41	-2.5	1.25	Litvinovsky B.A. et al., 2002	Chemical Geology
28	B626	N. of MOS	Transbaikalia-Mongolia	108.36	50.58	AFS syenite	Bryansky	280	16.37	97.70	0.1013	0.512293		-6.7	-0.49	-3.3	1.32	Litvinovsky B.A. et al., 2002	Chemical Geology
29	B626-1	N. of MOS	Transbaikalia-Mongolia	108.45	50.61	AFS syenite	Bryansky	280	9.61	62.43	0.0931	0.512301		-6.6	-0.53	-2.9	1.28	Litvinovsky B.A. et al., 2002	Chemical Geology
30	B425	N. of MOS	Transbaikalia-Mongolia	108.39	50.57	AFS granite	Bryansky	280	2.09	14.73	0.0858	0.512320		-6.2	-0.56	-2.2	1.23	Litvinovsky B.A. et al., 2002	Chemical Geology
31	A447-4	N. of MOS	Transbaikalia-Mongolia	108.41	50.62	Comendite	Bryansky	280	21.81	116.1	0.1135	0.512356		-5.5	-0.42	-2.5	1.25	Litvinovsky B.A. et al., 2002	Chemical Geology
32	B382-2	N. of MOS	Transbaikalia-Mongolia	108.32	50.88	Comendite	Bryansky	280	15.35	76.07	0.1220	0.512342		-5.8	-0.38	-3.1	1.30	Litvinovsky B.A. et al., 2002	Chemical Geology
33	M-347	N. of MOS	Transbaikalia-Mongolia	110.35	51.58	AFS syenite	Khorinsk	280	10.18	62.51	0.0985	0.512295		-6.7	-0.50	-3.2	1.30	Jahn et al., 2009	Lithos
34	3070	N. of MOS	Transbaikalia-Mongolia	110.34	51.57	AFS granite	Khorinsk	280	7.98	44.69	0.1080	0.512311		-6.4	-0.45	-3.2	1.31	Jahn et al., 2009	Lithos
35	M-340	N. of MOS	Transbaikalia-Mongolia	110.36	51.55	PA granite	Khorinsk	280	5.06	30.63	0.1000	0.512352		-5.6	-0.49	-2.1	1.22	Jahn et al., 2009	Lithos
36	479	N. of MOS	Transbaikalia-Mongolia	110.29	51.59	PA granite	Khorinsk	280	6.07	35.98	0.1020	0.512253		-7.5	-0.48	-4.1	1.38	Jahn et al., 2009	Lithos
37	M-350	N. of MOS	Transbaikalia-Mongolia	110.31	51.61	PA granite	Khorinsk	280	7.20	38.91	0.1119	0.512379		-5.1	-0.43	-2.0	1.21	Jahn et al., 2009	Lithos
38	M-492	N. of MOS	Transbaikalia-Mongolia	110.33	51.60	AFS rhyolite	Khorinsk	285	6.74	31.91	0.1276	0.512327		-6.1	-0.35	-3.6	1.34	Jahn et al., 2009	Lithos
39	M-511	N. of MOS	Transbaikalia-Mongolia	110.32	51.54	AFS rhyolite	Khorinsk	285	8.95	45.96	0.1177	0.512357		-5.5	-0.40	-2.6	1.26	Jahn et al., 2009	Lithos
40	M-501	N. of MOS	Transbaikalia-Mongolia	110.27	51.59	Syenite	Khorinsk	280	5.43	34.99	0.0938	0.512311		-6.4	-0.52	-2.7	1.27	Jahn et al., 2009	Lithos
41	530	N. of MOS	Transbaikalia-Mongolia	110.38	51.57	AFS syenite	Khorinsk	280	4.00	21.00	0.1152	0.512415		-4.4	-0.41	-1.4	1.16	Jahn et al., 2009	Lithos
42	536	N. of MOS	Transbaikalia-Mongolia	110.35	51.62	AFS granite	Khorinsk	280	12.00	59.00	0.1230	0.512279		-7.0	-0.37	-4.4	1.40	Jahn et al., 2009	Lithos
43	B341	N. of MOS	Transbaikalia-Mongolia	110.39	51.57	Syenite	Khorinsk	280	11.00	68.00	0.0978	0.512246		-7.6	-0.50	-4.1	1.38	Jahn et al., 2009	Lithos
44	B361-20	N. of MOS	Transbaikalia-Mongolia	110.41	51.56	Syenite	Khorinsk	280	3.30	17.49	0.1141	0.512238		-7.8	-0.42	-4.9	1.44	Jahn et al., 2009	Lithos
45	B111	N. of MOS	Transbaikalia-Mongolia	110.42	51.62	Monzonite	Khorinsk	280	6.50	36.31	0.1082	0.512263		-7.3	-0.45	-4.2	1.38	Jahn et al., 2009	Lithos
46	B144	N. of MOS	Transbaikalia-Mongolia	110.40	51.53	Syenite	Khorinsk	270	6.62	35.95	0.1113	0.512297		-6.7	-0.43	-3.7	1.34	Jahn et al., 2009	Lithos
47	B440	N. of MOS	Transbaikalia-Mongolia	112.17	50.78	Comendite	Tsagan-Khurtei	210	11.43	61.28	0.1128	0.512670		0.6	-0.43	2.9	0.75	Litvinovsky B.A. et al., 2002	Chemical Geology
48	B444	N. of MOS	Transbaikalia-Mongolia	112.09	50.69	Comendite	Tsagan-Khurtei	210	18.74	100.5	0.1127	0.512676		0.7	-0.43	3.0	0.74	Litvinovsky B.A. et al., 2002	Chemical Geology
49	1/8a	N. of MOS	Transbaikalia-Mongolia	112.03	50.73	comendite	Tsagan-Khurtei	210	12.10	66.70	0.1097	0.512625		-0.3	-0.44	2.1	0.82	Litvinovsky B.A. et al., 2002	Chemical Geology
50	A13	N. of MOS	Transbaikalia-Mongolia	112.53	50.75	PA granite	Atha	210	3.80	17.00	0.1351	0.512657		0.4	-0.31	2.0	0.82	Jahn et al., 2009	Lithos
51	A13-1	N. of MOS	Transbaikalia-Mongolia	112.39	50.69	PA granite	Atha	210	17.00	82.00	0.1253	0.512664		0.5	-0.36	2.4	0.79	Jahn et al., 2009	Lithos
52	L716	N. of MOS	Transbaikalia-Mongolia	112.48	50.43	PA granite	Atha	210	11.00	65.00	0.1023	0.512622		-0.3	-0.48	2.2	0.81	Jahn et al., 2009	Lithos
53	L722	N. of MOS	Transbaikalia-Mongolia	112.51	50.66	PA granite	Atha	210	12.00	70.00	0.1036	0.512619		-0.4	-0.47	2.1	0.81	Jahn et al., 2009	Lithos
54	L725	N. of MOS	Transbaikalia-Mongolia	112.59	50.78	PA granite	Atha	210	11.00	64.00	0.1039	0.512616		-0.4	-0.47	2.1	0.82	Jahn et al., 2009	Lithos
55	L726	N. of MOS	Transbaikalia-Mongolia	112.47	50.34	PA syenite	Atha	210	10.00	58.00	0.1042	0.512620		-0.4	-0.47	2.1	0.81	Jahn et al., 2009	Lithos
56	L727	N. of MOS	Transbaikalia-Mongolia	112.56	50.65	PA syenite	Atha	210	12.00	65.00	0.1116	0.512652		0.3	-0.43	2.6	0.78	Jahn et al., 2009	Lithos
57	L728	N. of MOS	Transbaikalia-Mongolia	112.53	50.73	PA syenite	Atha	210	11.00	65.00	0.1023	0.512634		-0.1	-0.48	2.5	0.79	Jahn et al., 2009	Lithos
58	A65	N. of MOS	Transbaikalia-Mongolia	112.58	50.75	PA granite	Atha	210	6.00	30.00	0.1209	0.512556		-1.6	-0.39	0.4	0.95	Jahn et al., 2009	Lithos
59	A66	N. of MOS	Transbaikalia-Mongolia	112.56	50.69	PA granite	Atha	210	6.00	32.00	0.1134	0.512585		-1.0	-0.42	1.2	0.89	Jahn et al., 2009	Lithos
60	B69-1	N. of MOS	Transbaikalia-Mongolia	112.54	50.73	Afs syenite	Atha	230	10.00	67.00	0.0902	0.512515		-2.4	-0.54	0.7	0.95	Jahn et al., 2009	Lithos
61	B69-2	N. of MOS	Transbaikalia-Mongolia	112.59	50.68	Afs syenite	Atha	230	5.00	33.00	0.0916	0.512514		-2.4	-0.53	0.7	0.95	Jahn et al., 2009	Lithos
62	A519	N. of MOS	Transbaikalia-Mongolia	112.57	50.71	PA syenite	Atha	220	5.33	28.23	0.1141	0.512689		1.0	-0.42	3.3	0.73	Jahn et al., 2009	Lithos
63	A516a	N. of MOS	Transbaikalia-Mongolia	112.55	50.81	PA syenite	Atha	220	7.00	42.00	0.1008	0.512718		1.6	-0.49	4.3	0.65	Jahn et al., 2009	Lithos
64	KH14	N. of MOS	Transbaikalia-Mongolia	107.68	50.39	Afs syenite	Kharitonovo	230	12.00	67.00	0.1083	0.512468		-3.3	-0.45	-0.7	1.06	Jahn et al., 2009	Lithos
65	870	N. of MOS	Transbaikalia-Mongolia	107.64	50.41	AFS syenite	Kharitonovo	230	14.00	77.00	0.1099	0.512513		-2.4	-0.44	0.1	1.00	Jahn et al., 2009	Lithos
66	3008	N. of MOS	Transbaikalia-Mongolia	107.59	50.38	AFS granite	Kharitonovo	230	4.80	31.00	0.0936	0.512336		-5.9	-0.52	-2.9	1.24	Jahn et al., 2009	Lithos
67	866	N. of MOS	Transbaikalia-Mongolia	107.65	50.42	PA syenite	Kharitonovo	230	29.00	155	0.1131	0.512496		-2.8	-0.43	-0.3	1.03	Jahn et al., 2009	Lithos
68	Jan-34	N. of MOS	Transbaikalia-Mongolia	107.91	51.30	Monzonite	Oshurkovo	130	14.46	93.20	0.0938	0.512451		-3.6	-0.52	-1.9	1.08	Jahn et al., 2009	Lithos
69	Jan-37	N. of MOS	Transbaikalia-Mongolia	107.92	51.29	Monzodiorite	Oshurkovo	130	25.11	153.1	0.0992	0.512437		-3.9	-0.50	-2.3	1.11	Jahn et al., 2009	Lithos

No.	Sample	Tectonic unit	Location and unit	Longitude	Latitude	Lithology	Pluton	Age	Sm	Nd	$^{147}Sm/^{144}Nd$	$^{143}Nd/^{144}Nd$	2σ	εNd(0)	$f_{Sm/Nd}$	εNd(t)	T_{DM2} (Ga)	References	
70	Jan-65	N. of MOS	Transbaikalia-Mongolia	107.89	51.28	AFS syenite	Oshurkovo	130	6.12	48.10	0.0769	0.512387		-4.9	-0.61	-2.9	1.16	Jahn et al., 2009	Lithos
71	14	N. of MOS	Transbaikalia-Mongolia	107.90	51.32	AFS syenite	Oshurkovo	130	7.14	55.90	0.0772	0.512358		-5.5	-0.61	-3.5	1.21	Jahn et al., 2009	Lithos
72	A102	N. of MOS	Transbaikalia-Mongolia	107.88	51.27	PA granite	Oshurkovo	150	5.45	33.40	0.0986	0.512427		-4.1	-0.50	-2.2	1.12	Jahn et al., 2009	Lithos
73	B590	N. of MOS	Transbaikalia-Mongolia	107.86	51.31	PA granite	Oshurkovo	150	6.92	38.50	0.1087	0.512438		-3.9	-0.45	-2.2	1.12	Jahn et al., 2009	Lithos
74	13023	N. of MOS	Transbaikalia-Mongolia	119.50	56.33	Granite	Katugin	155	30.10	143.9	0.1263	0.511656		-19.2	-0.36	-17.8	2.38	Larin A. M. et al.,2015	DES
75	14382	N. of MOS	Transbaikalia-Mongolia	119.52	56.35	Granite	Katugin	155	63.10	234	0.1630	0.512156		-9.4	-0.17	-8.7	1.64	Larin A. M. et al.,2015	DES
76	C-54-8	N. of MOS	Transbaikalia-Mongolia	119.53	56.35	Granite	Katugin	155	38.80	155.7	0.1507	0.511919		-14.0	-0.23	-13.1	2.00	Larin A. M. et al.,2015	DES
77	C-6-1	N. of MOS	Transbaikalia-Mongolia	119.48	56.35	Granite	Katugin	155	32.90	128.1	0.1551	0.512044		-11.6	-0.21	-10.8	1.81	Larin A. M. et al.,2015	DES
78	C-15-1	N. of MOS	Transbaikalia-Mongolia	119.48	56.35	Granite	Katugin	155	78.50	167.4	0.2837	0.513723		21.2	0.44	19.4	-0.65	Larin A. M. et al.,2015	DES
79	C-38A-1	N. of MOS	Transbaikalia-Mongolia	119.48	56.37	Granite	Katugin	155	73.20	195.4	0.2266	0.513014		7.3	0.15	6.7	0.40	Larin A. M. et al.,2015	DES
80	C-54-36	N. of MOS	Transbaikalia-Mongolia	119.47	56.37	Granite	Katugin	155	55.50	216	0.1557	0.511994		-12.6	-0.21	-11.8	1.89	Larin A. M. et al.,2015	DES
81	C-94-1	N. of MOS	Transbaikalia-Mongolia	119.47	56.37	Granite	Katugin	155	52.60	219	0.1452	0.511907		-14.3	-0.26	-13.2	2.01	Larin A. M. et al.,2015	DES
82	C-54-27	N. of MOS	Transbaikalia-Mongolia	119.47	56.37	Granite	Katugin	155	153.7	671	0.1385	0.511780		-16.7	-0.30	-15.6	2.20	Larin A. M. et al.,2015	DES
83	C-121-1	N. of MOS	Transbaikalia-Mongolia	119.47	56.37	Granite	Katugin	155	184.9	642	0.1740	0.512331		-6.0	-0.12	-5.5	1.38	Larin A. M. et al.,2015	DES
84	Zg51/2-03	N. of MOS	Transbaikalia-Mongolia	111.00	54.50	Bi-granite	Barguzin	325	1.44	7.00	0.12466	0.512093		-10.6	-0.37	-7.6	1.70	Tsygankov A.A.,2014	RGG
85	Zg64/3-04	N. of MOS	Transbaikalia-Mongolia	113.70	54.42	Bi-granite	Barguzin	325	0.86	4.30	0.12035	0.512148		-9.6	-0.39	-6.4	1.60	Tsygankov A.A.,2014	RGG
86	Zg64-04a	N. of MOS	Transbaikalia-Mongolia	111.17	55.33	Bi-granite	Barguzin	325	3.75	22.03	0.10286	0.511781		-16.7	-0.48	-12.8	2.12	Tsygankov A.A.,2014	RGG
87	Gl-8/3-03	N. of MOS	Transbaikalia-Mongolia	111.00	55.00	Bi-granite	Barguzin	313	2.41	13.17	0.11045	0.512152		-9.5	-0.44	-6.0	1.56	Tsygankov A.A.,2014	RGG
88	PR-050-04a	N. of MOS	Transbaikalia-Mongolia	113.83	56.30	Qtz monzonite	Romanovka	278	3.98	27.26	0.08832	0.512273		-7.1	-0.55	-3.3	1.31	Tsygankov A.A.,2014	RGG
89	Kh-6-03	N. of MOS	Transbaikalia-Mongolia	113.83	56.30	Qtz monzonite	Khangintui	302	6.27	35.54	0.10659	0.512093		-10.6	-0.46	-7.2	1.64	Tsygankov A.A.,2014	RGG
90	Bu-114-06a	N. of MOS	Transbaikalia-Mongolia	113.83	56.30	Qtz syenite	Burgasy	287	5.12	31.41	0.09860	0.512129		-9.9	-0.50	-6.3	1.57	Tsygankov A.A.,2014	RGG
91	002/1-04	N. of MOS	Transbaikalia-Mongolia	114.00	54.40	Granite	Angyr	303	1.96	11.78	0.10038	0.512163		-9.3	-0.49	-5.5	1.51	Tsygankov A.A.,2014	RGG
92	044/1-04	N. of MOS	Transbaikalia-Mongolia	104.17	50.50	Granite	Angyr	303	2.10	12.01	0.10561	0.512178		-9.0	-0.46	-5.5	1.51	Tsygankov A.A.,2014	RGG
93	543/4-04a	N. of MOS	Transbaikalia-Mongolia	114.33	54.40	Granite	Ulen-Burgasy	293	9.03	30.17	0.18102	0.512056		-11.4	-0.08	-10.8	1.91	Tsygankov A.A.,2014	RGG
94	Sh-07-25	N. of MOS	Transbaikalia-Mongolia	114.00	54.00	Qtz syenite	Shaluta	300	4.37	21.30	0.12412	0.512201		-8.5	-0.37	-5.7	1.53	Tsygankov A.A.,2014	RGG
95	174-05a	N. of MOS	Transbaikalia-Mongolia	110.00	52.17	Granite	Ulekchin	300	4.60	27.21	0.10225	0.512490		-2.9	-0.48	0.7	1.00	Tsygankov A.A.,2014	RGG
96	177-05a	N. of MOS	Transbaikalia-Mongolia	110.00	52.17	Qtz syenite	Ulekchin	300	5.87	32.69	0.10863	0.512539		-1.9	-0.45	1.4	0.94	Tsygankov A.A.,2014	RGG
97	162/4-05b	N. of MOS	Transbaikalia-Mongolia	104.17	50.50	Granite	Sharatala	286	5.53	31.60	0.10579	0.512114		-10.2	-0.46	-6.9	1.61	Tsygankov A.A.,2014	RGG
98	162-05b	N. of MOS	Transbaikalia-Mongolia	110.00	52.17	Granite	Sharatala	286	16.60	96.00	0.10453	0.512105		-10.4	-0.47	-7.0	1.62	Tsygankov A.A.,2014	RGG
99	162/6-05b	N. of MOS	Transbaikalia-Mongolia	114.00	54.40	Granite	Sharatala	286	6.31	32.90	0.11595	0.512093		-10.6	-0.41	-7.7	1.67	Tsygankov A.A.,2014	RGG
100	166-05b	N. of MOS	Transbaikalia-Mongolia	104.17	50.50	Granite	Sharatala	286	6.24	37.50	0.10059	0.512141		-9.7	-0.49	-6.2	1.55	Tsygankov A.A.,2014	RGG
101	Xc-59-02a	N. of MOS	Transbaikalia-Mongolia	106.50	51.33	Monzonite	Khasurta	283	7.78	46.21	0.10175	0.512114		-10.2	-0.48	-6.8	1.60	Tsygankov A.A.,2014	RGG
102	B341	N. of MOS	Transbaikalia-Mongolia	106.50	51.33	Syenite	Ust'-Khilok	280	11.00	68.00	0.09780	0.512246		-7.6	-0.50	-4.1	1.38	Tsygankov A.A.,2014	RGG
103	B361-20	N. of MOS	Transbaikalia-Mongolia	106.50	51.33	Syenite	Ust'-Khilok	280	3.30	17.49	0.11407	0.512238		-7.8	-0.42	-4.9	1.44	Tsygankov A.A.,2014	RGG
104	B111	N. of MOS	Transbaikalia-Mongolia	106.50	51.33	Monzonite	Ust'-Khilok	280	6.50	36.31	0.10822	0.512263		-7.3	-0.45	-4.2	1.38	Tsygankov A.A.,2014	RGG
105	B144	N. of MOS	Transbaikalia-Mongolia	106.50	51.33	Syenite	Nadeino	270	6.62	35.95	0.11133	0.512297		-6.7	-0.43	-3.7	1.34	Tsygankov A.A.,2014	RGG
106	Xp-2	N. of MOS	Transbaikalia-Mongolia	93.92	49.33	Granitoids		220	5.51	38.00	0.08767	0.512549		-1.7	-0.55	1.3	0.89	Kovach et al., 2011	Petrology
107	PM-7-05	N. of MOS	Transbaikalia-Mongolia	93.90	49.08	Granitoids	Sharatologoi	494	3.52	14.18	0.15011	0.512823		3.6	-0.24	6.6	0.69	Kovach et al., 2011	Petrology
108	PM-21-05	N. of MOS	Transbaikalia-Mongolia	93.92	49.07	Granitoids	Sharatologoi	519	2.76	13.02	0.12826	0.512742		2.0	-0.35	6.6	0.71	Kovach et al., 2011	Petrology
109	PM-12-05	N. of MOS	Transbaikalia-Mongolia	93.92	49.07	Granitoids	Sharatologoi	519	1.83	9.52	0.11624	0.512701		1.2	-0.41	6.6	0.71	Kovach et al., 2011	Petrology
110	DI-1	N. of MOS	Tuva	96.70	51.10	Jacupirangite	Goryachegorsk	500	6.68	34.60	0.11660	0.512591		-0.9	-0.41	4.2	0.89	Vrublevskii V.V. et al..2020	IGR
111	DI-2	N. of MOS	Tuva	96.70	51.10	Ijolite	Goryachegorsk	500	6.44	33.80	0.11511	0.512626		-0.2	-0.41	5.0	0.84	Vrublevskii V.V. et al..2020	IGR
112	DF	N. of MOS	Tuva	96.70	51.10	Ijolite*	Goryachegorsk	500	3.15	14.20	0.13436	0.512759		2.4	-0.32	6.3	0.62	Vrublevskii V.V. et al..2020	IGR
113	DC	N. of MOS	Tuva	96.70	51.10	Foyaite	Goryachegorsk	500	5.88	25.20	0.14136	0.512777		2.7	-0.28	6.2	0.59	Vrublevskii V.V. et al..2020	IGR
114	BF	N. of MOS	Tuva	96.70	51.10	Carbonatite	Goryachegorsk	500	2.87	12.40	0.14020	0.512789		2.9	-0.29	6.6	0.58	Vrublevskii V.V. et al..2020	IGR
115	BJ	N. of MOS	Tuva	96.70	51.10	Foyaite	Goryachegorsk	500	2.42	11.40	0.12864	0.512581		-1.1	-0.35	3.2	0.91	Vrublevskii V.V. et al..2020	IGR
116	BC	N. of MOS	Tuva	96.70	51.10	Foyaite	Goryachegorsk	500	1.15	5.60	0.12370	0.512583		-1.1	-0.37	3.6	0.90	Vrublevskii V.V. et al..2020	IGR
117	KL	N. of MOS	Tuva	96.70	51.10	Carbonatite	Goryachegorsk	500	30.30		0.10437	0.512521		-2.3	-0.47	3.6	1.00	Vrublevskii V.V. et al..2020	IGR
118	KF-1	N. of MOS	Tuva	96.70	51.10	Ijolite	Goryachegorsk	500	2.36	10.70	0.13306	0.512587		-1.0	-0.32	3.1	0.90	Vrublevskii V.V. et al..2020	IGR
119	KF-2	N. of MOS	Tuva	96.70	51.10	Foyaite*	Goryachegorsk	500	4.18	18.20	0.13838	0.512607		-0.6	-0.30	3.1	0.86	Vrublevskii V.V. et al..2020	IGR
120	KC	N. of MOS	Tuva	96.70	51.10	Foyaite	Goryachegorsk	500	2.22	9.73	0.13767	0.512603		-0.7	-0.30	3.1	0.87	Vrublevskii V.V. et al..2020	IGR
121	CL	N. of MOS	Tuva	96.70	51.10	Carbonatite	Goryachegorsk	500	15.40	93.80	0.09932	0.512480		-3.1	-0.50	3.1	1.07	Vrublevskii V.V. et al..2020	IGR
122	CL-1	N. of MOS	Tuva	96.70	51.10	Ijolite	Goryachegorsk	500	1.55	6.45	0.14481	0.512709		1.4	-0.26	4.7	0.70	Vrublevskii V.V. et al..2020	IGR
123	110	N. of MOS	Mongolia	98.00	48.00	juvite	Goryachegorsk	426	2.78	15.30	0.10991	0.512391	7	-4.8	-0.44	-0.1	1.21	Vrublevskii V.V. et al..2020	IGR
124	6657	N. of MOS	Transbaikalia-Mongolia	99.60	48.13	Granulite	Ider	2540	4.97	25.01	0.12020	0.511369		-24.8	-0.39	-18.9	2.83	Kröner, A. et al.2015	Gondwana Research
125	6658	N. of MOS	Transbaikalia-Mongolia	99.57	48.17	Granulite	Ider	2540	4.74	27.93	0.10250	0.511042		-31.1	-0.48	-23.9	3.24	Kröner, A. et al.2015	Gondwana Research
126	7340	N. of MOS	Transbaikalia-Mongolia	99.73	48.65	Granulite	Ider	2540	3.29	16.19	0.12290	0.511313		-25.8	-0.38	-20.2	2.94	Kröner, A. et al.2015	Gondwana Research
127	7011	N. of MOS	Transbaikalia-Mongolia	99.47	48.68	Granulite	Ider	1855	0.22	2.37	0.05610	0.510532		-41.1	-0.71	-30.3	3.76	Kröner, A. et al.2015	Gondwana Research
128	6151	N. of MOS	Transbaikalia-Mongolia	99.46	48.70	Granulite	Ider	2540	3.76	20.07	0.06850	0.510570		-40.3	-0.65	-30.5	3.78	Kröner, A. et al.2015	Gondwana Research
129	6152	N. of MOS	Transbaikalia-Mongolia	99.45	48.70	Granulite	Ider	2540	2.75	22.64	0.07330	0.510647		-38.8	-0.63	-29.4	3.69	Kröner, A. et al.2015	Gondwana Research
130	6153	N. of MOS	Transbaikalia-Mongolia	99.42	48.71	Granulite	Ider	2219	1.44	10.92	0.07970	0.510698		-37.8	-0.59	-28.9	3.64	Kröner, A. et al.2015	Gondwana Research
131	2034	Within MOS	Khentey uplift	107.03	47.80	granite	Bogd Uul	208	4.13	22.55	0.110723	0.512433			–0.437	1.2	0.886	Sodnom Khishigsuren et al.	Bull. Nagoya Univ. Museum
132	2053	Within MOS	Khentey uplift	107.00	47.79	granite	Bogd Uul	208	5.24	31.22	0.101467	0.512404			–0.484	0.7	0.933	Sodnom Khishigsuren et al.	Bull. Nagoya Univ. Museum
133	2057	Within MOS	Khentey uplift	106.59	47.52	granite	Bogd Uul	206	8.97	45.31	0.119683	0.512445			–0.391	1.4	0.87	Sodnom Khishigsuren et al.	Bull. Nagoya Univ. Museum
134	2064	Within MOS	Khentey uplift	106.98	47.88	granite	Bogd Uul	206	10.36	44.66	0.140242	0.512442			–0.287	1.3	0.874	Sodnom Khishigsuren et al.	Bull. Nagoya Univ. Museum
135	MO-132	S. of MOS	Transbaikalia-Mongolia	106.30	44.97	Rhyolite	Olzit	208	28.10	148	0.11510	0.512552		-1.7	-0.41	0.5	0.95	Zhu Mingshuai et al.,2016	JAES
136	MO-142	S. of MOS	Transbaikalia-Mongolia	106.30	44.97	Rhyolite	Olzit	208	35.40	182.8	0.11730	0.512562		-1.5	-0.40	0.6	0.94	Zhu Mingshuai et al.,2016	JAES
137	MO-156	S. of MOS	Transbaikalia-Mongolia	106.30	44.97	Rhyolite	Olzit	208	28.20	143.6	0.11900	0.512603		-0.7	-0.40	1.4	0.87	Zhu Mingshuai et al.,2016	JAES
138	MO-158	S. of MOS	Transbaikalia-Mongolia	106.30	44.97	Rhyolite	Olzit	208	27.90	138.7	0.12190	0.512602		-0.7	-0.38	1.3	0.88	Zhu Mingshuai et al.,2016	JAES
139	7580	S. of MOS	Transbaikalia-Mongolia	101.53	45.73	Granitoid	Tatsaingol	603	6.32	29.90	0.1276	0.512330	5	-6.0	-0.35	-0.7	1.36	Kozakov I. K. et al.,2015.	Petrology
140	7580—2	S. of MOS	Transbaikalia-Mongolia	101.53	45.73	Granitoids	Tatsaingol	603	6.75	31.60	0.1292	0.512345	5	-5.7	-0.34	-0.5	1.35	Kozakov I. K. et al.,2015.	Petrology

No.	Sample	Tectonic unit	Location and unit	Longitude	Latitude	Lithology	Pluton	Age	Sm	Nd	$^{147}Sm/^{144}Nd$	$^{143}Nd/^{144}Nd$	2σ	εNd(0)	$f_{Sm/Nd}$	εNd(t)	T_{DM2} (Ga)	References	
141	7580—3	S. of MOS	Transbaikalia-Mongolia	101.53	45.73	Granitoid	Tatsaingol	603	4.70	20.10	0.1415	0.512381	2	-5.0	-0.28	-0.8	1.37	Kozakov I. K. et al.,2015.	Petrology
142	7291—1	S. of MOS	Transbaikalia-Mongolia	101.53	45.73	Granitoids	Tatsaingol	603	4.40	22.00	0.1206	0.512229	2	-8.0	-0.39	-2.1	1.48	Kozakov I. K. et al.,2015.	Petrology
143	7291	S. of MOS	Transbaikalia-Mongolia	101.53	45.73	Granitoids	Tatsaingol	603	3.41	13.84	0.1490	0.512436	4	-3.9	-0.24	-0.3	1.33	Kozakov I. K. et al.,2015.	Petrology
144	6180	S. of MOS	Transbaikalia-Mongolia	101.53	45.73	Granitoids	Tatsaingol	603	10.46	57.00	0.1109	0.512238	1	-7.8	-0.44	-1.2	1.41	Kozakov I. K. et al.,2015.	Petrology
145	6184	S. of MOS	Transbaikalia-Mongolia	101.53	45.73	Granitoids	Tatsaingol	603	6.47	37.00	0.1056	0.512271	2	-7.2	-0.46	-0.1	1.32	Kozakov I. K. et al.,2015.	Petrology
146	7562	S. of MOS	Transbaikalia-Mongolia	101.53	45.90	Granitoids	Tatsaingol	570	3.64	19.96	0.1103	0.512085	3	-10.8	-0.44	-4.5	1.65	Kozakov I. K. et al.,2015.	Petrology
147	6176	S. of MOS	Transbaikalia-Mongolia	101.70	45.75	Granitoids	Tatsaingol	565	2.84	9.68	0.1773	0.512022	4	-12.0	-0.10	-10.6	2.12	Kozakov I. K. et al.,2015.	Petrology
148	6124	S. of MOS	Transbaikalia-Mongolia	101.40	45.77	Granitoids	Shargyn Gol R.	565	2.70	16.96	0.0962	0.511870	4	-15.0	-0.51	-7.7	1.91	Kozakov I. K. et al.,2015.	Petrology
149	5967	S. of MOS	Transbaikalia-Mongolia	101.40	45.77	Granitoids	Shargyn Gol R.	562	0.93	5.69	0.0988	0.511962	11	-13.2	-0.50	-6.2	1.78	Kozakov I. K. et al.,2015.	Petrology
150	6035	S. of MOS	Transbaikalia-Mongolia	101.40	45.77	Granitoids	Shargyn Gol R.	565	5.23	31.30	0.1008	0.512209	2	-8.4	-0.49	-1.5	1.40	Kozakov I. K. et al.,2015.	Petrology
151	6180—1	S. of MOS	Transbaikalia-Mongolia	101.40	45.77	Granitoids	Shargyn Gol R.	565	1.76	11.13	0.0954	0.512131	7	-9.9	-0.51	-2.6	1.49	Kozakov I. K. et al.,2015.	Petrology
152	6425	S. of MOS	Transbaikalia-Mongolia	101.40	45.67	Granitoid	Shargyn Gol R.	561	3.58	20.60	0.1052	0.511826	3	-15.8	-0.47	-9.3	2.03	Kozakov I. K. et al.,2015.	Petrology
153	6194	S. of MOS	Transbaikalia-Mongolia	101.40	45.77	Granitoid	Shargyn Gol R.	552	5.52	31.50	0.1059	0.512050	5	-11.5	-0.46	-5.1	1.68	Kozakov I. K. et al.,2015.	Petrology
154	5975	S. of MOS	Transbaikalia-Mongolia	101.40	45.77	Granitoid	Shargyn Gol R.	547	3.73	35.70	0.0632	0.512068	1	-11.1	-0.68	-1.8	1.41	Kozakov I. K. et al.,2015.	Petrology
155	6201—1	S. of MOS	Transbaikalia-Mongolia	101.40	45.77	Granitoid	Shargyn Gol R.	547	8.09	60.00	0.0814	0.512000	2	-12.4	-0.59	-4.4	1.62	Kozakov I. K. et al.,2015.	Petrology
156	6036	S. of MOS	Transbaikalia-Mongolia	101.40	45.77	Granitoid	Shargyn Gol R.	547	5.21	30.10	0.1047	0.512164	2	-9.2	-0.47	-2.8	1.49	Kozakov I. K. et al.,2015.	Petrology
157	6036—1	S. of MOS	Transbaikalia-Mongolia	101.40	45.77	Granitoid	Shargyn Gol R.	547	8.18	38.00	0.1300	0.512321	2	-6.2	-0.34	-1.5	1.39	Kozakov I. K. et al.,2015.	Petrology
158	6201	S. of MOS	Transbaikalia-Mongolia	101.40	45.77	Granitoid	Shargyn Gol R.	547	6.66	39.20	0.1026	0.512247	3	-7.6	-0.48	-1.1	1.35	Kozakov I. K. et al.,2015.	Petrology
159	6201—2	S. of MOS	Transbaikalia-Mongolia	101.40	45.77	Granitoid	Shargyn Gol R.	547	7.93	39.70	0.1207	0.512182	2	-8.9	-0.39	-3.6	1.55	Kozakov I. K. et al.,2015.	Petrology
160	6006	S. of MOS	Transbaikalia-Mongolia	101.40	45.71	Granitoid	Shargyn Gol R.	546	4.49	26.00	0.1042	0.511998	5	-12.5	-0.47	-6.0	1.75	Kozakov I. K. et al.,2015.	Petrology
161	6427	S. of MOS	Transbaikalia-Mongolia	101.38	45.71	Granitoid	Bayan Teg	545	8.65	47.30	0.1106	0.511807	3	-16.2	-0.44	-10.2	2.09	Kozakov I. K. et al.,2015.	Petrology
162	6197	S. of MOS	Transbaikalia-Mongolia	101.38	45.71	Granitoid	Bayan Teg	545	3.01	18.19	0.1000	0.511801	2	-16.3	-0.49	-9.6	2.04	Kozakov I. K. et al.,2015.	Petrology
163	5965	S. of MOS	Transbaikalia-Mongolia	101.38	45.71	Granitoid	Bayan Teg	545	10.27	52.70	0.1178	0.511819	4	-16.0	-0.40	-10.5	2.11	Kozakov I. K. et al.,2015.	Petrology
164	6422	S. of MOS	Transbaikalia-Mongolia	101.38	45.71	Granitoid	Bayan Teg	545	0.93	6.76	0.0828	0.512144	4	-9.6	-0.58	-1.7	1.40	Kozakov I. K. et al.,2015.	Petrology
165	12ER36-2	S. of MOS	Erguna Massif	120.50	51.50	Monzogranite	Moerdaoga	455			0.1248	0.512314	8	-6.3	-0.37	-2.1	1.33	Zhao Suo et al., .2017	PHD diss., JU (ICWEA)
166	12ER36-3	S. of MOS	Erguna Massif	120.50	51.50	Monzogranite	Moerdaoga	455			0.1310	0.512332	3	-6.0	-0.33	-2.2	1.30	Zhao Suo et al., .2017	PHD diss., JU (ICWEA)
167	ER7-2	S. of MOS	Erguna Massif	120.87	51.60	Syenogranite	Moerdaoga	851			0.1265	0.512202	5	-8.5	-0.36	-3.1	1.51	Zhao Suo et al., .2017	PHD diss., JU (ICWEA)
168	ER2M	S. of MOS	Erguna Massif	120.50	51.50	Syenogranite	Moerdaoga	851			0.1151	0.512119	2	-10.1	-0.41	-3.9	1.64	Zhao Suo et al., .2017	PHD diss., JU (ICWEA)
169	11ER23-3	S. of MOS	Erguna Massif	120.50	51.50	Syenogranite	Moerdaoga	792			0.1237	0.512166	7	-9.2	-0.37	-3.6	1.56	Zhao Suo et al., .2017	PHD diss., JU (ICWEA)
170	11ER23-4	S. of MOS	Erguna Massif	120.50	51.50	Syenogranite	Moerdaoga	792			0.1200	0.512155	3	-9.4	-0.39	-3.5	1.58	Zhao Suo et al., .2017	PHD diss., JU (ICWEA)
171	12ER14-2	S. of MOS	Erguna Massif	120.50	51.50	Monzogranite	Moerdaoga	762			0.0933	0.512102	2	-10.5	-0.53	-2.5	1.67	Zhao Suo et al., .2017	PHD diss., JU (ICWEA)
172	12ER14-3	S. of MOS	Erguna Massif	120.50	51.50	Monzogranite	Moerdaoga	762			0.0896	0.512039	2	-11.7	-0.54	-3.5	1.77	Zhao Suo et al., .2017	PHD diss., JU (ICWEA)
173	ER13-1	S. of MOS	Erguna Massif	120.50	51.50	Syenogranite	Moerdaoga	737			0.1249	0.512198	3	-8.6	-0.37	-3.1	1.51	Zhao Suo et al., .2017	PHD diss., JU (ICWEA)
174	ER23-5	S. of MOS	Erguna Massif	120.50	51.50	Syenogranite	Moerdaoga	737			0.1275	0.512236	4	-7.8	-0.35	-2.5	1.45	Zhao Suo et al., .2017	PHD diss., JU (ICWEA)
175	PM4TC-07	S. of MOS	Erguna Massif	124.93	51.83	Gneissic granite	Wuleshan	915				0.512657		0.4	-1.00	15.4	0.79	Yang Huaben et al.,2017	GBC
176	11ER16-1	S. of MOS	Erguna Massif	119.68	50.98	Granodiorite	Eredenet	246			0.12177	0.512342	1	-5.8	-0.38	-3.4	1.29	Tang. J.. et al., 2014	Lithos
177	11ER16-3	S. of MOS	Erguna Massif	119.68	50.98	Granodiorite	Eredenet	246			0.11053	0.512304	3	-6.5	-0.44	-3.8	1.35	Tang. J.. et al., 2014	Lithos
178	12ER11-1	S. of MOS	Erguna Massif	120.03	51.19	Monzogranite	Eredenet	247			0.09778	0.512239	4	-7.8	-0.50	-4.7	1.45	Tang. J.. et al., 2014	Lithos
179	12ER11-2	S. of MOS	Erguna Massif	120.03	51.19	Monzogranite	Eredenet	247			0.10998	0.512258	4	-7.4	-0.44	-4.7	1.42	Tang. J.. et al., 2014	Lithos
180	14BDG-11	S. of MOS	NE China	119.13	49.98	Granodioritic porphyry	Badaoka	230	3.80	19.50	0.11767	0.512540	7	-1.9	-0.40	0.4	0.97	Mi. K.. et al., 2017	OGR
181	14BDG-36	S. of MOS	NE China	119.13	49.98	Granodioritic porphyry	Badaoka	230	4.60	22.20	0.12666	0.512541	7	-1.9	-0.36	0.2	0.97	Mi. K.. et al., 2017	OGR
182	14BDG-80	S. of MOS	NE China	119.14	49.98	Granodioritic porphyry	Badaoka	230	4.60	24.30	0.11446	0.512574	7	-1.2	-0.42	1.2	0.92	Mi. K.. et al. 2017	OGR
183	ZKI-10301-3	S. of MOS	NE China	116.71	49.46	Qtz monzonite	Halasheng	128.2	10.30	59.40	0.11028	0.512591		-0.9	-0.44	0.5	0.89	Han. R. et al., 2020	OGR
184	ZKIV2703-66	S. of MOS	NE China	116.69	49.47	Qtz monzonite	Halasheng	131.7	10.40	59.10	0.11181	0.512596		-0.8	-0.43	0.6	0.88	Han. R. et al., 2020	OGR
185	ZKIV901-486	S. of MOS	NE China	116.69	49.47	Qtz monzonite	Halasheng	130.8	10.30	57.90	0.11310	0.512588		-1.0	-0.43	0.4	0.90	Han. R. et al., 2020	OGR
186	H0486	S. of MOS	Mongolia	98.49	45.38	Orthogneiss	Baydrag	540	4.50	35.30	0.07710	0.512345		-5.7	-0.61	2.5	1.28	Buriánek, D. et al., 2017	Gondwana Research
187	M13712-6.1	S. of MOS	Mongolia	114.33	47.79	Andsite	E.Mongolia	130	5.93	35.98	0.09958	0.512599	5	-0.8	-0.49	0.9	0.88	This study	
188	M13713-10.1	S. of MOS	Mongolia	113.69	47.13	Monzogranite	E.Mongolia	243	3.72	30.89	0.07287	0.512491	6	-2.9	-0.63	1.0	1.05	This study	
189	M13713-9.1	S. of MOS	Mongolia	114.03	47.18	Syenogranite	E.Mongolia	238	1.20	7.23	0.10047	0.512533		-2.1	-0.49	0.9	0.98	This study	
190	M13713-9.2	S. of MOS	Mongolia	114.03	47.18	Syenogranite	E.Mongolia	238	1.35	7.77	0.10486	0.512546	7	-1.8	-0.47	1.0	0.96	This study	
191	M13722-10.1	S. of MOS	Mongolia	110.68	45.83	Bi granite	E.Mongolia	125	3.76	20.59	0.11052	0.512724	6	1.7	-0.44	3.1	0.68	This study	
192	M13722-10.2	S. of MOS	Mongolia	110.68	45.83	Bi granite	E.Mongolia	125	3.67	19.42	0.11422	0.512720	8	1.6	-0.42	2.9	0.69	This study	
193	M13723-33.1	S. of MOS	Mongolia	110.66	45.81	Bi granite	E.Mongolia	141	3.42	19.38	0.10660	0.512729		1.8	-0.46	3.4	0.67	This study	
194	M13723-33.2	S. of MOS	Mongolia	110.66	45.81	Bi granite	E.Mongolia	141	3.38	18.95	0.10795	0.512722	4	1.6	-0.45	3.2	0.68	This study	
195	M13723-8.2	S. of MOS	Mongolia	110.70	45.81	Gneissic granodiorite	E.Mongolia	130	1.02	6.37	0.09704	0.512646	3	0.2	-0.51	1.8	0.80	This study	
196	M13723-8.1	S. of MOS	Mongolia	110.70	45.81	Gneissic granodiorite	E.Mongolia	130	0.93	5.63	0.09964	0.512651	4	0.3	-0.49	1.9	0.80	This study	
197	M1494-14.2	S. of MOS	Mongolia	109.38	45.23	Gnessic granite	Khatanblage	905	7.80	36.91	0.12850	0.511908	9	-14.2	-0.35	-9.0	1.97	This study	
198	M1494-14.1	S. of MOS	Mongolia	109.38	45.23	Gnessic granite	Khatanblage	905	5.63	28.70	0.11858	0.511928	8	-13.8	-0.40	-7.9	1.94	This study	
199	M90722-3A	S. of MOS	Mongolia	96.25	46.28	Granite	Mongolia Altai	420	0.30	1.00	0.18140	0.512913	1	5.4	-0.08	6.2	0.38	This study	
200	M90722-3E	S. of MOS	Mongolia	96.25	46.28	Granite	Mongolia Altai	420	0.35	1.10	0.19240	0.512869	1	4.5	-0.02	4.7	0.44	This study	
201	0075-3	S. of MOS	Great Xing'an	124.32	51.61	Granodiorite	Xinlinzhen	130	2.86	17.40	0.10000	0.512598	12	-0.8	-0.49	0.8	0.86	Zhang YL et al., 2008	JJU (ICWEA)
202	0075-4	S. of MOS	Great Xing'an	124.32	51.61	Granodiorite	Xinlinzhen	130	1.58	10.40	0.09000	0.512549	13	-1.7	-0.54	0.0	0.92	Zhang YL et al., 2008	JJU (ICWEA)
203	0076-2	S. of MOS	Great Xing'an	124.16	51.63	Granodiorite	Xinlinzhen	130	2.58	16.80	0.09000	0.512541	10	-1.9	-0.54	-0.1	0.93	Zhang YL et al., 2008	JJU (ICWEA)
204	0076-9	S. of MOS	Great Xing'an	124.16	51.63	Granodiorite	Xinlinzhen	130	2.86	16.00	0.11000	0.512553	14	-1.7	-0.44	-0.2	0.94	Zhang YL et al., 2008	JJU (ICWEA)
205	ML-1	S. of MOS	Great Xing'an	121.85	53.37	Granite-porphyry	Luogu River	130	5.25	28.21	0.11270	0.512400	10	-4.6	-0.43	-3.2	1.19	Wu G. et al., 2009	APS (ICWEA)
206	ML-16	S. of MOS	Great Xing'an	121.85	53.37	Granite-porphyry	Luogu River	130	6.42	41.89	0.09267	0.512373	6	-5.2	-0.53	-3.4	1.20	Wu G. et al., 2009	APS (ICWEA)
207	ML-3	S. of MOS	Great Xing'an	121.85	53.37	Granite-porphyry	Luogu River	130	6.34	37.52	0.10220	0.512400	7	-4.6	-0.48	-3.1	1.17	Wu G. et al., 2009	APS (ICWEA)
208	ML-34	S. of MOS	Great Xing'an	121.85	53.37	Qtz-monzonite porphyry	Luogu River	130	6.65	39.65	0.10140	0.512422	10	-4.2	-0.48	-2.6	1.14	Wu G. et al., 2009	APS (ICWEA)
209	ML-37	S. of MOS	Great Xing'an	121.85	53.37	Qtz-monzonite porphyry	Luogu River	130	7.04	43.18	0.09867	0.512419	5	-4.3	-0.50	-2.6	1.14	Wu G. et al., 2009	APS (ICWEA)
210	11ER16-1	S. of MOS	Erguna Massif	119.68	50.98	Granodiorites	Jiuka	246			0.12177	0.512342	10	-5.8	-0.38	-3.4	1.30	Tang J et al., 2014	Lithos
211	11ER16-3	S. of MOS	Erguna Massif	119.66	50.95	Granodiorites	Jiuka	246			0.11053	0.512304	3	-6.5	-0.44	-3.8	1.33	Tang J et al., 2014	Lithos

No.	Sample	Tectonic unit	Location and unit	Longitude	Latitude	Lithology	Pluton	Age	Sm	Nd	$^{147}Sm/^{144}Nd$	$^{143}Nd/^{144}Nd$	2σ	εNd(0)	$f_{Sm/Nd}$	εNd(t)	T_{DM2} (Ga)	References	
212	12ER11-1	S. of MOS	Erguna Massif	120.03	51.19	Monzogranites	Shiwei	247			0.09778	0.512239	4	-7.8	-0.50	-4.7	1.40	Tang J et al., 2014	Lithos
213	12ER11-2	S. of MOS	Erguna Massif	120.05	51.18	Monzogranites	Shiwei	247			0.10998	0.512258	4	-7.4	-0.44	-4.7	1.40	Tang J et al., 2014	Lithos
214	T-13	S. of MOS	Inner Mongolia	113.33	46.79	Bi granite	Tumueryanaobao	240	4.08	33.90	0.0728	0.512500	6	-2.7	-0.63	1.1	0.92	Jiang SH et al., 2010	AGS (ICWEA)
215	T-14	S. of MOS	Inner Mongolia	113.33	46.79	Bi granite	Tumueryanaobao	240	2.33	18.70	0.0754	0.512491	4	-2.9	-0.62	0.8	0.94	Jiang SH et al., 2010	AGS (ICWEA)
216	T-16	S. of MOS	Inner Mongolia	113.33	46.79	Bi granite	Tumueryanaobao	240	2.71	21.50	0.0763	0.512494	7	-2.8	-0.61	0.9	0.94	Jiang SH et al., 2010	AGS (ICWEA)
217	T-17-19	S. of MOS	Great Xing'an	113.33	46.79	Bi granite	Tumueryanaobao	240	3.63	27.20	0.0806	0.512529	14	-2.1	-0.59	1.4	0.90	Jiang SH et al., 2010	AGS (ICWEA)
218	T-17-2	S. of MOS	Great Xing'an	113.33	46.79	Bi granite	Tumueryanaobao	240	2.52	20.70	0.0738	0.512501	7	-2.7	-0.62	1.1	0.92	Jiang SH et al., 2010	AGS (ICWEA)
219	T-17-8	S. of MOS	Great Xing'an	113.33	46.79	Bi granite	Tumueryanaobao	240	3.03	18.50	0.0988	0.512573	7	-1.3	-0.50	1.7	0.87	Jiang SH et al., 2010	AGS (ICWEA)
220	HH2704-12	S. of MOS	Great Xing'an	119.98	48.29	Qtz monzonite	Honghuaerji	182	5.10	23.90	0.1290	0.512651	5	0.3	-0.34	1.8	0.82	Guo ZJ et al., 2014	GC (ICWEA)
221	HH4309-146	S. of MOS	Great Xing'an	119.98	48.29	Qtz monzonite	Honghuaerji	185	5.17	29.40	0.1064	0.512684	3	0.9	-0.46	3.0	0.72	Guo ZJ et al., 2014	GC (ICWEA)
222	HH4309-157	S. of MOS	Great Xing'an	119.99	48.29	Qtz monzonite	Honghuaerji	179	5.83	29.20	0.1205	0.512659	2	0.4	-0.39	2.2	0.79	Guo ZJ et al., 2014	GC (ICWEA)
223	HH4309-158	S. of MOS	Great Xing'an	119.99	48.29	Qtz monzonite	Honghuaerji	180	5.32	27.60	0.1167	0.512648	3	0.2	-0.41	2.0	0.80	Guo ZJ et al., 2014	GC (ICWEA)
224	HH4318-01	S. of MOS	Great Xing'an	119.98	48.29	Qtz monzonite	Honghuaerji	183	4.07	18.20	0.1352	0.512703	2	1.3	-0.31	2.7	0.75	Guo ZJ et al., 2014	GC (ICWEA)
225	HH5503-01	S. of MOS	Great Xing'an	119.99	48.29	Qtz monzonite	Honghuaerji	184	5.34	29.30	0.1104	0.512694	4	1.1	-0.44	3.1	0.71	Guo ZJ et al., 2014	GC (ICWEA)
226	HH5503-32	S. of MOS	Great Xing'an	119.99	48.29	Qtz monzonite	Honghuaerji	181	4.30	19.30	0.1349	0.512692	2	1.1	-0.31	2.5	0.76	Guo ZJ et al., 2014	GC (ICWEA)
227	WS-1	S. of MOS	Great Xing'an	117.30	49.42	Porphyry	Wunugetushan	179			0.08848	0.512559	12	-1.5	-0.55	0.9	0.89	Chen ZG et al., 2011	OGR
228	WS-2	S. of MOS	Great Xing'an	117.30	49.42	Porphyry	Wunugetushan	179			0.08997	0.512552	14	-1.7	-0.54	0.8	0.90	Chen ZG et al., 2011	OGR
229	WS-3	S. of MOS	Great Xing'an	117.30	49.42	Porphyry	Wunugetushan	179			0.10319	0.512577	13	-1.2	-0.48	0.9	0.89	Chen ZG et al., 2011	OGR
230	WS-4	S. of MOS	Great Xing'an	117.30	49.42	Porphyry	Wunugetushan	179			0.08782	0.512561	10	-1.5	-0.55	1.0	0.88	Chen ZG et al., 2011	OGR
231	WS-5	S. of MOS	Great Xing'an	117.30	49.42	Porphyry	Wunugetushan	179			0.09169	0.512531	12	-2.1	-0.53	0.3	0.94	Chen ZG et al., 2011	OGR
232	WS-9	S. of MOS	Great Xing'an	117.30	49.42	Porphyry	Wunugetushan	179	2.37	13.65	0.10500	0.512586	12	-1.0	-0.47	1.1	0.87	Chen ZG et al., 2011	OGR
233	TPC07-1	S. of MOS	Great Xing'an	120.43	51.47	Porphyry	Taipingchuan	202	3.36	17.14	0.11860	0.512361	13	-5.4	-0.40	-3.4	1.26	Chen ZG et al., 2010	APS (ICWEA)
234	TPC07-2	S. of MOS	Great Xing'an	120.43	51.47	Porphyry	Taipingchuan	202	4.11	23.09	0.10780	0.512334	12	-5.9	-0.45	-3.6	1.28	Chen ZG et al., 2010	APS (ICWEA)
235	TPC07-3	S. of MOS	Great Xing'an	120.43	51.47	Porphyry	Taipingchuan	202	4.07	20.04	0.12290	0.512361	14	-5.4	-0.38	-3.5	1.27	Chen ZG et al., 2010	APS (ICWEA)
236	TPC07-4	S. of MOS	Great Xing'an	120.43	51.47	Porphyry	Taipingchuan	202	4.20	24.50	0.10380	0.512318	14	-6.2	-0.47	-3.8	1.29	Chen ZG et al., 2010	APS (ICWEA)
237	TPC07-5	S. of MOS	Great Xing'an	120.43	51.47	Porphyry	Taipingchuan	202	4.29	23.51	0.11040	0.512351	12	-5.6	-0.44	-3.4	1.26	Chen ZG et al., 2010	APS (ICWEA)
238	TPC07-6	S. of MOS	Great Xing'an	120.43	51.47	Porphyry	Taipingchuan	202	4.40	25.21	0.10570	0.512334	12	-5.9	-0.46	-3.6	1.27	Chen ZG et al., 2010	APS (ICWEA)
239	TPC07-7	S. of MOS	Great Xing'an	120.43	51.47	Porphyry	Taipingchuan	202	3.46	18.41	0.11370	0.512328	13	-6.0	-0.42	-3.9	1.30	Chen ZG et al., 2010	APS (ICWEA)
240	TPC07-8	S. of MOS	Great Xing'an	120.43	51.47	Porphyry	Taipingchuan	202	4.63	27.00	0.10390	0.512331	11	-6.0	-0.47	-3.6	1.27	Chen ZG et al., 2010	APS (ICWEA)
241		S. of MOS	Great Xing'an	117.92	51.33	Granite porphyry	Shakhtama	155	5.23	32.38	0.09766	0.512645	16	0.1	-0.50	2.1	0.77	Berzina AP et al., 2014	JAES
242		S. of MOS	Great Xing'an	117.92	51.33	Granite	Shakhtama	159	5.59	38.10	0.08874	0.512386	14	-4.9	-0.55	-2.7	1.17	Berzina AP et al., 2014	JAES
243		S. of MOS	Great Xing'an	117.92	51.33	Monzonite porphyry	Shakhtama	159	5.97	35.49	0.10166	0.512643	17	0.1	-0.48	2.0	0.78	Berzina AP et al., 2014	JAES
244		S. of MOS	Great Xing'an	117.92	51.33	Monzonite	Shakhtama	160	4.41	24.65	0.10815	0.512489	16	-2.9	-0.45	-1.1	1.04	Berzina AP et al., 2014	JAES
245	CLK-011	S. of MOS	Great Xing'an	123.95	51.21	Biotite monzogranite	Chalukou	163	3.10	20.30	0.0908	0.512350		-5.6	-0.54	-3.4	1.23	Li ZZ. et al., 2014	Lithos
246	W-1	S. of MOS	Great Xing'an	125.65	52.56	Granite	Woduhe	130	3.02	6.68	0.2737	0.512706	6	1.3	0.39	0.0	0.93	Jahn BM et al., 2001	Lithos
247	W-2	S. of MOS	Great Xing'an	125.65	52.56	Granite	Woduhe	130	3.57	7.46	0.2893	0.512719	6	1.6	0.47	0.0	0.93	Jahn BM et al., 2001	Lithos
248	W-3	S. of MOS	Great Xing'an	125.65	52.56	Granite	Woduhe	130	2.90	6.47	0.2710	0.512703	5	1.3	0.38	0.0	0.93	Jahn BM et al., 2001	Lithos
249	W-4	S. of MOS	Great Xing'an	125.65	52.56	Granite	Woduhe	130	3.06	6.46	0.2860	0.512694	6	1.1	0.45	-0.4	0.96	Jahn BM et al., 2001	Lithos
250	W-5	S. of MOS	Great Xing'an	125.65	52.56	Granite	Woduhe	130	3.71	8.11	0.2762	0.512702	6	1.2	0.40	-0.1	0.94	Jahn BM et al., 2001	Lithos
251	W-6	S. of MOS	Great Xing'an	125.65	52.56	Granite	Woduhe	130	4.19	9.35	0.2708	0.512706	5	1.3	0.38	0.1	0.92	Jahn BM et al., 2001	Lithos
252	W-7	S. of MOS	Great Xing'an	125.65	52.56	Granite	Woduhe	130	4.51	10.37	0.2627	0.512692	5	1.1	0.34	0.0	0.93	Jahn BM et al., 2001	Lithos
253	1102-521	S. of MOS	Great Xing'an	124.00	51.19	Granite porphyry	Chalukou	147			0.1058	0.512598		-0.8	-0.46	0.9	0.86	Li ZZ et al., 2014	Lithos
254	1103-1165.5	S. of MOS	Great Xing'an	124.00	51.19	Aplite porphyry	Chalukou	148			0.1020	0.512551		-1.7	-0.48	0.1	0.93	Li ZZ et al., 2014	Lithos
255	1103-886	S. of MOS	Great Xing'an	124.00	51.19	Granite porphyry	Chalukou	147			0.1471	0.512561		-1.5	-0.25	-0.6	0.98	Li ZZ et al., 2014	Lithos
256	1104-205.7	S. of MOS	Great Xing'an	124.00	51.19	Granite porphyry	Chalukou	147			0.1295	0.512549		-1.7	-0.34	-0.5	0.97	Li ZZ et al., 2014	Lithos
257	1111-1116	S. of MOS	Great Xing'an	124.00	51.19	Aplite porphyry	Chalukou	148			0.1284	0.512591		-0.9	-0.35	0.4	0.91	Li ZZ et al., 2014	Lithos
258	1702-180	S. of MOS	Great Xing'an	124.00	51.19	Granite porphyry	Chalukou	147			0.1391	0.512616		-0.4	-0.29	0.7	0.88	Li ZZ et al., 2014	Lithos
259	1703-383	S. of MOS	Great Xing'an	124.00	51.19	Granite porphyry	Chalukou	147			0.2380	0.512665		0.5	0.21	-0.2	0.97	Li ZZ et al., 2014	Lithos
260	1708-361.7	S. of MOS	Great Xing'an	124.00	51.19	Feldspar porphyry	Chalukou	141			0.0977	0.512551		-1.7	-0.50	0.1	0.92	Li ZZ et al., 2014	Lithos
261	1902-674.1	S. of MOS	Great Xing'an	124.00	51.19	Aplite porphyry	Chalukou	148			0.1147	0.512577		-1.2	-0.42	0.4	0.91	Li ZZ et al., 2014	Lithos
262	804-228	S. of MOS	Great Xing'an	124.00	51.19	Granite porphyry	Chalukou	147			0.1175	0.512553		-1.7	-0.40	-0.2	0.95	Li ZZ et al., 2014	Lithos
263	902-1196	S. of MOS	Great Xing'an	124.00	51.19	Aplite porphyry	Chalukou	148			0.1213	0.512580		-1.1	-0.38	0.3	0.91	Li ZZ et al., 2014	Lithos
264	903-1273.3	S. of MOS	Great Xing'an	124.00	51.19	Feldspar porphyry	Chalukou	128			0.1000	0.512581		-1.1	-0.49	0.5	0.88	Li ZZ et al., 2014	Lithos
265	CL-02	S. of MOS	Great Xing'an	124.00	51.19	Monzogranite	Chalukou	172			0.0984	0.512461		-3.5	-0.50	-1.3	1.06	Li ZZ et al., 2014	Lithos
266	D2032	S. of MOS	Great Xing'an	122.88	49.41	Qtz monzonite	Liufangjian	172	1.62	9.07	0.1083	0.512579	9	-1.2	-0.45	0.8	0.89	Li H, 2012	CUG(ICWEA)
267	PM14LT111	S. of MOS	Great Xing'an	123.05	49.40	Qtz monzonite	Xieniqi	161	3.21	21.14	0.0917	0.512627	6	-0.2	-0.53	1.9	0.79	Li H, 2012	CUG(ICWEA)
268	PM14LT151	S. of MOS	Great Xing'an	123.05	49.40	Qtz monzonite	Xieniqi	161	6.90	34.37	0.1214	0.512626	8	-0.2	-0.38	1.3	0.84	Li H, 2012	CUG(ICWEA)
269	PM5LT124a	S. of MOS	Great Xing'an	122.88	49.41	Orthoclase granite	Liufangjian	172	2.24	14.53	0.0931	0.512506	7	-2.6	-0.53	-0.3	0.98	Li H, 2012	CUG(ICWEA)
270	GW05064	S. of MOS	Great Xing'an	126.17	52.50	Hb granodiorite	Zhengqicun	190	5.28	26.14	0.12208	0.512249	13	-7.6	-0.38	-5.8	1.44	Sui and Xu, 2010	GC (ICWEA)
271	GW05067	S. of MOS	Great Xing'an	126.17	52.50	Hb granodiorite	Zhengqicun	190	4.18	22.59	0.11196	0.512222	13	-8.1	-0.43	-6.1	1.46	Sui and Xu, 2010	GC (ICWEA)
272	GW05085	S. of MOS	Great Xing'an	126.17	52.08	Bi monzonite-granite	Xinghua-Fanshentun	178	3.68	22.07	0.10066	0.512253	14	-7.5	-0.49	-5.3	1.40	Sui and Xu, 2010	GC (ICWEA)
273	GW05088	S. of MOS	Great Xing'an	126.17	52.08	Bi monzonite-granite	Xinghua-Fanshentun	178	3.75	22.94	0.09877	0.512398	11	-4.7	-0.50	-2.5	1.16	Sui and Xu, 2010	GC (ICWEA)
274	GW05099	S. of MOS	Great Xing'an	125.33	52.03	Hb Qtz diorite	Hanjiayuanzi	188	12.49	60.12	0.12559	0.512362	8	-5.4	-0.36	-3.7	1.27	Sui and Xu, 2010	GC (ICWEA)
275	GW05101	S. of MOS	Great Xing'an	125.33	52.03	Hb Qtz diorite	Hanjiayuanzi	188	4.92	30.08	0.09877	0.512258	10	-7.4	-0.50	-5.1	1.38	Sui and Xu, 2010	GC (ICWEA)
276	GW05110	S. of MOS	Great Xing'an	125.33	52.00	Qtz monzonite	Wudingshan	188	4.50	25.17	0.10798	0.512031	10	-11.8	-0.45	-9.7	1.76	Sui and Xu, 2010	GC (ICWEA)
277	GW05112	S. of MOS	Great Xing'an	126.25	51.87	Porphyritic adamellite	Jiweidianzi	181	2.68	19.98	0.08105	0.512218	11	-8.2	-0.59	-5.5	1.41	Sui and Xu, 2010	GC (ICWEA)
278	GW05120	S. of MOS	Great Xing'an	126.25	51.87	Bi monzonite-granite	Jiweidianzi	176	5.62	35.95	0.09445	0.512215	12	-8.3	-0.52	-6.0	1.45	Sui and Xu, 2010	GC (ICWEA)
279	9436-3	S. of MOS	Great Xing'an	121.79	48.79	Tonalite	Boketou	156	5.07	710.6	0.1226	0.512587	8	-1.0	-0.38	0.5	0.90	Wu FY et al., 2003	Lithos
280	9485-3	S. of MOS	Great Xing'an	123.47	50.65	Moyite	Alihe	120	3.94	45.23	0.1249	0.512712	10	1.4	-0.37	2.5	0.71	Wu FY et al., 2003	Lithos
281	9825-12	S. of MOS	Great Xing'an	124.93	52.49	Diorote	Tafeng	430	5.21	38.36	0.0821	0.512233	8	-7.9	-0.58	-1.6	1.30	Wu FY et al., 2003	Lithos
282	9825-13	S. of MOS	Great Xing'an	124.93	52.49	Qtz diorote	Tafeng	430	5.46	28.73	0.1149	0.512227	12	-8.0	-0.42	-3.5	1.45	Wu FY et al., 2003	Lithos

No.	Sample	Tectonic unit	Location and unit	Longitude	Latitude	Lithology	Pluton	Age	Sm	Nd	$^{147}Sm/^{144}Nd$	$^{143}Nd/^{144}Nd$	2σ	εNd(0)	$f_{Sm/Nd}$	εNd(t)	T_{DM2} (Ga)	References	
283	9825-14	S. of MOS	Great Xing'an	124.93	52.49	Qtz diorote	Tafeng	430	4.20	22.72	0.1117	0.512227	12	-8.0	-0.43	-3.4	1.44	Wu FY et al., 2003	Lithos
284	9825-4	S. of MOS	Great Xing'an	124.93	52.49	Diorote	Tafeng	430	3.53	16.26	0.1311	0.512240	9	-7.8	-0.33	-4.2	1.50	Wu FY et al., 2003	Lithos
285	9825-6	S. of MOS	Great Xing'an	124.93	52.49	Diorote	Tafeng	430	6.39	29.89	0.1292	0.512308	8	-6.4	-0.34	-2.7	1.39	Wu FY et al., 2003	Lithos
286	9825-7	S. of MOS	Great Xing'an	124.93	52.49	Diorote	Tafeng	430	4.71	25.13	0.1133	0.512215	7	-8.3	-0.42	-3.7	1.47	Wu FY et al., 2003	Lithos
287	S-7	S. of MOS	Great Xing'an	123.47	50.65	Moyite	Alihe	120	2.56	26.65	0.1146	0.512610	36	-0.5	-0.42	0.7	0.86	Wu FY et al., 2003	Lithos
288		S. of MOS	Transbaikalia-Mongolia	117.83	51.30	Monzonite	Shakhtama deposit	160	4.41	24.65	0.10815	0.512489		-2.9	-0.45	-1.1	1.04	Berzina A.P. et al.2014.	JAES
289		S. of MOS	Transbaikalia-Mongolia	117.83	51.30	Granite	Shakhtama deposit	159	5.59	38.10	0.08874	0.512386		-4.9	-0.55	-2.7	1.17	Berzina A.P. et al.2014.	JAES
290		S. of MOS	Transbaikalia-Mongolia	117.83	51.30	Monzonite porphyry	Shakhtama deposit	159	5.97	35.49	0.10166	0.512643		0.1	-0.48	2.0	0.78	Berzina A.P. et al.2014.	JAES
291		S. of MOS	Transbaikalia-Mongolia	117.83	51.30	Granite porphyry	Shakhtama deposit	155	5.23	32.38	0.09766	0.512645		0.1	-0.50	2.1	0.77	Berzina A.P. et al.2014.	JAES
292	M99-01	S. of MOS	Transbaikalia-Mongolia	99.73	46.35	Granite	Tsagaan Nuruu	514	3.09	18.45	0.1012	0.511952		-13.4	-0.49	-7.1	1.81	Jahn BM et al., 2004	JAES
293	M99-02	S. of MOS	Transbaikalia-Mongolia	99.75	46.37	Granite	Tsagaan Nuruu	514	3.37	19.42	0.1049	0.511988		-12.7	-0.47	-6.7	1.78	Jahn BM et al., 2004	JAES
294	M99-03	S. of MOS	Transbaikalia-Mongolia	99.85	46.43	Granodiorite	Tsagaan Nuruu	540	6.15	39.66	0.0937	0.512196		-8.6	-0.52	-1.5	1.38	Jahn BM et al., 2004	JAES
295	M99-04	S. of MOS	Transbaikalia-Mongolia	100.02	46.48	Diorite	Tsagaan Nuruu	250	5.04	26.73	0.1139	0.512434		-4.0	-0.42	-1.3	1.13	Jahn BM et al., 2004	JAES
296	M99-05	S. of MOS	Transbaikalia-Mongolia	100.02	46.48	Granite	Tsagaan Nuruu	250	3.92	23.48	0.1010	0.512430		-4.1	-0.49	-1.0	1.10	Jahn BM et al., 2004	JAES
297	M99-06	S. of MOS	Transbaikalia-Mongolia	100.05	46.82	Granite	Tsagaan Nuruu	237	3.46	21.38	0.0977	0.512448		-3.7	-0.50	-0.7	1.07	Jahn BM et al., 2004	JAES
298	M99-07	S. of MOS	Transbaikalia-Mongolia	100.05	46.82	Granite	Tsagaan Nuruu	250	5.31	29.98	0.1072	0.512489		-2.9	-0.46	-0.1	1.02	Jahn BM et al., 2004	JAES
299	M99-08	S. of MOS	Transbaikalia-Mongolia	100.05	46.82	Granite	Tsagaan Nuruu	237	3.56	22.89	0.0941	0.512400		-4.6	-0.52	-1.5	1.14	Jahn BM et al., 2004	JAES
300	M99-09	S. of MOS	Transbaikalia-Mongolia	99.97	46.82	Granite	Tsagaan Nuruu	250	3.85	21.69	0.1074	0.512469		-3.3	-0.45	-0.4	1.06	Jahn BM et al., 2004	JAES
301	M99-10	S. of MOS	Transbaikalia-Mongolia	99.97	46.82	Granitic dike	Tsagaan Nuruu	237	1.65	6.20	0.1610	0.512542		-1.9	-0.18	-0.8	1.07	Jahn BM et al., 2004	JAES
302	M99-11	S. of MOS	Transbaikalia-Mongolia	99.92	46.70	Metapelite	Tsagaan Nuruu	600	5.13	29.23	0.1062	0.512111		-10.3	-0.46	-3.4	1.58	Jahn BM et al., 2004	JAES
303	M99-12	S. of MOS	Transbaikalia-Mongolia	99.92	46.70	Pelitic ss	Tsagaan Nuruu	600	5.15	25.92	0.1200	0.512179		-9.0	-0.39	-3.1	1.56	Jahn BM et al., 2004	JAES
304	M99-13	S. of MOS	Transbaikalia-Mongolia	100.28	46.63	Micrgranite	Tsagaan Nuruu	250	5.97	30.46	0.1185	0.512490		-2.9	-0.40	-0.4	1.05	Jahn BM et al., 2004	JAES
305	M99-15	S. of MOS	Transbaikalia-Mongolia	101.42	45.95	Granite	Tsagaan Nuruu	230	3.85	26.22	0.0887	0.512297		-6.7	-0.55	-3.5	1.29	Jahn BM et al., 2004	JAES
306	M99-16	S. of MOS	Transbaikalia-Mongolia	101.52	45.93	Granite	Tsagaan Nuruu	230	8.33	46.02	0.1094	0.512300		-6.6	-0.44	-4.0	1.33	Jahn BM et al., 2004	JAES
307	M99-17	S. of MOS	Transbaikalia-Mongolia	102.37	45.90	Granodiorite	Tsagaan Nuruu	230	4.26	25.40	0.1014	0.512134		-9.8	-0.48	-7.0	1.58	Jahn BM et al., 2004	JAES
308	M99-18	S. of MOS	Transbaikalia-Mongolia	104.55	47.27	Granite	Tsagaan Nuruu	120	5.68	30.20	0.1137	0.512450		-3.7	-0.42	-2.4	1.11	Jahn BM et al., 2004	JAES
309	M99-19	S. of MOS	Transbaikalia-Mongolia	105.18	47.03	Ongonite	Tsagaan Nuruu	120	5.86	14.59	0.2427	0.512639		0.0	0.23	-0.7	0.98	Jahn BM et al., 2004	JAES
310	M99-20	S. of MOS	Transbaikalia-Mongolia	105.18	47.03	Ongonite	Tsagaan Nuruu	120	3.40	8.06	0.2549	0.512632		-0.1	0.30	-1.0	1.01	Jahn BM et al., 2004	JAES
311	M99-21	S. of MOS	Transbaikalia-Mongolia	105.18	47.03	Ongonite	Tsagaan Nuruu	120	5.53	14.02	0.2383	0.512609		-0.6	0.21	-1.2	1.03	Jahn BM et al., 2004	JAES
312	M99-25	S. of MOS	Transbaikalia-Mongolia	105.08	47.03	Granite	Tsagaan Nuruu	120	5.18	35.68	0.0878	0.512517		-2.4	-0.55	-0.7	0.97	Jahn BM et al., 2004	JAES
313	M99-26	S. of MOS	Transbaikalia-Mongolia	105.13	46.97	Granite	Tsagaan Nuruu	120	7.24	40.04	0.1092	0.512529		-2.1	-0.44	-0.8	0.98	Jahn BM et al., 2004	JAES
314	MG11-89-2	S. of MOS	Transbaikalia-Mongolia	100.00	46.42	MMEs	Ulaan Uul batholith	547	11.90	63.80	0.1124	0.512256		-7.5	-0.43	-1.6	1.39	Zhang Y et al.,2015.	JAES
315	MG11-89-3	S. of MOS	Transbaikalia-Mongolia	100.00	46.42	MMEs	Ulaan Uul batholith	547	3.87	35.10	0.0667	0.512070		-11.1	-0.66	-2.0	1.43	Zhang Y et al.,2015.	JAES
316	MG11-89-5	S. of MOS	Transbaikalia-Mongolia	100.00	46.42	MMEs	Ulaan Uul batholith	547	13.00	67.40	0.1168	0.512264		-7.3	-0.41	-1.7	1.40	Zhang Y et al.,2015.	JAES
317	MG11-89-8	S. of MOS	Transbaikalia-Mongolia	100.00	46.42	MMEs	Ulaan Uul batholith	547	8.22	51.10	0.0972	0.512174		-9.1	-0.51	-2.1	1.43	Zhang Y et al.,2015.	JAES
318	MG11-89-9	S. of MOS	Transbaikalia-Mongolia	100.00	46.42	Host granite	Ulaan Uul batholith	546	3.47	30.60	0.0687	0.512074		-11.0	-0.65	-2.1	1.43	Zhang Y et al.,2015.	JAES
319	MG11-89-14	S. of MOS	Transbaikalia-Mongolia	100.00	46.42	Host granite	Ulaan Uul batholith	546	8.70	73.20	0.0718	0.512097		-10.6	-0.63	-1.9	1.41	Zhang Y et al.,2015.	JAES
320	MG11-89-15	S. of MOS	Transbaikalia-Mongolia	100.00	46.42	Host granite	Ulaan Uul batholith	546	5.37	31.30	0.1038	0.512212		-8.3	-0.47	-1.8	1.41	Zhang Y et al.,2015.	JAES
321	MG11-89-18	S. of MOS	Transbaikalia-Mongolia	100.00	46.42	Host granite	Ulaan Uul batholith	546	7.81	44.40	0.1062	0.512216		-8.2	-0.46	-1.9	1.42	Zhang Y et al.,2015.	JAES
322	MO-144	S. of MOS	Transbaikalia-Mongolia	106.28	44.97	Granite	Olzit	208	19.90	105.9	0.1140	0.512567		-1.4	-0.42	0.8	0.92	Zhu Mingshuai et al.,2016.	JAES
323	MO-145	S. of MOS	Transbaikalia-Mongolia	106.28	44.97	Granite	Olzit	208	17.20	90.10	0.1157	0.512567		-1.4	-0.41	0.8	0.92	Zhu Mingshuai et al.,2016.	JAES
324	ON-3	S. of MOS	Transbaikalia-Mongolia	107.50	45.50	Nepheline rock	dyke	120	6.20	15.11	0.2481	0.512627		-0.2	0.26	-1.0	1.01	Dostal J et al.,2015.	Lithos
325	ON-13	S. of MOS	Transbaikalia-Mongolia	107.50	45.50	Nepheline rock	dyke	120	4.96	12.34	0.2430	0.512626		-0.2	0.24	-0.9	1.01	Dostal J et al.,2015.	Lithos
326	ON-21	S. of MOS	Transbaikalia-Mongolia	107.50	45.50	Nepheline rock	dyke	120	3.43	8.19	0.2532	0.512625		-0.3	0.29	-1.1	1.02	Dostal J et al.,2015.	Lithos
327	M99-19*	S. of MOS	Transbaikalia-Mongolia	107.50	45.50	Nepheline rock	dyke	120	5.86	14.59	0.2427	0.512639		0.0	0.23	-0.7	0.98	Dostal J et al.,2015.	Lithos
328	M99-20*	S. of MOS	Transbaikalia-Mongolia	107.50	45.50	Nepheline rock	dyke	120	3.40	8.06	0.2549	0.512632		-0.1	0.30	-1.0	1.01	Dostal J et al.,2015.	Lithos
329	M99-21*	S. of MOS	Transbaikalia-Mongolia	107.50	45.50	Nepheline rock	dyke	120	5.53	14.02	0.2383	0.512609		-0.6	0.21	-1.2	1.03	Dostal J et al.,2015.	Lithos
330	7580	S. of MOS	Transbaikalia-Mongolia	101.52	45.73	Island-arc complex	Tatsaingol	603	6.32	29.90	0.1276	0.512330		-6.0	-0.35	-0.7	1.36	Kozakov I.K.et al.,2015.	Petrology
331	2074610	S. of MOS	Transbaikalia-Mongolia	101.38	45.80	Island-arc complex	Tatsaingol	603	6.75	31.60	0.1292	0.512345		-5.7	-0.34	-0.5	1.35	Kozakov I.K.et al.,2015.	Petrology
332	2074639	S. of MOS	Transbaikalia-Mongolia	101.38	45.80	Island-arc complex	Tatsaingol	603	4.70	20.10	0.1415	0.512381		-5.0	-0.28	-0.8	1.37	Kozakov I.K.et al.,2015.	Petrology
333	1969025	S. of MOS	Transbaikalia-Mongolia	101.38	45.80	Island-arc complex	Tatsaingol	603	4.40	22.00	0.1206	0.512229		-8.0	-0.39	-2.1	1.48	Kozakov I.K.et al.,2015.	Petrology
334	7291	S. of MOS	Transbaikalia-Mongolia	101.38	45.80	Island-arc complex	Tatsaingol	603	3.41	13.84	0.1490	0.512436		-3.9	-0.24	-0.3	1.33	Kozakov I.K.et al.,2015.	Petrology
335	6180	S. of MOS	Transbaikalia-Mongolia	101.38	45.80	Island-arc complex	Tatsaingol	603	10.46	57.00	0.1109	0.512238		-7.8	-0.44	-1.2	1.41	Kozakov I.K.et al.,2015.	Petrology
336	6184	S. of MOS	Transbaikalia-Mongolia	101.38	45.80	Island-arc complex	Tatsaingol	603	6.47	37.00	0.1056	0.512271		-7.2	-0.46	-0.1	1.32	Kozakov I.K.et al.,2015.	Petrology
337	7562	S. of MOS	Transbaikalia-Mongolia	100.88	45.91	Granitoid	Tatsaingol	570	3.64	19.96	0.1103	0.512085		-10.8	-0.44	-4.5	1.65	Kozakov I.K.et al.,2015.	Petrology
338	6176	S. of MOS	Transbaikalia-Mongolia	101.67	45.53	Granitoid	Tatsaingol	565	2.84	9.68	0.1773	0.512022		-12.0	-0.10	-10.6	2.12	Kozakov I.K.et al.,2015.	Petrology
339	6124	S. of MOS	Transbaikalia-Mongolia	101.67	45.53	Granitoid	Tatsaingol	565	2.70	16.96	0.0962	0.511870		-15.0	-0.51	-7.7	1.91	Kozakov I.K.et al.,2015.	Petrology
340	5967	S. of MOS	Transbaikalia-Mongolia	101.38	45.78	Granitoid	Tatsaingol	562	0.93	5.69	0.0988	0.511962		-13.2	-0.50	-6.2	1.78	Kozakov I.K.et al.,2015.	Petrology
341	6035	S. of MOS	Transbaikalia-Mongolia	101.37	45.72	Granitoid	Tatsaingol	565	5.23	31.30	0.1008	0.512209		-8.4	-0.49	-1.5	1.40	Kozakov I.K.et al.,2015.	Petrology
342	1563240	S. of MOS	Transbaikalia-Mongolia	101.38	45.68	Granitoid	Tatsaingol	565	1.76	11.13	0.0954	0.512131		-9.9	-0.51	-2.6	1.49	Kozakov I.K.et al.,2015.	Petrology
343	6425	S. of MOS	Transbaikalia-Mongolia	101.38	45.68	Granitoid	Tatsaingol	561	3.58	20.60	0.1052	0.511826		-15.8	-0.47	-9.3	2.03	Kozakov I.K.et al.,2015.	Petrology
344	6194	S. of MOS	Transbaikalia-Mongolia	101.40	45.75	Granitoid	Tatsaingol	552	5.52	31.50	0.1059	0.512050		-11.5	-0.46	-5.1	1.68	Kozakov I.K.et al.,2015.	Petrology
345	5975	S. of MOS	Transbaikalia-Mongolia	101.40	45.75	Granitoid	Tatsaingol	547	3.73	35.70	0.0632	0.512068		-11.1	-0.68	-1.8	1.41	Kozakov I.K.et al.,2015.	Petrology
346	1570910	S. of MOS	Transbaikalia-Mongolia	101.42	45.72	Granitoid	Tatsaingol	547	8.09	60.00	0.0814	0.512000		-12.4	-0.59	-4.4	1.62	Kozakov I.K.et al.,2015.	Petrology
347	6036	S. of MOS	Transbaikalia-Mongolia	101.42	45.72	Granitoid	Tatsaingol	547	5.21	30.10	0.1047	0.512164		-9.2	-0.47	-2.8	1.49	Kozakov I.K.et al.,2015.	Petrology
348	1510645	S. of MOS	Transbaikalia-Mongolia	101.42	45.72	Granitoid	Tatsaingol	547	8.18	38.00	0.1300	0.512321		-6.2	-0.34	-1.5	1.39	Kozakov I.K.et al.,2015.	Petrology
349	6201	S. of MOS	Transbaikalia-Mongolia	101.42	45.72	Granitoid	Tatsaingol	547	6.66	39.20	0.1026	0.512247		-7.6	-0.48	-1.1	1.35	Kozakov I.K.et al.,2015.	Petrology
350	1570941	S. of MOS	Transbaikalia-Mongolia	101.42	45.72	Granitoid	Tatsaingol	547	7.93	39.70	0.1207	0.512182		-8.9	-0.39	-3.6	1.55	Kozakov I.K.et al.,2015.	Petrology
351	6006	S. of MOS	Transbaikalia-Mongolia	101.42	45.72	Granitoid	Tatsaingol	546	4.49	26.00	0.1042	0.511998		-12.5	-0.47	-6.0	1.75	Kozakov I.K.et al.,2015.	Petrology
352	6427	S. of MOS	Transbaikalia-Mongolia	101.42	45.72	Postmetamorphic granitoid	Tatsaingol	545	8.65	47.30	0.1106	0.511807		-16.2	-0.44	-10.2	2.09	Kozakov I.K.et al.,2015.	Petrology
353	6197	S. of MOS	Transbaikalia-Mongolia	101.42	45.72	Postmetamorphic granitoid	Tatsaingol	545	3.01	18.19	0.1000	0.511801		-16.3	-0.49	-9.6	2.04	Kozakov I.K.et al.,2015.	Petrology

Supplementary table captions

Table S3 Geochemical data of Triassic granitoids from the CAOB.

No.	Sample	Tectonic unit	Location and unit	Longitude	Latitude	Lithology	Pluton	Age	Sm	Nd	$^{147}Sm/^{144}Nd$	$^{143}Nd/^{144}Nd$	2σ	εNd(0)	$f_{Sm/Nd}$	εNd(t)	T_{DM2} (Ga)	References	
354	5965	S. of MOS	Transbaikalia-Mongolia	101.42	45.72	Postmetamorphic granitoid	Tatsaingol	545	10.27	52.70	0.1178	0.511819		-16.0	-0.40	-10.5	2.11	Kozakov I.K.et al.,2015.	Petrology
355	6422	S. of MOS	Transbaikalia-Mongolia	101.42	45.72	Postmetamorphic granitoid	Tatsaingol	545	0.93	6.76	0.0828	0.512144		-9.6	-0.58	-1.7	1.40	Kozakov I.K.et al.,2015.	Petrology
356	Sh-11	S. of MOS	Transbaikalia-Mongolia	107.77	44.63	Adakitic	Dusiin Ovoo Formation	321.5	1.40	6.60	0.1290	0.512806		3.3	-0.34	6.1	0.59	Batkhishig et al. , 2010	JAES
357	Sh-17	S. of MOS	Transbaikalia-Mongolia	107.77	44.65	Adakitic	Dusiin Ovoo Formation	321.5	0.30	1.60	0.1180	0.512815		3.5	-0.40	6.7	0.53	Batkhishig et al. , 2010	JAES
358	Sh-18	S. of MOS	Transbaikalia-Mongolia	107.78	44.83	Adakitic	Shuteen	321.5	2.50	13.60	0.1120	0.512733		1.9	-0.43	5.3	0.64	Batkhishig et al. , 2010	JAES
359	Sh-0101	S. of MOS	Transbaikalia-Mongolia	107.78	44.87	Adakitic	Shuteen	321.5	2.40	13.90	0.1040	0.512748		2.1	-0.47	5.9	0.59	Batkhishig et al. , 2010	JAES
360	Sh-0104	S. of MOS	Transbaikalia-Mongolia	107.78	44.93	Adakitic	Shuteen	321.5	3.00	13.80	0.1310	0.512820		3.6	-0.33	6.2	0.57	Batkhishig et al. , 2010	JAES
361	Sh-0124	S. of MOS	Transbaikalia-Mongolia	107.78	44.95	Adakitic	Shuteen	321.5	3.00	14.60	0.1240	0.512792		3.0	-0.37	6.0	0.59	Batkhishig et al. , 2010	JAES
362	16	S. of MOS	Transbaikalia-Mongolia	114.11	56.57	Massif	Bodonchin nappe	370	2.79	12.94	0.1302	0.512842		4.0	-0.34	7.1	0.54	Kozakov I.K.et al.,2007	Petrology
363	17	S. of MOS	Transbaikalia-Mongolia	100.37	45.50	Ultrametamorphic granitoid	Bodonchin nappe	360	21.75	82.30	0.1597	0.512842		4.0	-0.19	5.7	0.65	Kozakov I.K.et al.,2007	Petrology
364	18	S. of MOS	Transbaikalia-Mongolia	100.33	45.92	Ultrametamorphic granitoid	Bodonchin nappe	360	2.23	8.26	0.1634	0.512571		-1.3	-0.17	0.2	1.09	Kozakov I.K.et al.,2007	Petrology
365	19	S. of MOS	Transbaikalia-Mongolia	100.50	45.67	Ultrametamorphic granitoid	Bodonchin nappe	360	5.86	35.10	0.1010	0.512607		-0.6	-0.49	3.8	0.80	Kozakov I.K.et al.,2007	Petrology
366	20	S. of MOS	Transbaikalia-Mongolia	100.58	45.42	Ultrametamorphic granitoid	Bodonchin nappe	360	4.89	24.20	0.1222	0.512535		-2.0	-0.38	1.4	1.00	Kozakov I.K.et al.,2007	Petrology
367	21	S. of MOS	Transbaikalia-Mongolia	100.92	45.25	Ultrametamorphic granitoid	Bodonchin nappe	360	4.86	24.90	0.1180	0.512538		-2.0	-0.40	1.7	0.97	Kozakov I.K.et al.,2007	Petrology
368	22	S. of MOS	Transbaikalia-Mongolia	100.83	45.33	Ultrametamorphic granitoid	Bodonchin nappe	355	3.67	13.18	0.1685	0.512514		-2.4	-0.14	-1.1	1.20	Kozakov I.K.et al.,2007	Petrology
369	23	S. of MOS	Transbaikalia-Mongolia	100.97	45.67	Ultrametamorphic granitoid	Bodonchin nappe	360	2.64	9.73	0.1635	0.512454		-3.6	-0.17	-2.1	1.27	Kozakov I.K.et al.,2007	Petrology
370	24	S. of MOS	Transbaikalia-Mongolia	97.08	46.33	Granitoid	Bodonchin nappe	280	2.50	14.20	0.1066	0.512587		-1.0	-0.46	2.2	0.86	Kozakov I.K.et al.,2007	Petrology
371	25	S. of MOS	Transbaikalia-Mongolia	97.17	46.38	Granitoid	Bodonchin nappe	280	10.90	42.90	0.1532	0.512675		0.7	-0.22	2.3	0.86	Kozakov I.K.et al.,2007	Petrology
372	26	S. of MOS	Transbaikalia-Mongolia	97.17	46.43	Granitoid	Bodonchin nappe	240	3.96	25.60	0.0926	0.512610		-0.5	-0.53	2.6	0.80	Kozakov I.K.et al.,2007	Petrology
373	27	S. of MOS	Transbaikalia-Mongolia	98.10	46.48	Granitoid	Bodonchin nappe	270	9.91	49.50	0.1200	0.512632		-0.1	-0.39	2.5	0.83	Kozakov I.K.et al.,2007	Petrology
374	28	S. of MOS	Transbaikalia-Mongolia	97.22	46.50	Granitoid	Bodonchin nappe	350	9.24	51.10	0.1083	0.512655		0.3	-0.45	4.3	0.75	Kozakov I.K.et al.,2007	Petrology
375	29	S. of MOS	Transbaikalia-Mongolia	97.25	46.58	Granitoid	Bodonchin nappe	270	8.42	43.30	0.1176	0.512553		-1.7	-0.40	1.1	0.95	Kozakov I.K.et al.,2007	Petrology
376	30	S. of MOS	Transbaikalia-Mongolia	98.17	46.33	Granitoid	Bodonchin nappe	280	3.56	22.50	0.0957	0.512636		0.0	-0.51	3.6	0.75	Kozakov I.K.et al.,2007	Petrology
377	31	S. of MOS	Transbaikalia-Mongolia	99.33	46.60	Granitoid	Bodonchin nappe	280	13.20	65.50	0.1218	0.512788		2.9	-0.38	5.6	0.59	Kozakov I.K.et al.,2007	Petrology
378	32	S. of MOS	Transbaikalia-Mongolia	100.43	46.67	Granitoid	Bodonchin nappe	330	9.02	26.00	0.2091	0.512886		4.8	0.06	4.3	0.72	Kozakov I.K.et al.,2007	Petrology
379	33	S. of MOS	Transbaikalia-Mongolia	100.38	46.92	Granitoid	Bodonchin nappe	330	5.62	26.80	0.1265	0.512693		1.1	-0.36	4.0	0.76	Kozakov I.K.et al.,2007	Petrology
380	34	S. of MOS	Transbaikalia-Mongolia	100.42	46.83	Granitoid	Bodonchin nappe	270	3.73	18.34	0.1218	0.512675		0.7	-0.38	3.3	0.77	Kozakov I.K.et al.,2007	Petrology
381	MO-122	S. of MOS	Middle Mongolia	106.31	44.96	Basalt	Olzit area	208	8.6	43.4	0.12	0.512514	±8			-0.38	1.02	Zhu Mingshuai et al.,2016	Journal of Asian Earth Sciences
382	MO-130	S. of MOS	Middle Mongolia	106.31	44.96	Basalt	Olzit area	208	7.9	40.6	0.1175	0.512492	±8			-0.74	1.05	Zhu Mingshuai et al.,2016	Journal of Asian Earth Sciences
383	MO-137	S. of MOS	Middle Mongolia	106.31	44.96	Basalt	Olzit area	208	7.8	39.4	0.1206	0.512496	±9			-0.76	1.05	Zhu Mingshuai et al.,2016	Journal of Asian Earth Sciences
384	MO-164	S. of MOS	Middle Mongolia	106.31	44.96	Basalt	Olzit area	208	9.7	32.6	0.1793	0.512486	±9			-2.5	1.13	Zhu Mingshuai et al.,2016	Journal of Asian Earth Sciences
385	MO-165	S. of MOS	Middle Mongolia	106.31	44.96	Basalt	Olzit area	208	5.6	28.8	0.1175	0.512472	±8			-1.13	1.08	Zhu Mingshuai et al.,2016	Journal of Asian Earth Sciences
386	MO-166	S. of MOS	Middle Mongolia	106.31	44.96	Basalt	Olzit area	208	5.6	28.5	0.1195	0.512473	±9			-1.17	1.08	Zhu Mingshuai et al.,2016	Journal of Asian Earth Sciences
387	MO-132	S. of MOS	Middle Mongolia	106.31	44.96	Rhyolite	Olzit area	208	28.1	148	0.1151	0.512552	±8			0.5	0.95	Zhu Mingshuai et al.,2016	Journal of Asian Earth Sciences
388	MO-142	S. of MOS	Middle Mongolia	106.31	44.96	Rhyolite	Olzit area	208	35.4	182.8	0.1173	0.512562	±10			0.62	0.94	Zhu Mingshuai et al.,2016	Journal of Asian Earth Sciences
389	MO-156	S. of MOS	Middle Mongolia	106.31	44.96	Rhyolite	Olzit area	208	28.2	143.6	0.119	0.512603	±9			1.38	0.87	Zhu Mingshuai et al.,2016	Journal of Asian Earth Sciences
390	MO-158	S. of MOS	Middle Mongolia	106.31	44.96	Rhyolite	Olzit area	208	27.9	138.7	0.1219	0.512602	±8			1.29	0.88	Zhu Mingshuai et al.,2016	Journal of Asian Earth Sciences
391	MO-144	S. of MOS	Middle Mongolia	106.28	44.97	Granite	Olzit area	208	19.9	105.9	0.114	0.512567	±7			0.8	0.93	Zhu Mingshuai et al.,2016	Journal of Asian Earth Sciences
392	MO-145	S. of MOS	Middle Mongolia	106.28	44.97	Granite	Olzit area	208	17.2	90.1	0.1157	0.512567	±8			0.77	0.93	Zhu Mingshuai et al.,2016	Journal of Asian Earth Sciences
393	D0925	S. of MOS	Middle Mongolia	105.00	46.15	Granodiorite	~296 Intrusion	350	4.2701	26.909	0.095915	0.51231918	4			-1.7	1.26	Ariuntsetseg G et al.,2021	Lithos
394	D1709	S. of MOS	Middle Mongolia	104.77	47.08	Rhyolite	Batkhaan	274	4.4565	19.189	0.140295	0.51267275	3			2.7	0.84	Ariuntsetseg G et al.,2021	Lithos
395	D1710	S. of MOS	Middle Mongolia	104.13	47.07	Syenogranite	Batkhaan	282	4.71	21.137	0.127136	0.51271864	2			4.1	0.73	Ariuntsetseg G et al.,2021	Lithos
396	D1726	S. of MOS	Middle Mongolia	104.34	46.94	Granodiorite	Zambalkhudag	220	4.9265	19.62	0.15177	0.51253692	2			-0.7	1.07	Ariuntsetseg G et al.,2021	Lithos
397	D1742	S. of MOS	Middle Mongolia	104.62	46.67053	Monzogranite	Zambalkhudag	240	2.6504	20.112	0.079656	0.51253893	4			1.7	0.89	Ariuntsetseg G et al.,2021	Lithos

Note:N. = North; S. = South;Transbaikalia-Mongolia= Transbaikalia and mongolia; MOS = Mongol-Okhotsk Suture; DES = Doklady Earth Sciences; GC = Geology in China; GBC = Geological Bulletin of China; IGR = International Geology Review; OGR = Ore Geology Reviews; AGS = Acta Geoscientica Sinica; RGG = Russian Geology and Geophysics; APS = Acta Petrologica Sinica; JJU = Journal of Jilin University; PHD diss., JU = PHD dissertation, Jili University; JAES = Journal of Asian Earth Sciences; CUG = China University of Geosciences; ICWEA = in Chinese with English abstract; Bi = biotite; Hb = hornblende; Qtz = quartz

Table S3 Geochemstry data of the Early Mesozoic (Triassic) granitoids in southern CAOB

No.	Location	Magmatic belt	Group	Longitude	Latitude	Sample	Rock type	Pluton	Age（Ma）	SiO_2	TiO_2	Al_2O	TFe_2	Fe_2O	FeO	MnO	MgO	CaO	Na_2O	K_2O	P_2O_5	LOI	$Mg^{\#}$	La	Ce	Pr	Nd	Sm	Eu	Gd	Tb	Dy	Ho	Er	Tm	Yb	Lu	Y	Cs	Rb	Sr
1	Altai	Altai	1			I9	Granitoid			####	0.32	####		0.79	1.13	0.13	0.50	2.17	3.95	8.22	0.14	1.02		90.38	200.3	19.51	73.41	11.26	2.09	9.59	1.12	6.09	1.11	3.14	0.42	2.18	0.23	26.04		384	742.7
2	Altai	Altai	1			N7	Granitoid			####	0.47	####		1.03	1.68	0.12	0.76	2.02	4.93	6.76	0.17	0.54		128.65	231.4	24.15	90.65	14.81	2.67	14.32	1.47	7.38	1.74	4.47	0.57	4.03	0.57	39.74		251	984.0
3	Altai	Altai	1			31	Granitoid			####	0.65	####		3.24	2.93	0.15	2.55	3.53	5.02	5.29	0.79	0.80		185.3	298.6	27.73	96.74	12.95	2.96	12.01	1.41	5.37	0.94	2.83	0.36	2.06	0.34	22.83		238	3890
4	Altai	Altai	1			18	Granitoid			####	0.36	####		2.02	1.24	0.08	0.79	2.14	6.21	5.87	0.25	1.31		69.59	105.2	9.39	32.31	4.10	1.48	3.24	0.39	1.51	0.28	0.73	0.10	0.49	0.10	5.28		207	1360
5	Altai	Altai	1			179/1	Granitoid			####	0.36	####		2.46	0.23	0.03	0.49	0.70	5.89	6.89	0.15	0.68		125.7	235.0	22.37	78.66	11.55	2.14	10.64	1.40	6.26	1.13	3.15	0.41	2.71	0.41	28.54		209	770
6	Altai	Altai	1			007/1	Granitoid			####	0.18	####		0.75	0.70	0.03	0.24	1.08	3.48	5.95	0.04	0.47		69.28	127.5	11.67	39.39	6.45	0.57	4.79	0.42	1.48	0.21	0.60	0.10	0.23	0.10	3.34		172	82.0
7	Altai	Altai	1			3211	Bi monzogranite			####	0.22	####		0.58	1.15	0.24	0.29	0.06	2.80	5.16	0.04	1.40		85.30	159.0	16.80	54.70	10.30	1.00	6.17	1.06	6.01	1.18	3.70	0.61	4.56	0.80	30.90	8.77	390	83.6
8	Altai	Altai	1			32112	Two-mica granite			####	0.10	####		0.60	1.19	0.10	0.56	0.03	3.41	3.02	0.03	1.70		46.50	80.30	8.15	26.10	5.49	0.58	4.25	0.91	6.09	1.26	4.28	0.78	6.37	1.13	40.70	4.71	348	52.7
9	Altai	Altai	1			3141-A	Two-mica granite			####	<0.01	####		0.23	0.45	0.04	0.01	0.25	4.25	4.10	0.02	0.80		5.60	17.90	2.47	12.60	5.89	0.02	10.20	2.27	18.70	3.89	13.59	2.38	16.86	2.60	135.0	42.00	534	2.10
10	Altai	Altai	1			3141-B	Two-mica granite			####	<0.01	####		0.25	0.51	0.03	0.02	0.33	4.12	4.02	<0.01	0.80		6.30	18.50	2.63	11.30	6.89	0.01	11.80	2.68	22.40	4.62	16.70	2.93	21.50	3.17	178.0	37.00	510	1.90
11	Altai	Altai	1			3142	Two-mica granite			####	<0.01	####		0.13	0.26	0.02	0.01	0.50	4.69	3.76	<0.01	0.80		7.30	18.20	3.00	14.30	8.78	0.02	14.90	3.30	27.60	5.86	21.00	3.73	27.30	4.58	215.0	45.20	485	2.20
12	Altai	Altai	1			3146	Two-mica granite			####	<0.01	####		0.11	0.21	<0.01	0.02	0.14	3.49	4.76	<0.01	0.70		7.30	21.10	4.33	23.40	14.30	0.02	19.30	4.15	31.00	5.38	18.50	3.24	23.50	3.43	122.0	10.80	515	1.90
13	Mongolian Altai	Altai	1			5041	Granite	Uench River	210	####	0.33	####		2.00		0.05	0.50	1.74	3.70	4.79	0.14			16.00	30.70	3.10	10.60	1.90	0.40	1.60	0.20	1.10	0.20	0.50	0.10	0.60	0.10	6.00	2.70	99.0	100
14	Altai	Altai	1			3013	Porphyritic Bi granite	Alaer	210	####	0.46	####		0.81	1.31	0.04	0.60	1.53	3.00	6.28	0.24			88.00	172.0	20.20	70.70	13.10	2.21	8.18	1.16	5.73	0.96	2.52	0.31	1.84	0.28	21.90	3.84	187	274
15	Altai	Altai	1			3014	Granodiorite	Alaer	210	####	0.24	####		0.99	1.28	0.03	0.52	0.93	2.53	6.36	0.12			138.0	276.0	32.40	118.0	21.30	2.34	12.70	1.59	7.15	1.02	2.46	0.23	1.18	0.18	23.20	3.30	186	297
16	Altai	Altai	1			3028	Bi monzogranite	Alaer	210	####	0.18	####		0.32	0.99	0.02	0.47	0.94	3.18	5.55	0.19			36.90	76.20	8.77	32.80	6.71	0.69	4.10	0.60	2.84	0.45	1.09	0.13	0.77	0.11	10.80	8.34	194	87.2
17	Altai	Altai	1			3033	Monzogranite	Alaer	210	####	0.27	####		0.38	1.19	0.02	0.52	1.09	2.93	5.66	0.29			49.20	101.0	12.20	42.50	9.07	0.92	5.27	0.73	3.66	0.60	1.59	0.21	1.24	0.20	14.10	5.52	288	107
18	Altai	Altai	1			3037	Bi monzogranite	Alaer	210	####	0.20	####		0.44	1.00	0.02	0.44	1.06	2.81	6.16	0.35			35.10	73.20	8.53	31.50	7.54	0.91	5.24	0.80	4.28	0.74	2.13	0.29	1.81	0.26	19.10	4.55	248	127
19	Altai	Altai	1			3143	Two-mica granite			####	0.01	####		0.18	0.46	0.01	0.02	0.28	5.33	3.04	0.01	0.58	0.06	11.70	35.80	5.18	21.40	12.80	0.06	14.90	4.40	33.80	7.58	26.70	5.38	40.40	6.43	109.0	11.40	374	8.15
20	Altai	Altai	1			3148	Porphyritic Bi granite			####	0.27	####		0.37	1.65	0.05	0.52	1.31	3.41	4.24	0.23	0.54	0.2	50.10	100.0	12.00	44.80	11.20	0.85	10.20	2.09	13.10	2.73	7.98	1.26	8.24	1.27	66.60	25.00	236	80.3
21	Altai	Altai	1			3150	Bi granite			####	0.01	####		0.03	0.48	0.01	0.04	0.40	4.95	3.26	<0.01	0.33	0.07	12.90	27.50	4.58	21.20	11.70	0.04	16.60	3.95	27.90	6.15	19.10	3.04	19.50	2.93	163.0	7.74	279	5.31
22	Altai	Altai	1			AT-1	Two-mica monzogranite			####	0.01	####				0.01	0.01	0.43	3.73	4.09	0.03	0.59	0	6.34	14.85	2.22	9.60	4.43	0.04	6.35	1.33	10.58	2.77	8.60	1.58	12.33	2.18	108.9	39.10	526	5.70
23	Altai	Altai	1			3212	Two-mica granite	Shankelan	200	####	0.22	####		1.12	0.39	0.04	0.26	0.44	4.50	4.22	0.07	0.68	0.05	85.30	159.0	16.80	54.70	10.30	1.00	6.17	1.06	6.01	1.18	3.70	0.61	4.56	0.80	30.90	8.77	390	83.6
24	Altai	Altai	1			3214	Bi monzogranite	Shankelan	200	####	0.11	####		0.27	0.47	0.04	0.22	0.08	3.76	4.74	0.03	0.65	0.07	46.50	80.30	8.15	26.10	5.49	0.58	4.25	0.91	6.09	1.26	4.28	0.78	6.37	1.13	40.70	4.71	348	52.7
25	Altai	Altai	1			XSK-2	Granitoid	Shankelan	200	####	0.08	####		0.28	0.54	0.05	0.12	0.05	5.45	4.17	0.19			27.01	45.16	5.82	15.67	3.44	0.23	1.76	0.32	1.24	0.25	0.81	0.18	1.35	0.21				
26	Beishan	Beishan	2			XT-42	Porphyritic Hb-Bi monzogra	North of Ma'anshan	237	####	0.23	####		0.05	1.85	0.46	0.46	1.66	3.86	3.86	0.06			17.10	26.00	2.13	9.17	2.23	0.37	1.74	0.28	1.75	0.31	1.15	0.19	1.18	0.16	9.92	8.10	144	320
27	Beishan	Beishan	2			XT-41	Porphyritic Hb-Bi monzogra	North of Ma'anshan	237	####	0.56	####		0.71	2.53	0.57	1.29	3.50	4.28	2.25	0.14			23.70	38.10	3.84	15.80	3.28	0.73	2.42	0.34	1.90	0.35	0.92	0.14	0.78	0.10	8.68	6.70	59.20	622
28	Beishan	Beishan	2			XT-61	Porphyritic Hb-Bi monzogra	North of Ma'anshan	237	####	0.76	####		1.45	3.59	0.09	2.61	5.24	4.39	2.15	0.28			29.50	53.80	5.96	25.70	4.81	1.19	3.97	0.62	3.44	0.57	1.83	0.28	1.60	0.23	14.70	6.20	63.00	679
29	Beishan	Beishan	2			XT-40	Porphyritic Hb-Bi monzogra	North of Ma'anshan	237	####	0.46	####		0.93	2.47	0.06	1.34	4.01	4.22	2.68	0.17			23.10	38.90	3.36	15.10	2.74	0.72	2.32	0.34	1.91	0.23	0.65	0.10	0.67	0.09	7.36	5.90	64.50	583
30	Beishan	Beishan	2			XT-39	Porphyritic Hb-Bi monzogra	North of Ma'anshan	237	####	0.89	####		1.54	4.42	0.11	3.40	6.29	4.30	1.92	0.28			27.30	45.90	5.36	22.40	4.70	1.23	3.88	0.62	3.83	0.67	2.18	0.28	1.61	0.19	16.00	5.90	57.70	669
31	Beishan	Beishan	2			NJS01-14	Monzogranite	South Jinshan	244	####	0.73	####		2.35	3.65	0.13	2.99	5.60	2.71	3.09	0.21			33.10	58.80	5.88	26.97	5.22	1.23	5.23	0.77	3.80	0.75	2.39	0.33	2.08	0.34	19.06		155	326
32	Beishan	Beishan	2			NJS01-15	Monzogranite	South Jinshan	244	####	0.70	####		2.14	3.58	0.13	2.53	5.00	3.11	2.96	0.20			32.97	60.99	5.77	26.29	5.18	1.23	5.23	0.71	3.88	0.75	2.51	0.33	2.17	0.37	19.47		121	364
33	Beishan	Beishan	2			NJS01-11	Monzogranite	South Jinshan	218	####	0.58	####		0.81	2.18	0.09	1.27	2.73	3.26	4.17	0.18			67.75	117.7	10.70	43.15	7.59	1.47	6.30	0.75	3.27	0.60	1.97	0.26	1.44	0.24	14.55		199	371
34	Beishan	Beishan	2			NJS01-13	Monzogranite	South Jinshan	218	####	0.41	####		1.88	2.16	0.12	1.44	3.83	2.67	4.53	0.12			27.48	48.63	4.38	19.58	3.52	0.87	3.74	0.55	2.76	0.52	1.49	0.21	1.37	0.24	12.46		197	296
35	Beishan	Beishan	2			NJS01-25	Monzogranite	South Jinshan	218	####	0.21	####		0.93	0.74	0.07	0.36	1.79	3.31	4.12	0.06			41.30	67.60	5.95	22.87	3.86	0.73	3.97	0.53	2.28	0.42	1.26	0.16	0.93	0.17	8.85		186	216
36	Beishan	Beishan	2			B70822-4	K-feldspar granite	Huaniushan	220	####	0.27	####		0.85	0.37	0.04	0.20	0.82	3.87	5.01	0.07	0.58		85.581	154.45	15.64	48.15	6.94	0.86	4.95	0.71	3.92	0.77	2.39	0.37	2.40	0.35	22.79			
37	Beishan	Beishan	2			B70822-8	K-feldspar granite	Huaniushan	235	####	0.15	####		0.44	0.94	0.02	0.14	0.59	3.50	4.66	0.07	0.41		49.809	98.00	11.12	39.81	8.55	0.33	8.39	1.51	9.53	2.09	6.53	1.03	6.57	0.95	61.87			
38	East Tianshan	East Tianshan	2			x-358	Qtz syenite	Weiya	235	####	0.45	####		0.81	3.57	0.12	0.37	2.21	5.94	5.06	0.23	0.53		21.09	43.84	5.62	22.54	4.49	4.18	4.44	0.58	4.02	0.81	2.49	0.34	2.19	0.39	22.08	1.45	80.13	63.90
39	East Tianshan	East Tianshan	2			x-58	Qtz syenite	Weiya	235	####	0.70	####		2.22	3.27	0.17	0.36	2.25	4.64	6.33	0.07	0.68		21.66	46.49	6.03	24.01	4.94	2.59	4.69	0.60	4.16	0.91	2.71	0.37	2.70	0.44	24.17	1.79	88.55	53.30
40	East Tianshan	East Tianshan	2			x-60	Qtz syenite	Weiya	235	####	0.63	####		3.15	2.19	0.07	0.42	2.39	4.34	6.15	0.08	0.45		32.37	66.87	7.63	27.10	4.26	1.32	4.22	0.47	3.23	0.68	2.01	0.31	2.26	0.35	19.55	3.11	156.26	255.6
41	East Tianshan	East Tianshan	2			x-61	Qtz syenite	Weiya	235	####	0.70	####		2.06	3.54	0.22	0.34	2.75	4.05	6.20	0.06	0.31		21.57	54.10	7.72	32.14	7.31	2.44	6.76	0.91	5.89	1.23	3.44	0.49	3.39	0.56	33.36	4.51	128.91	158.2
42	East Tianshan	East Tianshan	2			x-99	Qtz syenite	Weiya	235	####	0.57	####		0.69	1.83	0.11	0.43	1.72	4.17	6.66	0.08	0.57		14.88	32.18	4.21	17.45	4.07	3.20	4.31	0.64	4.81	1.07	3.60	0.61	4.77	0.76	36.31	42.36	410.77	129.4
43	East Tianshan	East Tianshan	2			1501/2	Bi monzogranite	Tianhu	220	####	0.43	####		0.51	1.92	0.06	1.43	2.24	3.70	3.39	0.12			30.89	60.11	7.44	25.25	4.46	0.75	3.58	0.49	3.07	0.58	1.49	0.24	1.46	0.22	16.23	4.90	115	225
44	East Tianshan	East Tianshan	2			1503/1-2	Monzogranite	Tianhu	220	####	0.43	####		1.23	1.26	0.05	1.39	2.44	3.89	3.52	0.12			21.53	44.88	4.84	17.75	3.12	0.77	2.59	0.35	2.19	0.41	1.09	0.18	1.12	0.17	12.08	5.10	128	258
45	East Tianshan	East Tianshan	2			1505-1	Bi granodiorite	Tianhu	220	####	0.43	####		0.46	1.92	0.05	1.53	2.20	3.63	3.78	0.12			37.52	79.23	8.40	28.47	4.62	0.77	3.62	0.45	2.84	0.56	1.40	0.22	1.43	0.22	15.34	6.60	141	228
46	Inner Mongolia	Solonker-Xilamulun	2			D106-1	Granite	Shuangjingzi	232	####	0.10	####		0.21	0.57	0.11	0.02	1.27	3.76	4.15	0.02			12.10	23.00	2.06	6.91	1.24	0.33	0.98	0.14	0.54	0.09	0.28	0.05	0.32	0.05	2.67	1.67	63.8	92.9
47	Inner Mongolia	Solonker-Xilamulun	2			D105-4	Granite	Shuangjingzi	232	####	0.31	####		0.31	1.45	0.49	0.03	1.72	3.17	5.14	0.09			63.50	114.0	11.10	34.90	4.78	0.82	3.67	0.42	1.66	0.28	0.88	0.12	0.81	0.10	9.01	6.01	255	274
48	Inner Mongolia	Solonker-Xilamulun	2			D103-1	Granite	Shuangjingzi	232	####	0.20	####		0.09	1.33	0.34	0.02	1.22	3.78	5.07	0.06			33.50	59.60	5.89	19.00	2.83	0.57	2.27	0.30	1.46	0.27	0.85	0.11	0.72	0.11	8.84	4.39	250	209
49	Inner Mongolia	Solonker-Xilamulun	2			D100-1	Granite	Shuangjingzi	232	####	0.23	####		0.80	0.93	0.27	0.04	0.76	3.56	4.99	0.06			25.70	50.60	4.84	15.90	2.67	0.44	2.28	0.35	1.78	0.32	0.99	0.15	0.99	0.15	10.60	3.87	295	116
50	Inner Mongolia	Solonker-Xilamulun	2			D98-2	Granite	Shuangjingzi	232	####	0.27	####		0.75	1.07	0.32	0.03	1.32	3.09	4.67	0.07			34.80	60.20	6.11	19.80	3.11	0.55	2.27	0.34	1.63	0.29	0.92	0.14	0.96	0.15	10.00	3.87	177	208
51	Inner Mongolia	Solonker-Xilamulun	2			D97-1	Granite	Shuangjingzi	232	####	0.22	####		0.35	1.20	0.25	0.03	1.46	3.35	5.31	0.05			49.80	91.80	9.00	28.90	4.54	0.58	3.32	0.46	2.03	0.35	1.07	0.13	0.88	0.13	11.40	9.78	243	206
52	Inner Mongolia	Solonker-Xilamulun	2			I32	Garnet monzogranite	Yagan	228	####	0.32	####		0.79	1.13	0.13	0.50	2.17	3.95	8.22	0.14			90.38	200.3	19.91	73.41	11.26	2.09	9.59	1.12	6.09	1.11	3.14	0.42	2.18	0.23	26.04		384	742.7
53	Inner Mongolia	Solonker-Xilamulun	2			I09	Qtz-monzonite	Yagan	228	####	0.56	####		2.02	1.99	0.14	1.21	2.08	5.05	5.69	0.32			133.9	252.0	25.78	101.60	15.99	2.77	15.20	1.67	9.36	1.84	4.74	0.68	4.29	0.67	45.49		273	1044
54	Inner Mongolia	Solonker-Xilamulun	2			N07	Qtz-monzonite	Yagan	228	####	0.47	####		1.03	1.68	0.12	0.76	2.02	4.93	6.76	0.17			128.65	231.4	24.15	90.65	14.81	2.67	14.32	1.47	7.38	1.74	4.47	0.57	4.03	0.57	39.74		251	984
55	Inner Mongolia	Solonker-Xilamulun	2			7	Bi-monzogranite	Yagan	228	####	0.18	####		0.75	0.70	0.03	0.24	1.08	3.48	5.95	0.04			69.28	127.5	11.67	39.39	6.45	0.57	4.79	0.42	1.48	0.21	0.60	0.10	0.23	0.10	3.34		172	82.0
56	Inner Mongolia	Solonker-Xilamulun	2			113	Qtz-monzonite	Yagan	228	####	0.06	####		0.09	0.29	0.09	0.09	0.09	3.91	4.71	0.01			4.79	5.72	0.74	3.33	1.02	0.41	1.92	0.42	3.26	0.79	2.63	0.41	3.62	0.61	22.74		337	235
57	Inner Mongolia	Solonker-Xilamulun	2			BP20-1	Leucogranite	Halatu	228	####	0.05	####				0.05	0.08	1.08	3.69	5.22	0.12	0.03		7.40	12.00	1.50	5.80	1.70	0.50	1.00	0.14	0.60	0.13	0.30	0.04	0.22		2.40		310	103
58	Inner Mongolia	Solonker-Xilamulun	2			BP21-1	Qtz monzonite	Halatu	228	####	0.18	####				0.04	0.20	1.10	3.79	5.19	0.15	0.47		16.00	38.00	4.00	15.00	3.60	0.50	2.90	0.38	1.50	0.30	0.90	0.10	0.50		6.10		231	118
59	Inner Mongolia	Solonker-Xilamulun	2			BP27-1	Qtz monzonite	Halatu	228	####	0.20	####				0.07	0.30	1.36	4.06	3.58	0.14	0.31		24.00	40.00	4.50	18.00	4.20	0.80	3.60	0.47	2.10	0.47	1.20	0.20	1.00		11.40		283	128
60	Inner Mongolia	Solonker-Xilamulun	2			BP24-1	Granodiorite	Halatu	228	####	0.20	####				0.07	0.48	1.08	3.53	4.33	0.18	1.31		54.00	88.00	9.20	35.00	6.00	0.80	5.00	0.64	2.70	0.62	1.60	0.23	1.20		13.70		226	146
61	Eastern Xilamulun	Eastern Xilamulun	2			HL-1-1	Bi granite			####	0.37	####	2.79			0.05	0.77	1.84	3.80	3.86	0.11	0.79		52.90	82.70	7.59	23.40	3.07	0.83	2.21	0.29	1.52	0.29	0.78	0.12	0.76	0.12	8.30	0.96	96.2	621
62	Eastern Xilamulun	Eastern Xilamulun	2			HL-1-2	Bi granite			####	0.29	####	2.25			0.04	0.59	1.83	3.23	4.50	0.07	0.43		66.70	97.50	8.43	26.90	3.88	0.99	2.94	0.38	2.12	0.41	1.09	0.16	1.06	0.16	11.60	1.49	92.6	618
63	Eastern Xilamulun	Eastern Xilamulun	2			HL-1-3	Bi granite			####	0.51	####	3.73			0.07	1.20	3.28	3.97	3.66	0.18	0.55		36.10	66.80	7.81	29.30	5.23	1.30	3.98	0.58	3.01	0.57	1.54	0.23	1.49	0.22	16.70	0.88	86.5	645
64	Eastern Xilamulun	Eastern Xilamulun	2			HL-1-4	Bi granite			####	0.41	####	3.36			0.04	0.74	1.70	3.69	5.18	0.12	0.81		93.10	161.0	13.00	38.10	4.49	1.05	2.55	0.33	1.66	0.30	0.89	0.12	0.90	0.13	9.50	1.07	124	704
65	Eastern Xilamulun	Eastern Xilamulun	2			HL-1-5	Bi granite			####	0.37	####	3.20			0.05	0.73	2.58	4.01	3.14	0.11	1.12		35.60	69.00	6.75	22.40	3.10	0.85	2.14	0.31	1.72	0.34	0.96	0.16	1.14	0.18	11.10	1.80	95.8	552
66	Eastern Xilamulun	Eastern Xilamulun	2			HL-5-1	Granodiorite			####	0.46	####	4.16			0.06	1.12	2.83	4.17	2.90	0.17	0.80		48.40	93.50	9.07	29.70	4.50	1.05	3.14	0.42	2.47	0.46	1.34	0.20	1.40	0.21	14.40	1.85	74.6	706
67	Eastern Xilamulun	Eastern Xilamulun	2			HL-5-2	Qtz diorite			####	1.02	####	7.40			0.11	3.07	6.39	4.10	1.80	0.31	0.89		29.20	57.50	6.65	26.30	5.05	1.45	4.34	0.58	3.19	0.58	1.53	0.21	1.31	0.21	16.40	2.95	64.8	849
68	Eastern Xilamulun	Eastern Xilamulun	2			HL-5-3	Qtz diorite			####	0.96	####	7.03			0.10	2.90	6.28	4.13	1.72	0.30	1.07		26.30	53.50	6.32	25.10	4.86	1.53	4.20	0.59	3.12	0.57	1.46	0.20	1.24	0.20	15.90	2.64	62.4	878
69	Eastern Xilamulun	Eastern Xilamulun	2			HL-5-4	Diorite			####	1.40	####	####			0.15	4.14	8.07	3.44	1.92	0.41	1.67		22.80	58.10	7.92	34.50	7.21	2.02	6.49	0.87	4.46	0.81	1.95	0.27	1.58	0.22	22.10	2.73	65.8	1020
70	Eastern Xilamulun	Eastern Xilamulun	2			HL-5-5	Diorite			####	1.40	####	####			0.14	4.11	8.15	3.55	2.05	0.42	1.57		24.50	59.40	8.04	33.60	6.75	2.07	6.04	0.81	4.20	0.81	1.87	0.26	1.56	0.22	21.10	8.36	78.5	986
71	Eastern Xilamulun	Eastern Xilamulun	2			YH48	Monzogranite			####	0.09	####	0.95			0.04	0.13	1.13	4.03	3.57	0.02	0.12		8.90	17.60	2.14	7.78	1.93	0.36	2.48	0.45	3.14	0.68	1.99	0.31	1.94	0.31	23.90	2.69	94.5	334
72	Eastern Xilamulun	Eastern Xilamulun	2			P4B16-2	Monzogranite			####	0.20	####	1.19			0.03	0.36	2.24	5.33	1.81	0.07	0.55		6.02	12.00	1.54	5.93	1.35	0.41	1.15	0.19	1.07	0.20	0.58	0.10	0.64	0.10	7.60	1.46	47.6	586
73	Eastern Xilamulun	Eastern Xilamulun	2			B3164-1	Monzogranite			####	0.10	####	0.98			0.07	0.14	0.99	3.90	3.90	0.03	0.20		3.56	6.91	0.73	2.53	0.56	0.25	0.66	0.11	0.69	0.14	0.38	0.06	0.38	0.06	4.63	3.69	141	192
74	Eastern Xilamulun	Eastern Xilamulun	2			HL-3-1	Granodiorite			####	0.43	####	3.21			0.13	0.82	2.31	4.69	3.12	0.13	1.04		27.50	54.60	5.93	21.70	3.93	1.09	3.45	0.52	3.26	0.69	1.94	0.31	2.04	0.32	19.50	5.64	114	335
75	Eastern Xilamulun	Eastern Xilamulun	2			HL-3-3	Granodiorite			####	0.58	####	4.04			0.07	1.34	1.94	5.43	2.95	0.22	1.90		33.50	76.80	8.39	30.00	5.12	1.01	4.21	0.67	4.35	0.95	2.90	0.47	3.28	0.51	28.00	3.26	93.8	377
76	Eastern Xilamulun	Eastern Xilamulun	2			HL-3-4	Granodiorite			####	0.21	####	2.26			0.05	0.32	1.40	3.75	4.44	0.06	0.65		35.00	68.80	6.78	22.90	3.87	0.82	3.35	0.50	2.73	0.55	1.67	0.25	1.74	0.28	16.50	3.49	158.5	210
77	Eastern Xilamulun	Eastern Xilamulun	2			HL-3-5	Granodiorite			####	0.21	####	2.26			0.05	0.32	1.42	3.76	4.46	0.07	0.67		39.30	73.30	7.63	24.70	4.30	0.92	3.39	0.48	2.94	0.56	1.70	0.27	1.84	0.28	16.60	3.83	161	216
78	Eastern Xilamulun	Eastern Xilamulun	2			HL-2-1	Rhyolite			####	0.13	####	2.17			0.01	0.04	0.03	3.13	4.91	0.01	0.59		59.80	140.0	15.35	58.60	11.85	0.13	10.35	1.83	12.55	2.74	8.52	1.34	9.09	1.38	76.70	1.94	221	7.70
79	Eastern Xilamulun	Eastern Xilamulun	2			HL-2-2	Rhyolite			####	0.13	####	2.88			0.13	0.02	0.05	2.74	4.61	0.01	1.05		63.40	147.5	17.15	65.10	14.40	0.21	14.40	2.48	15.45	3.21	9.27	1.40	9.17	1.40	81.10	2.89	220	11.30
80	Eastern Xilamulun	Eastern Xilamulun	2			HL-2-3	Rhyolite			####	0.14	####	2.27			0.03	0.04	0.02	3.09	4.88	0.02	0.73		54.50	139.5	14.05	52.80	10.30	0.13	8.85	1.46	9.90	2.29	7.68	1.29	8.62	1.37	65.20	2.00	226	6.70
81	Eastern Xilamulun	Eastern Xilamulun	2			HL-2-4	Rhyolite			####	0.13	####	1.85			0.02	0.02	0.01	3.00	5.11	0.01	0.64		52.60	122.5	14.00	53.80	11.05	0.15	10.40	1.86	13.10	2.90	9.07	1.48	9.50	1.48	83.00	2.04	240	6.50
82	Eastern Xilamulun	Eastern Xilamulun	2			HL-6-1	Rhyolite			####	0.20	####	3.17			0.05	0.28	1.62	5.27	2.89	0.06	0.41		21.40	43.80	4.92	18.50	4.16	1.25	4.30	0.69	4.50	0.92	2.69	0.42	2.90	0.45	26.70	3.09	85	213
83	Eastern Xilamulun	Eastern Xilamulun	2			HL-6-2	Rhyolite			####	0.19	####	3.25			0.05	0.27	1.59	4.98	2.94	0.06	0.44		20.60	42.60	4.82	18.20	4.22	1.12	4.16	0.68	4.31	0.92	2.66	0.42	2.84	0.44	26.40	3.17	87.6	205
84	Eastern Xilamulun	Eastern Xilamulun	2			HL-6-3	Rhyolite			####	0.19	####	2.69			0.06	0.29	1.35	5.31	3.34	0.06	0.46		22.60	46.20	5.24	20.10	4.40	0.98	4.39	0.72	4.63	0.96	2.76	0.44	2.89	0.44	28.00	2.76	90.7	173
85	Eastern Xilamulun	Eastern Xilamulun	2			HL-6-4	Rhyolite			####	0.11	9.98	2.95			0.07	0.15	2.34	3.82	0.95	0.03	0.38		17.40	35.50	4.14	15.20	3.50	0.54	3.69	0.61	3.94	0.83	2.39	0.36	2.41	0.38	23.80	0.48	21.5	184
86	Erguna Massif	Erguna belt	3	122.09	52.06	13ER13-6	Gabbro–diorite	Mangui	227	####	1.04	####	7.89			0.12	6.72	7.45	3.21	1.65	0.19	3.24	63	21.70	45.70	5.51	22.30	4.74	1.42	4.37	0.70	4.07	0.78	2.19	0.33	2.12	0.31	23.60	5.23	49	365
87	Erguna Massif	Erguna belt	3			13ER13-7	Granitoid		228	####	1.08	####	8.39			0.12	6.93	7.60	3.00	2.06	0.20	3.00	62	22.80	46.60	5.51	22.20	4.50	1.38	4.16	0.66	4.04	0.75	2.12	0.32	1.99	0.30	25.20	6.26	68.1	339
88	Erguna Massif	Erguna belt	3			13ER13-8	Granitoid		229	####	1.10	####	8.04			0.12	6.75	7.12	3.20	1.67	0.19	2.88	62	22.80	46.80	5.59	21.90	4.49	1.36	4.23	0.65	3.98	0.79	2.26	0.33	2.10	0.30	23.20	4.91	49.3	370
89	Erguna Massif	Erguna belt	3			13ER13-9	Granitoid		230	####	0.96	####	7.79			0.11	6.43	6.85	3.32	1.77	0.21	3.16	62	21.70	45.10	5.33	21.90	4.34	1.32	4.31	0.67	3.82	0.74	2.19	0.34	2.24	0.29	22.10	5.43	53.8	362
90	Erguna Massif	Erguna belt	3	120.03	50.86	ER12-1	Granitoid		229	####	0.15	####	1.14			0.03	0.09	0.71	3.34	4.60	0.05	2.81	14	17.10	69.30	4.02	13.00	2.41	0.64	2.18	0.32	1.86	0.37	1.14	0.19	1.27	0.20	10.80	2.30	132	106
91	Erguna Massif	Erguna belt	3			ER22-2	Granitoid		229	####	0.15	####	0.78			0.02	0.11	0.80	3.77	4.47	0.05	2.06	22	15.20	47.10	4.17	14.10	3.08	0.61	2.57	0.41	2.33	0.49	1.46	0.22	1.51	0.23	13.80	2.76	140	110
92	Erguna Massif	Erguna belt	3			ER22-4	Granitoid		229	####	0.15	####	0.95			0.02	0.12	0.81	3.79	4.69	0.05	2.28	20	17.30	12.50	4.57	14.80	2.48	0.60	1.94	0.31	1.80	0.37	1.14	0.19	1.24	0.20	10.40	4.87	142	108
93	Erguna Massif	Erguna belt	3			ER22-7	Granitoid		229	####	0.16	####	0.96			0.02	0.10	0.83	3.77	4.64	0.05	2.55	17	7.62	52.70	2.19	7.70	1.93	0.58	2.00	0.35	2.23	0.47	1.46	0.23	1.60	0.24	13.80	2.45	144	112
94	Erguna Massif	Erguna belt	3			13ER42-1	Granitoid		229	####	0.14	####	1.14			0.03	0.15	0.66	3.72	5.02	0.04	0.64	21	26.50	56.50	5.54	18.80	3.43	0.59	2.38	0.39	2.22	0.42	1.24	0.18	1.17	0.17	13.10	2.85	156	112
95	Erguna Massif	Erguna belt	3			13ER42-2	Granitoid		229	####	0.15	####	1.28			0.04	0.15	0.73	3.64	4.89	0.04	0.66	19	28.70	67.90	6.09	20.60	3.56	0.63	2.82	0.41	2.40	0.44	1.29	0.17	1.30	0.19	14.30	3.50	150	107
96	Erguna Massif	Erguna belt	3			13ER42-3	Granitoid		229	####	0.16	####	1.42			0.04	0.17	0.67	3.72	4.87	0.04	0.92	19	29.20	64.30	6.04	20.70	3.50	0.61	2.73	0.40	2.33	0.44	1.24	0.18	1.24	0.17	14.10	3.88	153	110
97	Erguna Massif	Erguna belt	3			13ER45-1	Granitoid		224	####	0.25	####	1.06			0.01	0.20	1.06	3.94	4.48	0.07	1.02	27	24.60	56.60	5.17	18.60	3.63	0.73	2.90	0.48	2.76	0.53	1.46	0.22	1.62	0.24	16.40	3.64	185	301
98	Erguna Massif	Erguna belt	3			13ER45-3	Granitoid		224	####	0.26	####	1.52			0.01	0.25	1.08	4.02	4.67	0.07	1.04	25	27.30	61.80	5.67	20.40	3.74	0.65	3.23	0.51	2.85	0.55	1.61	0.24	1.74	0.25	17.40	4.34	197	279

No.	Location	Magmatic belt	Group	Longitude	Latitude	Sample	Rock type	Pluton	Age（Ma）	SiO_2	TiO_2	Al_2O	TFe_2	Fe_2O	FeO	MnC	MgC	CaO	Na_2C	K_2O	P_2O_5	LOI	$Mg^{\#}$	La	Ce	Pr	Nd	Sm	Eu	Gd	Tb	Dy	Ho	Er	Tm	Yb	Lu	Y	Cs	Rb	Sr
99	Erguna Massif	Erguna belt	3			13ER46-1	Granitoid		228	####	0.35	####	2.52			0.10	0.83	4.05	4.64	1.94	0.15	2.22	39	20.20	41.30	4.62	17.50	3.18	0.93	2.51	0.35	2.02	0.36	1.01	0.14	0.88	0.14	12.10	7.18	61	658
100	Erguna Massif	Erguna belt	3			13ER46-4	Granitoid		228	####	0.45	####	3.35			0.04	0.93	4.10	4.32	1.30	0.17	2.18	35	22.00	45.40	5.19	20.30	3.72	0.98	2.86	0.41	2.21	0.41	1.13	0.16	1.03	0.15	13.80	1.23	34.7	1061
101	Erguna Massif	Erguna belt	3			13ER46-5	Granitoid		228	####	0.44	####	3.14			0.04	0.88	4.24	4.42	1.02	0.17	1.82	36	29.50	59.90	6.56	24.60	4.26	1.06	3.14	0.43	2.30	0.41	1.14	0.18	1.10	0.15	13.80	1.00	26.9	1179
102	Erguna Massif	Erguna belt	3			13ER34-1	Granitoid		204	####	0.18	####	4.39			0.07	6.25	####	1.37	0.19	0.02	1.62	74	2.74	5.50	0.70	3.17	0.76	0.48	0.83	0.14	0.95	0.19	0.49	0.07	0.46	0.06	11.00	0.35	4.23	486
103	Erguna Massif	Erguna belt	3			13ER34-2	Granitoid		204	####	0.19	####	4.85			0.08	6.82	####	1.23	0.25	0.02	1.84	74	2.89	5.98	0.74	3.17	0.89	0.54	0.93	0.15	1.04	0.24	0.61	0.08	0.51	0.07	6.68	0.53	6.99	479
104	Erguna Massif	Erguna belt	3			13ER34-3	Granitoid		204	####	0.20	####	4.95			0.08	6.96	####	1.22	0.21	0.02	1.66	74	2.61	5.37	0.72	3.27	0.90	0.53	0.96	0.16	1.07	0.19	0.58	0.08	0.52	0.07	7.12	0.44	4.92	460
105	Erguna Massif	Erguna belt	3			13ER34-4	Granitoid		204	####	0.20	####	4.79			0.08	6.80	####	1.22	0.23	0.02	1.96	74	2.18	4.52	0.58	2.66	0.75	0.53	0.86	0.16	0.94	0.19	0.51	0.07	0.46	0.08	7.96	0.57	6.46	476
106	Erguna Massif	Erguna belt	3			12ER30-5	Granitoid		200	####	1.83	####	####			0.13	4.29	6.60	3.76	1.10	0.25	2.08	41	26.00	57.80	7.90	35.00	7.99	1.90	7.67	1.18	7.00	1.38	3.80	0.52	3.44	0.50	40.30	2.48	38	640
107	Erguna Massif	Erguna belt	3			12ER30-6	Granitoid		200	####	1.73	####	####			0.12	4.13	6.67	3.79	1.09	0.25	2.02	42	24.40	54.20	7.44	33.40	7.74	1.85	7.50	1.13	6.67	1.31	3.60	0.51	3.27	0.46	37.40	2.38	36.2	620
108	Erguna Massif	Erguna belt	3			12ER30-8	Granitoid		200	####	1.81	####	####			0.13	4.08	6.11	3.74	1.25	0.20	2.80	42	21.00	48.70	6.82	31.30	7.49	1.71	7.37	1.15	6.68	1.34	3.72	0.53	3.32	0.50	39.20	2.48	46.6	690
109	Erguna Massif	Erguna belt	3			12ER30-9	Granitoid		200	####	1.80	####	####			0.12	4.03	6.42	3.54	1.05	0.22	2.42	42	22.00	50.80	7.09	32.20	7.55	1.81	7.37	1.12	6.63	1.32	3.61	0.48	3.29	0.48	36.90	2.55	37.4	627
110	Erguna Massif	Erguna belt	3			13ER8-1	Granitoid		204	####	1.38	####	####			0.16	5.34	7.24	3.65	1.68	0.31	2.04	50	23.20	58.30	7.97	35.30	8.42	1.86	7.58	1.25	7.95	1.58	4.12	0.62	3.80	0.59	44.60	2.10	45.4	398
111	Erguna Massif	Erguna belt	3			13ER8-2	Granitoid		204	####	1.83	####	####			0.17	4.93	8.28	3.46	1.12	0.38	1.36	43	32.60	88.20	12.10	53.40	12.50	2.23	11.30	1.83	11.30	2.25	6.12	0.87	5.70	0.81	65.50	1.63	26.1	438
112	Erguna Massif	Erguna belt	3			13ER8-4	Granitoid		204	####	1.88	####	####			0.18	4.97	8.49	3.44	1.07	0.37	1.28	42	34.00	91.90	12.70	56.70	13.60	2.32	11.90	1.98	12.40	2.44	6.55	0.98	5.97	0.85	69.70	1.34	22.2	416
113	Erguna Massif	Erguna belt	3			13ER8-5	Granitoid		204	####	1.86	####	####			0.17	4.79	8.35	3.53	1.12	0.36	1.26	42	33.80	92.70	12.40	54.60	12.50	2.30	11.50	1.91	12.30	2.33	6.44	0.91	6.00	0.84	67.30	1.49	24.3	435
114	Erguna Massif	Erguna belt	3			13ER8-6	Granitoid		204	####	0.89	####	8.36			0.12	5.27	7.39	3.12	1.06	0.16	1.38	56	25.00	47.80	5.81	23.60	4.91	1.30	4.72	0.75	4.68	0.95	2.62	0.40	2.51	0.35	28.00	1.75	27.5	373
115	Erguna Massif	Erguna belt	3			13ER8-7	Granitoid		204	####	0.93	####	8.52			0.12	5.34	7.48	3.08	0.94	0.18	1.60	55	22.90	46.70	5.86	23.80	5.24	1.30	4.97	0.76	4.92	0.96	2.74	0.43	2.72	0.38	30.00	1.46	21.7	360
116	Erguna Massif	Erguna belt	3			13ER8-10	Granitoid		204	####	1.00	####	9.42			0.13	6.37	8.00	2.96	1.21	0.20	1.68	57	17.90	38.50	4.79	19.90	4.26	1.26	4.07	0.69	4.15	0.84	2.43	0.35	2.29	0.32	24.50	2.01	31.2	367
117	Erguna Massif	Erguna belt	3			12ER34-1	Granitoid		206	####	0.31	####	2.29			0.04	0.41	1.30	3.77	5.14	0.07	0.46	26	43.60	76.60	8.45	29.50	5.33	0.85	4.49	0.72	4.23	0.86	2.43	0.34	2.31	0.34	26.90	1.83	147	120
118	Erguna Massif	Erguna belt	3			12ER34-2	Granitoid		206	####	0.30	####	2.12			0.04	0.40	1.37	3.98	4.80	0.06	0.60	27	36.00	67.90	6.82	25.00	4.81	0.87	4.17	0.67	4.12	0.81	2.39	0.35	2.29	0.34	25.30	1.05	125	135
119	Erguna Massif	Erguna belt	3			12ER34-3	Granitoid		206	####	0.29	####	2.07			0.05	0.38	1.20	3.76	5.19	0.06	0.62	27	38.00	67.40	7.36	26.70	5.13	0.78	4.74	0.77	4.61	0.96	2.87	0.39	2.73	0.41	29.40	1.06	127	134
120	Erguna Massif	Erguna belt	3			11ER26-2	Granitoid		206	####	0.30	####	2.41			0.52	0.63	2.51	3.84	3.52	0.10	0.44	34	33.00	61.00	6.92	23.80	4.53	0.87	4.02	0.66	3.94	0.80	2.47	0.37	2.39	0.37	25.30	4.01	142	353
121	Erguna Massif	Erguna belt	3			11ER26-3	Granitoid		206	####	0.26	####	2.13			0.01	0.54	2.33	3.68	3.74	0.08	0.57	33	27.40	51.10	6.03	21.80	4.16	0.87	3.80	0.61	3.67	0.77	2.23	0.33	2.42	0.33	23.70	3.81	141	346
122	Erguna Massif	Erguna belt	3			12ER15-1	Granitoid		206	####	0.27	####	2.30			0.05	0.61	2.37	3.90	3.43	0.09	0.40	34	31.60	59.70	6.68	23.80	4.46	0.72	3.54	0.55	3.33	0.64	1.85	0.28	2.01	0.32	20.80	3.37	124	287
123	Erguna Massif	Erguna belt	3			12ER15-2	Granitoid		206	####	0.26	####	2.20			0.05	0.60	2.44	3.92	3.34	0.08	0.42	35	21.30	38.70	4.68	17.40	3.75	0.71	3.43	0.55	3.32	0.64	1.91	0.29	1.97	0.31	21.20	3.03	134	306
124	Erguna Massif	Erguna belt	3			12ER16-1	Granitoid		206	####	0.32	####	2.65			0.05	0.78	2.79	3.88	3.05	0.11	0.82	37	29.20	55.40	6.25	22.70	4.31	0.92	3.46	0.51	2.81	0.53	1.54	0.23	1.46	0.23	17.10	2.87	96.7	466
125	Erguna Massif	Erguna belt	3			12ER16-2	Granitoid		206	####	0.32	####	2.62			0.05	0.78	2.69	3.78	3.44	0.11	0.74	37	24.80	48.30	5.31	19.20	3.81	0.95	2.92	0.45	2.63	0.51	1.37	0.22	1.38	0.20	15.90	3.11	114	481
126	Erguna Massif	Erguna belt	3			11ER27-1	Granitoid		206	####	0.34	####	2.70			0.06	1.04	3.57	3.86	3.49	0.12	0.82	43	33.20	64.20	7.44	27.10	5.16	1.10	4.32	0.68	3.76	0.71	2.13	0.29	2.04	0.29	22.20	2.99	121	485
127	Erguna Massif	Erguna belt	3			11ER27-2	Granitoid		206	####	0.34	####	2.69			0.55	0.82	2.90	3.80	3.38	0.12	0.84	38	30.80	60.50	6.82	24.40	4.63	1.05	4.19	0.62	3.47	0.69	2.07	0.30	2.02	0.29	21.10	2.95	116	469
128	Erguna Massif	Erguna belt	3			12ER17-2	Granitoid		205	####	0.27	####	2.35			0.05	0.63	1.95	3.96	3.61	0.08	1.12	35	30.30	57.30	6.55	23.00	4.47	0.76	3.65	0.58	3.32	0.65	1.87	0.29	1.92	0.31	21.30	3.71	134	328
129	Erguna Massif	Erguna belt	3			12ER17-3	Granitoid		205	####	0.25	####	2.19			0.05	0.60	2.20	4.02	3.15	0.08	0.94	35	19.70	37.50	4.38	16.20	3.62	0.71	3.16	0.53	3.10	0.63	1.84	0.29	2.01	0.31	20.10	3.05	127	316
130	Erguna Massif	Erguna belt	3			12ER17-5	Granitoid		205	####	0.25	####	2.19			0.05	0.60	2.14	3.74	3.24	0.07	0.88	35	32.10	61.30	6.84	24.70	4.52	0.72	3.72	0.60	3.47	0.67	2.07	0.32	2.17	0.33	21.70	2.69	127	306
131	Erguna Massif	Erguna belt	3			12ER19-1	Granitoid		205	####	0.20	####	1.94			0.02	0.34	1.28	3.78	3.74	0.06	0.54	26	23.20	51.90	4.05	13.00	2.32	0.48	1.85	0.35	2.12	0.45	1.28	0.20	1.29	0.21	14.20	1.91	119	128
132	Erguna Massif	Erguna belt	3			12ER19-2	Granitoid		205	####	0.19	####	1.81			0.02	0.30	1.30	3.67	3.96	0.06	0.50	25	27.90	57.10	4.84	15.50	2.59	0.54	1.97	0.37	2.26	0.47	1.34	0.21	1.29	0.20	13.80	1.69	122	139
133	Erguna Massif	Erguna belt	3			12ER19-3	Granitoid		205	####	0.19	####	1.77			0.02	0.32	1.18	3.69	3.94	0.05	0.50	26	32.90	57.00	6.39	21.40	3.59	0.53	2.90	0.45	2.54	0.53	1.60	0.26	1.83	0.29	16.10	1.52	123	127
134	Erguna Massif	Erguna belt	3			12ER19-4	Granitoid		205	####	0.18	####	1.70			0.02	0.29	1.16	3.82	4.24	0.04	0.50	25	32.10	65.00	6.12	21.00	3.53	0.51	2.70	0.43	2.51	0.50	1.46	0.24	1.65	0.25	15.10	1.36	127	135
135	Erguna Massif	Erguna belt	3			12ER9-1	Granitoid		202	####	0.45	####	3.11			0.05	0.71	1.90	3.49	2.79	0.12	2.86	31	37.70	72.10	7.91	27.30	5.01	1.00	4.12	0.66	3.86	0.76	2.12	0.35	2.38	0.36	25.00	4.56	130	204
136	Erguna Massif	Erguna belt	3			12ER9-2	Granitoid		202	####	0.36	####	2.48			0.05	0.55	2.77	4.04	2.56	0.10	3.58	31	35.20	67.60	7.42	26.30	4.85	1.06	3.97	0.64	3.73	0.73	2.07	0.32	2.30	0.33	23.60	4.82	137	145
137	Erguna Massif	Erguna belt	3			12ER27-1	Granitoid		197	####	0.38	####	3.05			0.05	1.13	2.94	4.30	1.88	0.12	0.96	42	41.60	75.00	7.02	22.90	3.03	1.09	1.89	0.26	1.42	0.27	0.78	0.13	0.85	0.14	9.84	0.50	38.6	330
138	Erguna Massif	Erguna belt	3			12ER27-2	Granitoid		197	####	0.42	####	3.08			0.05	0.93	2.32	4.52	2.70	0.12	1.34	37	51.80	92.90	8.94	28.20	3.71	1.23	2.35	0.33	1.79	0.35	1.00	0.16	1.13	0.18	11.30	0.76	79.9	319
139	Erguna Massif	Erguna belt	3			12ER27-3	Granitoid		197	####	0.47	####	3.68			0.06	1.03	2.91	4.32	2.08	0.14	1.18	36	45.10	82.00	7.91	26.00	3.77	1.21	2.52	0.34	1.88	0.37	1.09	0.16	1.25	0.19	12.90	0.50	44.5	335
140	Erguna Massif	Erguna belt	3			12ER27-4	Granitoid		197	####	0.38	####	2.96			0.05	0.86	2.27	4.70	2.20	0.11	1.28	37	41.10	72.50	7.12	24.20	3.28	1.17	2.22	0.33	1.82	0.39	1.18	0.18	1.34	0.21	13.10	0.88	58.1	386
141	Erguna Massif	Erguna belt	3			13ER21-1	Granitoid		195	####	0.56	####	4.40			0.06	1.43	3.63	4.47	1.77	0.22	1.34	39	60.40	111.0	10.80	36.60	5.32	1.41	3.81	0.47	2.48	0.49	1.31	0.19	1.32	0.23	14.20	2.22	40.8	525
142	Erguna Massif	Erguna belt	3			13ER21-5	Granitoid		195	####	0.94	####	6.85			0.10	3.90	6.58	3.79	1.59	0.19	1.46	53	14.40	32.20	4.49	19.40	4.22	1.24	3.87	0.55	3.45	0.63	1.78	0.29	1.87	0.29	21.20	1.65	31.9	544
143	Erguna Massif	Erguna belt	3			12ER24-1	Granitoid		197	####	0.31	####	3.32			0.14	0.34	0.86	5.80	3.23	0.08	0.82	17	117.0	212.0	23.00	78.70	11.80	1.46	7.96	1.17	6.68	1.29	3.74	0.58	4.09	0.65	39.20	3.53	123	105
144	Erguna Massif	Erguna belt	3			12ER24-2	Granitoid		197	####	0.24	####	2.78			0.12	0.29	0.86	5.76	3.30	0.07	0.56	17	124.0	223.0	23.50	80.00	11.80	1.49	7.55	1.06	5.87	1.16	3.45	0.50	3.49	0.56	34.30	3.72	117	99.5
145	Erguna Massif	Erguna belt	3			12ER24-3	Granitoid		197	####	0.33	####	3.68			0.16	0.38	0.99	5.65	3.42	0.08	0.60	17	134.0	249.0	25.90	89.50	12.90	1.44	8.25	1.14	5.99	1.14	3.44	0.52	3.49	0.58	34.60	4.66	132	106
146	Erguna Massif	Erguna belt	3			12ER23-1	Granitoid		196	####	0.12	####	1.18			0.05	0.15	0.78	4.29	4.20	0.03	0.34	20	24.50	50.50	5.46	19.10	4.09	0.43	3.41	0.62	3.91	0.81	2.52	0.38	2.75	0.41	25.60	3.09	124	59.90
147	Erguna Massif	Erguna belt	3			12ER23-2	Granitoid		196	####	0.12	####	1.19			0.05	0.15	0.54	4.20	4.23	0.03	0.36	20	26.70	55.70	6.01	21.10	4.27	0.45	3.77	0.71	4.45	0.89	2.71	0.45	2.96	0.46	29.10	3.15	128	62.30
148	Erguna Massif	Erguna belt	3			12ER23-3	Granitoid		196	####	0.12	####	1.14			0.05	0.13	0.53	4.23	4.30	0.03	0.38	18	23.70	48.50	5.47	18.90	3.98	0.53	4.06	0.74	4.80	1.04	3.18	0.48	3.36	0.52	33.30	3.40	135	77.60
149	Erguna Massif	Erguna belt	3			MZ18-2	Granitoid		180	####	0.24	####	1.93			0.06	0.35	0.94	4.26	3.91	0.05	0.23	26	36.40	—	8.22	29.40	6.04	0.84	5.61	0.91	5.58	1.19	3.58	0.57	4.01	0.62	32.20	6.08	175	107
150	Erguna Massif	Erguna belt	3			12MZ2-1	Granitoid		180	####	0.25	####	1.76			0.05	0.32	0.90	4.13	4.03	0.05	0.32	26	38.40	76.80	8.63	31.50	5.97	0.76	5.21	0.89	5.73	1.19	3.77	0.61	4.42	0.69	40.00	6.30	202	101
151	Erguna Massif	Erguna belt	3			12MZ2-2	Granitoid		180	####	0.23	####	1.60			0.05	0.30	0.95	4.26	3.99	0.05	0.30	27	28.00	57.60	6.60	24.60	5.06	0.76	4.60	0.80	5.24	1.09	3.44	0.54	3.84	0.62	36.80	5.37	185	103
152	Erguna Massif	Erguna belt	3			12MZ2-3	Granitoid		180	####	0.23	####	1.57			0.05	0.32	0.85	4.19	4.10	0.04	0.28	29	29.90	60.40	6.80	24.90	5.08	0.75	4.45	0.79	5.13	1.05	3.44	0.54	3.82	0.62	35.50	5.22	198	105
153	Erguna Massif	Erguna belt	3			11ER1-2	Granitoid		186	####	0.05	####	0.88			0.01	0.30	0.75	3.01	4.57	0.02	0.58	40	16.60	44.10	5.12	20.30	5.22	0.10	5.86	1.07	7.44	1.55	4.63	0.74	5.17	0.76	47.80	4.69	251	14.00
154	Erguna Massif	Erguna belt	3			11ER1-3	Granitoid		186	####	0.06	####	0.88			0.01	0.58	1.44	3.09	4.57	0.02	0.65	57	18.80	47.90	5.87	22.50	5.97	0.11	6.42	1.20	8.35	1.81	5.39	0.91	6.14	0.96	56.60	5.30	238	15.7
155	Erguna Massif	Erguna belt	3			11ER1-4	Granitoid		186	####	0.06	####	0.79			0.01	0.08	0.21	2.86	4.69	0.02	0.90	17	21.30	51.40	6.37	24.80	6.65	0.14	6.38	1.10	6.73	1.35	3.81	0.60	4.15	0.62	40.20	5.93	255	16.4
156	Erguna Massif	Erguna belt	3			HL-6-5	Rhyolite			####	0.17	####	2.88			0.06	0.27	1.72	5.24	2.30	0.06	0.41		21.70	45.60	5.19	19.70	4.16	0.90	4.42	0.75	4.70	0.96	2.88	0.43	2.91	0.45	28.50	1.39	56.7	185
157	Erguna Massif	Erguna belt	3	121.87	53.15	14ER16-1	Granodiorite		256	####	0.43	####	2.48			0.05	0.85	2.70	3.37	4.16	0.09	1.02	38	32.40	56.00	5.98	22.30	4.64	1.03	3.63	0.53	2.91	0.55	1.46	0.22	1.41	0.21	16.00	4.25	138	362
158	Erguna Massif	Erguna belt	3	121.87	53.15	14ER16-2	Granodiorite		256	####	0.40	####	2.27			0.04	0.78	2.59	3.35	4.18	0.08	1.10	38	26.70	46.40	5.03	18.80	4.10	0.97	3.39	0.49	2.81	0.48	1.34	0.20	1.30	0.18	14.80	4.15	138	355
159	Erguna Massif	Erguna belt	3	121.87	53.15	14ER16-3	Granodiorite		256	####	0.40	####	3.71			0.06	1.33	3.08	2.61	4.26	0.09	0.86	39	28.70	53.80	6.36	25.90	6.12	1.14	5.66	0.91	5.52	1.09	3.13	0.46	2.94	0.43	32.40	5.33	142	231
160	Erguna Massif	Erguna belt	3	121.87	53.15	14ER16-4	Granodiorite		256	####	0.41	####	2.40			0.04	0.81	2.36	3.29	3.97	0.09	0.98	38	19.70	36.60	4.17	16.60	3.85	0.95	3.35	0.49	2.76	0.51	1.37	0.21	1.24	0.19	14.90	3.70	127	359
161	Erguna Massif	Erguna belt	3	123.61	52.40	14ER9-1	Monzonite		246	####	0.92	####	6.10			0.09	3.62	5.76	4.00	3.10	0.30	1.42	51	42.10	80.20	9.00	35.90	6.40	1.79	4.99	0.69	3.73	0.69	1.96	0.29	1.87	0.28	19.30	3.08	110	1017
162	Erguna Massif	Erguna belt	3	123.61	52.40	14ER9-2	Monzonite		246	####	0.95	####	6.24			0.09	3.76	5.99	3.82	3.18	0.32	1.72	52	44.40	86.60	9.58	38.00	7.06	1.82	5.09	0.69	3.92	0.71	1.98	0.28	1.82	0.27	19.50	2.80	117	977
163	Erguna Massif	Erguna belt	3	123.61	52.40	14ER9-3	Monzonite		246	####	0.96	####	6.13			0.09	3.80	5.85	3.79	3.08	0.30	2.14	52	40.50	79.10	8.87	35.90	6.79	1.79	4.82	0.67	3.74	0.67	1.86	0.27	1.61	0.23	18.70	2.17	113	967
164	Erguna Massif	Erguna belt	3	120.92	51.88	15ER9-2	Monzogranite		246	####	0.70	####	3.63			0.05	1.64	4.10	4.58	2.77	0.24	1.26	45	32.20	66.20	7.83	29.90	5.52	1.40	3.94	0.50	2.50	0.45	1.11	0.16	1.02	0.14	12.40	1.86	69.2	807
165	Erguna Massif	Erguna belt	3	120.92	51.88	15ER9-3	Monzogranite		246	####	0.77	####	3.83			0.05	1.72	4.11	4.58	2.89	0.25	1.22	44	43.50	80.60	9.48	35.50	6.47	1.51	4.55	0.57	2.94	0.51	1.26	0.19	1.14	0.16	13.90	1.83	68.1	774
166	Erguna Massif	Erguna belt	3	120.92	51.88	15ER9-5	Monzogranite		246	####	0.82	####	4.09			0.06	1.81	4.24	4.48	2.83	0.27	1.26	44	40.10	80.80	9.56	36.30	6.49	1.61	4.76	0.61	3.24	0.53	1.41	0.20	1.26	0.17	15.10	2.05	73.9	784
167	Erguna Massif	Erguna belt	3	120.05	51.01	15ER2-1	Qtz monzonite		240	####	0.80	####	5.15			0.12	1.38	3.48	4.30	3.81	0.31	0.72	32	40.10	74.30	8.86	34.80	6.84	3.13	6.09	0.91	5.49	1.10	3.01	0.44	3.08	0.45	31.20	2.03	76.4	604
168	Erguna Massif	Erguna belt	3	120.05	51.01	15ER2-3	Qtz monzonite		240	####	0.83	####	4.94			0.09	1.38	3.59	4.53	3.97	0.29	0.76	33	39.50	71.30	8.46	33.00	6.62	3.01	5.81	0.87	5.24	1.02	2.96	0.45	3.04	0.46	29.90	2.23	83	572
169	Erguna Massif	Erguna belt	3			ER25-4	Granitoid		239	####	0.81	####	5.31			0.07	1.28	3.40	4.81	3.08	0.27	2.35	30	63.50	118.0	12.20	45.20	7.88	2.72	6.93	1.03	5.97	1.20	3.33	0.49	3.20	0.47	34.90	2.35	103	511
170	Erguna Massif	Erguna belt	3			ER25-5	Granitoid		239	####	0.75	####	5.22			0.10	1.23	3.21	4.51	4.21	0.28	3.18	30	64.10	107.0	12.10	43.20	6.96	2.89	6.08	0.85	4.75	0.97	2.84	0.43	2.84	0.45	29.20	2.47	112	486
171	Erguna Massif	Erguna belt	3	120.75	51.55	15ER4-1	Monzogranite□		239	####	0.04	####	1.02			0.04	0.06	0.62	3.57	4.55	0.03	0.70	9	22.70	48.00	5.70	21.50	5.17	0.47	5.24	0.97	6.01	1.22	3.66	0.56	3.77	0.54	37.90	4.90	167	40.4
172	Erguna Massif	Erguna belt	3	120.75	51.55	15ER4-3	Monzogranite□		239	####	0.05	####	0.98			0.03	0.05	0.60	3.37	4.63	0.04	0.74	8	21.20	45.40	5.42	20.50	4.93	0.49	4.95	0.88	5.44	1.09	3.16	0.51	3.34	0.47	33.90	3.97	170	40.9
173	Erguna Massif	Erguna belt	3	119.93	49.79	15ER12-1	Monzogranite□		238	####	0.35	####	2.43			0.06	0.81	1.73	3.87	3.53	0.10	0.96	37	32.10	56.80	6.40	21.90	3.87	0.77	3.36	0.49	2.99	0.60	1.78	0.27	2.00	0.32	18.80	4.40	141	219
174	Erguna Massif	Erguna belt	3	119.93	49.79	15ER12-3	Monzogranite□		238	####	0.33	####	2.20			0.06	0.80	2.02	4.04	3.46	0.10	0.80	39	29.10	54.80	5.76	19.50	3.50	0.74	2.91	0.44	2.66	0.55	1.60	0.26	1.82	0.29	16.10	3.57	131	245
175	Erguna Massif	Erguna belt	3	119.93	49.79	15ER12-6	Monzogranite□		238	####	0.35	####	2.30			0.06	0.78	1.81	3.87	3.88	0.10	0.76	38	30.90	57.00	5.87	19.70	3.36	0.69	2.90	0.42	2.65	0.53	1.67	0.26	1.96	0.31	16.00	4.45	153	223
176	Erguna Massif	Erguna belt	3			11ER5-1	Granitoid			####	0.49	####	2.69			0.38	1.09	2.60	3.92	3.33	0.16	1.34	45	33.10	57.30	7.07	26.00	4.25	1.14	3.32	0.41	2.23	0.38	0.93	0.13	0.93	0.14	10.80		98.6	547
177	Erguna Massif	Erguna belt	3			11ER5-4	Granitoid			####	0.52	####	2.76			0.04	1.21	2.87	3.82	3.56	0.16	1.25	46	40.00	69.80	8.22	29.40	4.69	1.24	3.42	0.42	2.11	0.38	1.02	0.14	0.89	0.13	11.60		100	535
178	Erguna Massif	Erguna belt	3			11ER5-5	Granitoid			####	0.43	####	2.33			0.03	1.00	2.56	4.00	3.75	0.14	1.01	46	33.20	52.70	7.22	26.30	4.33	1.21	3.23	0.42	2.13	0.38	0.99	0.14	0.85	0.12	11.40		101	554
179	Erguna Massif	Erguna belt	3			11ER5-6	Granitoid			####	0.50	####	3.24			0.06	1.05	2.64	3.85	3.60	0.15	1.37	39	40.80	67.60	8.34	31.00	5.11	1.35	3.74	0.47	2.27	0.43	1.14	0.14	0.98	0.14	13.60		98.5	534
180	Erguna Massif	Erguna belt	3			11ER15-3	Granitoid			####	0.87	####	3.88			0.05	1.81	4.12	3.48	2.93	0.19	1.52	48	33.50	79.90	10.40	38.40	7.27	1.61	5.55	0.75	3.97	0.71	2.05	0.30	1.90	0.28	22.80		110	661
181	Erguna Massif	Erguna belt	3			11ER16-1	Granitoid			####	0.74	####	4.37			0.06	2.20	4.35	3.53	3.13	0.19	0.80	50	27.60	53.50	6.25	23.70	4.77	1.11	3.76	0.52	2.74	0.47	1.33	0.22	1.21	0.17	14.80		115	539
182	Erguna Massif	Erguna belt	3			11ER16-3	Granitoid			####	0.83	####	4.17			0.05	2.07	4.13	3.63	3.27	0.19	0.77	50	31.50	65.00	7.75	28.90	5.28	1.41	4.29	0.60	3.08	0.60	1.65	0.24	1.47	0.21	19.10		166	551
183	Erguna Massif	Erguna belt	3			11ER16-4	Granitoid			####	0.77	####	4.23			0.05	2.13	4.31	3.56	2.84	0.18	0.82	50	29.80	58.30	7.15	27.60	5.37	1.24	4.36	0.61	3.07	0.59	1.55	0.20	1.26	0.19	17.40		121	566
184	Erguna Massif	Erguna belt	3			11ER16-5	Granitoid			####	0.90	####	4.82			0.06	2.55	4.85	3.57	2.97	0.20	0.64	51	33.80	73.70	9.21	35.90	6.98	1.54	5.84	0.81	4.36	0.77	2.09	0.29	1.86	0.25	23.40		204	525
185	Erguna Massif	Erguna belt	3			11ER16-9	Granitoid			####	1.46	####	8.58			0.15	4.71	6.37	3.52	2.03	0.59	1.59	52	41.40	87.80	11.30	45.30	8.60	2.11	7.03	0.96	4.87	0.86	2.28	0.32	2.07	0.28	26.80		145	712
186	Erguna Massif	Erguna belt	3			11ER28-1	Granitoid			####	0.71	####	4.31			0.07	2.33	3.76	4.15	2.61	0.22	1.82	52	28.30	51.90	5.87	22.00	3.64	1.21	2.98	0.40	2.13	0.43	1.23	0.16	1.00	0.17	12.70		72.40	673
187	Erguna Massif	Erguna belt	3			11ER28-3	Granitoid			####	1.20	####	6.58			0.10	3.64	5.62	3.32	2.39	0.28	1.57	52	34.30	69.10	8.46	32.60	6.24	1.82	5.20	0.77	4.23	0.76	2.24	0.32	1.93	0.31	24.40		81.10	622
188	Erguna Massif	Erguna belt	3			11ER28-4	Granitoid			####	0.78	####	4.39			0.07	2.36	3.89	4.25	2.66	0.22	1.44	52	36.60	70.30	8.33	30.60	5.28	1.48	4.26	0.57	3.10	0.56	1.56	0.24	1.47	0.22	16.60		71.90	673
189	Erguna Massif	Erguna belt	3			12ER10-1	Granitoid			####	0.25	####	1.61			0.02	0.24	1.12	3.48	5.00	0.08	0.98	23	35.90	61.50	6.19	20.60	3.07	1.01	2.29	0.32	1.89	0.38	1.15	0.19	1.24	0.21	12.00		77.60	207
190	Erguna Massif	Erguna belt	3			12ER10-2	Granitoid			####	0.29	####	1.58			0.02	0.25	1.13	3.48	4.88	0.09	0.82	24	54.80	94.10	9.13	28.70	4.13	1.05	2.71	0.39	2.19	0.44	1.30	0.21	1.42	0.24	14.70		80.60	211
191	Erguna Massif	Erguna belt	3			12ER10-3	Granitoid			####	0.22	####	1.58			0.02	0.19	0.97	3.28	4.91	0.07	1.00	19	35.10	59.40	5.98	19.80	3.06	0.92	2.04	0.30	1.68	0.36	1.04	0.17	1.16	0.20	11.00		80.00	194
192	Erguna Massif	Erguna belt	3			12ER10-4	Granitoid			####	0.24	####	1.57			0.02	0.20	0.97	3.28	4.86	0.07	0.96	20	24.80	42.00	4.32	14.50	2.39	0.92	1.78	0.28	1.70	0.33	0.99	0.16	1.07	0.19	10.90		77.10	201
193	Erguna Massif	Erguna belt	3			12ER11-1	Granitoid			####	0.27	####	1.74			0.04	0.73	1.46	4.08	4.36	0.07	0.78	45	26.20	48.20	5.20	18.50	2.99	0.69	2.21	0.32	1.85	0.35	1.03	0.17	1.13	0.19	12.00		170	346
194	Erguna Massif	Erguna belt	3			12ER11-2	Granitoid			####	0.27	####	1.78			0.04	0.42	1.71	4.28	3.47	0.08	0.80	32	34.10	62.00	7.17	25.80	4.69	0.84	3.51	0.54	3.06	0.58	1.71	0.28	1.78	0.25	19.50		143	319
195	Erguna Massif	Erguna belt	3			12ER11-3	Granitoid			####	0.27	####	1.77			0.04	0.37	1.54	3.95	4.73	0.07	0.66	29	36.60	64.30	7.28	25.30	4.29	0.83	3.13	0.44	2.53	0.48	1.40	0.22	1.46	0.24	16.10		171	321
196	Erguna Massif	Erguna belt	3			12ER11-4	Granitoid			####	0.30	####	1.96			0.04	0.37	1.37	3.74	4.32	0.07	0.60	27	37.60	74.00	7.67	26.70	4.31	0.78	2.94	0.44	2.35	0.47	1.36	0.20	1.42	0.22	15.40		148	294
197	Erguna Massif	Erguna belt	3			11ER1-5	Granitoid		186	####	0.06	####	0.77			0.01	trace	0.16	3.32	4.55	0.02	0.86	—	18.30	51.10	5.70	22.50	6.03	0.12	6.28	1.20	8.22	1.81	5.19	0.81	5.46	0.80	54.00	4.29	235	10.9
198	Central Mongolia	Bayanhongor-Ulaan Baatar	3			M99-04	Diorite	Daltyn-Am stock	250	####	0.88	####		8.25		0.13	5.58	7.54	3.80	1.14	0.13			26.63	54.26	6.48	25.56	5.15	1.53	3.79	0.47	2.71	0.51	1.35	0.19	1.08	0.19	13.47	3.42	32.10	854

No.	Location	Magmatic belt	Group	Longitude	Latitude	Sample	Rock type	Pluton	Age（Ma）	SiO_2	TiO_2	Al_2O_3	TFe_2O_3	Fe_2O_3	FeO	MnO	MgO	CaO	Na_2O	K_2O	P_2O_5	LOI	$Mg^{\#}$	La	Ce	Pr	Nd	Sm	Eu	Gd	Tb	Dy	Ho	Er	Tm	Yb	Lu	Y	Cs	Rb	Sr
199	Central Mongolia	Bayanhongor-Ulaan Baatar	3			M99-05	Monzogranite	Daltyn-Am stock	250	####	0.37	####		3.19		0.04	0.97	2.46	3.83	3.90	0.14			30.55	58.23	6.27	22.41	3.57	0.96	2.28	0.31	1.64	0.27	0.69	0.11	0.61	0.10	7.57	3.36	115	479
200	Central Mongolia	Bayanhongor-Ulaan Baatar	3			M99-06	Granite	Hangay batholith	250	####	0.25	####		1.65			0.43	1.95	3.96	4.59	0.07			32.64	66.25	6.43	21.17	3.60	0.65	2.00	0.28	1.31	0.17	0.43	0.05	0.35	0.06	4.94	11.60	203	215.4
201	Central Mongolia	Bayanhongor-Ulaan Baatar	3			M99-07	Granite	Hangay batholith	250	####	0.60	####		3.95		0.05	1.36	3.02	4.02	3.78	0.20			35.94	68.93	7.44	27.02	4.87	1.15	3.48	0.45	2.17	0.32	0.95	0.13	0.75	0.13	10.50	15.10	159	421.9
202	Central Mongolia	Bayanhongor-Ulaan Baatar	3			M99-08	Granite	Hangay batholith	250	####	0.26	####		1.73			0.43	1.34	3.90	4.70	0.11			31.09	61.62	6.07	19.62	3.17	0.60	1.92	0.27	1.02	0.15	0.37	0.05	0.33	0.05	4.24	15.80	171	202.5
203	Central Mongolia	Bayanhongor-Ulaan Baatar	3			M99-09	Granite	Hangay batholith	250	####	0.54	####		3.57		0.04	1.07	2.98	3.69	3.97	0.19			30.48	57.01	6.06	22.64	3.95	1.30	3.02	0.36	1.82	0.27	0.71	0.10	0.54	0.07	8.20	13.80	124	470.5
204	Central Mongolia	Bayanhongor-Ulaan Baatar	3			M99-10	Granitic dike	Hangay batholith	250	####		####		0.88		0.03		0.96	3.84	4.87				7.54	15.59	1.70	6.87	1.74	0.29	1.83	0.26	1.55	0.29	0.90	0.14	0.90	0.13	10.27	9.70	251	72.60
205	Central Mongolia	Bayanhongor-Ulaan Baatar	3			M99-13	Microgranite	Hangay batholith	250	####	0.42	####		3.00		0.06	0.69	1.21	4.51	3.02	0.12			33.96	73.30	8.30	32.89	6.10	1.07	5.75	0.90	5.38	1.13	3.36	0.56	4.01	0.62	32.61	2.89	82.30	201.4
206	Central Mongolia	Bayanhongor-Ulaan Baatar	3			M99-15	Granite	Nariy Teel pluton	230	####	0.10	####		0.99			0.20	1.19	3.33	5.36	0.05			41.42	81.92	8.49	28.43	4.12	0.50	1.85	0.23	0.94	0.14	0.46	0.05	0.41	0.05	3.79	2.04	217	206.1
207	Central Mongolia	Bayanhongor-Ulaan Baatar	3			M99-16	Granite	Nariy Teel pluton	230	####	0.45	####		2.44		0.03	0.60	1.10	3.76	5.04	0.16			55.75	133.27	13.73	49.83	8.99	1.00	6.25	0.84	4.69	0.77	2.14	0.30	1.88	0.28	23.69	2.99	236	297
208	Central Mongolia	Bayanhongor-Ulaan Baatar	3			M99-17	Granodiorite	Unname pluton	230	####	0.63	####		4.12		0.04	1.43	3.81	3.90	3.22	0.19			31.36	60.16	6.54	24.90	4.34	1.07	3.07	0.37	1.76	0.26	0.69	0.09	0.67	0.10	7.64	2.34	98.80	508.4
209	North Mongolia	Kharkhorin	3			KhAR4486	Diorite	Kharkhorin massif	210	####	0.64	####		2.67	3.24	0.08	5.20	4.23	4.06	2.95	0.22			28.00	50.00		24.00	5.50	1.20	4.50	0.70					1.50	0.20	22.00	7.00	65	433
210	North Mongolia	Khentei-Dauria	3			DKh1734	Granodiorite	Khentei-Dauria batholith	210	####	0.50	####		2.20	0.72	0.07	0.72	1.82	4.16	4.22	0.20			20.00	40.00	5.90	23.00	5.10	0.70	4.20	0.60	3.30	0.60	1.60	0.20	1.50	0.20	15.00	9.00	117	109
211	North Mongolia	Khentei-Dauria	3			BKh392	Granodiorite	Khentei-Dauria batholith	210	####	0.60	####		0.40	1.69	0.10	1.34	2.45	4.42	3.93	0.14			36.00	79.00	8.50	30.00	5.20	0.80	3.80	0.60	3.20	0.60	1.60	0.20	0.70	0.10	16.00	2.00	120	361
212	North Mongolia	Khentei-Dauria	3			Kh4426	Granite	Khentei-Dauria batholith	210	####	0.29	####		1.05	1.67	0.05	0.19	0.81	3.92	4.30	0.05			106.0	173.0		76.00	16.20	0.50	10.00	1.70					4.60	0.60	67.00	5.00	164	73.00
213	North Mongolia	Khentei-Dauria	3			IL2071	Granite	Khentei-Dauria batholith	210	####	0.06	####		0.49	1.79	0.06	0.16	0.58	4.02	4.32				33.00	58.00	8.40	31.00	6.40	0.40	5.10	0.90	5.00	1.00	2.70	0.40	2.90	0.40	26.00	8.00	134	31.00
214	North Mongolia	Khentei-Dauria	3			KRN001	Granite	Khentei-Dauria batholith	210	####	0.18	####		0.50	1.80	0.05	0.17	0.83	3.83	4.49	0.07			55.00	112.0	11.20	39.00	7.40	0.40	6.60	1.10	5.80	1.20	3.30	0.40	3.10	0.40	33.00	7.00	299	68.00
215	North Mongolia	Khentei-Dauria	3			KRN002	Granodiorite	Khentei-Dauria batholith	210	####	0.57	####		0.57	3.50	0.08	2.16	3.12	4.13	3.42	0.13			44.00	84.00	8.20	28.00	4.20	1.10	3.80	0.60	3.20	0.60	1.70	0.30	1.60	0.30	17.00	7.00	110	309
216	North Mongolia	Khentei-Dauria	3			KRN003	Granodiorite	Khentei-Dauria batholith	210	####	0.46	####		0.38	3.23	0.06	1.02	2.73	3.87	3.66	0.11			29.00	55.00	5.60	20.00	3.70	0.90	3.30	0.50	2.80	0.50	1.50	0.20	1.50	0.20	15.00	13.00	127	242
217	North Mongolia	Khentei-Dauria	3			KRN004	Granodiorite	Khentei-Dauria batholith	210	####	0.50	####		0.39	3.59	0.08	1.42	2.91	3.51	3.81	0.11			50.00	90.00	9.20	30.00	5.20	0.70	4.30	0.70	3.70	0.70	2.00	0.30	2.00	0.30	21.00	13.00	150	244
218	North Mongolia	Khentei-Dauria	3			KRN005	Granodiorite	Khentei-Dauria batholith	210	####	0.52	####		0.10	2.51	0.05	0.43	1.91	4.62	3.97	0.14			52.00	98.00	9.60	33.00	4.80	1.00	3.30	0.40	1.50	0.20	0.60	0.10	0.40	0.00	6.00	7.00	128	377
219	North Mongolia	Khentei-Dauria	3			KRN006	Granodiorite	Khentei-Dauria batholith	210	####	0.45	####		0.28	3.41	0.09	1.17	2.71	3.30	4.06	0.10			51.00	95.00	8.90	26.00	4.10	0.80	3.80	0.60	3.30	0.70	2.10	0.30	2.00	0.20	19.00	11.00	139	239
220	North Mongolia	Khentei-Dauria	3			KRN007	Granodiorite	Khentei-Dauria batholith	210	####	0.49	####		0.55	3.30	0.09	1.33	2.92	3.79	3.80	0.11			52.00	100.0	9.30	32.00	5.70	0.90	4.60	0.80	4.30	0.80	2.50	0.40	2.50	0.40	23.00	11.00	141	235
221	North Mongolia	Khentei-Dauria	3			KRN008	Granodiorite	Khentei-Dauria batholith	210	####	0.60	####		0.40	3.92	0.11	2.06	3.08	3.79	3.57	0.14			41.00	84.00	8.10	29.00	4.70	0.90	3.90	0.60	3.40	0.70	1.80	0.30	1.60	0.30	17.00	8.00	129	263
222	North Mongolia	Zhanchivlan	3			ZhZh4436	Plagiogranite	Zhanchivlan massif	210	####	0.57	####		0.17	1.44	0.02	0.55	1.65	5.70	1.30	0.05			12.00	24.00		14.00	3.60	0.60	1.60	0.20					0.50	0.10	15.00	2.00	25.00	321
223	North Mongolia	Zhanchivlan	3			ZhZh4427	Granite	Zhanchivlan massif	210	####	0.41	####		0.83	1.90	0.05	0.44	1.51	3.69	4.62	0.16			37.00	67.00		34.00	7.90	0.80	4.70	0.70					1.80	0.20	36.00	15.00	184	144
224	North Mongolia	Zhanchivlan	3			ZhZh4428	Granite	Zhanchivlan massif	210	####	0.30	####		0.56	0.13	0.06	0.20	0.77	3.55	4.52	0.04			22.00	44.00		25.00	6.60	0.30	4.80	0.90					3.70	0.60	55.00	23.00	317	66.00
225	North Mongolia	Zhanchivlan	3			ZhZh4430	Li-F granite	Zhanchivlan massif	210	####	0.22	####		0.43	2.07	0.04	0.06	0.47	3.52	4.57	0.02			46.00	87.00		46.00	11.20	0.20	10.00	2.00					11.20	1.80	214.0	37.00	598	41.00
226	North Mongolia	Zhanchivlan	3			ZhZh4433	Li-F granite	Zhanchivlan massif	210	####	0.32	####		0.18	0.63	0.03	0.01	0.20	4.84	4.11	0.01			14.00	38.00		30.00	10.40	0.02	10.40	2.20					17.80	3.00	214.0	30.00	597	41.00
227	North Mongolia	Zhanchivlan	3			ZhZh4434	Li-F granite	Zhanchivlan massif	210	####	0.01	####		0.13	0.62	0.07	0.02	0.22	4.04	5.34	0.01			3.00	7.00		5.00	1.40	0.03	1.10	0.30					2.20	0.40	654.0	25.00	2427	36.00
228	Jiamusi Block	Southeastern MOS	3	132.07	46.14	HLJ-(0)4	Andesite	Baoqing area	280	####	0.83	####	6.52			0.12	2.62	5.63	3.52	1.95	0.25	3.27	45	26.60	51.70	6.87	28.40	5.52	1.74	5.30	0.78	4.48	0.93	2.63	0.41	2.54	0.42	20.40		40.00	775
229	Jiamusi Block	Southeastern MOS	3	132.05	46.20	HLJ-(0)5	Andesite	Baoqing area	282	####	0.83	####	6.77			0.11	2.76	6.11	3.74	1.49	0.25	2.12	45	25.20	49.80	6.73	28.20	5.56	1.67	5.35	0.80	4.55	0.93	2.59	0.41	2.53	0.41	20.50		70.00	416
230	Jiamusi Block	Southeastern MOS	3	132.07	46.14	HLJ-(0)6	Andesite	Baoqing area	280	####	0.90	####	8.13			0.14	3.16	5.64	4.10	1.16	0.25	3.57	44	23.30	44.50	6.19	26.60	5.26	1.55	4.70	0.72	4.14	0.86	2.48	0.42	2.88	0.38	22.50		26.00	780
231	Jiamusi Block	Southeastern MOS	3	132.07	46.14	HLJ-(0)7	Andesite	Baoqing area	282	####	1.00	####	7.91			0.12	3.09	5.58	3.28	1.78	0.30	4.03	44	24.40	48.50	6.55	28.10	5.50	1.50	4.80	0.72	4.16	0.87	2.45	0.43	2.82	0.39	22.10		28.00	817
232	Jiamusi Block	Southeastern MOS	3	132.05	46.20	HLJ-(0)8	Andesite	Baoqing area	280	####	0.90	####	7.97			0.11	3.33	5.31	3.86	1.72	0.25	4.10	46	23.70	51.20	6.36	27.10	5.25	1.54	4.54	0.70	4.08	0.83	2.37	0.40	2.79	0.38	20.70		29.00	839
233	Jiamusi Block	Southeastern MOS	3	132.05	46.20	HLJ-(0)9	Andesite	Baoqing area	282	####	0.80	####	7.56			0.10	3.10	6.80	3.94	1.72	0.25	4.19	45	20.90	48.80	5.78	25.60	5.15	1.62	4.64	0.71	4.09	0.84	2.38	0.40	2.83	0.37	22.60		23.00	579
234	Jiamusi Block	Southeastern MOS	3	132.05	46.20	HLJ-(0)10	Andesite	Baoqing area	280	####	1.01	####	7.06			0.14	3.32	6.06	3.40	1.44	0.33	4.53	48	23.90	51.80	6.21	25.70	5.09	1.67	4.72	0.70	3.95	0.82	2.25	0.36	2.53	0.37	22.70		68.00	725
235	Eastern CAOB	Southeastern MOS	3			15D3WP-11	Appinite	Duobaoshan suite	224	####	0.81	####		5.49		0.09	3.23	7.15	3.68	2.34	0.16	7.58	53.8	23.61	43.84	5.80	21.67	3.43	1.03	2.63	0.37	2.16	0.43	1.24	0.19	1.30	0.22	13.22	0.67	24.81	977.27
236	Eastern CAOB	Southeastern MOS	3			15D3WP-12	Appinite	Duobaoshan suite	224	####	0.74	####		5.06		0.07	3.28	8.92	3.99	1.93	0.19	4.00	56.2	14.72	35.24	5.28	22.31	4.71	1.17	3.37	0.44	2.17	0.41	1.03	0.14	0.90	0.13	11.16	1.08	15.67	935.56
237	Eastern CAOB	Southeastern MOS	3			15D3WP-13	Appinite	Duobaoshan suite	224	####	0.85	####		5.58		0.07	3.78	8.56	3.98	1.75	0.16	3.98	57.3	14.80	35.49	5.13	21.47	4.70	1.19	3.33	0.44	2.14	0.40	1.02	0.14	0.85	0.13	13.63	1.08	25.00	1357
238	Eastern CAOB	Southeastern MOS	3			15D3WP-14	Appinite	Duobaoshan suite	224	####	1.08	####		6.68		0.09	5.04	8.85	3.24	1.56	0.25	4.30	59.9	18.48	42.75	5.97	24.29	5.03	1.23	4.16	0.56	2.89	0.57	1.57	0.23	1.52	0.23	15.99	0.59	25.15	1279.5
239	Eastern CAOB	Southeastern MOS	3			15D3WP-15	Appinite	Duobaoshan suite	224	####	1.04	####		6.77		0.09	4.67	9.68	3.41	1.31	0.24	4.08	57.7	34.86	74.27	9.80	37.93	6.88	1.96	5.26	0.64	2.99	0.51	1.42	0.20	1.31	0.20	16.41	0.66	18.24	1364.7
240	Eastern CAOB	Southeastern MOS	3			15D3WP-16	Appinite	Duobaoshan suite	224	####	0.97	####		6.13		0.08	4.47	9.35	3.17	2.18	0.15	3.98	59.1	32.63	69.65	9.26	35.29	6.60	1.85	4.94	0.62	2.87	0.53	1.36	0.20	1.26	0.19	15.06	1.36	29.07	1318.6
241	Eastern CAOB	Southeastern MOS	3			15D3WP-18	Qtz monzonite	Duobaoshan suite	223	####	0.31	####		2.24		0.09	0.73	2.42	5.35	2.91	0.16	3.74	39.2	58.52	119.27	14.45	50.91	9.25	2.38	6.92	0.88	4.15	0.78	1.98	0.27	1.69	0.25	10.64	1.06	28.74	262.75
242	Eastern CAOB	Southeastern MOS	3			15D3WP-19	Qtz monzonite	Duobaoshan suite	223	####	0.31	####		2.22		0.09	0.74	2.57	5.26	2.89	0.15	3.84	39.8	36.33	80.01	11.12	46.21	10.10	3.00	7.42	0.90	4.06	0.72	1.76	0.24	1.44	0.21	11.55	0.36	24.22	246.08
243	Eastern CAOB	Southeastern MOS	3			15D3WP-20	Qtz monzonite	Duobaoshan suite	223	####	0.32	####		2.07		0.09	0.72	2.42	5.56	2.76	0.16	3.74	40.8	51.76	111.83	14.86	58.73	11.41	2.85	7.49	0.89	3.95	0.70	1.72	0.24	1.48	0.22	12.38	0.24	27.29	261.08
244	Eastern CAOB	Southeastern MOS	3			KCA1-189	Granite porphyry	Duobaoshan suite	223	####	0.10	####		1.08		0.02	0.38	0.68	5.47	2.07	0.05	1.40	41.1	25.40	50.29	6.42	21.98	3.89	0.98	2.66	0.37	2.15	0.42	1.17	0.18	1.18	0.20	11.49	0.73	31.91	225.27
245	Eastern CAOB	Southeastern MOS	3			KCA1-190	Granite porphyry	Duobaoshan suite	223	####	0.11	####		0.88		0.04	0.24	0.89	4.95	3.57	0.06	1.16	35.1	18.17	41.93	6.26	26.10	5.22	0.96	3.65	0.45	2.16	0.41	1.02	0.14	0.86	0.13	10.25	0.61	39.15	252.33
246	Southeast of the MOS	Southern MOS	3	111.70	48.31	TCS 7.1	Low-Si adakite		138	####	1.56	####	6.86			0.10	2.15	4.67	4.44	3.42	0.81	1.17		76.32	148.6	17.15	62.37	10.15	2.88	7.89	0.77	3.95	0.67	1.44	0.18	1.12	0.15	15.91		78.00	1136
247	Southeast of the MOS	Southern MOS	3	111.70	48.31	TCS 7.2	Low-Si adakite			####	1.55	####	7.08			0.09	1.75	4.55	4.51	3.60	0.80	1.13		80.02	156.4	18.96	67.60	11.42	3.02	8.70	0.92	4.65	0.78	1.89	0.23	1.42	0.20	19.43		80.80	1153
248	Southeast of the MOS	Southern MOS	3	111.70	48.31	TCS 7.3	Low-Si adakite			####	1.51	####	6.89			0.07	2.26	5.99	4.39	3.44	0.79	1.84		83.21	163.0	18.74	69.43	11.63	3.02	8.83	0.95	4.87	0.81	2.00	0.25	1.48	0.22	22.01		76.40	1135
249	Southeast of the MOS	Southern MOS	3	111.70	48.31	TCS 7.4	Low-Si adakite			####	1.53	####	6.84			0.09	2.25	4.67	4.47	3.56	0.80	1.17		84.94	164.5	19.38	70.14	12.01	3.12	8.34	0.95	4.83	0.83	1.95	0.25	1.50	0.21	22.00		77.60	1135
250	Southeast of the MOS	Southern MOS	3	111.70	48.31	TCS 7.5	Low-Si adakite			####	1.72	####	7.88			0.07	2.85	5.94	2.51	3.95	0.93	5.03		91.42	180.1	21.00	77.27	12.85	3.33	9.06	1.00	5.01	0.88	1.97	0.24	1.36	0.18	20.99		81.90	1932
251	Southeast of the MOS	Southern MOS	3	111.70	48.31	TCS 7.6	Low-Si adakite			####	1.70	####	8.07			0.07	2.12	5.35	4.74	3.30	0.96	0.72		89.13	183.6	20.88	76.42	13.04	3.29	9.30	1.03	5.26	0.84	1.89	0.26	1.53	0.22	21.85		82.40	1261
252	Southeast of the MOS	Southern MOS	3	111.70	48.31	TCS 7.7	Low-Si adakite		139	####	1.70	####	7.81			0.10	2.28	5.09	4.73	3.29	0.92	1.23		93.56	182.7	21.53	79.04	12.84	3.33	9.54	1.01	5.27	0.81	1.95	0.26	1.46	0.22	21.74		66.70	1273
253	Southeast of the MOS	Southern MOS	3	107.76	46.28	TCS 59.1	High-Si adakites			####	0.71	####	4.52			0.08	2.05	4.01	4.39	2.99	0.24	1.92		45.67	87.43	9.90	33.94	6.21	1.52	4.49	0.57	3.14	0.58	1.51	0.22	1.39	0.20	14.97		163.4	805.84
254	Southeast of the MOS	Southern MOS	3	107.76	46.28	TCS 59.2	High-Si adakites		230	####	0.73	####	4.27			0.06	0.96	3.71	4.39	3.84	0.26	0.77		46.43	88.09	9.67	34.37	5.75	1.51	4.73	0.53	2.91	0.53	1.33	0.21	1.22	0.17	13.59		139.6	809.41
255	Southeast of the MOS	Southern MOS	3	107.76	46.28	TCS 59.3	High-Si adakites			####	0.71	####	4.26			0.08	2.08	4.08	4.63	3.10	0.25	1.84		47.86	92.15	9.60	35.62	5.80	1.53	5.05	0.53	3.09	0.57	1.60	0.21	1.33	0.21	16.37		144.5	775.78
256	Southeast of the MOS	Southern MOS	3	107.76	46.28	TCS 59.4	High-Si adakites			####	0.72	####	4.24			0.08	2.01	4.01	4.15	3.04	0.25	1.85		46.45	89.45	9.92	34.80	5.83	1.56	4.36	0.55	3.11	0.56	1.46	0.22	1.28	0.21	15.36		148.9	799.62
257	Southeast of the MOS	Southern MOS	3	111.04	47.67	TCS 2.1	Trachyandesite			####	2.42	####	####			0.13	2.43	6.41	3.60	2.96	1.40	1.43		103.82	222.7	26.22	105.3	18.09	4.06	13.38	1.63	7.77	1.37	3.44	0.46	2.67	0.45	39.83		83.10	802.7
258	Southeast of the MOS	Southern MOS	3	111.38	47.85	TCS 3.1	Trachyandesite			####	2.14	####	8.74			0.19	2.02	6.57	3.99	2.84	1.19	1.47		86.92	175.1	20.49	83.34	15.24	3.51	10.91	1.31	6.17	1.13	2.69	0.35	2.13	0.34	31.14		70.60	879.6
259	Southeast of the MOS	Southern MOS	3	111.49	47.89	TCS 4.1	Trachyandesite		121	####	3.27	####	####			0.18	3.47	7.47	3.38	2.02	1.95	1.16		98.25	218.1	27.20	121.2	21.10	5.07	15.07	1.89	8.60	1.57	3.65	0.47	2.77	0.45	43.21		46.20	1182.8
260	Southeast of the MOS	Southern MOS	3	111.51	48.02	TCS 5.1	Trachyandesite			####	0.87	####	4.79			0.15	0.61	2.98	3.94	4.52	0.41	0.73		96.31	196.3	24.03	96.05	17.84	4.35	12.72	1.67	8.40	1.61	4.12	0.57	3.54	0.60	43.41		152.2	627.6
261	Southeast of the MOS	Southern MOS	3	111.67	48.19	TCS 6.1	Trachyandesite			####	2.34	####	9.87			0.12	2.53	6.88	3.95	2.07	1.31	1.62		91.07	195.1	23.12	96.21	16.19	3.72	11.88	1.46	7.15	1.29	3.25	0.41	2.55	0.40	36.40		62.00	887
262	Southeast of the MOS	Southern MOS	3	112.81	49.29	TCS 8.1	Trachyandesite			####	3.28	####	####			0.11	2.18	6.69	3.67	2.94	1.87	1.29		121.57	218.9	30.20	133.8	22.44	5.20	16.28	2.00	8.97	1.57	3.90	0.47	2.66	0.42	45.55		58.00	1077.7
263	Southeast of the MOS	Southern MOS	3	112.85	49.31	TCS 9.1	Trachyandesite			####	3.01	####	####			0.21	3.56	6.96	3.65	2.02	1.84	1.48		113.1	247.3	29.66	120.14	21.49	4.74	15.23	1.76	8.53	1.47	3.77	0.47	2.81	0.48	43.88		49.90	1081.3
264	Southeast of the MOS	Southern MOS	3	112.86	49.31	TCS 10.1	Trachyandesite			####	3.03	####	####			0.18	3.60	7.08	3.51	2.03	1.86	1.73		110.7	239.6	29.36	129.57	22.03	5.16	15.84	1.94	9.27	1.59	3.96	0.50	3.03	0.45	45.32		51.20	1041.8
265	Southeast of the MOS	Southern MOS	3	112.91	49.34	TCS 11.1	Trachyandesite			####	2.49	####	####			0.15	1.35	5.35	3.84	3.09	1.44	1.56		113.45	239.1	28.4	119.84	19.21	4.40	13.75	1.69	7.91	1.48	3.57	0.46	2.83	0.45	41.22		51.90	898.1
266	Southeast of the MOS	Southern MOS	3	113.01	49.38	TCS 13.1	Trachyandesite			####	1.84	####	7.12			0.06	2.14	5.80	3.79	3.11	1.10	1.96		93.90	208.1	23.03	95.24	16.50	3.38	11.49	1.41	6.81	1.26	3.22	0.42	2.59	0.41	35.32		71.80	717.2
267	Central MOS	Central MOS	3	106.98	47.86	38	I phase granite	Bogd Uul	211	75.8	0.15	12.7		1.64		0.03	0.08	0.68	3.79	4.34	0.03			39	101	50	55	50										63	11	294	53
268	Central MOS	Central MOS	3	107.06	47.74	56	I phase granite	Bogd Uul	211	75.1	0.19	13		1.77		0.03	0.1	0.47	3.75	4.47	0.03			78	88	50	60	50										31	6	249	44
269	Central MOS	Central MOS	3	107.02	47.85	64	I phase granite	Bogd Uul	211	73.2	0.14	14		1.65		0.03	0.07	0.55	3.94	5.52	0.03			70	147	50	43	14										77	21	546	37
270	Central MOS	Central MOS	3	106.94	47.86	2003	I phase granite	Bogd Uul	211	74.3	0.29	12.8		2.22		0.04	0.22	0.92	3.53	4.64	0.07			104	173	50	87	50										78	8	233	110
271	Central MOS	Central MOS	3	107.01	47.83	2005	I phase granite	Bogd Uul	211	74.5	0.2	12.9		1.93		0.04	0.13	0.65	3.9	4.53	0.04			83	125	50	57	50										54	9	326	42
272	Central MOS	Central MOS	3	107.05	47.86	2028	I phase granite	Bogd Uul	211	74	0.16	13.2		1.89		0.04	0.07	0.8	3.96	4.69	0.03			58	151	11.17	50	50										88	19	440	51
273	Central MOS	Central MOS	3	107.08	47.86	2030	I phase granite	Bogd Uul	211	71.6	0.13	14.6		1.38		0.03	0.08	0.82	4.15	6.11	0.01			44.61	99.35	11.17	39.17	8.48	0.16	9.17	1.82	12.13	2.67	8.86	1.57	11.09	1.65	78	12	499	16
274	Central MOS	Central MOS	3	106.99	47.78	2051	I phase granite	Bogd Uul	211	75.5	0.21	12.4		1.9		0.04	0.18	0.76	3.82	4.28	0.05			71	102	50	55	50										32	10	288	59
275	Central MOS	Central MOS	3	107.00	47.81	2052	I phase granite	Bogd Uul	211	76.5	0.16	12.1		1.74		0.04	0.11	0.47	3.57	4.4	0.03			47	90	50	50	50										14	12	270	37
276	Central MOS	Central MOS	3	107.00	47.79	2053	I phase granite	Bogd Uul	211	74	0.22	13.2		1.99		0.04	0.17	0.88	4.06	4.35	0.05			38.8	81.41	8.83	31.22	5.24	0.7	4.99	0.86	5.82	1.18	3.75	0.74	4.47	0.78	32	10	299	72
277	Central MOS	Central MOS	3	106.59	47.52	2057	I phase granite	Bogd Uul	211	68	0.28	16.3		2.27		0.04	0.3	1.05	4.4	6.83	0.13			46.92	104.95	12.02	45.31	8.97	1	8.87	1.47	8.7	1.64	5.72	0.88	5.45	0	35	11	274	120
278	Central MOS	Central MOS	3	106.98	47.88	2064	II phase granite	Bogd Uul	211	68.4	0.33	14.7		3.03		0.06	0.29	1.63	4.19	6.27	0.15			32.85	90.89	11.67	44.66	10.36	0.86	9.61	1.81	12.18	2.28	7.64	1.13	7.63	1.2	60	17	284	112
279	Central MOS	Central MOS	3	107.09	47.83	29	II phase granite	Bogd Uul	211	76.7	0.08	12.3		1.23		0.03	0.01	0.5	3.51	4.46	0.01			104			64	50										113	15	522	12
280	Central MOS	Central MOS	3	107.05	47.87	33	II phase granite	Bogd Uul	211	76	0.1	12.6		1.37		0.02	0.04	0.47	3.71	4.87	0.01			43.61	96.99	10.96	35.08	8.5	0	8.44	1.82	13.03	2.88	10.03	1.7	12.36	1.69	99	17	517	17
281	Central MOS	Central MOS	3	107.12	47.79	34	II phase granite	Bogd Uul	211	76.6	0.09	12.2		1.41		0.03	0.04	0.52	3.42	4.77	0.01			62	102	50	39	14										83	16	509	24
282	Central MOS	Central MOS	3	106.94	47.83	43	II phase granite	Bogd Uul	211	75.6	0.21	12.4		2.06		0.04	0.15	0.82	3.75	4.04	0.05			90	120	50	66	50										44	11	279	64
283	Central MOS	Central MOS	3	106.97	47.80	47	II phase granite	Bogd Uul	211	75	0.21	12.8		1.82		0.05	0.21	0.79	3.53	4.73	0.05			56	112	50	32	14										39	18	336	73
284	Central MOS	Central MOS	3	106.98	47.79	48	II phase granite	Bogd Uul	211	76.7	0.15	12.2		1.47		0.03	0.08	0.53	3.66	4.35	0.02			87	95	50	80	50										10	10	271	31
285	Central MOS	Central MOS	3	107.05	47.85	65	II phase granite	Bogd Uul	211	76.9	0.09	12.5		0.6		0.01	0.01	0.44	3.56	4.61	0.02			61	91	50	56	50										79	8	419	39
286	Central MOS	Central MOS	3	107.03	47.87	2004	II phase granite	Bogd Uul	211	76.2	0.1	12.3		1.19		0.02	0.02	0.56	3.26	5.19	0.01			54	111	50	51	50										99	15	469	25
287	Central MOS	Central MOS	3	107.10	47.50	2018	II phase granite	Bogd Uul	211	77	0.1	12		1.28		0.02	0.01	0.5	3.62	4.33	0.01			63	128	50	83	50										109	14	444	6
288	Central MOS	Central MOS	3	107.03	47.80	2034	II phase granite	Bogd Uul	211	77.2	0.06	12.1		1.18		0.01	0.01	0.42	3.69	4.26	0			30.64	64.36	7.04	22.55	4.13	0.24	4.23	0.72	5.09	1.17	3.35	0.64	4.38	0.67	109	10	516	4
289	Central MOS	Central MOS	3	107.08	47.80	2038	II phase granite	Bogd Uul	211	77.1	0.08	12.3		1.01		0.03	0.02	0.5	4.02	4.07	0.01			29	66	50	50	50										57	16	380	14
290	Central MOS	Central MOS	3	106.96	47.79	2047	II phase granite	Bogd Uul	211	75.3	0.22	12.9		1.69		0.03	0.2	0.74	3.25	4.92	0.04			71	108		57	50										53	10	179	112
291	Central MOS	Central MOS	3	106.98	47.86	38/1	Microgranite dyke	Bogd Uul	211	76.6	0.09	12.5		0.9		0.02	0.01	0.6	3.77	4.87	0.01			20	35		50	50										69	7	317	21
292	Central MOS	Central MOS	3	107.04	47.75	59/1	Microgranite dyke	Bogd Uul	211	77.6	0.08	12.3		0.84		0.02	0.01	0.44	3.77	4.18	0.01			40	29		50	50										3	5	261	10
293	Central Mongolia	Southern MOS	3	106.28	44.97	MO-144	Miaroliticgranite	Olzit	209	69.6	0.35	14.3	3.35			0.06	0.06	0.59	4.16	5.54	0.02	1.8	0.03	124.8	273.3	29.8	104.7	19.38	0.84	18.38	2.86	18.69	3.63	11.5	1.77	11.43	1.72	106.5		205.7	28.5
294	Central Mongolia	Southern MOS	3	106.28	44.97	MO-145	Miaroliticgranite	Olzit	209	71.6	0.29	14.5	1			0.01	0.15	0.56	4.68	5.7	0.02	1.3	0.21	102.6	173.9	24.58	78.5	16.8	0.7	16.84	2.63	16.86	3.6	10.93	1.68	10.34	1.66	103.2		227.5	28.2
295	Central Mongolia	Southern MOS	3	106.28	44.97	MO-146	Miaroliticgranite	Olzit	209	69.4	0.35	14.6	3.1			0.05	0.09	0.56	4.74	5.71	0.03	1.1	0.05	119.9	249.7	28.72	102.6	18.23	0.9	16.46	2.52	17.08	3.22	10.01	1.64	10.77	1.6	93.2		210.1	33.3
296	Central Mongolia	Southern MOS	3	106.28	44.97	MO-147	Miaroliticgranite	Olzit	209	70.3	0.29	14.3	3.18			0.11	0.04	0.46	4.21	5.39	0.02	1.4	0.02	91.2	253.7	22.3	75.6	15.12	0.62	14.79	2.48	18.06	3.68	11.6	1.92	12.65	1.81	110.5		218.5	25
297	Central Mongolia	Southern MOS	3	106.28	44.97	MO-148	Miaroliticgranite	Olzit	209	71.5	0.26	13.9	1.97			0.01	0.13	0.48	4.65	5.28	<0.01	1.6	0.11	134.3	285.8	32.57	120.2	22.81	0.75	23.54	4.05	26.61	5.53	16.19	2.35	16.72	2.35	144.9		282.3	20.6
298	Central Mongolia	Southern MOS	3	106.29	44.97	MO-150	Biotite-bearinggranite	Olzit	213	62.4	0.58	15.6	5.76			0.14	0.13	1.89	4.29	5.58	0.08	3.4	0.04	73.7	156.8	18.23	67.8	12.52	2.78	11.56	1.65	9.66	1.97	5.57	0.88	6.12	0.97	50		97.6	67.8

No.	Location	Magmatic belt	Group	Longitude	Latitude	Sample	Rock type	Pluton	Age（Ma）	SiO_2	TiO_2	Al_2O_3	TFe_2O_3	Fe_2O_3	FeO	MnO	MgO	CaO	Na_2O	K_2O	P_2O_5	LOI	Mg#	La	Ce	Pr	Nd	Sm	Eu	Gd	Tb	Dy	Ho	Er	Tm	Yb	Lu	Y	Cs	Rb	Sr
299	Central Mongolia	Southern MOS	3	106.29	44.97	MO-152	Biotite-bearinggranite	Olzit	213	65.4	0.44	14.4	6.63			0.2	0.07	0.53	5.43	4.77	0.05	1.8	0.02	90.9	216	24.76	92.3	18.85	2.03	19.57	2.98	19.62	3.77	11.97	1.75	11.79	1.69	115.4		103.3	23.8
300	Central Mongolia	Southern MOS	3	106.29	44.97	MO-153	Biotite-bearinggranite	Olzit	213	63.9	0.6	15.2	6.88			0.17	0.09	0.62	5.5	5.65	0.08	1.2	0.02	53.8	143.9	16.24	68.5	12.21	1.81	12.76	1.85	11.55	2.37	6.85	1.11	7.62	1.2	63.7		109.7	29.2
301	Northern Mongolia	Nrothern MOS	3			1046	Granite	Kataev volcanoplutonic complex	Late Triassic	66.8	0.44	16.1	3.65			0.03	0.52	1.74	5.83	3.93	0.1	1.02		31.34	52.59	6.31	20.83	3.69	0.80	2.52	0.32	2.06	0.39	1.19	0.18	1.51	0.25	12.84		53.55	588.00
302	Northern Mongolia	Nrothern MOS	3			1072	Granite	Kataev volcanoplutonic complex	Late Triassic	71.1	0.28	15.4	2.08			0.05	0.1	0.91	4.05	5.09	0.07	0.9		46.20	87.64	10.47	34.97	6.27	1.54	4.00	0.51	2.99	0.56	1.58	0.24	1.60	0.28	16.54		96.06	490.51
303	Northern Mongolia	Nrothern MOS	3			06525	Granite	Kataev volcanoplutonic complex	Late Triassic	71.7	0.27	15		1.03	0.42	0.01	0.13	0.26	4.89	5.38	0.05	0.48		34.04	80.52	7.27	23.51	5.31	0.66	2.72	0.37	2.28	0.46	1.25	0.22	1.47	0.24	16.38		99.40	169.31
304	Northern Mongolia	Nrothern MOS	3			06527	Granite	Kataev volcanoplutonic complex	Late Triassic	69.9	0.29	15.8		1.24	0.54	0.03	0.32	0.64	4.89	5.25	0.07	0.64		53.73	105.16	10.01	28.65	3.89	0.70	1.60	0.24	1.31	0.27	0.74	0.13	0.86	0.14	9.02		86.47	300.69
305	Northern Mongolia	Nrothern MOS	3			06529	Granite	Kataev volcanoplutonic complex	Late Triassic	70.2	0.28	15.4		1.18	0.91	0.02	0.58	0.9	5.76	4.07	0.12	0.57		57.75	95.76	10.69	36.51	5.16	1.12	2.89	0.38	2.39	0.46	1.28	0.20	1.34	0.25	18.81		64.74	507.03
306	Northern Mongolia	Nrothern MOS	3			06530	Granite	Kataev volcanoplutonic complex	Late Triassic	70.2	0.27	15.3		1.39	0.56	0.03	0.4	0.75	4.96	5.03	0.1	0.57		51.87	90.63	11.21	35.48	5.47	0.99	2.47	0.42	2.34	0.45	1.13	0.17	1.06	0.22	16.43		86.75	277.04
307	Northern Mongolia	Nrothern MOS	3			05154	Granite	Kataev volcanoplutonic complex	Late Triassic	69.5	0.33	14.7		1.21	1.4	0.04	0.44	1.7	4.46	4.91	0.14	0.68		27.68	52.85	6.42	17.46	3.02	0.45	2.24	0.31	1.71	0.36	1.15	0.19	1.18	0.20	14.81		82.08	416.09
308	Central-western Mongolia	Central MOS	3			4	Granodiorite	Khentey batholith	Late Triassic	64.8	0.78	17		1.77	2.05	0.12	1.39	3.02	4.94	3.59	0.18	0.5																			
309	Central-western Mongolia	Central MOS	3			5	Gneissic tonalite	Khentey batholith	Late Triassic	66.3	0.54	16.2		0.91	2.64	0.05	1.21	3.09	4.61	2.92	0.12	0.47																			
310	Central-western Mongolia	Central MOS	3			6	Gneissic granite	Khentey batholith	Late Triassic	68.3	0.4	17.4		0.46	1.98	0.09	0.84	1.63	4.33	3.88	0.14	0.41																			
311	Central-western Mongolia	Central MOS	3			7	Granite	Khentey batholith	Late Triassic	67.8	0.43	17.1		0.57	2.14	0.08	0.93	1.99	4.4	3.64	0.14	0.42																			
312	Central-western Mongolia	Central MOS	3			9	Gneissic granite	Khentey batholith	Late Triassic	74.6	0.12	13.8		0.84	0.99	0.03	0.28	0.82	3.76	4.28	0.06	0.39																			
313	Central-western Mongolia	Central MOS	3			25	Granodiorite	Khentey batholith	Late Triassic	67.9	0.46	15.6		0.6	3.28	0.05	1.01	2.38	4.14	3.59	0.06	0.24																			
314	Central-western Mongolia	Central MOS	3			26	Granite	Khentey batholith	Late Triassic	72.5	0.26	14.1		0.5	0.81	0.02	0.58	1.29	3.77	4.15	0.02	0.3																			
315	Central-western Mongolia	Central MOS	3			27	Granite	Khentey batholith	Late Triassic	72.1	0.26	14.1		0.47	2.28	0.02	0.43	1.25	3.8	3.9	0.02	0.17																			
316	Central-western Mongolia	Central MOS	3			28	Granite	Khentey batholith	Late Triassic	71.7	0.27	14.9		0.18	1.96	0.02	0.56	1.31	3.75	4.09	0.03	0.45																			
317	Central-western Mongolia	Central MOS	3			30	Gneissic granite	Khentey batholith	Late Triassic	66.9	0.57	15.5		0.86	2.72	0.05	1.8	2.87	4.12	3.29	0.08	0.4																			
318	Central-western Mongolia	Central MOS	3			31	Gneissic granite	Khentey batholith	Late Triassic	68.7	0.45	15.2		0.67	2.62	0.04	1.21	2.32	4.01	3.59	0.06	0.35																			
319	Central-western Mongolia	Central MOS	3			79	Granite	Khentey batholith	Late Triassic	69.2	0.48	14.7		1.94	1.59	0.06	0.71	1.97	4.1	4.67	0.09	0.52																			
320	Transbaikalia-Mongolia	Notth of the MOS	3			B 163/3*	Syenite	Kharitonovo complex	Late Triassic	65.6	0.63	16	2.56			0.02	0.63	1.55	4.43	7.2	0.11	0.75		120	420		190	25	3.5			4.8				11	1.4	140		110	180
321	Transbaikalia-Mongolia	Notth of the MOS	3			B 275	Alkaline syenite	Kharitonovo complex	Late Triassic	65.7	0.87	16.2	2.49			0.1	0.43	0.25	6.2	5.89	0.1	0.97																44		153	20
322	Transbaikalia-Mongolia	Notth of the MOS	3			A 143	Alkaline syenite	Kharitonovo complex	Late Triassic	64.6	0.75	17.1	2.75			0.1	0.49	0.49	6.71	6.29	0.12	0.34		61.3	112.8		56.1	7.5	1.49	7.68		5.34			0.41	2.25	0.33	53		118	15
323	Transbaikalia-Mongolia	Notth of the MOS	3			B 318-1 (864)	Alkaline syenite	Kharitonovo complex	Late Triassic	65.1	0.82	16.6	4.09			0.09	0.36	0.78	6.17	6.33	0.09	0.12		31.9	64.8		38.9	6	1.12	5.94		4.6			0.28	1.72	0.25	33		100	19
324	Transbaikalia-Mongolia	Notth of the MOS	3			A 145-2	Alkaline syenite	Kharitonovo complex	Late Triassic	65.8	0.71	16.4	2.74			0.18	0.44	0.28	6.6	5.91	0.18	0.55		53.1	128.1		52.4	7.9	1.06	7.82		6.24			0.57	3.09	0.45	81		174	17
325	Transbaikalia-Mongolia	Notth of the MOS	3			B-317 (866-1	Alkaline granite	Kharitonovo complex	Late Triassic	71.9	0.35	12.7	3.79			0.09	0.22	0.42	4.63	4.79	0.04	0.64		63	114		43.81	5.94	0.7	5.96		5.43			0.37	2.14	0.3	45		185	28
326	Transbaikalia-Mongolia	Notth of the MOS	3			B-278/2	Alkaline granite	Kharitonovo complex	Late Triassic	72.9	0.33	12.4	2.61			0.08	0.15	0.26	5.08	4.85	0.01	0.78		53	120		39	5.7	0.72		1.1					4.3	0.69	30		201	35
327	Transbaikalia-Mongolia	Notth of the MOS	3			865	Alkaline syenite	Kharitonovo complex	Late Triassic	65.7	0.54	17	3.29			0.06	0.39	0.36	6.36	6.09	0.09	0.73		36.3	88.2		36.8	5.6	0.87	5.71		4.48			0.36	2.24	0.33	32		190	9
328	Transbaikalia-Mongolia	Notth of the MOS	3			866	Alkaline syenite	Kharitonovo complex	Late Triassic	66.1	0.73	15.7	4.72			0.1	0.44	0.45	5.9	5.65	0.06	0.3		48.4	100.6		49.4	8.4	1.3	7.38		5.01			0.33	2.11	0.3	37		177	10
329	Transbaikalia-Mongolia	Notth of the MOS	3			A 145a	Alkaline granite	Kharitonovo complex	Late Triassic	67.2	0.77	15.3	4.28			0.17	0.43	0.08	5.88	5.17	0.13	0.51		84.2	175.1		73.9	9.7	1.31	9.77		7.98			0.68	4.28	0.62	72		322	13
330	Transbaikalia-Mongolia	Notth of the MOS	3			864-1	Alkaline granite	Kharitonovo complex	Late Triassic	73.3	0.29	11.3	4.41			0.08	0.18	0.3	3.98	5.25	0.03	0.59		33.2	60.2		18.4	3.7	0.34	3.28		3.74			0.3	1.73	0.25	59		244	4
331	Transbaikalia-Mongolia	Notth of the MOS	3			A 149	Alkaline granite	Kharitonovo complex	Late Triassic	64.8	0.33	17.1	3.36			0.16	0.51	1.18	5.41	6.83	0.12	0.27		29.7	65.3		38.1	6	1.74	5.74		3.81			0.29	1.62	0.25	33		80	41
332	Transbaikalia-Mongolia	Notth of the MOS	3			B 440	Comendite	Tsagan–Hurtei volcanic suite	Late Triassic	74.5	0.29	10.7	4.08			0.06	0.23	0.4	3.22	5.95	0.04	0.64		91	180		72	12	1.5		1.8					6.1	0.95	52		207	21
333	Transbaikalia-Mongolia	Notth of the MOS	3			B 444	Comendite	Tsagan–Hurtei volcanic suite	Late Triassic	72	0.34	11	6.7			0.15	0.2	0.2	5.03	4.17	0.05	0.59																110		135	18
334	Transbaikalia-Mongolia	Notth of the MOS	3			B 445-1	Comendite	Tsagan–Hurtei volcanic suite	Late Triassic	70.9	0.53	11.9	5.38			0.16	0.36	0.76	4.56	5.15	0.1	0.68		120	200		100	17	2.4		3.1					11	1.7	190		172	57
335	Transbaikalia-Mongolia	Notth of the MOS	3			A 13-1	Alkaline granite	Tsagan–Hurtei volcanic suite	Late Triassic	71.4	0.46	12.6	3.55			0.12	0.35	0.34	4.84	5.09	0.04	0.48		84	213.9	22.8	82.4	16.8	1.84	16.03	3.14	19.45	4.06	12.29	2.06	11.9	1.75	134		161	18
336	Transbaikalia-Mongolia	Notth of the MOS	3			L 716	Alkaline granite	Tsagan–Hurtei volcanic suite	Late Triassic	74.2	0.23	11.1	3.7			0.1	0.13	0.21	4.74	4.53	0.02	0.76		74.5	155.2	17	58.2	10.5	0.42	9.48	1.64	10.77	2.5	7.94	1.34	8.2	1.4	78		132	7
337	Transbaikalia-Mongolia	Notth of the MOS	3			L 722	Alkaline granite	Tsagan–Hurtei volcanic suite	Late Triassic	73	0.42	12.1	3.71			0.13	0.23	0.26	4.98	4.68	0.02	0.57		68.1	167.6	17.5	65.4	12.5	1.52	10.7	1.93	11.09	2.29	7.24	1.12	7.49	1.16	70		140	16
338	Transbaikalia-Mongolia	Notth of the MOS	3			L 725	Alkaline granite	Tsagan–Hurtei volcanic suite	Late Triassic	72.3	0.42	12.5	3.62			0.12	0.3	0.31	5.12	4.94	0.03	0.5		81.5	153.6	18.2	64.6	11.3	1.42	10.07	1.75	9.23	2.02	5.8	0.91	5.65	0.91	56		130	9
339	Transbaikalia-Mongolia	Notth of the MOS	3			L 727	Alkaline syenite	Tsagan–Hurtei volcanic suite	Late Triassic	66.6	0.89	15	3.62			0.17	0.62	0.59	6.49	5.45	0.1	0.46		53	119.3	14.1	56.2	10.9	1.98	10.14	1.8	10.65	2.28	6.5	1.02	6.64	1.01	72		73	27
340	Transbaikalia-Mongolia	Notth of the MOS	3			L 728	Alkaline syenite	Tsagan–Hurtei volcanic suite	Late Triassic	64.9	0.91	16.2	3.22			0.14	0.75	0.56	6.71	5.72	0.11	0.28		58	129	14.6	57.5	11	1.84	8.62	1.42	7.13	1.4	3.66	0.55	3.5	0.53	43		55	10
341	Transbaikalia-Mongolia	Notth of the MOS	3			B 440	Comendite	Tsagan–Hurtei volcanic suite	Late Triassic	74.5	0.29	10.7	4.08			0.06	0.23	0.4	3.22	5.95	0.04	0.64		91	180		72	12	1.5		1.8					6.1	0.95	52		207	21
342	Transbaikalia-Mongolia	Notth of the MOS	3			A 13	Peralkaline granite	Tsagan–Hurtei volcanic suite	Late Triassic	74.4	0.25	11.4		1.1	3.08	0.1	0.14	0.2	4.29	4.44	0.02	0.54		19	82		15	3.5	0.32	4.4	1.22	9.8	2.36	7.64	1.31	9.14	1.44	70		162	6
343	Transbaikalia-Mongolia	Notth of the MOS	3			716	Peralkaline granite	Tsagan–Hurtei volcanic suite	Late Triassic	74.2	0.23	11.1		0.88	2.56	0.1	0.13	0.21	4.74	4.53	0.02	0.76		75	155	17	58	10.5	0.42	9.5	1.64	10.8	2.5	7.94	1.34	8.2	1.4	63		133	3
344	Transbaikalia-Mongolia	Notth of the MOS	3			722	Peralkaline granite	Tsagan–Hurtei volcanic suite	Late Triassic	73	0.42	12.1		2.2	1.37	0.13	0.23	0.26	4.98	4.68	0.02	0.57		68	168	17.5	65	12.5	1.52	10.7	1.93	11.1	2.29	7.24	1.12	7.49	1.16	52		133	13
345	Transbaikalia-Mongolia	Notth of the MOS	3			725	Peralkaline granite	Tsagan–Hurtei volcanic suite	Late Triassic	72.3	0.42	12.5		1.77	1.68	0.12	0.3	0.31	5.12	4.94	0.03	0.5		81	154	18.2	65	11.3	1.42	10.1	1.75	9.2	2.02	5.8	0.91	5.65	0.91	46		133	7
346	Transbaikalia-Mongolia	Notth of the MOS	3			727	Peralkaline syenite	Tsagan–Hurtei volcanic suite	Late Triassic	66.6	0.89	15		2.07	1.41	0.17	0.62	0.59	6.49	5.45	0.1	0.46		53	119	14.1	56	10.9	1.98	10.1	1.8	10.7	2.28	6.5	1.02	6.64	1.01	61		76	31
347	Transbaikalia-Mongolia	Notth of the MOS	3			728	Peralkaline syenite	Tsagan–Hurtei volcanic suite	Late Triassic	64.9	0.91	16.2		2.01	1.1	0.14	0.75	0.56	6.71	5.72	0.11	0.28		43	91	12.8	53	9.6	1.56	8.2	1.2	6.4	1.26	3.48	0.49	3.17	0.49	31		51	6
348	Transbaikalia-Mongolia	Notth of the MOS	3			KH 14	Peralkaline syenite	Kharitonovo complex	Late Triassic	63.3	1.27	16.2		2.61	1.68	0.15	0.72	1.43	5.46	6.09	0.28	1.08		96	210		90	15	3.5		2.2					4.9	0.73	50		120	65
349	Transbaikalia-Mongolia	Notth of the MOS	3			866	Peralkaline syenite	Kharitonovo complex	Late Triassic	66.1	0.73	15.7	4.29			0.1	0.44	0.45	5.9	5.65	0.06	0.3		48	101		49	8.4	1.3	7.4		5			0.33	2.11	0.3	37		177	10
350	Transbaikalia-Mongolia	Notth of the MOS	3			3008	Alkali-feldspar syenite	Kharitonovo complex	Late Triassic	71.2	0.34	14.6	2.64			0.08	0.14	0.3	4.62	5.33	0.06	1		62	132		50	8.3	0.51	5.2		3.6			0.28	1.62	0.23	56		377	36
351	Transbaikalia-Mongolia	Notth of the MOS	3			871	Syenite	Kharitonovo complex	Late Triassic	63.4	0.67	18.4		0.44	2.18	0.1	0.61	0.83	5.43	6.27	0.16	0.69		30	55		23	6.8	2.1	8.1		6.6			0.67	3.2	0.5	23		82	93
352	Transbaikalia-Mongolia	Notth of the MOS	3			A-92-a	Syenite	Kharitonovo complex	Late Triassic	63.2	1.11	16.6		0.55	2.76	0.17	0.71	0.51	6.09	5.98	0.24	1.28		61	112		56	7.54	1.49	7.68		5.34			0.41	2.25	0.33	53		90	28
353	Transbaikalia-Mongolia	Notth of the MOS	3			Oro-1	Granite	Kharitonovo complex	Late Triassic	75.7	0.34	11.6	4.37			0.15	0.35	0.51	2.21	4.71	0.01			56.97	117.4	11.62	36.68	7.42	0.8	7.02	1.41	9.32	3.26	7.91	1.43	10.07	1.54	66.4		235	13.25
354	Transbaikalia-Mongolia	Notth of the MOS	3			Oro-9	Granite	Kharitonovo complex	Late Triassic	73.3	0.38	15.4	1.36			0.03	0.21	0.52	2.98	5.65	0.02			123.2	252.1	30.14	103.5	18.59	1.88	13.98	2.33	13.25	2.93	8.9	1.56	9.81	1.49	83.59		271	30.4
355	Transbaikalia-Mongolia	Notth of the MOS	3			Oro-10	Granite	Kharitonovo complex	Late Triassic	69.5	0.76	15.2	3.3			0.2	0.21	0.57	4.25	5.83	0.02			124.2	244.1	25.25	84.68	13.85	1.48	10.45	1.6	8.12	2.18	6.36	1.06	7.09	1.11	77.81		267	34.51
356	Transbaikalia-Mongolia	Notth of the MOS	3			Oro-35	Granite	Kharitonovo complex	Late Triassic	72.2	0.43	14	2.36			0.03	0.05	0.21	3.38	6.96	0.02			64.4	111.5	16.05	56.25	10.53	1.04	9.19	1.71	10.6	2.45	7.37	1.16	7.56	1.28	57.84		246	8.48
357	Transbaikalia-Mongolia	Notth of the MOS	3	105.00	46.15	D0925	Granodiorite	296 Intrusion	296	66.7	0.46	16.7			2.58	0.04	0.76	2.68	4.63	3.27	0.14	1.45		28.0	54.0	6.5	25.0	4.00	0.92	2.70	0.36	1.63	0.29	0.76	0.12	0.75	0.11	9.9	1.12	73	1260
358	Transbaikalia-Mongolia	Notth of the MOS	3	105.00	46.16	D0926	Granodiorite	296 Intrusion	296	66.6	0.39	15.8			2.97	0.04	0.61	2.53	4.74	3.24	0.13	1.63		35.8	65.1	7.6	25.6	4.19	1.00	2.73	0.38	1.91	0.31	0.89	0.13	0.78	0.12	10.3	0.8	65	1377
359	Transbaikalia-Mongolia	Notth of the MOS	3	105.00	46.18	D0927	Granodiorite	296 Intrusion	296	65.8	0.61	15			3.94	0.1	1.29	2.63	4.05	4.21	0.19	2.17		29.2	59.1	6.7	23.7	4.46	1.02	3.54	0.56	3.24	0.66	1.93	0.27	1.87	0.33	18.7	4.2	137	519
360	Transbaikalia-Mongolia	Notth of the MOS	3	104.40	47.09	D0802	Rhyolite	Batkhaan	274	77.7	0.14	11.7			3.04	0.02	0.1	0.11	4.19	3.37	0.01	0.34		38.6	78.4	10.2	38.6	7.67	0.22	7.19	1.28	7.36	1.63	4.75	0.77	4.97	0.77	39.81	1.691	114.09	14.26
361	Transbaikalia-Mongolia	Notth of the MOS	3	104.40	47.09	D0803	Rhyolite	Batkhaan	274	74.6	0.19	13.4			2.42	0.03	0.18	0.18	3.86	4.91	0.02	0.27		52.2	92.5	13.4	52.1	10.69	0.36	10.47	1.69	9.50	1.92	5.61	0.88	5.65	0.88	48.564	4.074	181.89	24.41
362	Transbaikalia-Mongolia	Notth of the MOS	3	104.40	47.10	D0804	Rhyolite	Batkhaan	274	74.9	0.17	13.7			1.65	0.03	0.3	0.33	4.82	3.99	0.04	0.18		50.2	117.6	12.5	48.3	9.30	0.21	8.86	1.44	8.45	1.93	5.37	0.86	5.42	0.86	45.207	2.1	96.786	21.77
363	Transbaikalia-Mongolia	Notth of the MOS	3	104.77	47.08	D1709	Rhyolite	Batkhaan	274	74.7	0.15	11.3			2.53	0.02	0.13	0.05	3.03	4.34	0.04	2.33		48.0	104.0	11.8	45.5	10.40	0.76	10.40	1.82	10.70	2.13	6.66	0.99	6.62	1.00	62.3	2.5	153	20
364	Transbaikalia-Mongolia	Notth of the MOS	3	104.77	47.08	D1714	Rhyolite	Batkhaan	274	67.9	0.69	13			5.98	0.07	0.38	1.75	3.85	2.63	0.32	1.46		24.4	52.8	6.7	26.4	6.45	1.32	7.04	1.14	6.64	1.32	3.88	0.60	4.08	0.69	35.5	2.9	92	157
365	Transbaikalia-Mongolia	Notth of the MOS	3	104.34	47.07	D1013	Granosyenite	Batkhaan	282	74.5	0.17	14.3			2.52	0.03	0.19	0.78	4.3	4.74	0.03	0.28		32.8	66.1	7.8	30.1	6.12	0.62	6.12	1.05	6.90	1.52	4.31	0.70	5.39	0.73	55.202	2.437	120.92	65
366	Transbaikalia-Mongolia	Notth of the MOS	3	104.34	47.07	D1015	Granosyenite	Batkhaan	282	73.4	0.21	14.2			2.51	0.04	0.23	0.75	4.98	4.94	0.04	0.28		31.7	68.7	7.9	30.5	6.32	0.67	6.01	1.11	7.10	1.59	4.61	0.72	4.99	0.74	51.202	3.637	122.92	72.8
367	Transbaikalia-Mongolia	Notth of the MOS	3	104.34	47.07	D1016	Granosyenite	Batkhaan	282	77.3	0.12	11.5			2.77	0.02	0.14	0.08	4.19	4.55	0.01	0.31		32.7	65.6	7.9	29.9	5.95	0.52	5.73	1.03	6.46	1.41	4.07	0.65	4.98	0.62	49.01	1.226	110.4	55.65
368	Transbaikalia-Mongolia	Notth of the MOS	3	104.34	47.07	D1018	Granosyenite	Batkhaan	282	64.6	0.56	15.3			3.53	0.06	3.08	3.63	4.11	3.44	0.16	0.46		32.2	67.9	7.8	30.0	5.99	0.58	5.99	1.09	6.87	1.49	4.20	0.67	5.12	0.71	50.4	2.626	110.4	58.4
369	Transbaikalia-Mongolia	Notth of the MOS	3	104.23	47.10	D1703	Granosyenite	Batkhaan	282	75.4	0.14	13.2			1.41	0.03	0.19	0.71	3.52	4.92	0.11	0.16		22.8	55.9	6.0	24.5	5.82	0.35	7.40	1.10	8.01	1.59	5.23		5.48		56.16	1.024	195.8	22
370	Transbaikalia-Mongolia	Notth of the MOS	3	104.33	47.10	D1705	Granosyenite	Batkhaan	282	75.6	0.14	11.4			2.11	0.03	0.11	0.12	3.6	3.77	0.04	1.63		24.3	62.4	6.5	24.2	6.04	0.42	6.64	1.33	8.82	1.85	5.63	0.87	6.11	0.94	52.3	1.6	148	17
371	Transbaikalia-Mongolia	Notth of the MOS	3	104.47	47.11	D1706	Monzonite	Batkhaan	282	74.2	0.16	13.1			1.97	0.03	0.1	0.12	4.19	4.53	0.04	0.47		19.6	50.0	5.2	21.0	5.00	0.36	5.60	1.00	7.40	1.64	4.90	0.74	4.70	0.69	48	1.62	153	17.8
372	Transbaikalia-Mongolia	Notth of the MOS	3	104.15	47.10	D1707	Monzogranite	Batkhaan	282	74.2	0.14	12.5			2.03	0.03	0.09	0.1	4.21	4.3	0.05	1.48		25.6	64.8	7.0	25.7	6.34	0.44	7.09	1.39	9.33	1.95	6.03	0.91	6.38	0.99	55.4	1.7	151	17
373	Transbaikalia-Mongolia	Notth of the MOS	3	104.06	47.08	D1713	Monzogranite	Batkhaan	282	60.6	0.72	18.6			3.04	0.06	0.3	5.91	5.17	1.64	0.25	2.7		17.3	37.8	4.8	19.3	4.57	3.30	4.77	0.71	4.24	0.81	2.32	0.35	2.20	0.37	22	0.8	40	420
374	Transbaikalia-Mongolia	Notth of the MOS	3	104.04	47.14	D1721	Monzonite	Batkhaan	282	59.5	0.72	19.7			2.91	0.05	0.31	5.5	5.89	1.83	0.26	2.96		17.1	38.3	4.9	19.9	4.57	3.36	4.76	0.76	4.42	0.85	2.42	0.32	2.12	0.35	22.4	0.7	39	456
375	Transbaikalia-Mongolia	Notth of the MOS	3	104.13	47.07	D1710	Syenogranite	Batkhaan	282	72.9	0.18	14.9			1.19	0.02	0.11	0.75	4.36	4.37	0.06	0.13		21.1	54.4	5.9	24.5	5.81	0.45	6.80	1.30	7.99	1.62	4.99		4.25		48	1.01	140	58
376	Transbaikalia-Mongolia	Notth of the MOS	3	104.13	47.12	D1710-1	Monzonite	Batkhaan	282	60.9	0.98	16.5			6.51	0.13	1.03	3.42	4.86	2.35	0.24	1.3		22.0	44.0	5.7	23.0	5.20	1.63	5.40	0.83	5.00	1.10	3.10	0.49	3.20	0.49	30	2.4	51	245
377	Transbaikalia-Mongolia	Notth of the MOS	3	104.40	47.10	D0808	Granite	Delgerkhaan	236	71.2	0.29	16.4			0.9	0.01	0.59	1.84	4.56	3.83	0.09	0.1		18.5	31.2	3.6	12.5	2.07	0.87	1.65	0.23	1.11	0.22	0.57	0.09	0.56	0.09	5.484	2.509	106.93	460
378	Transbaikalia-Mongolia	Notth of the MOS	3	104.54	46.90	D0812	Granodiorite	Delgerkhaan	236	64.5	0.31	14.5			2.34	0.05	1.35	4.4	4.44	3.29	0.1	0.26		16.5	29.5	3.7	13.6	2.62	0.77	2.22	0.34	1.81	0.35	0.97	0.15	0.98	0.16	9.068	1.558	87.085	378
379	Transbaikalia-Mongolia	Notth of the MOS	3	104.51	46.94	D0815	Granodiorite	Delgerkhaan	236	68.4	0.5	15.1			2.85	0.05	1.92	2.7	4.06	3.95	0.15	0.32		37.3	74.8	8.4	29.6	4.86	1.08	3.92	0.56	2.75	0.53	1.50	0.23	1.52	0.24	14.12	8.453	165.36	415
380	Transbaikalia-Mongolia	Notth of the MOS	3	104.46	46.97	D0817	Granodiorite	Delgerkhaan	236	71.8	0.28	15.1			1.53	0.03	0.83	1.79	4.34	3.98	0.09	0.17		24.7	42.3	5.1	17.9	2.77	0.84	2.15	0.29	1.40	0.27	0.75	0.12	0.77	0.13	6.965	4.378	112.3	449
381	Transbaikalia-Mongolia	Notth of the MOS	3	104.50	46.99	D0818	Granodiorite	Delgerkhaan	236	65.4	0.55	15.3			3.79	0.06	3.01	3.51	4.06	3.04	0.17	0.42		23.5	49.2	6.4	24.6	4.71	1.16	3.61	0.52	2.45	0.47	1.38	0.21	1.38	0.22	12.925	3.257	100.24	482
382	Transbaikalia-Mongolia	Notth of the MOS	3	104.52	46.98	D0819	Monzodiorite	Delgerkhaan	236	65	0.57	14.9			3.82	0.07	3.11	3.52	3.89	3.52	0.16	0.43		26.4	51.6	6.5	24.8	4.28	1.19	3.47	0.49	2.48	0.48	1.27	0.19	1.23	0.20	11.464	4.644	113.4	492
383	Transbaikalia-Mongolia	Notth of the MOS	3	104.52	46.98	D0831	Granite	Delgerkhaan	236	70.6	0.25	15.4			1.33	0.02	0.75	1.79	4.4	4.31	0.08	0.15		21.1	34.9	4.4	15.1	2.45	0.69	1.82	0.25	1.28	0.24	0.64	0.10	0.64	0.11	6.145	4.097	121.43	485
384	Transbaikalia-Mongolia	Notth of the MOS	3	104.61	46.83	D1001	Granite	Delgerkhaan	236	62.5	0.64	15.2			4.27	0.07	3.29	4.05	3.97	3.22	0.19	0.47		35.8	69.0	8.2	29.7	5.14	1.29	4.06	0.56	2.73	0.52	1.43	0.21	1.37	0.22	13.205	7.03	111.9	591
385	Transbaikalia-Mongolia	Notth of the MOS	3	104.60	46.80	D1003	Porphyritic granite	Delgerkhaan	236	66.2	0.54	15.6			3.19	0.05	2.61	3.34	4.31	3.21	0.16	0.35		27.9	52.5	6.4	23.3	4.21	1.03	3.14	0.44	2.17	0.44	1.19	0.19	1.28	0.20	12.789	6.566	110.38	576
386	Transbaikalia-Mongolia	Notth of the MOS	3	104.56	46.74	D1006	Granodiorite	Delgerkhaan	236	70.8	0.33	14.6			2.14	0.04	1.23	2.25	4.37	3.32	0.17	0.24		30.0	55.5	6.3	23.4	4.00	0.67	3.18	0.48	2.76	0.57	1.72	0.27	1.93	0.32	17.063	16.685	132.11	439
387	Transbaikalia-Mongolia	Notth of the MOS	3	104.57	46.73	D1008	Granodiorite	Delgerkhaan	236	71.4	0.18	16			1.45	0.05	0.42	1.61	4.97	3.8	0.14	0.16		55.2	97.3	10.6	35.8	4.87	0.85	3.39	0.40	1.99	0.39	1.08	0.16	1.09	0.17	10.943	9.65	119.75	463
388	Transbaikalia-Mongolia	Notth of the MOS	3	104.58	46.72	D1009	Granodiorite	Delgerkhaan	236	70.5	0.3	15.2			1.75	0.02	0.89	1.77	4.45	4.15	0.1	0.2		25.0	49.0	5.3	19.0	3.14	0.56	2.34	0.30	1.55	0.30	0.81	0.11	0.74	0.12	8.117	7.443	138.27	478
389	Transbaikalia-Mongolia	Notth of the MOS	3	104.55	46.64	D1011	Granodiorite	Delgerkhaan	236	68.1	0.38	15.3			2.3	0.05	1.63	2.44	4.38	3.57	0.12	0.26		32.9	59.9	6.4	22.4	3.59	0.86	2.77	0.37	1.99	0.37	1.02	0.16	1.10	0.18	10.653	8.042	131.46	498
390	Transbaikalia-Mongolia	Notth of the MOS	3	104.49	46.81	D1012	Monzogranite	Delgerkhaan	236	64.6	0.56	15.3			3.53	0.06	3.08	3.63	4.11	3.44	0.16	0.39		29.5	61.6	7.4	28.2	5.02	1.12	3.80	0.53	2.62	0.51	1.42	0.23	1.44	0.23	14.731	5.765	108.52	598
391	Transbaikalia-Mongolia	Notth of the MOS	3	104.56	46.77	D1024	Granodiorite	Delgerkhaan	236	67.4	0.37	16.6			2.36	0.04	1.71	3.18	4.74	3.22	0.14	0.26		25.2	39.6	4.6	16.0	2.36	0.71	1.94	0.24	1.14	0.22	0.60	0.09	0.63	0.10	6.551	4.481	77.333	653
392	Transbaikalia-Mongolia	Notth of the MOS	3	104.55	46.64	D1031	Granite	Delgerkhaan	236	69.5	0.29	15.6			1.74	0.04	1.25	2.39	4.65	3.32	0.1	0.19		21.8	38.6	4.2	14.8	2.44	0.81	1.87	0.25	1.29	0.27	0.72	0.10	0.68	0.11	7.02	6.886	112.8	529
393	Transbaikalia-Mongolia	Notth of the MOS	3	104.51	46.38	D0931	Granite	Delgerkhaan	236	74.6	0.14	12.9			1.89	0.02	0.01	0.23	4.53	4.42	0.02	0.21			49.4	5.4	19.2					1.60	0.31	0.84	0.12	0.76	0.12	8.312	7.657	149.69	489
394	Transbaikalia-Mongolia	Notth of the MOS	3	104.51	46.38	D0933	Granite	Delgerkhaan	236	63.8	0.79	15.3			4.53	0.06	3.82	3.3	4.35	3.78	0.23	0.5			49.2	6.4	24.6					2.45	0.47	1.38	0.21	1.38	0.22	12.925	3.257	100.24	482
395	Transbaikalia-Mongolia	Notth of the MOS	3	104.04	47.14	D1718	Granite	Delgerkhaan	236	76.5	0.07	12.9			1.33	0.02	0.06	0.61	3.86	3.94	0.04	1.24		12.8	23.7	2.4	7.8	1.22	0.42	0.85	0.12	0.67	0.14	0.34	0.05	0.39	0.07	3.6	1.5	89	263
396	Transbaikalia-Mongolia	Notth of the MOS	3	104.81	46.91	D1729	Quartz-diorite	Delgerkhaan	236	61.7	0.37	15.1			4.78	0.09	3.91	3.43	4.34	4.22	0.24	0.53			51.6	6.5	24.8					2.48	0.48	1.27	0.19	1.23	0.20	11.464	4.644	113.4	497
397	Transbaikalia-Mongolia	Notth of the MOS	3	104.61	46.91	D1730	Granite	Delgerkhaan	236	71	0.33	14.7			2.01	0.04	1.14	2.23	4.18	3.26	0.09	0.43		26.0	46.0	5.4	19.6	3.60	0.66	2.90	0.43	2.50	0.45	1.28	0.21	1.35	0.20	13.7	6	119	399
398	Transbaikalia-Mongolia	Notth of the MOS	3	104.62	46.67	D1742	Monzogranite	Delgerkhaan	236	69.5	0.34	15			2.19	0.04	1.22	2.11	4.28	3.78	0.12	0.24		13.1	30.5	3.2	11.8	2.73	0.59	3.22	0.30	2.45	0.35	1.41		1.49		15.24	4.347	154.9	248

No.	Location	Magmatic belt	Group	Longitude	Latitude	Sample	Rock type	Pluton	Age（Ma）	SiO_2	TiO_2	Al_2O	TFe_2	Fe_2O	FeO	MnO	MgO	CaO	Na_2O	K_2O	P_2O_5	LOI	$Mg^{\#}$	La	Ce	Pr	Nd	Sm	Eu	Gd	Tb	Dy	Ho	Er	Tm	Yb	Lu	Y	Cs	Rb	Sr
399	Transbaikalia-Mongolia	Notth of the MOS	3	104.62	46.67	D1742-1	Monzogranite	Delgerkhaan	236	64.4	0.57	15.1			3.64	0.07	3.18	3.78	4.03	3.07	0.18	0.41																22		202	495
400	Transbaikalia-Mongolia	Notth of the MOS	3	104.74	46.78	D1746	Monzogranite	Delgerkhaan	236	67.4	0.71	14.1			6.11	0.08	0.5	1.68	4.24	3.01	0.21	2.75		15.4	56.6	5.0	20.2	5.28	1.24	6.10	1.10	6.67	1.40	4.03	0.60	4.17	0.66	35.9	4	89	174
401	Transbaikalia-Mongolia	Notth of the MOS	3	104.70	46.85	D1747	Granodiorite	Delgerkhaan	236	65.8	0.48	15.9			3.07	0.05	2.49	3.27	4.32	3.23	0.16	0.34																13		144	438
402	Transbaikalia-Mongolia	Notth of the MOS	3	104.29	46.94	D1724	Granite	Zambalkhudag	220	75.3	0.03	13.4			0.8	0.02	0.03	0.75	4.48	3.91	0.02	0.09																20		221	488
403	Transbaikalia-Mongolia	Notth of the MOS	3	104.35	46.94	D1725	Granite	Zambalkhudag	220	74.5	0.15	14.3			1.22	0.04	0.31	1.2	3.88	4.27	0.03	0.14																19		131	258
404	Transbaikalia-Mongolia	Notth of the MOS	3	104.34	46.94	D1726	Granodiorite	Zambalkhudag	220	66.3	0.64	16.1			2.96	0.05	1.45	2.59	3.9	4.09	0.23	0.93		36.0	74.0	8.6	32.0	5.80	1.27	4.70	0.68	3.50	0.68	2.00	0.31	1.85	0.27	19.5	7.1	164	425
405	Transbaikalia-Mongolia	Notth of the MOS	3	104.32	46.94	D1727	Granite	Zambalkhudag	220	71.3	0.33	13.2			2.18	0.06	0.89	3.28	3.21	3.19	0.09	2.15		15.2	36.1	3.7	13.5	2.85	0.68	2.24	0.32	1.65	0.31	0.85	0.12	0.74	0.12	8.3	1.5	113	156
406	Transbaikalia-Mongolia	Notth of the MOS	3	104.32	46.94	D1728	Granite	Zambalkhudag	220	75.4	0.14	13.1			1.34	0.04	0.3	1.49	3.25	3.95	0.05	1.3		13.9	28.7	3.2	12.2	3.24	0.64	3.15	0.50	2.82	0.57	1.64	0.28	2.02	0.35	16.5	2.6	119	251
407	Transbaikalia-Mongolia	Notth of the MOS	3	104.73	46.26	D0930	Granite	Zambalkhudag	220	61.4	0.7	14.9			4.79	0.06	3.36	3.54	4.43	3.34	0.21	3.03		69.4	142.0	14.5	47.4	7.58		5.20	0.66	3.35	0.60	1.62	0.21	1.47	0.23	17.3	5.6	180	477
408	Transbaikalia-Mongolia	Notth of the MOS	3	104.51	46.38	D0932	Granite	Zambalkhudag	220	61.8	0.82	15.2			4.63	0.08	3.65	3.41	3.8	4.26	0.24	2.2		47.7	98.3	11.0	38.8	7.10	1.34	5.13	0.68	3.74	0.70	1.87	0.26	1.71	0.27	19.6	6.6	185	503
409	Transbaikalia-Mongolia	Notth of the MOS	3	104.19	47.00	D1734	Trachyandesite	Triassic volcanic	240	60.2	0.92	16.2			6.2	0.05	2.83	3.74	3.79	3.68	0.29	0.69		41.3	61.6	7.4	28.2	2.07	0.87	1.75	0.33	2.62	0.51	1.42	0.10	1.44	0.23	14.731	5.765	108.52	598
410	Transbaikalia-Mongolia	Notth of the MOS	3	104.22	46.68	D1735	Trachyandesite	Triassic volcanic	240	65.9	0.71	15.3			4.16	0.04	2.15	2.15	3.26	5.32	0.21	0.46		37.3	46.7	5.5	19.4	5.33	1.23	3.02	0.69	1.57	0.31	0.86	0.26	0.81	0.13	8.234	5.15	115.73	481
411	Transbaikalia-Mongolia	Notth of the MOS	3	104.23	46.68	D1736	Trachyandesite	Triassic volcanic	240	59.1	0.95	15.1			5.72	0.1	5.61	5.24	3.28	3.28	0.3	0.64		45.7	97.3	10.6	35.8	2.62	0.91	2.22	0.34	1.99	0.39	1.08	0.21	1.09	0.17	10.943	9.65	119.75	463
412	Transbaikalia-Mongolia	Notth of the MOS	3	104.22	46.68	D1737	Andesite	Triassic volcanic	240	59.5	0.92	15.2			5.49	0.08	4.61	5.31	3.79	3.67	0.3	0.61		59.0	77.3	8.8	29.0	3.82	0.82	3.89	0.55	2.01	0.41	1.21	0.26	1.22	0.21	11.943	8.79	118.98	476
413	Transbaikalia-Mongolia	Notth of the MOS	3	104.25	46.69	D1738	Trachyandesite	Triassic volcanic	240	65.4	0.69	14.8			4.44	0.04	1.19	2.35	2.94	5.31	0.19	2.35		39.5	75.9	8.3	28.3	4.86	1.18	3.72	0.50	2.70	0.57	1.36	0.18	1.21	0.18	13.7	7.3	169	586
414	Transbaikalia-Mongolia	Notth of the MOS	3	104.09	46.70	D1739	Dacite	Triassic volcanic	240	58.6	0.92	14.8			5.55	0.11	3.74	5.66	2.82	2.31	0.3	3.68		43.5	85.8	9.7	36.5	6.60	1.63	5.08	0.70	3.70	0.57	1.92	0.26	1.71	0.27	19.7	3.2	67	789
415	Transbaikalia-Mongolia	Notth of the MOS	3	104.67	46.68	D1743	Trachyandesite	Triassic volcanic	240	68.1	0.7	13.6			6.17	0.08	0.5	1.76	3.9	2.78	0.2	1.71		16.2	58.3	5.1	20.5	5.65	1.30	6.22	1.13	6.94	0.57	4.08	0.63	4.04	0.65	35.6	4.1	94	168
416	Transbaikalia-Mongolia	Notth of the MOS	3	104.57	46.73	D1744	Trachyandesite	Triassic volcanic	240	59.8	1	15.5			6.7	0.15	7.28	2.38	3.79	1.41	0.25	0.75		41.7	47.8	7.6	21.5	4.29	1.59	2.89	0.63	3.33	0.49	1.23	0.19	1.84	0.23	6.145	6.2	121.43	485
417	Transbaikalia-Mongolia	Notth of the MOS	3	104.57	46.73	D1745	Andesite	Triassic volcanic	240	60.3	0.79	14.8			5.67	0.11	4.46	4.09	3.36	4.11	0.11	0.63		49.2	55.5	6.3	23.4	5.65	1.30	6.22	1.13	2.76	0.57	1.72	0.25	1.93	0.32	17.063	16.685	132.11	439

Note: MOS= Mongol-Okhotsk suture; CAOB = Central Asian Orogenic Belt; Bi = biotite; Qtz = quartz; ICWEA = In Chinese with English abtract

Ba	Be	Nb	Ta	Th	U	Pb	Zr	Hf	V	Co	Ni	Cr	W	Cu	Zn	Ga	Ge	Sc	Sn	As	Refereces	
1275		43.00	2.30	13.00	2.50		84.2	2.30	10.20	1.70	4.00	7.30	0.41			26.00					Wang Tao et al., 2004	Journal of Asian Earth Sciences
1234		42.00	3.80	11.00	3.20		476	2.60	27.00	25.00	4.20	6.00	0.38			27.00					Wang Tao et al., 2004	Journal of Asian Earth Sciences
2670		35.00	2.40	35.00	5.50		78.0	1.50	92.00	12.00	6.70	16.00	0.53			32.00					Wang Tao et al., 2004	Journal of Asian Earth Sciences
1600		15.00	1.80	14.00	2.70		69.0	1.90	25.00	2.90	4.00	14.00	0.62			26.00					Wang Tao et al., 2004	Journal of Asian Earth Sciences
1110		43.00	3.30	30.00	4.00		174	4.60	24.00	1.40	4.10	12.00	0.91			29.00					Wang Tao et al., 2004	Journal of Asian Earth Sciences
246		8.00	0.20	104.00	4.20		105	1.70	1.50	0.10	4.00	14.00	0.52			13.00					Wang Tao et al., 2004	Journal of Asian Earth Sciences
171		59.00	5.96	46.30	6.53		168	8.01	10.80	1.46	2.27					35.10		9.41			Wang Tao et al., 2014	American Journal of Science
152		62.30	6.62	29.00	10.00		102	5.59	6.80	0.17	0.82					37.10		11.30			Wang Tao et al., 2014	American Journal of Science
<1		17.40	6.30	23.90	2.20		60.6	6.60	<8	<0.2	3.10					35.90		2.00			Wang Tao et al., 2014	American Journal of Science
<1		15.50	4.90	23.40	1.40		63.8	5.70	<8	<0.2	5.90					33.60		2.00			Wang Tao et al., 2014	American Journal of Science
<1		15.40	5.80	22.90	2.40		76.9	9.90	<8	0.30	2.90					32.90		2.00			Wang Tao et al., 2014	American Journal of Science
3		15.40	7.00	11.70	2.50		29.0	4.90	<8	0.30	4.80					38.50		4.00			Wang Tao et al., 2014	American Journal of Science
285		16.00	1.30	10.60	1.90		53.0	1.70													Yarmolyuk et al.,2001	Petrologiya
612		17.20	1.13	38.90	1.11		354	9.47								21.90					Wang Tao et al., 2014	Acta Petrologica Sinica (ICWEA)
1029		8.34	0.47	87.00	2.32		110	2.80	21.70	3.26	1.74	2.08				22.10					Wang Tao et al., 2014	Acta Petrologica Sinica (ICWEA)
232		9.15	0.96	27.50	5.53		123	4.42								21.60					Wang Tao et al., 2014	Acta Petrologica Sinica (ICWEA)
347		18.40	2.15	35.10	4.86		166	5.79	24.10	2.91	5.08	5.98				23.80					Wang Tao et al., 2014	Acta Petrologica Sinica (ICWEA)
387		13.30	0.98	22.90	4.35		147	5.03								22.30					Wang Tao et al., 2014	American Journal of Science
9.93		18.10	11.10	17.70	1.52		78.5	7.49	3.11	0.16	1.02	1.82				59.00		8.12			Wang Tao et al., 2014	American Journal of Science
208		22.50	3.35	19.30	2.27		236	8.66	21.60	3.09	4.15	7.20				23.60		6.65			Wang Tao et al., 2014	American Journal of Science
3.09		21.80	5.61	31.10	2.59		74.6	5.98	<1.00	0.11	0.62	1.65				36.50		1.95			Wang Tao et al., 2014	American Journal of Science
15		14.86	6.69	20.30	1.76		52.2	4.44	0.60	0.10	1.70										Wang Tao et al., 2014	American Journal of Science
171		59.00	5.96	46.30	6.53		168	8.01	10.80	1.46	2.27	3.05				35.10		9.41			Wang Tao et al., 2014	American Journal of Science
152		62.30	6.62	29.00	10.00		102	5.59	6.76	0.17	0.82	2.06				37.10		11.30			Wang Tao et al., 2014	American Journal of Science
																					Zhao et al., 1993	Science Press, Beijing (ICWEA)
578		7.20	0.87	10.50	2.00		60.1	2.35													Liu Mingqiang et al.,2006	Acta Petrologica et Mineralogica (ICWEA)
536		5.04	0.61	7.66	2.00		104	3.29													Liu Mingqiang et al.,2006	Acta Petrologica et Mineralogica (ICWEA)
506		9.50	1.00	6.95	2.33		212	5.70													Liu Mingqiang et al.,2006	Acta Petrologica et Mineralogica (ICWEA)
658		5.10	0.50	4.78	2.00		112	3.31													Liu Mingqiang et al.,2006	Acta Petrologica et Mineralogica (ICWEA)
401		6.91	0.50	5.74	1.33		142	4.16													Liu Mingqiang et al.,2006	Acta Petrologica et Mineralogica (ICWEA)
374		13.00	1.30	18.00	2.90		214	5.20													Jiang Sihong et al., 2006	Acta Geologica Sinca (ICWEA)
381		11.00	1.10	15.00	2.40		166	3.60													Jiang Sihong et al., 2006	Acta Geologica Sinca (ICWEA)
660		19.00	1.80	33.00	6.30		241	6.10													Jiang Sihong et al., 2006	Acta Geologica Sinca (ICWEA)
397		9.60	0.90	31.00	5.80		173	4.40													Jiang Sihong et al., 2006	Acta Geologica Sinca (ICWEA)
406		18.00	2.20	22.00	3.60		131	3.60													Jiang Sihong et al., 2006	Acta Geologica Sinca (ICWEA)
		30.33																			Jiang Sihong et al., 2006	Acta Geologica Sinca (ICWEA)
		22.56																			Jiang Sihong et al., 2006	Acta Geologica Sinca (ICWEA)
1305	0.8	6.35	0.55	7.20	1.25	10.32	277.9	4.94	3.83	1.83			0.63			20.60			1.48		Zhang Zunzhong et al., 2006	Acta Petrologica Sinica (ICWEA)
666	1.22	7.48	0.61	5.01	1.08	13.48	501.4	7.36	6.69	1.17			1.05			18.95			1.78		Zhang Zunzhong et al., 2006	Acta Petrologica Sinica (ICWEA)
938	1.99	10.95	1.22	12.56	2.47	23.31	1404.1	19.46	21.16	2.67			0.62			21.04			3.53		Zhang Zunzhong et al., 2006	Acta Petrologica Sinica (ICWEA)
829	1.82	18.57	1.16	7.25	1.54	19.29	1264.8	18.45	7.100	1.68			0.51			21.83			1.74		Zhang Zunzhong et al., 2006	Acta Petrologica Sinica (ICWEA)
2013	10.74	18.55	4.26	13.04	5.36	30.87	333.2	7.05	30.69	1.55			1.18			18.80			36.67		Zhang Zunzhong et al., 2006	Acta Petrologica Sinica (ICWEA)
554	2.6	9.10	2.20	16.20			157	4.80													Zhang Zunzhong et al., 2006	Acta Petrologica Sinica (ICWEA)
649	3	9.50	2.00	9.90			151	4.90													Li Wuping et al., 2001	Geological Review (ICWEA)
701	3	9.80	2.00	15.10			160	4.40													Li Wuping et al., 2001	Geological Review (ICWEA)
702		6.11	0.39	6.44	0.52		65.5	1.91													Li Jinyi et al.,2007	Acta Geologica Sinica (ICWEA)
895		10.20	1.50	33.40	1.15		172	5.92													Li Jinyi et al.,2007	Acta Geologica Sinica (ICWEA)
724		11.10	1.12	24.70	1.28		123	4.18													Li Jinyi et al.,2007	Acta Geologica Sinica (ICWEA)
289		12.30	1.38	21.00	1.24		135	4.89													Li Jinyi et al.,2007	Acta Geologica Sinica (ICWEA)
416		10.30	1.60	27.60	1.14		150	5.17													Li Jinyi et al.,2007	Acta Geologica Sinica (ICWEA)
629		13.90	1.61	28.50	1.69		145	5.39													Li Jinyi et al.,2007	Acta Geologica Sinica (ICWEA)
1275		43.00	2.30	13.00	2.50		84.2	2.30													Wang Tao et al.,2004	Journal of Asian Earth Sciences
1122		36.00	3.00	10.00	5.00		581	2.00													Wang Tao et al.,2004	Journal of Asian Earth Sciences
1234		42.00	3.80	11.00	3.20		476	2.60													Wang Tao et al.,2004	Journal of Asian Earth Sciences
246		8.00	0.20	104.00	4.20		105	1.70													Wang Tao et al.,2004	Journal of Asian Earth Sciences
170		24.00	1.80	21.00	2.40		38.0	2.10													Wang Tao et al.,2004	Journal of Asian Earth Sciences
478		1.00	0.20			29.00	38.0		11.00	1.00	2.00	7.00		2.00							Chen Bin at al.,2000	Tectonophysics
664		10.00	1.80			29.00	63.0		21.00	5.00	12.00	21.00		5.00							Chen Bin at al.,2000	Tectonophysics
410		6.30	1.10			26.00	142		17.00	1.00	5.00	6.00		1.00							Chen Bin at al.,2000	Tectonophysics
558		5.50	0.90			27.00	143		15.00	2.00	26.00	8.00		8.00							Chen Bin at al.,2000	Tectonophysics
902		5.40	1.00	15.40	1.48		158	4.20	30.00			10.00				16.60					Guan Qingbin et al., 2020	Gondwana Research
974		7.00	1.20	21.20	1.51		112	3.30	25.00			20.00				15.50					Guan Qingbin et al., 2020	Gondwana Research
896		11.60	1.30	9.58	1.95		156	4.30	46.00			20.00				18.90					Guan Qingbin et al., 2020	Gondwana Research
1345		6.60	0.80	30.30	1.69		224	5.60	44.00			10.00				18.60					Guan Qingbin et al., 2020	Gondwana Research
754		10.60	2.00	14.25	2.06		198	5.50	33.00			20.00				18.30					Guan Qingbin et al., 2020	Gondwana Research
1125		10.80	0.70	13.30	1.08		201	5.70	62.00			10.00				19.40					Guan Qingbin et al., 2020	Gondwana Research
590		7.20	0.40	5.27	1.57		222	5.60	139.00			20.00				23.10					Guan Qingbin et al., 2020	Gondwana Research
554		6.80	0.30	4.77	1.56		214	5.40	131.00			20.00				23.00					Guan Qingbin et al., 2020	Gondwana Research
650		8.00	0.30	2.68	0.77		109	3.20	190.00			10.00				24.30					Guan Qingbin et al., 2020	Gondwana Research
694		8.00	0.30	3.26	0.76		114	3.20	204.00			10.00				24.20					Guan Qingbin et al., 2020	Gondwana Research
742		4.12	0.26	4.96	1.19		66.8	2.01				2.56									Guan Qingbin et al., 2020	Gondwana Research
198		4.14	0.38	4.31	0.84		85.8	2.58				6.12									Guan Qingbin et al., 2020	Gondwana Research
712		5.72	0.50	3.03	0.71		74.5	2.63				4.18									Guan Qingbin et al., 2020	Gondwana Research
823		7.50	0.60	10.00	2.75		232	5.80	34.00			20.00				17.60					Guan Qingbin et al., 2020	Gondwana Research
906		13.50	1.10	11.30	3.66		280	6.80	55.00			10.00				15.30					Guan Qingbin et al., 2020	Gondwana Research
707		8.30	0.80	17.60	3.13		195	5.30	17.00			10.00				15.80					Guan Qingbin et al., 2020	Gondwana Research
754		8.20	0.90	17.65	3.07		209	5.60	13.00			20.00				16.10					Guan Qingbin et al., 2020	Gondwana Research
23.3		22.10	2.00	22.50	7.44		729	19.00	<5			30.00				25.30					Guan Qingbin et al., 2020	Gondwana Research
105		21.40	1.80	21.40	6.47		709	18.20	<5			10.00				25.10					Guan Qingbin et al., 2020	Gondwana Research
30.7		21.60	1.90	22.50	6.25		746	19.00	<5			10.00				25.30					Guan Qingbin et al., 2020	Gondwana Research
28.9		24.40	1.90	22.10	7.65		744	19.10	<5			20.00				25.70					Guan Qingbin et al., 2020	Gondwana Research
644		7.80	0.70	8.16	3.34		316	8.10	11.00			20.00				18.80					Guan Qingbin et al., 2020	Gondwana Research
606		7.80	0.70	8.09	3.31		281	7.20	12.00			20.00				18.90					Guan Qingbin et al., 2020	Gondwana Research
645		7.60	0.70	8.83	3.65		231	6.40	12.00			30.00				18.40					Guan Qingbin et al., 2020	Gondwana Research
255		6.00	0.50	7.39	2.89		134	4.10	20.00			60.00				13.60					Guan Qingbin et al., 2020	Gondwana Research
377	1.11	9.88	0.57	4.04	0.87	7.39	176	4.12	142.0	31.90	114.0	288.0				17.60		22.30			Tang Jie et al., 2016	Gondwana Research
354	1.20	9.65	0.58	4.19	0.92	7.14	176	4.14	185.0	34.40	117.0	276.0				17.60		22.00			Tang Jie et al., 2016	Gondwana Research
406	1.11	9.77	0.57	4.08	0.82	7.27	167	4.11	151.0	32.00	116.0	285.0				17.40		21.80			Tang Jie et al., 2016	Gondwana Research
397	1.19	9.28	0.55	4.25	0.89	7.65	171	4.20	136.0	30.70	110.0	271.0				17.50		20.10			Tang Jie et al., 2016	Gondwana Research
460	2.17	10.40	0.99	18.30	1.99	20.30	140	4.35	5.43	0.75	0.44	0.41				14.90		2.48			Tang Jie et al., 2016	Gondwana Research
411	2.35	11.60	0.94	18.90	6.36	20.30	150	4.84	5.42	0.52	0.18	0.49				15.20		2.52			Tang Jie et al., 2016	Gondwana Research
423	2.14	12.60	0.96	17.80	3.58	20.80	154	4.86	5.43	0.60	0.27	0.55				15.10		2.60			Tang Jie et al., 2016	Gondwana Research
431	2.50	12.90	1.25	22.50	3.27	21.00	149	4.71	5.84	0.72	0.26	0.69				15.80		2.53			Tang Jie et al., 2016	Gondwana Research
398	1.81	12.80	0.62	22.10	2.13	20.50	134	3.96	4.97	0.66	0.40	0.48				15.40		1.95			Tang Jie et al., 2016	Gondwana Research
407	1.75	14.00	0.59	23.20	2.57	20.70	153	4.36	6.24	0.69	0.35	0.45				15.40		2.08			Tang Jie et al., 2016	Gondwana Research
414	1.84	15.60	0.72	21.00	2.33	20.80	149	4.38	5.75	0.87	0.36	0.48				16.30		2.45			Tang Jie et al., 2016	Gondwana Research
1051	3.41	14.20	1.17	15.90	1.59	23.40	167	4.37	10.20	0.89	0.45	1.17				20.80		2.76			Tang Jie et al., 2016	Gondwana Research
887	3.24	14.30	1.13	19.70	1.64	22.90	171	4.79	10.70	0.84	0.22	1.07				21.20		2.83			Tang Jie et al., 2016	Gondwana Research

Ba	Be	Nb	Ta	Th	U	Pb	Zr	Hf	V	Co	Ni	Cr	W	Cu	Zn	Ga	Ge	Sc	Sn	As	Refereces	
317	2.49	5.60	0.38	4.24	1.26	17.30	124	3.30	30.10	3.64	1.24	1.73				16.80		4.12			Tang Jie et al., 2016	Gondwana Research
285	1.65	7.02	0.43	4.55	2.75	14.40	147	4.03	35.70	3.90	1.85	2.57				20.00		5.08			Tang Jie et al., 2016	Gondwana Research
228	1.60	7.13	0.41	5.15	2.85	14.80	151	3.94	33.50	3.68	1.83	2.39				20.20		4.74			Tang Jie et al., 2016	Gondwana Research
57.5	0.22	0.53	0.04	0.18	0.06	1.75	11.2	0.34	101.0	26.60	13.70	65.00				17.70		16.90			Tang Jie et al., 2016	Gondwana Research
66.9	0.22	0.47	0.04	0.21	0.06	1.56	9.72	0.33	125.0	27.80	12.80	81.40				16.90		21.10			Tang Jie et al., 2016	Gondwana Research
57	0.19	0.50	0.04	0.16	0.08	1.63	10.7	0.34	123.0	27.80	12.60	79.30				16.30		20.30			Tang Jie et al., 2016	Gondwana Research
64.3	0.15	0.36	0.02	0.08	0.03	1.36	7.93	0.24	126.0	28.50	13.00	80.00				16.70		20.20			Tang Jie et al., 2016	Gondwana Research
331	1.87	12.20	0.92	5.19	1.80	7.36	115	3.18	335.0	30.90	9.24	17.10				20.20		30.90			Tang Jie et al., 2016	Gondwana Research
312	1.79	11.50	0.83	4.58	1.70	7.34	137	3.62	292.0	28.10	8.53	16.00				19.70		29.20			Tang Jie et al., 2016	Gondwana Research
360	1.73	13.10	1.05	3.80	1.69	6.39	91	2.60	310.0	27.40	8.38	14.90				18.60		29.90			Tang Jie et al., 2016	Gondwana Research
318	1.87	12.30	0.95	4.16	1.80	8.10	112	3.11	304.0	28.40	8.31	15.30				19.20		29.30			Tang Jie et al., 2016	Gondwana Research
478	1.45	10.60	0.59	3.28	0.83	5.94	124	3.76	217.0	30.10	15.00	93.60				21.60		37.80			Tang Jie et al., 2016	Gondwana Research
501	1.55	17.60	0.71	1.68	0.52	6.59	186	4.63	316.0	35.30	5.67	10.20				26.20		33.80			Tang Jie et al., 2016	Gondwana Research
415	1.58	18.10	0.79	2.28	0.57	6.68	241	5.85	338.0	34.40	5.37	9.25				25.60		37.00			Tang Jie et al., 2016	Gondwana Research
459	1.54	17.70	0.78	1.75	0.55	6.81	205	5.14	329.0	33.70	5.11	8.19				25.80		34.50			Tang Jie et al., 2016	Gondwana Research
427	1.21	7.54	0.47	4.39	1.03	7.09	98.1	2.96	157.0	29.90	45.10	90.40				18.90		23.20			Tang Jie et al., 2016	Gondwana Research
359	1.15	7.86	0.52	4.00	1.17	6.81	121	3.51	161.0	30.90	46.80	96.70				18.90		23.40			Tang Jie et al., 2016	Gondwana Research
501	0.93	7.24	0.50	3.24	1.07	7.12	111	3.18	175.0	34.90	57.40	117.00				18.20		24.70			Tang Jie et al., 2016	Gondwana Research
1031	1.95	10.00	0.57	11.60	1.79	18.80	253	6.15	15.00	2.74	0.73	0.97				17.40		5.15			Tang Jie et al., 2016	Gondwana Research
1072	2.16	9.47	0.61	10.50	1.74	22.60	217	5.34	13.80	2.42	0.69	0.74				17.20		5.46			Tang Jie et al., 2016	Gondwana Research
1008	2.00	10.40	0.76	10.90	1.71	24.20	179	4.60	13.50	2.23	0.58	0.90				16.20		5.18			Tang Jie et al., 2016	Gondwana Research
577	2.82	13.60	1.20	13.30	2.30	21.90	163	4.71	17.90	3.30	1.78	4.84				20.20		4.44			Tang Jie et al., 2016	Gondwana Research
629	2.72	12.20	1.06	12.90	1.67	22.30	154	4.33	16.50	2.95	1.75	4.18				19.00		4.04			Tang Jie et al., 2016	Gondwana Research
556	2.86	11.90	0.99	14.30	2.43	22.10	155	4.49	16.60	3.10	1.76	4.21				18.30		3.38			Tang Jie et al., 2016	Gondwana Research
599	2.87	11.50	0.96	10.40	1.93	22.40	142	4.17	16.70	3.01	2.60	4.01				18.40		3.56			Tang Jie et al., 2016	Gondwana Research
881	2.39	10.70	0.84	10.60	1.66	19.20	166	4.39	17.80	3.31	6.08	8.99				18.10		3.69			Tang Jie et al., 2016	Gondwana Research
1243	2.27	10.50	0.76	7.41	1.47	20.60	165	4.31	18.80	3.30	1.56	4.45				18.40		4.08			Tang Jie et al., 2016	Gondwana Research
911	2.70	11.80	0.91	12.30	1.78	20.70	184	4.78	19.50	3.67	1.73	4.77				20.90		4.83			Tang Jie et al., 2016	Gondwana Research
904	2.51	12.00	0.89	10.90	1.79	20.50	181	4.82	19.30	3.58	1.99	4.73				20.50		4.90			Tang Jie et al., 2016	Gondwana Research
616	3.05	11.80	0.92	16.60	5.20	24.10	165	4.66	17.50	3.18	3.08	6.39				18.90		3.32			Tang Jie et al., 2016	Gondwana Research
408	3.02	11.60	0.96	13.30	3.55	23.20	138	3.99	15.60	2.93	1.46	3.64				18.50		3.22			Tang Jie et al., 2016	Gondwana Research
469	2.97	12.70	1.12	18.60	6.17	23.40	144	4.23	15.70	2.78	1.55	3.40				18.10		3.52			Tang Jie et al., 2016	Gondwana Research
647	2.10	9.28	0.50	9.07	0.96	17.30	153	4.11	8.81	1.75	0.89	1.29				16.20		2.52			Tang Jie et al., 2016	Gondwana Research
724	2.04	9.12	0.46	8.62	0.97	18.10	139	3.76	8.28	1.68	1.19	1.80				16.80		2.44			Tang Jie et al., 2016	Gondwana Research
625	2.18	7.56	0.55	8.77	1.47	16.00	150	4.17	8.36	1.64	0.51	1.06				15.60		2.88			Tang Jie et al., 2016	Gondwana Research
692	2.34	6.60	0.44	8.53	1.29	17.10	153	4.18	8.40	1.61	0.78	1.29				16.70		2.90			Tang Jie et al., 2016	Gondwana Research
306	2.86	11.40	0.92	16.50	3.15	16.90	218	5.99	29.10	4.38	2.15	2.23				16.80		4.52			Tang Jie et al., 2016	Gondwana Research
246	2.53	10.00	0.94	14.70	3.26	11.80	193	5.43	22.40	3.54	0.93	1.72				15.60		4.03			Tang Jie et al., 2016	Gondwana Research
948	1.23	3.90	0.16	8.45	1.33	10.80	179	4.48	31.60	3.75	0.50	0.72				16.40		3.20			Tang Jie et al., 2016	Gondwana Research
1105	1.20	5.14	0.25	10.60	1.42	10.20	202	5.09	33.50	3.80	0.45	0.83				17.00		3.49			Tang Jie et al., 2016	Gondwana Research
1011	1.19	5.55	0.26	9.92	1.72	11.50	221	5.54	37.40	4.71	0.52	0.87				17.40		3.95			Tang Jie et al., 2016	Gondwana Research
1076	1.83	5.00	0.49	7.18	3.42	11.90	198	4.90	31.20	3.81	0.50	0.81				17.10		3.55			Tang Jie et al., 2016	Gondwana Research
861	1.80	7.07	0.46	11.00	3.21	10.60	294	7.11	51.70	6.05	1.51	3.03				20.50		5.68			Tang Jie et al., 2016	Gondwana Research
441	1.59	9.23	0.53	5.30	1.70	10.40	93.2	2.58	156.0	20.20	13.80	54.60				20.20		19.60			Tang Jie et al., 2016	Gondwana Research
1966	4.59	25.30	2.79	29.20	3.01	20.00	400	10.30	3.30	0.82	0.18	0.28				22.40		5.44			Tang Jie et al., 2016	Gondwana Research
2085	3.85	16.60	1.43	30.30	2.60	20.90	365	9.42	2.58	0.68	0.59	0.69				19.90		4.46			Tang Jie et al., 2016	Gondwana Research
2121	3.83	21.80	1.71	34.00	2.58	20.10	482	12.40	3.12	0.88	0.45	0.17				21.10		5.46			Tang Jie et al., 2016	Gondwana Research
871	3.19	13.90	1.24	10.60	2.59	22.70	107	3.53	2.81	0.46	0.27	0.33				15.40		2.67			Tang Jie et al., 2016	Gondwana Research
909	3.26	14.50	1.23	12.20	2.16	23.50	110	3.53	3.05	0.44	0.47	0.33				15.80		2.66			Tang Jie et al., 2016	Gondwana Research
907	3.64	13.80	1.13	11.60	2.51	24.20	117	3.71	5.16	1.09	9.43	15.20				16.60		3.18			Tang Jie et al., 2016	Gondwana Research
654	2.94	10.50	0.79	15.40	3.63	15.70	220	6.25	12.30	1.27	0.43	0.64				15.90		5.37			Tang Jie et al., 2016	Gondwana Research
655	3.55	14.20	1.36	18.60	3.42	21.10	220	6.83	12.90	1.42	0.38	0.76				17.80		5.67			Tang Jie et al., 2016	Gondwana Research
656	2.62	12.60	1.06	23.00	4.21	21.30	171	5.30	11.20	1.23	0.44	0.49				17.00		5.06			Tang Jie et al., 2016	Gondwana Research
690	2.95	13.20	1.10	18.80	2.70	21.10	185	5.78	10.40	1.27	0.30	0.52				17.30		4.94			Tang Jie et al., 2016	Gondwana Research
112	2.36	29.40	1.93	25.40	2.22	25.20	104	5.49	0.99	0.17	0.50	0.30				18.80		1.48			Tang Jie et al., 2016	Gondwana Research
115	2.23	28.90	1.98	30.10	2.88	25.80	131	6.49	1.23	0.17	0.45	0.29				18.40		1.64			Tang Jie et al., 2016	Gondwana Research
151	2.22	36.70	2.13	29.40	2.51	25.00	115	5.70	2.27	0.21	0.52	0.27				18.80		1.36			Tang Jie et al., 2016	Gondwana Research
555		7.20	0.70	8.88	3.48		209	5.70	18.00			30.00				17.20					Guan et al., 2020	Gondwana Research
826	2.38	12.20	1.08	11.20	2.30	35.80	167	4.78	17.00	3.22	2.37	5.51				21.10					Li Yu et al., 2017	Journal of Asian Earth Sciences
841	2.35	10.90	1.00	8.33	1.52	35.60	157	4.36	15.70	2.90	2.04	5.19				20.10					Li Yu et al., 2017	Journal of Asian Earth Sciences
676	2.67	7.16	0.69	8.28	1.89	22.30	144	4.20	62.80	7.26	4.52	26.40				17.20					Li Yu et al., 2017	Journal of Asian Earth Sciences
721	2.32	11.00	1.02	7.60	1.46	33.50	165	4.72	16.00	3.02	1.89	5.34				19.90					Li Yu et al., 2017	Journal of Asian Earth Sciences
1035	3.03	13.20	0.91	9.40	3.20	13.50	230	5.98	128.0	17.60	36.70	78.90				21.10					Li Yu et al., 2017	Journal of Asian Earth Sciences
1098	2.25	11.00	0.75	10.30	2.46	13.00	149	4.04	130.0	18.00	38.70	81.60				20.90					Li Yu et al., 2017	Journal of Asian Earth Sciences
1004	2.07	10.50	0.69	10.30	2.46	12.80	129	3.54	129.0	18.20	40.00	84.60				21.00					Li Yu et al., 2017	Journal of Asian Earth Sciences
1157	2.44	10.00	0.74	5.22	2.23	11.60	241	5.82	53.10	7.13	5.23	16.80				25.00					Li Yu et al., 2017	Journal of Asian Earth Sciences
1177	2.38	11.40	0.87	7.24	2.28	11.90	249	6.15	55.30	7.36	5.19	18.10				24.70					Li Yu et al., 2017	Journal of Asian Earth Sciences
1126	2.49	12.00	0.93	6.85	2.51	11.60	256	6.12	60.70	7.94	6.26	19.00				25.50					Li Yu et al., 2017	Journal of Asian Earth Sciences
2726	1.62	16.60	0.96	6.14	2.08	19.80	591	11.10	37.60	7.94	1.06	1.11				18.20					Li Yu et al., 2017	Journal of Asian Earth Sciences
2401	1.63	14.30	0.84	5.88	2.18	18.50	719	13.80	41.80	7.52	1.09	1.16				19.20					Li Yu et al., 2017	Journal of Asian Earth Sciences
1355	2.25	15.40	0.88	13.00	2.05	15.80	655	13.70	33.80	6.24	0.98	0.92				21.30					Li Yu et al., 2017	Journal of Asian Earth Sciences
1944	2.19	13.40	0.77	8.14	1.99	17.20	521	11.00	35.20	6.18	0.88	0.99				21.30					Li Yu et al., 2017	Journal of Asian Earth Sciences
720	13.80	9.26	0.90	10.50	2.41	17.00	76.6	3.20	1.80	0.35	0.42	0.80				18.60					Li Yu et al., 2017	Journal of Asian Earth Sciences
757	3.20	9.88	0.94	9.80	3.34	17.40	74.5	3.21	2.06	0.35	0.48	0.70				18.00					Li Yu et al., 2017	Journal of Asian Earth Sciences
480	2.70	9.61	0.71	16.00	3.45	17.80	128	3.79	33.20	2.68	1.17	2.01				17.30					Li Yu et al., 2017	Journal of Asian Earth Sciences
501	2.18	8.56	0.65	13.00	2.59	17.80	132	3.74	30.90	2.80	1.14	1.30				17.10					Li Yu et al., 2017	Journal of Asian Earth Sciences
679	2.43	9.52	0.71	15.20	3.39	20.60	152	4.53	30.90	3.62	1.12	1.59				16.90					Li Yu et al., 2017	Journal of Asian Earth Sciences
1065		7.90	0.57	8.36	1.59		202	5.11	36.50	5.15	2.41	6.11				21.10					Tang Jie et al., 2014	Lithos
1192		9.23	0.67	11.60	1.62		179	4.55	37.10	5.32	2.47	5.97				20.90					Tang Jie et al., 2014	Lithos
1256		6.97	0.51	7.03	1.58		158	4.03	32.20	4.72	2.19	5.10				20.40					Tang Jie et al., 2014	Lithos
1271		8.79	0.62	11.40	1.61		187	4.70	33.50	4.81	2.54	5.67				20.80					Tang Jie et al., 2014	Lithos
814		10.70	1.17	8.56	3.37		195	5.24	64.50	4.42	8.72	33.50				22.00					Tang Jie et al., 2014	Lithos
738		6.02	0.47	6.58	2.08		186	5.00	71.50	10.20	11.00	43.60				22.00					Tang Jie et al., 2014	Lithos
870		8.74	0.63	8.44	3.06		168	4.65	70.00	9.47	10.80	42.70				22.30					Tang Jie et al., 2014	Lithos
741		6.76	0.55	6.55	2.48		160	4.36	71.20	10.10	11.10	41.70				22.70					Tang Jie et al., 2014	Lithos
786		9.04	0.79	7.66	2.95		201	5.38	79.10	10.90	12.30	47.60				22.80					Tang Jie et al., 2014	Lithos
484		14.70	0.90	10.70	4.53		205	5.15	181.0	26.10	42.90	181.0				21.80					Tang Jie et al., 2014	Lithos
1098		7.58	0.47	3.43	0.90		184	4.41	77.10	9.80	8.58	37.70				21.30					Tang Jie et al., 2014	Lithos
734		11.30	0.82	7.87	2.91		250	5.90	126.0	16.80	15.00	52.70				21.50					Tang Jie et al., 2014	Lithos
1103		9.98	0.70	4.41	1.03		188	4.68	79.40	10.10	9.18	38.40				21.60					Tang Jie et al., 2014	Lithos
730		6.51	0.42	9.59	1.10		195	4.77	10.60	1.55	0.54	0.70				14.40					Tang Jie et al., 2014	Lithos
709		7.65	0.49	13.40	1.25		200	4.95	11.90	1.64	0.48	0.67				15.10					Tang Jie et al., 2014	Lithos
690		6.33	0.48	9.86	1.60		149	3.98	9.12	1.41	0.45	0.71				14.20					Tang Jie et al., 2014	Lithos
704		6.15	0.42	7.09	1.25		151	3.83	8.81	1.56	0.44	0.44				13.70					Tang Jie et al., 2014	Lithos
927		13.20	0.87	15.30	2.78		163	4.58	11.60	1.85	1.25	1.89				20.20					Tang Jie et al., 2014	Lithos
655		14.20	1.24	21.00	3.90		174	4.92	12.40	1.74	0.63	1.72				20.10					Tang Jie et al., 2014	Lithos
1049		13.70	1.05	16.10	2.26		158	4.59	11.70	1.57	1.29	2.03				19.40					Tang Jie et al., 2014	Lithos
853		14.80	1.09	24.40	4.93		179	5.22	11.70	1.64	1.03	3.11				18.10					Tang Jie et al., 2014	Lithos
84.7	2.34	26.00	2.14	29.40	1.86	34.40	115	5.70	1.60	0.16	0.28	0.32				19.10		1.79			Tang Jie et al., 2016	Gondwana Research
621	1.76	5.02	0.41	3.40	0.89	11.40	86	2.30													Jahn et al., 2004	Journal of Asian Earth Sciences

Ba	Be	Nb	Ta	Th	U	Pb	Zr	Hf	V	Co	Ni	Cr	W	Cu	Zn	Ga	Ge	Sc	Sn	As	Refereces	
1022	2.96	7.39	0.93	16.32	2.77	22.60	155	4.70													Jahn et al., 2004	Journal of Asian Earth Sciences
612.8	4.46	9.93	1.64	34.31	9.57	40.00	151	4.80													Jahn et al., 2004	Journal of Asian Earth Sciences
813.3	9.67	9.68	1.12	19.56	3.75	23.40	192	5.10													Jahn et al., 2004	Journal of Asian Earth Sciences
614.3	4.34	8.87	1.42	30.56	5.70	35.20	126	3.80													Jahn et al., 2004	Journal of Asian Earth Sciences
1314	4.58	8.21	1.08	11.28	3.58	23.10	164	4.70													Jahn et al., 2004	Journal of Asian Earth Sciences
168.7	4.56	16.99	1.81	17.21	5.32	76.80	60	2.80													Jahn et al., 2004	Journal of Asian Earth Sciences
619.7	2.68	10.28	0.92	10.43	2.53	20.90	293	7.60													Jahn et al., 2004	Journal of Asian Earth Sciences
416.4	3.39	3.72	0.35	69.29	4.65	43.20	140	4.40													Jahn et al., 2004	Journal of Asian Earth Sciences
630.8	4.63	16.92	2.69	54.21	4.55	39.60	269	7.70													Jahn et al., 2004	Journal of Asian Earth Sciences
822.6	1.31	7.84	0.61	9.96	1.24	21.90	184	5.10													Jahn et al., 2004	Journal of Asian Earth Sciences
767		5.00	1.00	12.00	2.00		140	5.00													Kovalenko et al.,2003	Petrology
440		9.00	1.00	8.00	1.00	10.00	52	2.00													Kovalenko et al.,2003	Petrology
764	2.00	9.00	1.00	8.00	1.00	16.00	48	1.00													Kovalenko et al.,2003	Petrology
122		11.00	1.00	57.00	7.00		270	10.00													Kovalenko et al.,2003	Petrology
335		11.00	2.00	23.00	3.00	17.00	127	5.00													Kovalenko et al.,2003	Petrology
183	7.00	16.00	2.00	28.00	3.00	24.00	126	5.00													Kovalenko et al.,2003	Petrology
602	3.00	6.00	1.00	14.00	3.00	14.00	98	3.00													Kovalenko et al.,2003	Petrology
517	4.00	6.00	1.00	10.00	5.00	1.00	89	3.00													Kovalenko et al.,2003	Petrology
385	3.00	7.00	1.00	19.00	3.00	3.00	84	3.00													Kovalenko et al.,2003	Petrology
680	3.00	4.00	0.00	18.00	2.00	17.00	115	4.00													Kovalenko et al.,2003	Petrology
460	2.00	6.00	1.00	14.00	5.00	15.00	56	2.00													Kovalenko et al.,2003	Petrology
412	4.00	7.00	1.00	25.00	7.00	17.00	75	3.00													Kovalenko et al.,2003	Petrology
585	3.00	7.00	1.00	15.00	3.00	10.00	78	3.00													Kovalenko et al.,2003	Petrology
103		3.00	1.00	3.00	1.00		133	3.00													Kovalenko et al.,2003	Petrology
419		14.00	2.00	23.00	4.00		212	6.00													Kovalenko et al.,2003	Petrology
133		9.00	4.00	27.00	8.00		135	5.00													Kovalenko et al.,2003	Petrology
101		23.00	3.00	51.00	9.00		151	7.00													Kovalenko et al.,2003	Petrology
101		34.00	16.00	17.00	16.00		151	9.00													Kovalenko et al.,2003	Petrology
102		221.00	23.00	14.00	5.00		12	10.00													Kovalenko et al.,2003	Petrology
1246		7.40	0.61	6.37	1.24	10.60	160	4.52	150.0	16.50	7.00	13.00		20.37	67.9	31.68		13.06			Li et al., 2020	Geological Journal
721		7.60	0.61	6.23	1.22	10.20	160	4.46	158.0	14.91	9.00	21.00		19.65	73.8	22.72		15.62			Li et al., 2020	Geological Journal
1110		7.80	0.62	5.35	1.15	9.60	161	4.51	163.0	17.10	6.00	17.00		18.1	63.5	39.29		13.08			Li et al., 2020	Geological Journal
791		7.80	0.61	5.61	1.40	12.90	166	4.76	166.0	17.78	5.00	13.00		24.53	68	30.46		11.44			Li et al., 2020	Geological Journal
983		7.40	0.51	6.04	1.21	11.60	152	4.27	142.0	16.64	5.00	12.00		18.24	62.2	35.48		19.73			Li et al., 2020	Geological Journal
1208		7.60	0.57	5.07	1.30	10.10	158	4.37	158.0	17.36	7.00	18.00		20.71	60.8	41.3		12.43			Li et al., 2020	Geological Journal
868		11.50	0.62	4.90	0.97	8.30	162	4.69	153.0	21.22	8.00	24.00		22.21	74.5	25.57		16.54			Li et al., 2020	Geological Journal
713.63	1.56	3.50	0.39	4.76	1.14	6.89	97.4	5.10	103.1	15.81	11.56	1.33		27.05	58.91	18.21		9.29			Zhao et al., 2021	Lithos
224.43	1.65	3.02	0.05	1.58	0.59	0.97	60.99	1.26	68.01	17.01	42.49	4.26		68.38	37.61	17.95		6.74			Zhao et al., 2021	Lithos
199.04	1.59	3.38	0.05	1.58	0.45	1.14	93.12	1.26	131.0	18.82	38.56	2.54		70.98	44.7	18.36		11.84			Zhao et al., 2021	Lithos
249.38	1.33	3.78	0.27	2.30	1.15	3.73	98.99	2.91	166.1	25.86	84.92	2.11		87.94	56.39	17.81		13.47			Zhao et al., 2021	Lithos
635.6	1.18	3.69	0.25	5.22	1.15	10.14	87.04	3.89	156.4	25.45	64.04	3.85		90.88	53.51	17.54		14.88			Zhao et al., 2021	Lithos
619.22		3.26	0.24	4.74	1.10	10.48	86.02	3.66	146.2	23.91	58.72	17.49		78.71	45.85	18.21		13.38			Zhao et al., 2021	Lithos
591.73		5.58	0.69	9.72	1.97	7.23	180.52	5.95	17.18	2.38	36.15	10.02		14	54.23	17.49		13.64			Zhao et al., 2021	Lithos
2275		5.03	0.20	4.59	1.20	6.93	217.38	4.62	16.31	1.95	24.65	1.52			53.59	17.39		1.49			Zhao et al., 2021	Lithos
495.1	2.00	5.74	0.21	8.15	1.41	7.50	230.73	3.97	15.90	2.06	10.40	3.46		10.38	52.89	17.66		2.21			Zhao et al., 2021	Lithos
605.68		9.00	0.36	5.55	1.20	6.30	69.51	4.33	9.83	1.58	57.31	18.29			22.56	18.72		2.11			Zhao et al., 2021	Lithos
303.27	2.07	9.56	0.03	1.68	0.44	2.56	76.38	1.21	5.13	0.63	10.92	1.39		11.2	18.05	16.36		5.88			Zhao et al., 2021	Lithos
1426.2		21.61	0.89	7.50	1.10	20.94	482.7	11.75	128.5	13.90	11.80	28.00		22.6	111.8						Thomas C. Sheldrick et al., 2020	Gondwana Research
1446.1		20.21	0.85	6.77	1.11	21.50	498.6	11.06	135.6	18.10	13.90	32.60		27.8	116.8						Thomas C. Sheldrick et al., 2020	Gondwana Research
1400.5		23.95	0.93	6.85	1.09	20.27	470.6	10.63	124.7	16.30	14.80	33.90		23.5	111.5						Thomas C. Sheldrick et al., 2020	Gondwana Research
1450.3		20.78	0.87	7.18	1.15	19.92	475.6	11.08	127.1	19.80	15.70	35.80		24	112.6						Thomas C. Sheldrick et al., 2020	Gondwana Research
1442.2		24.90	0.91	5.02	0.88	16.75	512.2	13.07	117.1	21.90	27.40	51.60		27.3	122.7						Thomas C. Sheldrick et al., 2020	Gondwana Research
1452.3		23.52	0.95	4.95	0.90	16.21	512.3	11.27	114.6	23.90	21.10	43.70		25.2	122.7						Thomas C. Sheldrick et al., 2020	Gondwana Research
1540.4		28.38	1.12	5.00	0.87	18.80	524.8	11.61	137.2	21.00	24.40	37.80		21.5	106.3						Thomas C. Sheldrick et al., 2020	Gondwana Research
1150.7		10.20	0.53	10.91	1.94	21.59	280.2	6.77	82.70	12.50	10.10	20.20		18.8	62.6						Thomas C. Sheldrick et al., 2020	Gondwana Research
1262.6		10.38	0.56	11.43	2.03	21.83	297.5	6.96	87.40	10.00	9.90	20.00		20.2	64.1						Thomas C. Sheldrick et al., 2020	Gondwana Research
1189.5		9.87	0.57	10.97	1.96	20.90	277.3	6.88	86.00	12.90	11.80	22.80		20.9	65.1						Thomas C. Sheldrick et al., 2020	Gondwana Research
1209.7		10.41	0.57	11.16	2.00	21.10	277.2	6.69	86.10	12.40	9.70	23.50		19.8	64.2						Thomas C. Sheldrick et al., 2020	Gondwana Research
1325.5		37.11	1.65	6.59	1.58	24.58	729.9	13.77	152.9	23.60	14.80	25.20		42.6	136.8						Thomas C. Sheldrick et al., 2020	Gondwana Research
1208.5		32.17	1.49	5.83	1.77	19.98	585.6	11.18	152.0	19.90	14.80	33.20		41.1	132.2						Thomas C. Sheldrick et al., 2020	Gondwana Research
1417.3		38.44	1.73	4.07	1.08	21.70	631.6	12.40	189.8	29.60	18.40	29.70		37.2	183.8						Thomas C. Sheldrick et al., 2020	Gondwana Research
2151.6		41.84	2.25	12.08	3.61	31.50	855	13.84	30.30	3.10	BDL	BDL		7.5	111.6						Thomas C. Sheldrick et al., 2020	Gondwana Research
1173.3		34.09	1.48	5.70	2.09	22.54	645.3	12.23	165.1	26.60	28.90	47.10		37.8	152.4						Thomas C. Sheldrick et al., 2020	Gondwana Research
1443.5		39.67	1.77	3.94	0.92	19.92	682.1	10.14	165.4	22.40	16.70	26.70		20.9	181.7						Thomas C. Sheldrick et al., 2020	Gondwana Research
1254		44.56	2.01	4.87	1.31	23.49	741.8	13.54	198.1	29.60	17.10	29.60		40.6	185						Thomas C. Sheldrick et al., 2020	Gondwana Research
1315		44.74	1.90	4.71	1.34	23.48	747.3	14.41	193.6	26.70	17.90	27.60		42.2	190.6						Thomas C. Sheldrick et al., 2020	Gondwana Research
1272.2		40.27	1.71	6.42	1.69	22.47	718.3	15.57	189.9	26.00	17.00	46.60		41.7	183.8						Thomas C. Sheldrick et al., 2020	Gondwana Research
1529.6		34.06	1.42	6.01	1.78	24.80	780.9	15.20	171.8	21.80	15.60	28.80		32.4	142.8						Thomas C. Sheldrick et al., 2020	Gondwana Research
181		15	5	32	7	35	201	5	11	3	3	3	5	10	54	25		2	3	3	Sodnom Khishigsuren et al., 2012	Bull. Nagoya Univ. Museum
213		23	5	28	5	30	229	10	13	4	3	3	6	10	60	24		3	10	2	Sodnom Khishigsuren et al., 2012	Bull. Nagoya Univ. Museum
201		32	7	48	12	36	217	8	5	3	3	4	10	15	47	33		2	15	13	Sodnom Khishigsuren et al., 2012	Bull. Nagoya Univ. Museum
354		17	5	49	7	27	355	9	10	3	3	3	5	10	51	25		4	7	19	Sodnom Khishigsuren et al., 2012	Bull. Nagoya Univ. Museum
189		24	5	47	6	46	264	8	5	4	3	3	7	10	32	19		2	4	5	Sodnom Khishigsuren et al., 2012	Bull. Nagoya Univ. Museum
193		25	5	36	17	42	250	7	12	3	3	3	15	10	43	30		2	24	15	Sodnom Khishigsuren et al., 2012	Bull. Nagoya Univ. Museum
40		27	5	56	12	60	176	5	5	3	3	3	5	10	45	28		2	4	2	Sodnom Khishigsuren et al., 2012	Bull. Nagoya Univ. Museum
199		19	5	40	8	25	221	9	5	3	3	3	6	10	47	21		2	47	4	Sodnom Khishigsuren et al., 2012	Bull. Nagoya Univ. Museum
157		18	5	36	7	30	217	10	5	3	3	3	7	10	35	23		3	5	2	Sodnom Khishigsuren et al., 2012	Bull. Nagoya Univ. Museum
253		21	5	47	11	30	243	6	10	3	4	3	16	10	35	25		2	9	4	Sodnom Khishigsuren et al., 2012	Bull. Nagoya Univ. Museum
484		15		19	15	26	106									25					Sodnom Khishigsuren et al., 2012	Bull. Nagoya Univ. Museum
388		18		35		59	178									25					Sodnom Khishigsuren et al., 2012	Bull. Nagoya Univ. Museum
32		44		66		21	224									31					Sodnom Khishigsuren et al., 2012	Bull. Nagoya Univ. Museum
65		44	7	57	12	32	237	10	5	3	2	4	16	5	81	33		1	5	2	Sodnom Khishigsuren et al., 2012	Bull. Nagoya Univ. Museum
117		32	7	47	3	30	161	10	5	3	3	4	6	6	64	29		1	14	19	Sodnom Khishigsuren et al., 2012	Bull. Nagoya Univ. Museum
202		15	7	41	11	25	201	5	12	3	3	3	5	10	54	25		2	8	6	Sodnom Khishigsuren et al., 2012	Bull. Nagoya Univ. Museum
238		14	5	43	6	35	181	7	8	3	3	5	5	10	39	19		2	7	6	Sodnom Khishigsuren et al., 2012	Bull. Nagoya Univ. Museum
124		10	5	47	3	34	206	8	5	4	3	3	5	10	27	25		2	6	2	Sodnom Khishigsuren et al., 2012	Bull. Nagoya Univ. Museum
117		27	5	51	8	22	193	6	5	3	3	3	16	10	81	28		2	23	7	Sodnom Khishigsuren et al., 2012	Bull. Nagoya Univ. Museum
88		24	5	45	8	32	195	8	5	8	3	3	11	10	57	28		2	10	5	Sodnom Khishigsuren et al., 2012	Bull. Nagoya Univ. Museum
17		27	5	49	6	26	212	8	6	3	3	3	8	10	40	28		2	20	10	Sodnom Khishigsuren et al., 2012	Bull. Nagoya Univ. Museum
5		47	5	45	3	23	195	8	5	3	4	3	10	10	67	34		2	23	2	Sodnom Khishigsuren et al., 2012	Bull. Nagoya Univ. Museum
58		26	5	54	13	28	155	6	5	3	3	11	5	10	26	24		2	13	8	Sodnom Khishigsuren et al., 2012	Bull. Nagoya Univ. Museum
320		19	5	28	7	34	253	9	5	3	3	3	17	10	34	22		2	3	2	Sodnom Khishigsuren et al., 2012	Bull. Nagoya Univ. Museum
42		16	5	45	6	45	133	5	5	3	3	3	5	10	28	26		2	3	2	Sodnom Khishigsuren et al., 2012	Bull. Nagoya Univ. Museum
17		17	5	38	4	29	78	5	5	3	3	3	5	10	22	23		2	2	2	Sodnom Khishigsuren et al., 2012	Bull. Nagoya Univ. Museum
126		43.9	3	29.9	8.3	44.9	863.8	20.9	<8	0.4	<20					27.4		2			Zhu Mingshuai et al., 2016	Journal of Asian Earth Sciences
118		40.2	3.3	30.8	7.2	10.1	769.2	20.6	<8	<0.2	<20					28.7		1			Zhu Mingshuai et al., 2016	Journal of Asian Earth Sciences
136		42	2.9	30.6	6.1	43	861.7	22.5	<8	0.3	<20					26.5		2			Zhu Mingshuai et al., 2016	Journal of Asian Earth Sciences
117		44.6	3.5	32.9	8.1	46.5	853.4	23.2	<8	1.4	<20					27.6		2			Zhu Mingshuai et al., 2016	Journal of Asian Earth Sciences
91		49.2	4	46.9	7	29.6	755.9	22.5	<8	1.3	<20					28.6		1			Zhu Mingshuai et al., 2016	Journal of Asian Earth Sciences
550		23.3	1.3	8.6	4.2	11.6	360.6	9.5	<8	1.2	<20					24.7		6			Zhu Mingshuai et al., 2016	Journal of Asian Earth Sciences

Ba	Be	Nb	Ta	Th	U	Pb	Zr	Hf	V	Co	Ni	Cr	W	Cu	Zn	Ga	Ge	Sc	Sn	As	Refereces	
195		39	3.1	22.2	5.8	16.1	1164	27.2	<8	0.3	<20					26.7		5			Zhu Mingshuai et al., 2016	Journal of Asian Earth Sciences
313		29.9	1.5	9.2	2.3	22.9	527.7	11.2	<8	0.6	<20					26		6			Zhu Mingshuai et al., 2016	Journal of Asian Earth Sciences
1148.95		6.5696		15.015	2.1187		347.97	8.2855													Donskaya et al., 2012	Russian Geology and Geophysics
1618.75		7.3496		12.411	1.3874		242.36	5.8963													Donskaya et al., 2012	Russian Geology and Geophysics
794.45		10.46		15.956	1.358		115.42	3.4197													Donskaya et al., 2012	Russian Geology and Geophysics
1345.06		9.0781		16.351	2.3365		230.69	4.8578													Donskaya et al., 2012	Russian Geology and Geophysics
1111.16		11.287		15.123	2.2582		255.47	5.6786													Donskaya et al., 2012	Russian Geology and Geophysics
997.96		9.9814		18.342	2.3595		185.92	3.8293													Donskaya et al., 2012	Russian Geology and Geophysics
820.55		8.6533		25.164	4.5727		124.45	3.9376													Donskaya et al., 2012	Russian Geology and Geophysics
																					Koval, 1998	Siberian branch RAS, Novosibirsk (in Russian)
																					Koval, 1998	Siberian branch RAS, Novosibirsk (in Russian)
																					Koval, 1998	Siberian branch RAS, Novosibirsk (in Russian)
																					Koval, 1998	Siberian branch RAS, Novosibirsk (in Russian)
																					Koval, 1998	Siberian branch RAS, Novosibirsk (in Russian)
																					Koval, 1998	Siberian branch RAS, Novosibirsk (in Russian)
																					Koval, 1998	Siberian branch RAS, Novosibirsk (in Russian)
																					Koval, 1998	Siberian branch RAS, Novosibirsk (in Russian)
																					Koval, 1998	Siberian branch RAS, Novosibirsk (in Russian)
																					Koval, 1998	Siberian branch RAS, Novosibirsk (in Russian)
																					Koval, 1998	Siberian branch RAS, Novosibirsk (in Russian)
																					Koval, 1998	Siberian branch RAS, Novosibirsk (in Russian)
610		61		140			1400														Vorontsov et al., 2007	Petrology
131		38		9			458														Yarmolyuk et al. 2001	Petrology
96		22		8			570														Yarmolyuk et al. 2001	Petrology
39		22	2.06				161														Yarmolyuk et al. 2001	Petrology
93		36	2.42				511														Yarmolyuk et al. 2001	Petrology
22		32	8.5				282														Yarmolyuk et al. 2001	Petrology
147		26		24			626														Yarmolyuk et al. 2001	Petrology
42		19	2.89				336														Yarmolyuk et al. 2001	Petrology
65		18	0.98				228														Yarmolyuk et al. 2001	Petrology
32		40	3.97				777														Yarmolyuk et al. 2001	Petrology
17		63	3.01				1751														Yarmolyuk et al. 2001	Petrology
148		43	4.09				114														Yarmolyuk et al. 2001	Petrology
62		42		21			1024	14													Yarmolyuk et al. 2001	Petrology
110		55		17			1450	50													Yarmolyuk et al. 2001	Petrology
170		64					1500														Yarmolyuk et al. 2001	Petrology
93		75	4.5	21			1108	29.2													Yarmolyuk et al. 2001	Petrology
44		41	3.56	14.5			733	20.6													Yarmolyuk et al. 2001	Petrology
60		42	2.38	13.8			916	20.7													Yarmolyuk et al. 2001	Petrology
65		35	2.1	11.8			652	16.7													Yarmolyuk et al. 2001	Petrology
259		31	1.77	5.5			981	17.6													Yarmolyuk et al. 2001	Petrology
57		20	0.82	2.4			284	6.2													Yarmolyuk et al. 2001	Petrology
62		42		21			1024	14													Jahn et al., 2009	Lithos
56		54	3.29	14.99	3.78		990	23.4													Jahn et al., 2009	Lithos
46		40	3.56	15			910	20.6													Jahn et al., 2009	Lithos
44		37	2.38	14			820	20.7													Jahn et al., 2009	Lithos
53		32	2.1	17			725	16.7													Jahn et al., 2009	Lithos
225		26	1.77	11			990	17.6													Jahn et al., 2009	Lithos
27		16	0.72	2.19	0.83		290	5.7													Jahn et al., 2009	Lithos
540		25		19	4		520														Jahn et al., 2009	Lithos
65		18.3	0.98				228														Jahn et al., 2009	Lithos
120		46	1.34				503														Jahn et al., 2009	Lithos
810		12					224														Reichow et al., 2010	Chemical Geology
81		22					570														Reichow et al., 2010	Chemical Geology
63.18		77.5	4.4	29.6	5.44		1102	29.28													Lykhin et al., 2004	Geology of Ore Deposits
120.9		97.85	6.02	32.98	8.41		714	20.12													Lykhin et al., 2004	Geology of Ore Deposits
149.4		74.02	4.25	19.75	4.35		562	15.64													Lykhin et al., 2004	Geology of Ore Deposits
51.59		75.55	6.02	22.76	2.94		681	15.94													Lykhin et al., 2004	Geology of Ore Deposits
1515		6.6	0.4	4.6	0.91		216	4.8			20	20				20					Ariuntsetseg G et al.,2021	Lithos
1431		3.6	0.38	7.19	1.26	19	154	3.9		4	20	20			50	20		3			Ariuntsetseg G et al.,2021	Lithos
1029		6.6	0.59	13.4	2.39	19	263	6.8		9	20	20			80	18		8			Ariuntsetseg G et al.,2021	Lithos
110		12.448	0.911	22.374	4.689	37.151	322.47	9.56		0.884	7.157	10.149			65.578	20.142		2.299			Ariuntsetseg G et al.,2021	Lithos
201		12.681	0.985	24.333	4.326	34.777	362.29	10.631		0.884	4.806	2.955			88.394	23.302		3.466			Ariuntsetseg G et al.,2021	Lithos
206		12.902	0.926	22.266	4.492	53.427	386.6	11.535		0.753	7.2	8.673			80.095	21.257		1.646			Ariuntsetseg G et al.,2021	Lithos
561		7.7	0.75	14.8	4.04	16	345	10.5		1	20	50			60	22		2			Ariuntsetseg G et al.,2021	Lithos
607		7	0.67	10	3.15	14	292	8.4		2	20	20			80	20		13			Ariuntsetseg G et al.,2021	Lithos
543		22.993	1.29	15.29	4.09	13.3	210.9	7.31		1.606	0.93	5.25			45.4	22.18		3.78			Ariuntsetseg G et al.,2021	Lithos
643		16.993	1.768	16.327	4.909	13.835	300.7	8.923		1.706	0.936	5.525			45.544	22.218		4.178			Ariuntsetseg G et al.,2021	Lithos
541		19.446	1.597	11.924	2.595	11.157	188.02	5.613		1.136	1.528	3.472			45.296	22.221		3.865			Ariuntsetseg G et al.,2021	Lithos
549		25.446	1.79	13.54	3.495	12.2	283.03	7.13		1.14	1.42	3.72			45.29	22.19		3.99			Ariuntsetseg G et al.,2021	Lithos
346		11.1		17.39	3.335		151	7.13		0.09	4.794	50.19			64	27.91		2.602			Ariuntsetseg G et al.,2021	Lithos
318		6.3	0.75	15.5	3.53	16	289	9.7		1	20	20			40	20		2			Ariuntsetseg G et al.,2021	Lithos
351		9.8	0.72	10.2	2.3		327	8.1			20	20				20					Ariuntsetseg G et al.,2021	Lithos
350		6.1	0.73	16	3.64	17	314	9.5		1	20	20			60	20		2			Ariuntsetseg G et al.,2021	Lithos
1344		4.9	0.35	4.26	1.26	9	241	5.7		1	20	20			30	24		13			Ariuntsetseg G et al.,2021	Lithos
1421		4.6	0.35	4.34	1.25	9	250	5.3		2	20	20			30	24		14			Ariuntsetseg G et al.,2021	Lithos
70		14		18.2	3.31		116	6.99		0	4	8			6	28.2		2.97			Ariuntsetseg G et al.,2021	Lithos
729		10	0.59	6.4	1.48		411	7.9													Ariuntsetseg G et al.,2021	Lithos
948.6		5.181	0.393	15.394	2.737	39.949	124.21	3.225		2.684	11.659	38.478			14.992	19.148		2.651			Ariuntsetseg G et al.,2021	Lithos
790.21		6.676	0.545	56.707	2.345	32.425	128.7	3.366		5.709	20.608	48.362			31.257	14.64		4.079			Ariuntsetseg G et al.,2021	Lithos
815		10.282	0.917	36.396	7.122	36.73	207.17	5.583		8.208	31.986	70.215			43.349	19.679		6.186			Ariuntsetseg G et al.,2021	Lithos
973.96		5.804	0.498	24.913	1.952	38.473	121.88	3.462		3.397	21.186	21.612			30.222	19.596		3.161			Ariuntsetseg G et al.,2021	Lithos
803.7		6.926	0.762	22.574	2.45	34.735	170.33	4.423		12.312	56.529	122.19			47.072	18.859		9.107			Ariuntsetseg G et al.,2021	Lithos
906.28		6.205	0.496	23.272	1.873	32.25	173.21	4.616		13.095	60.653	144.28			53.758	18.469		9.62			Ariuntsetseg G et al.,2021	Lithos
1392		5.042	0.432	21.964	1.775	43.198	119.91	3.296		3.083	7.029	20.683			25.095	19.839		2.807			Ariuntsetseg G et al.,2021	Lithos
852		6.781	0.34	19.377	2.148	30.543	217.31	5.416		14.355	61.149	145.27			57.639	21.027		10.404			Ariuntsetseg G et al.,2021	Lithos
805.27		6.088	0.377	12.518	1.542	20.966	189.43	4.656		14.385	46.744	107.38			58.438	19.494		9.618			Ariuntsetseg G et al.,2021	Lithos
652		9.144	1.268	17.216	2.476	26.132	209.49	5.683		6.331	15.541	45.737			48.337	20.045		5.103			Ariuntsetseg G et al.,2021	Lithos
1025		7.761	0.597	21.193	1.825	30.761	152.26	4.197		1.136	4.221	5.712			39.576	18.762		2.369			Ariuntsetseg G et al.,2021	Lithos
1112.8		5.949	0.502	18.283	2.333	17.188	159.53	4.244		3.831	10.287	24.531			20.174	19.168		4.2			Ariuntsetseg G et al.,2021	Lithos
810.1		7.81	0.902	19.754	2.995	24.055	213.21	5.87		8.009	21.012	58.916			46.461	20.078		5.989			Ariuntsetseg G et al.,2021	Lithos
806.72		6.913	0.549	16.181	1.328	22.257	169.71	4.595		13.845	60.658	123.33			57.458	19.435		10.359			Ariuntsetseg G et al.,2021	Lithos
1059.5		4.307	0.201	7.102	1.611	21.548	168.71	4.397		8.021	22.56	63.074			45.65	20.384		5.785			Ariuntsetseg G et al.,2021	Lithos
831.05		5.443	0.434	16.284	2.427	23.922	108.07	3.089		5.793	15.84	42.491			33.654	19.567		4.371			Ariuntsetseg G et al.,2021	Lithos
1148.6		6.08	0.52	18.885	2.358	17.796	162.63	4.305		3.949	17.387	25.507				20.125		4.249			Ariuntsetseg G et al.,2021	Lithos
803.7		6.926	0.762	22.574	2.45	34.735	170.33	4.423		12.312	56.529	122.19				18.859		9.107			Ariuntsetseg G et al.,2021	Lithos
999		0.8	0.2	9.14	0.82	26	71	2.1		1	20	20			30	13		1			Ariuntsetseg G et al.,2021	Lithos
906.28		6.205	0.496	23.272	1.873	32.25	173.21	4.616		13.095	60.653	144.28				18.469		9.62			Ariuntsetseg G et al.,2021	Lithos
455		8.1	1.31	15.6	1.87		189	4.7													Ariuntsetseg G et al.,2021	Lithos
447		8.642		15.76	4.437		78.67	3.398		1.751	10.38	26.16			22	22.01		5.081			Ariuntsetseg G et al.,2021	Lithos

Ba	Be	Nb	Ta	Th	U	Pb	Zr	Hf	V	Co	Ni	Cr	W	Cu	Zn	Ga	Ge	Sc	Sn	As	Refereces	
642		13					551			18	84	236			67						Ariuntsetseg G et al.,2021	Lithos
702		6.2	0.6	10	3.09	15	321	7.6		5	20	20			90	20		15			Ariuntsetseg G et al.,2021	Lithos
680		7					140			6	18	140			37						Ariuntsetseg G et al.,2021	Lithos
613		12					301			11	80	230			41						Ariuntsetseg G et al.,2021	Lithos
696		6					101			2	3	4			20						Ariuntsetseg G et al.,2021	Lithos
694		14	1.24	24	1.74		261	5.8													Ariuntsetseg G et al.,2021	Lithos
459		5.7	0.67	13.1	1.51	16	97	3		2	20	20			30	16		4			Ariuntsetseg G et al.,2021	Lithos
677		3.5	0.87	15.1	5.36	29	70	2.5		1	20	20			30	15		3			Ariuntsetseg G et al.,2021	Lithos
643		8.1	0.84	31.9	7.79	17	237	5.9		11	70	220			40	20		10			Ariuntsetseg G et al.,2021	Lithos
768		9.5	1.03	30.4	3.06	31	343	8.4		17	80	230			60	19		11			Ariuntsetseg G et al.,2021	Lithos
806		6.913	0.549	16.181	1.328	22.257	169.71	4.595		13.845	60.658	123.33				19.435		10.359			Ariuntsetseg G et al.,2021	Lithos
785		5.949	0.473	19.977	1.874	22.306	138.51	3.902		4.103	11.635	21.44			65.58	19.161		3.729			Ariuntsetseg G et al.,2021	Lithos
1024		7.761	0.597	21.193	1.825	30.761	152.26	4.197		1.136	4.221	5.712				18.762		2.369			Ariuntsetseg G et al.,2021	Lithos
1022		7.2	0.59	23.4	1.56	32.7	159.6	4.45		1.32	4.16	5.45				18.87		2.41			Ariuntsetseg G et al.,2021	Lithos
1211		5	0.67	20.7	5.54	23	170	4.2		9	30	70			50	18		8			Ariuntsetseg G et al.,2021	Lithos
813		7.2	0.53	17.1	3.84	16	206	6.3		18	70	120			70	20		14			Ariuntsetseg G et al.,2021	Lithos
642		7.7	0.62	10.1	3.1	16	298	8.6		2	20	20			80	21		14			Ariuntsetseg G et al.,2021	Lithos
701		5.042	0.59	22.77	1.99	21.5	119.91	4.23		5.32	14.7	39.75				20.02		5.34			Ariuntsetseg G et al.,2021	Lithos
652		9.144	1.268	17.216	2.476	26.132	209.49	5.683		6.331	15.541	45.737				20.045		5.103			Ariuntsetseg G et al.,2021	Lithos
